# Design of pseudosymmetric protein hetero-oligomers

**DOI:** 10.1038/s41467-024-54913-8

**Published:** 2024-12-18

**Authors:** Ryan D. Kibler, Sangmin Lee, Madison A. Kennedy, Basile I. M. Wicky, Stella M. Lai, Marius M. Kostelic, Ann Carr, Xinting Li, Cameron M. Chow, Tina K. Nguyen, Lauren Carter, Vicki H. Wysocki, Barry L. Stoddard, David Baker

**Affiliations:** 1 Department of Biochemistry, University of Washington, Seattle, WA 98195 USA; 2 Institute for Protein Design, University of Washington, Seattle, WA 98195 USA; 3 Howard Hughes Medical Institute, University of Washington, Seattle, WA 98195 USA; 4 Department of Chemical Engineering, Pohang University of Science and Technology (POSTECH), Pohang, Republic of Korea; 5 Division of Basic Sciences, Fred Hutchinson Cancer Center, Seattle, WA 98006 USA; 6 Department of Chemistry and Biochemistry, The Ohio State University, Columbus, OH 43210 USA; 7 Resource for Native Mass Spectrometry Guided Structural Biology, The Ohio State University, Columbus, OH 43210 USA

**Keywords:** Protein design, Nanostructures, Structural biology

## Abstract

Pseudosymmetric hetero-oligomers with three or more unique subunits with overall structural (but not sequence) symmetry play key roles in biology, and systematic approaches for generating such proteins de novo would provide new routes to controlling cell signaling and designing complex protein materials. However, the de novo design of protein hetero-oligomers with three or more distinct chains with nearly identical structures is a challenging unsolved problem because it requires the accurate design of multiple protein-protein interfaces simultaneously. Here, we describe a divide-and-conquer approach that breaks the multiple-interface design challenge into a set of more tractable symmetric single-interface redesign tasks, followed by structural recombination of the validated homo-oligomers into pseudosymmetric hetero-oligomers. Starting from de novo designed circular homo-oligomers composed of 9 or 24 tandemly repeated units, we redesigned the inter-subunit interfaces to generate 19 new homo-oligomers and structurally recombined them to make 24 new hetero-oligomers, including ABC heterotrimers, A2B2 heterotetramers, and A3B3 and A2B2C2 heterohexamers which assemble with high structural specificity. The symmetric homo-oligomers and pseudosymmetric hetero-oligomers generated for each system have identical or nearly identical backbones, and hence are ideal building blocks for generating and functionalizing larger symmetric and pseudosymmetric assemblies.

## Abstract (web summary)

Hetero-oligomeric proteins offer many advantages for bioengineering efforts but are difficult to make from scratch. Here, authors present a simple method for creating pseudosymmetric hetero-oligomers from input symmetrical proteins.

## Introduction

Pseudosymmetric hetero-oligomers in Nature likely evolved from homo-oligomers composed of identical subunits which, following gene duplication, underwent divergence and specialization, leading to structures where each subunit is structurally similar but can have different specialized functions^1,2^ (Supplementary Fig. 1). Specialization of binding surfaces can create hetero-oligomeric assembly hubs where each subunit can recruit a unique set of auxiliary proteins. For example, the eukaryotic pseudosymmetric heteroheptameric and heterohexameric complexes of Sm and LSm proteins, which share a common ancestor with the bacterial homohexamer Hfq^3,4^, mediate a wide network of RNA-protein interactions via specific combinations of protomers^3,5^. Another example, PCNA, acts as a homotrimeric DNA-modifying protein recruitment hub in eukaryotes but forms pseudosymmetric heterotrimers in archaea with unique protein binding sites on each subunit and a different assembly pathway^6^. More generally, evolution has used pseudosymmetrization as a route to enhance the functionality of originally homo-oligomeric assemblies. As in Nature, pseudosymmetrization of oligomers could enhance the functionality and complexity of designed protein assemblies. For example, pseudosymmetrization provides a route from designed polyhedral assemblies with triangulation number = 1 and 60 identical subunits to more complex higher triangulation number assemblies with multiple distinct subunits and much larger interior volumes for packaging cargoes^7,8^. However, while there has been considerable progress with the computational design of asymmetric protein hetero-oligomers^9–18^, with the exception of heterodimers^13^ and coiled coils^11,15–18^, there are no systematic design protocols for homogenous pseudosymmetric hetero-oligomeric protein complexes with nearly identical protomer backbones but different sequences. This is a challenging problem because for a pseudosymmetric cyclic n-mer with n distinct chains in the target ground state oligomer there are off-target assemblies on the order of n^n-1^, all with similar or identical backbone geometry. Therefore, the specificity for the intended heteromeric complex must come from the amino acid sidechain identities.

**Fig. 1:**
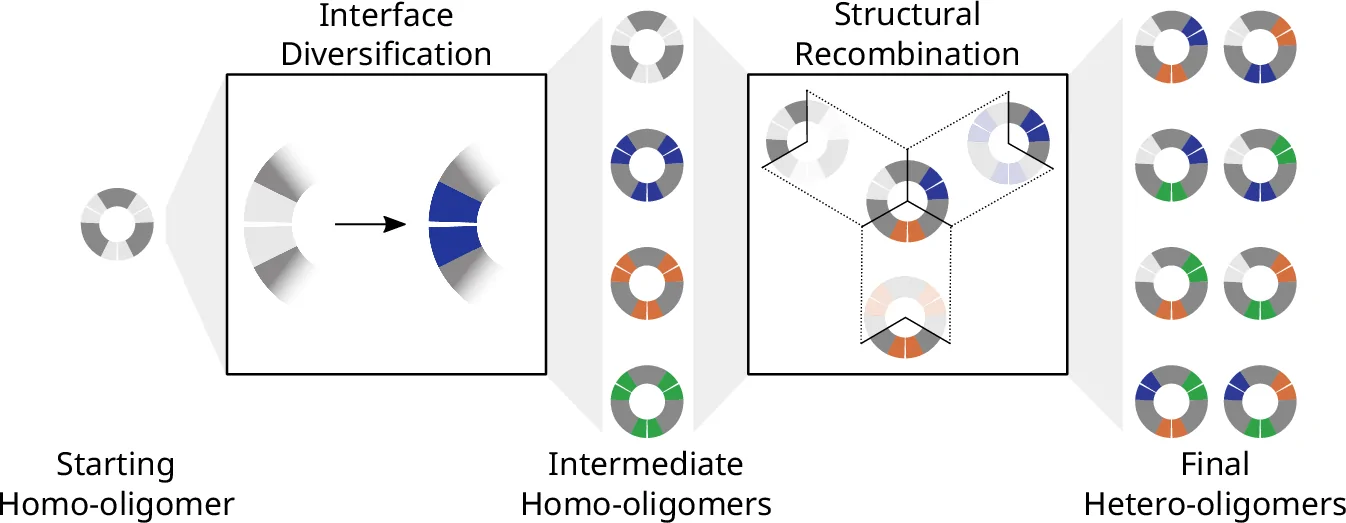
Overview of pseudosymmetric structural recombination design approach. Diagram of our stepwise hetero-oligomer creation strategy. Flowing left to right, the interface of the starting parental homo-oligomer is redesigned to generate variant homo-oligomeric interfaces, followed by junction splicing to assemble them into hetero-oligomers. Cartoons represent trimers with colored interfaces that match up (separated by a small white gap in the cartoon) and have unique sequences according to their color. Interfaces on the same chain are connected via a homology region in dark gray, which has the same sequence and structure across all protomer variants. In this strategy, the interface of an existing homo-oligomer (left; a homotrimer in this example) is redesigned symmetrically (termed Interface Diversification), with the junction regions remaining unchanged. Homo-oligomers that assemble correctly (middle column) are cut at the same point within their common homology regions and then spliced back together (termed Structural Recombination), making two-chain hybrids with two different interfaces (Supplementary Fig. 3b) which assemble into heterotrimers (right column). It is possible to create two different heterotrimers with the same interfaces by rearranging the order of the interfaces (Supplementary Fig. 3c).

We set out to develop a general de novo design approach for creating pseudosymmetric hetero-oligomers (Fig. 1) inspired by the likely evolutionary route from symmetric homo-oligomers to hetero-oligomers (Supplementary Fig. 1). We focused on architectures in which the interfaces between different subunits are structurally disjoint; in the cyclic case these correspond to circular ring-like assemblies with each subunit forming part of the arc of the ring (Supplementary Fig. 2). With these architectures, we can independently evaluate each interface in the context of individually experimentally characterized homo-oligomers. We aimed to make each of these inter-subunit interfaces distinct and specific from each other by incorporating different hydrogen bonding networks (HBNets) that work through implicit negative design; because there is a large energetic penalty disfavoring burial of polar groups, interfaces with extensive hydrogen bond networks have higher specificity than purely hydrophobic interfaces, with reduced off-target interactions^19^. HBNets have been used previously to create a large number of orthogonal heterodimeric interfaces on helical bundle backbones^9^.

In this work, we developed a stepwise design strategy for generating pseudosymmetric hetero-oligomers with distinct HBNets at each subunit-subunit interface (Fig. 1). First, we diversify the interface of a homo-oligomeric scaffold and experimentally characterize multiple variants, each with distinct HBNets at the inter-subunit interface^9,19^, increasing the variety of HBNets that can be incorporated by introducing backbone perturbations in the secondary structure elements present at the interface^20^. Second, we structurally recombine experimentally validated homo-oligomers to generate pseudosymmetric hetero-oligomers. This divide-and-conquer approach avoids the highly error-prone approach of designing multiple interfaces simultaneously (i.e. one-shot design) and also generates a large number of hetero-oligomers through the structural recombination of a small set of homo-oligomers (Supplementary Fig. 3a).

## Results

### Pseudosymmetrizing a computationally designed 9-repeat toroid

**Fig. 2:**
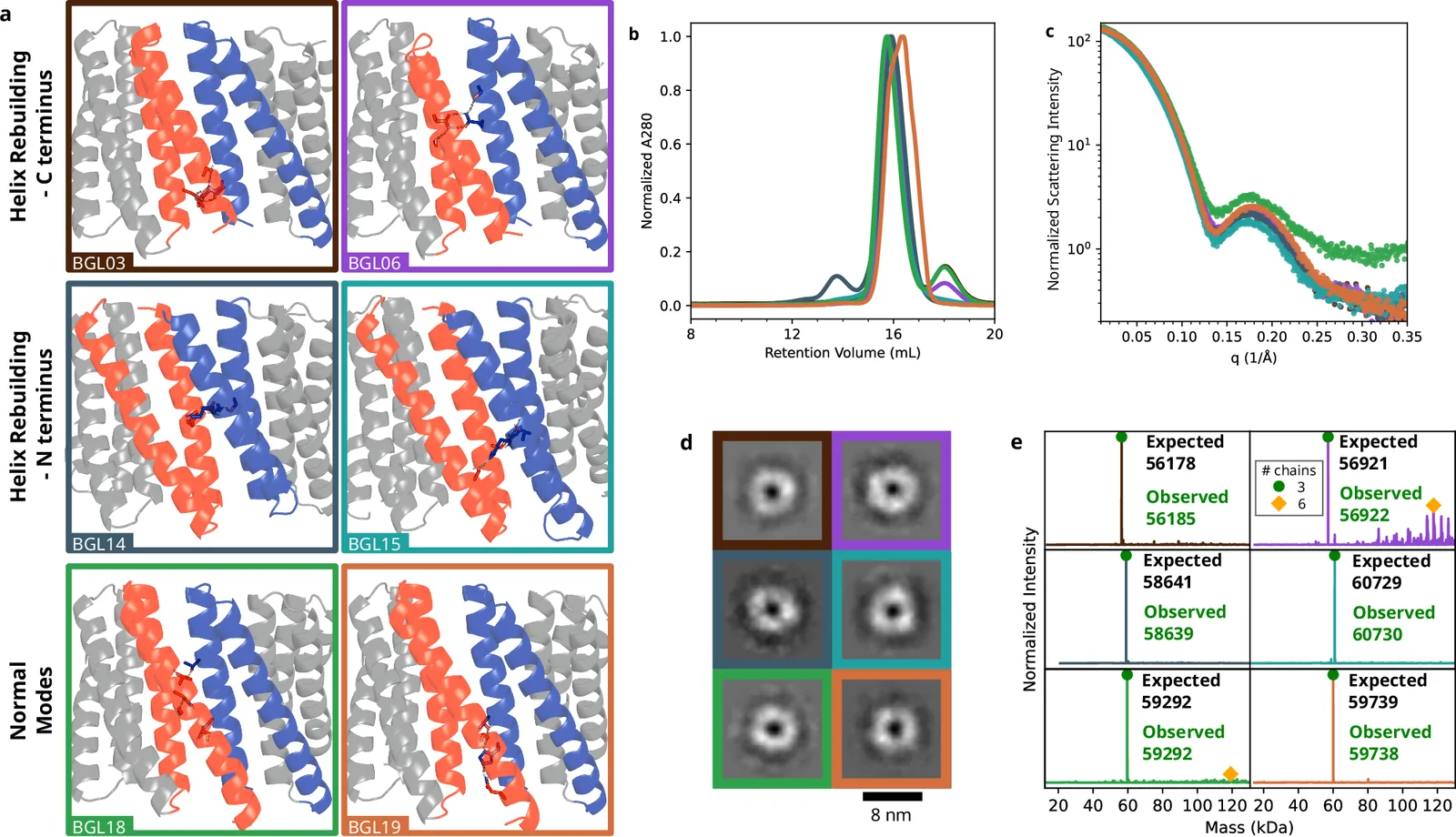
Characterization of BGL homotrimers. **a** Cartoon representation of six representative successful redesigns of BGL0, with two from each design set: Helix Rebuilding plus C-terminal attachment (HR-C), Helix Rebuilding plus N-terminal attachment (HR-N), and Normal Modes (NM) relaxation. Blue and orange colored backbones highlight N- and C-terminal two-helix segments comprising the interface, which was redesigned, and newly designed hydrogen bond networks (HBNets) are shown in sticks with the hydrogen bonds in dashed lines. Design names in the bottom left corner of each box and box outline and trace colors are used consistently throughout. Characterization of the six examples from (**a**) by (**b**) SEC, (**c**) SAXS, (**d**) nsEM, and (**e**) nMS. **b** Overlay of SEC traces normalized to maximum absorbance at 280 nm (A280), after soluble aggregate removal and TEV cleavage. SEC traces of all 20 designs are provided in Supplementary Fig. 4. **c** Overlay of SAXS data. Data were scaled appropriately to align at *q* = 0.01 to highlight the similarity of the profiles. **d** Individual 2D class averages of nsEM data for each sample. **e** Individual mass deconvolved nMS data of TEV-cleaved samples (Supplementary Data 13). All peaks that match the expected mass in Daltons for each homotrimer are annotated with green circles, and homohexamers (likely to be dimers of trimers) are annotated with orange diamonds.

We chose as a first model system a circular Tandem Repeat Protein or cTRP composed of three identical subunits forming a ring (Supplementary Fig. 2a, Supplementary Note 1). The interface between subunits of this ring bears a strong resemblance to the helical bundles used with HBNet previously^9,19^, giving confidence in the applicability of the method here. Because Rosetta’s HBNetMover is very sensitive to small backbone displacements^20^, we enhanced HBNet sampling without drastically altering the interface by generating small backbone variations at the interface using normal modes (NM) relaxation^21^ restricted to the interface (NM design set) or by complete backbone resampling of the C-terminal helix which is on the outer ring of helices and forms the interface in conjunction with two inner ring helices. In the latter resampling, we varied the internal geometry of the helix using the Crick alpha-helix parameters^22^ starting from the closest fit parameters to the C-terminal helix and varying r_0_, ω_0_, ω_1_, φ_0_, φ_1_, and ∆z. With a new loop, we rejoined the new helix to the C-terminus (HR-C design set) or, in an effort to add an additional implicit negative design element, attached it anew to the N-terminus (HR-N design set). The HR-N design set was expected to be orthogonal to the parental design and the HR-C and NM designs (which retain the C-terminal outer helix) due to steric hindrance imposed by mismatching terminal helices (clashing in one case, a large gap with reduced hydrophobic contacts in the other). Following backbone perturbation, HBNets were installed into backbones using HBNetMover (Fig. 2a), and the sequences of the helices comprising the interface were redesigned with flexible backbones using FastDesign^23^.

We obtained synthetic genes encoding 20 His6-tagged redesigns of BGL0 (named BGL01-20; Supplementary Data 1) and BGL0 (Supplementary Note 2) and produced the proteins in *E. coli*. The proteins were purified by immobilized metal affinity chromatography (IMAC) and size-exclusion chromatography (SEC), the His6-tags were cleaved by TEVp^24^, and the cleaved His6-tags and His6-tagged-TEVp were removed with a second round of IMAC (Supplementary Fig. 4, Materials and Methods). Out of the 20 purified redesigns, 14 (NM: 3/4; HR-N: 3/8; HR-C: 8/8) had a prominent peak near the expected retention volume for the trimeric species according to SEC (Fig. 2b, Supplementary Fig. 4, Supplementary Data 2), with varying degrees of heterogeneity. Small-angle X-ray scattering (SAXS)^25,26^ analysis of the prominent peak (except for BGL13 which aggregated) revealed that 12 samples (NM: 3/4; HR-N; 3/7; HR-C 6/8) have radii of gyration (Rg) and molecular weights (MW) close to the expected values and for these the experimental scattering profiles agreed well with the predicted scattering profiles based on the design models (Supplementary Data 2, Supplementary Data 3). The realspace P(r) transformations of the raw data for the passing designs show distance distributions which are slightly right-shifted compared to the expected distances based on the design models, including for BGL0, suggesting a small global ‘swelling’^27^, and large D_max_ and non-gaussian Kratky plots for most samples also indicate different degrees of particle flexibility (Supplementary Data 3). The overlay of intensity-normalized SAXS curves for two representative samples from each of the three design sets (six total; NM: BGL18, BGL19; HR-N: BGL14, BGL15; HR-C: BGL03, BGL06) illustrate the structural similarity of the redesigns (Fig. 2c). The general shape of these six representatives was confirmed by the appearance of small donut-shaped particles on negative stain electron microscopy (nsEM) 2D class averages (Fig. 2d, Supplementary Fig. 5). Of these 12 SAXS-verified redesigns, all were confirmed to be mostly trimeric by native mass spectrometry (nMS)^28,29^ (Fig. 2e, Supplementary Fig. 6, Supplementary Data 2). SEC-Multi-Angle Light Scattering (SEC-MALS) similarly indicated that all 12 samples are primarily trimeric, although measured masses skew smaller than expected (Supplementary Fig. 7). For some samples, nMS and/or SEC-MALS suggested the presence of minor populations of alternative species like dimers or tetramers that may incorporate different numbers of subunit in the ring (BGL02, BGL08, BGL19) or hexamers that could arise from weak non-specific surface interactions between trimeric complexes (BGL06, BGL18) (Supplementary Data 2).

**Fig. 3:**
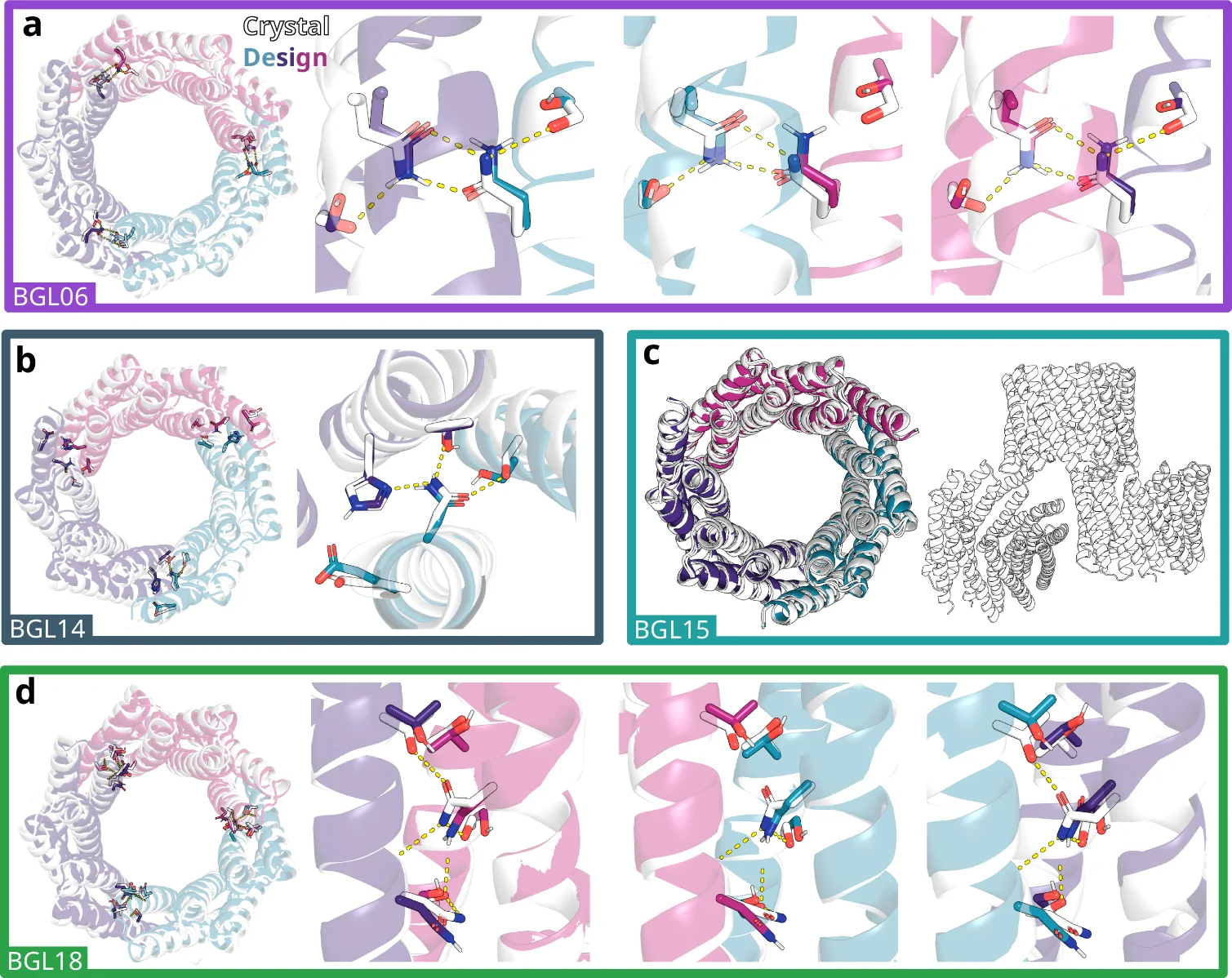
Crystal structures of BGL homotrimers. **a**, **b**, **d** Crystal structures (white) of four different BGL homotrimers are superimposed onto the design models colored in teal, purple, and magenta. (left) The top-down view of the full ring with HBNets highlighted and visible through transparent cartoon models. The backbones of all crystal structures match well with the design models (<1.4 Å RMSD). (Right) Close-up view(s) of each HBNet well enough resolved to build full side chains. **a** BGL06 (2.1 Å resolution; RMSD = 1.2 Å over all 483 Cα atoms; PDB: 8E0L) and (**d**) BGL18 (3.0 Å resolution; RMSD = 1.4 Å over all 513 Cα atoms; PDB: 8E0N) were both well enough resolved to model all three interfaces. BGL18 contained two copies of the full trimer in the crystal unit cell, which are structurally similar to each other (RMSD = 0.3 Å over all 511 Cα atoms between copies). **b** One interface of BGL14 (3.0 Å resolution; RMSD = 1.3 Å over all 487 Cα atoms; PDB: 8E12) was well resolved. **c** BGL15 (4.0 Å resolution; PDB: 8E0M) had three copies of the trimer in the unit cell and all three were close to the design model (0.6 Å (519 residues), 0.6 Å (519 residues), and 0.7 Å (519 residues) RMSD over Cα atoms).

We obtained crystal structures for representatives of each design set: BGL06 from the HR-C set (Fig. 3a), BGL14 and BGL15 from the HR-N set (Fig. 3b,c), and BGL18 from the NM set (Fig. 3d, Supplementary Table 1, Supplementary Fig. 8). At the backbone level, the crystal structures show good agreement with the design models (Cα-RMSDs of 1.2 Å over 483 residues, 1.3 Å over 487 residues, 0.6 Å over 519 residues, and 1.4 Å over 513 residues, respectively). Sidechains at the interfaces of BGL06, BGL14, and BGL18 are well enough resolved to determine that HBNets in all three structures are formed correctly. BGL06 has an interchain bidentate HBNet involving Gln14 and Gln146, each backed up with second shell interactions from Ser18 and Ser125, respectively (Fig. 3a). The string of hydrogen bonds in the BGL14 interface alternates between chains, with a fully satisfied Asn13 in the very middle which donates a hydrogen bond to His153. BGL15 has three copies of the ring in the unit cell, but the low resolution of the dataset (4.0 Å) obscured details about the HBNet residue sidechains. The other two copies had Cα-RMSDs of 0.6 Å over 519 residues and 0.7 Å over 519 residues to the design model. BGL18 has two copies of the ring in the unit cell, and all six interfaces were resolved and highly similar. The other copy of the trimer in the unit cell is 1.6 Å Cα-RMSD over 513 residues to the design model. It has a generally polar interface with a meandering HBNet spanning Asn10, Thr17, Asn126 (which may be donating a hydrogen bond to the backbone oxygen of Asn10), Thr152, Ser155, and Ser159.

We constructed multiple pseudosymmetric trimers by selecting three homotrimers at a time from the 13 validated designs (12 redesigns plus the original) and structurally recombined them, as illustrated in Fig. 1 and Supplementary Fig. 3. Each subunit was split in the middle at Arg82, which is far from either interface. Complementary pairs were then reconnected at the splicing junction to form hybrid protomers (Supplementary Fig. 3b) which, in principle, can assemble into a heterotrimer. Two distinct heterotrimers can be generated from each selected set of homotrimeric interfaces (*a*, *b*, and *c*) by changing the order of structural recombination along the circle: (*a*-*b*; *b*-*c*; *c*-*a*) and (*a*-*c*; *c*-*b*; *b*-*a*) (Supplementary Fig. 3c). This procedure can generate considerable diversity. The number of possible hetero-n-mers grows very quickly with the number of validated homo-n-mers from which the interfaces are extracted (Supplementary Fig. 3a); there are

1 $$\frac{k!\left(n-1\right)!}{n!\left(k-n\right)!}$$

ways to combine *k* interfaces into a hetero-n-mer with n different interfaces (Eq. 1). With *k* = 13 interfaces to choose from for a heterotrimer (*n* = 3), there are 572 possible heterotrimeric variants that could theoretically be generated. We tested two different strategies for choosing interfaces for structural recombination. First, to attempt to maximize binding specificity, we constructed a set of six heterotrimers (called Set 1) with one interface from each of HR-N, HR-C, and NM. Second, we enumerated all eight possible combinations of BGL0 and the three validated homo-trimers from the minimally-perturbed NM set (BGL17, BGL18, and BGL19) to generate heterotrimers with nearly identical subunit backbone structures (called Set 2).

**Fig. 4:**
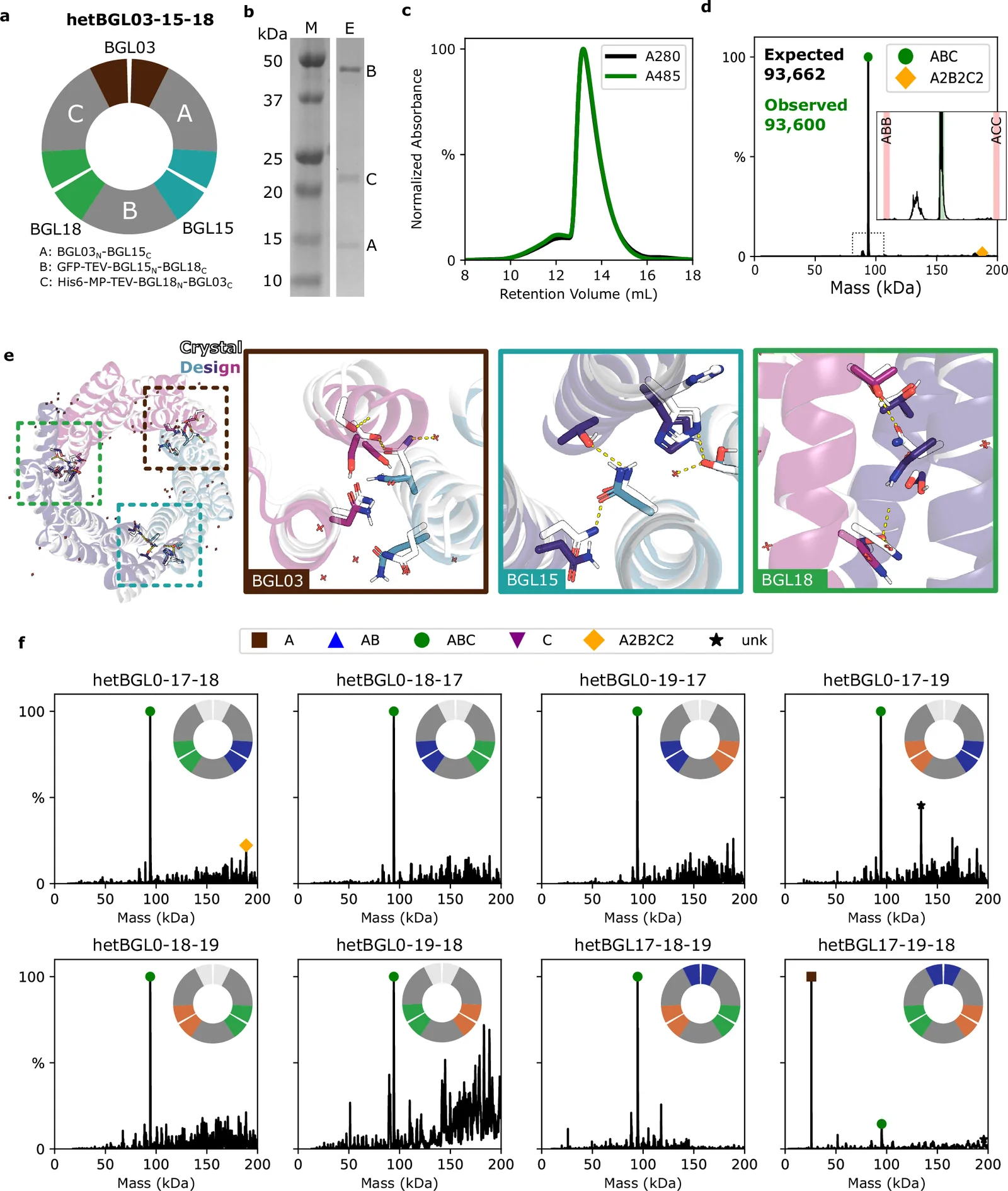
Characterization of hetBGL heterotrimers. **a**–**e** Results of hetBGL03-15-18. **a** (top) Schematic showing the result of structural recombination between BGL03, BGL15, and BGL18. (bottom) The expression constructs for each chain as expressed tricistronically. GFP is superfolderGFP^69^, included to add a large amount of mass and provide an additional spectroscopic identification, and MP is EHEE_rd2_0005^30^, a stable and small protein included to add a small amount of mass. **b** SDS-PAGE of IMAC elution. M: protein ladder. E: IMAC elution. Staining intensity follows the trend expected for three proteins of very different masses at equal abundance (larger proteins binding more stain). Representative image showing similar results found from three separate purifications. Source data are provided as a Source Data file. **c** SEC trace overlaying normalized absorbances at A280 and A485. **d** Mass deconvolved nMS data showing all annotated species (Supplementary Data 13). The insert plot is the zoomed-in region indicated by the dashed box on the main plot, showing a 24 kDa wide region centered on the ABC peak on the x-axis and with signal intensity between 0% and 10% on the *y*-axis. Shaded regions in red indicate the expected locations for off-target trimers. **e** (left) Crystal structure in white (2.1 Å resolution, PDB: 8E0O) and design model in teal, purple, and magenta overlaid to show global agreement (Cα-RMSD: 1.2 Å over 506 residues, TM-score: 0.96). Dashed boxes indicate approximate locations of zoomed-in views of the interfaces derived from BGL03, BGL15, and BGL18, showing side chains of HBNet residues and their inferred hydrogen bonds as yellow dashes. **f** Individual mass deconvolved nMS data for all hetBGL Set 2 samples (Supplementary Data 13). Peaks labeled unk are unknown species believed to be distinct from noise.

To favor the formation of pseudosymmetric heterotrimers, we used a co-expression and co-purification strategy, which would enrich for the correctly assembled complexes. Both sets of designed heterotrimers (total 14; Supplementary Data 4) were ordered as tricistronic constructs with TEVp-cleavable GFP or miniprotein^30^ tags on two of the chains to differentiate their masses by SDS-polyacrylamide gel electrophoresis (SDS-PAGE) and nMS, and the third chain (expressed last on the transcript to be in limiting concentration) having a His6-tag for affinity co-purification (Fig. 4a). We found that 11/14 co-purified with three distinct bands on SDS-PAGE, with nine qualitatively showing roughly stoichiometric ratios between bands (Fig. 4b, Supplementary Fig. 9, Supplementary Data 5). All 14 samples were subjected to SEC analysis, and nine (two from Set 1 (hetBGL03-15-18 and hetBGL08-09-19) and seven from Set 2) were found to have distinct main peaks near the expected retention volume (Fig. 4c, Supplementary Fig. 10, Supplementary Data 5; this roughly aligned with the SDS-PAGE results). Analysis of the main assembly peaks from SEC by nMS showed that all samples contained the intended ABC species (consisting of the three unique chains: A, B, and C) without any of the many possible off-target trimeric combinations of subunits (AAB, BBB, etc.; Fig. 4d, f; Supplementary Fig. 11). Several pairs of designs, for example, hetBGL03-18-15 and hetBGL03-15-18, share the same interfaces arranged in a different order but display markedly different biophysical properties; hetBGL03-15-18 elutes earlier than expected with a right-hand tail (suggesting some polydispersity) and only a slight left-hand shoulder on SEC and contains an A2B2C2 species by nMS (together suggesting a transient dimerization of the ABC particle) while the hetBGL03-18-15 arrangement has considerably lower expression and elutes at the expected volume with more prominent shoulders and an aggregate fraction. Overall, eight designs (two from Set 1 and six from Set 2) were confirmed to be well-behaving heterotrimers (Supplementary Data 5).

We solved the crystal structure of hetBGL03-15-18, a heterotrimer from Set 1 composed of the interfaces extracted from BGL03, BGL15, and BGL18, at 2.1 Å resolution (Fig. 4e, Supplementary Table 1, Supplementary Fig. 8). The structure superimposes to the design model with 1.2 Å Cα-RMSD between 506 residues. The TMscore (TM-align version 20220412)^31^ between the single-chain versions of hetBGL03-15-18 and the symmetric parental BGL0 complex was 0.94 (Cα-RMSD of 1.7 between 501 residues) with a sequence identity of only 62.3%, showing that this method can produce highly pseudosymmetric hetero-oligomers. The BGL18-derived HBNet is very similar to that in the homotrimer crystal structure, and the HBNet of BGL15, which was not resolved in the homotrimer crystal structure, is confirmed to be in the correct conformation, with the addition of a hydrogen bond between Ser140 and a crystallographic water. The previously unsolved BGL03 interface differs from the design model due to fraying of the C-terminal outer helix, likely due to the reduction of hydrophobic packing stemming from polar HBNet residues placed too close to the C-terminus; placement of HBNet residues too close to the termini was avoided in future designs. To structurally characterize and compare the Set 1 designs, we obtained SAXS profiles for hetBGL03-15-18, hetBGL03-18-15, and hetBGL08-09-19 which show some deviation from the predicted profiles with large Rg and some disorder as indicated by the Kratky plots, but with overall similar features in the raw profile and P(r) plots compared to each other (Supplementary Data 6) and the SAXS data of the constituent BGLs (Supplementary Data 3).

Each subunit of a heterotrimer from Set 1 has a different number of helices due to the use of interfaces from the HR-N set with interfaces from the HR-C or NM sets. To generate more topologically pseudosymmetric designs with termini positioned more usefully for structural engineering applications^7^, we made new versions that were more like the subunits of heterotrimers from Set 2 (Supplementary Fig. 12a), which have the same number of helices in each subunit by deleting the loop of the N-terminal outer helix in the HR-N-derived interface and reconnecting it at the C-terminus of the next protomer (essentially converting the HR-N interface to HR-C). The relooped versions of hetBGL03-15-18 and hetBGL08-09-19 remained heterotrimeric (Supplementary Fig. 12b-d) while the relooped version of hetBGL03-18-15 exhibited non-specific assembly, further indicating the incompatibility of this interface ordering. This brought the number of validated hetBGLs to 10 (Supplementary Data 5).

### Pseudosymmetrizing a computationally designed 24-repeat toroid

**Fig. 5:**
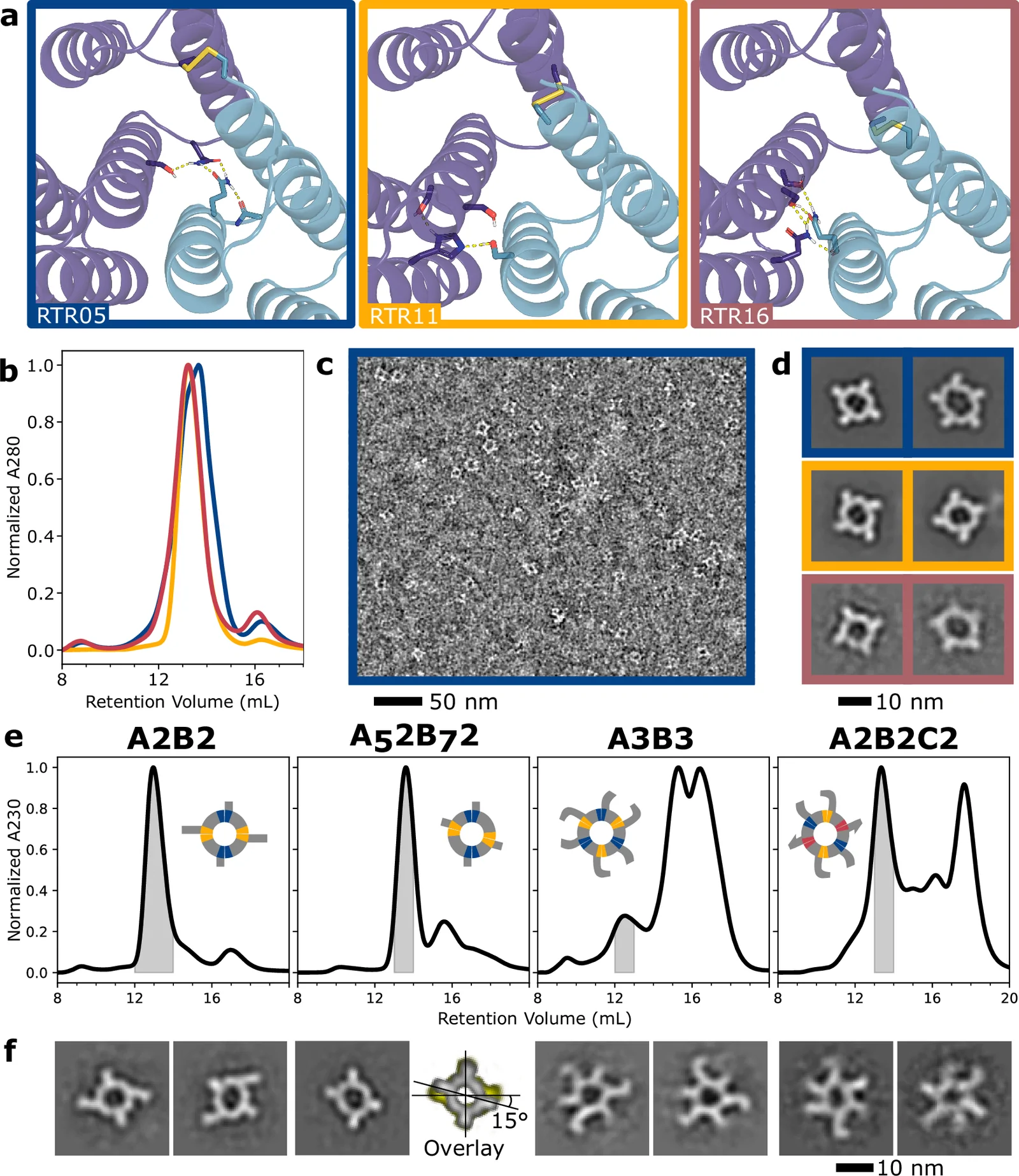
Characterization of RTR homotetramers and hetero-oligomers. **a** Cartoon representation of three representative successful redesigns of RTR0. Blue and purple colored backbones represent N- and C-terminal interfaces, respectively. HBNets and disulfide bonds are shown in sticks with hydrogen bonds in dashed lines. Design names (bottom left corner of each box) and box outline and trace colors are used consistently throughout to represent the same interfaces. Characterization of the three examples from (**a**) by (**b**) SEC, **c** nMS, **d** SAXS, and (**e**) nsEM. **b** Overlay of A280 normalized SEC traces. **c** Cropped view from one of 173 micrographs of RTR05 collected from a single sample. **d** Individual 2D class averages of nsEM data collected on each sample. The outline color corresponds to the sample ID. **e** SEC traces (normalized absorbance at 230 nm) of four hetRTRs: (left to right) A2B2-hetRTR05-11_noCys_, A_5_2B_7_2-hetRTR05-11_noCys_, A3B3-hetRTR05-11_noCys_, and A2B2C2-hetRTR11_noCys_-05-16. Inset with cartoons representing the expected oligomer shapes. Shading indicates the region used for nsEM analysis. **f** Pairs of 2D class averages matching the SEC traces above, except A_5_2B_7_2-hetRTR05-11_noCys_ which shows one 2D class average and an overlay of the 2D class with a 2D class of RTR11 to show the angular shift.

Next, we tested whether our hierarchical pseudosymmetrization method could be generalized to another homo-oligomeric system. We selected a designed tetrameric toroid with 24 internal repeats separated into four protomers of six repeats each (Fig. 2b, Supplementary Note 1). Since there are multiple ways of factoring 24 repeats into protomers, this system can be readily redesigned to generate C3, C4, C6, and C12 assemblies (Supplementary Fig. 13a), offering multiple possibilities for hetero-oligomerization (trimers: ABC; tetramers: A2B2, ABCD; hexamers: A3B3, A2B2C2, ABCDEF; etc.). Based on the success of the NM set of the BGL system, we only used NM perturbation with the RTR system. HBNets and the surrounding sequences at the interfaces were designed similarly to BGL0, with the exception of avoiding the addition of HBNet residues near the ends of the N-terminal helix. We also installed disulfide bonds at diverse positions^32^, since interface disulfides were required for the original toroid to form tetramers and higher-order symmetries^33^; the disulfides could also provide an additional level of specificity (Fig. 5a).

We tested 24 redesigns of RTR0, called RTR01 through RTR24, as homotetramers (Supplementary Data 7). For 10 out of 24 redesigns, SEC profiles contained a peak near the expected retention volume for a tetramer (Fig. 5b, Supplementary Fig. 14, Supplementary Data 8). According to nMS, all samples contained a monomeric species but the primary oligomeric species for eight of those designs were homotetrameric (Supplementary Fig. 15, Supplementary Data 8) and, for seven of these, SAXS Rg values and profiles were consistent with the design models (Supplementary Data 9). Kratky analysis showed considerable disorder for all samples, likely due to the presence of disordered purification tags, and the P(r) plots also suggested the presence of a more compact, likely monomeric, species in most samples. In contrast to the other redesigns, RTR11, RTR18, and RTR24 were found to remain assembled under reducing buffer conditions by SEC and SAXS (Supplementary Fig. 14b, Supplementary Data 9), so their cysteines were mutated to alanines in some later designs involving these interfaces to reduce the risk of disulfide-locked off-target species. Arm-like studs protruding from the C-terminus of each subunit (Supplementary Fig. 2b)^34^ could easily be identified by nsEM and facilitated the determination of the oligomeric states (Fig. 5c,d, Supplementary Fig. 16). The 2D class averages of RTR0 show a nearly perfectly circular ring with four studs spaced equally around the circle, corresponding to a top-down view of the design model along its symmetry axis (Supplementary Fig. 16). By nsEM, RTR05 and RTR16 contained a population of pentamers with larger rings and five studs (Fig. 5d); native mass spectrometry did not identify any species corresponding to pentameric RTR16.

The larger size of the RTR system makes it readily characterizable by nsEM, which has the advantage over other techniques of directly identifying both the oligomeric state and the shapes of the species present in a small amount of sample (with the caveat that the particles may be affected by stain or grid surface interactions and monomers may be too small to resolve). However, any pseudosymmetric hetero-oligomers built from the base RTR homo-oligomers would be indistinguishable by nsEM as they would have the same overall shape. To enable disambiguation of the chains, we diversified the shape of the RTRs by either extending the existing designed helical repeat (DHR) extensions, attaching DHR-DHR junctions with diverse shapes^35^ to the C-terminus, or using HFuse^36^ to attach miniproteins^37^ into the lumen on the N-terminus. We characterized these as extensions of RTR24 or RTR11 and found 5 (RTR24_arm5, RTR24_DHR52, RTR24_DHR70_1, RTR24_DHR80_1, and RTR24_DHR81) to have approximately the correct structure and visually distinct shapes (Supplementary Note 3); by construction, the extensions should be cross-compatible with all RTR variants.

For hetero-oligomer demonstration we chose five redesigned homo-oligomers based on maximizing HBNet and disulfide bond placement diversity: RTR05, RTR11_noCys_ (a variant where all Cys were changed to Ala), RTR16, RTR18_noCys_, and RTR24_noCys_. We assessed their pairwise interface compatibility by building the full set of ten C2-symmetric A2B2 heterotetramers plus two others using the original Cys-containing interface variants of RTR11 and RTR18 (Supplementary Data 10) via the same structural recombination protocol used for the BGLs, using Leu98 as the splicing junction. We designed the A2B2 heterotetramers so that each unique chain displayed a different C-terminal extension and were tagged with either GFP or a His6-tag. Many of the designs had multiple peaks on SEC (Fig. 5e, Supplementary Fig. 18), but the peaks containing GFP (according to absorbance at 485 nm) in ten of the designs, including A2B2-hetRTR05-11, A2B2-hetRTR05-11_noCys_, A2B2-hetRTR05-16, and A2B2-hetRTR11_noCys_-16, were found to contain the intended A2B2 heterotetramers and no other tetrameric species according to nMS (Supplementary Fig. 19, Supplementary Data 11; some showed potential partial assemblies, species with three copies of chain A (A3), and tetramer-dimers). The A2B2 species of A2B2-hetRTR05-11_noCys_ was clearly identified by nsEM as indicated by the C2 symmetry of the radial extensions (Fig. 5f, Supplementary Fig. 20) and, while the homotetrameric RTR05 and RTR16 samples partially formed pentamers, no obvious off-target pentameric species were observed. This suggests that the added specificity constraints of heteromeric interactions may prevent off-target assemblies. This has also been observed in heteromeric assemblies of native LSm proteins, which have reduced variation in ring size compared to related homomeric assemblies^4^.

We reasoned that the A2B2 complex should assemble successfully as long as the final assembly contained 24 total repeats in the ring, so to test this, we modified the protomers to have one less or one more ring repeat (Chain A: 6 changed to 5; Chain B: 6 changed to 7; 5 × 2 + 7 × 2 = 24; these subunits were sufficiently differentiated in mass for identification by nMS so the GFP mass tag was omitted). Out of six tested designs (Supplementary Data 10), A_5_2B_7_2-hetRTR05-11_noCys_ and A_5_2B_7_2-hetRTR11_noCys_-16 still assembled (Fig. 5e, Supplementary Fig. 18b, Supplementary Fig. 19b, Supplementary Data 11), and nsEM of A_5_2B_7_2-hetRTR05-11_noCys_ revealed the studs shifted by about 15°, as expected for rotation by a single repeat (Fig. 5f, Supplementary Fig. 20).

To explore the creation of oligomers with different numbers of subunits, we designed homomeric C3, C6, C8, and C12 versions of the RTR toroid (Supplementary Data 7). The C3 versions assembled as desired, but complexes with C6 symmetry and above assembled into a range of oligomers with different numbers of subunits (Supplementary Fig. 13, Supplementary Fig. 15, Supplementary Data 9). As noted above, we hypothesized that the heteromeric versions of these assemblies might better assemble into the target stoichiometries than their homomeric counterparts. Therefore, we created heterohexameric rings using the structural recombination approach, which combined either two different interfaces into A3B3 hexamers or three interfaces into A2B2C2 hexamers, employing the interfaces found to be successful in the A2B2 designs. The same hybrid protomers could be used in both A3B3 or A2B2C2 hexamers as the combination of interfaces programs the final assembly, and hence we expressed these subunits separately, with either GFP, a His6-tag, or no additional tag, and mixed prior to IMAC (Supplementary Data 12). As indicated by SEC, four of the 12 A3B3 combinations (A3B3-hetRTR05-11_noCys_, A3B3-hetRTR11_noCys_-16, A3B3-hetRTR11_noCys_-24_noCys_, and A3B3-hetRTR11_noCys_-0) and two of the four A2B2C2 combinations (A2B2C2-hetRTR11_noCys_-05-16 and A2B2C2-hetRTR11_noCys_-05-0) produced assemblies, though all contained large partial- or non-assembly peaks (Supplementary Fig. 21, Fig. 5d, Supplementary Data 11). A3B3-hetRTR05-11_noCys_ was verified by nMS (Supplementary Fig. 19c), and the expected ring shape with two different alternating extensions was observed by nsEM (Fig. 5f, Supplementary Fig. 20). Due to difficulties producing samples at sufficient concentrations for nMS, we were only able to confirm one A2B2C2 assembly by nsEM, A2B2C2-hetRTR11_noCys_-05-16, where three different extensions with the intended circular arrangement can be observed (Fig. 5f, Supplementary Fig. 20). In both cases, the heterohexameric designs were found to be homogenous in their oligomeric state, in contrast with the polydispersity found in their homohexameric parental designs.

## Discussion

The pseudosymmetric designs presented here go beyond previous design work and almost all the heterotrimers in nature by having subunits with nearly identical backbones but distinct sequences. This has been a challenging design problem because almost all the interaction specificity must come from sidechain-sidechain interactions rather than inter-subunit shape complementarity. Pseudosymmetry enables interconversion between homo-oligomeric and hetero-oligomeric forms by simple sequence substitution, which allows considerable compositional diversification, as illustrated by the C6, A3B3, and A2B2C2 versions of the RTR toroid, and is critical for building larger pseudosymmetric materials, as discussed below. Heterodimeric and heterotrimeric coiled-coils have been described with subunits with nearly identical backbones^15,17^, but these can be difficult to extend laterally to be used as components of larger globular assemblies. The pseudosymmetric designs presented here enable the modular construction of a wide variety of protein complexes with different component stoichiometries, chain identities, shapes, and spacings of their termini, all from a small set of validated components, providing a wide range of diverse hubs for target clustering in cell signaling and building blocks for multicomponent materials^36,38–43^.

In the one-shot creation of new hetero-oligomeric assemblies with the simultaneous design of more than one interface, the failure of a single interface to assemble correctly leads to the complete failure of the design, even if the other interfaces form correctly. Our stepwise homologous recombination-inspired design strategy considers interface formation separately from interface specificity, allowing the identification and discarding of problematic interfaces so that they do not poison otherwise good assemblies as they would in a one-shot design. Our success rate of 24 experimentally-verified hetero-oligomers out of 51 total tested designs (10/17 hetBGLs and 14/34 hetRTRs; Supplementary Data 5, Supplementary Data 11) is higher than previous studies, despite the structural similarity between the subunits of each system. While HBNets can make important contributions to specificity, and the Rosetta HBNetMover can efficiently design them, newer deep-learning techniques could be used instead during interface redesign. For example, partial diffusion with RFdiffusion could be used for backbone diversification while accurately preserving residues important for protein function (such as in enzymes)^44^ and/or hydrogen bond networks could be installed using ProteinMPNN with upweighted polar residues^45^. Although we applied our pseudosymmetrization strategy to all-helical de novo designed assemblies, the approach could be applied to natural homomeric proteins of interest to aid in their characterization^13^ or introduce new functionalities for therapeutic applications, for example, making specifically assembling hetero-oligomeric TNF ligands to induce novel signaling responses^46^.

As noted above, pseudosymmetry provides a means to easily transition between simple homo-oligomeric systems and more complicated hetero-oligomeric systems. Fusions or other modifications to hetero-oligomers can first be prototyped using their homo-oligomeric paralogs, which are easier to generate and validate (Supplementary Fig. 17). Homo-oligomers with pseudosymmetric variants can similarly be used for material design by initially building higher-order symmetric assemblies out of homo-oligomers and, as a second step, breaking symmetry by substituting the homo-oligomers for their pseudosymmetric hetero-oligomeric cousins, which enables the specialization of a subset of the interactions while preserving the interaction geometry between the pseudosymmetric building blocks. This opens up routes to more complex protein nanomaterials; for example, we used the BGL homotrimers to design simple tetrahedral, octahedral, and icosahedral nanocages, which we then transformed into more complex T = 4 nanocages by substituting in hetBGL trimers to desymmetrize the nanocage, preserving the interface geometries of two of the interfaces and modifying the third to interact with a new homotrimer to increase the triangulation number, physical size, and complexity of the nanocage^7^. The BGL system of homo- and hetero-oligomers has worked well as building blocks for the construction of larger assemblies, possibly due in part to small inherent conformational flexibility as observed by SAXS enabling subunit exchange and rearrangement on reasonable timescales despite the high cooperativity of such assemblies.

Beyond the design of pseudosymmetric nanocages for applications in vaccinology and cellular delivery, our heterotrimers have numerous possible applications in cell biology. For example, PROTAC^47^ and LYTAC^48,49^ approaches for targeting proteins for degradation could be enhanced by attachment of the separate protein-targeting and degradation-meditating domains to separate hetBGL or hetRTR subunits, allowing targeting of multiple endogenous proteins or the coupling to more complex molecular logic systems for tighter control over activity^50^. The RTR designs could be used to precisely geometrically control the spacing, orientations, and valency of any combination of several receptor binders to generate novel signaling molecules and help decipher the geometric constraints associated with the signaling processes involved in T-cell activation^43^ or cell differentiation^38,41,42,51^.

## Methods

### HBNet design

Using Rosetta 2018.41.post.dev+171.bcov.dev.507725abe46, we generated about 4 k different backbones using the HR and NM methods described above and in the Computational Methods section of the Supplementary Methods for the BGL0 scaffold and about 15 k backbones for the RTR0 scaffold. HBNet design was carried out differently for each scaffold set to produce good diversity in each case, but typically we restricted HBNet design to the region comprising the interface and required at least one intermolecular hydrogen bond (hbond) between residues that are buried and have second-shell hbonds to enforce the sidechain conformation. Due to the large number of backbones, we used MC-HBnet sampling to speed up the search, taking between 10 k and 100 k MC steps per input. HBNets created using backbones from NM perturbation were highly redundant due to the similarity of input backbone conformations, so outputs were clustered according to the buried HBNet residues and the best scoring outputs were kept for each cluster. See the Computational Methods section of the Supplementary Methods for further details.

### Interface design

Flexible backbone Rosetta FastDesign was performed with the standard suite of TaskOperations (InitializeFromCommandline, DesignRestrictions to specify surface/interface/core layers, IncludeCurrent, LimitAromaChi2, and ExtraRotamersGeneric). Specific design details vary by scaffold (and Set), but all methods restrict movement to the interface only by using coordinate constraints to hold the non-interface regions in place, using a 7.0 Å neighborhood or InterfaceByVector to define the interface region. Surfaces were specifically designed so that both halves of the protein subunit separately have low (<7) isoelectric points to avoid issues of high pI in hybrid chains causing insolubility. Disulfide bonds were designed into the interfaces of the RTR0 redesigns either using Stapler^32^ or manually using PyMol^52^.

### Filtering

Designs were filtered similarly to Chen et al.^9^ to have predicted DSSP^53^ (‘mismatch_probability’), voids near the interface and HBNet residues, interface shape complementarity, and secondary structure chunk shape complementarity not worse than the original scaffold. We selected designs with no buried unsatisfied hydrogen bond acceptors, good sidechain pre-organization (low RotamerBoltzmannWeight), and with fragments that are well represented in the PDB according to the worst9mer filter. These were cartesian relaxed first with AtomPair constraints from HBNet, then without, and designs whose constraint score increased significantly with unconstrained relaxation were removed. RTR designs were also filtered on DeepAccNet^54^. All designs were visually inspected using PyMol for good hydrogen bonding and hydrophobic residue interdigitation before ordering. See the Computational Methods section of the Supplementary Methods, filtering subsections, for more scaffold-specific information.

### Computational recombination to form hetero-oligomers

The process of combining multiple homo-oligomers into one hetero-oligomeric structure was carried out using a custom Python script, recombine_2.1.py, written for Python 3.8.2. It uses PyMol (version 2.5.0) to superimpose and splice together homo-oligomer structures at the specified structural recombination point in the desired order, controlled through command line arguments. The recombined structure could then be directly outputted or optimized using Rosetta cartesian backbone minimization to fix any bad backbone geometry. Finally, if not present before, tyrosines were added manually to the surfaces to act as crystal contacts to aid crystallography.

### Protein production

All synthetic genes were ordered in pET29b+ vectors from Integrated DNA Technologies Inc. (Coralville, IA, USA) (IDT) or Genscript Inc. (Piscataway, NJ, USA), with tags added to aid in the purification and identification of protein assemblies. Plasmids for some heterooligomer designs were constructed for multicistronic expression, in which case the His6-tag was included on the final open reading frame. Plasmids were transformed into *E. coli* strain BL21(DE3)Star (Invitrogen) and overnight cultures were used to inoculate autoinduction expression media^55^ supplemented with 100 ug/mL kanamycin, which were grown with shaking at 37 °C for 18 – 24 h. Cultures were harvested by centrifugation and lysed in the presence of phenylmethylsulfonyl fluoride (PMSF). Lysates were cleared by centrifugation, and proteins were purified using immobilized metal affinity chromatography (IMAC) with Ni-NTA affinity resin (Qiagen). Samples were concentrated using 10k MWCO spin concentrators and were purified using a Superdex 200 10/300 increase column (Cytiva) in SEC buffer (25 mM Tris, 300 mM NaCl, pH 8) using an ÄKTA pure system (Cytiva). See the Supplementary Methods for more purification details, as well as biochemical and structural characterization.

### Native mass spectrometry (nMS)

Online buffer exchange coupled to native mass spectrometry (OBE-nMS) was used to analyze the oligomeric states of SEC-purified and LC-MS verified samples. Samples were prepared from SEC fractions at about 1 mg/ml prior to analysis. Using a Vanquish Duo Ultra-High-Performance LC (UHPLC) system (Thermo Scientific) coupled to a Q Exactive Ultra High Mass Range (UHMR) Hybrid Quadrupole-Orbitrap mass spectrometer (Thermo Scientific) and desalting cartridge prototypes (about 2 mm × 25, 30, or 50 mm; 80, 120, or 250 Å pore size; packed with 3 µm silica or polymer particles) provided by Thermo Fisher Scientific (Sunnyvale, CA), samples were buffer exchanged into 200 mM ammonium acetate^28,29^. Mass spectra were then deconvolved with UniDecs versions 4.1.2 and later^56^. Species assignment was restricted to close matches to expected masses, informed by on-target and off-target protein complex molecular weight predictions (considering fMet cleavage)^57^ and denaturing LC-MS data for individual subunit masses. Some peaks with masses significantly different from expected were left unassigned, and could be the result of additional adducts that remain after the online buffer exchange process. LC-MS data agreement generally disqualifies post-translational modifications or truncations but this cannot be ruled out in all cases. Presented data represents the final dataset collected, which for most samples was the only dataset.

### Negative stain electron microscopy (nsEM)

SEC purified samples were diluted to about 0.005 mg/ml using SEC buffer immediately before application for 45 s to glow discharged thick carbon film-coated 400 mesh copper grids (CF400-CU TH) (Electron Microscopy Sciences). Grids were then stained and dried immediately twice using 2% uranyl formate. Dried grids were screened on a 120 kV Talos L120C transmission electron microscope. The *E. Pluribus Unum* (EPU) (FEI Thermo Scientific, version 3.1) software was used for automated data collection. Data processing was carried out in CryoSPARC™ (Structura Biotechnology Inc, version 3).

### Small-angle X-ray scattering (SAXS)

TEVp-cleaved (and optionally AEC purified) BGL and hetBGL samples and non-cleaved RTR samples were re-purified by SEC in SAXS buffer and concentrated using thoroughly washed 10 k MWCO small spin concentrators; the flowthrough of concentration was used as blanks for buffer subtraction. Scattering measurements were performed at the SIBYLS 12.3.1 beamline at the Advanced Light Source as part of the HT-SAXS program. The X-­ray wavelength (λ) was 1.27 Å, and the sample-to-­detector distance was 1.5 m, corresponding to a scattering vector q (q = 4π sin θ/λ, where 2θ is the scattering angle) range of 0.01 – 0.3 Å^−1^. A series of exposures, in equal sub­second time slices, were taken of each well: 0.3 s exposures for 10 s resulting in 32 frames per sample. For each sample, data was collected for two different concentrations to test for concentration ­dependent effects; low concentration samples were about 2.5 mg/mL and high concentration samples were about 5  mg/mL. Data was processed using the SAXS FrameSlice online server (https://bl1231.als.lbl.gov/ran). FoXS^58^ (from IMP 2.12) was used to compare design models to experimental scattering profiles and calculate the quality of fit (χ) values. The c1 and c2 parameters were fixed to 1.05 and 2.0, respectively, to avoid issues of overfitting. Additional analysis was performed using ScÅtter IV^59^.

### X-ray crystallographic analyses

Crystals of BGL06, BGL14_styr (a variant of BGL14 with a single R- > Y surface mutation to promote crystallization), BGL15, BGL18, and hetBGL03-15-18 were grown using protein purified as described above and TEVp cleaved and optionally AEC purified. Protein samples dispensed in 1 uL drops at purification concentrations were mixed with an equal volume of a crystallization solution and set in hanging drops (refer to Supplementary Table 1 for conditions). Vapor phase equilibration of the resulting drops against a 1 mL reservoir of the same crystallization solution, resulting in the growth of crystals. The crystals were flash-cooled in liquid nitrogen after transfer into a cryoprotective solution (refer to Supplementary Table 1 for conditions). Diffraction data were collected on a Pilatus area detector at the Advanced Light Source (ALS) synchrotron facility at beamline 5.0.2 for BGL06, BGL14_styr, BGL15, and BGL18. Diffraction data were collected on a Rigaku HyPix-6000HE hybrid photon counting detector at the Fred Hutchinson Cancer Center (Fred Hutch) for hetBGL03-15-18. The resulting data sets (Supplementary Table 1) extend to 2.1 Å, 3.0 Å, 3.3 Å, 3.0 Å, and 2.1 Å resolution for BGL06, BGL14_styr, BGL15, BGL18, and hetBGL03-15-18, respectively. Most data had complete trimers within the asymmetric unit (three copies of a protein subunit), with exceptions to BGL18 and BGL15, which had two and four trimers in the asymmetric unit, respectively. Data was processed using the program HKL2000^60^ or Aimless^61^.

The placement of subunits was determined using the molecular replacement algorithm in program PHENIX^62^, using the original design models (which were modified prior to those computations by removing all newly incorporated side chains throughout each subunit interface). Individual side chains were then manually rebuilt into unbiased difference density, using the program COOT^63^, followed by refinement using the program PHENIX^62^. The density for all modeled side chains and corresponding backbone was unambiguous and allowed for straightforward modeling and subsequent refinement. The final values for Rwork/Rfree are notated in Supplementary Table 1.

For two crystallographic structures (BGL06 and hetBGL03-15-18), while the diffraction patterns were quite strong and visible, reflections extended well beyond 2.1 Å resolution, the spot profiles were streaky and poorly resolved beyond that point. Attempts to extend resolution beyond 2.1 Å led to poor behavior in refinement (specifically, deterioration of Rwork/Rfree values). Therefore, we conservatively maintained a 2.1 Å resolution cut-off for those refinements.

RMSD calculations between design model and crystal structure was performed using TMalign or MMalign^64^ with default settings, expect for the comparison between hetBGL03-15-18 and BGL0 which included the -cp flag to account for the circular permutations due to inclusion of an HR-N interface.

### Statistics and reproducibility

All BGL homotrimer constructs, hetBGL constructs, and RTR homo-oligomeric constructs were purified on at least two separate occasions. Those which passed screening were purified at least one additional time. All hetRTR constructs were purified at least once and A2B2-hetRTR05-11, A_5_2B_7_2-hetRTR05-11_noCys_, A3B3-hetRTR05-11_noCys_, and A2B2C2-hetRTR11_noCys_-05-16 were purified twice. All data presented originates from the final purification, though results obtained from earlier purifications were comparable where applicable.

### Reporting summary

Further information on research design is available in the Nature Portfolio Reporting Summary linked to this article.

## Supplementary information

Supplementary Information

Description of Additional Supplementary Files

Supplementary Data 1

Supplementary Data 2

Supplementary Data 3

Supplementary Data 4

Supplementary Data 5

Supplementary Data 6

Supplementary Data 7

Supplementary Data 8

Supplementary Data 9

Supplementary Data 10

Supplementary Data 11

Supplementary Data 12

Supplementary Data 13

Reporting Summary

Transparent Peer Review file

## Source data

Source Data

## Data Availability

The X-ray crystallography coordinates and structure files data generated in this study have been deposited in the Protein Data Bank under the following accession codes: 8E0L (BGL06), 8E0M (BGL15), 8E0N (BGL18), 8E0O (hetBGL03-15-18), and 8E12 (BGL14_styr). Coordinates of BGL0 and structure files used in this study are available at the Protein Data Bank with accession code 6XR2. The design models tested in this study have been deposited to Zenodo^65^ with accession code 7851258. The SAXS, SEC-MALS, SEC, and nMS data generated in this study have been deposited to Zenodo^66^ with accession code 7853172. The raw OBE MS data generated in this study have been deposited to MassIVE^67^ under accession code MSV000092722 (Raw data files correspond to the file names given in the reports in Supplementary Data 13). Source data are provided with this paper.

## Code availability

The code used to carry out protein design in this study has been deposited to Zenodo^68^ with accession code 13411451 and is also available at https://github.com/rdkibler/pseudosymmetric_hetero-oligomers.
