# Supplementary material for: Design of pseudosymmetric protein hetero-oligomers
Source: Nat Commun. 2024 Dec 18;15:10684. doi: 10.1038/s41467-024-54913-8 (PMC11655659; doi:10.1038/s41467-024-54913-8)
Supplement: Supplementary file 1 [file 41467_2024_54913_MOESM15_ESM.pdf]

BGL0

Exp:trimer
Date Analyzed: Mon, 13 Jun 2022, 12:39 US Mountain Standard Time
File Name: 20220512_RKOG_6K_IST050_HCD050

Quality Check:
Average Peaks Score: 0.18

Expected Oligomers:
Passed:
Mixed:
Failed:

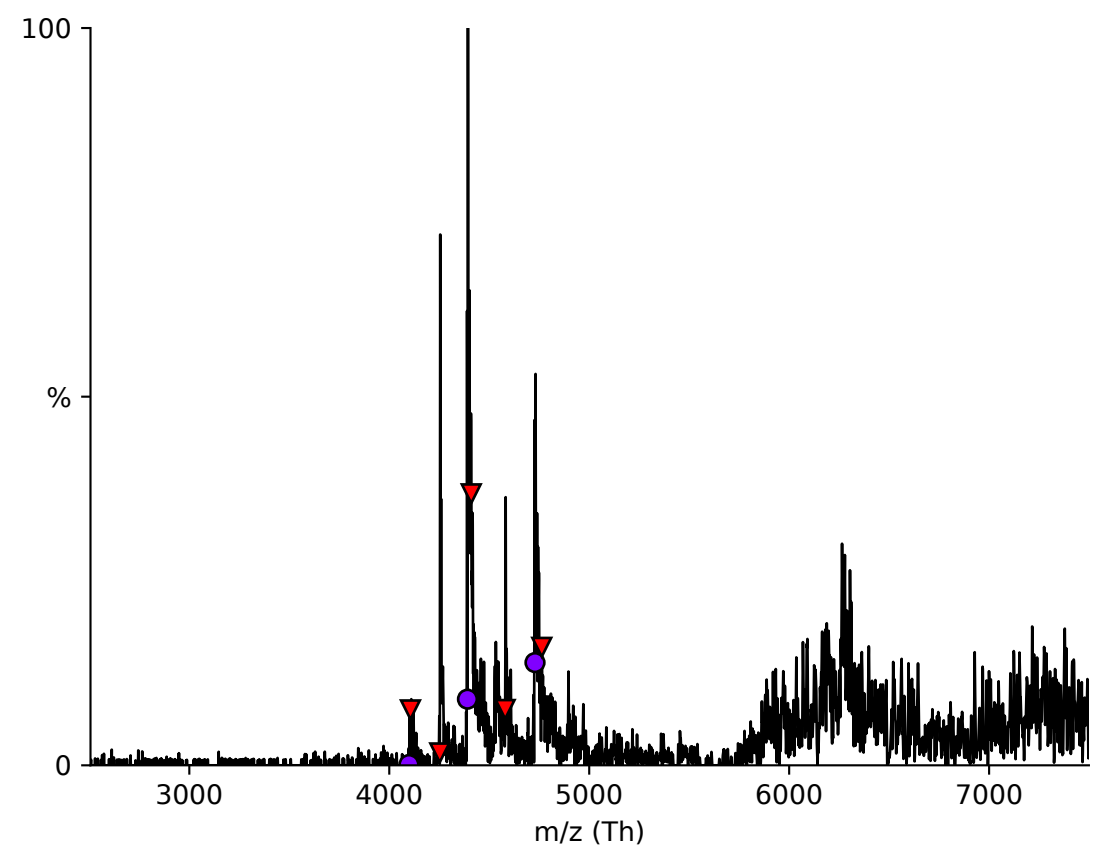

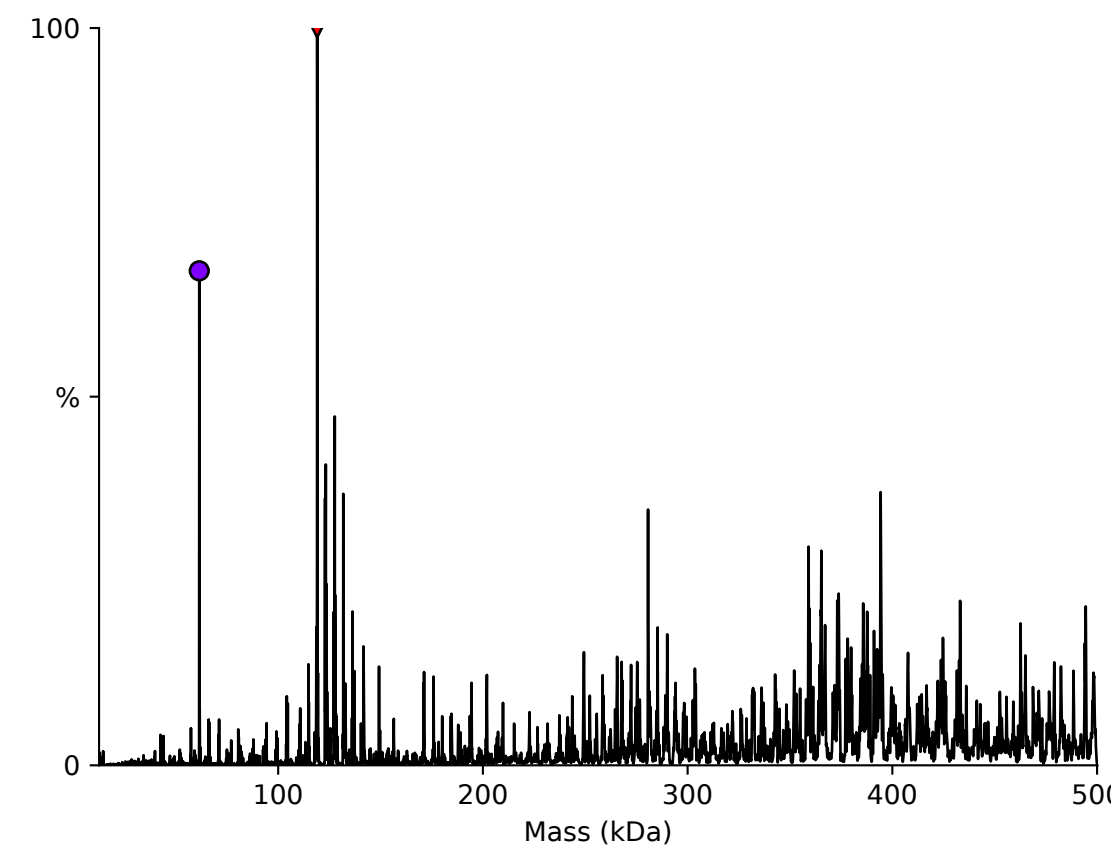

Symbol	Mass (Da)	Intensity[1]	Expected	Difference (Da)	Name/Species/Oligomer
○	61462	67	63522	−2060	3
▽	119043	100	127044	−8001	6

Comments: trimer present, but so is a hexamer. Also, masses are off

[1]Intensity values should not be considered quantitative

BGL01

Exp: homotrimer monomer mass:19275
Date Analyzed: Wed, 03 Aug 2022, 11:28 Eastern Daylight Time
File Name: 20220421_RK371_6K_IST005_HCD005_selection

Quality Check:
Average Peaks Score: 0.93

Expected Oligomers:
Passed:
Mixed:
Failed:

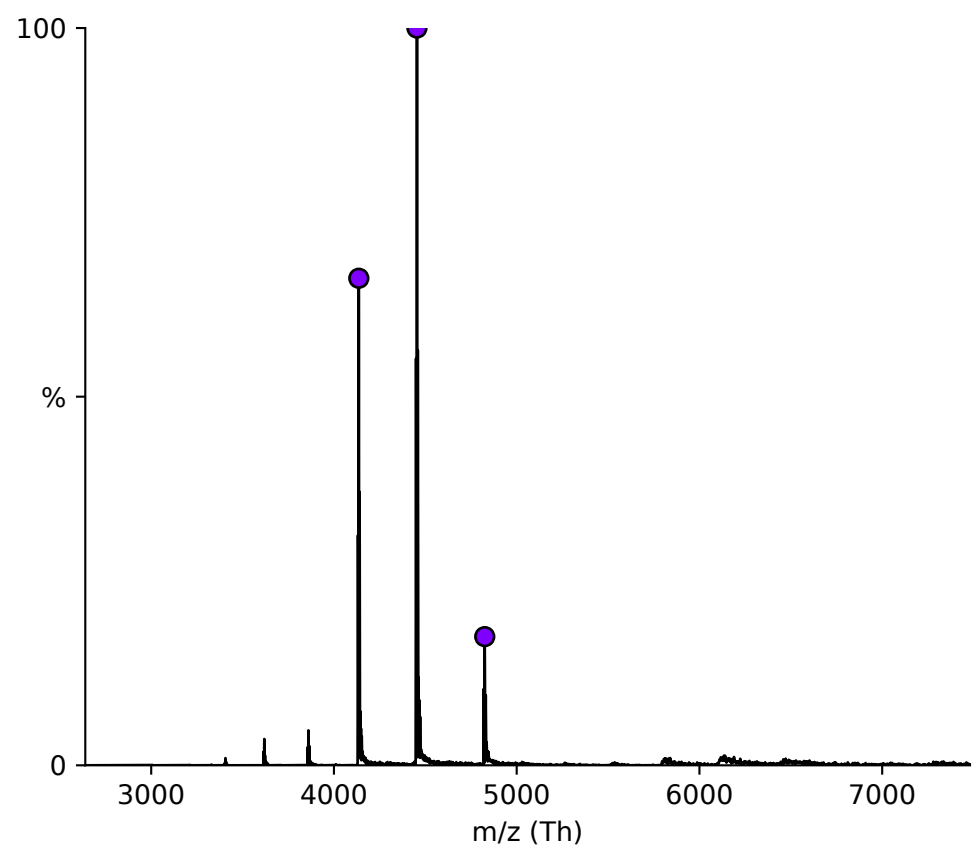

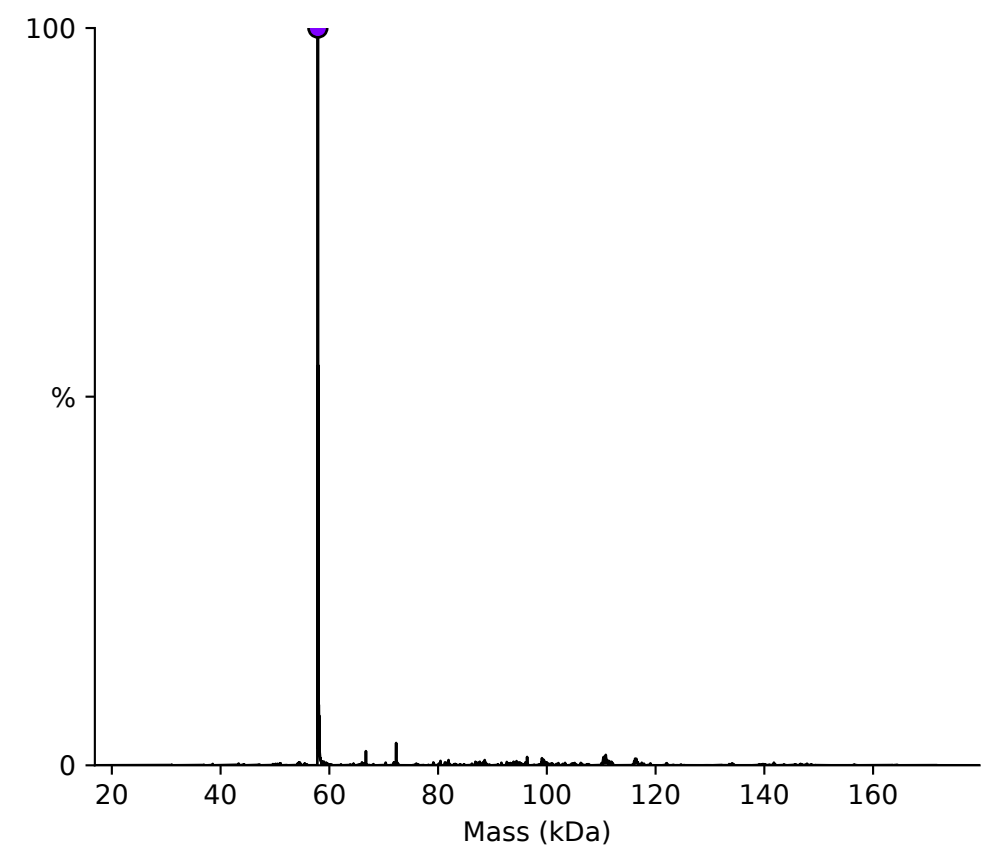

Symbol	Mass (Da)	Intensity[1]	Expected	Difference (Da)	Name/Species/Oligomer
○	57890	100	57825	65	3

[1]Intensity values should not be considered quantitative

BGL02

exp: homotrimer monomer mass:19262
Date Analyzed: Wed, 03 Aug 2022, 11:31 Eastern Daylight Time
File Name: 20220421_RK372_6K_IST005_HCD005_selection

Quality Check:
Average Peaks Score: 0.9

Expected Oligomers:
Passed:
Mixed:
Failed:

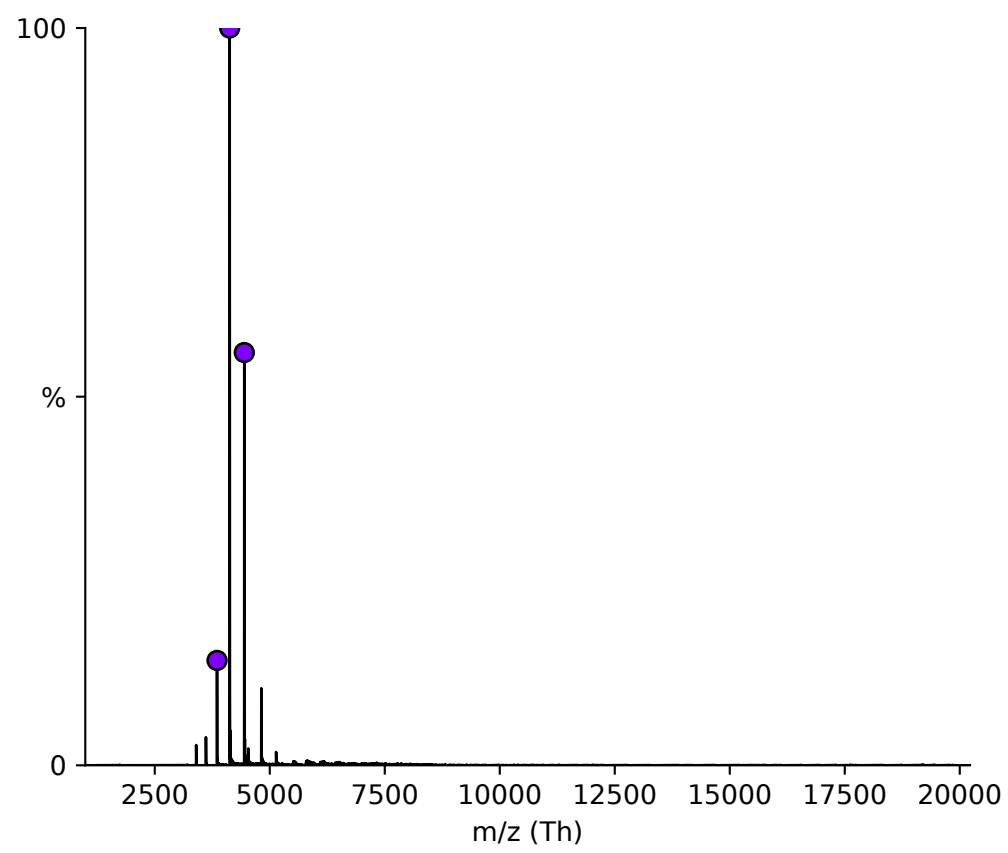

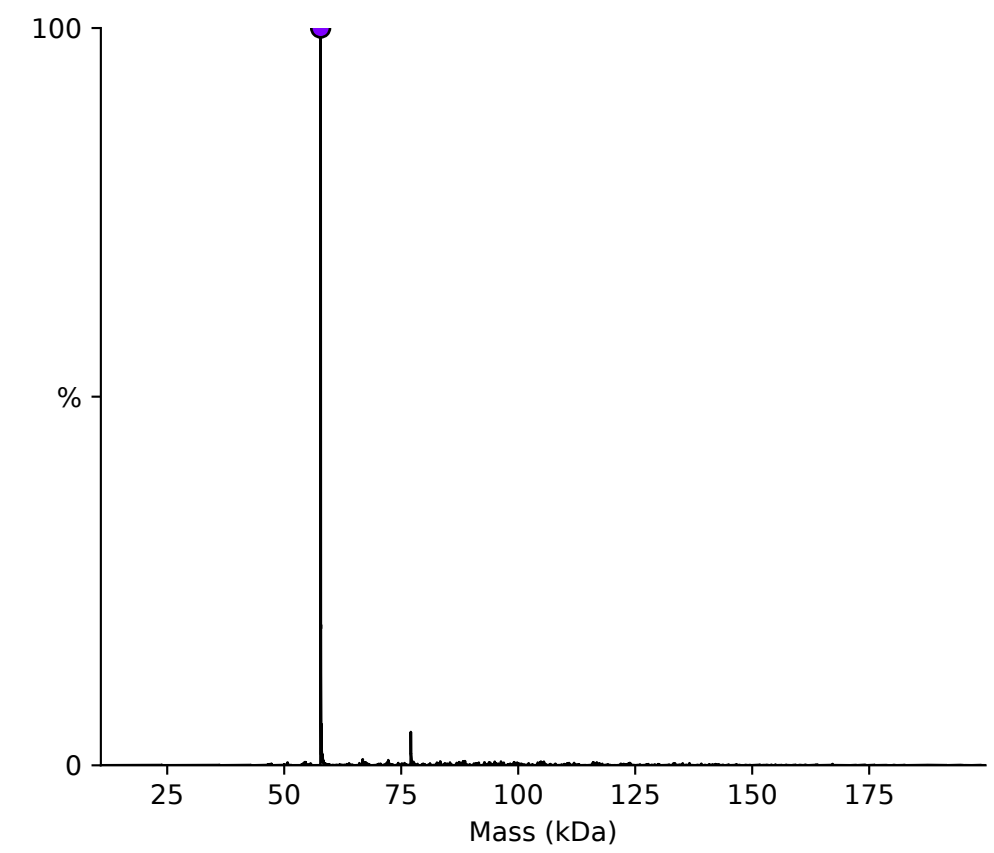

Symbol	Mass (Da)	Intensity[1]	Expected	Difference (Da)	Name/Species/Oligomer
○	57783	100	57786	−3	3

[1] Intensity values should not be considered quantitative

BGL03

homotrimer monomer mass:18726
Date Analyzed: Wed, 03 Aug 2022, 11:33 Eastern Daylight Time
File Name: 20220421_RK373_6K_IST005_HCD005_selection

Quality Check:
Average Peaks Score: 0.89

Expected Oligomers:
Passed:
Mixed:
Failed:

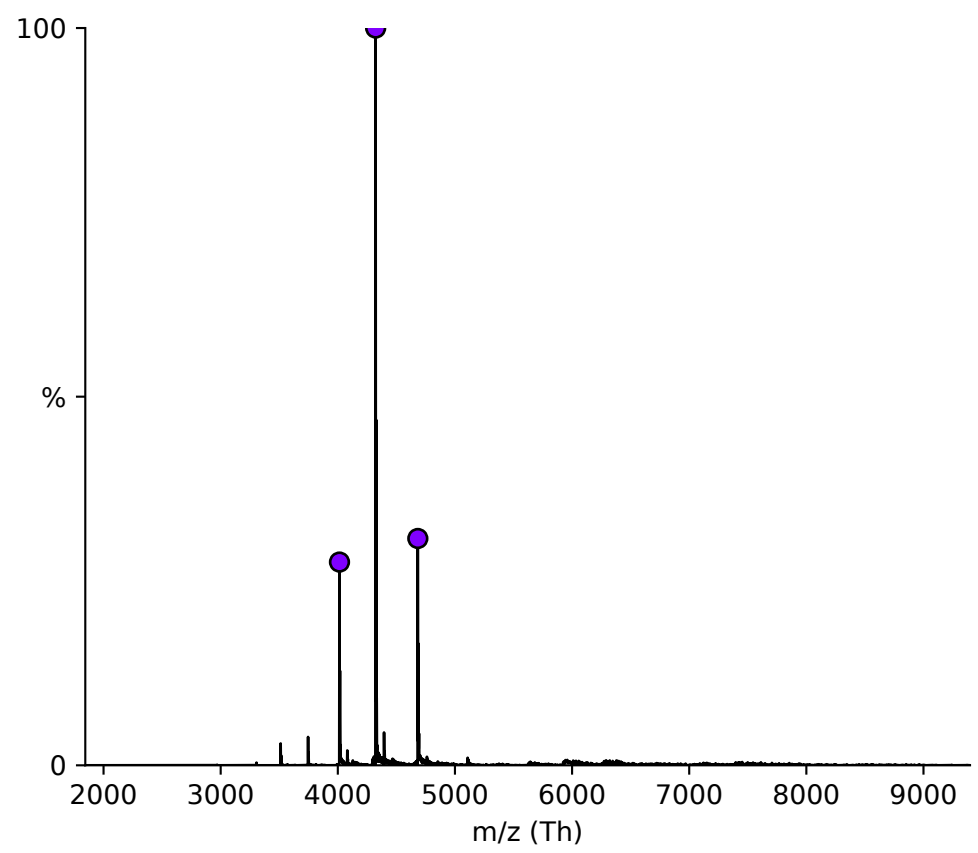

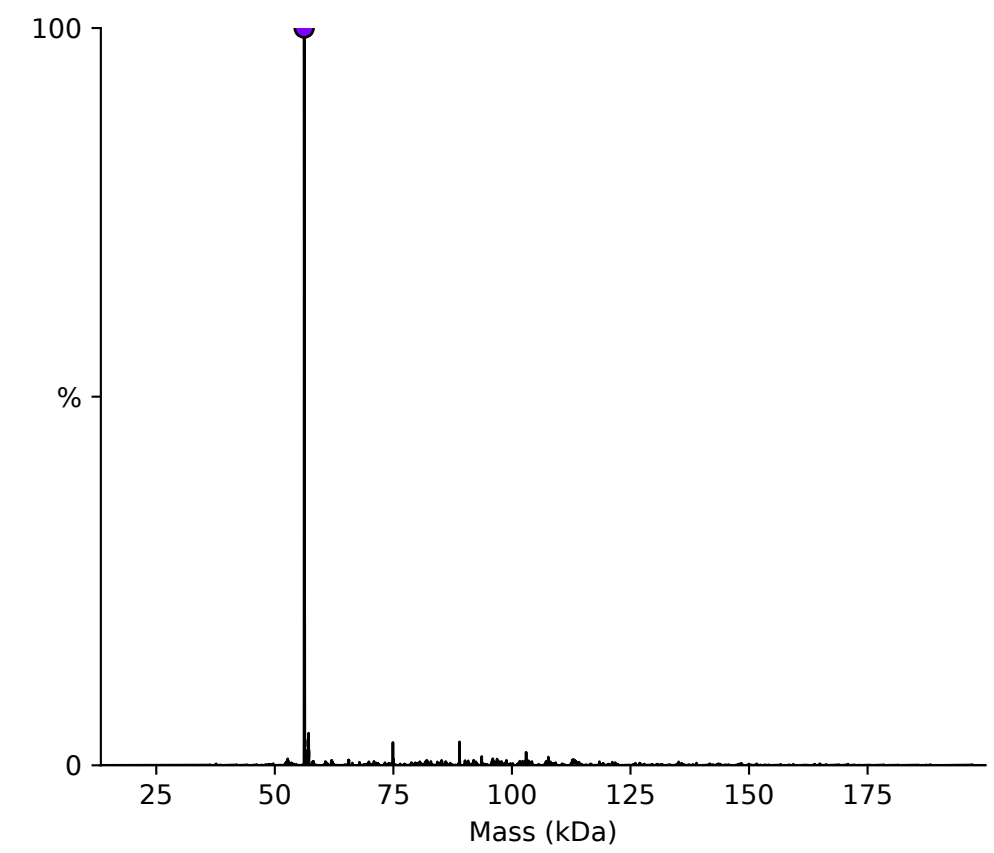

Symbol	Mass (Da)	Intensity[1]	Expected	Difference (Da)	Name/Species/Oligomer
◯	56185	100	56178	7	3

[1] Intensity values should not be considered quantitative

BGL05

RK375 homotrimer monomer mass: 18140
Date Analyzed: Wed, 03 Aug 2022, 11:39 Eastern Daylight Time
File Name: 20220421_RK375_6K_IST005_HCD005_selection

Quality Check:
Average Peaks Score: 0.93

Expected Oligomers:
Passed:
Mixed:
Failed:

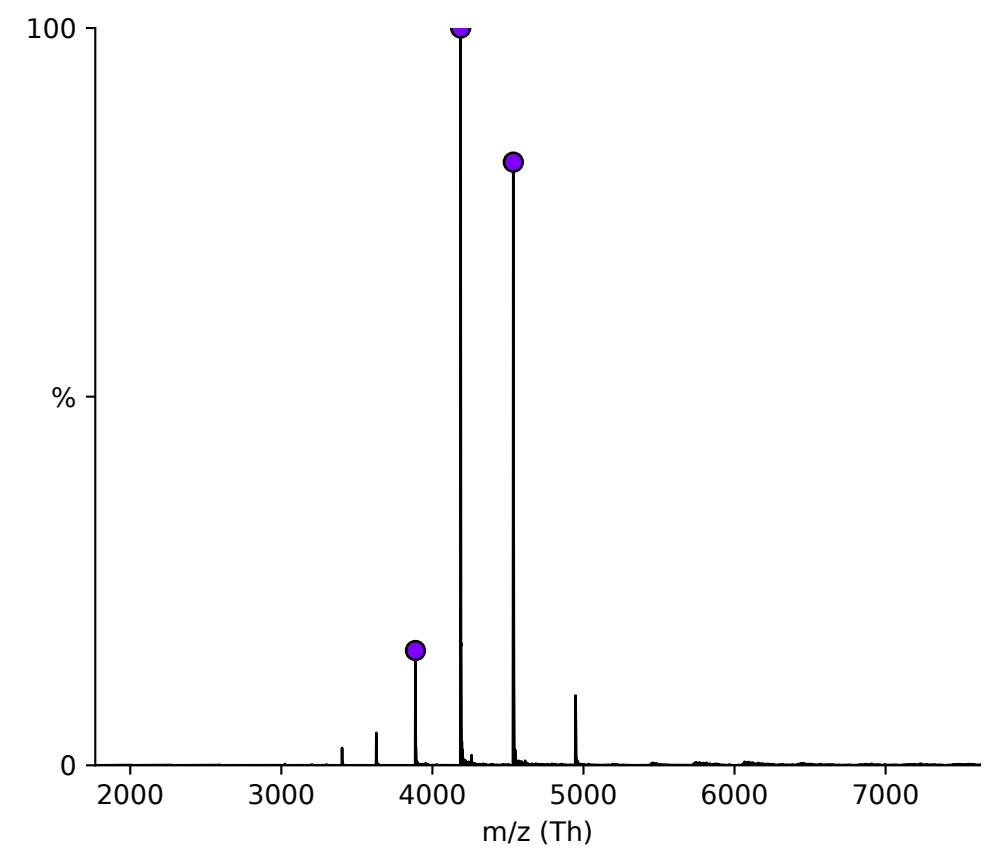

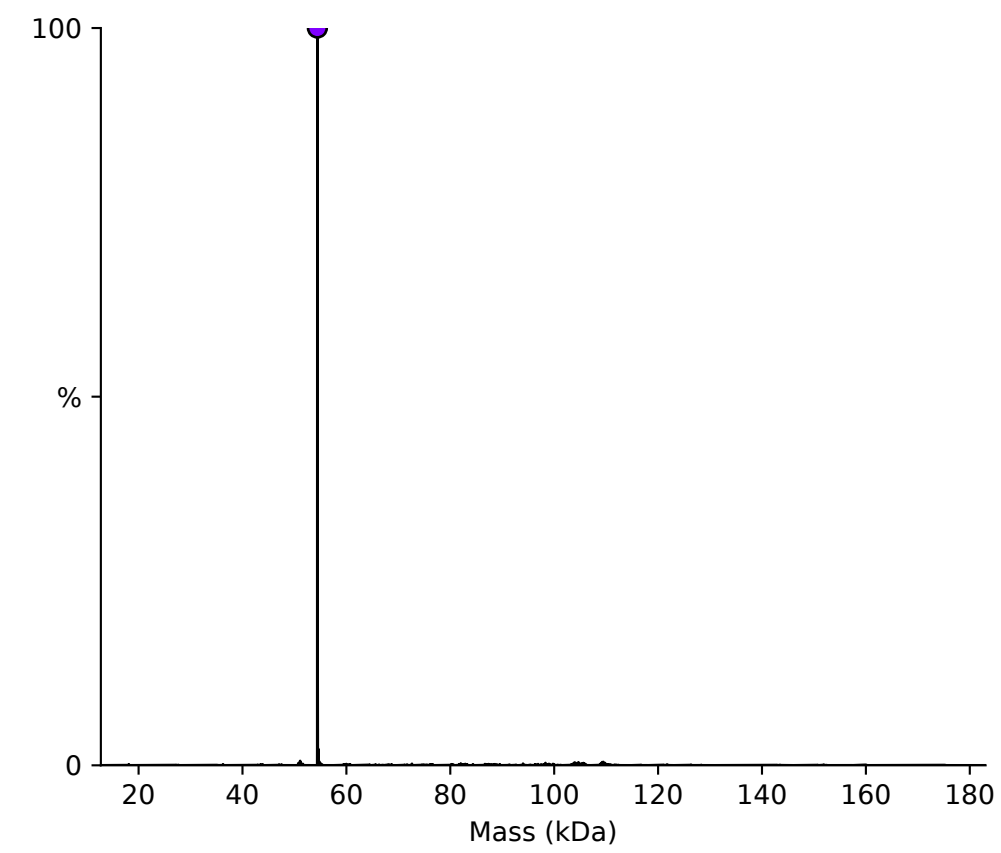

Symbol	Mass (Da)	Intensity[1]	Expected	Difference (Da)	Name/Species/Oligomer
○	54417	100	54420	−3	3[A]

[1]Intensity values should not be considered quantitative

BGL06

RK376
expected: trimer, monomer mass: 20792 Da
Date Analyzed: Tue, 18 Oct 2022, 11:29 Eastern Daylight Time
File Name: 20220512_RK376_6K_IST005_HCD005_selection

Quality Check:
Average Peaks Score: 0.51

Expected Oligomers:
Passed:
Mixed:
Failed:

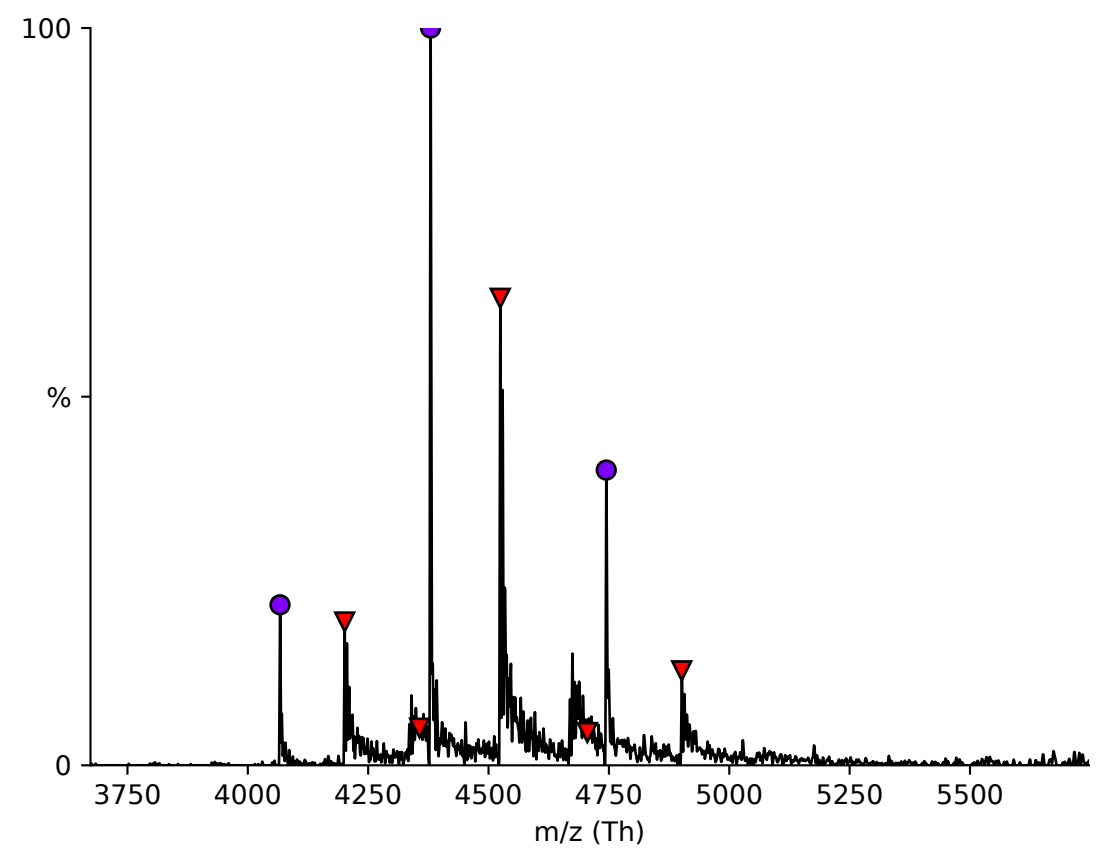

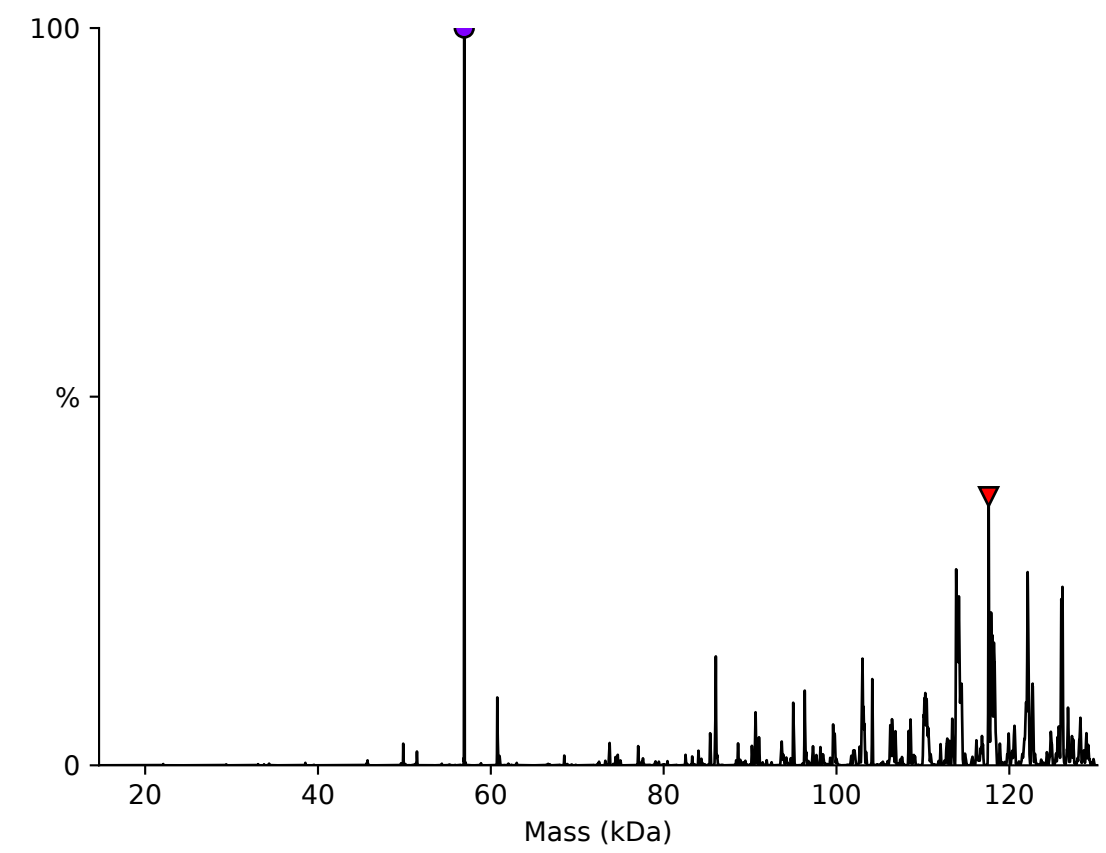

Symbol	Mass (Da)	Intensity[1]	Expected	Difference (Da)	Name/Species/Oligomer
○	56922	100	62376	−5454	3[RK376]
▽	117605	36	124752	−7147	6[RK376]

Masses are slightly off

[1]Intensity values should not be considered quantitative

BGL07

RK377 Expected: trimer
Date Analyzed: Fri, 17 Jun 2022, 11:10 US Mountain Standard Time
File Name: 20220519_RK377_C_6K_IST060_HCD005

Quality Check:
Average Peaks Score: 0.8

Expected Oligomers:
Passed:
Mixed:
Failed:

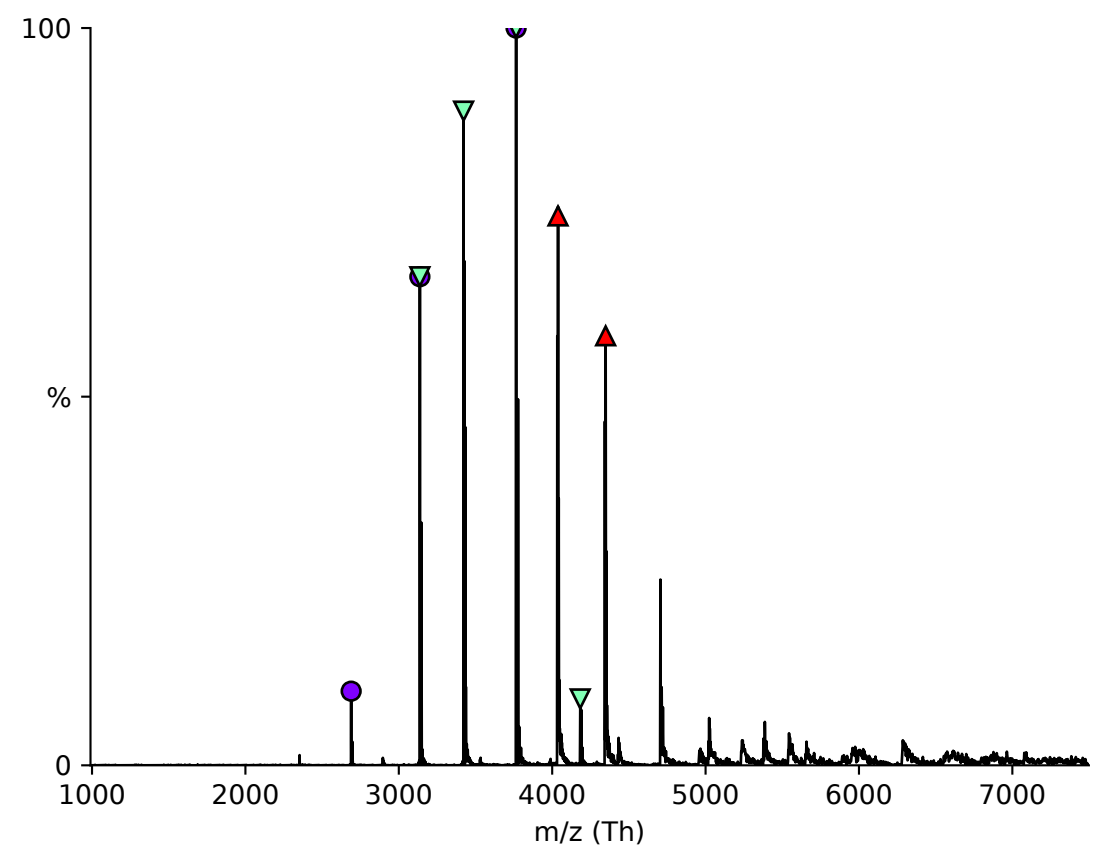

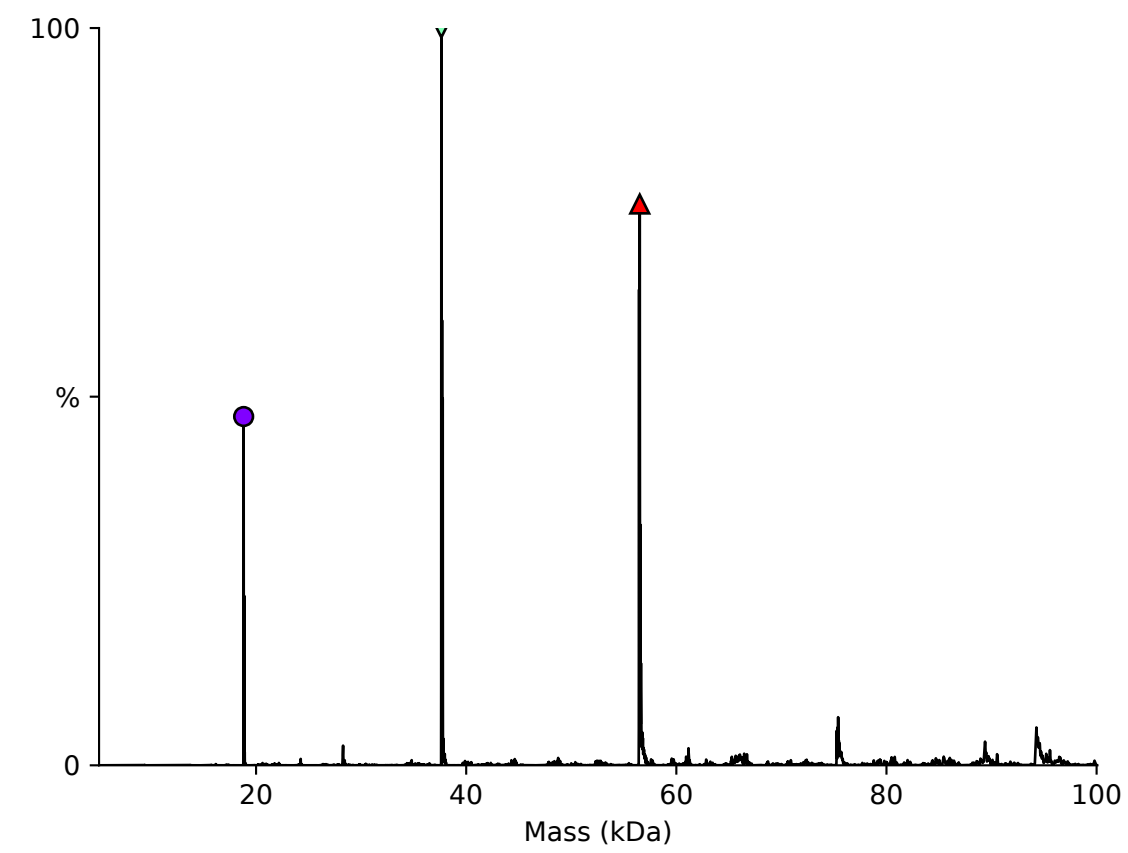

Symbol	Mass (Da)	Intensity[1]	Expected	Difference (Da)	Name/Species/Oligomer
○	18818	47	18818	0	1
▽	37636	100	37637	−1	2
△	56517	76	56455	62	3

Comments: Monomer, dimer, and trimer are present

[1] Intensity values should not be considered quantitative

BGL08

RK378 homotrimer monomer mass:18831
Date Analyzed: Wed, 03 Aug 2022, 11:42 Eastern Daylight Time
File Name: 20220421_RK378_6K_IST005_HCD005_selection

Quality Check:
Average Peaks Score: 0.93

Expected Oligomers:
Passed:
Mixed:
Failed:

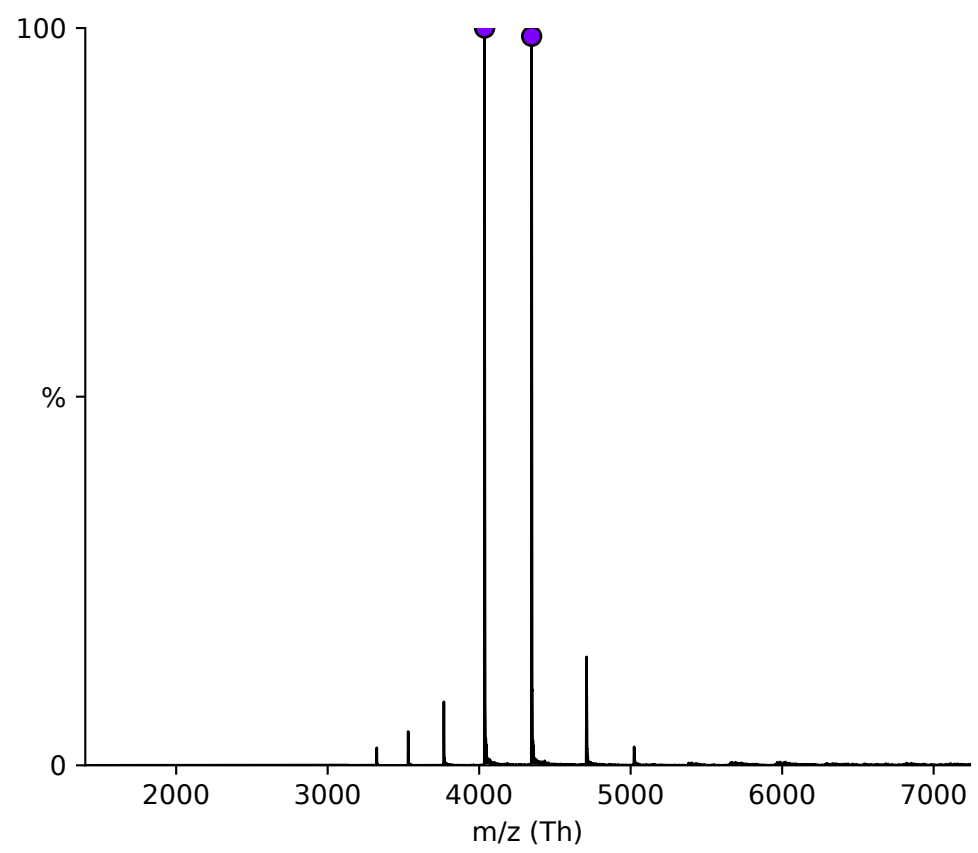

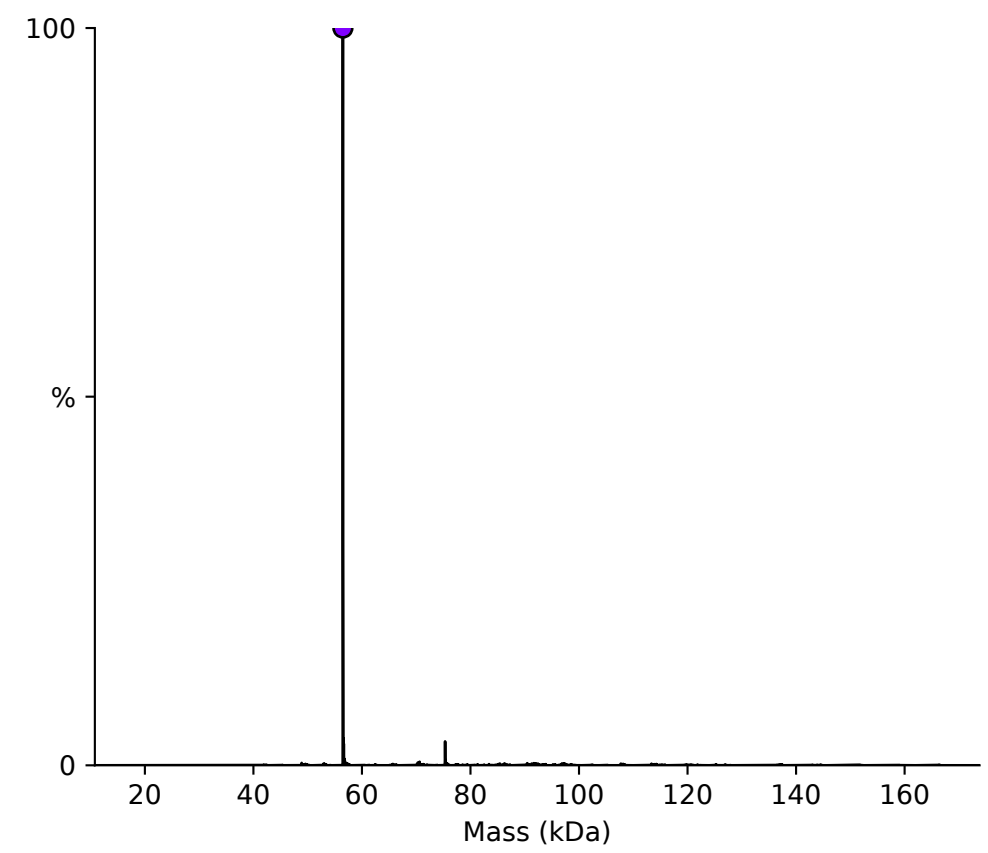

Symbol	Mass (Da)	Intensity[1]	Expected	Difference (Da)	Name/Species/Oligomer
○	56489	100	56493	−4	3[RK378]

[1] Intensity values should not be considered quantitative

BGL09

RK379 homotrimer monomer mass: 19868
Date Analyzed: Wed, 03 Aug 2022, 11:48 Eastern Daylight Time
File Name: 20220421_RK379_6K_IST005_HCD005_selection

Quality Check:
Average Peaks Score: 0.96

Expected Oligomers:
Passed:
Mixed:
Failed:

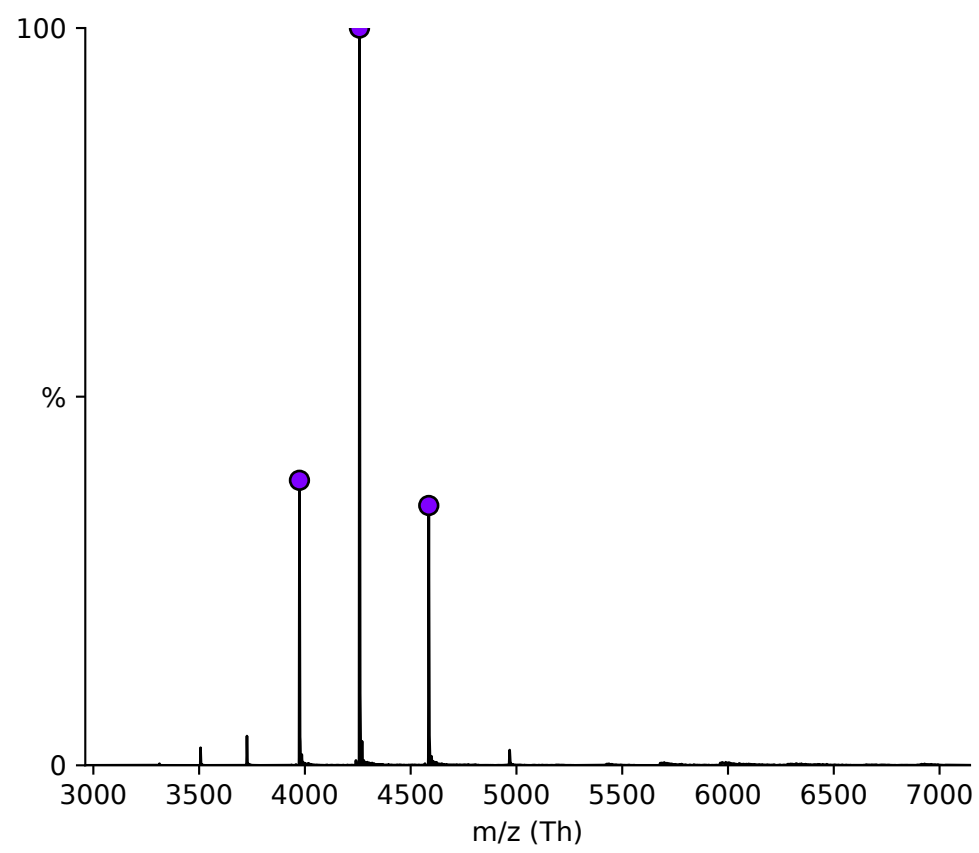

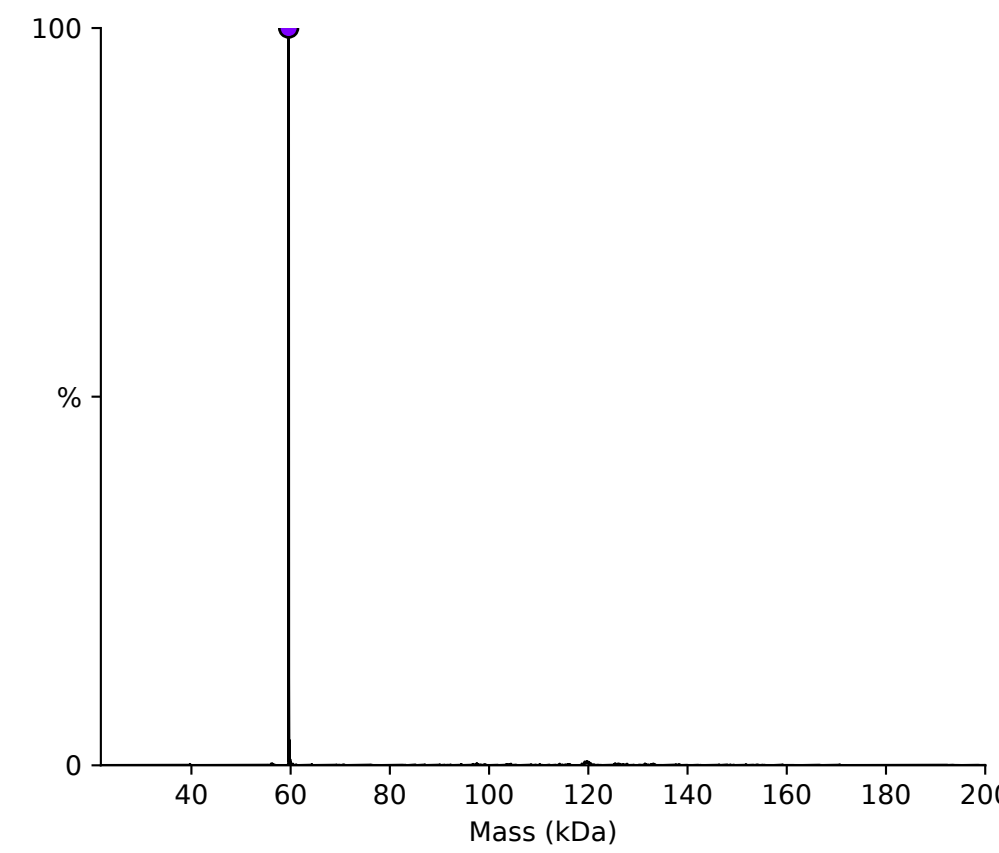

Symbol	Mass (Da)	Intensity[1]	Expected	Difference (Da)	Name/Species/Oligomer
◯	59598	100	59604	−6	3[RK379]

[1]Intensity values should not be considered quantitative

BGL11

RK381 Expected: trimer
Date Analyzed: Fri, 17 Jun 2022, 11:23 US Mountain Standard Time
File Name: 20220512_RK381_C_6K_IST060_HCD005

Quality Check:
Average Peaks Score: 0.29

Expected Oligomers:
Passed:
Mixed:
Failed:

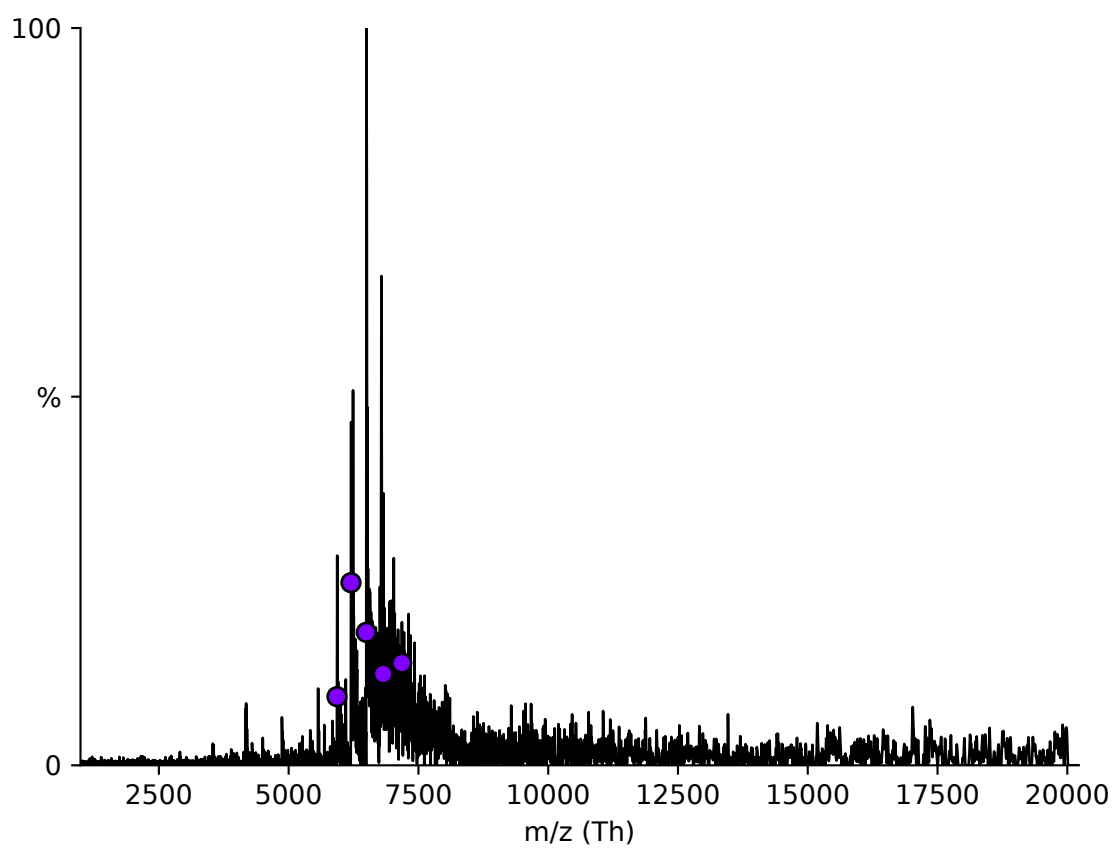

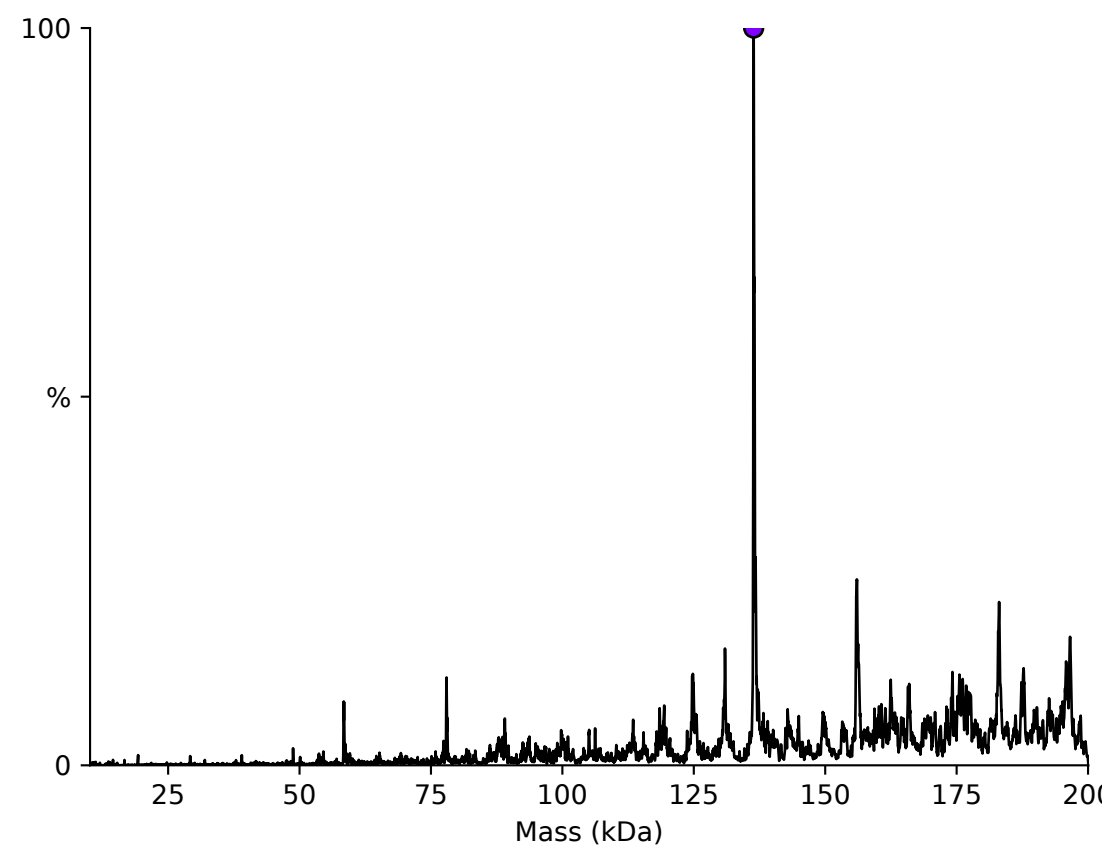

Symbol	Mass (Da)	Intensity[1]	Expected	Difference (Da)	Name/Species/Oligomer
	136390	100	130285	6105	6[RK381]

Comments: No trimer but there is a high abundance of hexamer

[1]Intensity values should not be considered quantitative

BGL12

RK382 homotrimer monomer mass: 19141
Date Analyzed: Wed, 03 Aug 2022, 11:52 Eastern Daylight Time
File Name: 20220421_RK382_6K_IST005_HCD005_selection

Quality Check:
Average Peaks Score: 0.91

Expected Oligomers:
Passed:
Mixed:
Failed:

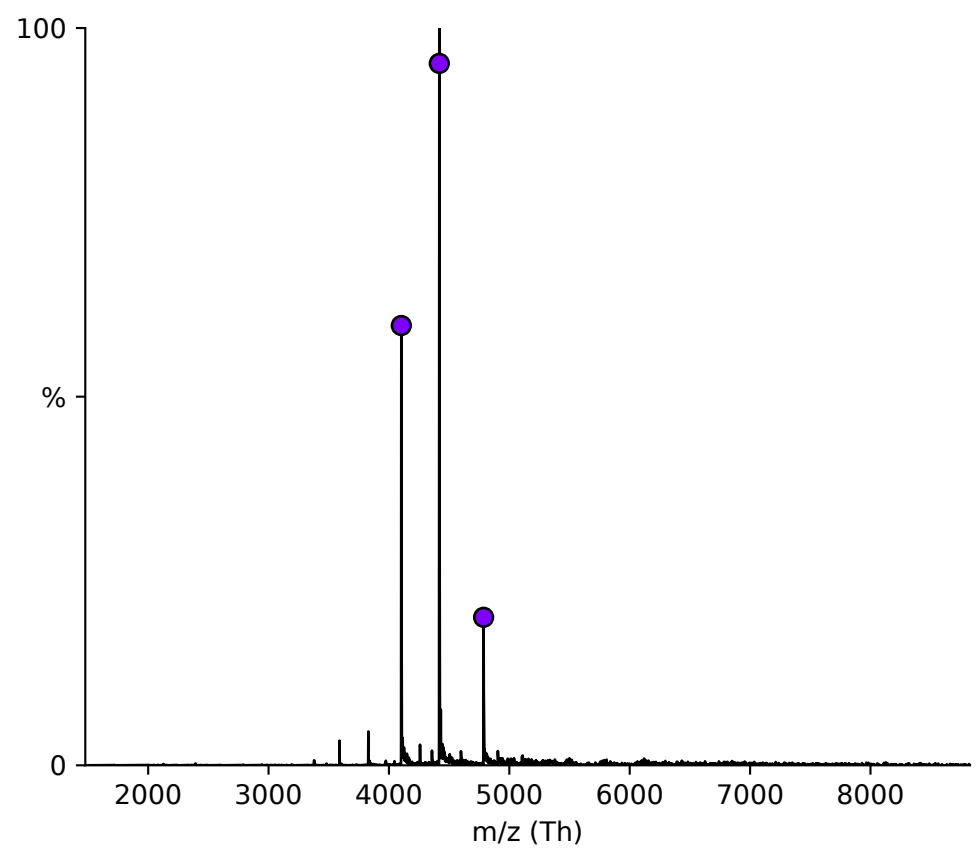

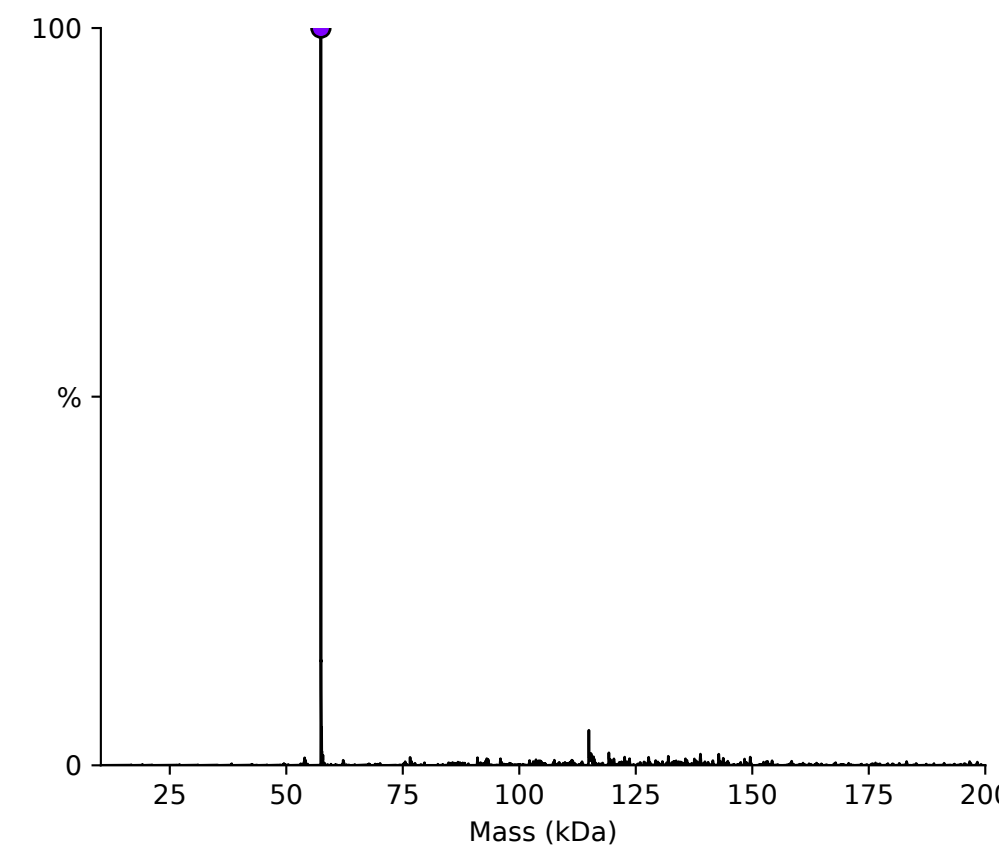

Symbol	Mass (Da)	Intensity[1]	Expected	Difference (Da)	Name/Species/Oligomer
○	57424	100	57423	1	3[RK382]

[1]Intensity values should not be considered quantitative

BGL14

RK384
expected trimer
monomer mass: 19547
Date Analyzed: Tue, 18 Oct 2022, 11:56 Eastern Daylight Time
File Name: 20220421_RK384_6K_IST005_HCD005_selection

Quality Check:
Average Peaks Score: 0.94

Expected Oligomers:
Passed:
Mixed:
Failed:

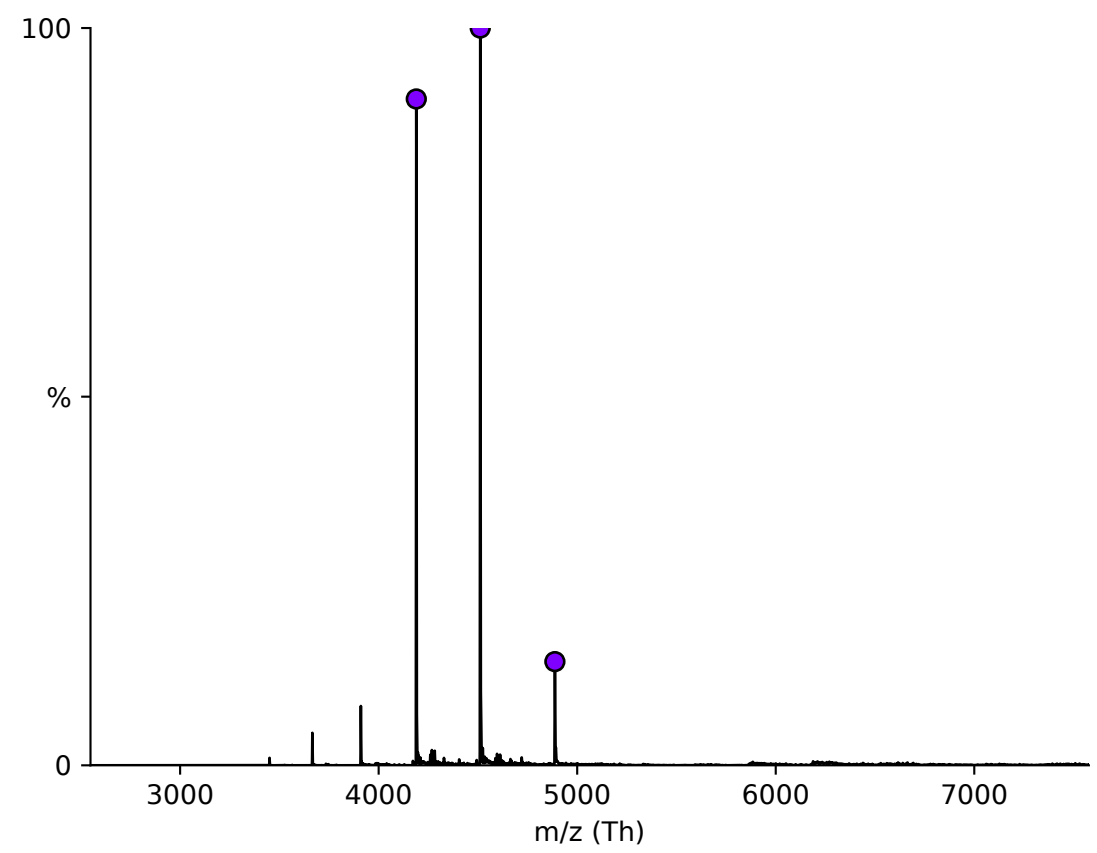

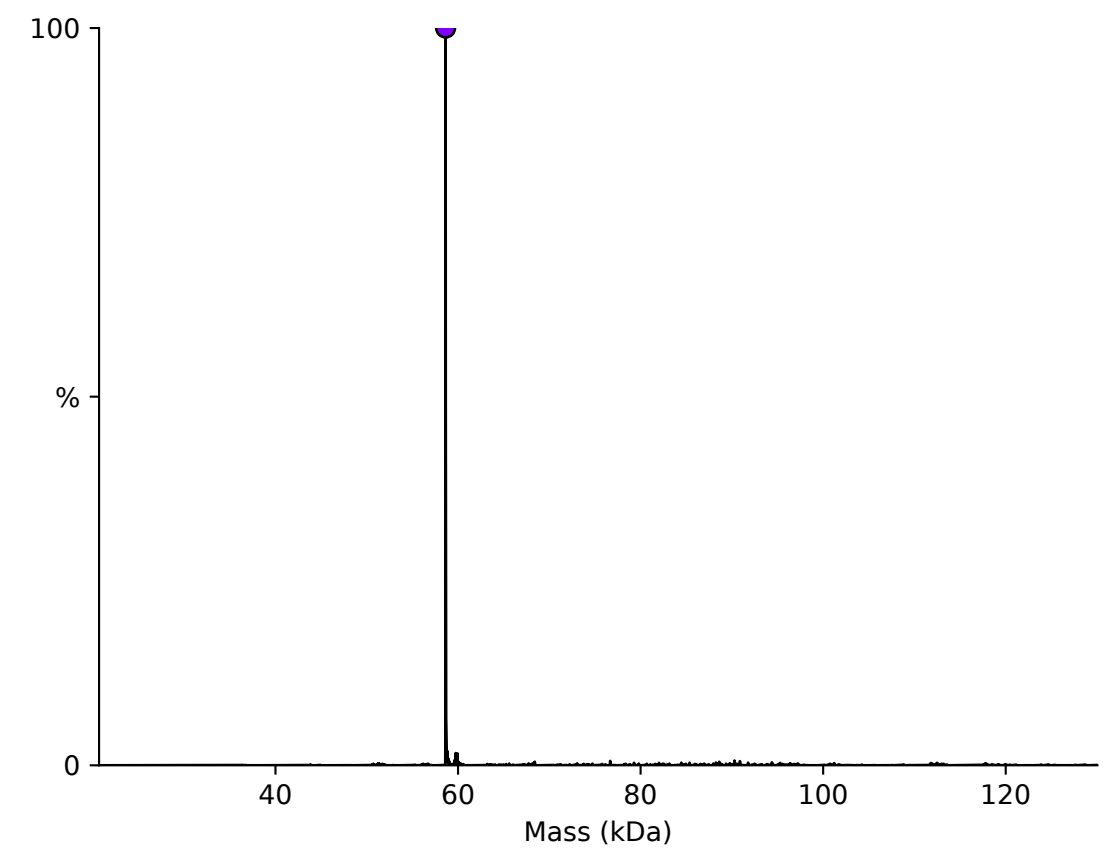

Symbol	Mass (Da)	Intensity[1]	Expected	Difference (Da)	Name/Species/Oligomer
○	58639	100	58641	−2	3[RK384]

[1]Intensity values should not be considered quantitative

BGL15

RK385 homotrimer monomer mass: 20243
Date Analyzed: Wed, 03 Aug 2022, 11:56 Eastern Daylight Time
File Name: 20220421_RK385_6K_IST060_HCD005_selection

Quality Check:
Average Peaks Score: 0.92

Expected Oligomers:
Passed:
Mixed:
Failed:

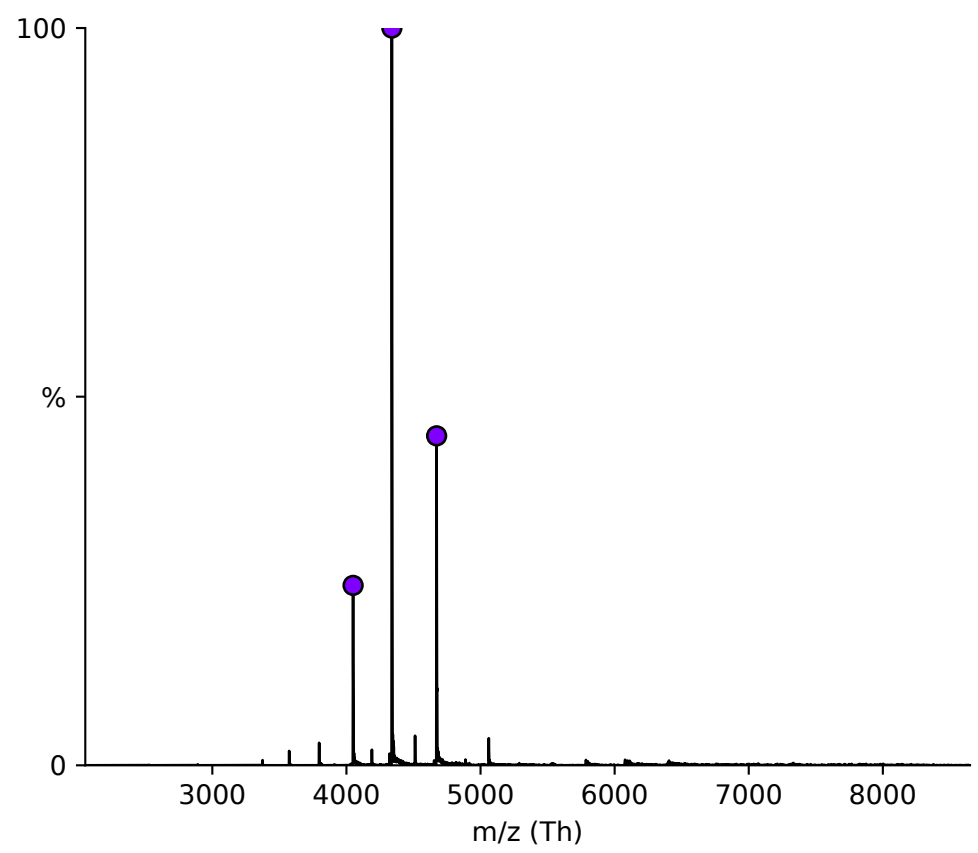

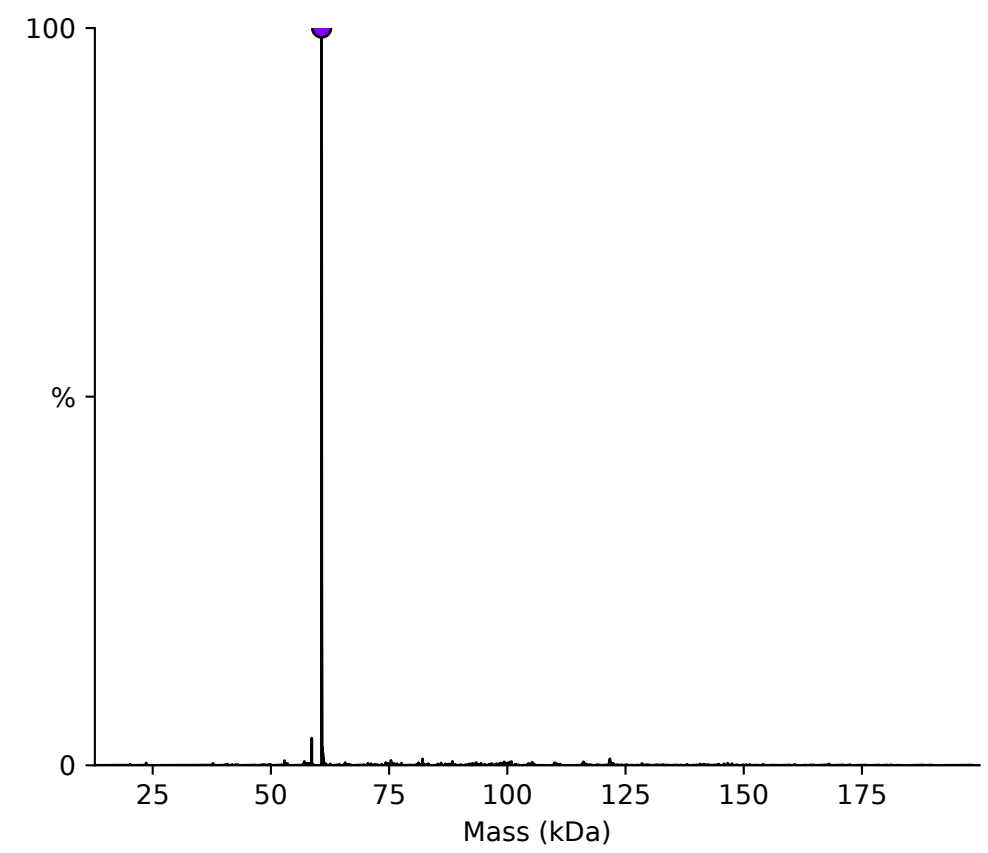

Symbol	Mass (Da)	Intensity[1]	Expected	Difference (Da)	Name/Species/Oligomer
◯	60730	100	60729	1	3[RK385]

[1] Intensity values should not be considered quantitative

BGL17

RK387 homotrimer monomer mass: 19835
Date Analyzed: Wed, 03 Aug 2022, 12:02 Eastern Daylight Time
File Name: 20220421_RK387_6K_IST060_HCD005_selection

Quality Check:
Average Peaks Score: 0.94

Expected Oligomers:
Passed:
Mixed:
Failed:

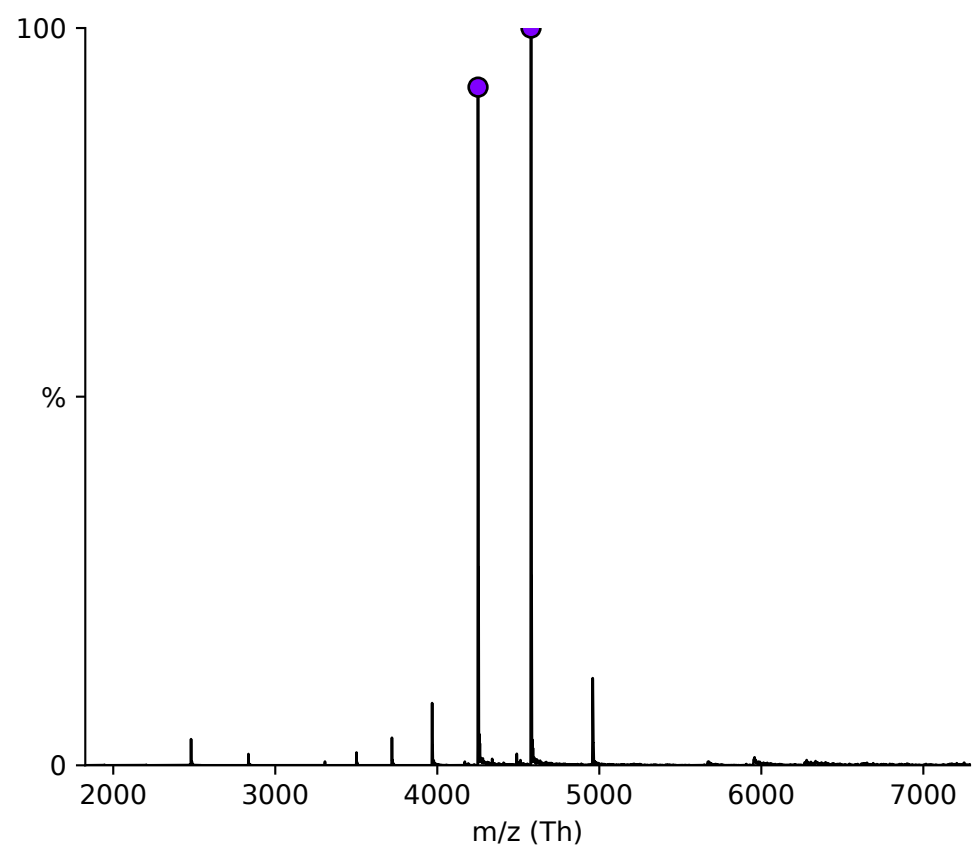

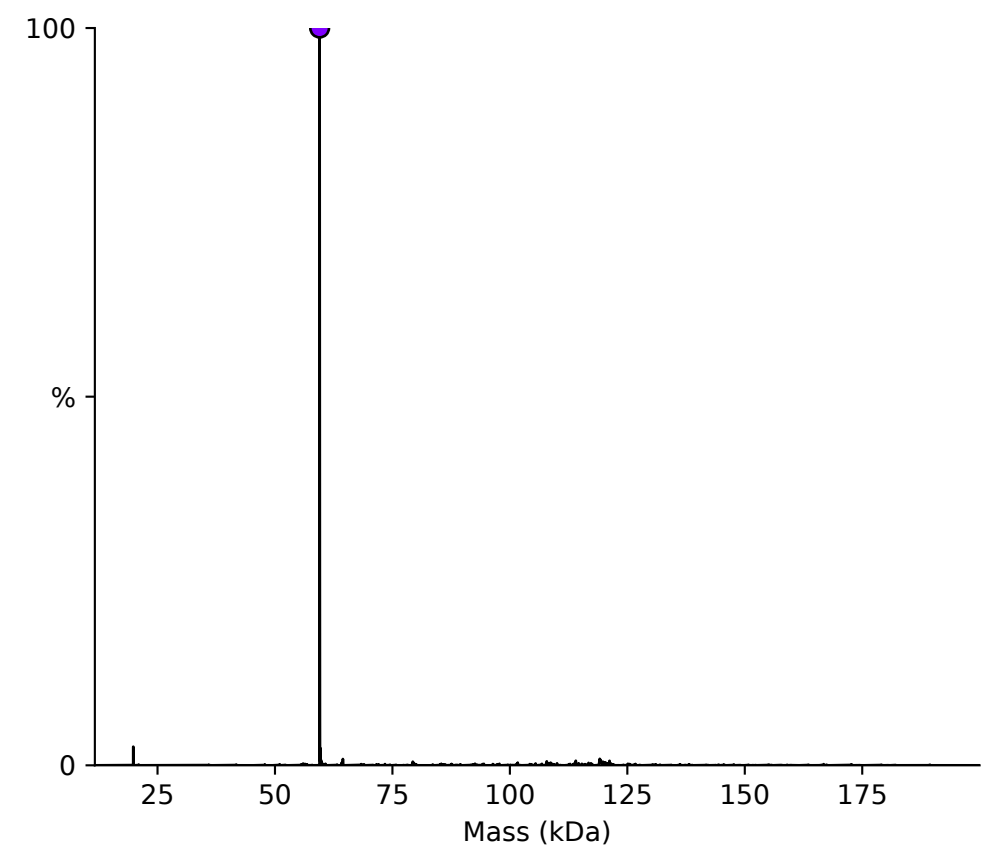

Symbol	Mass (Da)	Intensity[1]	Expected	Difference (Da)	Name/Species/Oligomer
○	59506	100	59505	1	3[RK387]

[1]Intensity values should not be considered quantitative

BGL18

RK388
expected trimer monomer mass: 21583
Date Analyzed: Tue, 18 Oct 2022, 12:03 Eastern Daylight Time
File Name: 20220512_RK388_6K_IST060_HCD005_selection

Quality Check:
Average Peaks Score: 0.86

Expected Oligomers:
Passed:
Mixed:
Failed:

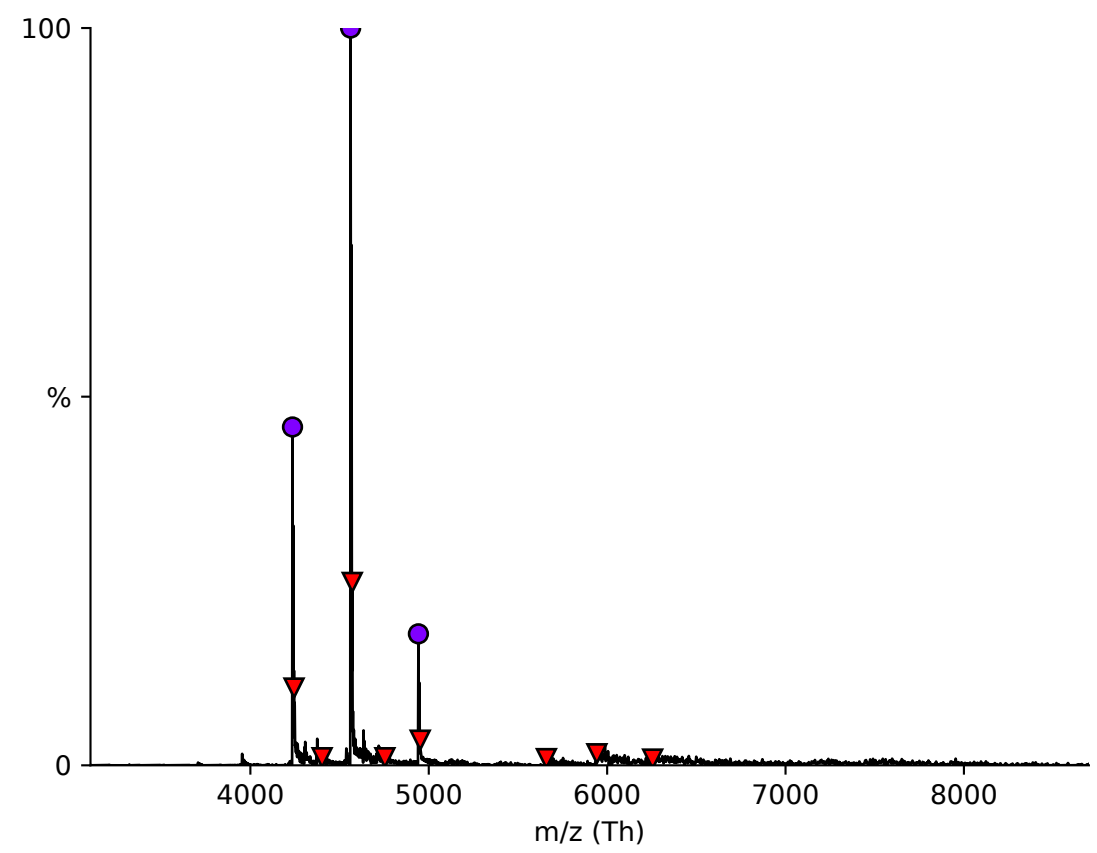

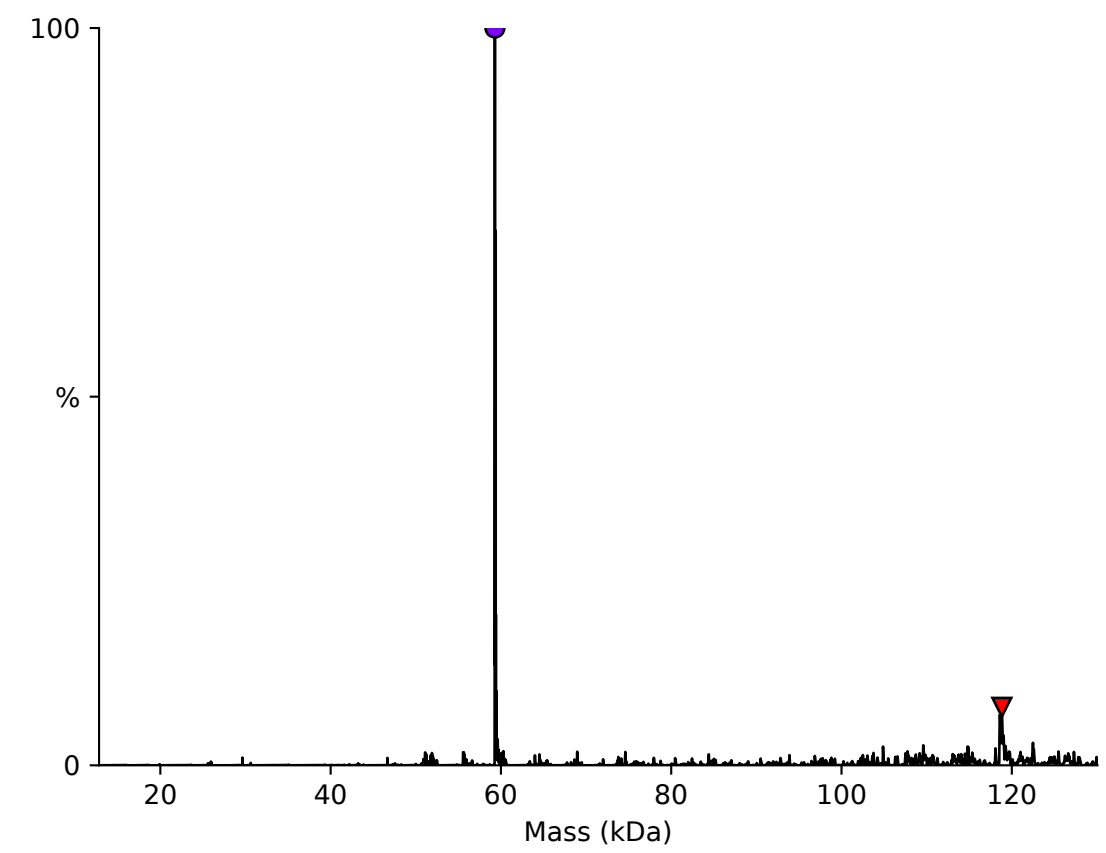

Symbol	Mass (Da)	Intensity[1]	Expected	Difference (Da)	Name/Species/Oligomer
○	59292	100	64749	−5457	3[RK388]
▽	118827	8	129498	−10671	6[RK388]

[1]Intensity values should not be considered quantitative

BGL19

RK389 expected: homotrimer, monomer mass:19913 Da
Date Analyzed: Tue, 18 Oct 2022, 12:08 Eastern Daylight Time
File Name: 20220421_RK389_6K_IST005_HCD005_selection

Quality Check:
Average Peaks Score: 0.94

Expected Oligomers:
Passed:
Mixed:
Failed:

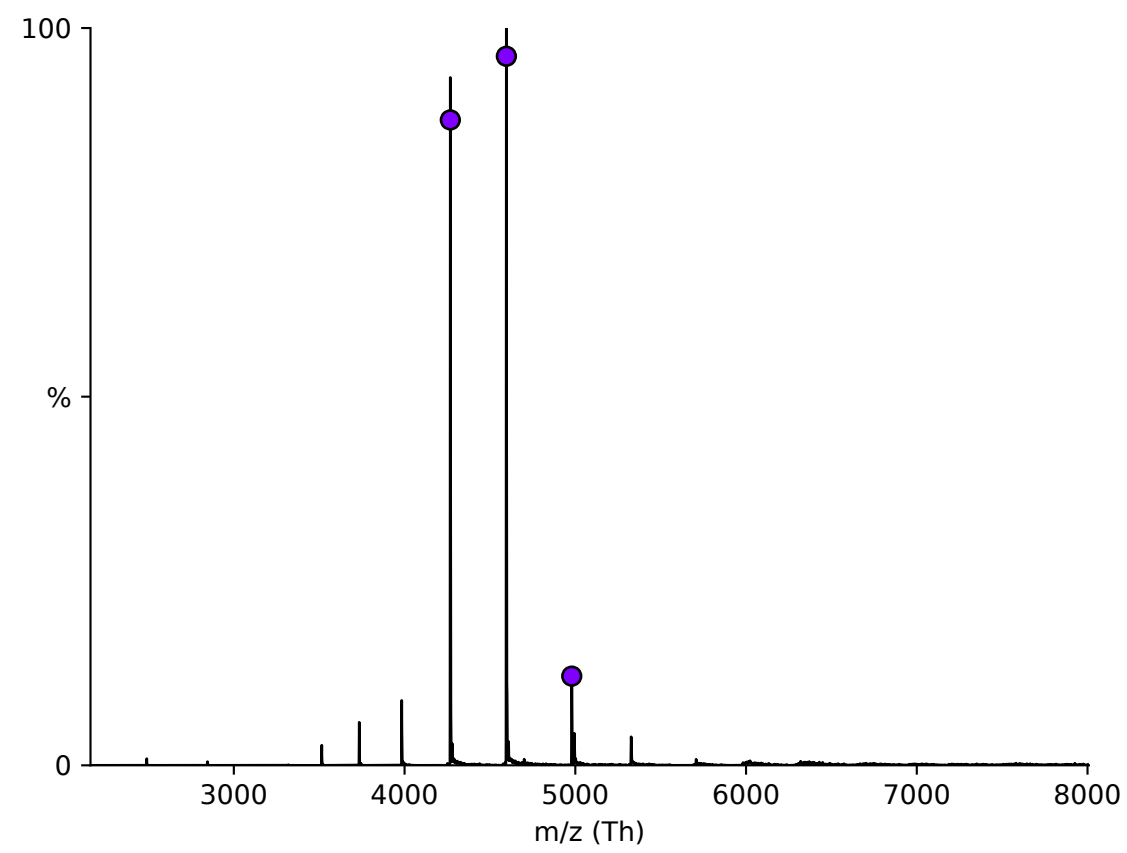

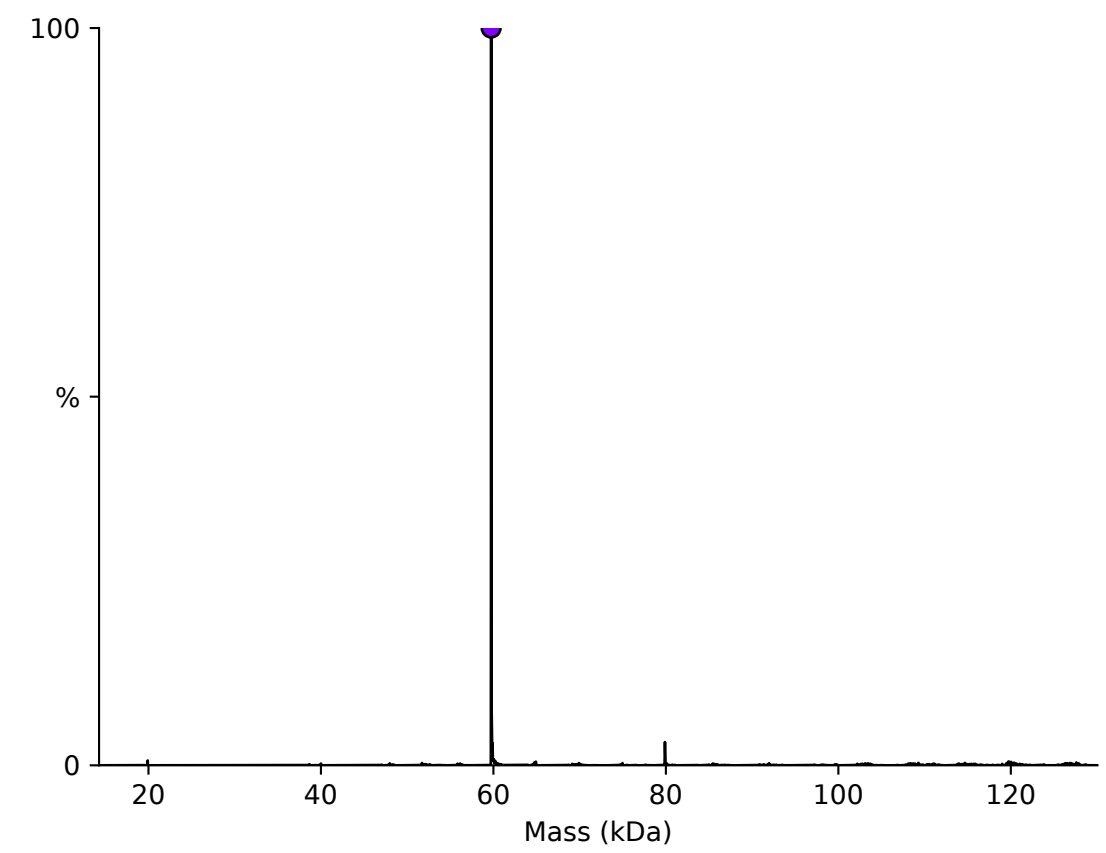

Symbol	Mass (Da)	Intensity[1]	Expected	Difference (Da)	Name/Species/Oligomer
○	59738	100	59739	−1	3[RK389]

[1]Intensity values should not be considered quantitative

BGL20

RK390e homotrimer monomer mass: 19707
Date Analyzed: Wed, 03 Aug 2022, 12:28 Eastern Daylight Time
File Name: 20220421_RK390e_6K_IST005_HCD005_selection

Quality Check:
Average Peaks Score: 0.85

Expected Oligomers:
Passed:
Mixed:
Failed:

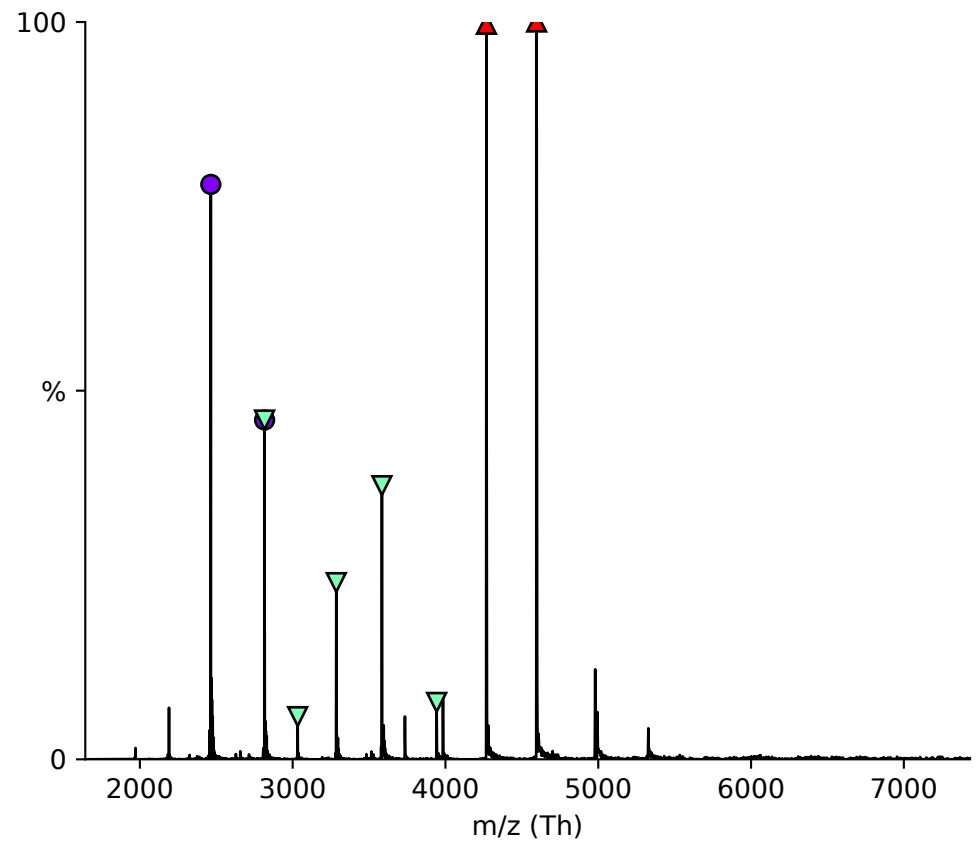

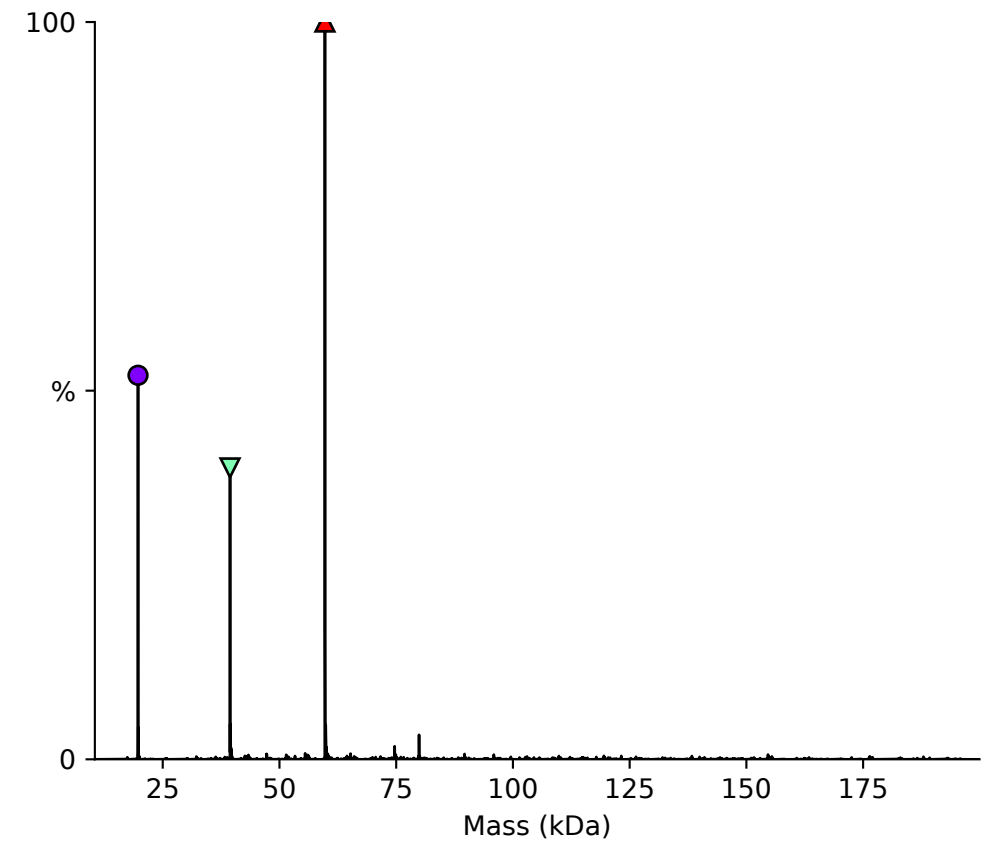

Symbol	Mass (Da)	Intensity[1]	Expected	Difference (Da)	Name/Species/Oligomer
○	19707	52	19707	0	1[RK390e]
▽	39413	40	39414	−1	2[RK390e]
△	59741	100	59121	620	3[RK390e]

[1] Intensity values should not be considered quantitative

hetBGL03-05-06

RK601
Date Analyzed: Thu, 21 Jul 2022, 16:46 US Mountain Standard Time
File Name: 20220625_RK601_6K_IST060_HCD005_selection

Quality Check:
Average Peaks Score: 0.84

Expected Oligomers:
Passed:
Mixed:
Failed:

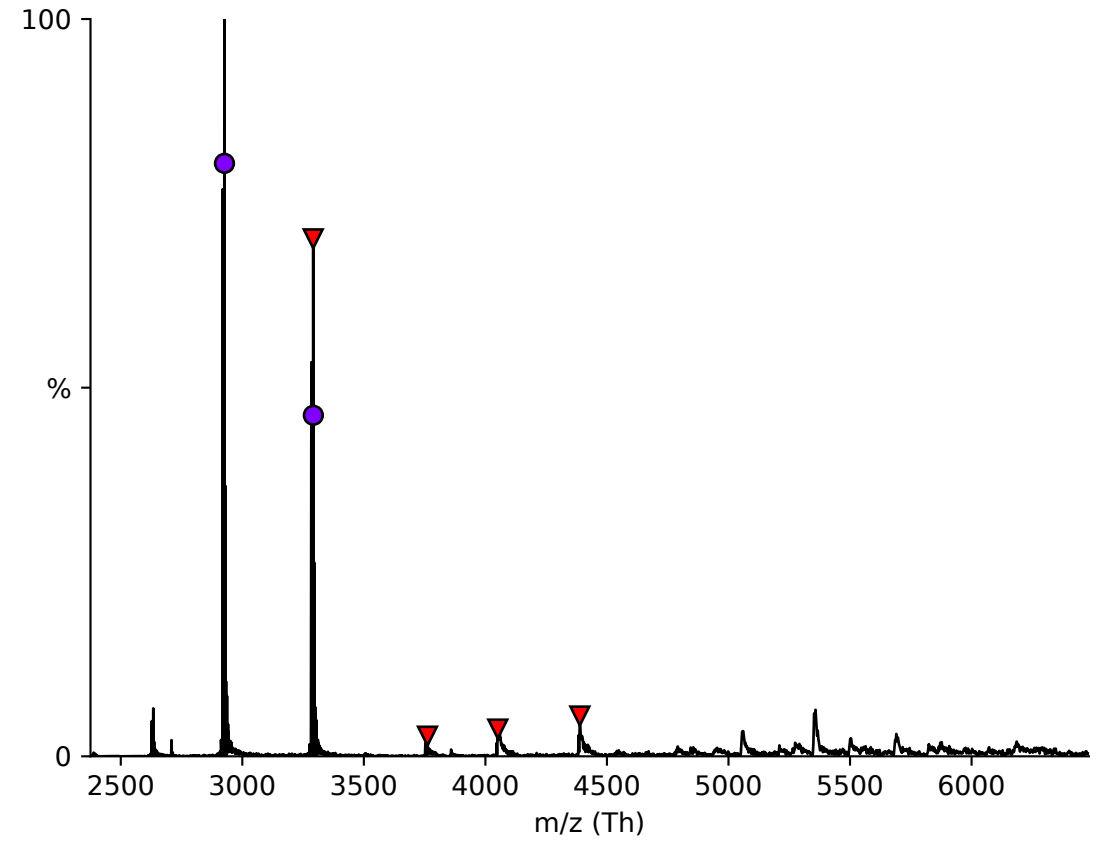

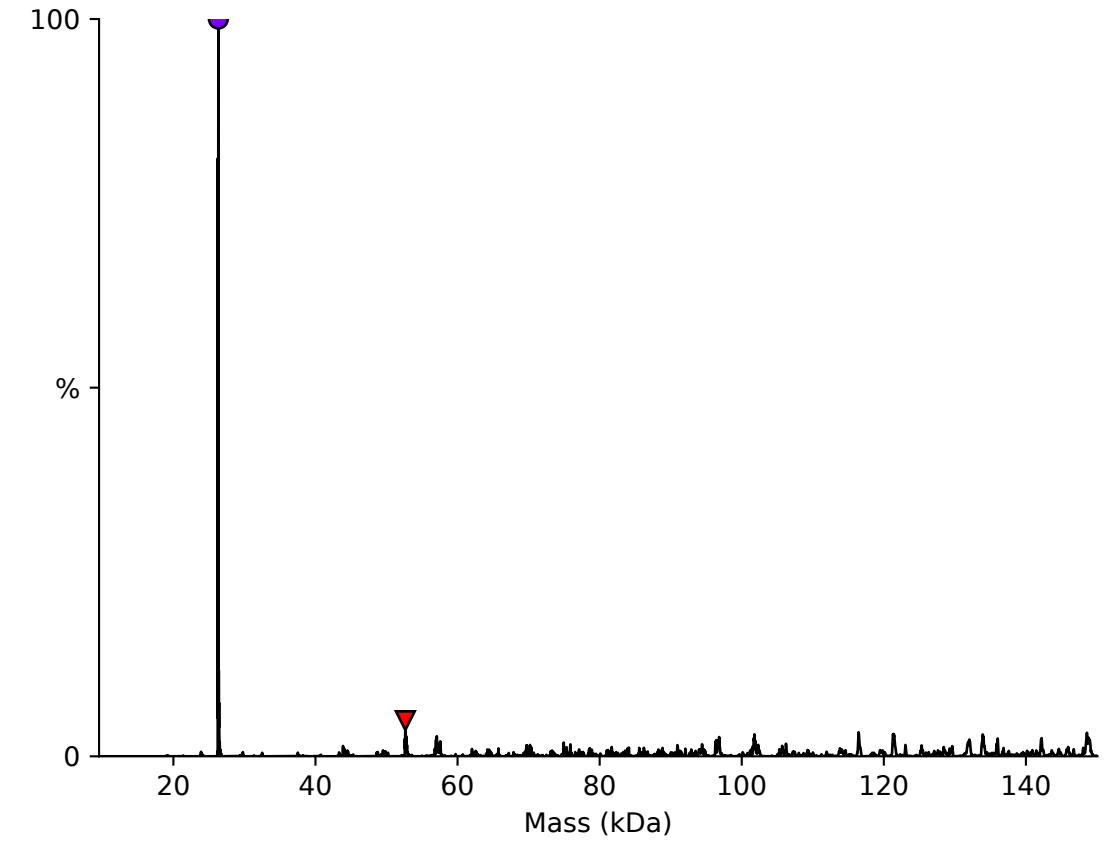

Symbol	Mass (Da)	Intensity[1]	Expected	Difference (Da)	Name/Species/Oligomer
○	26330	100	26333	−3	1[RK601C]
▽	52650	5	46423	6227	1[RK601A]

Only monomers present

[1]Intensity values should not be considered quantitative

hetBGL03-06-05

RK602A = 46429.1 Da RK602B = 18263 Da RK602C = 26230.9 Da
Date Analyzed: Thu, 21 Jul 2022, 16:50 US Mountain Standard Time
File Name: 20220625_RK602_6K_IST060_HCD005_selection

Quality Check:
Average Peaks Score: 0.77

Expected Oligomers:
Passed:
Mixed:
Failed:

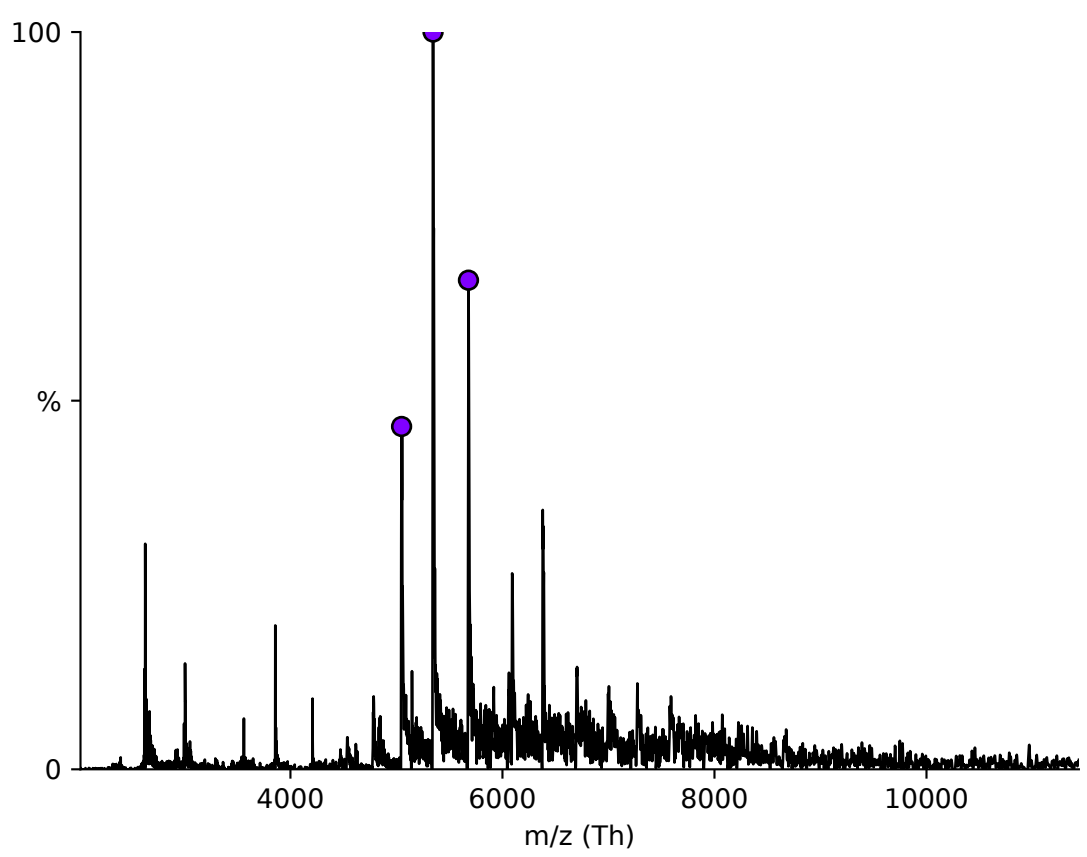

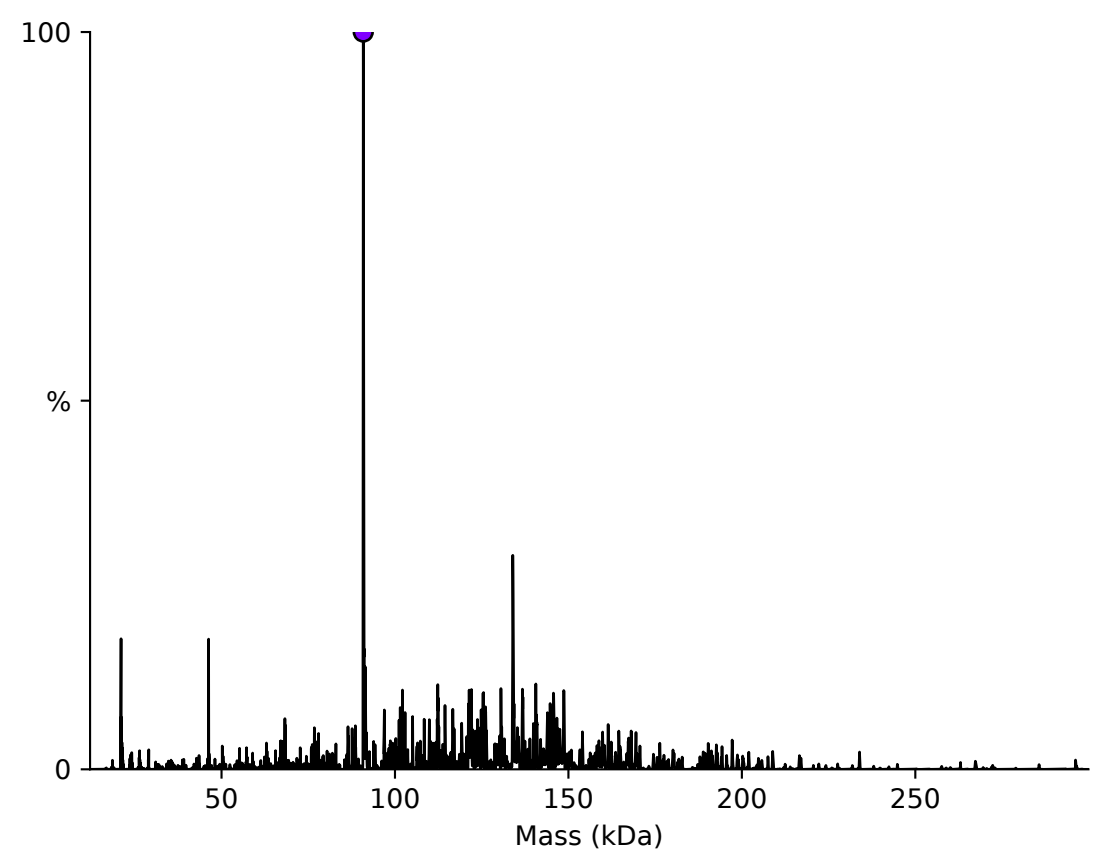

Symbol	Mass (Da)	Intensity[1]	Expected	Difference (Da)	Name/Species/Oligomer
○	90854	100	90923	−69	1[RK602C] 1[RK602B] 1[RK602A]

Heterotrimer is present. The spectrum is very clean, and the mass is close to the expected.

[1] Intensity values should not be considered quantitative

hetBGL03-15-18

RK11_OBE-RO1
Date Analyzed: Tue, 18 Oct 2022, 12:12 Eastern Daylight Time
File Name: 20201106_Ryan_RK11_6k_HCD060_OBE-RO1_selection

Quality Check:
Average Peaks Score: 0.92

Expected Oligomers:
Passed:
Mixed:
Failed:

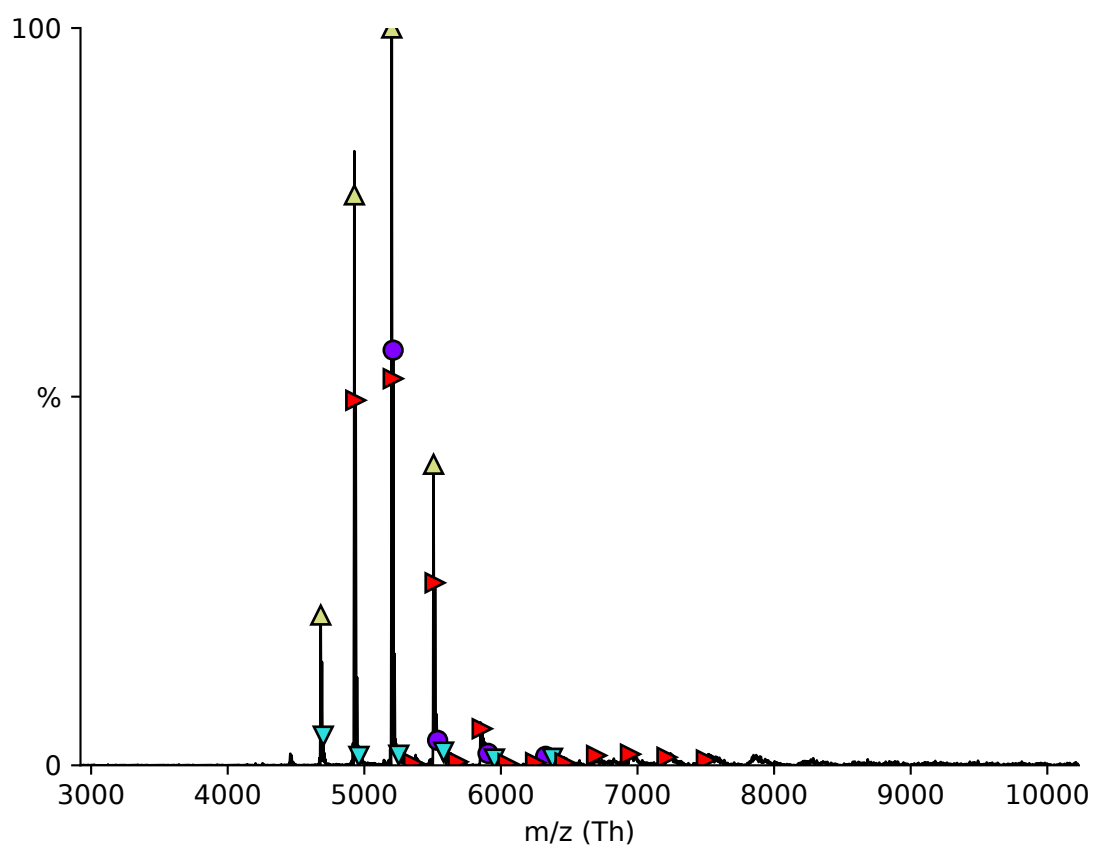

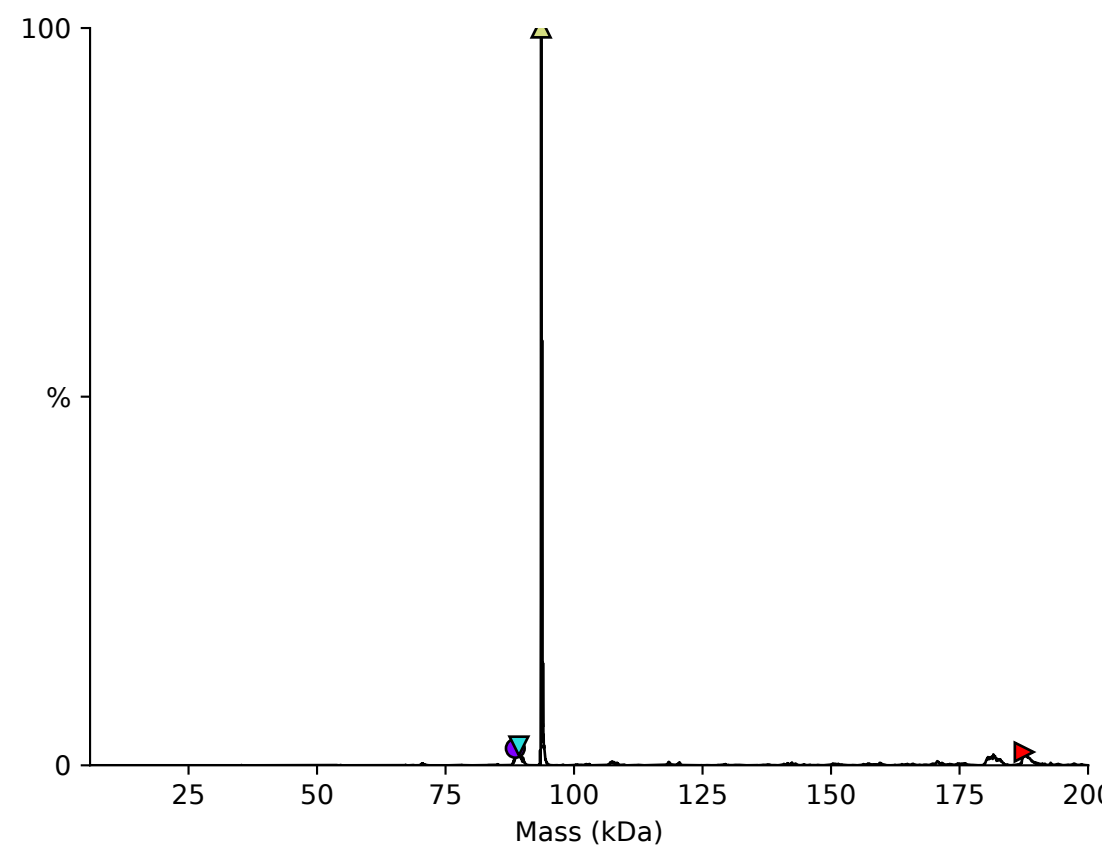

Symbol	Mass (Da)	Intensity[1]	Expected	Difference (Da)	Name/Species/Oligomer
○	88570	2	–	–	-
▽	89270	3	–	–	-
△	93600	100	–	–	ABC
▷	187570	2	–	–	A2B2C2

[1]Intensity values should not be considered quantitative

hetBGL03-18-15

RK604A = 50379.3 Da RK604B = 16282.5 Da RK604C = 27319.9 Da
Date Analyzed: Thu, 21 Jul 2022, 17:01 US Mountain Standard Time
File Name: 20220625_RK604_6K_IST060_HCD005_selection

Quality Check:
Average Peaks Score: 0.64

Expected Oligomers:
Passed:
Mixed:
Failed:

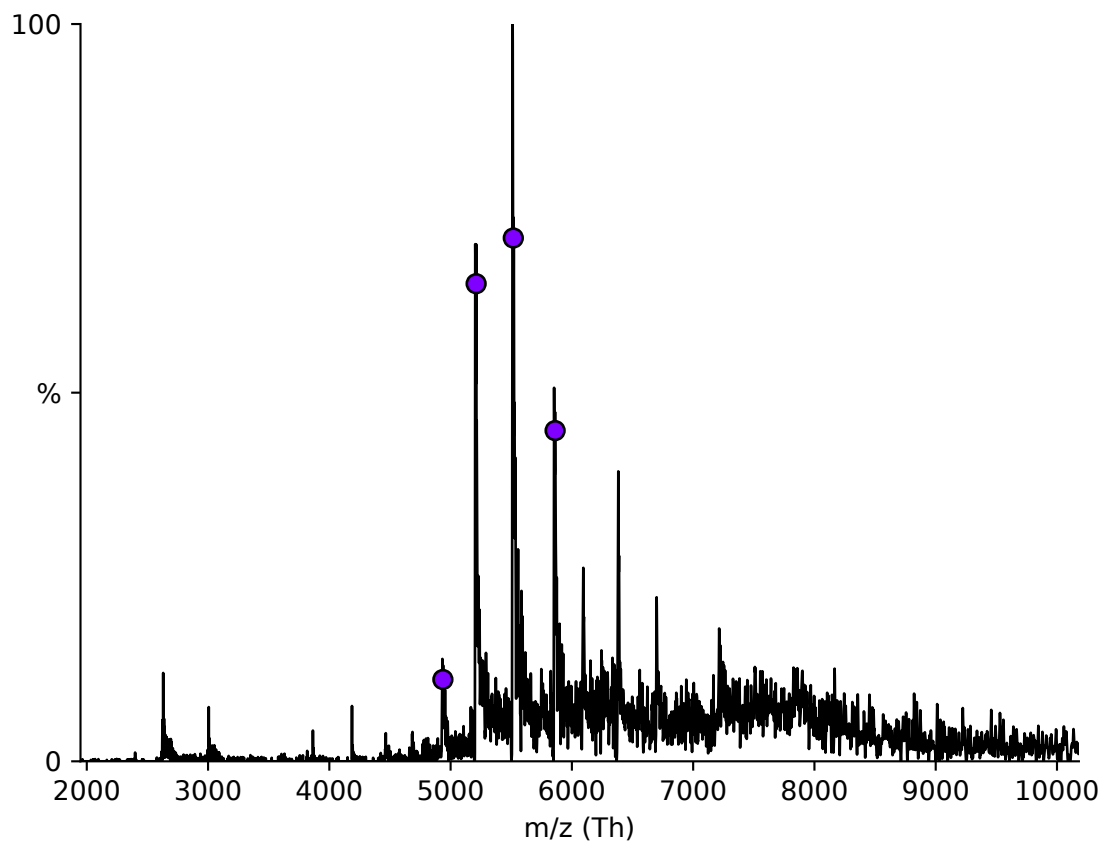

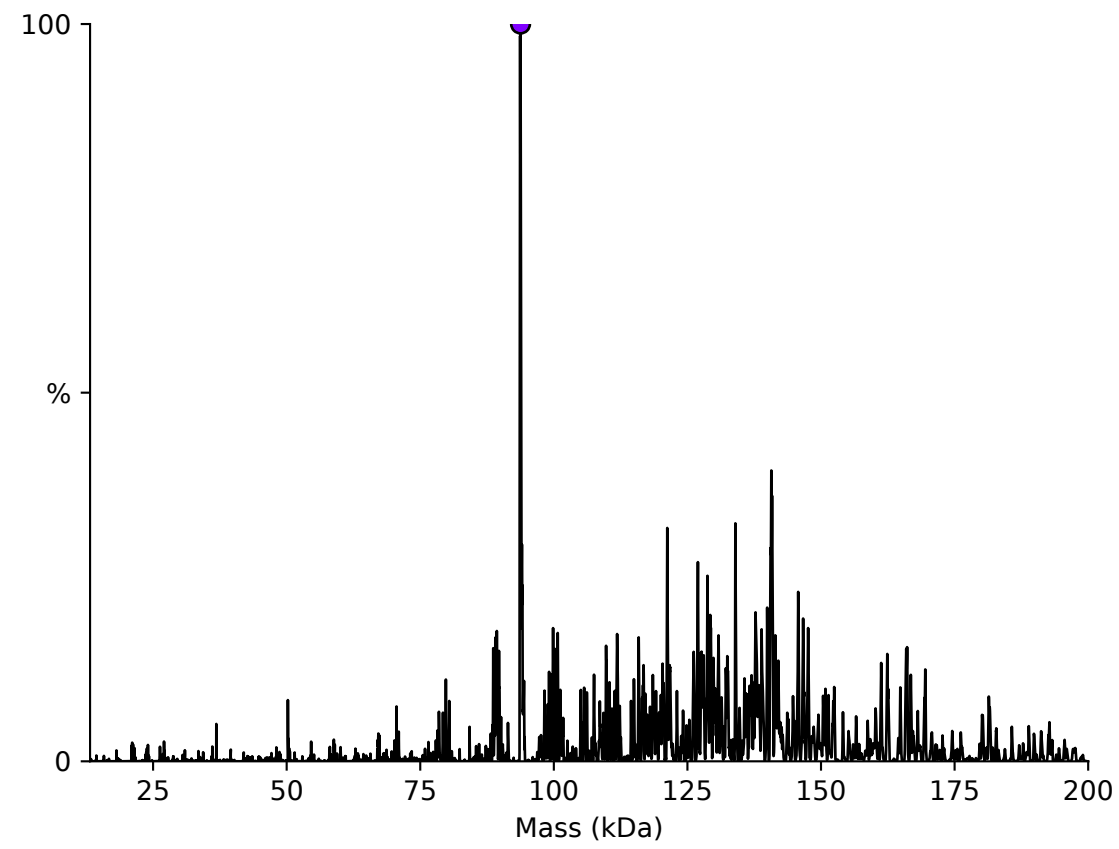

Symbol	Mass (Da)	Intensity[1]	Expected	Difference (Da)	Name/Species/Oligomer
○	93770	100	93912	−142	1[RK604C] 1[RK604B] 1[RK604A]

Heterotrimer is present. Spectrum is very clean

[1] Intensity values should not be considered quantitative

hetBGL08-09-19

RK605A = 50975.9 Da RK605B = 16282.5 Da RK605C = 26350.1 Da
Date Analyzed: Thu, 21 Jul 2022, 17:07 US Mountain Standard Time
File Name: 20220625_RK605_6K_IST060_HCD005_selection

Quality Check:
Average Peaks Score: 0.6

Expected Oligomers:
Passed:
Mixed:
Failed:

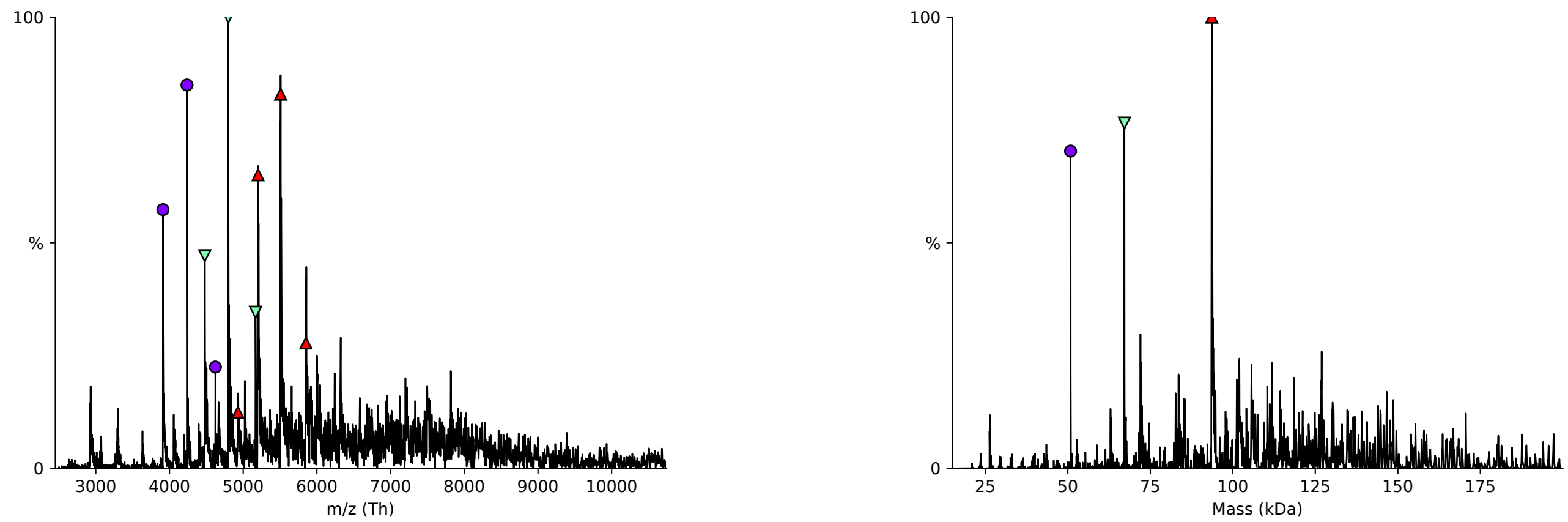

Symbol	Mass (Da)	Intensity[1]	Expected	Difference (Da)	Name/Species/Oligomer
◯	50838	70	50976	−138	1[RK605A]
▽	67165	77	67258	−93	1[RK605A] 1[RK605B]
△	93619	100	93608	10	1[RK605A] 1[RK605B] 1[RK605C]

Heterotrimer is present

[1] Intensity values should not be considered quantitative

hetBGL08-19-09

RK606A = 50146.2 Da RK606B = 16214.2 Da RK606C = 27516.5 Da
Date Analyzed: Thu, 21 Jul 2022, 17:22 US Mountain Standard Time
File Name: 20220625_RK606_6K_IST060_HCD005_selection

Quality Check:
Average Peaks Score: 0.75

Expected Oligomers:
Passed:
Mixed:
Failed:

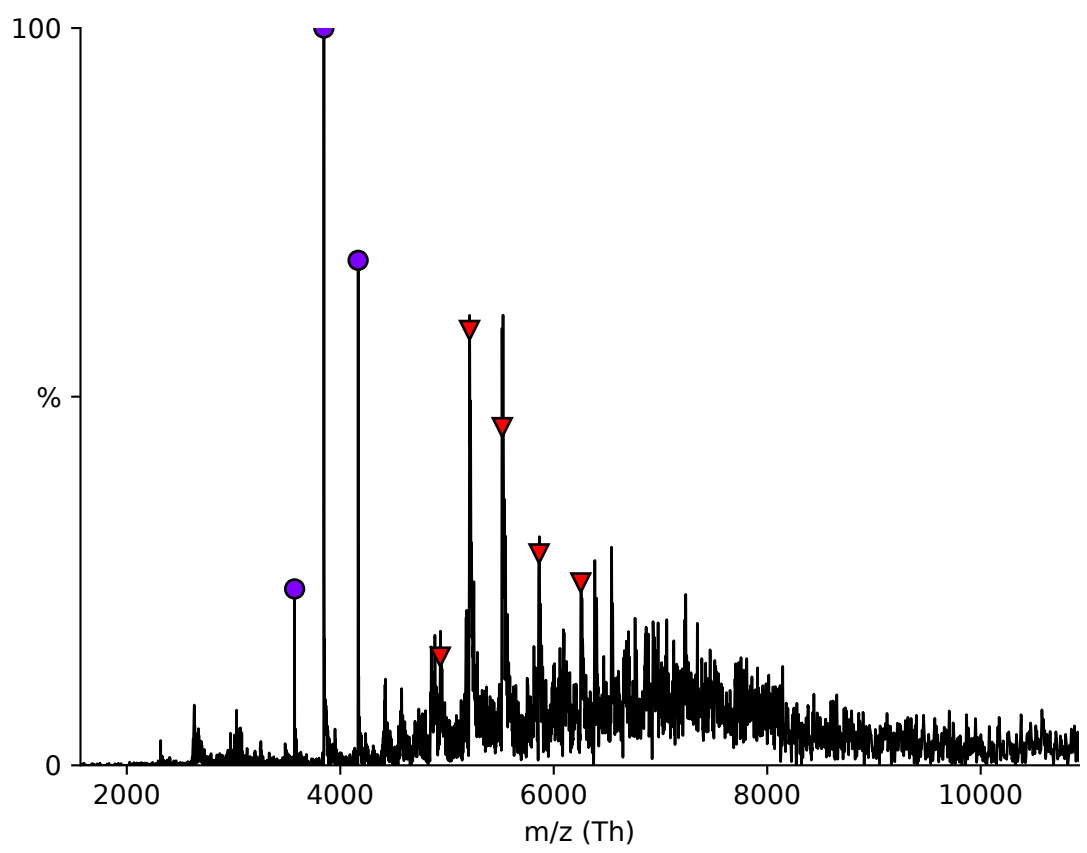

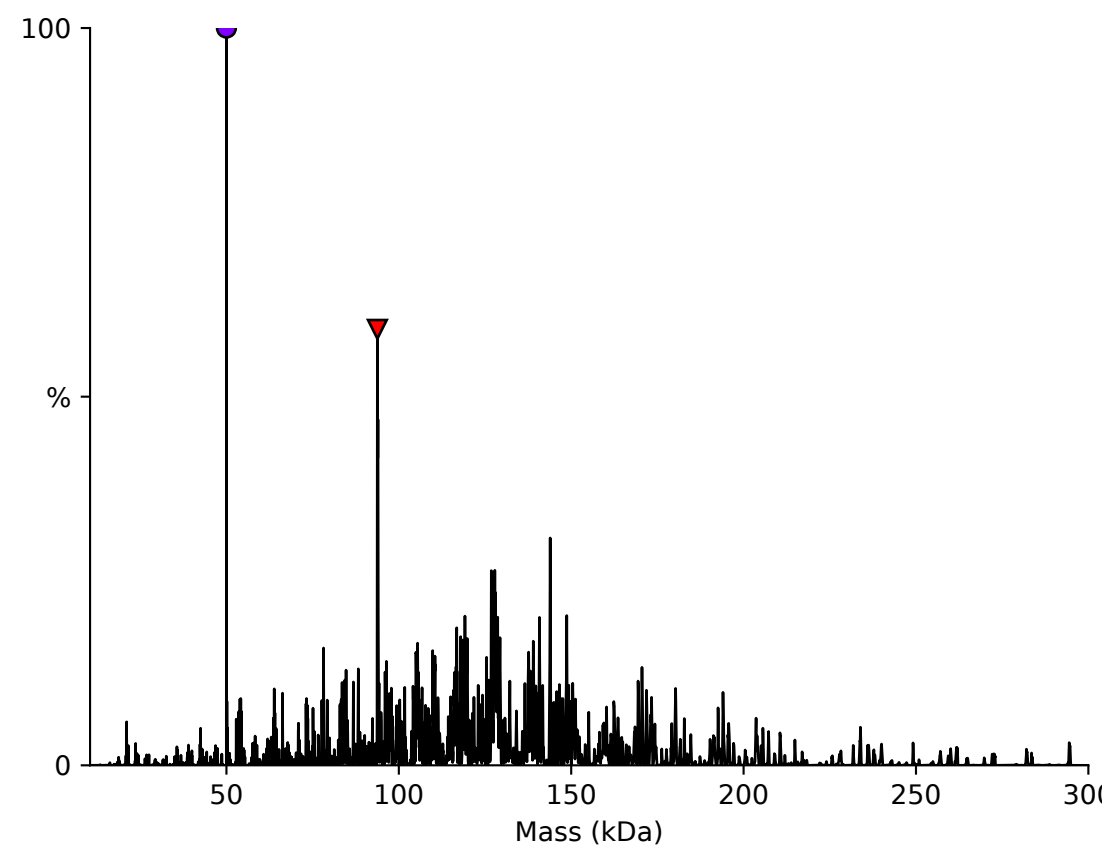

Symbol	Mass (Da)	Intensity[1]	Expected	Difference (Da)	Name/Species/Oligomer
◯	49994	100	50146	−152	1[RK606A]
▽	93781	59	93877	−96	1[RK606C] 1[RK606B] 1[RK606A]

Heterotrimer is present

[1] Intensity values should not be considered quantitative

hetBGL0-17-18

RK645A = 25508.4 Da RK645B = 19551.9 Da RK645C = 49042.5 Da
Date Analyzed: Thu, 21 Jul 2022, 17:27 US Mountain Standard Time
File Name: 20220625_RK645_6K_IST060_HCD005_selection

Quality Check:
Average Peaks Score: 0.65

Expected Oligomers:
Passed:
Mixed:
Failed:

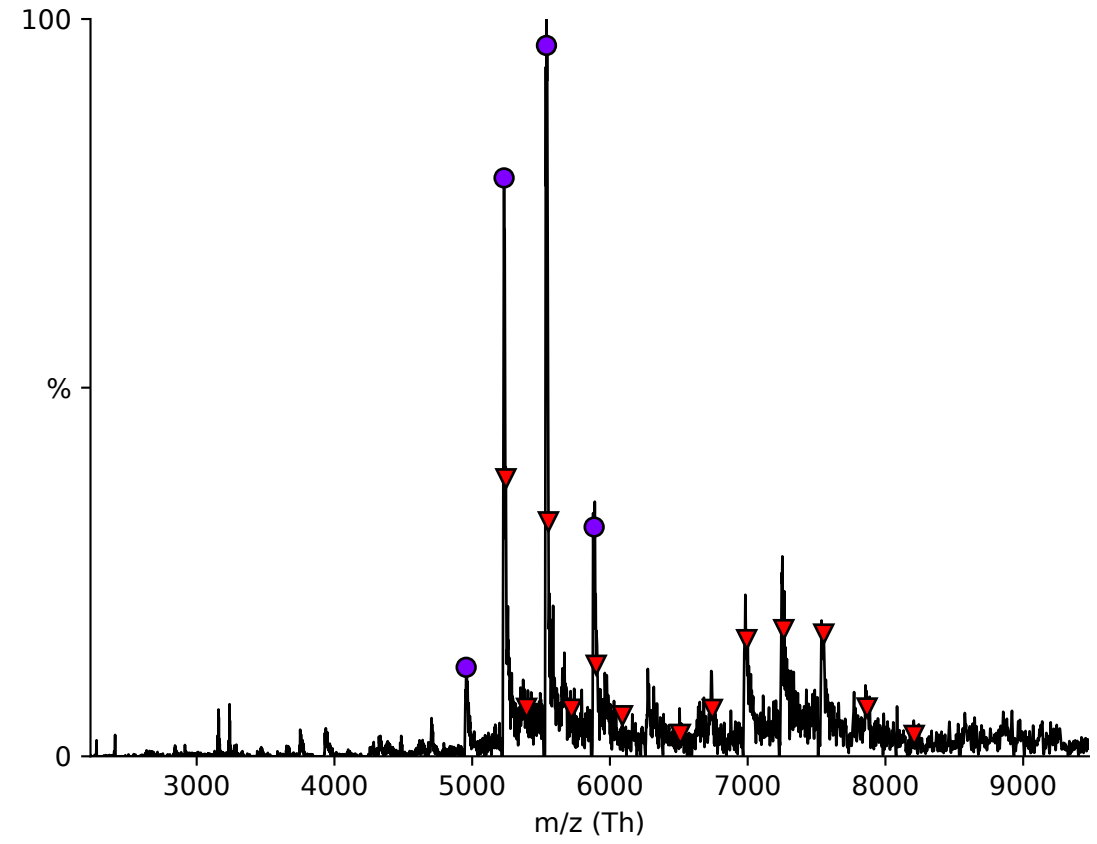

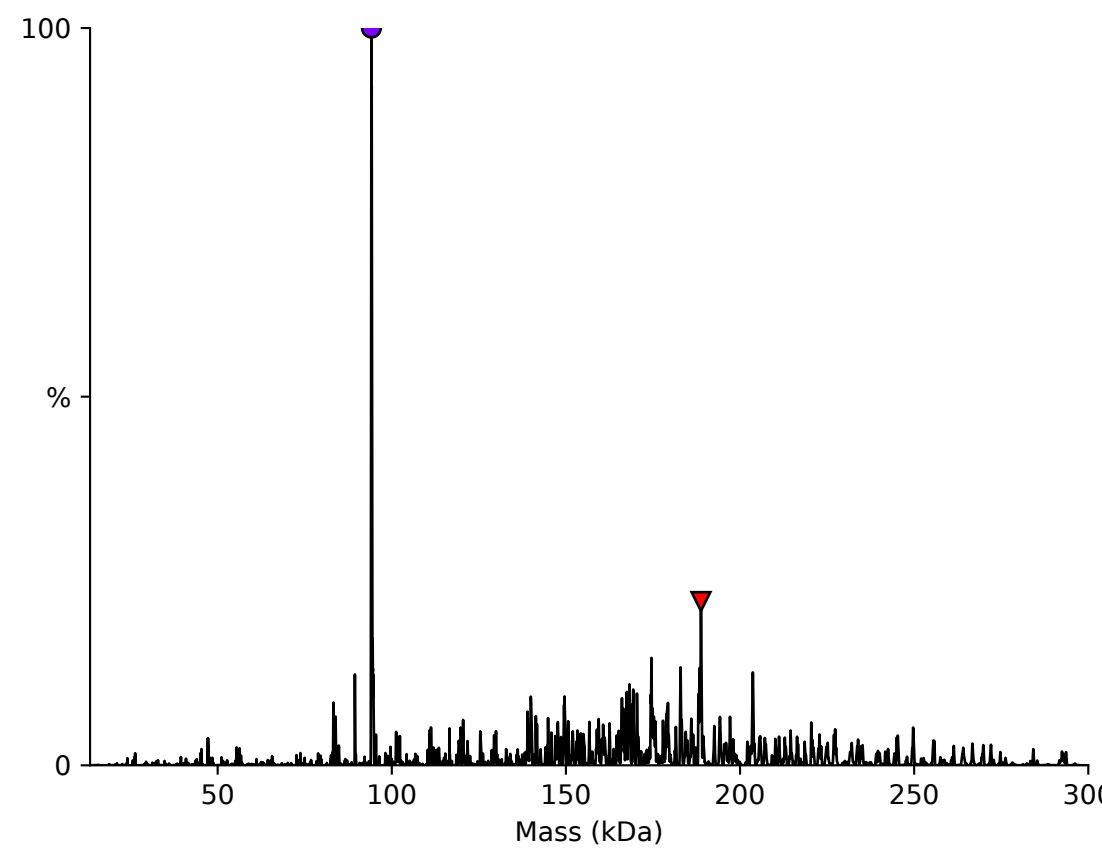

Symbol	Mass (Da)	Intensity[1]	Expected	Difference (Da)	Name/Species/Oligomer
○	94149	100	94103	46	1[RK645C] 1[RK645B] 1[RK645A]
▽	188781	22	188206	575	2[RK645C] 2[RK645B] 2[RK645A]

Heterotrimer is present. Also, there is a hexamer made of two heterotrimers

[1]Intensity values should not be considered quantitative

hetBGL0-17-19

RK646A = 25700.6 Da RK646B = 19552.9 Da RK646C = 49072.6 Da
Date Analyzed: Thu, 21 Jul 2022, 17:32 US Mountain Standard Time
File Name: 20220625_RK646_6K_IST060_HCD005_selection

Quality Check:
Average Peaks Score: 0.79

Expected Oligomers:
Passed:
Mixed:
Failed:

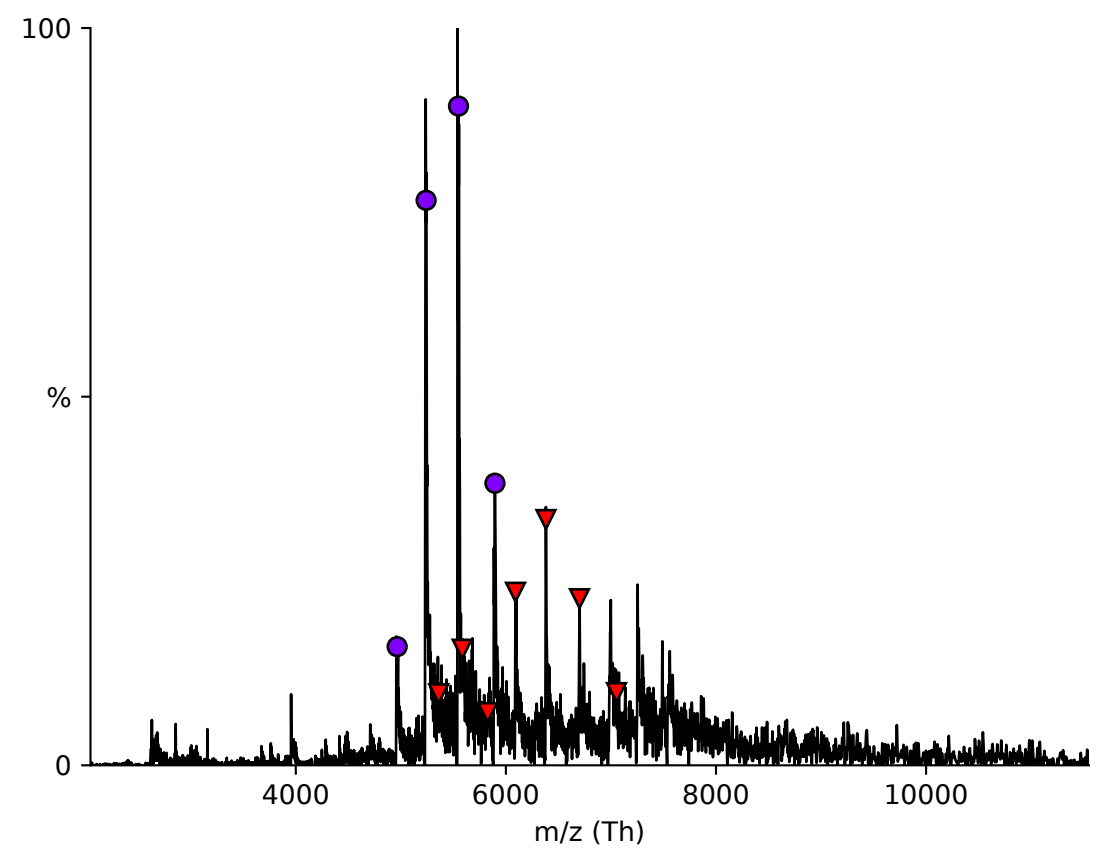

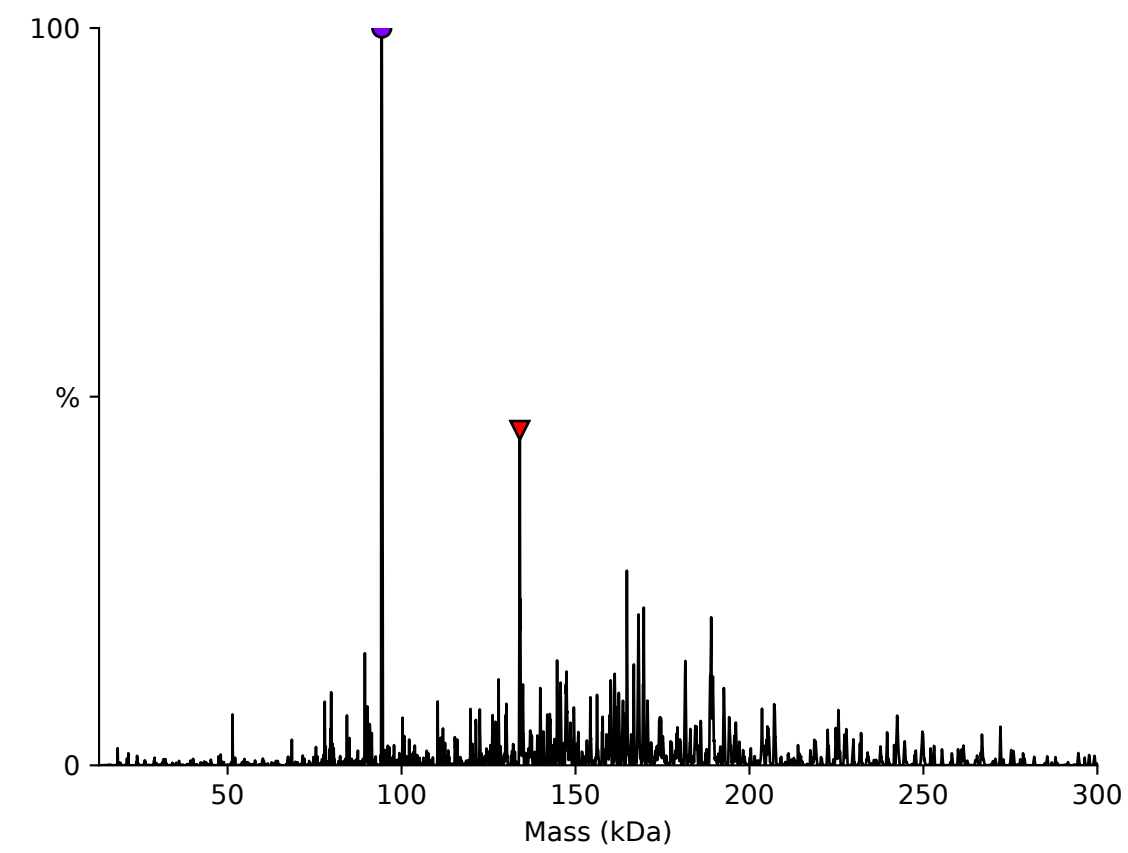

Symbol	Mass (Da)	Intensity[1]	Expected	Difference (Da)	Name/Species/Oligomer
○	94314	100	94326	−12	1[RK646C] 1[RK646B] 1[RK646A]
▽	133989	45	137251	−3262	

Heterotrimer is present

[1]Intensity values should not be considered quantitative

hetBGL0-18-17

RK647A = 25622.3 Da RK647B = 19571.9 Da RK647C = 49001.4 Da
Date Analyzed: Thu, 21 Jul 2022, 17:36 US Mountain Standard Time
File Name: 20220625_RK647_6K_IST060_HCD005_selection

Quality Check:
Average Peaks Score: 0.7

Expected Oligomers:
Passed:
Mixed:
Failed:

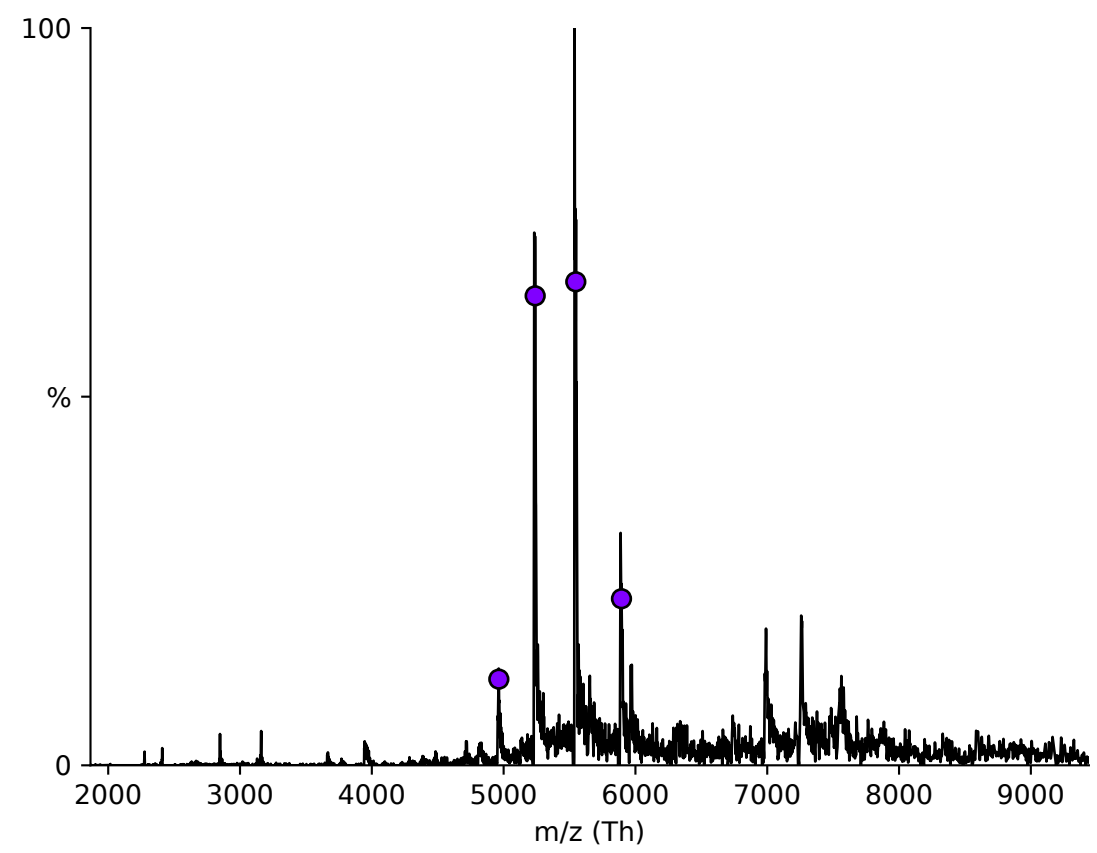

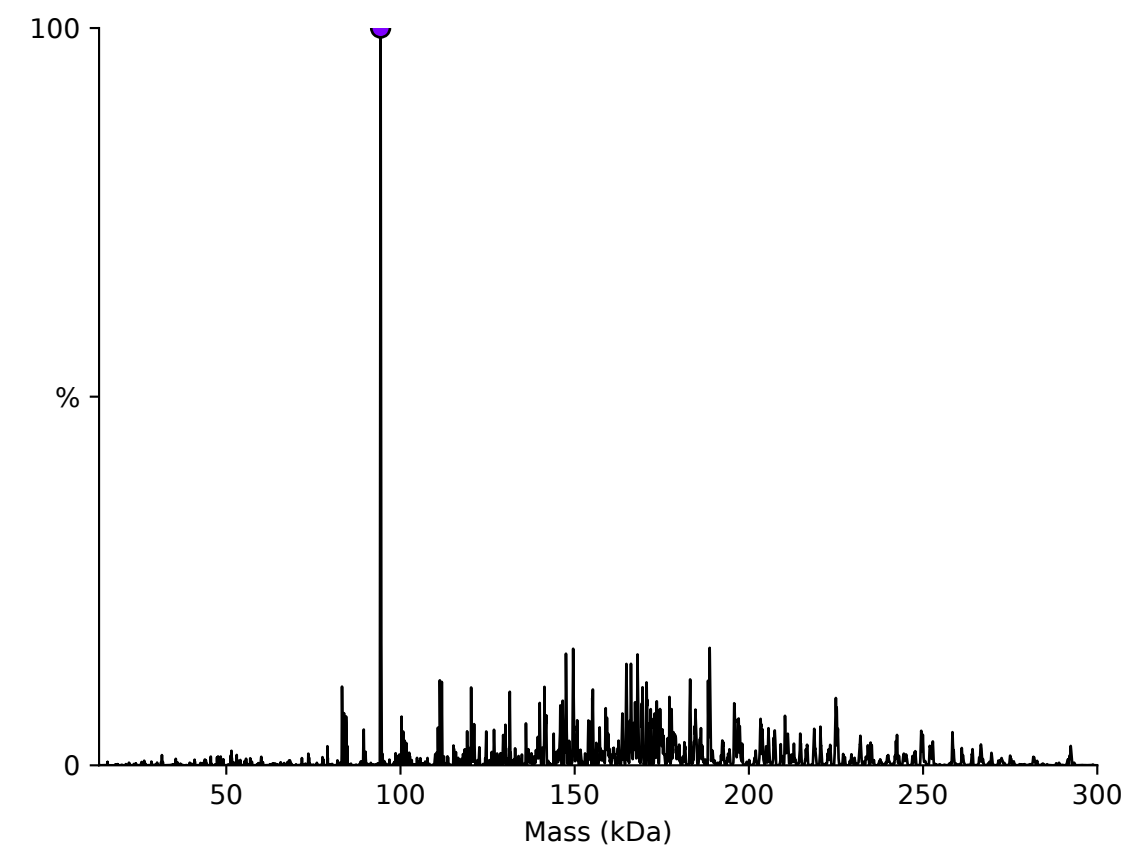

Symbol	Mass (Da)	Intensity[1]	Expected	Difference (Da)	Name/Species/Oligomer
○	94290	100	94267	23	1[RK647C] 1[RK647B] 1[RK647A]

Heterotrimer is present. Spectrum is very clean

[1]Intensity values should not be considered quantitative

hetBGL0-18-19

RK648A = 25700.6 Da RK648B = 19543.9 Da RK648C = 49018.3 Da
Date Analyzed: Thu, 21 Jul 2022, 17:41 US Mountain Standard Time
File Name: 20220625_RK648_6K_IST060_HCD005_selection

Quality Check:
Average Peaks Score: 0.3

Expected Oligomers:
Passed:
Mixed:
Failed:

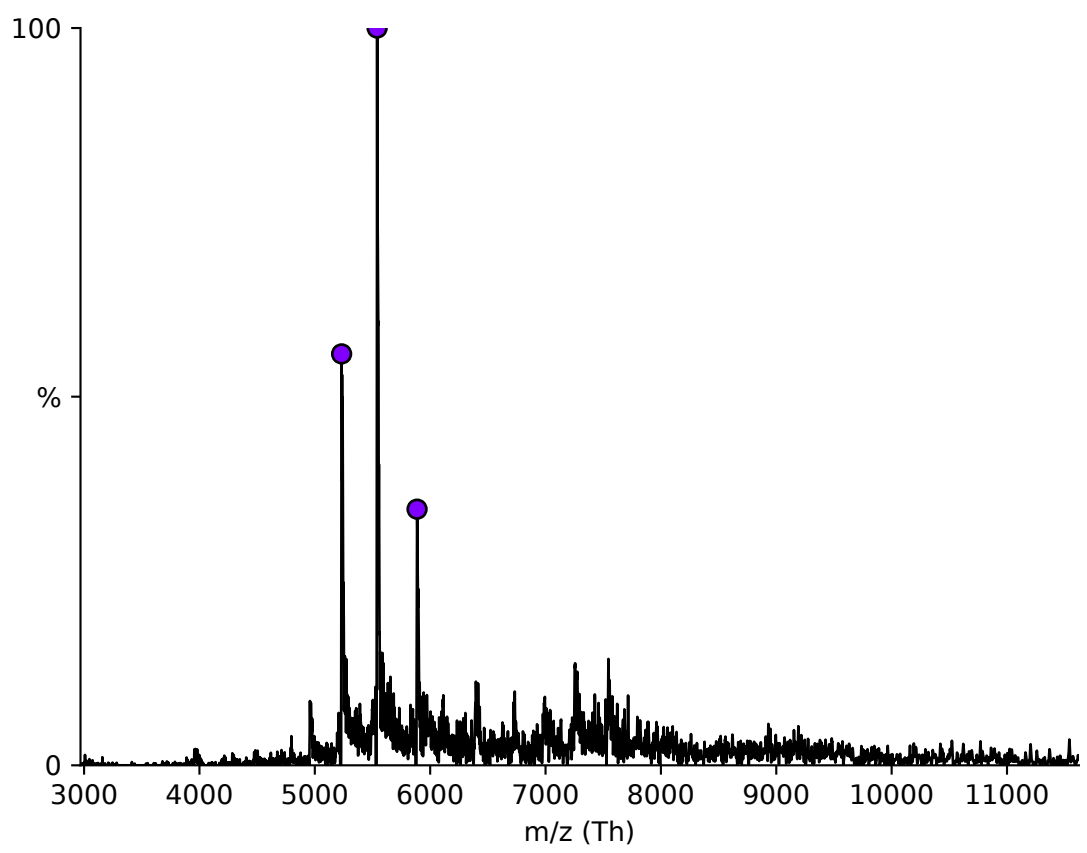

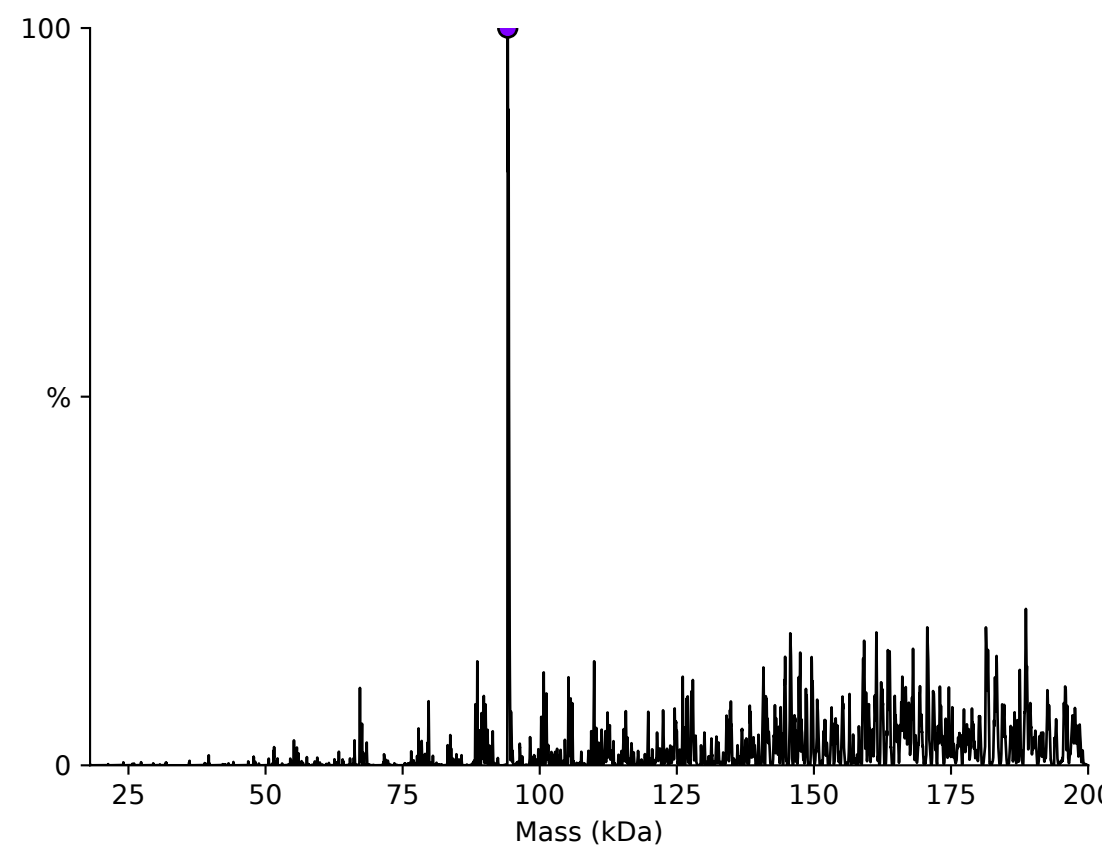

Symbol	Mass (Da)	Intensity[1]	Expected	Difference (Da)	Name/Species/Oligomer
○	94164	100	94263	−99	1[RK648C] 1[RK648B] 1[RK648A]

Heterotrimer is present

[1]Intensity values should not be considered quantitative

hetBGL0-19-17

RK649A = 25620.5 Da RK649B = 19574.8 Da RK649C = 49108.7 Da
Date Analyzed: Thu, 21 Jul 2022, 17:45 US Mountain Standard Time
File Name: 20220625_RK649_6K_IST060_HCD005_selection

Quality Check:
Average Peaks Score: 0.59

Expected Oligomers:
Passed:
Mixed:
Failed:

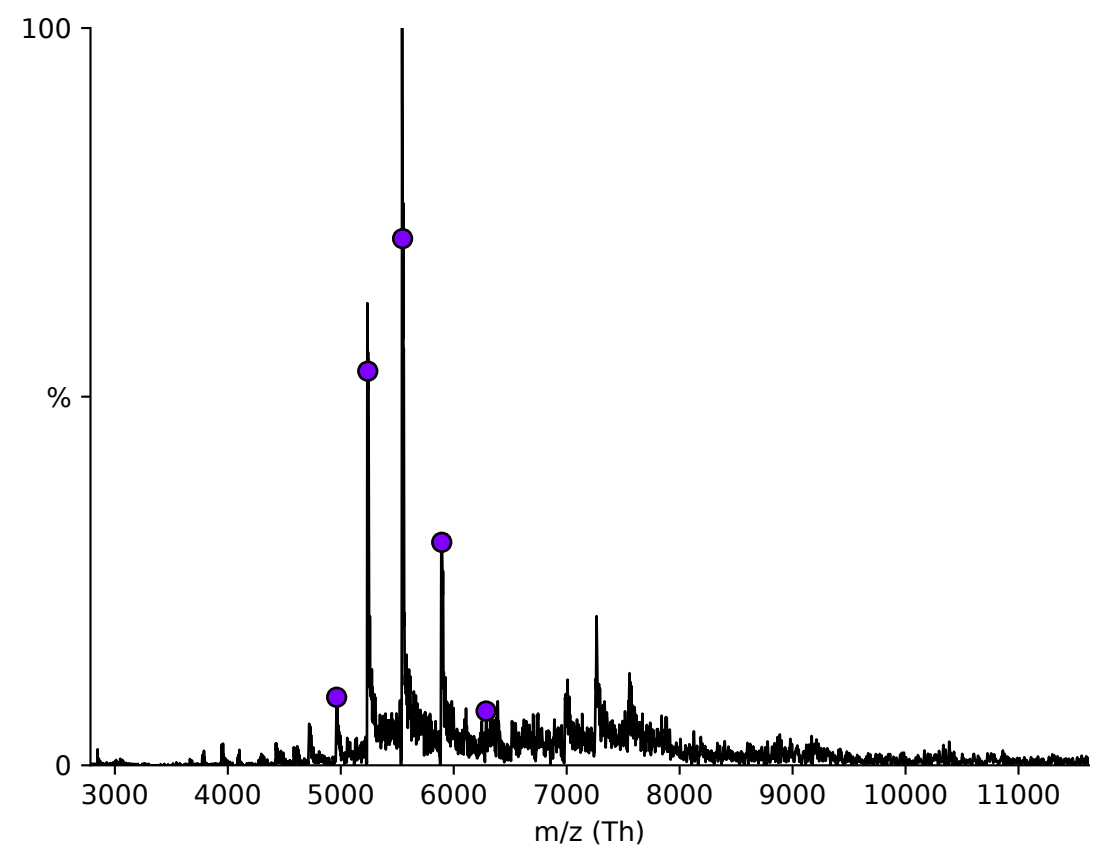

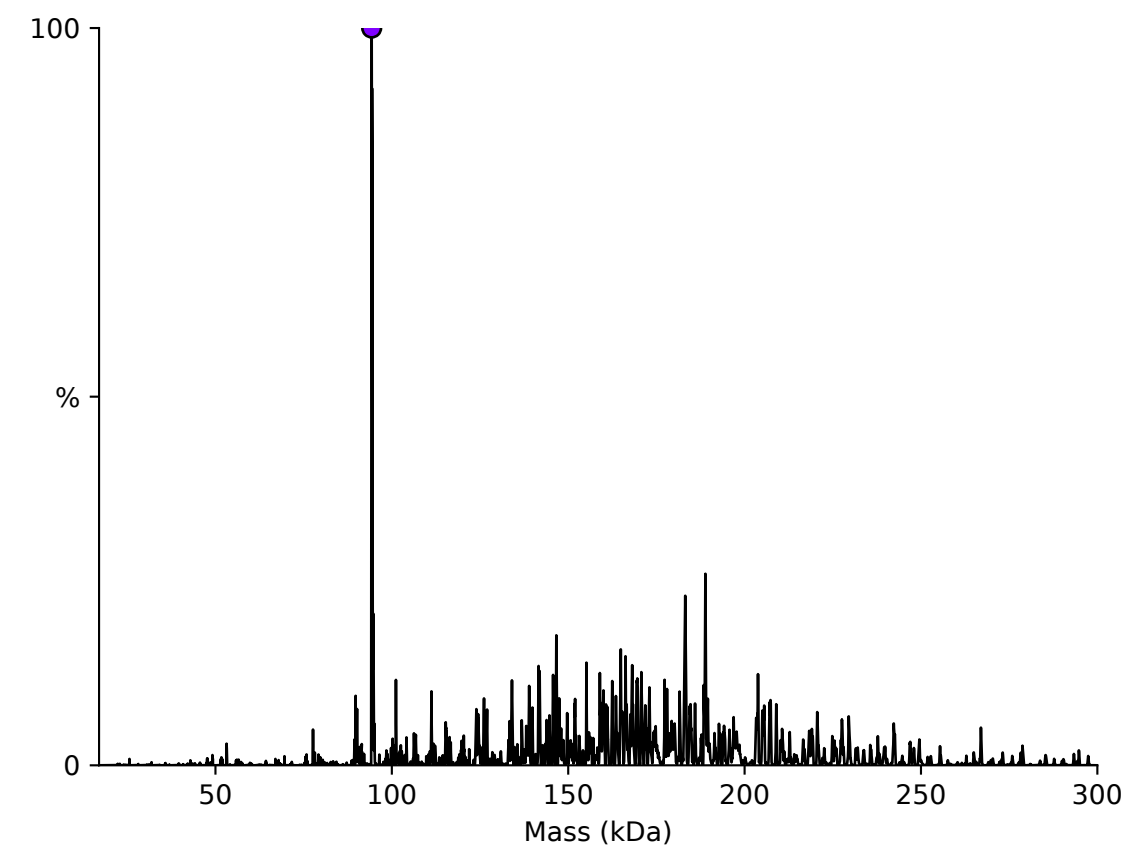

Symbol	Mass (Da)	Intensity[1]	Expected	Difference (Da)	Name/Species/Oligomer
○	94284	100	94304	−20	1[RK649A] 1[RK649B] 1[RK649C]

Heterotrimer is present

[1] Intensity values should not be considered quantitative

hetBGL0-19-18

RK650A = 25507.3 Da RK650B = 19616.9 Da RK650C = 490494.7 Da
Date Analyzed: Thu, 21 Jul 2022, 17:51 US Mountain Standard Time
File Name: 20220625_RK650_6K_IST060_HCD005_selection

Quality Check:
Average Peaks Score: 0.26

Expected Oligomers:
Passed:
Mixed:
Failed:

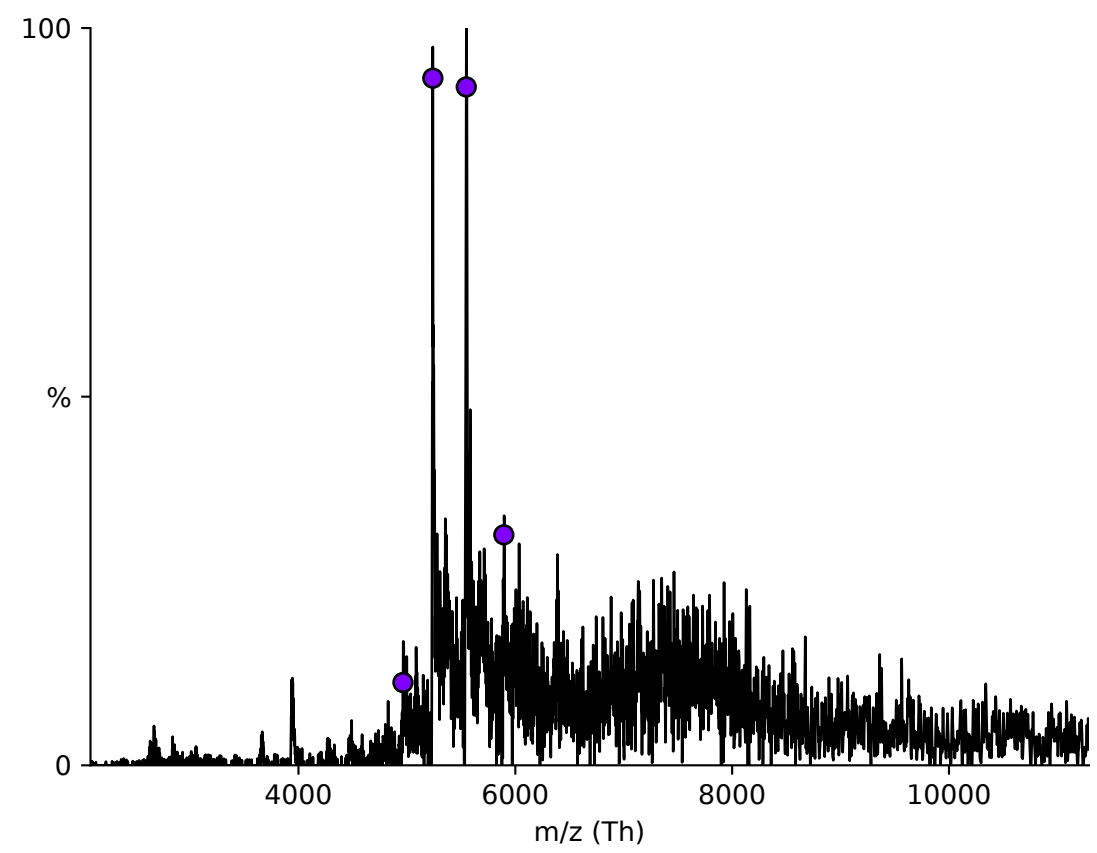

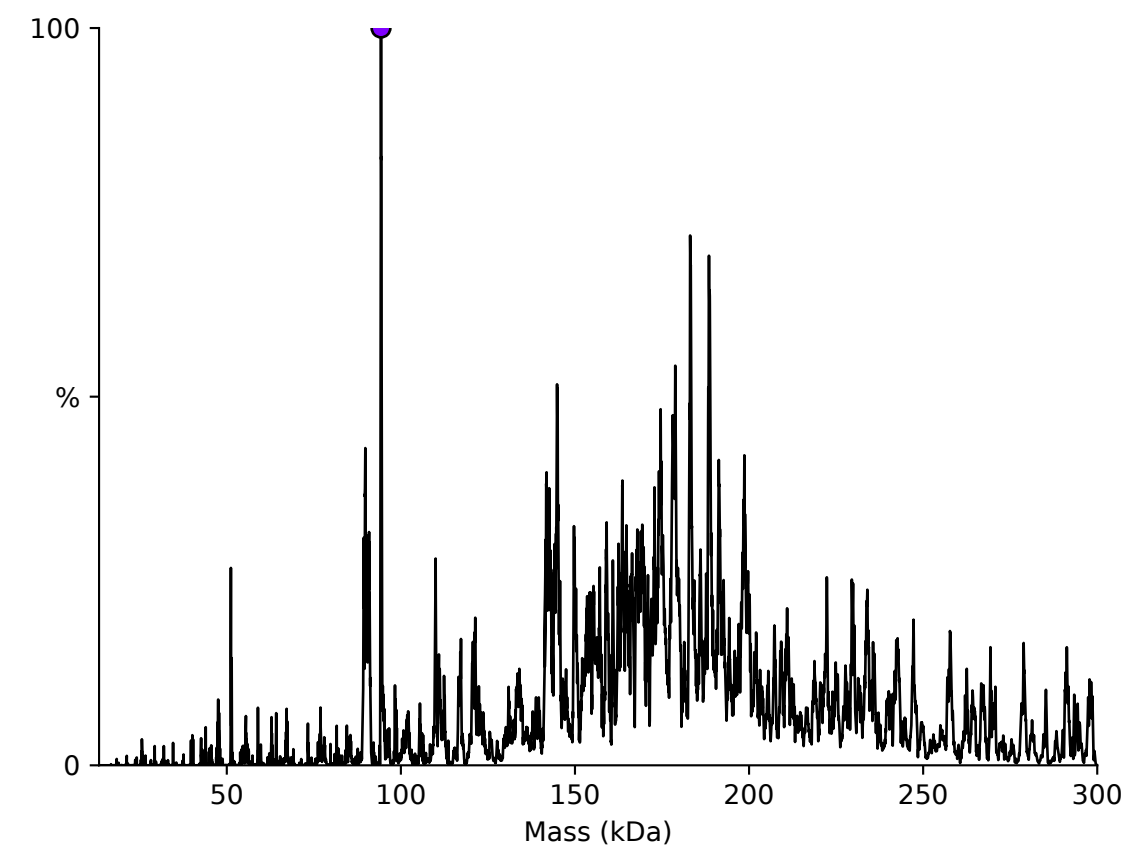

Symbol	Mass (Da)	Intensity[1]	Expected	Difference (Da)	Name/Species/Oligomer
○	94291	100	94219	71	A

Heterotrimer is present

[1] Intensity values should not be considered quantitative

hetBGL17-18-19

RK651 a =25881 b=19084 c =48896
Date Analyzed: Tue, 02 Aug 2022, 12:49 Eastern Daylight Time
File Name: 20210318_RyanK_RK651_6k_IST030_HCD120_selection

Quality Check:
Average Peaks Score: 0.76

Expected Oligomers:
Passed:
Mixed:
Failed:

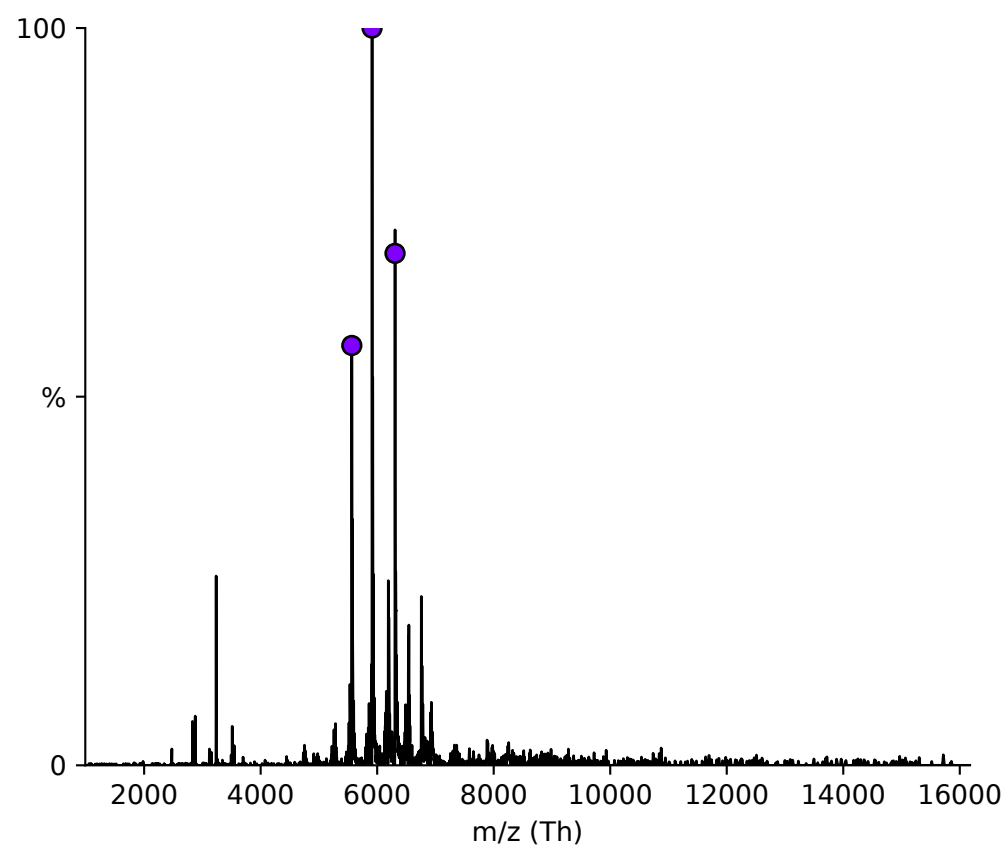

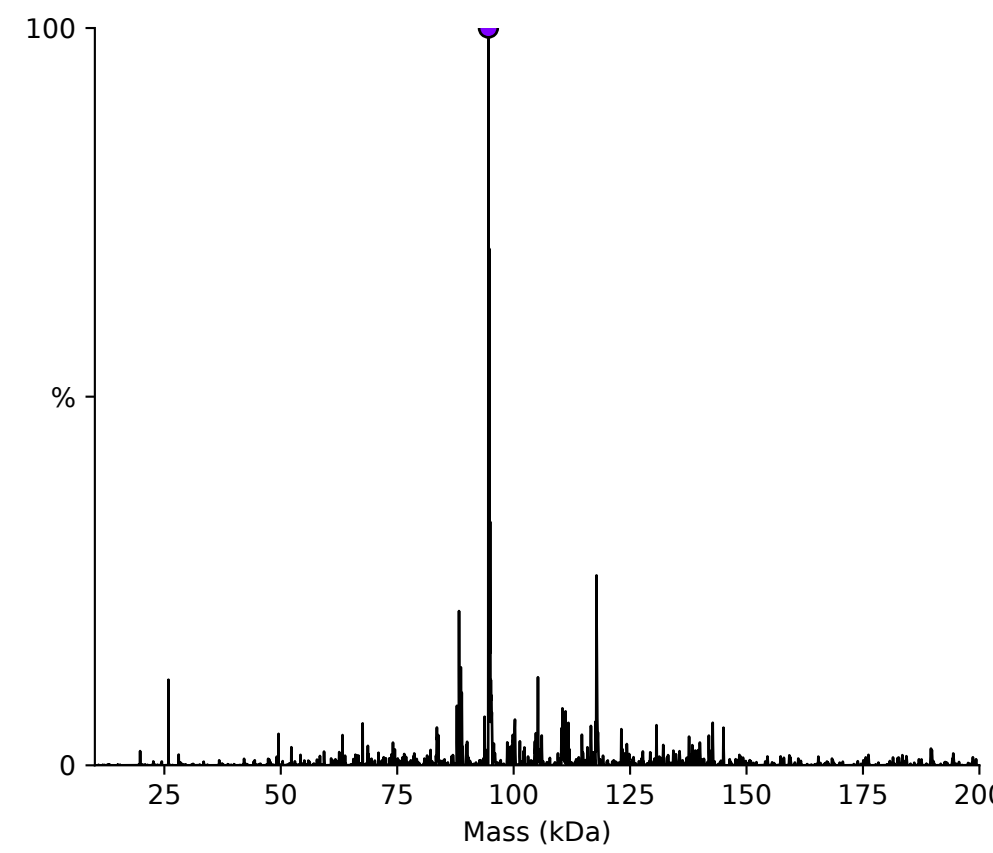

Symbol	Mass (Da)	Intensity[1]	Expected	Difference (Da)	Name/Species/Oligomer
○	94580	100	94581	−1	1[C] 1[B] 1[A]

[1]Intensity values should not be considered quantitative

hetBGL17-19-18

RK652
expected: ABC
masses A=25792, B=19916, C=48944 Da
Date Analyzed: Tue, 18 Oct 2022, 10:44 Eastern Daylight Time
File Name: 20220625_RK652_6K_IST060_HCD005_selection

Quality Check:
Average Peaks Score: 0.67

Expected Oligomers:
Passed:
Mixed:
Failed:

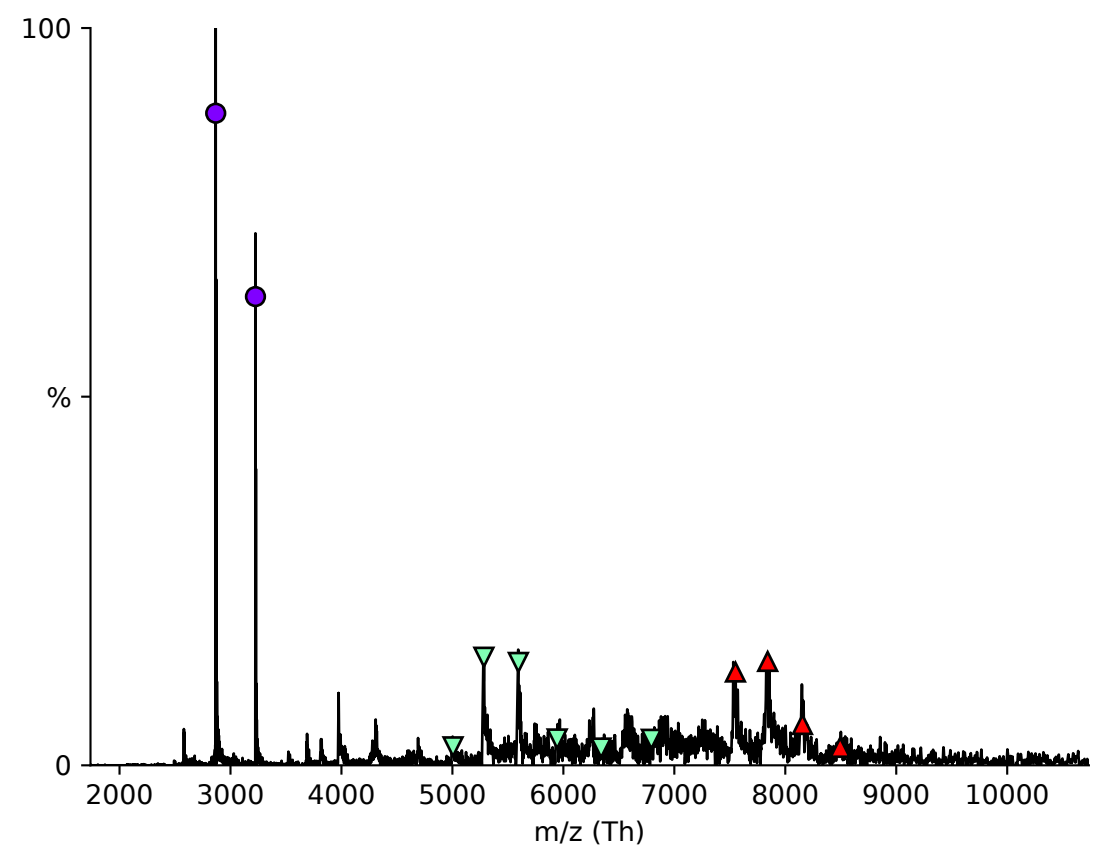

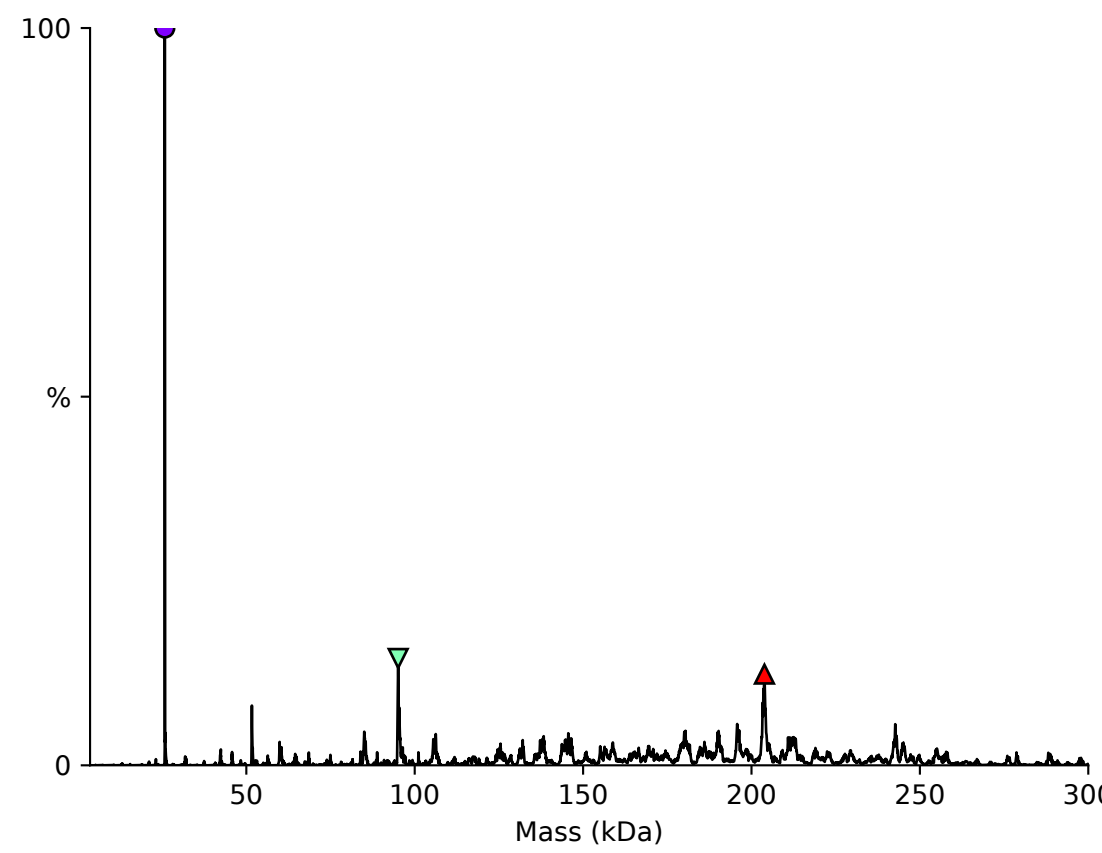

Symbol	Mass (Da)	Intensity[1]	Expected	Difference (Da)	Name/Species/Oligomer
○	25793	100	25792	1	1[A]
▽	95091	14	94652	439	1[C] 1[B] 1[A]
△	203843	12	206580	−2737	3[C] 3[B]

[1]Intensity values should not be considered quantitative

hetBGL03-15-18_reloop

Laboratory: Wysocki-AN
Client: Baker-Ryan
Shipment Date: 4/28/2021
Data Recorded: Fri, 30 Apr 2021, 23:11
Instrument: Q Exactive UHMR Orbitrap
Study: OBE 200 mM AmAc, RO22
Sample Conditions: 25mM Tris pH8, 300mM NaCl, 10% glycerol, 300mM Imidazole, 10mM EDTA
Concentration (ug/uL): 1.0
Injection (uL): 0.6
Comment:
Date Analyzed: Tue, 11 May 2021, 12:13 Eastern Daylight Time
File Name: 20210429_Ryan_RK774RAW_6k_HCD120

Quality Check:
Average Peaks Score: 0.92

Expected Oligomers:
Passed:
Mixed:
Failed:

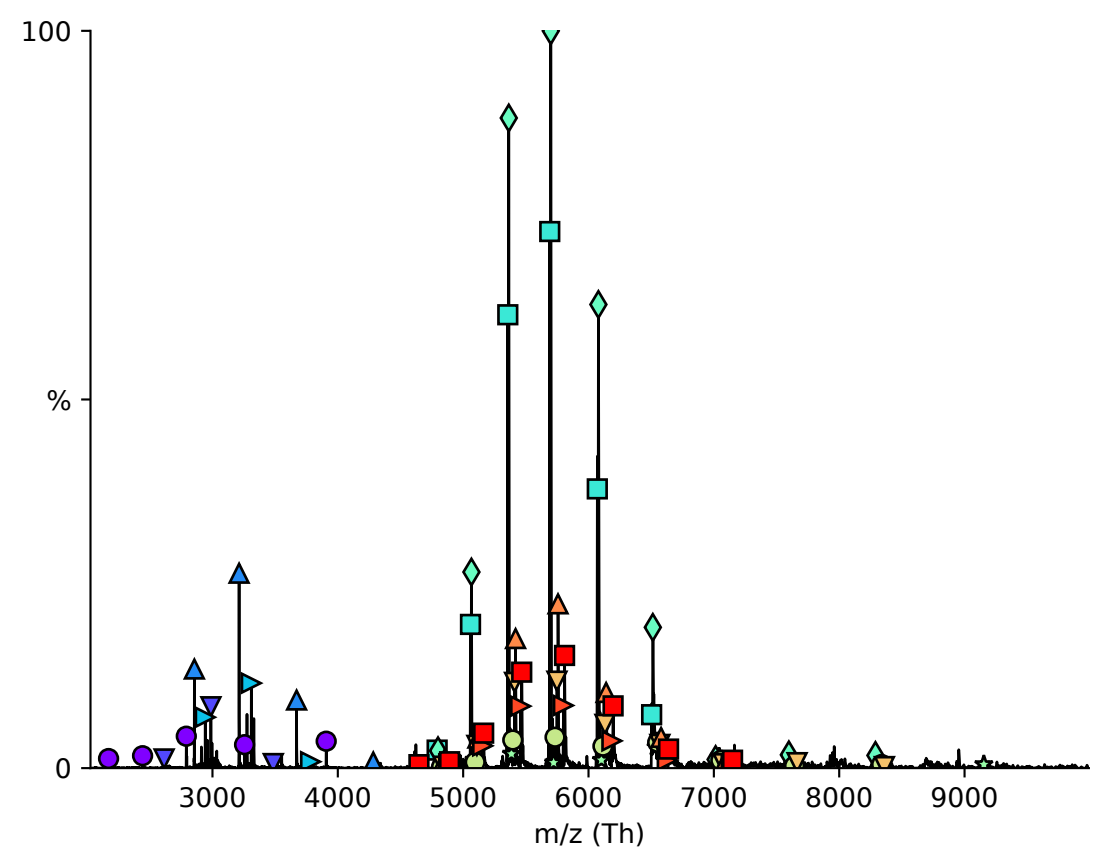

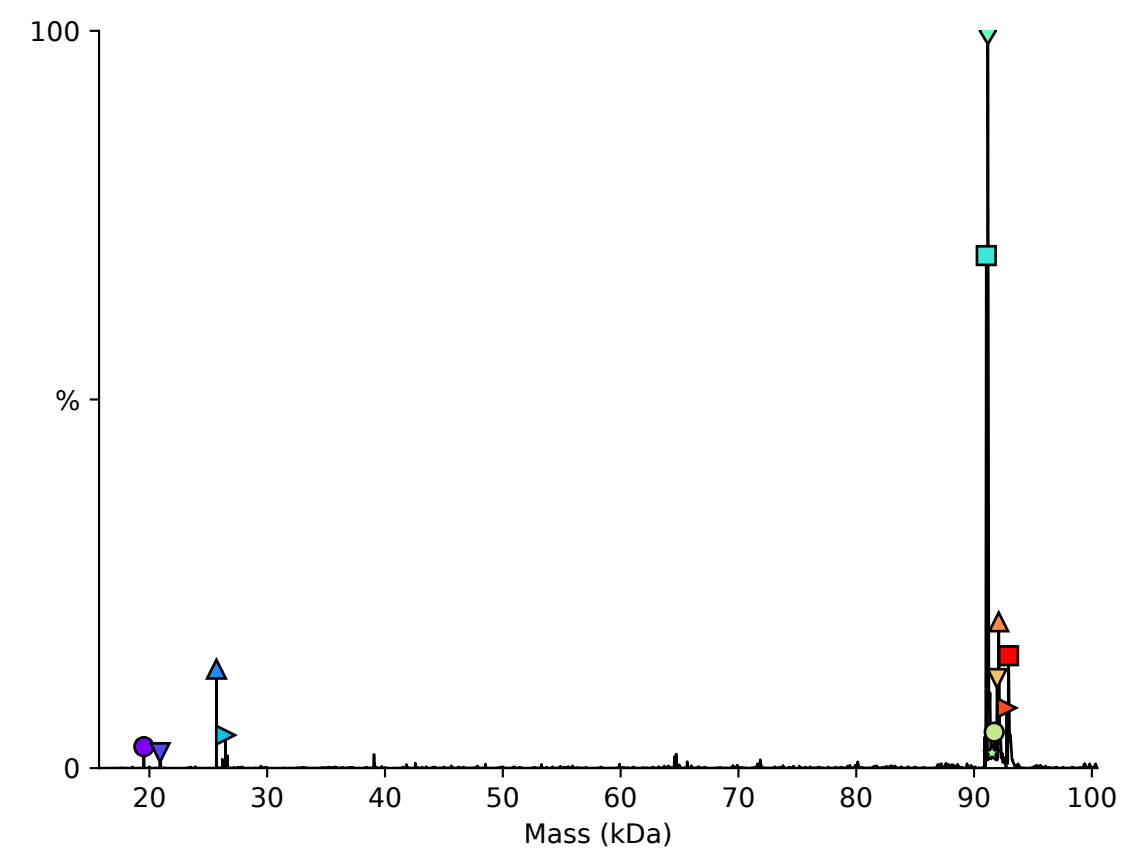

Symbol	Mass (Da)	Intensity[1]	Expected	Difference (Da)	Name/Species/Oligomer
○	19531	3	19531	0	1[+Met] 1[RK774r-B]
▽	20906	2	19531	1375	
△	25691	13	25692	−1	1[RK774r-A]
▷	26471	4	25823	648	
□	91036	70	92653	−1617	
◊	91169	100	92653	−1484	
☆	91530	2	92653	−1123	
○	91712	5	92653	−941	
▽	91955	12	92653	−698	
△	92091	20	92653	−562	
▷	92779	8	92784	−5	1[+Met] 1[RK774r-A] 1[RK774r-B] 1[RK774r-C]
□	92925	15	92784	141	

[1]Intensity values should not be assumed as quantitative

hetBGL03-18-15_reloop

Laboratory: Wysocki-ASN
Client: Baker-RyanK
Shipment Date: 2021_03_03
Data Recorded: Sat, 06 Mar 2021, 04:14
Instrument: Q Exactive UHMR Orbitrap
Study: OBE 200 mM AmAc, RO15
Sample Conditions: 25mM Tris pH8, 300mM NaCl
Concentration (ug/uL): 1.0
Injection (uL): 0.5
Comment: ThermoOBE RO15 2.1x50mm 3um 80A
Date Analyzed: Mon, 15 Mar 2021, 15:32 Eastern Daylight Time
File Name: 20210304_RyanK_RK775_6k_HCD120

Quality Check:
Average Peaks Score: -0.31

Expected Oligomers:
Passed:
Mixed:
Failed:

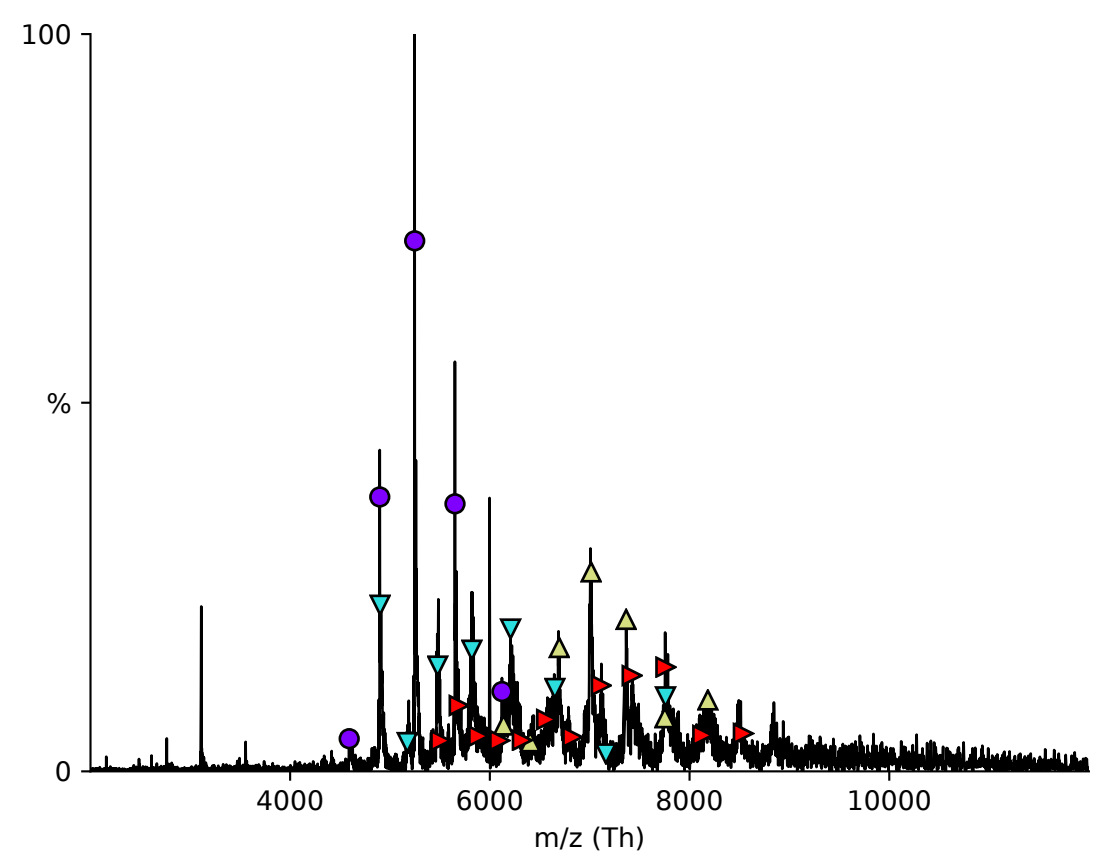

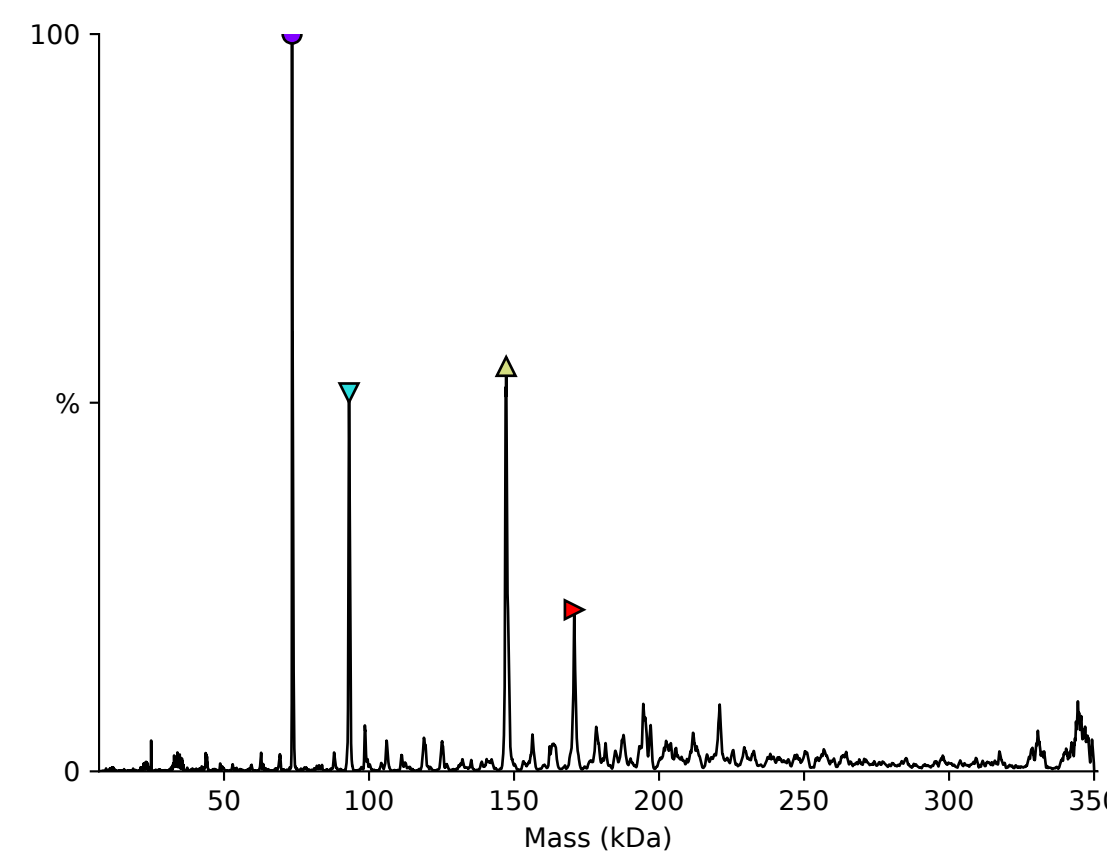

Symbol	Mass (Da)	Intensity[1]	Expected	Difference (Da)	Name/Species/Oligomer
○	73450	100	73463	−13	1[RK775_A] 1[RK775_C]
▽	93100	51	93065	35	1[RK775_A] 1[RK775_B] 1[RK775_C]
△	147280	55	146927	353	2[RK775_A] 2[RK775_C]
▷	170750	22	170641	109	1[RK775_A] 3[RK775_C]

[1] Intensity values should not be assumed as quantitative

hetBGL08-09-19_reloop

Laboratory: Wysocki-AN
Client: Baker-Ryan
Shipment Date: 4/28/2021
Data Recorded: Fri, 30 Apr 2021, 23:22
Instrument: Q Exactive UHMR Orbitrap
Study: OBE 200 mM AmAc, RO22
Sample Conditions: 25mM Tris pH8, 300mM NaCl, 10% glycerol, 300mM Imidazole, 10mM EDTA
Concentration (ug/uL): 1.0
Injection (uL): 0.6
Comment:
Date Analyzed: Tue, 11 May 2021, 12:16 Eastern Daylight Time
File Name: 20210429_Ryan_RK776RAW_6k_HCD120

Quality Check:
Average Peaks Score: 0.93

Expected Oligomers:
Passed:
Mixed:
Failed:

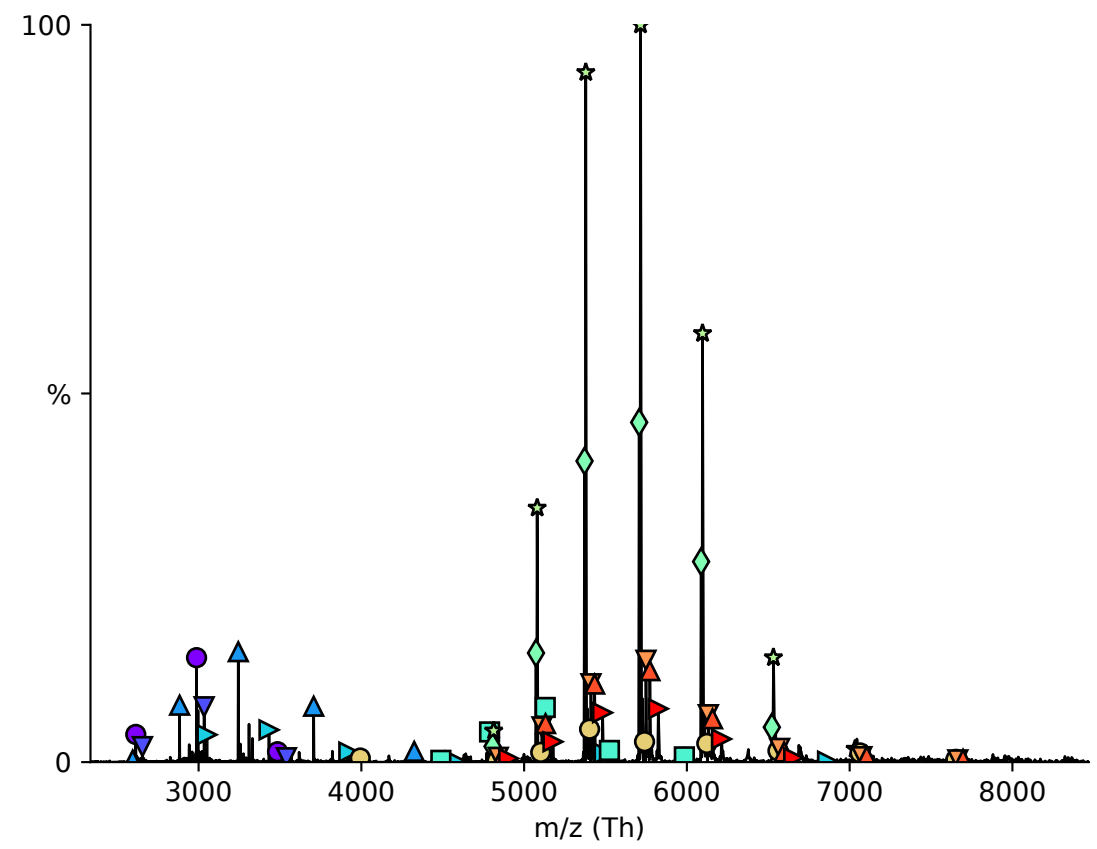

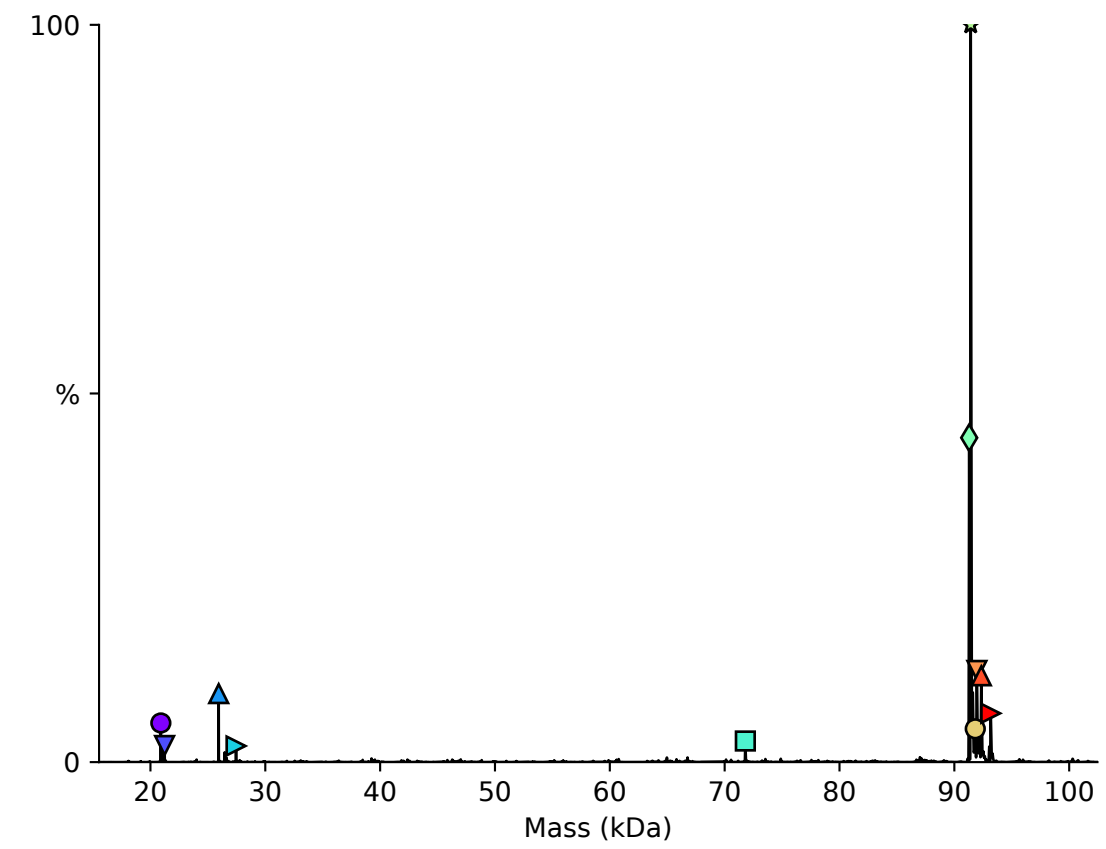

Symbol	Mass (Da)	Intensity[1]	Expected	Difference (Da)	Name/Species/Oligomer
○	20905	5	19598	1307	
▽	21231	2	19598	1633	
△	25942	9	25942	0	1[RK776r-A]
▷	27452	2	26073	1379	
□	71801	3	73430	−1629	
◊	91294	44	92897	−1603	
☆	91426	100	92897	−1471	
○	91822	5	92897	−1075	
▽	91962	12	92897	−935	
△	92348	12	92897	−549	
▷	93171	7	93028	143	

It is interesting that the cleaved version seems to have more lower oligomers.

[1]Intensity values should not be assumed as quantitative

RTR03_C4

Laboratory: Wysocki-AN
Client: Baker-Ryan
Shipment Date: 2021_03_17
Data Recorded: Thu, 18 Mar 2021, 22:14
Instrument: Q Exactive UHMR Orbitrap
Study: OBE 200 mM AmAc, RO24
Sample Conditions: 25mM Tris pH8, 300mM NaCl
Concentration (ug/uL): 1.0
Injection (uL): 0.5
Comment: ThermoOBE RO24 2.1x50mm 3um 80A
Date Analyzed: Tue, 23 Mar 2021, 10:40 Eastern Daylight Time
File Name: 20210318_Ryan_RK752_6k_IST030_HCD120

Quality Check:
Average Peaks Score: 0.8

Expected Oligomers:
Passed:
Mixed:
Failed:

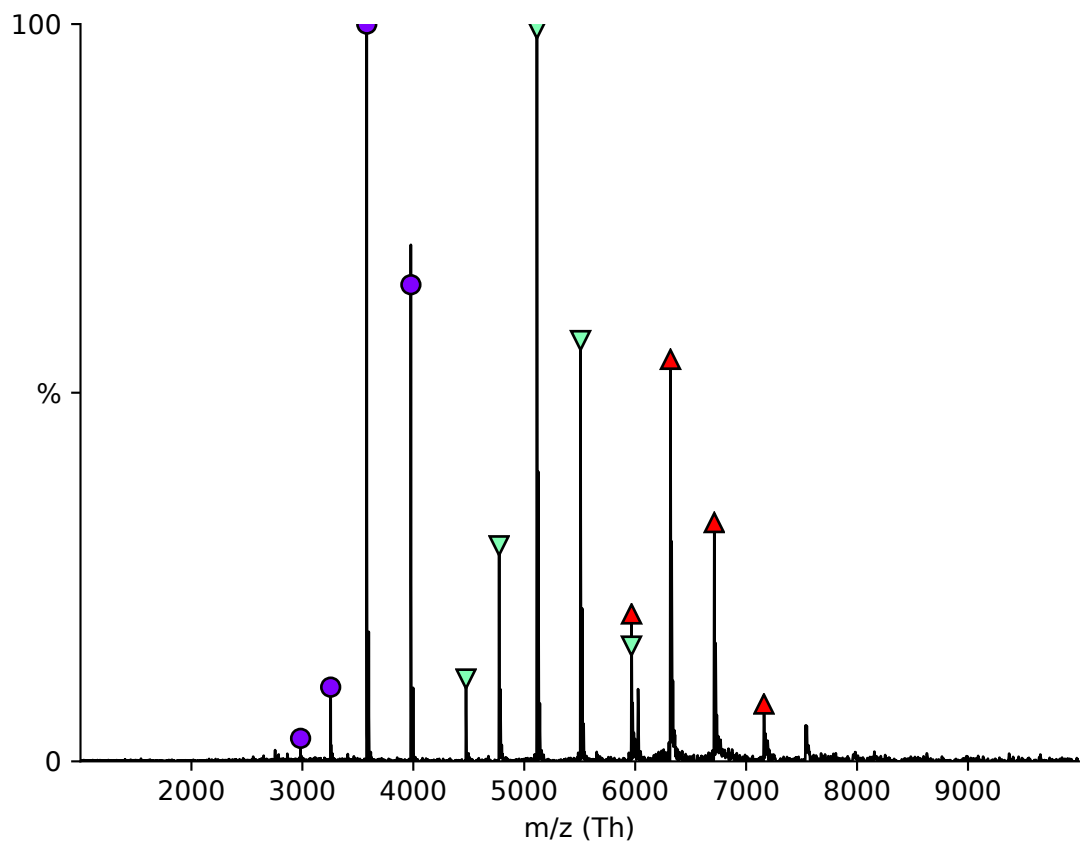

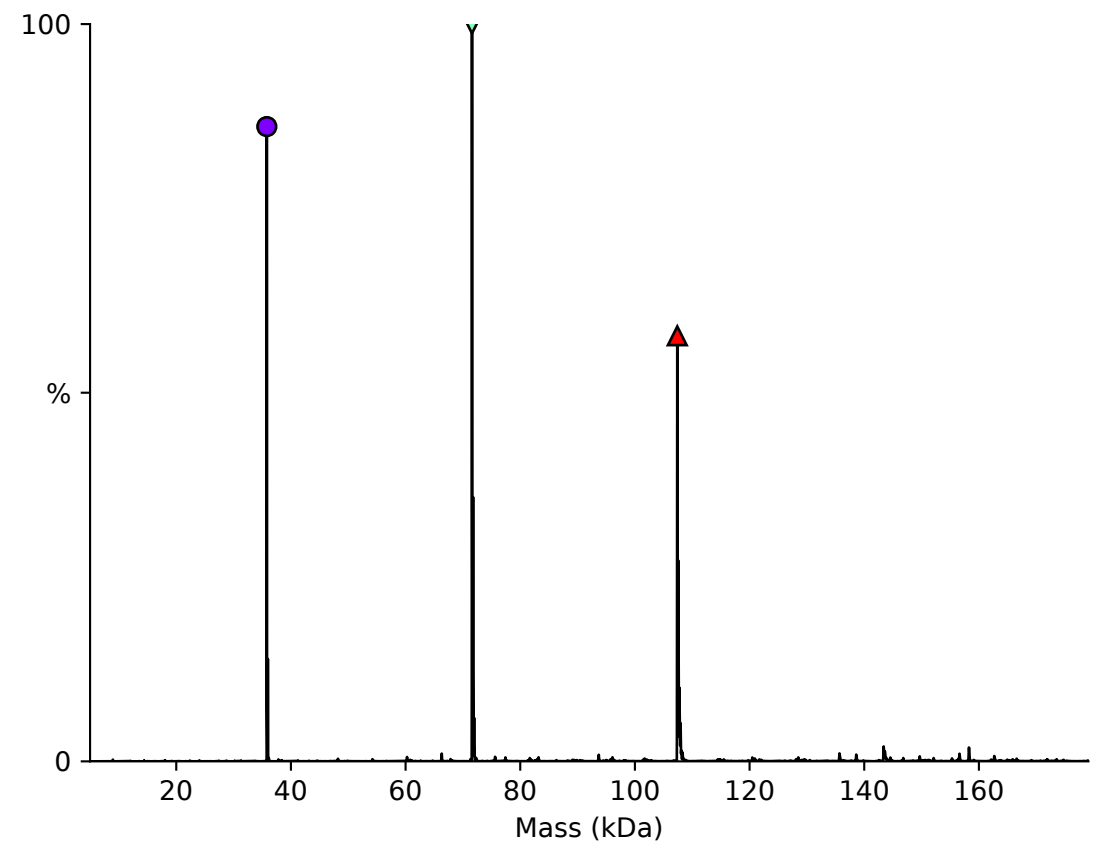

Symbol	Mass (Da)	Intensity[1]	Expected	Difference (Da)	Name/Species/Oligomer
○	35795	86	35797	−2	1[RK752]
▽	71584	100	71594	−10	2[RK752]
△	107392	58	107390	2	3[RK752]

[1] Intensity values should not be assumed as quantitative

RTR04_C4

Laboratory: Wysocki-AN
Client: Baker-Ryan
Shipment Date: 2021_03_17
Data Recorded: Thu, 18 Mar 2021, 22:25
Instrument: Q Exactive UHMR Orbitrap
Study: OBE 200 mM AmAc, RO24
Sample Conditions: 25mM Tris pH8, 300mM NaCl
Concentration (ug/uL): 1.0
Injection (uL): 0.5
Comment: ThermoOBE RO24 2.1x50mm 3um 80A
Date Analyzed: Tue, 23 Mar 2021, 10:57 Eastern Daylight Time
File Name: 20210318_Ryan_RK753_6k_IST030_HCD120

Quality Check:
Average Peaks Score: 0.77

Expected Oligomers:
Passed:
Mixed:
Failed:

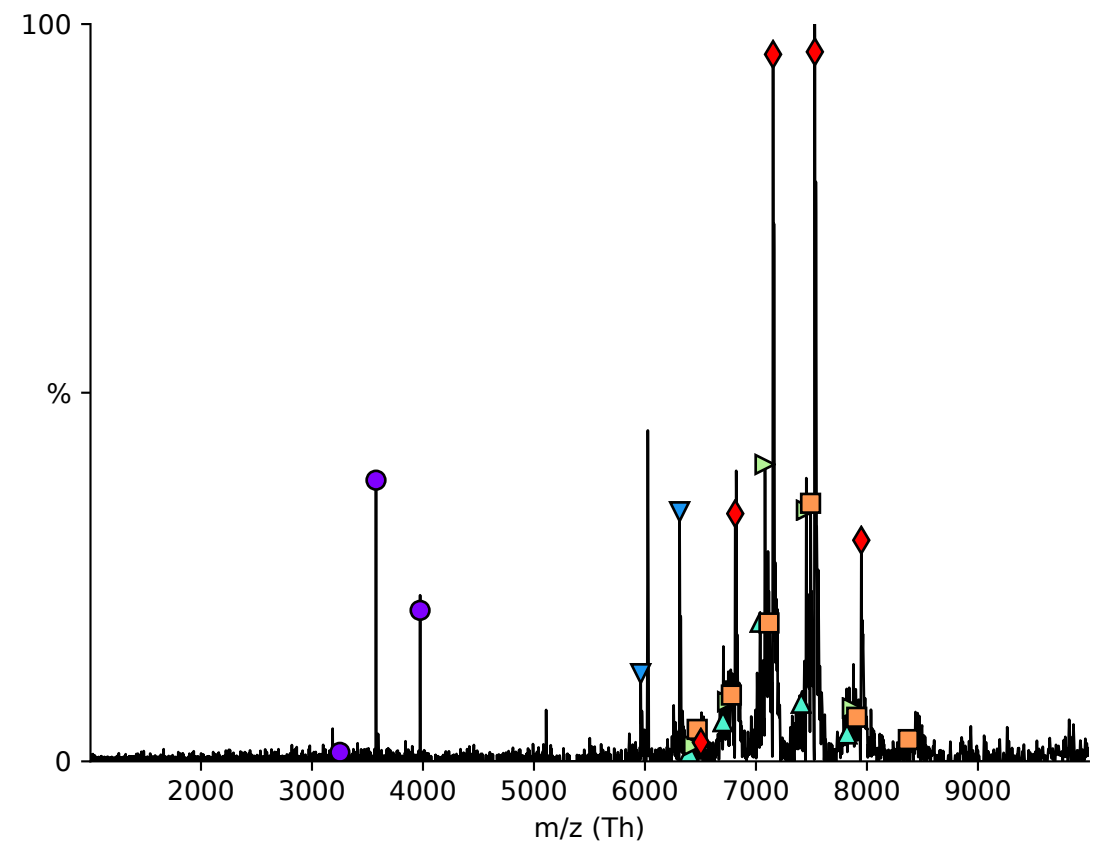

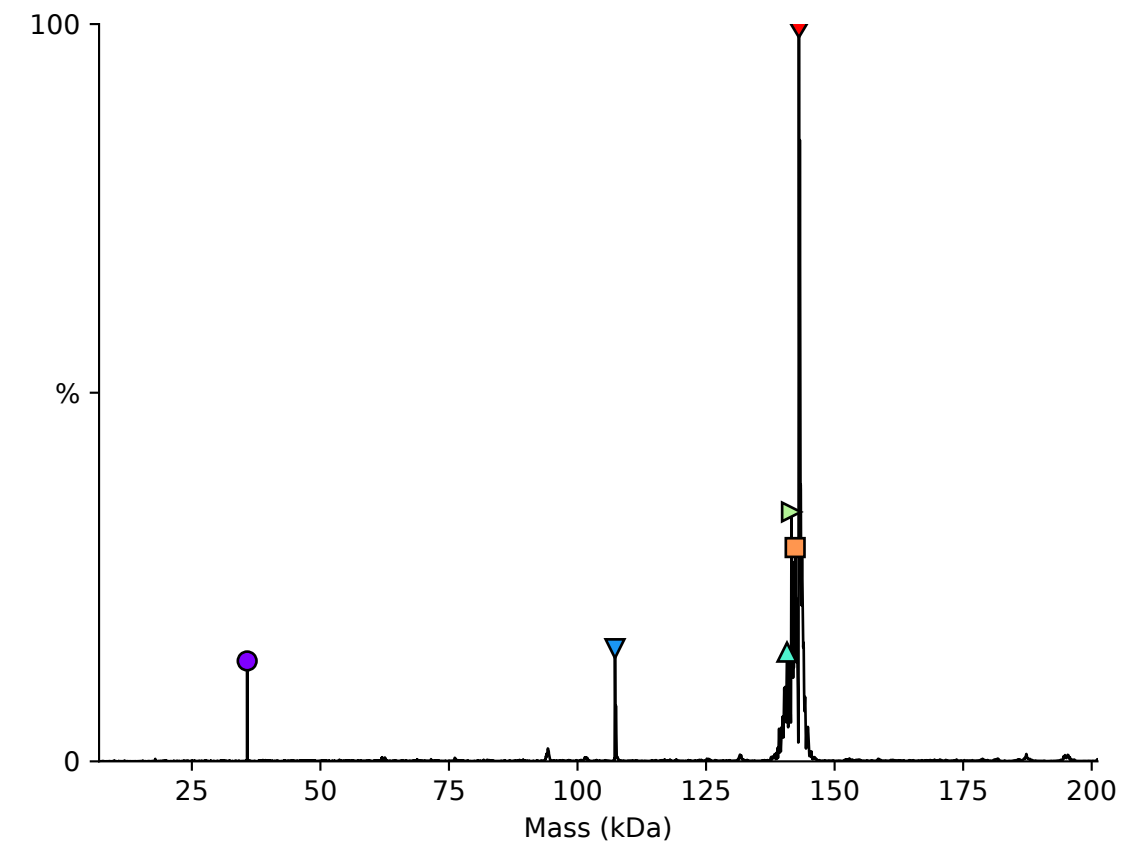

Symbol	Mass (Da)	Intensity[1]	Expected	Difference (Da)	Name/Species/Oligomer
○	35756	14	35760	−4	1[RK753]
▽	107289	15	107279	10	3[RK753]
△	140740	15	143039	−2299	
▷	141611	34	143039	−1428	
□	142327	29	143039	−712	
◊	143061	100	143039	22	4[RK753]

[1] Intensity values should not be assumed as quantitative

RTR05_C4

Laboratory: Wysocki-AN
Client: Baker-Ryan
Shipment Date: 2021_03_17
Data Recorded: Thu, 18 Mar 2021, 22:36
Instrument: Q Exactive UHMR Orbitrap
Study: OBE 200 mM AmAc, RO24
Sample Conditions: 25mM Tris pH8, 300mM NaCl
Concentration (ug/uL): 1.0
Injection (uL): 0.5
Comment: ThermoOBE RO24 2.1x50mm 3um 80A
Date Analyzed: Tue, 23 Mar 2021, 11:07 Eastern Daylight Time
File Name: 20210318_Ryan_RK754_6k_IST030_HCD120

Quality Check:
Average Peaks Score: 0.57

Expected Oligomers:
Passed:
Mixed:
Failed:

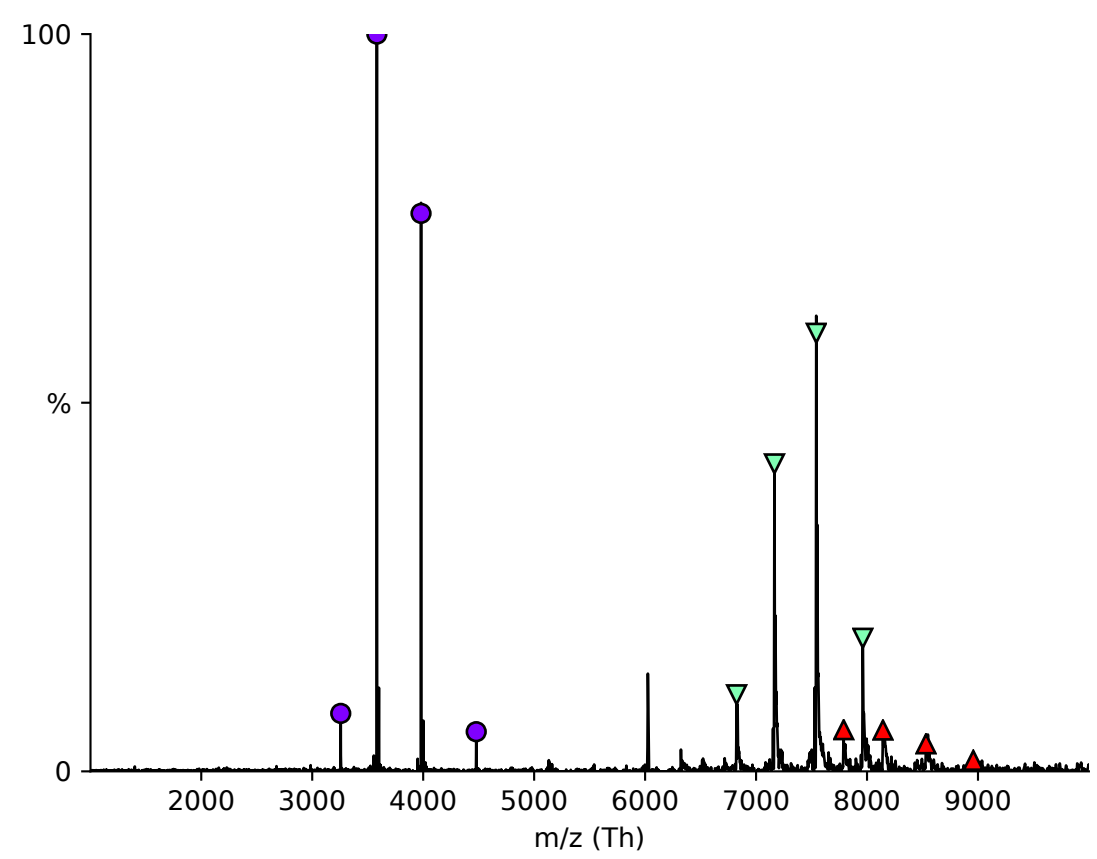

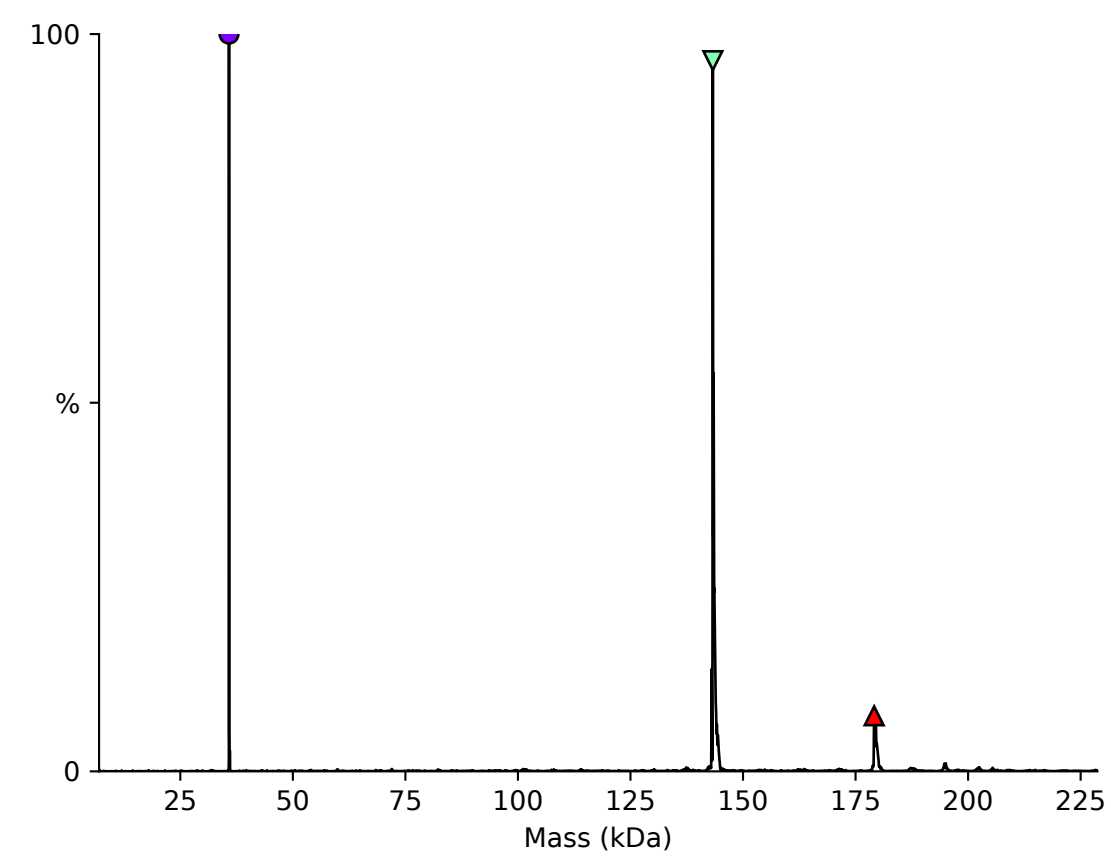

Symbol	Mass (Da)	Intensity[1]	Expected	Difference (Da)	Name/Species/Oligomer
○	35816	100	35820	−4	1[RK754]
▽	143296	96	143279	17	4[RK754]
△	179134	8	179098	36	5[RK754]

[1]Intensity values should not be assumed as quantitative

RTR06_C4

Laboratory: Wysocki-AN
Client: Baker-Ryan
Shipment Date: 2021_03_17
Data Recorded: Thu, 18 Mar 2021, 22:47
Instrument: Q Exactive UHMR Orbitrap
Study: OBE 200 mM AmAc, RO24
Sample Conditions: 25mM Tris pH8, 300mM NaCl
Concentration (ug/uL): 1.0
Injection (uL): 0.5
Comment: ThermoOBE RO24 2.1x50mm 3um 80A
Date Analyzed: Tue, 23 Mar 2021, 11:10 Eastern Daylight Time
File Name: 20210318_Ryan_RK755_6k_IST030_HCD120

Quality Check:
Average Peaks Score: 0.72

Expected Oligomers:
Passed:
Mixed:
Failed:

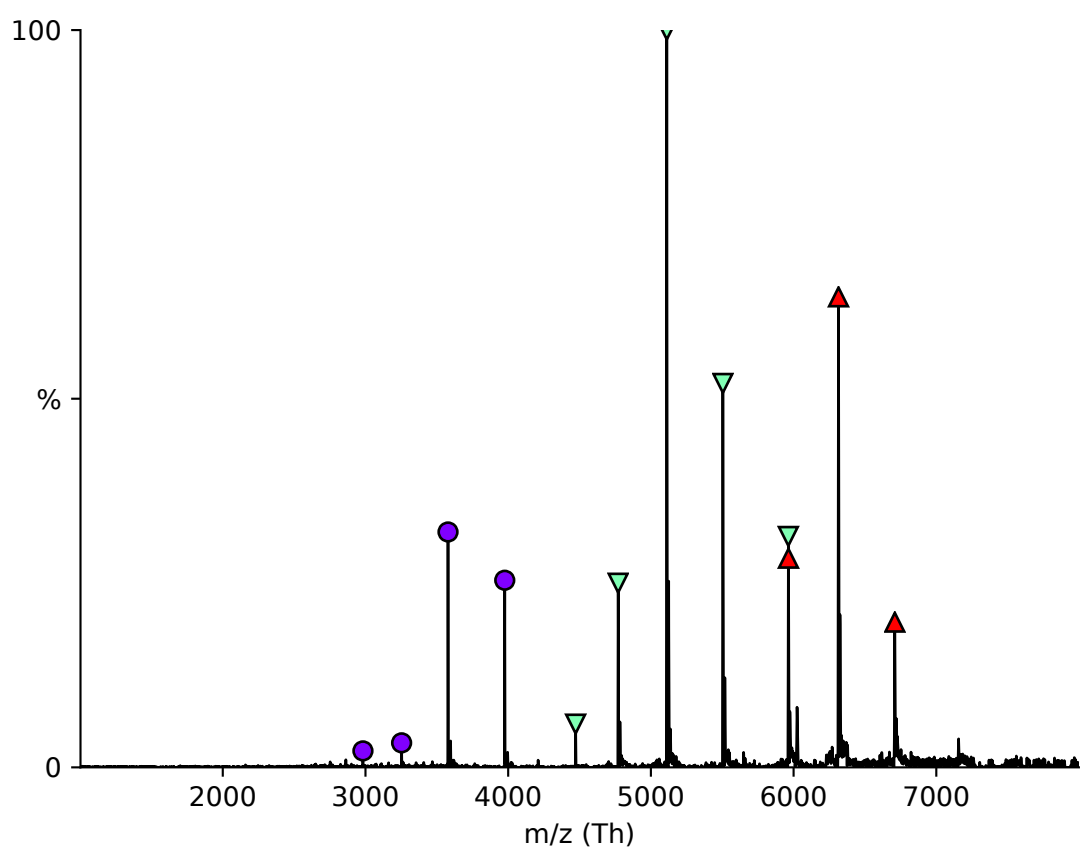

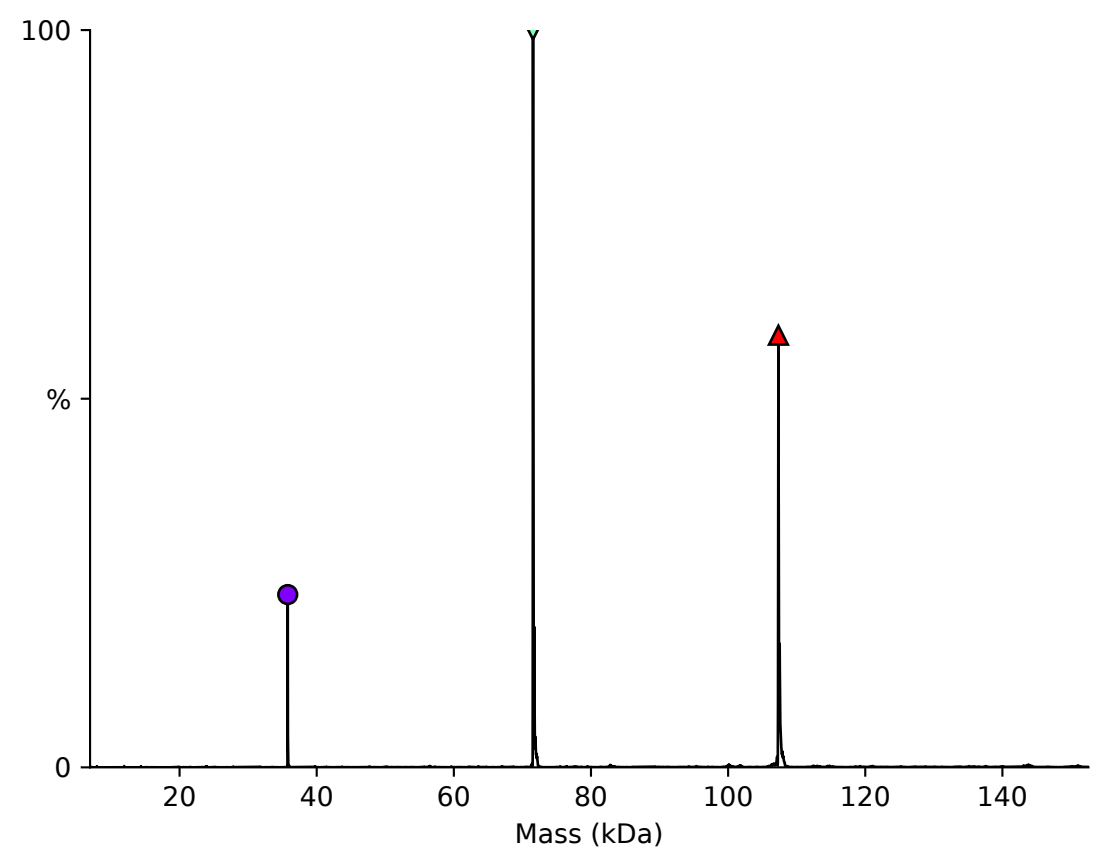

Symbol	Mass (Da)	Intensity[1]	Expected	Difference (Da)	Name/Species/Oligomer
○	35774	23	35777	−3	1[RK755]
▽	71546	100	71553	−7	2[RK755]
△	107334	59	107330	4	3[RK755]

[1]Intensity values should not be assumed as quantitative

RTR07_C4

Laboratory: Wysocki-AN
Client: Baker-Ryan
Shipment Date: 2021_03_17
Data Recorded: Thu, 18 Mar 2021, 22:58
Instrument: Q Exactive UHMR Orbitrap
Study: OBE 200 mM AmAc, RO24
Sample Conditions: 25mM Tris pH8, 300mM NaCl
Concentration (ug/uL): 1.0
Injection (uL): 0.5
Comment: ThermoOBE RO24 2.1x50mm 3um 80A
Date Analyzed: Tue, 23 Mar 2021, 11:15 Eastern Daylight Time
File Name: 20210318_Ryan_RK756_6k_IST030_HCD120

Quality Check:
Average Peaks Score: 0.51

Expected Oligomers:
Passed:
Mixed:
Failed:

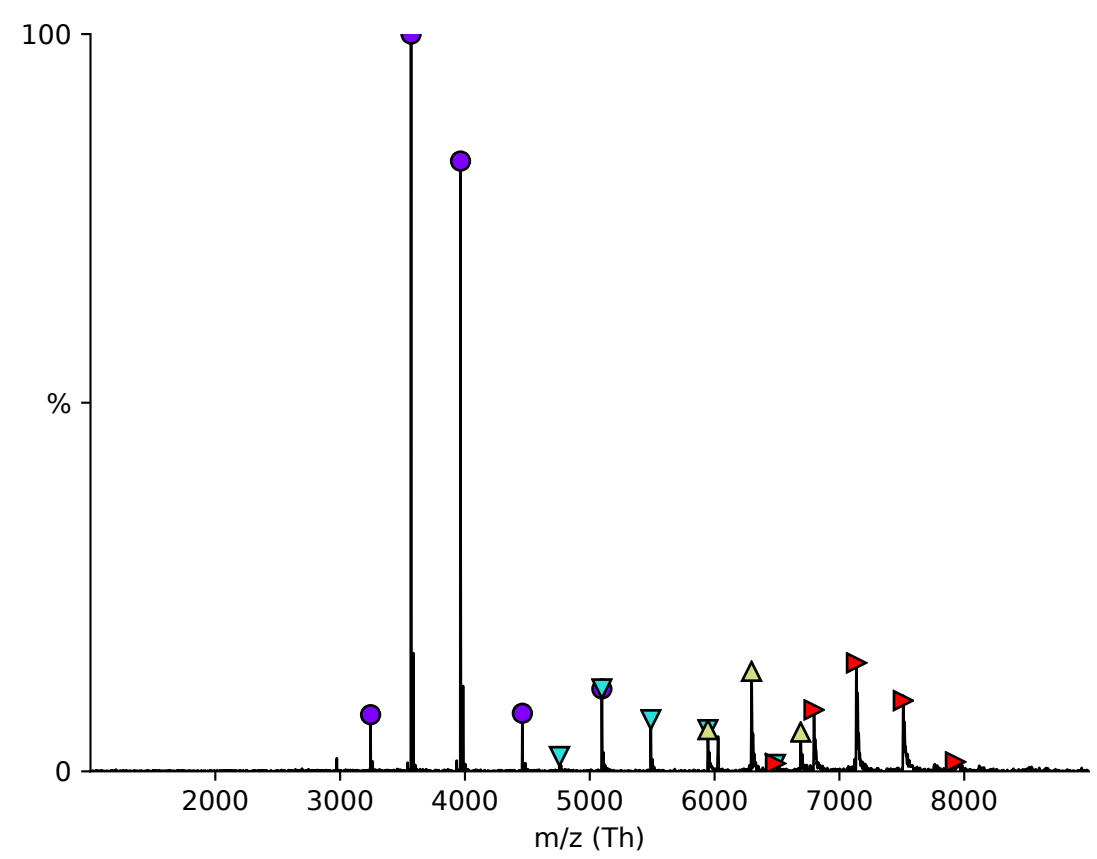

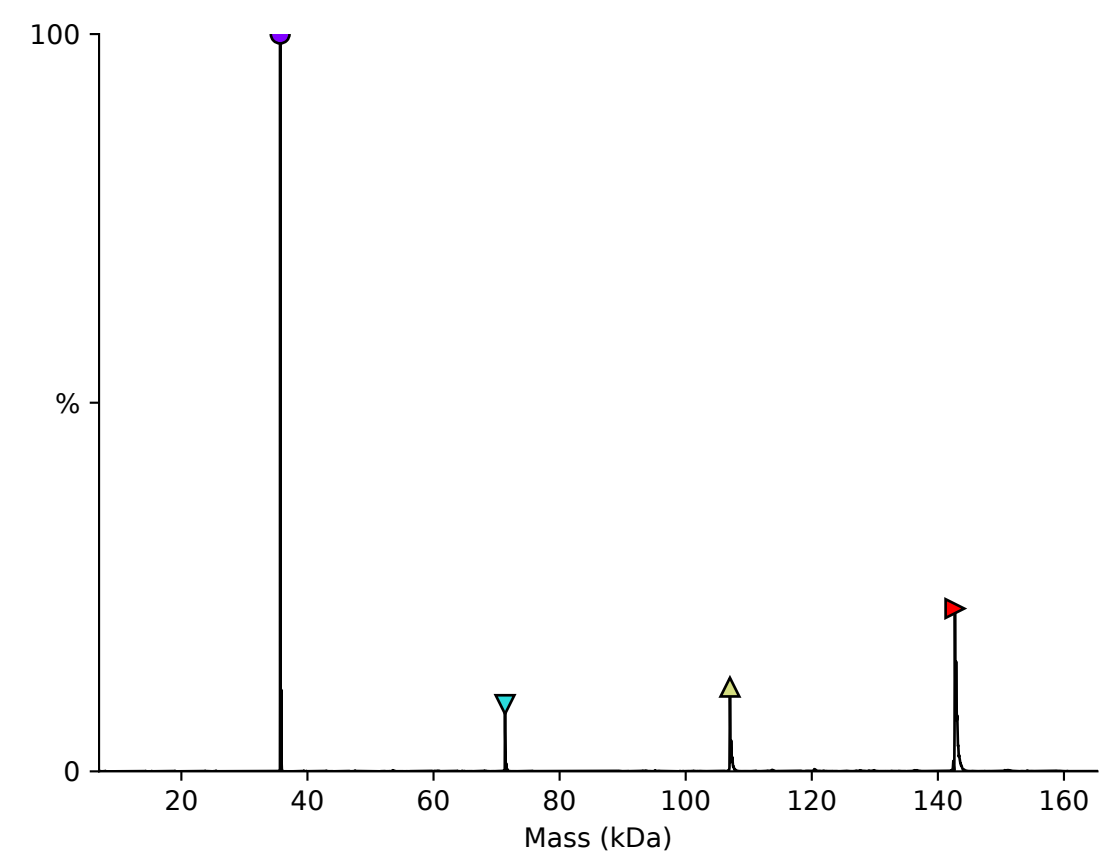

Symbol	Mass (Da)	Intensity[1]	Expected	Difference (Da)	Name/Species/Oligomer
○	35671	100	35674	−3	1[RK756]
▽	71343	9	71347	−4	2[RK756]
△	107017	11	107021	−4	3[RK756]
▷	142707	22	142694	13	4[RK756]

[1]Intensity values should not be assumed as quantitative

RTR08_C4

Laboratory: Wysocki-AN
Client: Baker-Ryan
Shipment Date: 2021_03_17
Data Recorded: Thu, 18 Mar 2021, 23:09
Instrument: Q Exactive UHMR Orbitrap
Study: OBE 200 mM AmAc, RO24
Sample Conditions: 25mM Tris pH8, 300mM NaCl
Concentration (ug/uL): 1.0
Injection (uL): 0.5
Comment: ThermoOBE RO24 2.1x50mm 3um 80A
Date Analyzed: Tue, 23 Mar 2021, 11:16 Eastern Daylight Time
File Name: 20210318_Ryan_RK757_6k_IST030_HCD120

Quality Check:
Average Peaks Score: 0.53

Expected Oligomers:
Passed:
Mixed:
Failed:

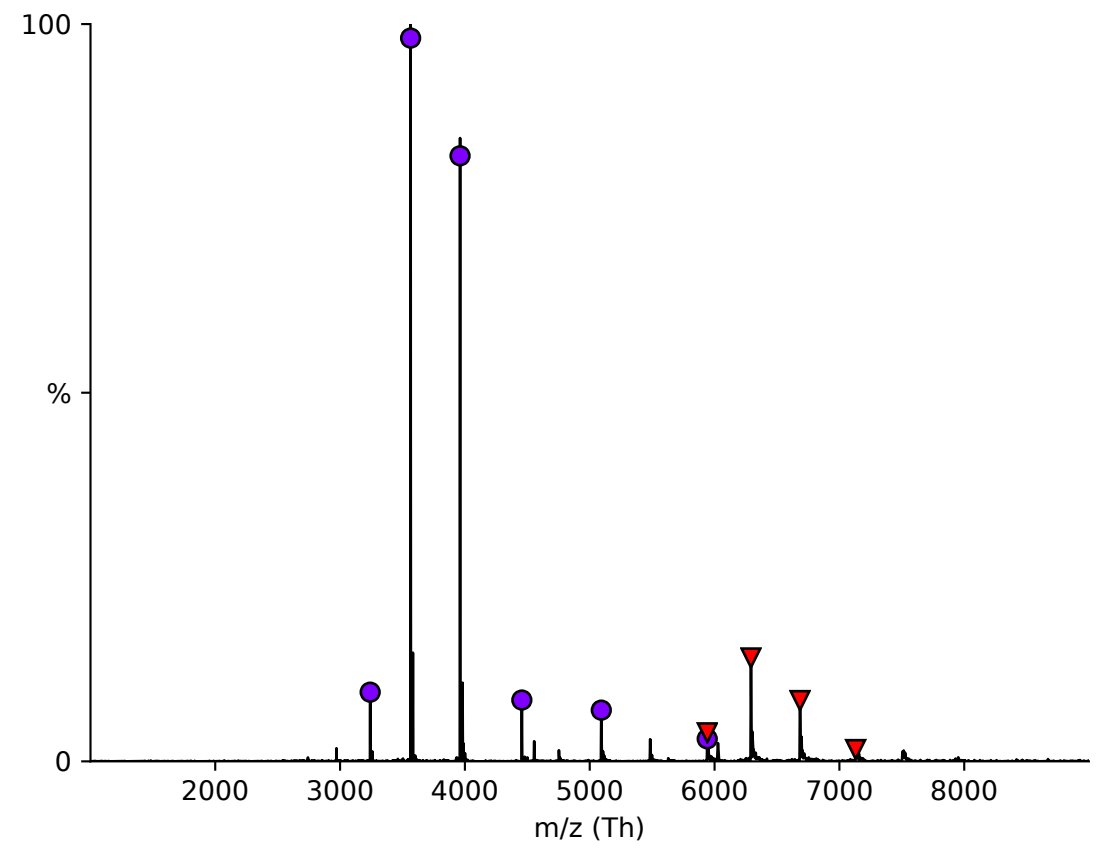

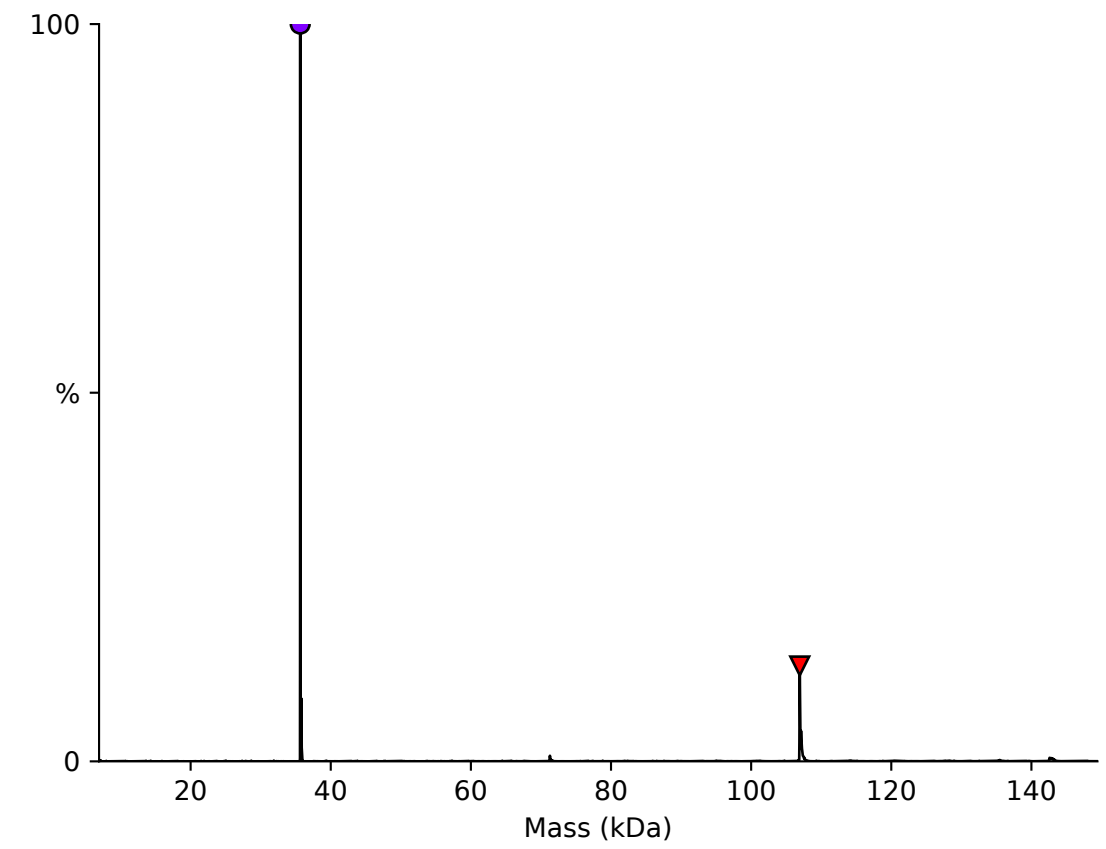

Symbol	Mass (Da)	Intensity[1]	Expected	Difference (Da)	Name/Species/Oligomer
○	35641	100	35644	−3	1[RK757]
▽	106937	13	106931	6	3[RK757]

[1]Intensity values should not be assumed as quantitative

RTR11_C4

Laboratory: Wysocki-AN
Client: Baker-Ryan
Shipment Date: 2021_03_17
Data Recorded: Thu, 18 Mar 2021, 23:20
Instrument: Q Exactive UHMR Orbitrap
Study: OBE 200 mM AmAc, RO24
Sample Conditions: 25mM Tris pH8, 300mM NaCl
Concentration (ug/uL): 1.0
Injection (uL): 0.5
Comment: ThermoOBE RO24 2.1x50mm 3um 80A
Date Analyzed: Tue, 23 Mar 2021, 11:19 Eastern Daylight Time
File Name: 20210318_Ryan_RK760_6k_IST030_HCD120

Quality Check:
Average Peaks Score: 0.52

Expected Oligomers:
Passed:
Mixed:
Failed:

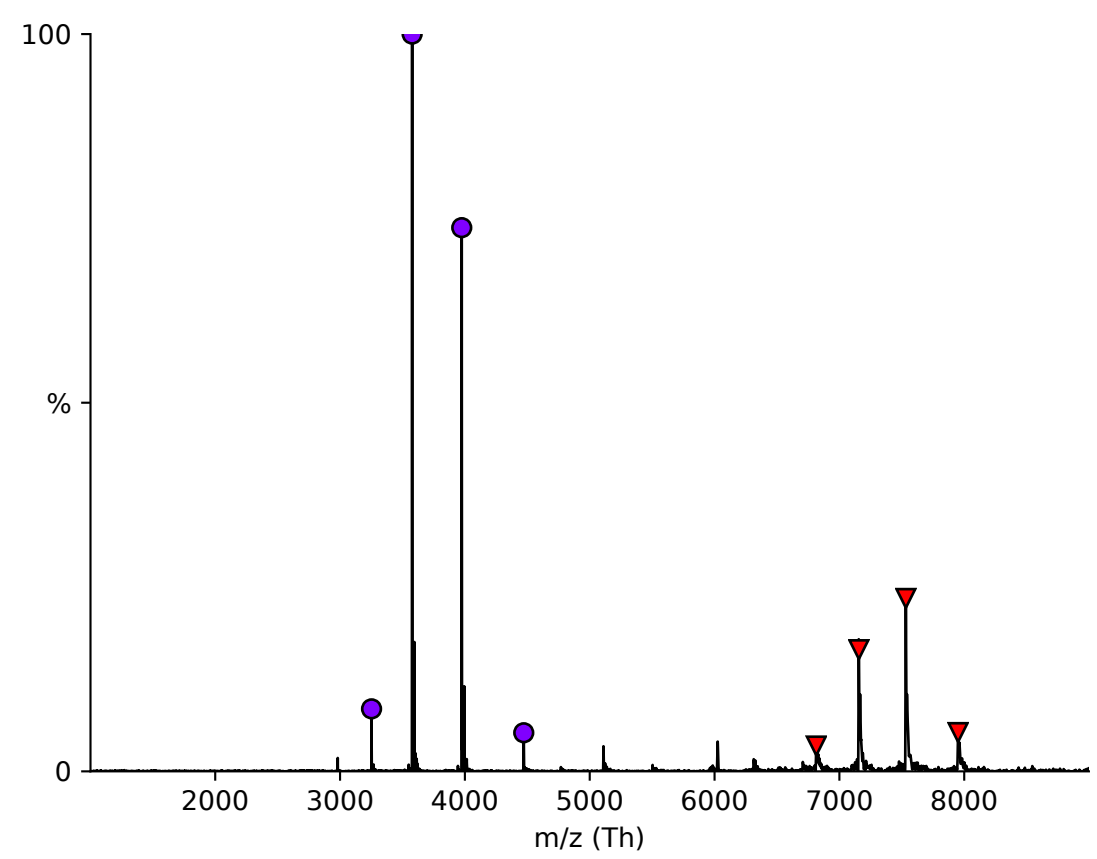

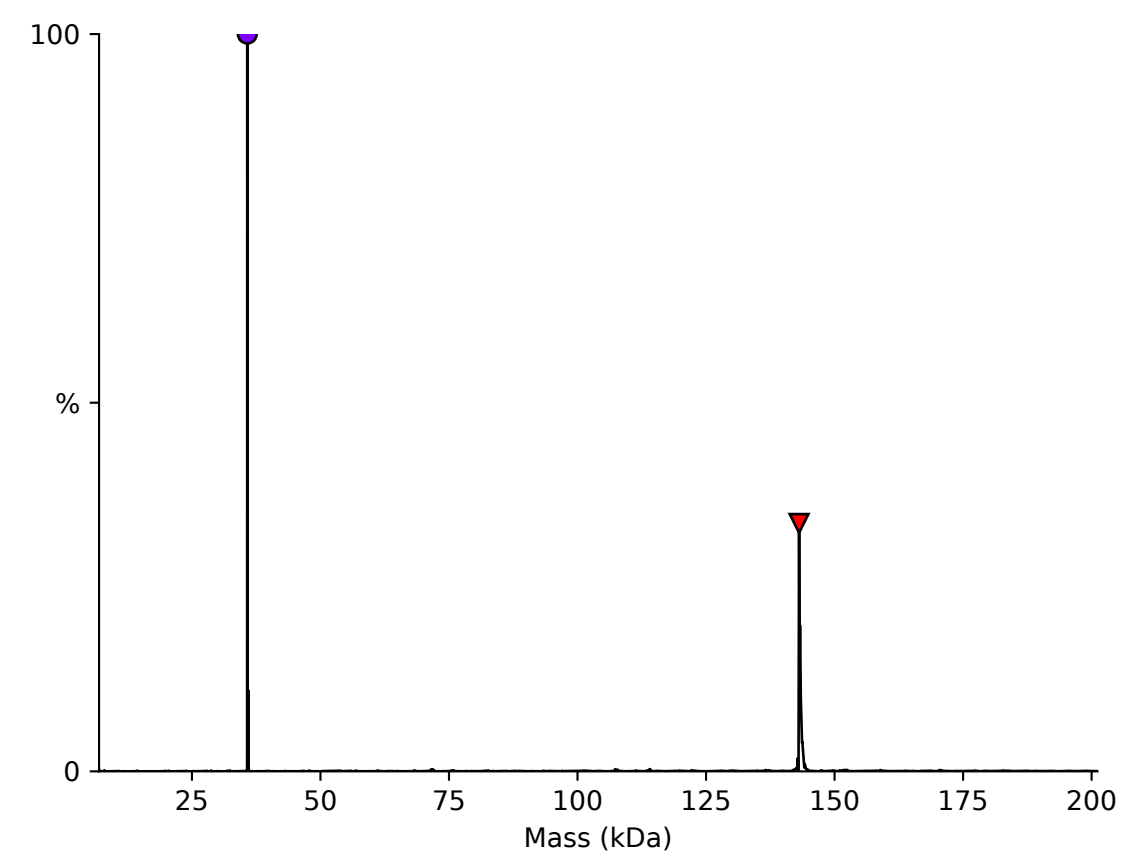

Symbol	Mass (Da)	Intensity[1]	Expected	Difference (Da)	Name/Species/Oligomer
○	35764	100	35766	−2	1[RK760]
▽	143098	34	143063	35	4[RK760]

[1]Intensity values should not be assumed as quantitative

RTR12_C4

Laboratory: Wysocki-AN
Client: Baker-Ryan
Shipment Date: 2021_03_17
Data Recorded: Thu, 18 Mar 2021, 23:31
Instrument: Q Exactive UHMR Orbitrap
Study: OBE 200 mM AmAc, RO24
Sample Conditions: 25mM Tris pH8, 300mM NaCl
Concentration (ug/uL): 1.0
Injection (uL): 0.5
Comment: ThermoOBE RO24 2.1x50mm 3um 80A
Date Analyzed: Tue, 23 Mar 2021, 11:38 Eastern Daylight Time
File Name: 20210318_Ryan_RK761_6k_IST030_HCD120

Quality Check:
Average Peaks Score: 0.68

Expected Oligomers:
Passed:
Mixed:
Failed:

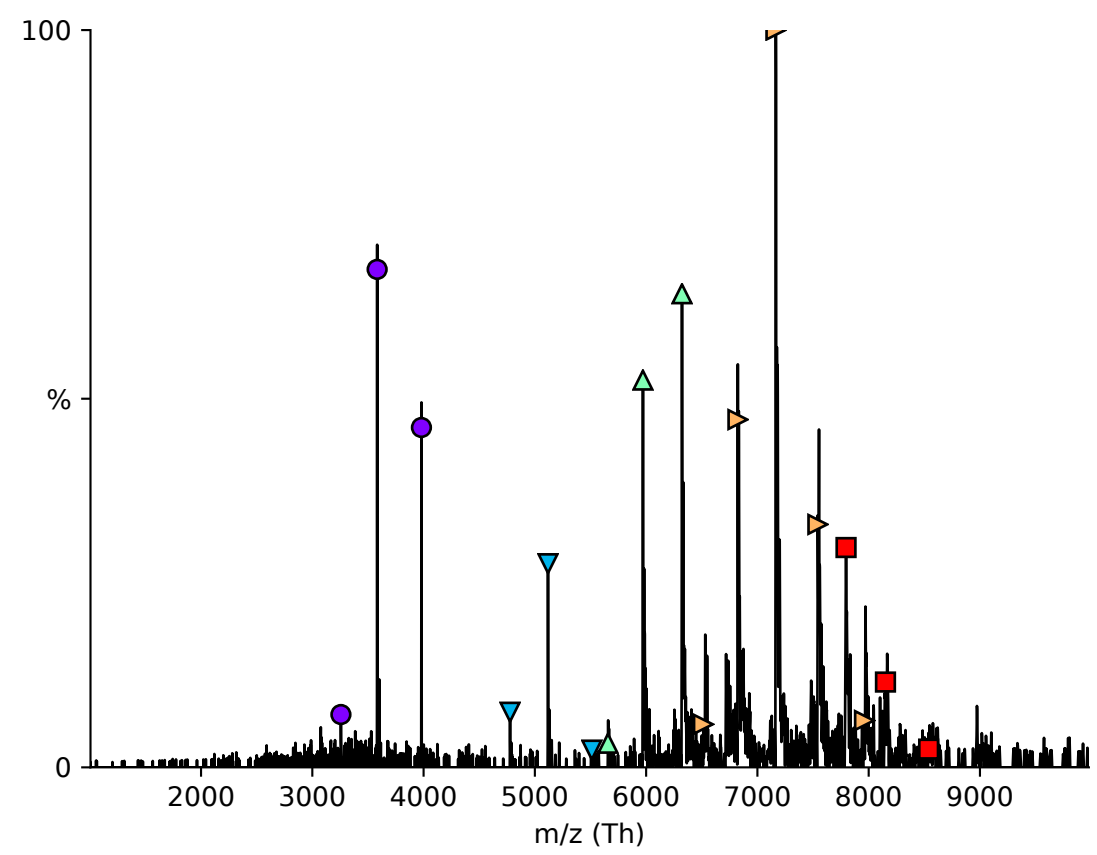

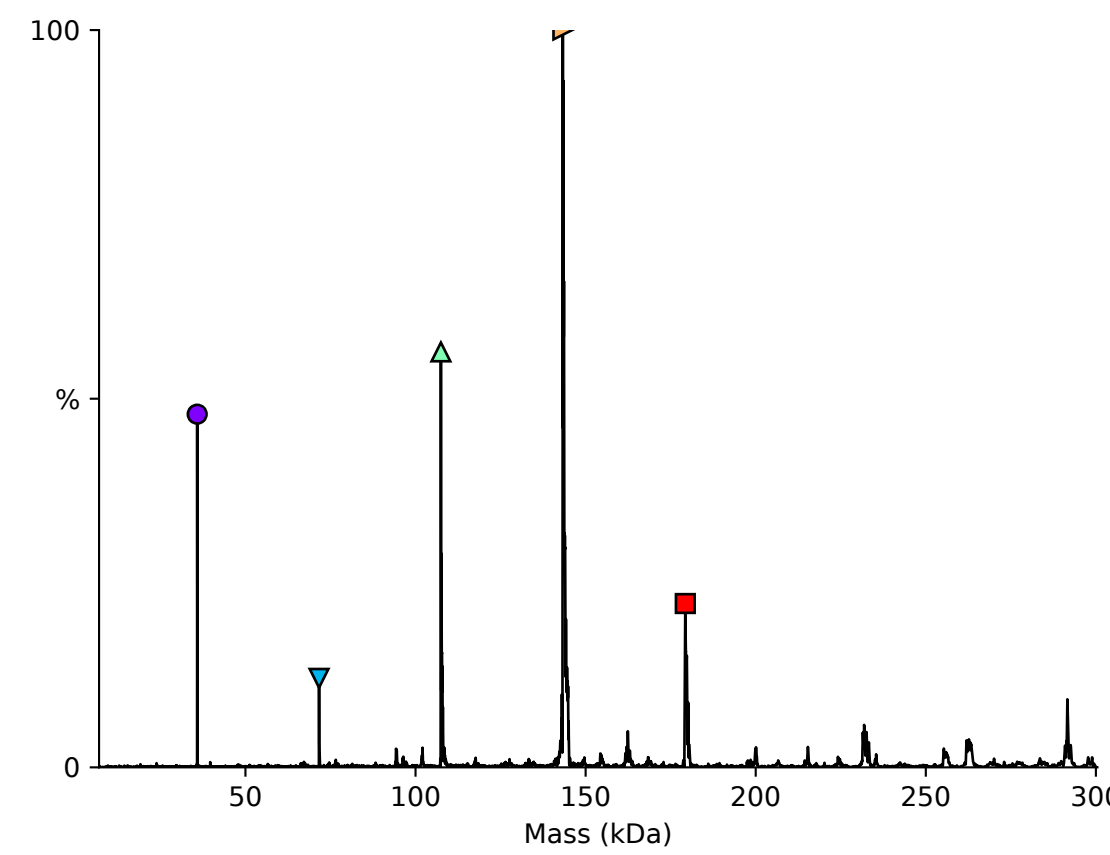

Symbol	Mass (Da)	Intensity[1]	Expected	Difference (Da)	Name/Species/Oligomer
○	35816	48	35820	−4	1[RK761]
▽	71640	12	71640	0	2[RK761]
△	107461	56	107460	1	3[RK761]
▷	143302	100	143280	22	4[RK761]
□	179298	22	179100	198	

[1]Intensity values should not be assumed as quantitative

RTR13_C4

Laboratory: Wysocki-AN
Client: Baker-Ryan
Shipment Date: 2021_03_17
Data Recorded: Thu, 18 Mar 2021, 23:42
Instrument: Q Exactive UHMR Orbitrap
Study: OBE 200 mM AmAc, RO24
Sample Conditions: 25mM Tris pH8, 300mM NaCl
Concentration (ug/uL): 1.0
Injection (uL): 0.5
Comment: ThermoOBE RO24 2.1x50mm 3um 80A
Date Analyzed: Tue, 23 Mar 2021, 11:45 Eastern Daylight Time
File Name: 20210318_Ryan_RK762_6k_IST030_HCD120

Quality Check:
Average Peaks Score: 0.92

Expected Oligomers:
Passed:
Mixed:
Failed:

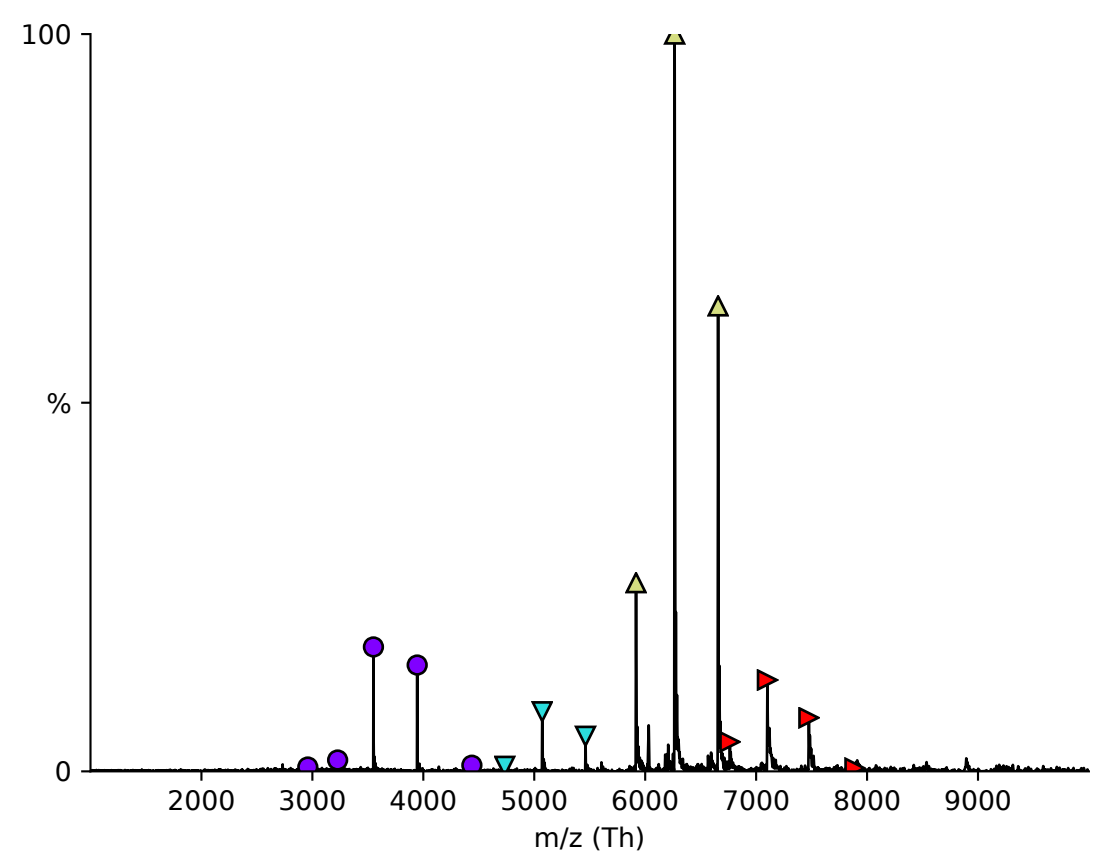

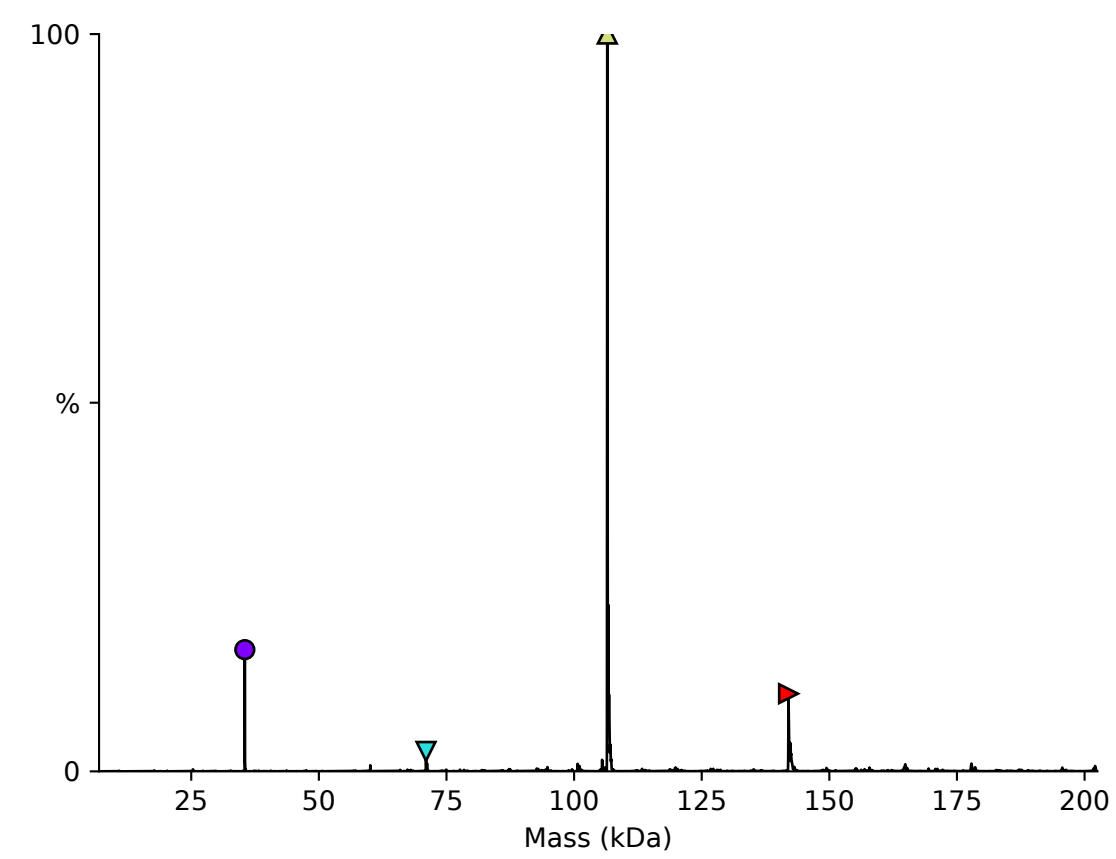

Symbol	Mass (Da)	Intensity[1]	Expected	Difference (Da)	Name/Species/Oligomer
○	35494	17	35497	−3	1[RK762]
▽	70993	3	70995	−2	2[RK762]
△	106499	100	106492	7	3[RK762]
▷	142006	11	141989	17	4[RK762]

[1] Intensity values should not be assumed as quantitative

RTR14_C4

Laboratory: Wysocki-AN
Client: Baker-Ryan
Shipment Date: 2021_03_17
Data Recorded: Thu, 18 Mar 2021, 23:53
Instrument: Q Exactive UHMR Orbitrap
Study: OBE 200 mM AmAc, RO24
Sample Conditions: 25mM Tris pH8, 300mM NaCl
Concentration (ug/uL): 1.0
Injection (uL): 0.5
Comment: ThermoOBE RO24 2.1x50mm 3um 80A
Date Analyzed: Tue, 23 Mar 2021, 11:49 Eastern Daylight Time
File Name: 20210318_Ryan_RK763_6k_IST030_HCD120

Quality Check:
Average Peaks Score: 0.74

Expected Oligomers:
Passed:
Mixed:
Failed:

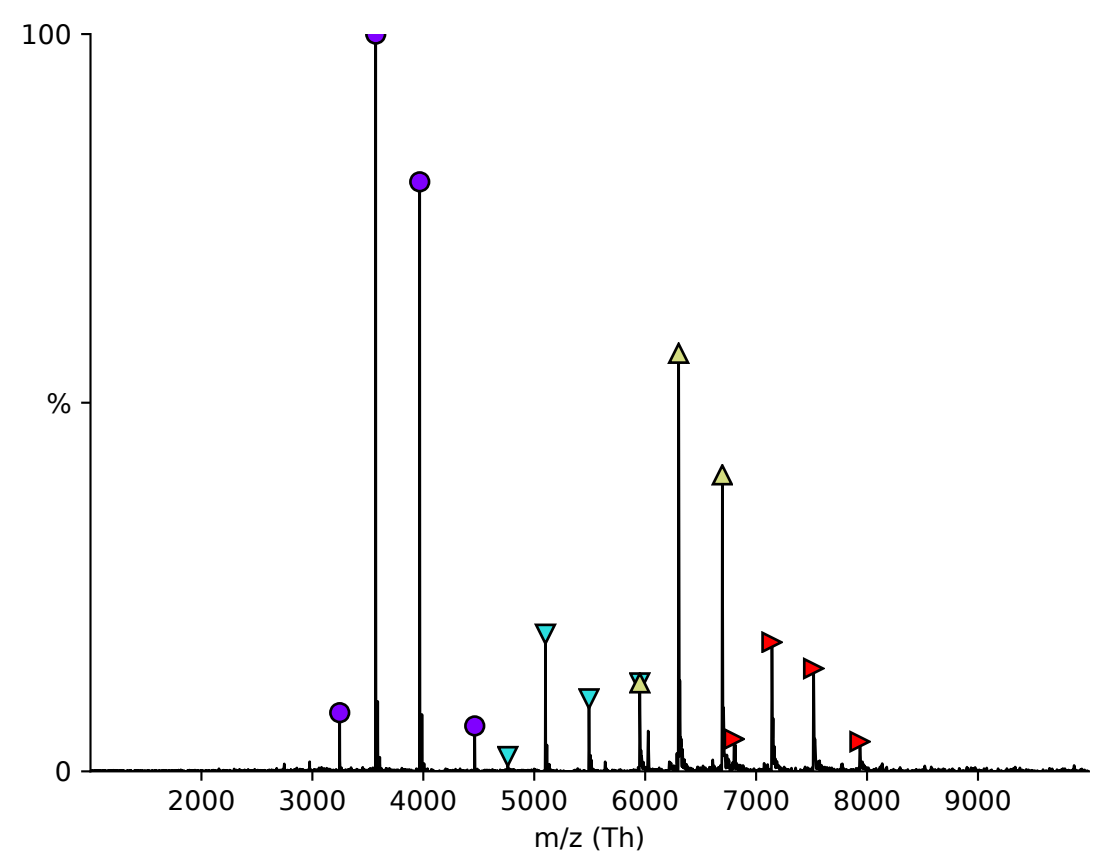

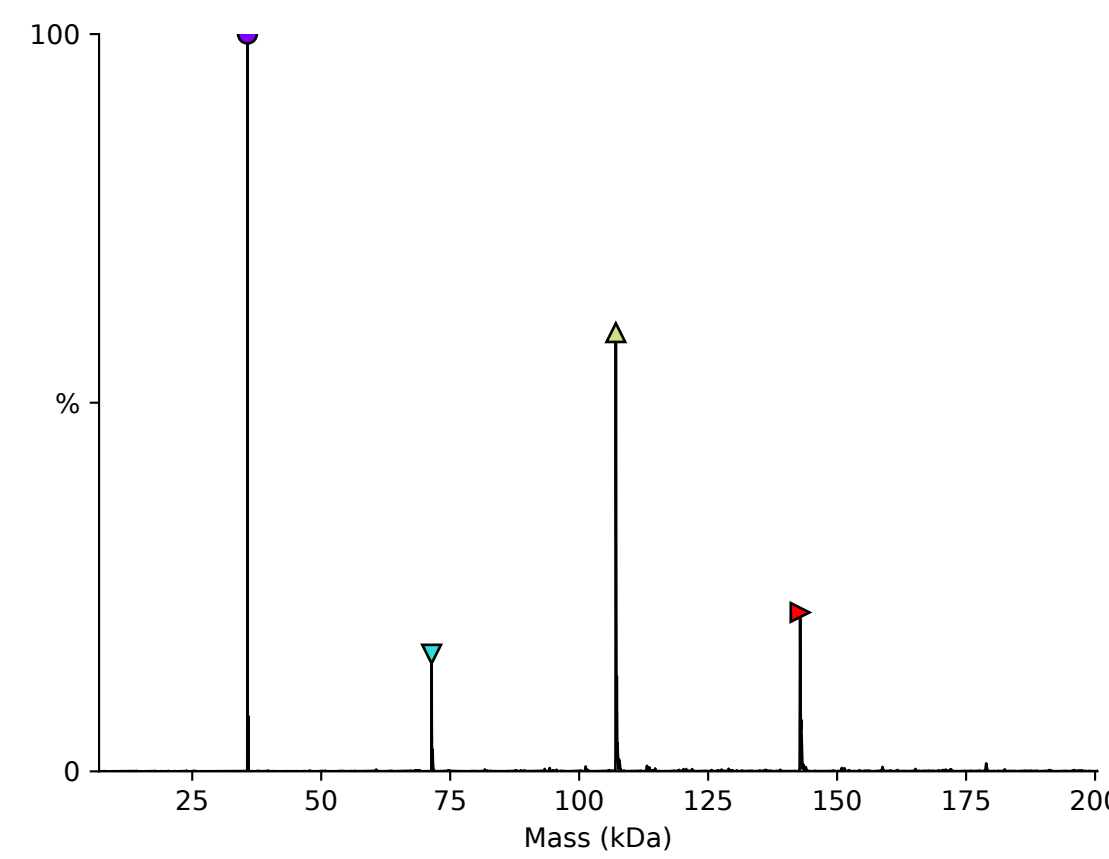

Symbol	Mass (Da)	Intensity[1]	Expected	Difference (Da)	Name/Species/Oligomer
○	35701	100	35704	−3	1[RK763]
▽	71400	16	71407	−7	2[RK763]
△	107110	60	107111	−1	3[RK763]
▷	142827	22	142815	12	4[RK763]

[1]Intensity values should not be assumed as quantitative

RTR15_C4

Laboratory: Wysocki-AN
Client: Baker-Ryan
Shipment Date: 2021_03_17
Data Recorded: Fri, 19 Mar 2021, 00:04
Instrument: Q Exactive UHMR Orbitrap
Study: OBE 200 mM AmAc, RO24
Sample Conditions: 25mM Tris pH8, 300mM NaCl
Concentration (ug/uL): 1.0
Injection (uL): 0.5
Comment: ThermoOBE RO24 2.1x50mm 3um 80A
Date Analyzed: Tue, 23 Mar 2021, 11:50 Eastern Daylight Time
File Name: 20210318_Ryan_RK764_6k_IST030_HCD120

Quality Check:
Average Peaks Score: 0.74

Expected Oligomers:
Passed:
Mixed:
Failed:

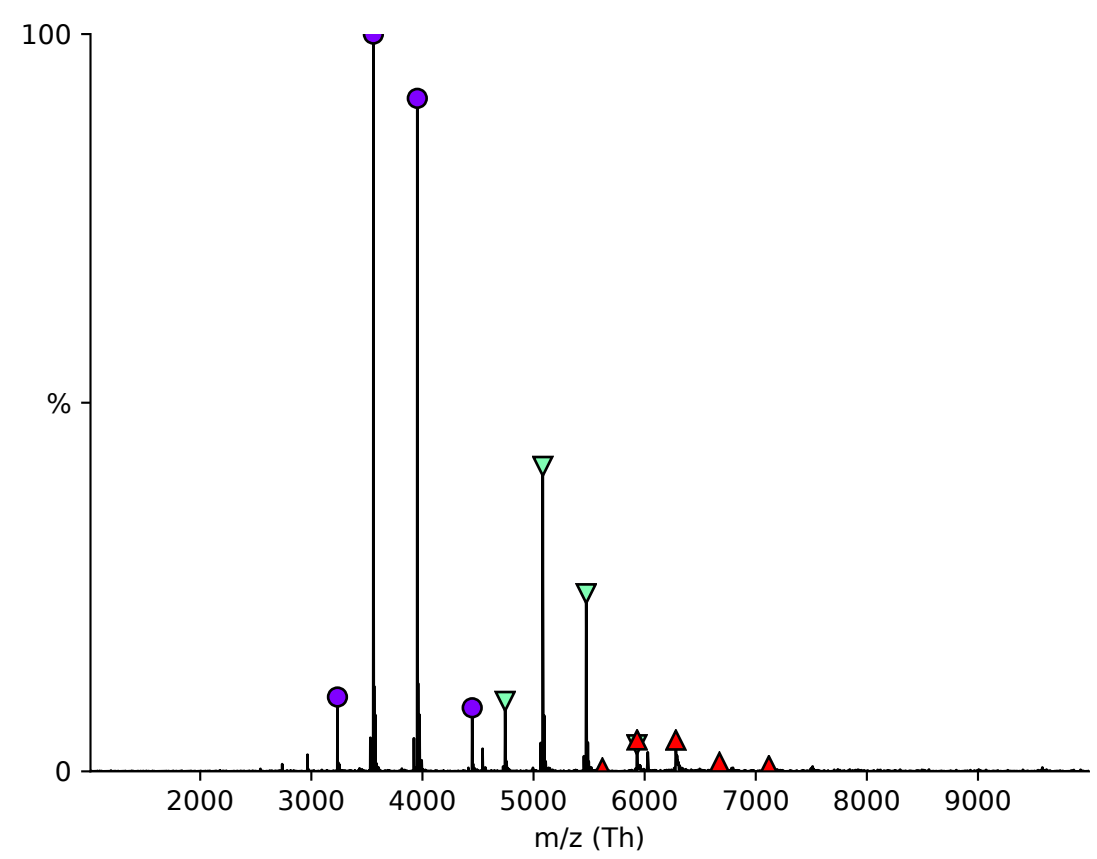

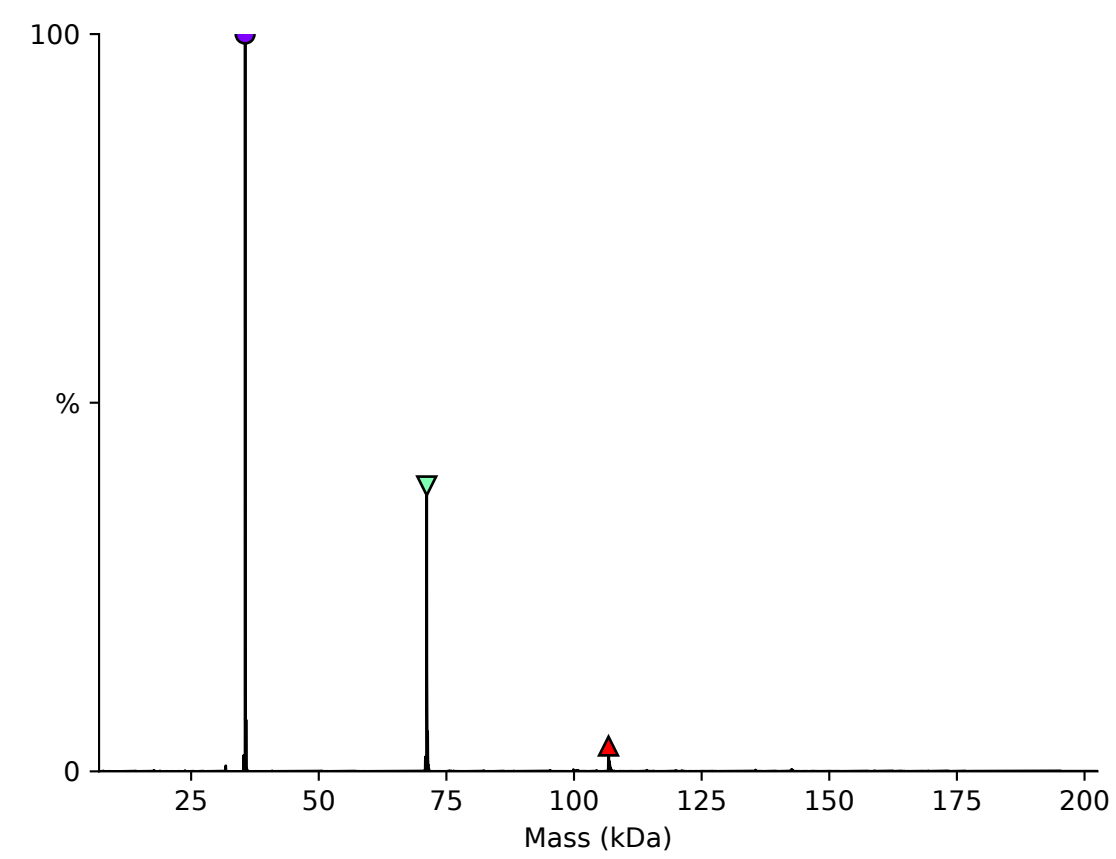

Symbol	Mass (Da)	Intensity[1]	Expected	Difference (Da)	Name/Species/Oligomer
○	35575	100	35577	−2	1[RK764]
▽	71151	39	71155	−4	2[RK764]
△	106755	3	106732	23	3[RK764]

[1]Intensity values should not be assumed as quantitative

RTR16_C4

Laboratory: Wysocki-AN
Client: Baker-Ryan
Shipment Date: 2021_03_17
Data Recorded: Fri, 19 Mar 2021, 00:15
Instrument: Q Exactive UHMR Orbitrap
Study: OBE 200 mM AmAc, RO24
Sample Conditions: 25mM Tris pH8, 300mM NaCl
Concentration (ug/uL): 1.0
Injection (uL): 0.5
Comment: ThermoOBE RO24 2.1x50mm 3um 80A
Date Analyzed: Tue, 23 Mar 2021, 11:57 Eastern Daylight Time
File Name: 20210318_Ryan_RK765_6k_IST030_HCD120

Quality Check:
Average Peaks Score: 0.72

Expected Oligomers:
Passed:
Mixed:
Failed:

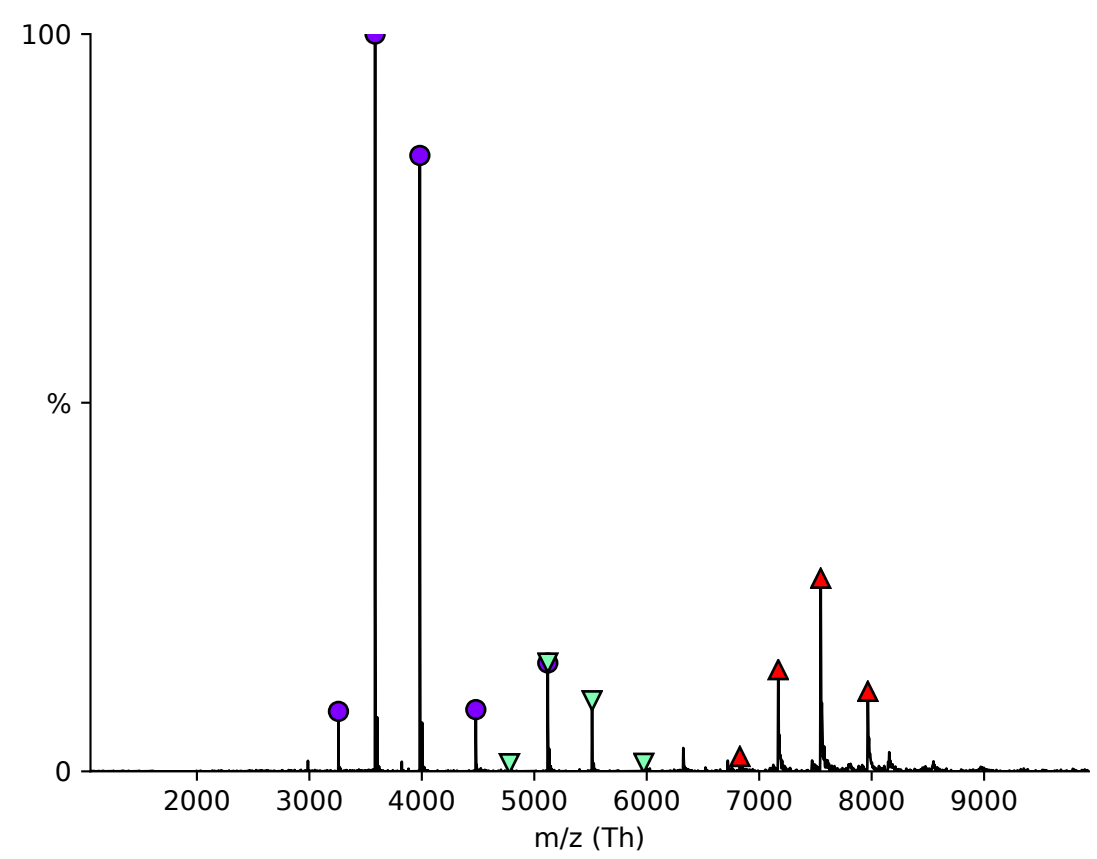

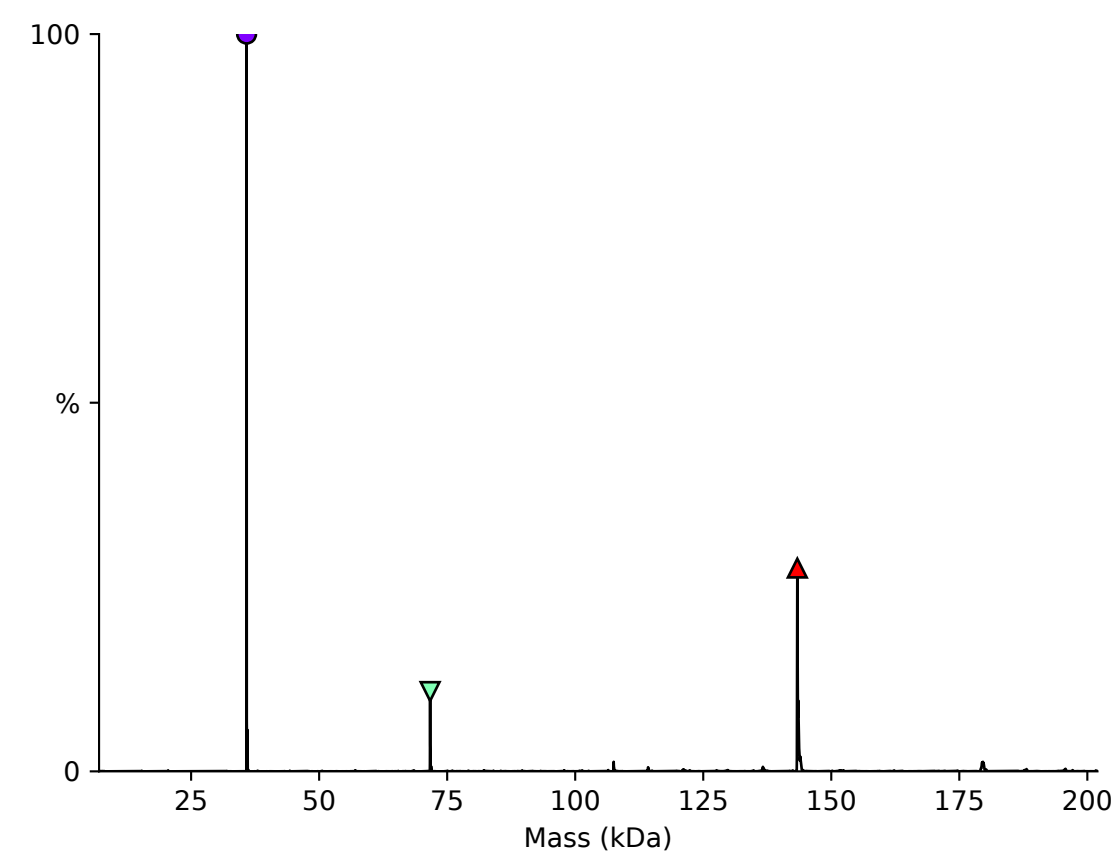

Symbol	Mass (Da)	Intensity[1]	Expected	Difference (Da)	Name/Species/Oligomer
○	35833	100	35837	−4	1[RK765]
▽	71670	11	71673	−3	2[RK765]
△	143370	28	143347	23	4[RK765]

[1]Intensity values should not be assumed as quantitative

RTR17_C4

Laboratory: Wysocki-AN
Client: Baker-Ryan
Shipment Date: 2021_03_17
Data Recorded: Fri, 19 Mar 2021, 00:26
Instrument: Q Exactive UHMR Orbitrap
Study: OBE 200 mM AmAc, RO24
Sample Conditions: 25mM Tris pH8, 300mM NaCl
Concentration (ug/uL): 1.0
Injection (uL): 0.5
Comment: ThermoOBE RO24 2.1x50mm 3um 80A
Date Analyzed: Tue, 23 Mar 2021, 11:59 Eastern Daylight Time
File Name: 20210318_Ryan_RK766_6k_IST030_HCD120

Quality Check:
Average Peaks Score: 0.7

Expected Oligomers:
Passed:
Mixed:
Failed:

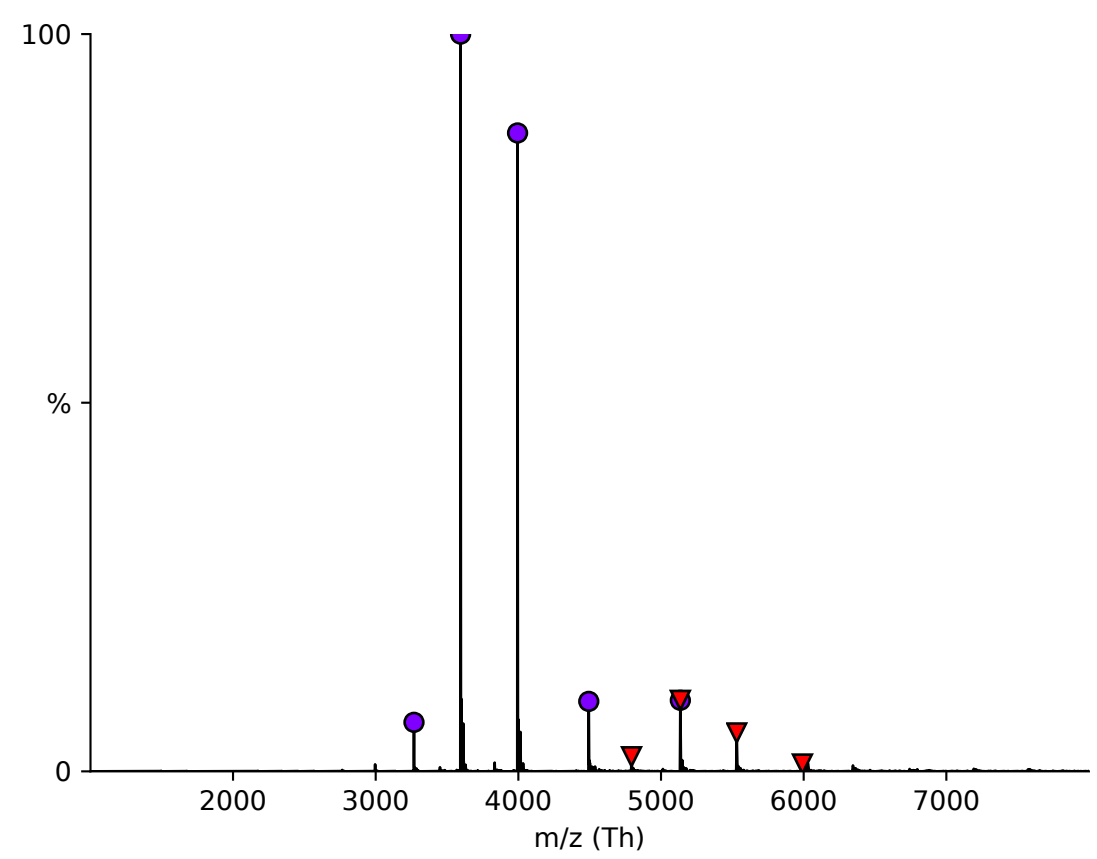

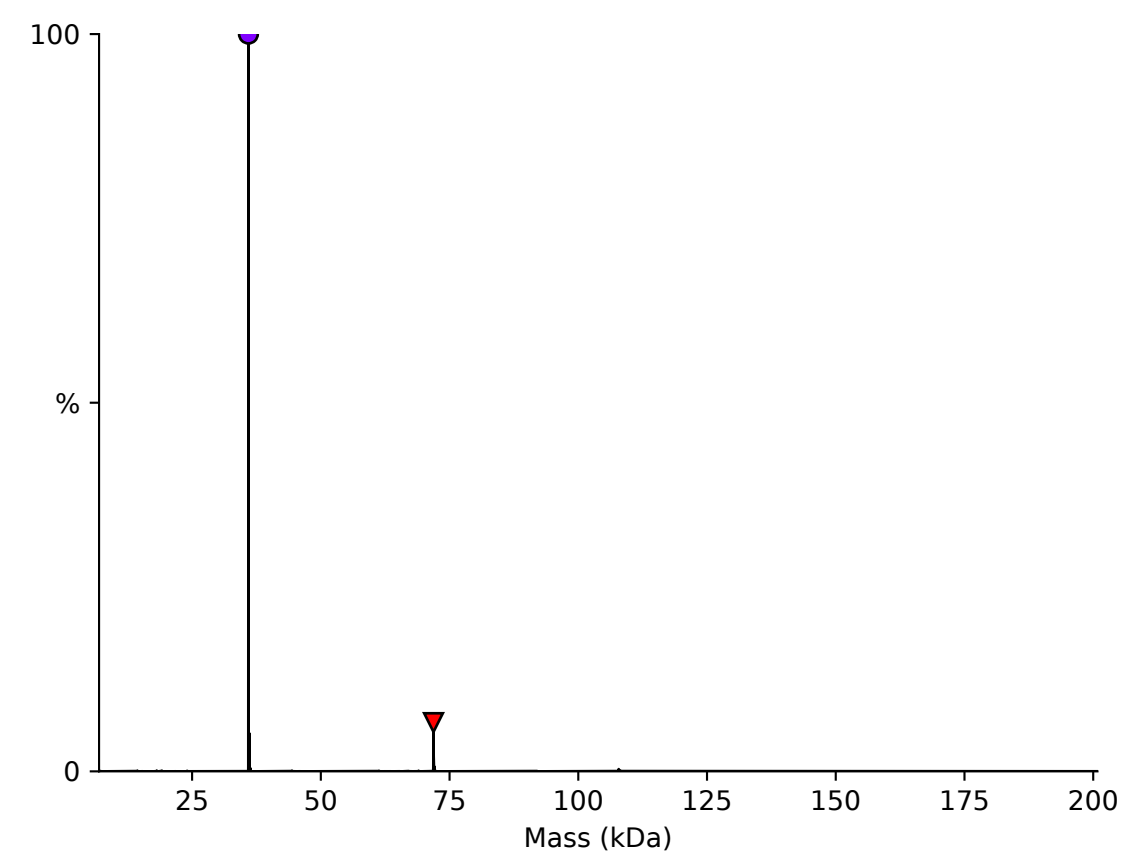

Symbol	Mass (Da)	Intensity[1]	Expected	Difference (Da)	Name/Species/Oligomer
○	35941	100	35942	−1	1[RK766]
▽	71882	7	71884	−2	2[RK766]

[1]Intensity values should not be assumed as quantitative

RTR18_C4

Laboratory: Wysocki-AN
Client: Baker-Ryan
Shipment Date: 2021_03_17
Data Recorded: Fri, 19 Mar 2021, 00:37
Instrument: Q Exactive UHMR Orbitrap
Study: OBE 200 mM AmAc, RO24
Sample Conditions: 25mM Tris pH8, 300mM NaCl
Concentration (ug/uL): 0.961
Injection (uL): 0.5
Comment: ThermoOBE RO24 2.1x50mm 3um 80A
Date Analyzed: Tue, 23 Mar 2021, 12:04 Eastern Daylight Time
File Name: 20210318_Ryan_RK767_6k_IST030_HCD120

Quality Check:
Average Peaks Score: 0.91

Expected Oligomers:
Passed:
Mixed:
Failed:

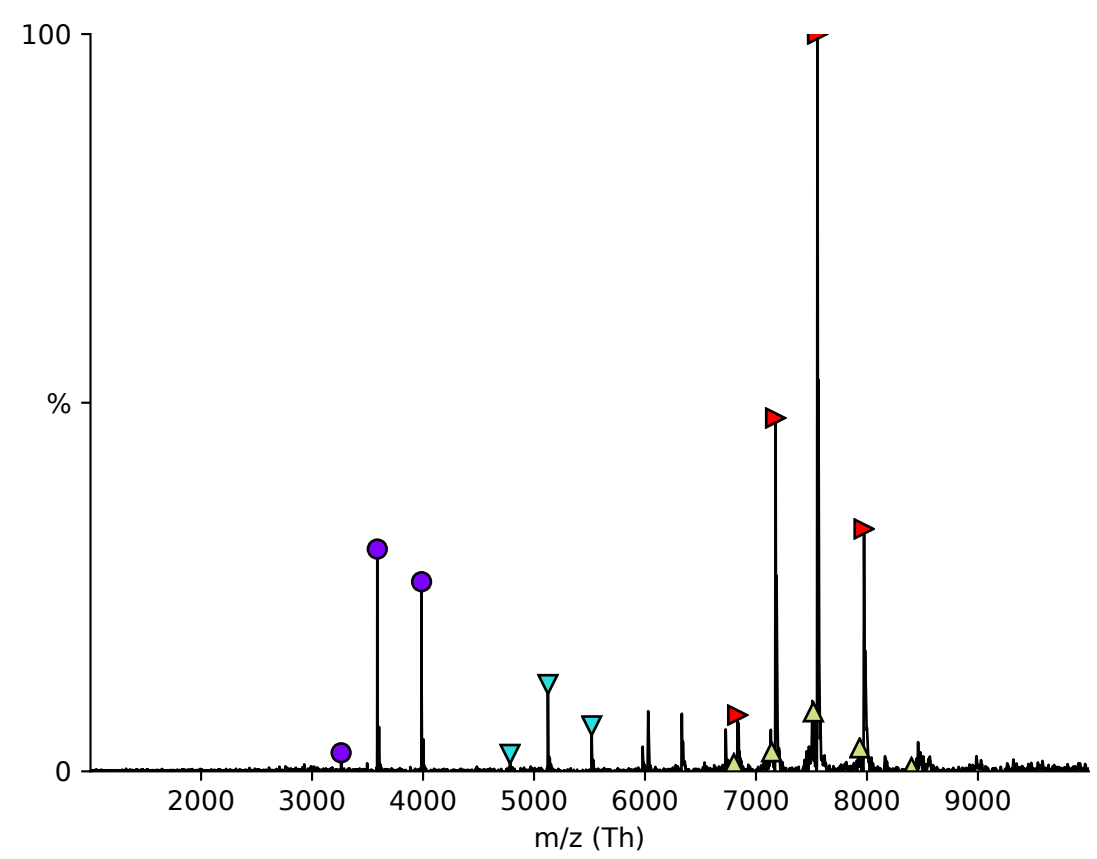

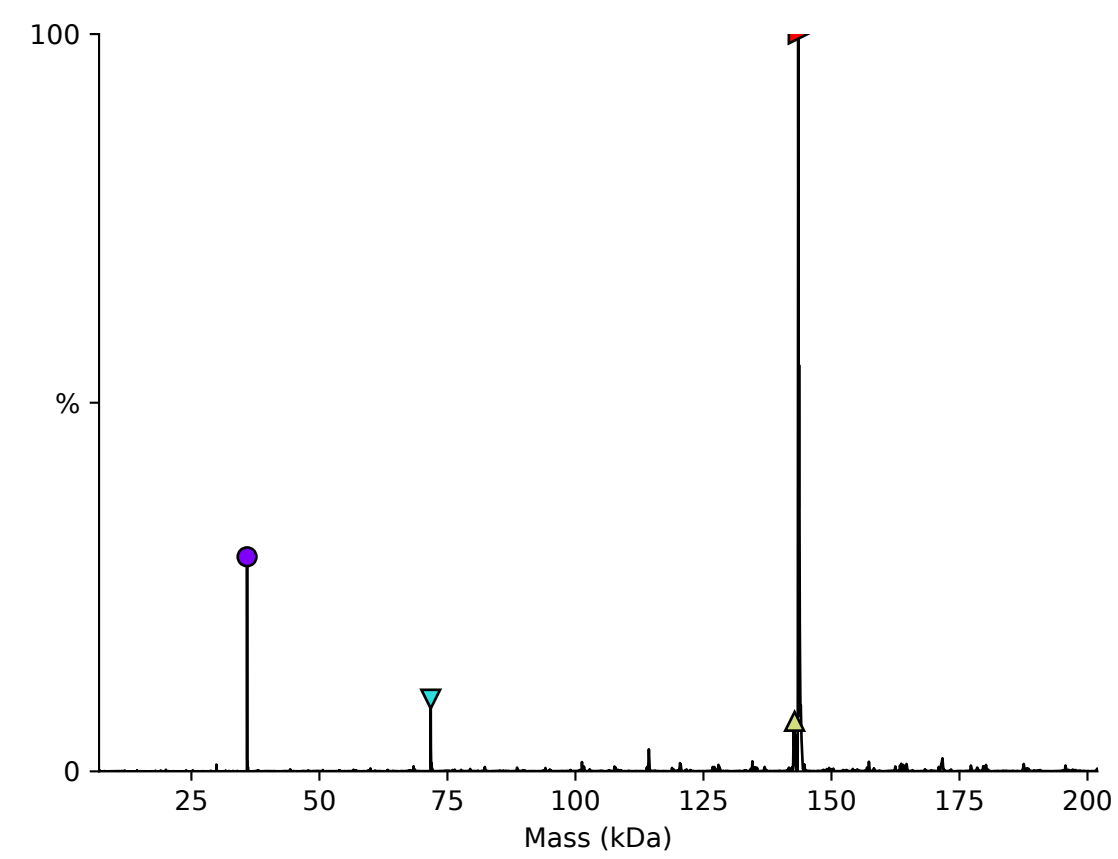

Symbol	Mass (Da)	Intensity[1]	Expected	Difference (Da)	Name/Species/Oligomer
○	35868	29	35871	−3	1[RK767]
▽	71738	10	71742	−4	2[RK767]
△	142801	7	143483	−682	
▷	143504	100	143483	21	4[RK767]

[1]Intensity values should not be assumed as quantitative

RTR19_C4

Laboratory: Wysocki-AN
Client: Baker-Ryan
Shipment Date: 2021_03_17
Data Recorded: Fri, 19 Mar 2021, 00:48
Instrument: Q Exactive UHMR Orbitrap
Study: OBE 200 mM AmAc, RO24
Sample Conditions: 25mM Tris pH8, 300mM NaCl
Concentration (ug/uL): 1.0
Injection (uL): 0.5
Comment: ThermoOBE RO24 2.1x50mm 3um 80A
Date Analyzed: Tue, 23 Mar 2021, 12:07 Eastern Daylight Time
File Name: 20210318_Ryan_RK768_6k_IST030_HCD120

Quality Check:
Average Peaks Score: 0.83

Expected Oligomers:
Passed:
Mixed:
Failed:

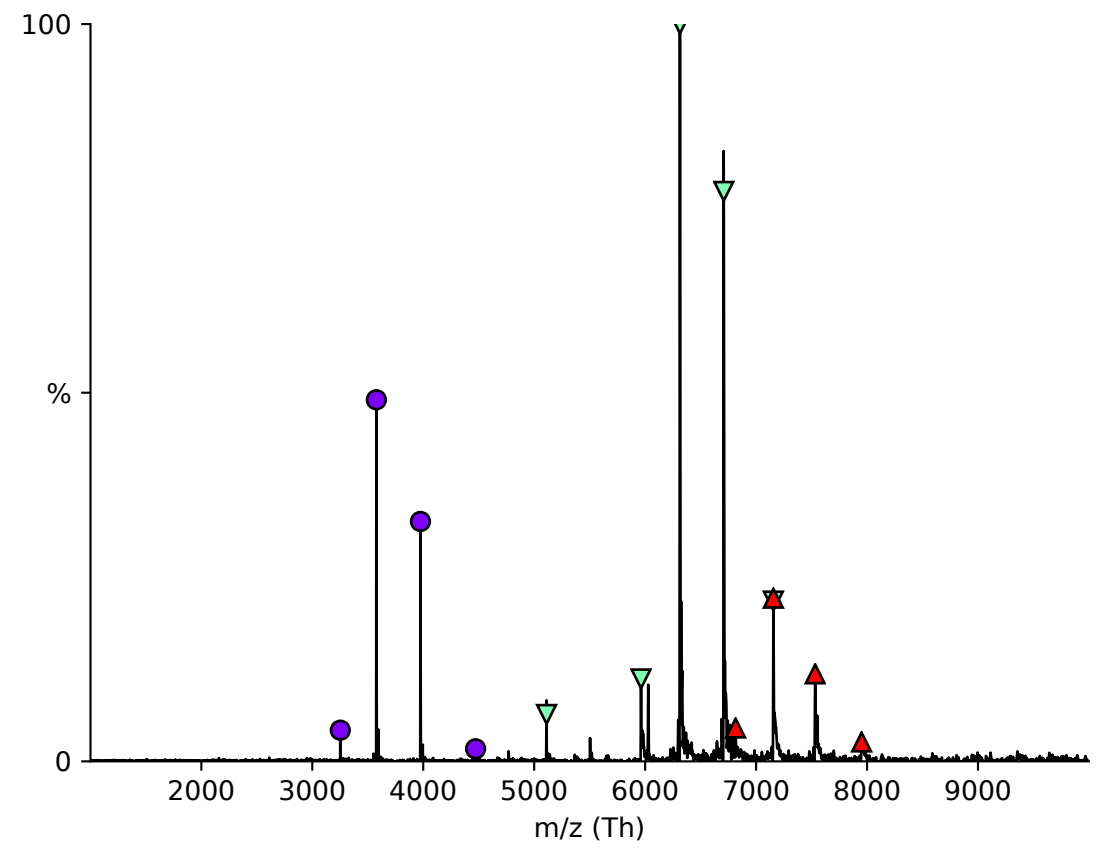

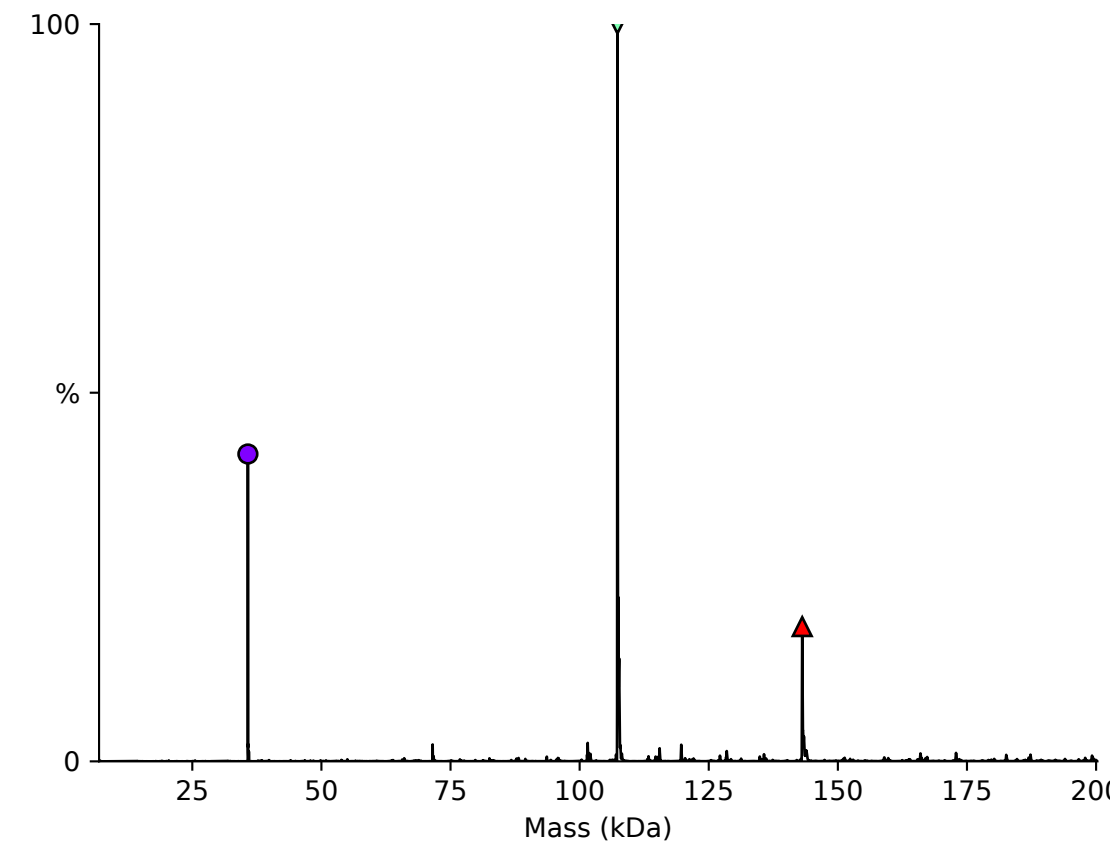

Symbol	Mass (Da)	Intensity[1]	Expected	Difference (Da)	Name/Species/Oligomer
○	35765	42	35769	−4	1[RK768]
▽	107319	100	107306	13	3[RK768]
△	143097	18	143075	22	4[RK768]

[1]Intensity values should not be assumed as quantitative

RTR20_C4

Laboratory: Wysocki-AN
Client: Baker-Ryan
Shipment Date: 2021_03_17
Data Recorded: Fri, 19 Mar 2021, 00:59
Instrument: Q Exactive UHMR Orbitrap
Study: OBE 200 mM AmAc, RO24
Sample Conditions: 25mM Tris pH8, 300mM NaCl
Concentration (ug/uL): 0.969
Injection (uL): 0.5
Comment: ThermoOBE RO24 2.1x50mm 3um 80A
Date Analyzed: Tue, 23 Mar 2021, 12:11 Eastern Daylight Time
File Name: 20210318_Ryan_RK769_6k_IST030_HCD120

Quality Check:
Average Peaks Score: 0.82

Expected Oligomers:
Passed:
Mixed:
Failed:

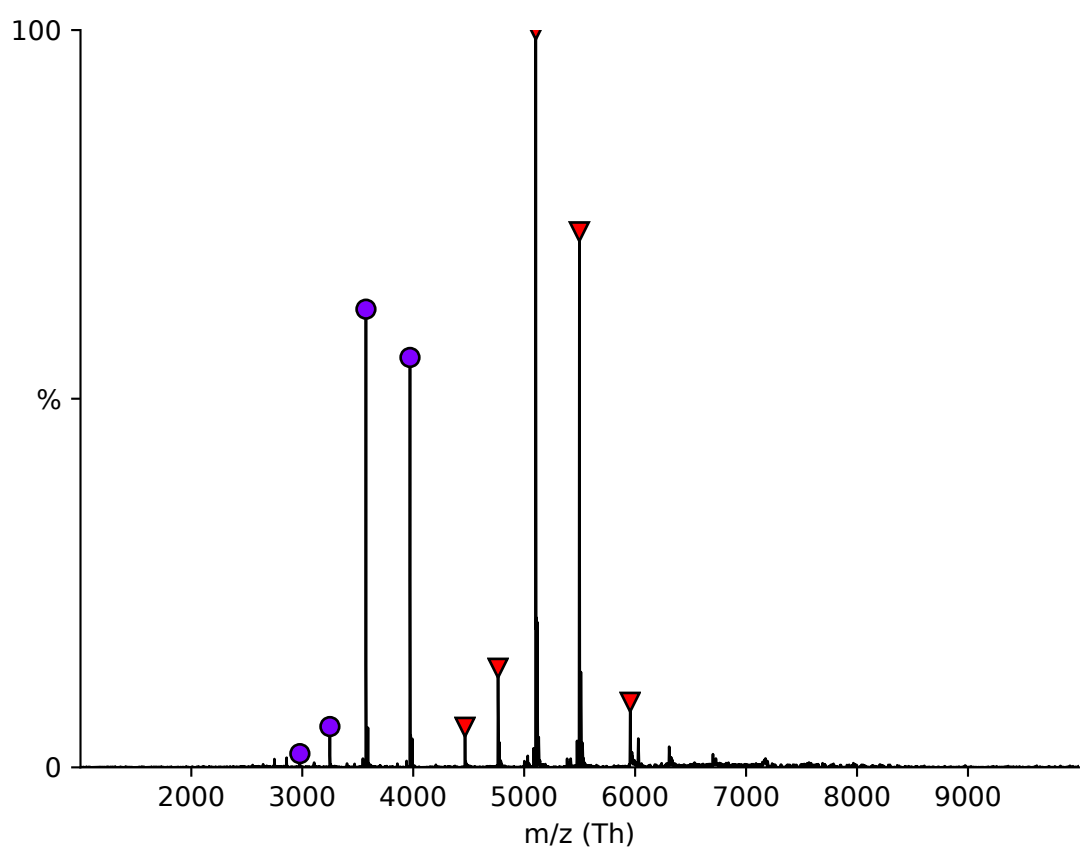

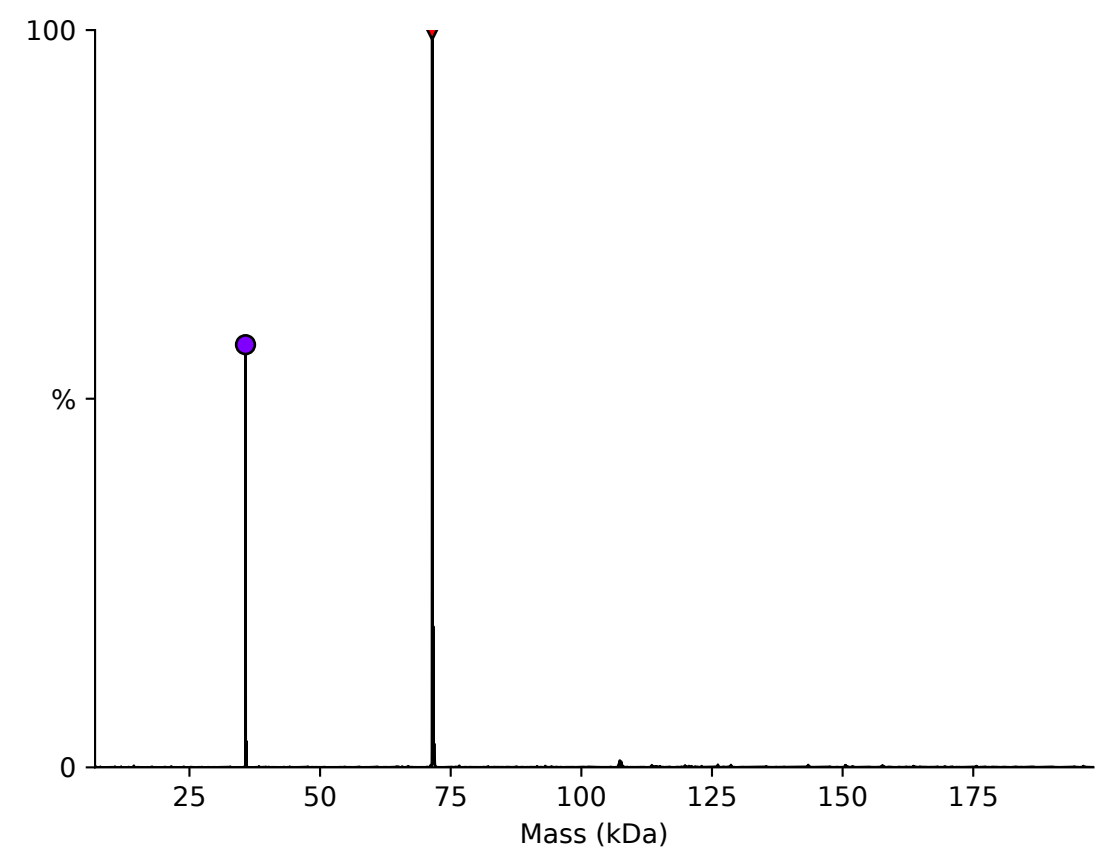

Symbol	Mass (Da)	Intensity[1]	Expected	Difference (Da)	Name/Species/Oligomer
○	35728	57	35730	−2	1[RK769]
▽	71451	100	71461	−10	2[RK769]

[1]Intensity values should not be assumed as quantitative

RTR21_C4

Laboratory: Wysocki-AN
Client: Baker-Ryan
Shipment Date: 2021_03_17
Data Recorded: Fri, 19 Mar 2021, 01:23
Instrument: Q Exactive UHMR Orbitrap
Study: OBE 200 mM AmAc, RO24
Sample Conditions: 25mM Tris pH8, 300mM NaCl
Concentration (ug/uL): 1.0
Injection (uL): 0.5
Comment: ThermoOBE RO24 2.1x50mm 3um 80A
Date Analyzed: Tue, 23 Mar 2021, 12:25 Eastern Daylight Time
File Name: 20210318_Ryan_RK771_6k_IST030_HCD120

Quality Check:
Average Peaks Score: 0.7

Expected Oligomers:
Passed:
Mixed:
Failed:

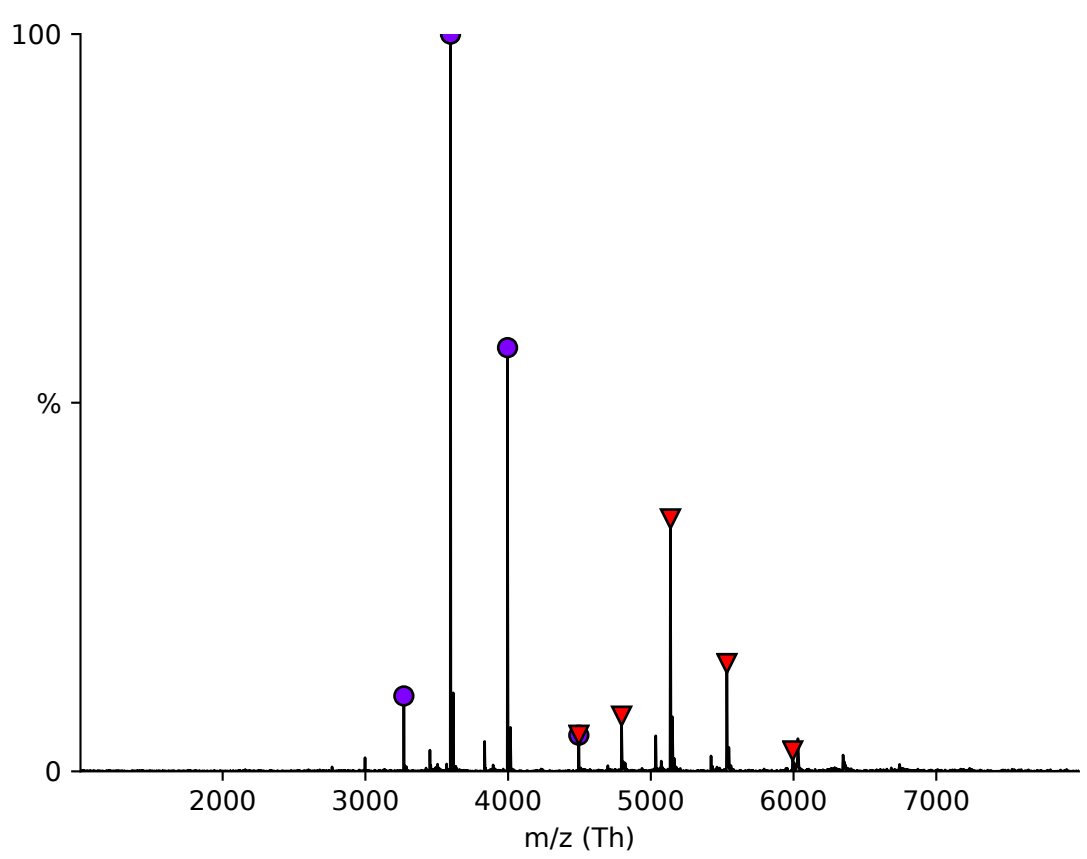

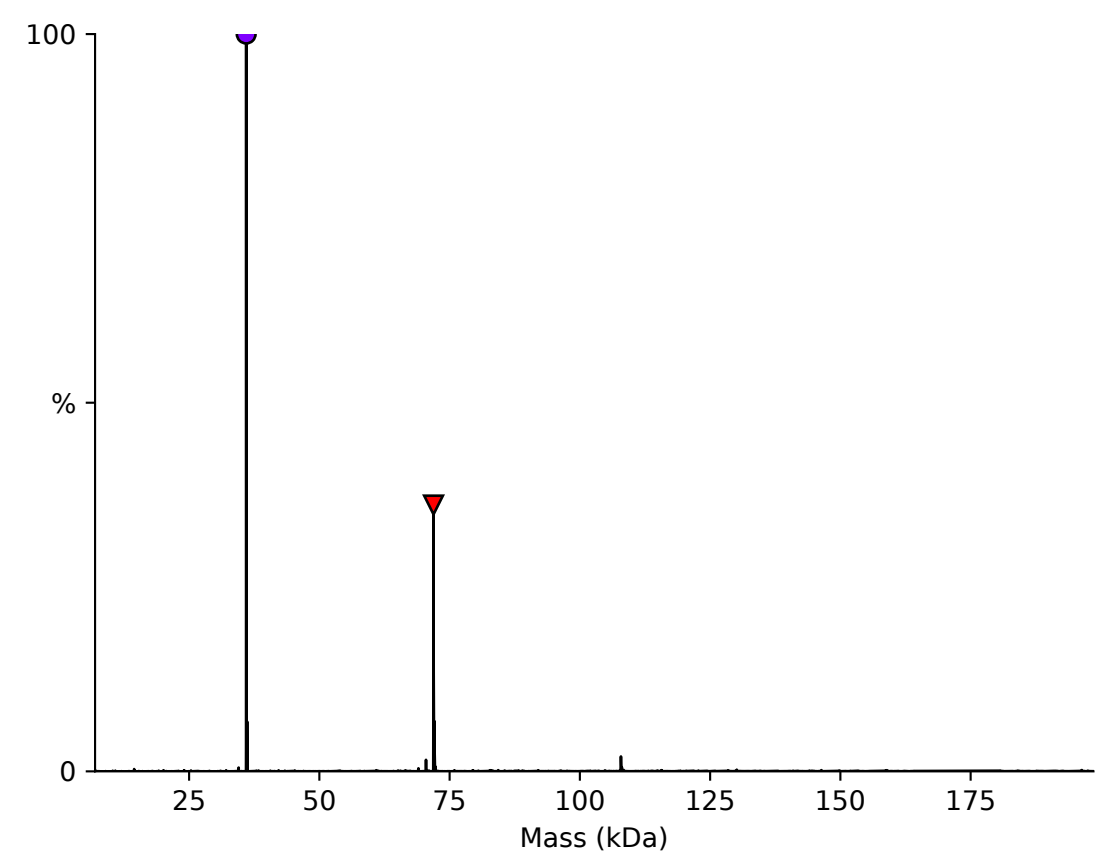

Symbol	Mass (Da)	Intensity[1]	Expected	Difference (Da)	Name/Species/Oligomer
○	35955	100	35959	−4	1[RK770]
▽	71913	36	71918	−5	2[RK770]

Corrected sample labeling error between RK771 and RK770

[1]Intensity values should not be assumed as quantitative

RTR22_C4

Laboratory: Wysocki-AN
Client: Baker-Ryan
Shipment Date: 2021_03_17
Data Recorded: Fri, 19 Mar 2021, 01:12
Instrument: Q Exactive UHMR Orbitrap
Study: OBE 200 mM AmAc, RO24
Sample Conditions: 25mM Tris pH8, 300mM NaCl
Concentration (ug/uL): 0.828
Injection (uL): 0.5
Comment: ThermoOBE RO24 2.1x50mm 3um 80A
Date Analyzed: Tue, 23 Mar 2021, 12:26 Eastern Daylight Time
File Name: 20210318_Ryan_RK770_6k_IST030_HCD120

Quality Check:
Average Peaks Score: 0.7

Expected Oligomers:
Passed:
Mixed:
Failed:

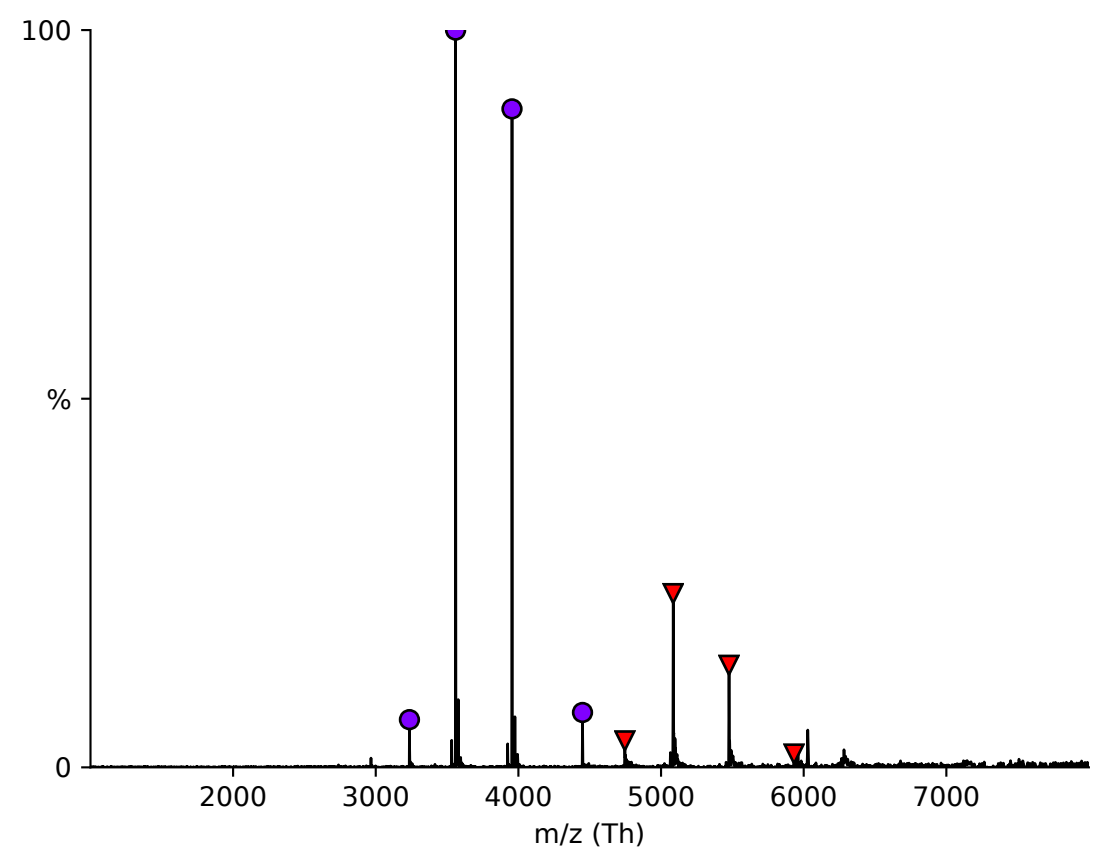

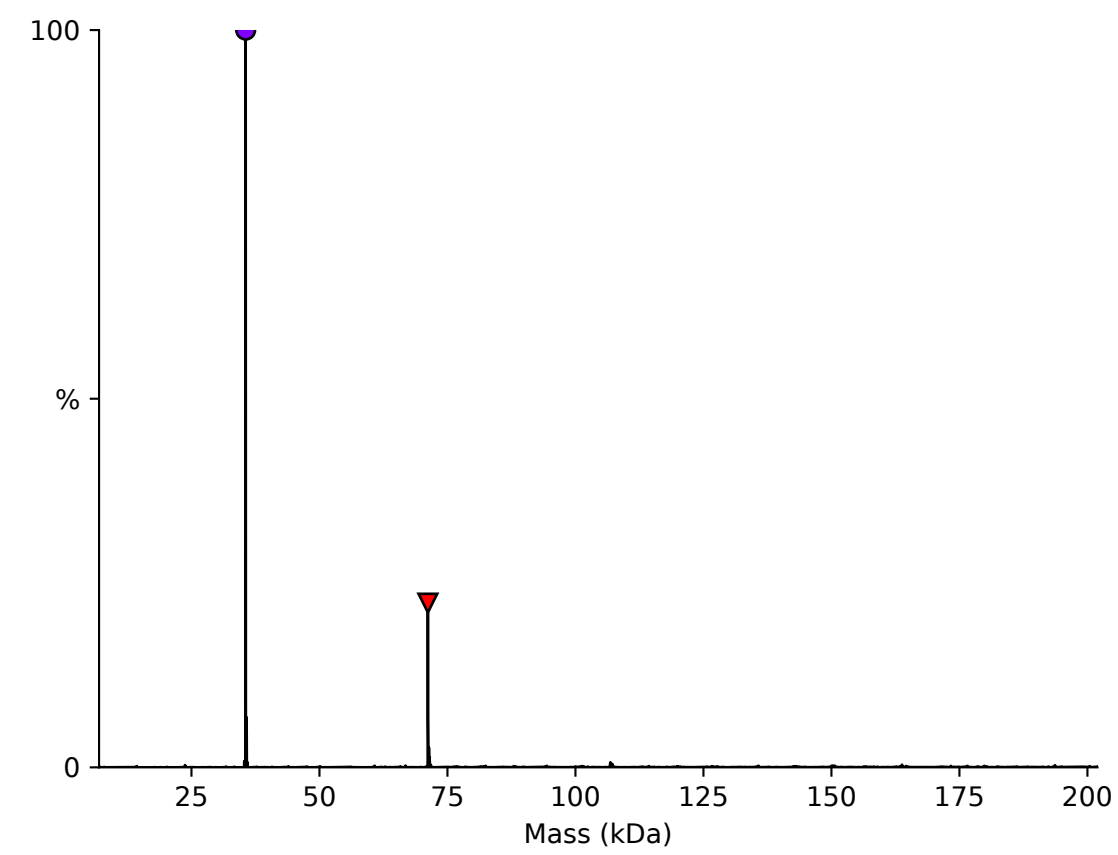

Symbol	Mass (Da)	Intensity[1]	Expected	Difference (Da)	Name/Species/Oligomer
○	35587	100	35590	−3	1[RK771]
▽	71177	22	71181	−4	2[RK771]

Corrected sample labeling error between RK771 and RK770

[1]Intensity values should not be assumed as quantitative

RTR23_C4

Laboratory: Wysocki-AN
Client: Baker-Ryan
Shipment Date: 2021_03_17
Data Recorded: Fri, 19 Mar 2021, 01:45
Instrument: Q Exactive UHMR Orbitrap
Study: OBE 200 mM AmAc, RO24
Sample Conditions: 25mM Tris pH8, 300mM NaCl
Concentration (ug/uL): 1.0
Injection (uL): 0.5
Comment: ThermoOBE RO24 2.1x50mm 3um 80A
Date Analyzed: Tue, 23 Mar 2021, 12:33 Eastern Daylight Time
File Name: 20210318_Ryan_RK773_6k_IST030_HCD120

Quality Check:
Average Peaks Score: 0.72

Expected Oligomers:
Passed:
Mixed:
Failed:

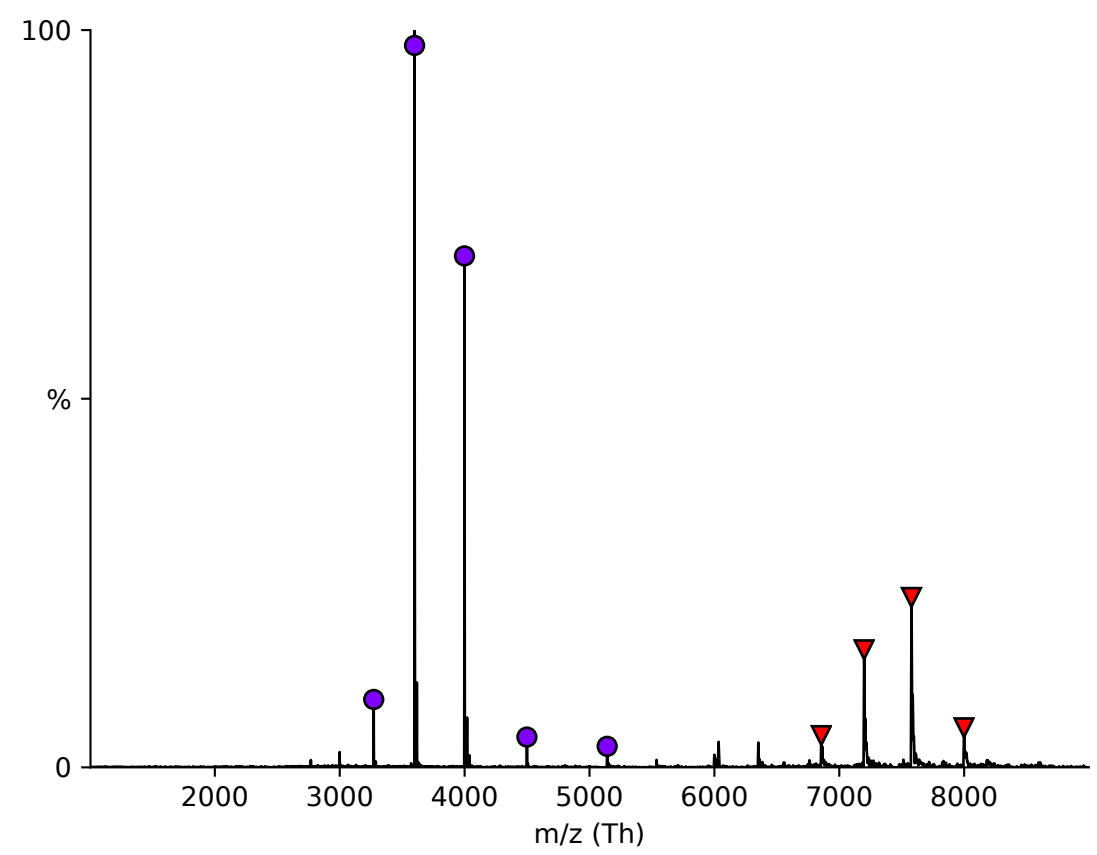

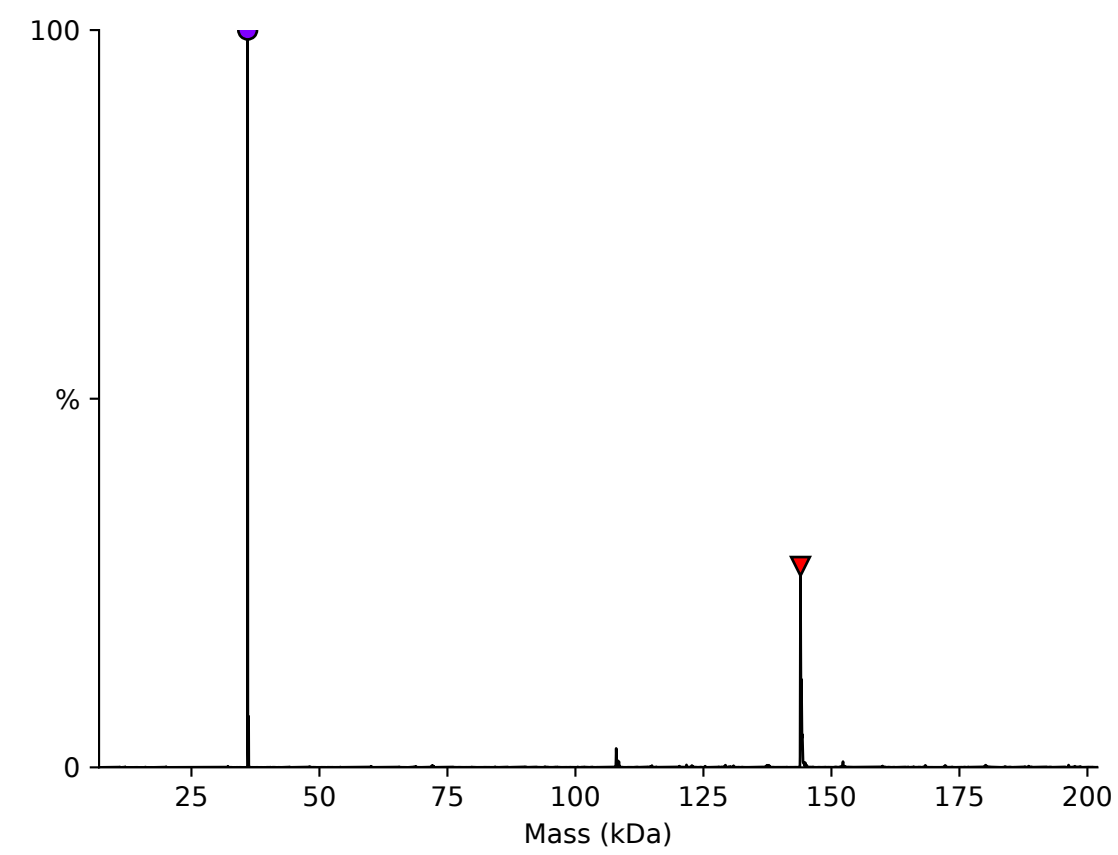

Symbol	Mass (Da)	Intensity[1]	Expected	Difference (Da)	Name/Species/Oligomer
○	35983	100	35986	−3	1[RK772]
▽	143959	27	143944	15	4[RK772]

Corrected sample labeling error between RK773 and RK772

[1]Intensity values should not be assumed as quantitative

RTR24_C4

Laboratory: Wysocki-AN
Client: Baker-Ryan
Shipment Date: 2021_03_17
Data Recorded: Fri, 19 Mar 2021, 01:34
Instrument: Q Exactive UHMR Orbitrap
Study: OBE 200 mM AmAc, RO24
Sample Conditions: 25mM Tris pH8, 300mM NaCl
Concentration (ug/uL): 1.0
Injection (uL): 0.5
Comment: ThermoOBE RO24 2.1x50mm 3um 80A
Date Analyzed: Tue, 23 Mar 2021, 12:34 Eastern Daylight Time
File Name: 20210318_Ryan_RK772_6k_IST030_HCD120

Quality Check:
Average Peaks Score: 0.77

Expected Oligomers:
Passed:
Mixed:
Failed:

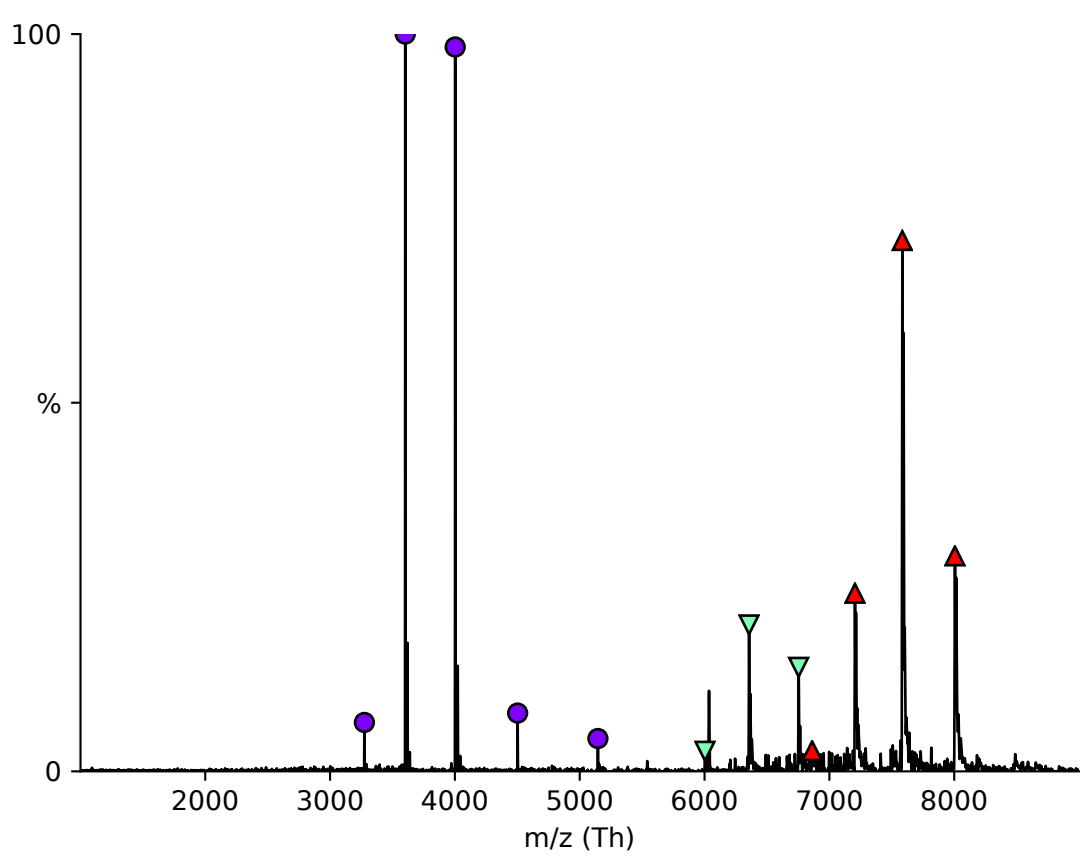

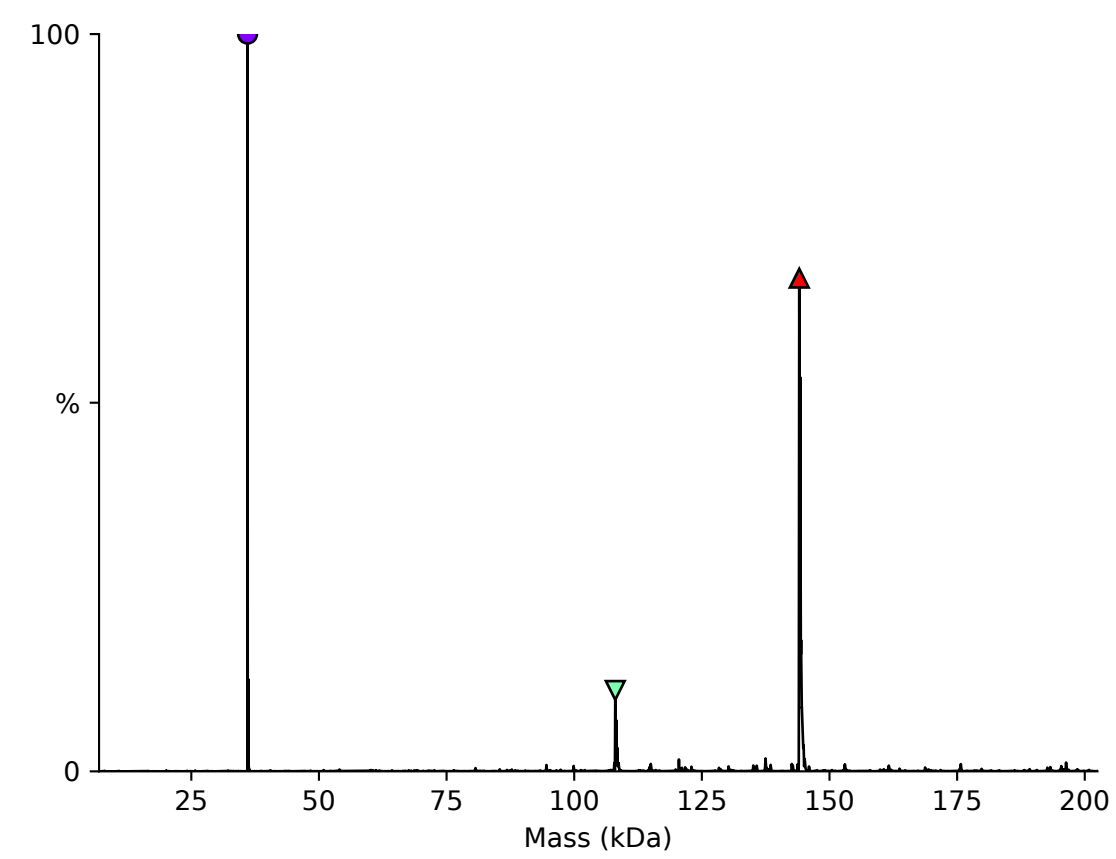

Symbol	Mass (Da)	Intensity[1]	Expected	Difference (Da)	Name/Species/Oligomer
○	36012	100	36015	−3	1[RK773]
▽	108040	11	108045	−5	3[RK773]
△	144078	67	144060	18	4[RK773]

Corrected sample labeling error between RK773 and RK772

[1]Intensity values should not be assumed as quantitative

RTR0_C2

Laboratory: Wysocki-SC, SML
Client: Ryan K
Shipment Date:
Data Recorded: Mon, 18 Oct 2021, 14:06
Instrument: Q Exactive UHMR Orbitrap
Study: OBE 200 mM AmAc, RO33
Sample Conditions: 25mM Tris pH 8, 300mM NaCl
Concentration (ug/uL): 1.004913079
Injection (uL): 0.995110941
Comment: Masses are slightly different than reported. Looks like close to all dimer though
Date Analyzed: Mon, 18 Oct 2021, 16:27 Eastern Daylight Time
File Name: 20211014_RK990_6k_IST060_HCD005

Quality Check:
Average Peaks Score: 0.89

Expected Oligomers:
Passed:
Mixed:
Failed:

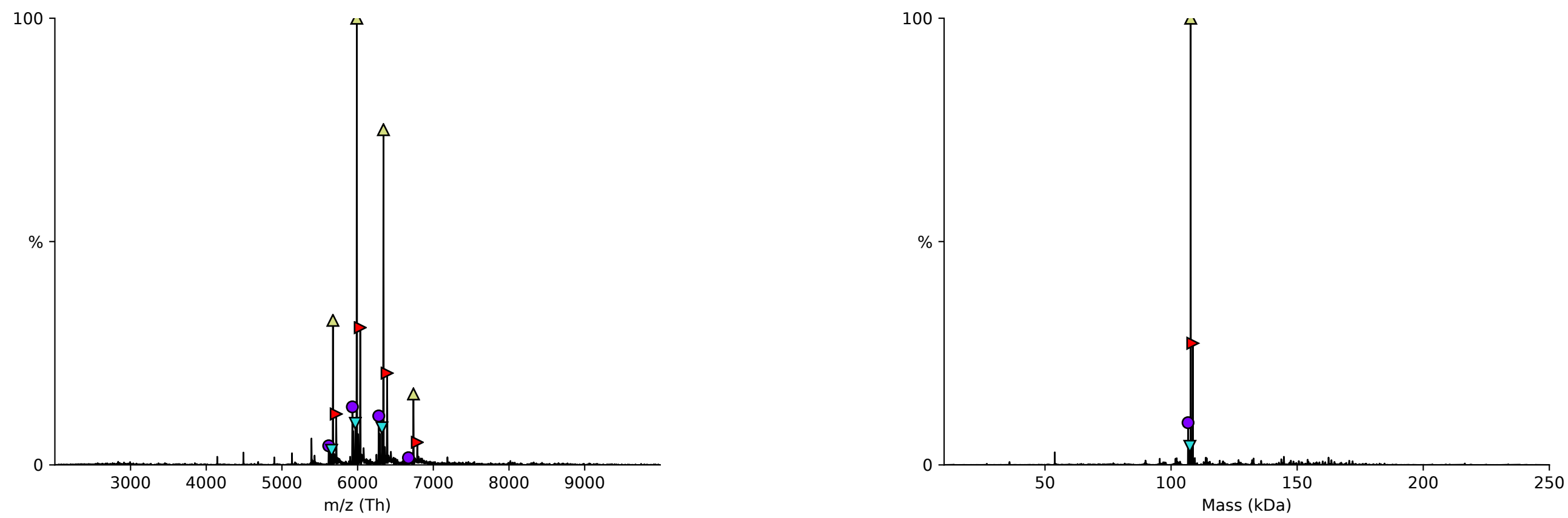

Symbol	Mass (Da)	Intensity[1]	Expected	Difference (Da)	Name/Species/Oligomer
○	106728	9	109706	−2978	
▽	107447	4	109706	−2259	
△	107783	100	109706	−1923	
▷	108603	27	109706	−1103	

[1] Intensity values should not be considered quantitative

RTR05_C2

Laboratory: Wysocki-SC, SML
Client: Ryan K
Shipment Date:
Data Recorded: Mon, 18 Oct 2021, 14:06
Instrument: Q Exactive UHMR Orbitrap
Study: OBE 200 mM AmAc, RO33
Sample Conditions: 25mM Tris pH 8, 300mM NaCl
Concentration (ug/uL): 1.007007768
Injection (uL): 0.993040999
Comment: Very clean looking spectrum. There's an unknown species in there as well that's also dimerizing with the known species
Date Analyzed: Mon, 18 Oct 2021, 16:00 Eastern Daylight Time
File Name: 20211014_RK985_6k_IST060_HCD005

Quality Check:
Average Peaks Score: 0.87

Expected Oligomers:
Passed:
Mixed:
Failed:

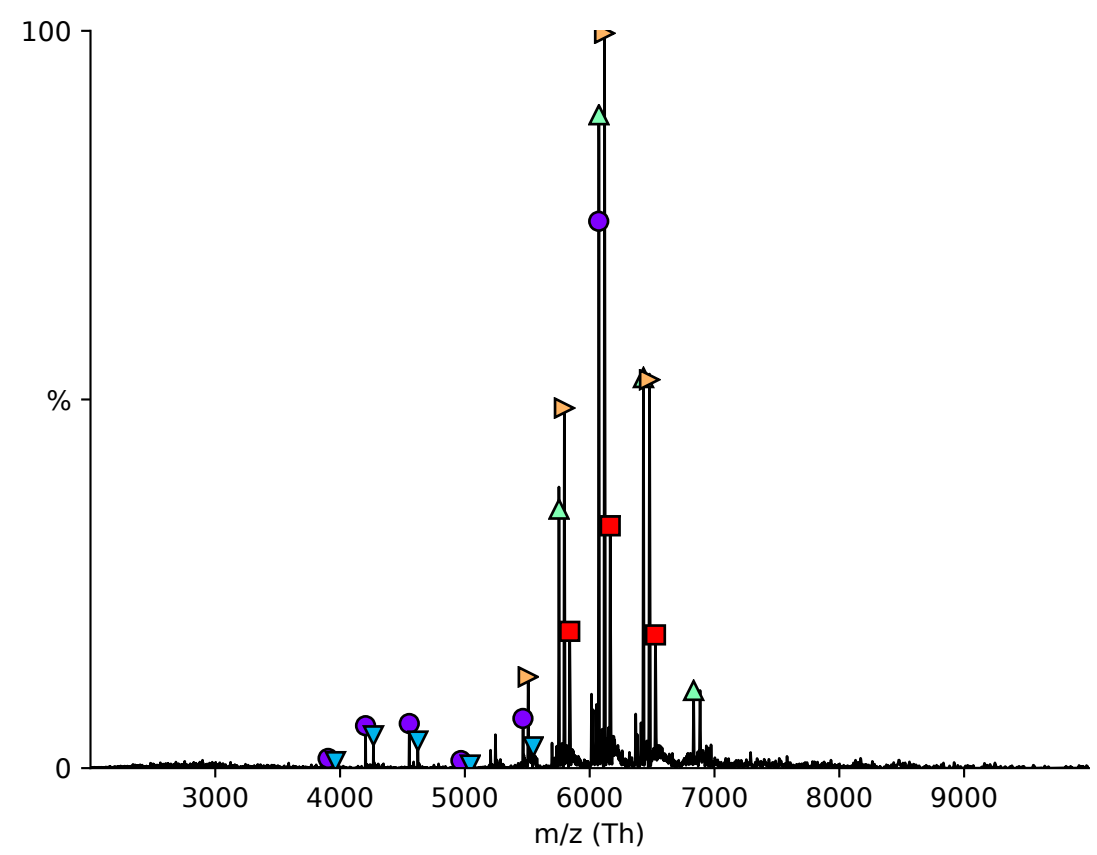

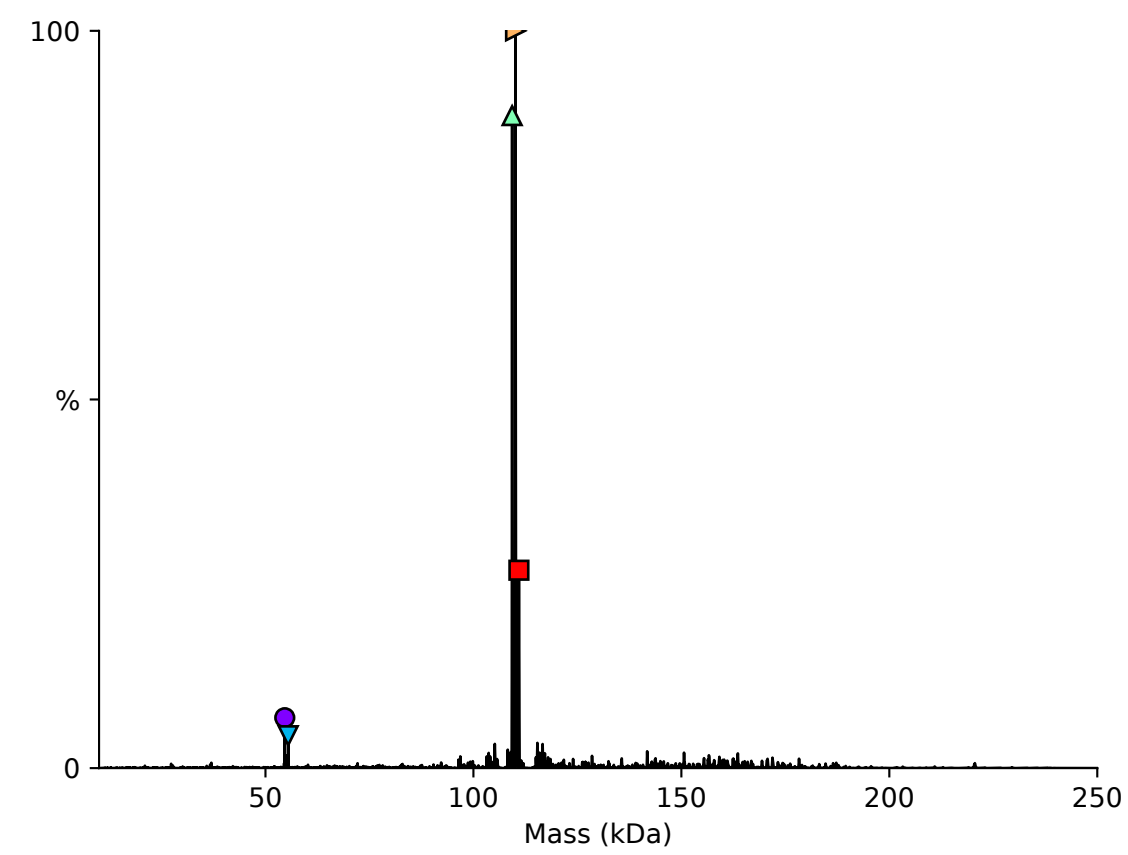

Symbol	Mass (Da)	Intensity[1]	Expected	Difference (Da)	Name/Species/Oligomer
○	54641	7	54641	0	1[unknown]
▽	55465	4	55482	−17	1[RK985]
△	109309	89	109282	27	2[unknown]
▷	110126	100	110123	3	1[unknown] 1[RK985]
□	110940	27	110964	−24	2[RK985]

[1] Intensity values should not be considered quantitative

RTR11noCys_C2

Laboratory: Wysocki-SC, SML
Client: Ryan K
Shipment Date:
Data Recorded: Mon, 18 Oct 2021, 14:06
Instrument: Q Exactive UHMR Orbitrap
Study: OBE 200 mM AmAc, RO33
Sample Conditions: 25mM Tris pH 8, 300mM NaCl
Concentration (ug/uL): 1.015319029
Injection (uL): 0.984912103
Comment: Unknown species is present with the known species. The unknown also dimerizes with the known species
Date Analyzed: Mon, 18 Oct 2021, 16:21 Eastern Daylight Time
File Name: 20211014_RK986_6k_IST060_HCD005

Quality Check:
Average Peaks Score: 0.92

Expected Oligomers:
Passed:
Mixed:
Failed:

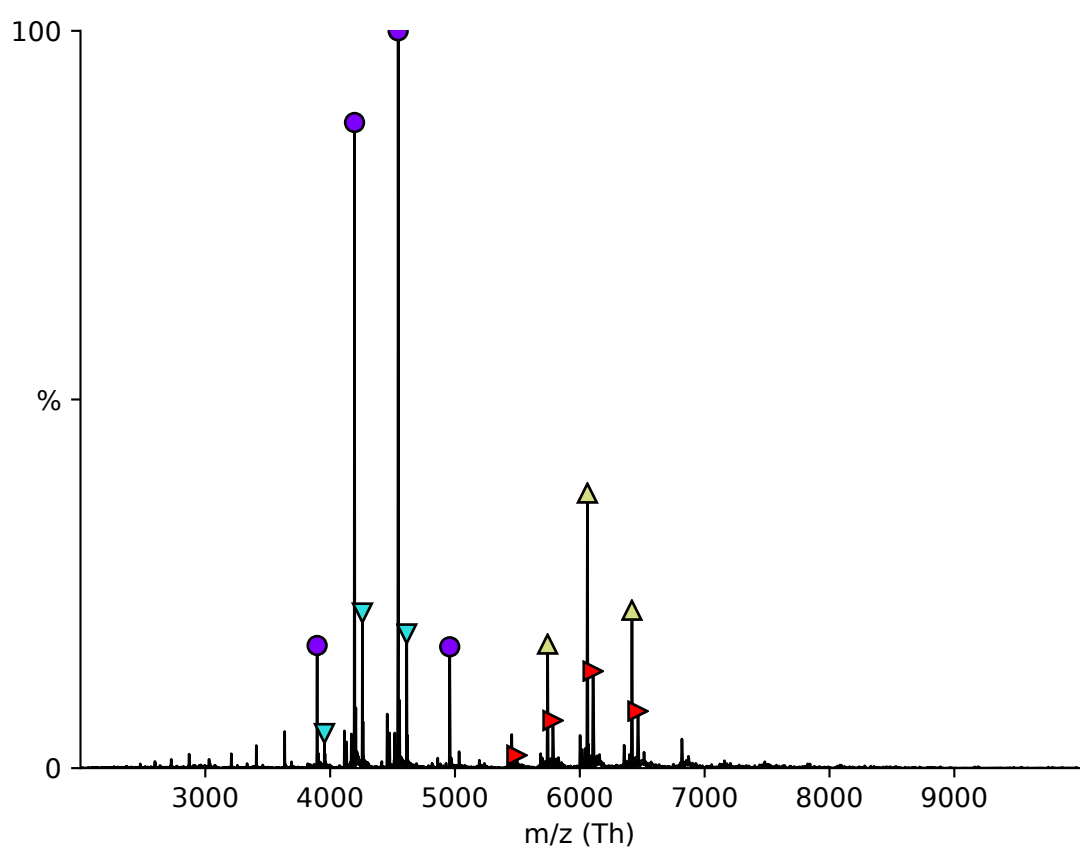

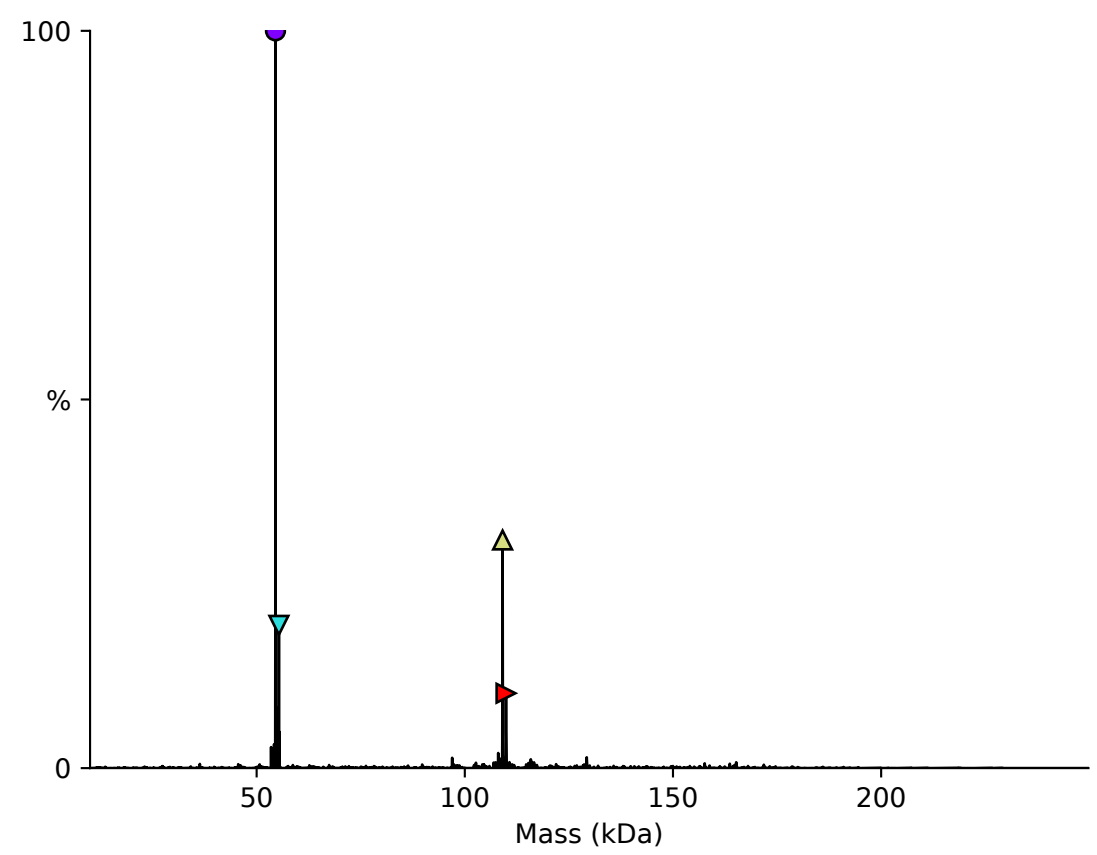

Symbol	Mass (Da)	Intensity[1]	Expected	Difference (Da)	Name/Species/Oligomer
○	54525	100	54525	0	1[Unknown]
▽	55347	19	55365	−18	1[RK986]
△	109076	31	109050	26	2[Unknown]
▷	109893	10	109890	3	1[Unknown] 1[RK986]

[1]Intensity values should not be considered quantitative

RTR18noCys_C2

Laboratory: Wysocki-SC, SML
Client: Ryan K
Shipment Date:
Data Recorded: Mon, 18 Oct 2021, 14:06
Instrument: Q Exactive UHMR Orbitrap
Study: OBE 200 mM AmAc, RO33
Sample Conditions: 25mM Tris pH 8, 300mM NaCl
Concentration (ug/uL): 1.002368502
Injection (uL): 0.997637095
Comment: only found monomer in this spectrum
Date Analyzed: Mon, 18 Oct 2021, 16:18 Eastern Daylight Time
File Name: 20211014_RK988_6k_IST005_HCD005

Quality Check:
Average Peaks Score: 0.88

Expected Oligomers:
Passed:
Mixed:
Failed:

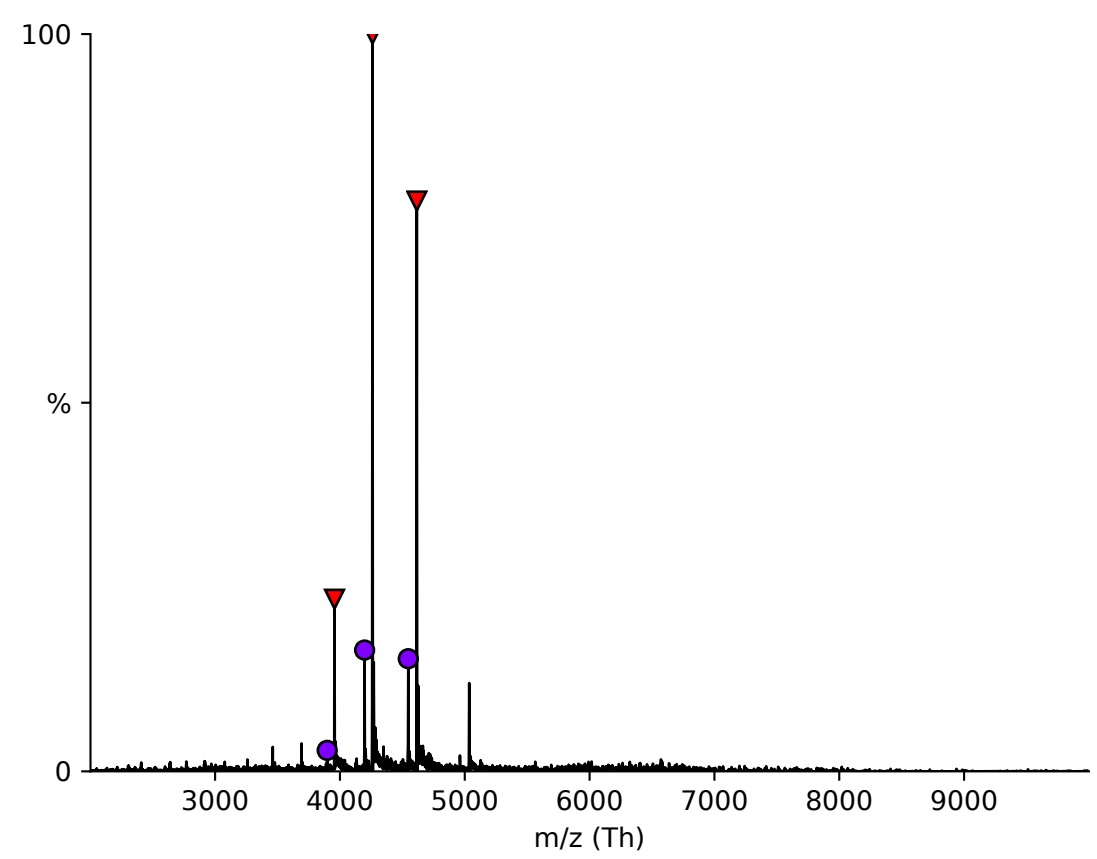

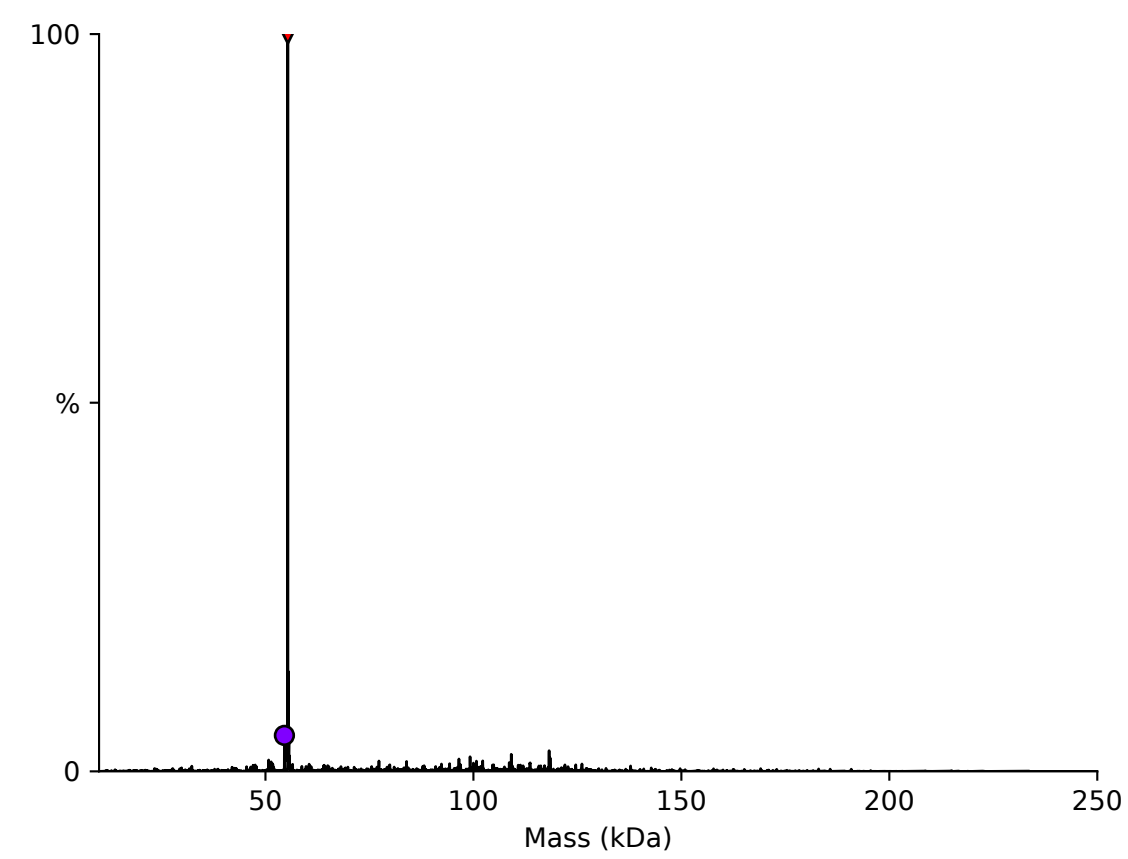

Symbol	Mass (Da)	Intensity[1]	Expected	Difference (Da)	Name/Species/Oligomer
○	54556	5	54556	0	1[Unknown]
▽	55378	100	55394	−16	1[RK988]

[1]Intensity values should not be considered quantitative

RTR0_C3

Laboratory: Wysocki-SC, SML
Client: Ryan K
Shipment Date:
Data Recorded: Mon, 18 Oct 2021, 14:06
Instrument: Q Exactive UHMR Orbitrap
Study: OBE 200 mM AmAc, RO33
Sample Conditions: 25mM Tris pH 8, 300mM NaCl
Concentration (ug/uL): 1.001664613
Injection (uL): 0.998338153
Comment: Found the disulfide linked trimer but also found some other proteins as well.
Date Analyzed: Tue, 19 Oct 2021, 12:44 Eastern Daylight Time
File Name: 20211014_RK996_6k_IST060_HCD005

Quality Check:
Average Peaks Score: 0.93

Expected Oligomers:
Passed:
Mixed:
Failed:

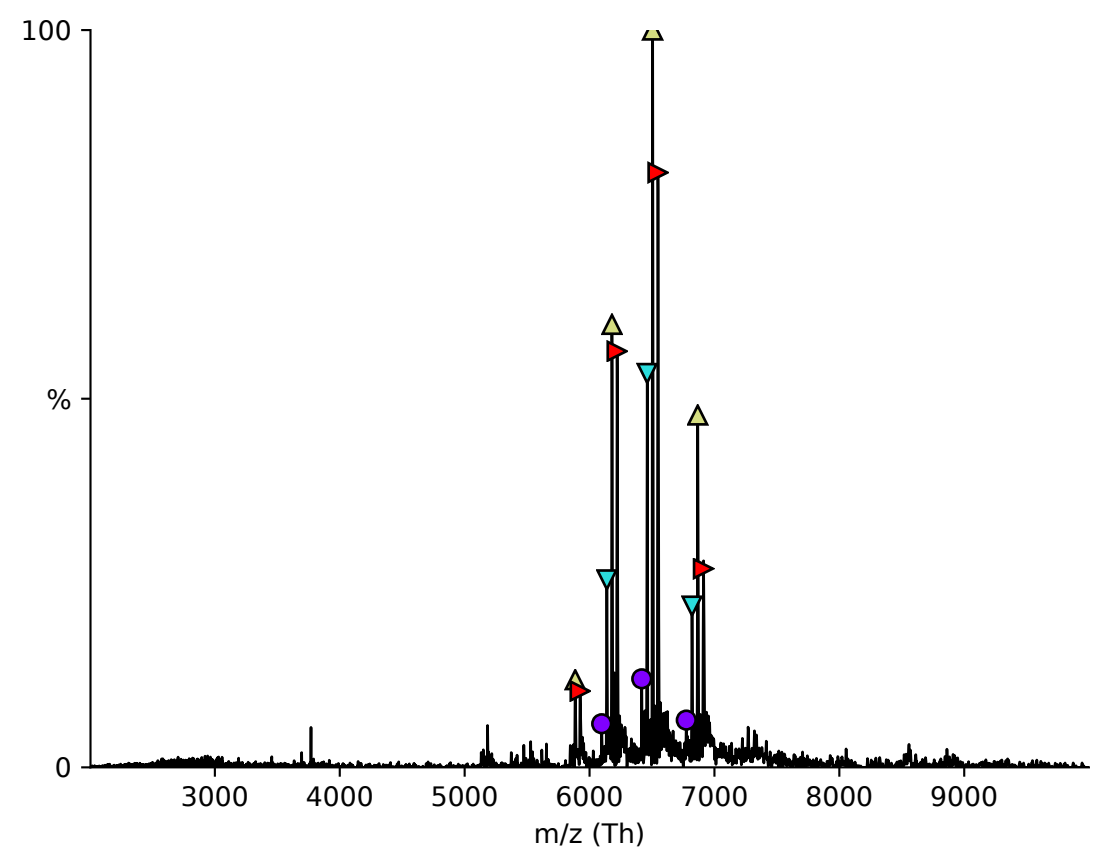

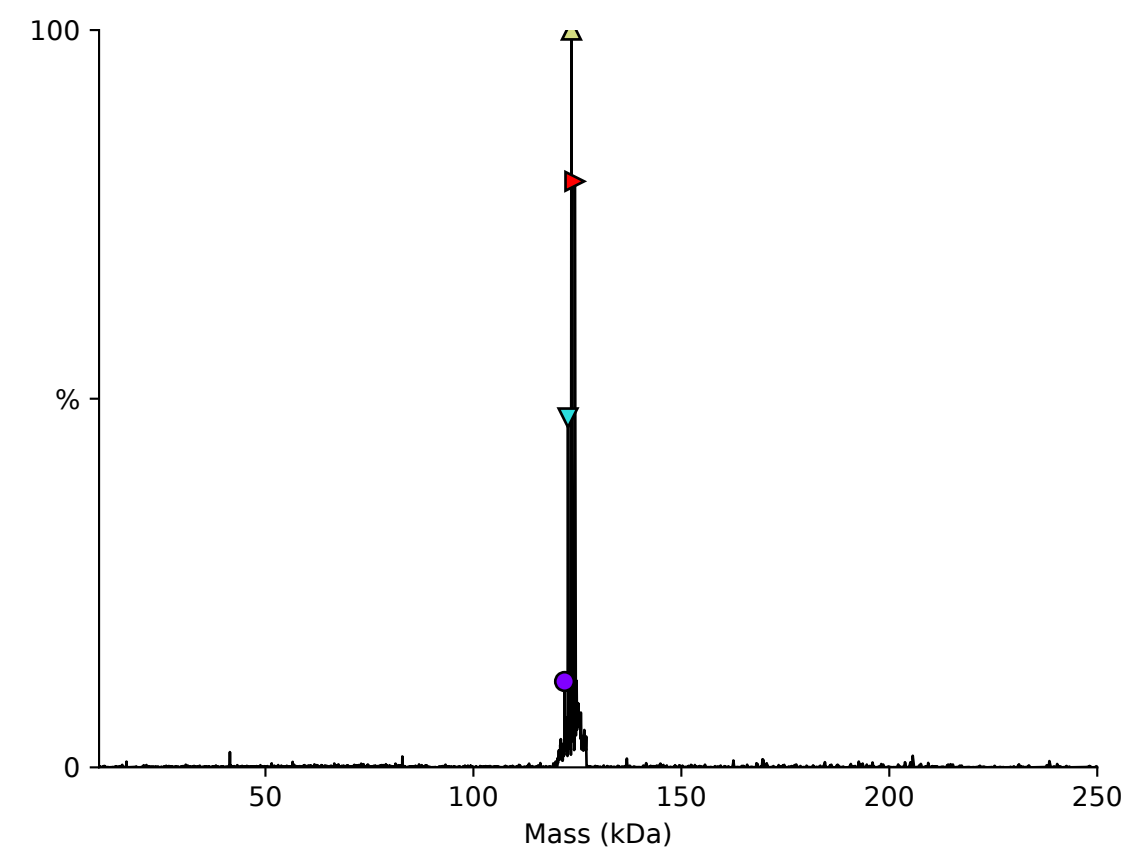

Symbol	Mass (Da)	Intensity[1]	Expected	Difference (Da)	Name/Species/Oligomer
○	121924	12	124408	−2484	
▽	122749	47	124408	−1659	
△	123571	100	124408	−837	
▷	124389	79	124408	−19	1[sulfide-linked trimer RK996]

[1] Intensity values should not be considered quantitative

RTR16_C3

Laboratory: Wysocki-SC, SML
Client: Ryan K
Shipment Date:
Data Recorded: Mon, 18 Oct 2021, 14:06
Instrument: Q Exactive UHMR Orbitrap
Study: OBE 200 mM AmAc, RO33
Sample Conditions: 25mM Tris pH 8, 300mM NaCl
Concentration (ug/uL): 1.018106059
Injection (uL): 0.98221594
Comment: An unknown species is binding with the known species here. Found monomers, dimers, and trimers though
Date Analyzed: Mon, 18 Oct 2021, 16:40 Eastern Daylight Time
File Name: 20211014_RK993_6k_IST060_HCD005

Quality Check:
Average Peaks Score: 0.9

Expected Oligomers:
Passed:
Mixed:
Failed:

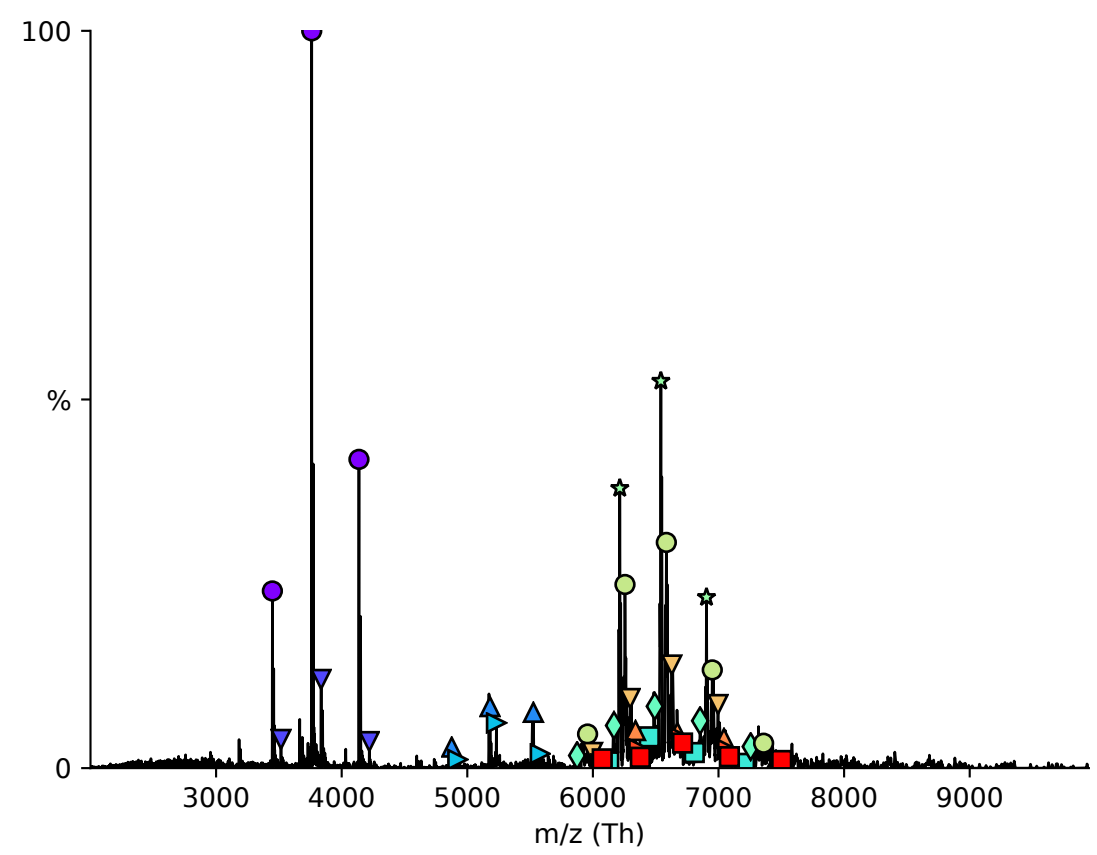

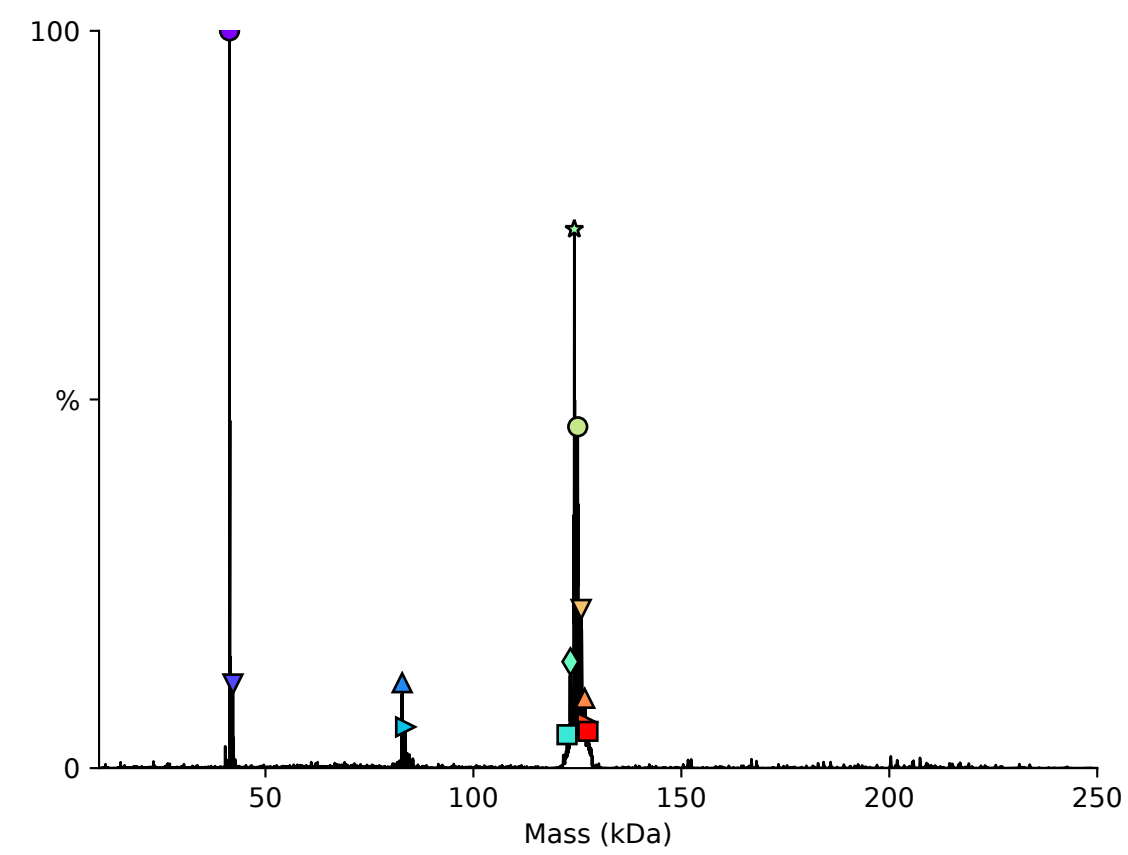

Symbol	Mass (Da)	Intensity[1]	Expected	Difference (Da)	Name/Species/Oligomer
○	41365	100	41365	0	1[Unknown]
▽	42187	11	42203	−16	1[RK993]
△	82875	12	82730	145	2[Unknown]
▷	83694	6	83568	126	1[Unknown] 1[RK993]
□	122507	5	124095	−1588	
◊	123339	14	124095	−756	
☆	124261	73	124095	166	3[Unknown]
○	125089	46	124933	156	2[Unknown] 1[RK993]
▽	125913	22	125771	142	1[Unknown] 2[RK993]
△	126766	9	126609	157	3[RK993]
▷	127292	6	126609	683	
□	127540	5	126609	931	

[1]Intensity values should not be considered quantitative

RTR0_C6

Laboratory: Wysocki-SC, SML
Client: Ryan K
Shipment Date:
Data Recorded: Mon, 18 Oct 2021, 14:06
Instrument: Q Exactive UHMR Orbitrap
Study: OBE 200 mM AmAc, RO33
Sample Conditions: 25mM Tris pH 8, 300mM NaCl
Concentration (ug/uL): 1.00432367
Injection (uL): 0.995694944
Comment: Found monomer and maybe hexamer. Mass for hexamer is off by about 5 kDa, but it's also not very well resolved.
Date Analyzed: Mon, 18 Oct 2021, 15:52 Eastern Daylight Time
File Name: 20211014_RK654_6k_IST060_HCD005

Quality Check:
Average Peaks Score: 0.79

Expected Oligomers:
Passed:
Mixed:
Failed:

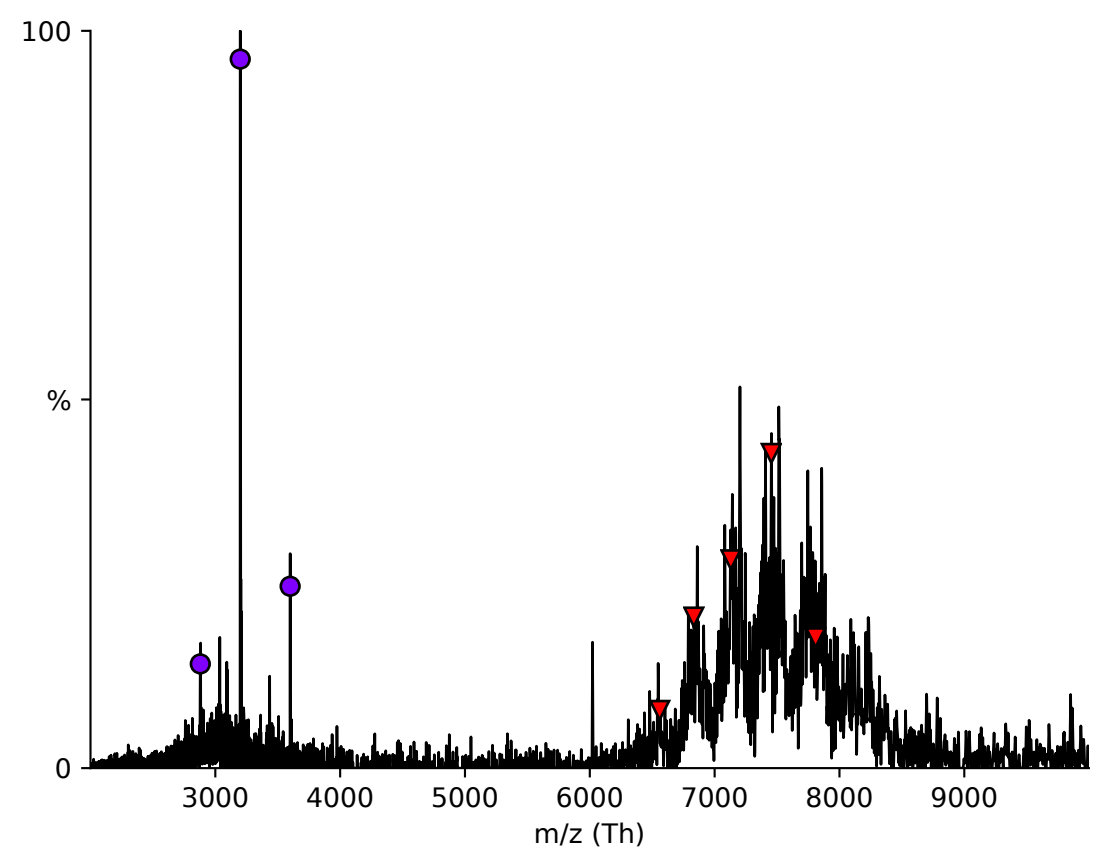

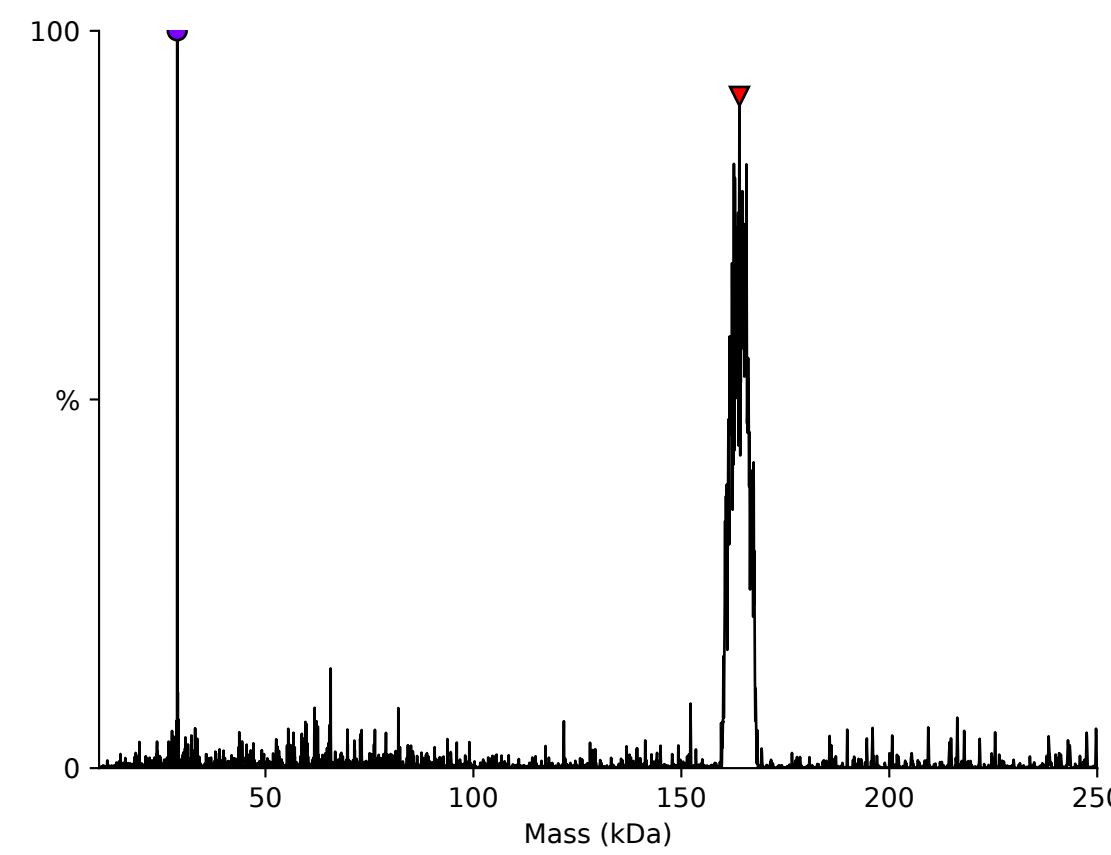

Symbol	Mass (Da)	Intensity[1]	Expected	Difference (Da)	Name/Species/Oligomer
○	28787	100	28798	−11	1[RK654]
▽	163969	91	172788	−8819	

[1]Intensity values should not be considered quantitative

RTR11noCys_C6

Laboratory: Wysocki-SC, SML
Client: Ryan K
Shipment Date:
Data Recorded: Mon, 11 Oct 2021, 16:58
Instrument: Q Exactive UHMR Orbitrap
Study: OBE 200 mM AmAc, RO33
Sample Conditions: 25mM Tris pH 8, 300mM NaCl
Concentration (ug/uL): 1.0
Injection (uL): 1.0
Comment: Lots of different proteins in this one. Higher mass appears to be pentamer rather than hexamer
Date Analyzed: Mon, 18 Oct 2021, 12:54 Eastern Daylight Time
File Name: 20211007_RK998_6k_IST060_HCD005

Quality Check:
Average Peaks Score: 0.87

Expected Oligomers:
Passed:
Mixed:
Failed:

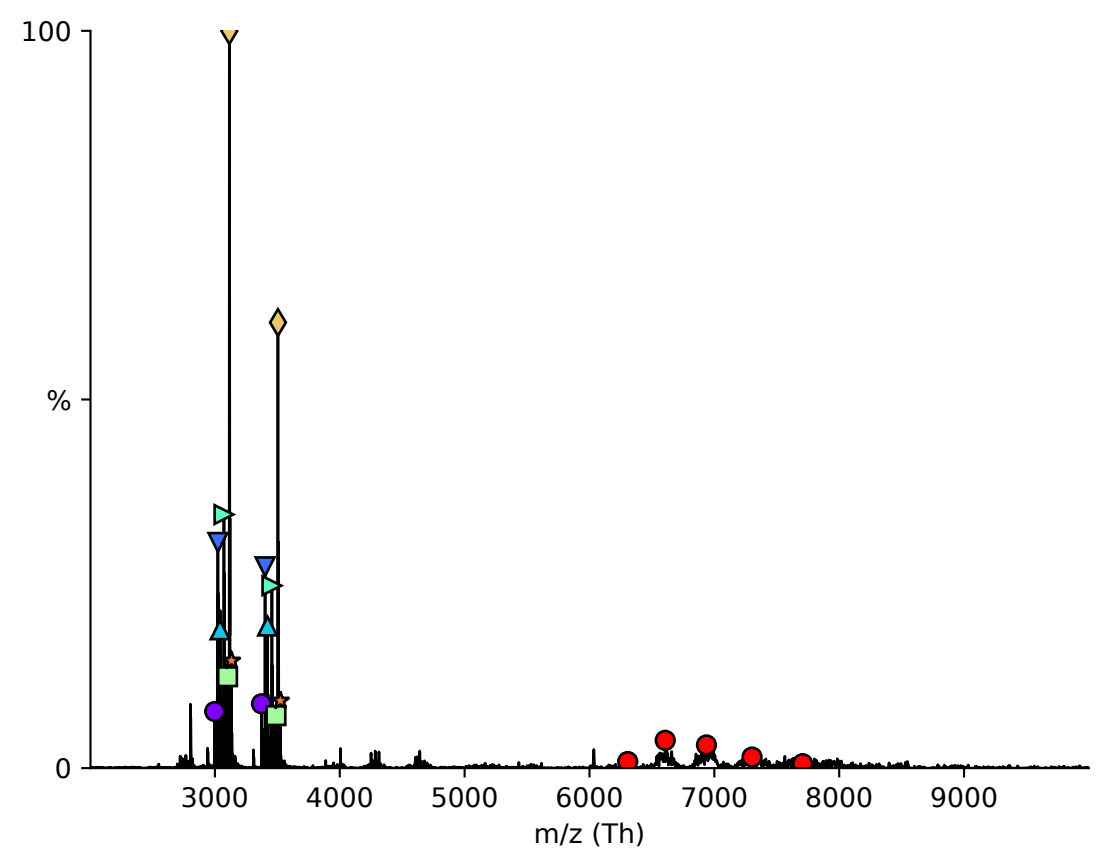

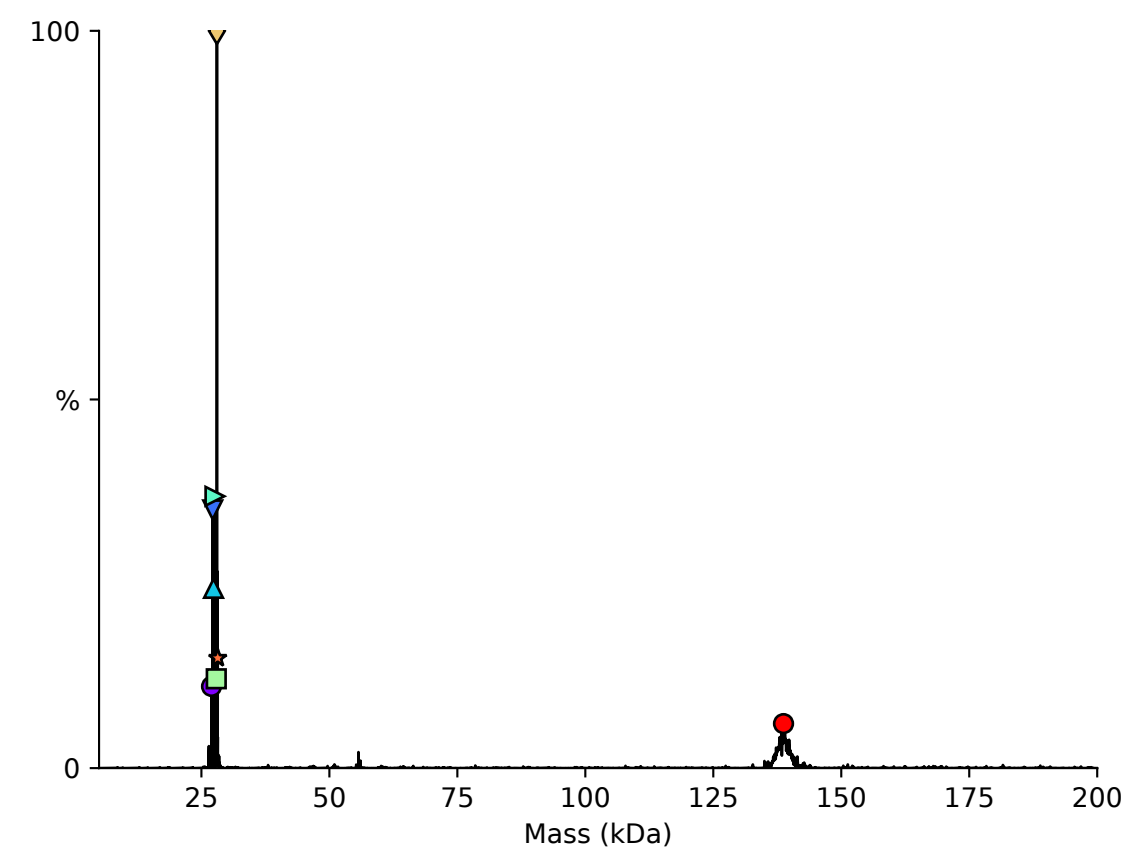

Symbol	Mass (Da)	Intensity[1]	Expected	Difference (Da)	Name/Species/Oligomer
○	26980	11	28043	−1063	
▽	27206	35	28043	−837	
△	27368	24	28043	−675	
▷	27645	37	28043	−398	
□	27902	12	28043	−141	
◊	28031	100	28043	−12	1[RK998]
☆	28188	15	28043	145	
○	138719	6	140215	−1496	

[1] Intensity values should not be considered quantitative

RTR16_C6

Laboratory: Wysocki-SC, SML
Client: Ryan K
Shipment Date:
Data Recorded: Mon, 11 Oct 2021, 16:58
Instrument: Q Exactive UHMR Orbitrap
Study: OBE 200 mM AmAc, RO33
Sample Conditions: 25mM Tris pH 8, 300mM NaCl
Concentration (ug/uL): 0.952
Injection (uL): 1.050420168
Comment: A couple different proteins in this one. No higher order species seen unfortunately
Date Analyzed: Mon, 18 Oct 2021, 12:58 Eastern Daylight Time
File Name: 20211007_RK999_6k_IST005_HCD005

Quality Check:
Average Peaks Score: 0.85

Expected Oligomers:
Passed:
Mixed:
Failed:

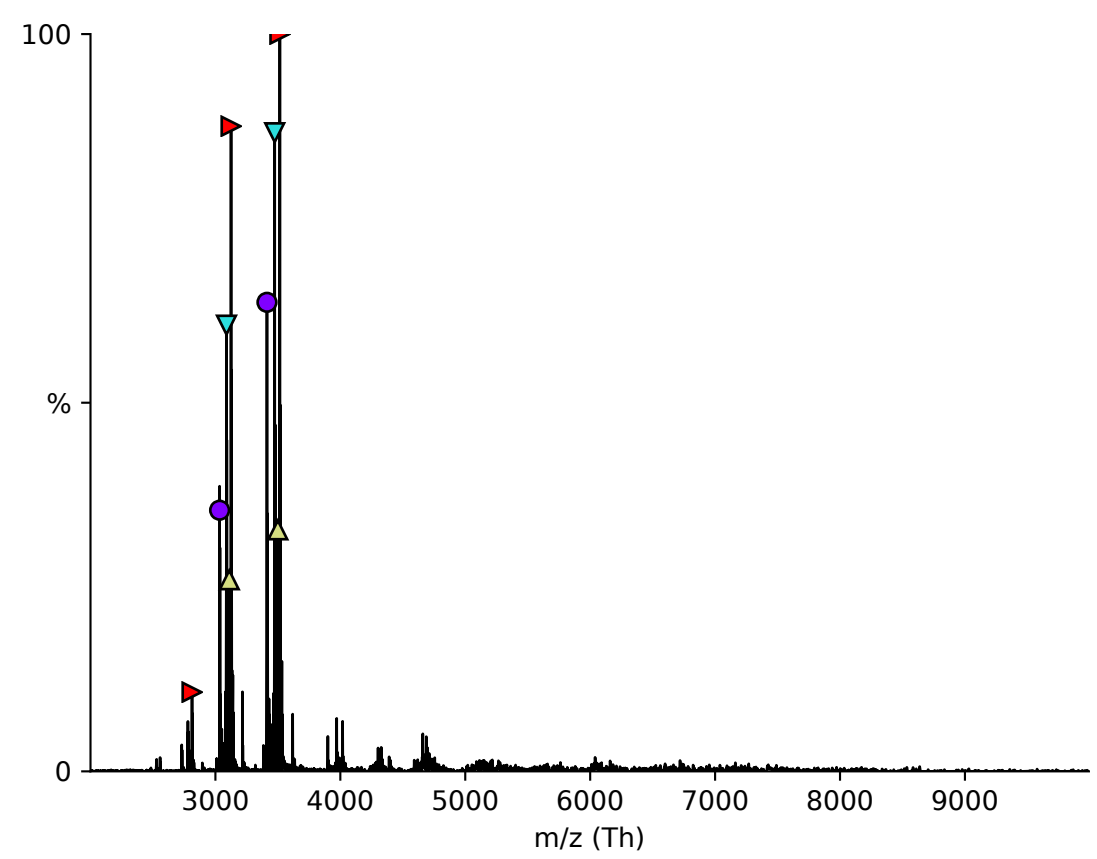

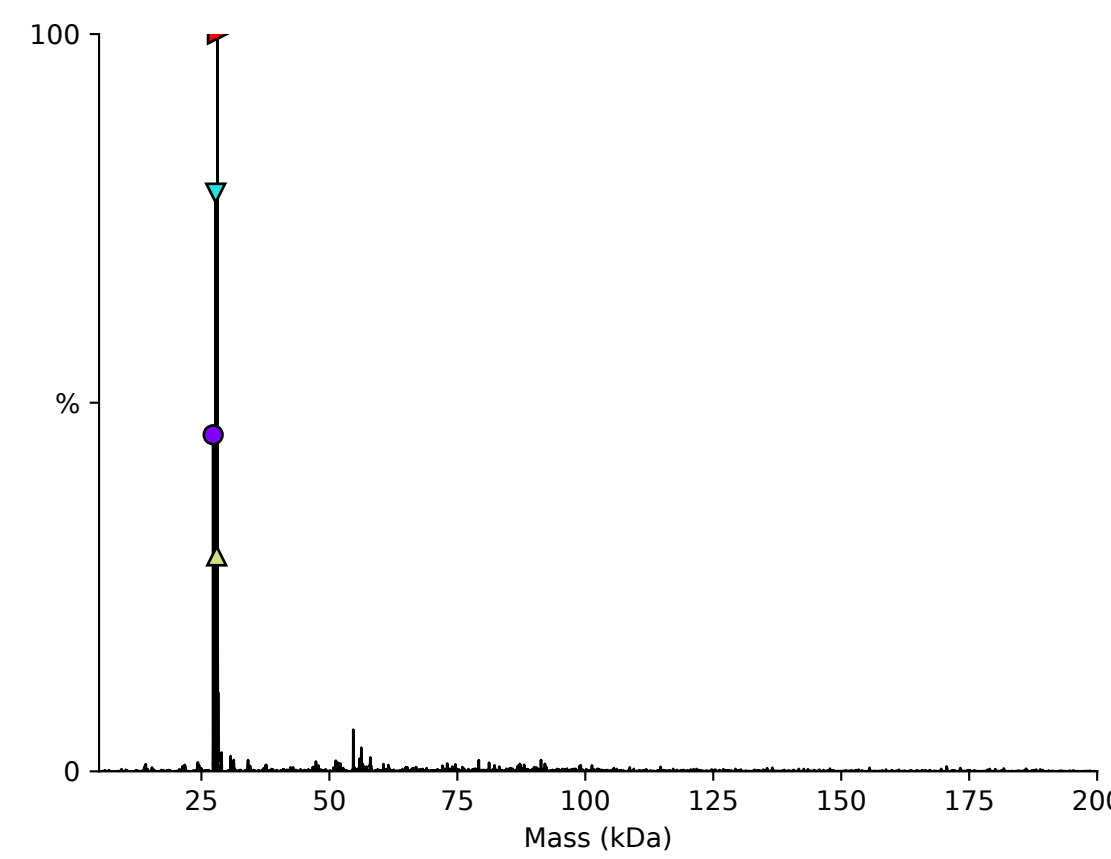

Symbol	Mass (Da)	Intensity[1]	Expected	Difference (Da)	Name/Species/Oligomer
○	27294	46	28129	−835	
▽	27789	78	28129	−340	
△	27989	29	28129	−140	
▷	28118	100	28129	−11	1[RK999]

[1]Intensity values should not be considered quantitative

RTR24noCys_C6

Laboratory: Wysocki-SC, SML
Client: Ryan K
Shipment Date:
Data Recorded: Mon, 11 Oct 2021, 16:58
Instrument: Q Exactive UHMR Orbitrap
Study: OBE 200 mM AmAc, RO33
Sample Conditions: 25mM Tris pH 8, 300mM NaCl
Concentration (ug/uL): 0.976214846
Injection (uL): 1.024364671
Comment: Some other proteins present. Found monomer and dimer
Date Analyzed: Mon, 18 Oct 2021, 13:03 Eastern Daylight Time
File Name: 20211007_RK1001_6k_IST060_HCD005

Quality Check:
Average Peaks Score: 0.85

Expected Oligomers:
Passed:
Mixed:
Failed:

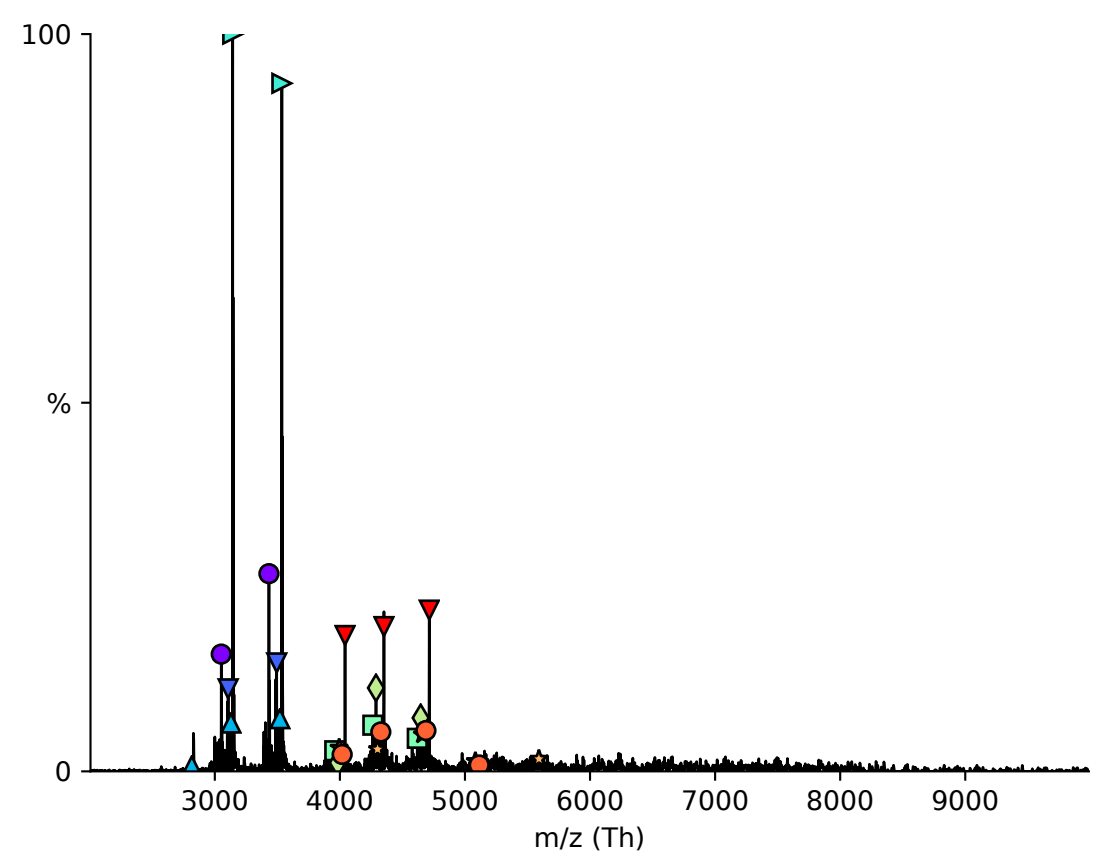

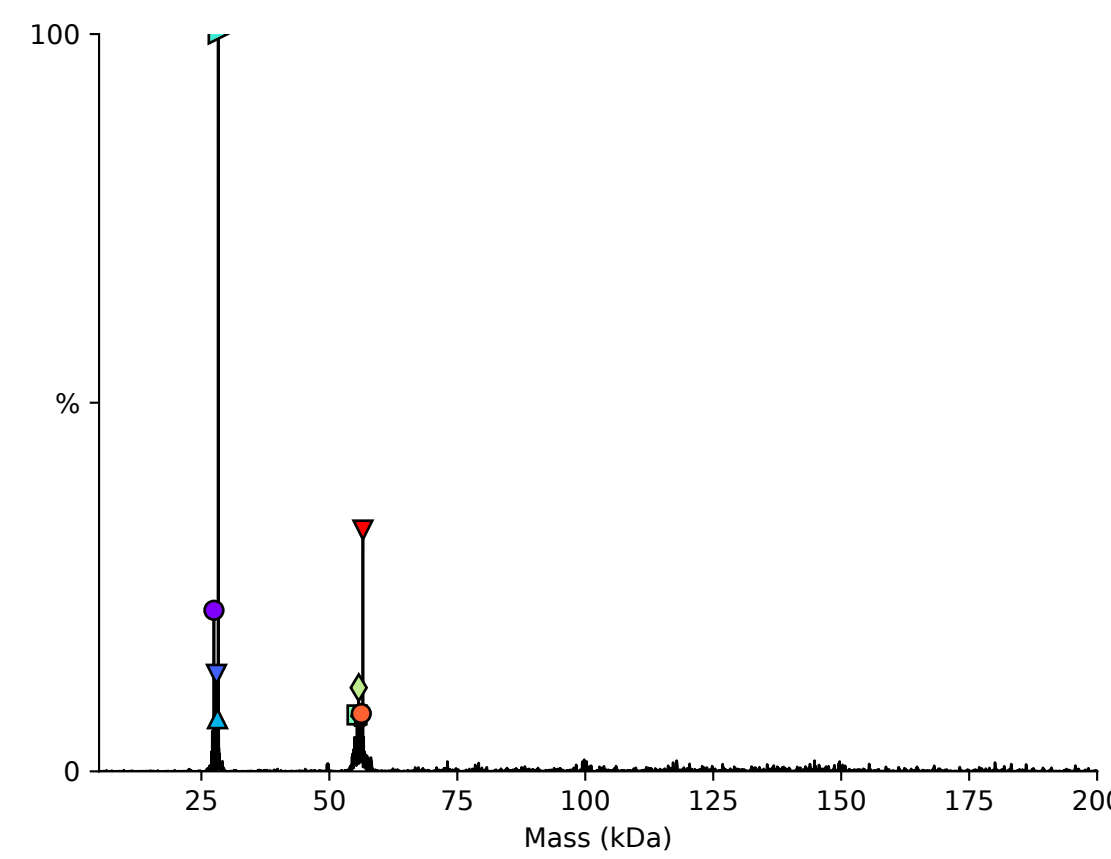

Symbol	Mass (Da)	Intensity[1]	Expected	Difference (Da)	Name/Species/Oligomer
○	27454	22	28291	−837	
▽	27951	13	28291	−340	
△	28151	7	28291	−140	
▷	28279	100	28291	−12	1[RK1001]
□	55413	8	56582	−1169	
◊	55739	11	56582	−843	
☆	55903	7	56582	−679	
○	56240	8	56582	−342	
▽	56563	33	56582	−19	2[RK1001]

[1] Intensity values should not be considered quantitative

RTR0_C8

Laboratory: Wysocki-NH
Client: Baker-Ryan
Shipment Date: 2021_01_13
Data Recorded: Fri, 15 Jan 2021, 05:11
Instrument: Q Exactive UHMR Orbitrap
Sample Conditions: 25mM Tris pH8, 300mM NaCl, 10mM EDTA
Study: OBE 200 mM AmAc
Injection (uL): 1.0
Comment: OBE P6
Date Analyzed: Mon, 25 Jan 2021, 08:50 Eastern Standard Time
File Name: 20210114_Ryan_RK655_6k_IST005_HCD060_OBE-P6

Quality Check:
Average Peaks Score: 0.7

Expected Oligomers:
Passed:
Mixed:
Failed:

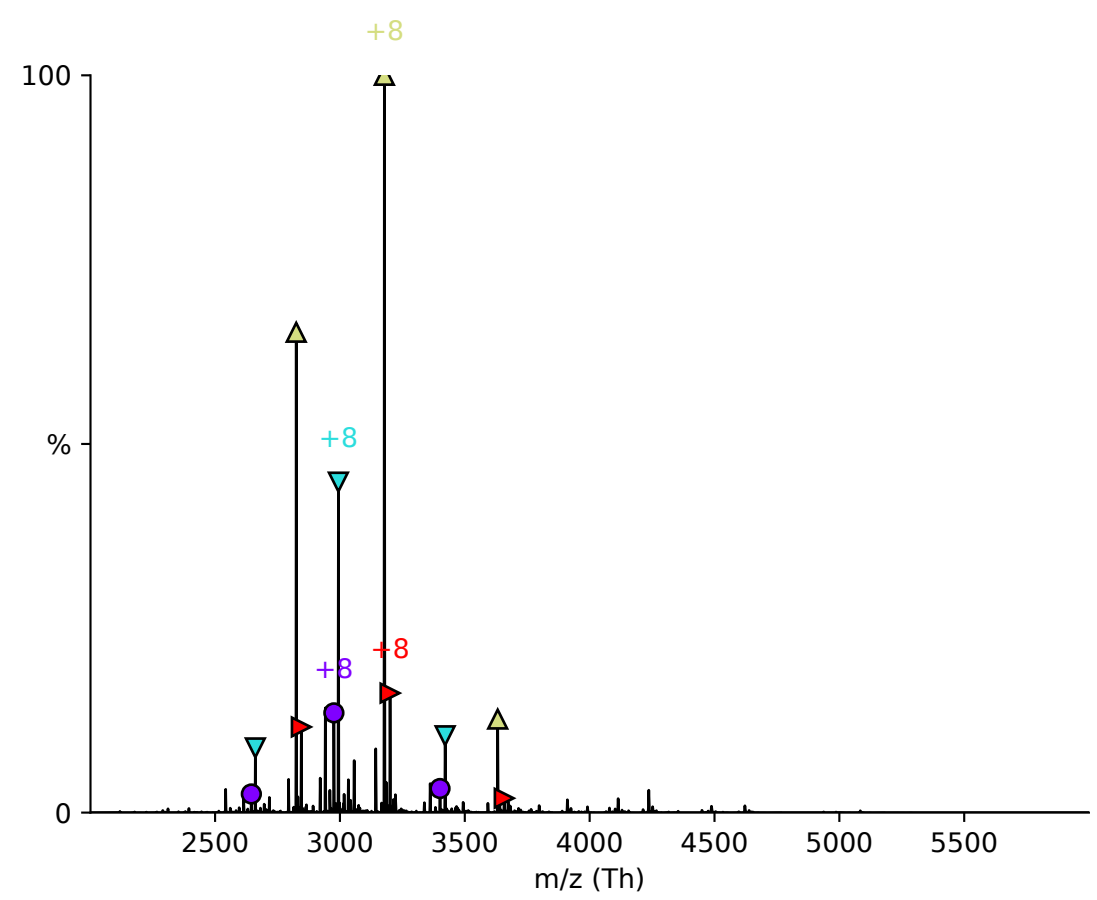

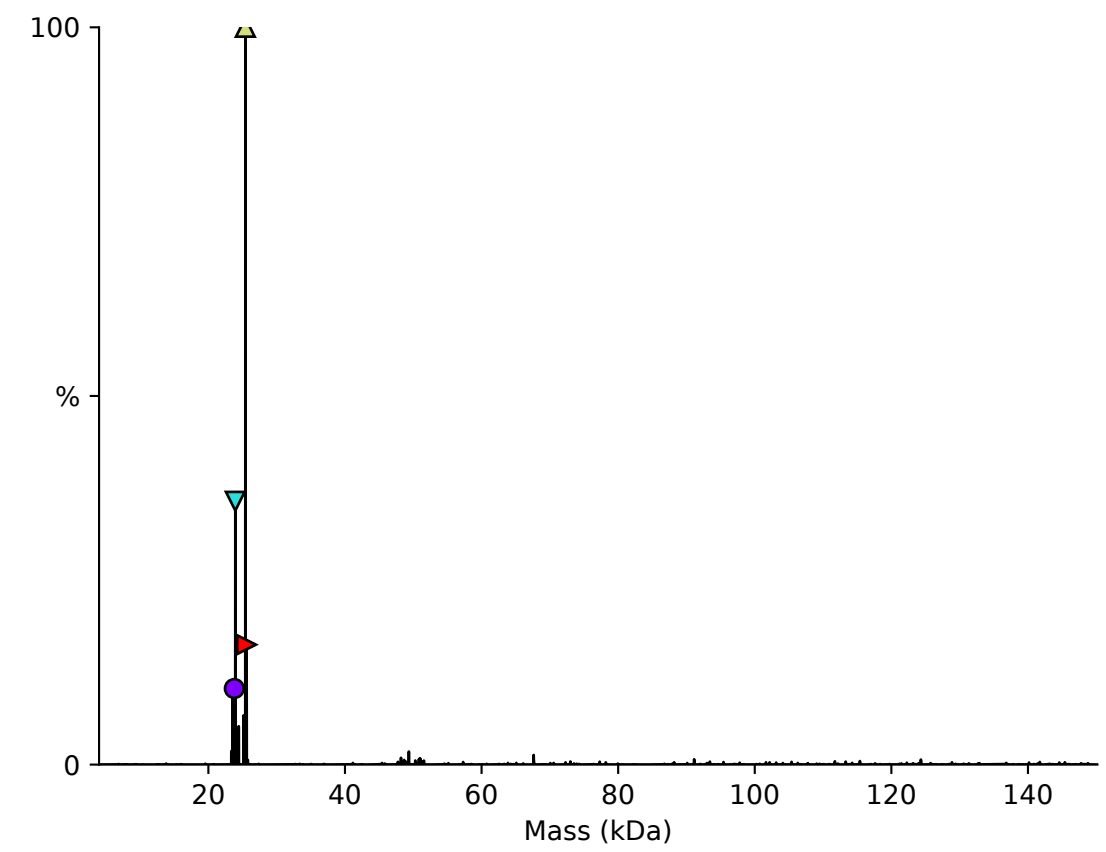

Symbol	Mass (Da)	Intensity[1]	Expected	Difference (Da)	Name/Species/Oligomer
◯	23798	10	25416	−1618	
▽	23942	36	25416	−1474	
△	25415	100	25416	−1	1[RK655]
▷	25598	16	25416	182	

[1]Intensity values should not be considered quantitative

RTR05_C8

Laboratory: Wysocki-SC, SML
Client: Ryan K
Shipment Date:
Data Recorded: Mon, 11 Oct 2021, 16:58
Instrument: Q Exactive UHMR Orbitrap
Study: OBE 200 mM AmAc, RO33
Sample Conditions: 25mM Tris pH 8, 300mM NaCl
Concentration (ug/uL): 0.985
Injection (uL): 1.015228426
Comment: Mostly monomer. There might be a small amount of octamer. The high mass species is pretty close
Date Analyzed: Mon, 18 Oct 2021, 13:25 Eastern Daylight Time
File Name: 20211007_RK1002_6k_IST060_HCD005

Quality Check:
Average Peaks Score: 0.92

Expected Oligomers:
Passed:
Mixed:
Failed:

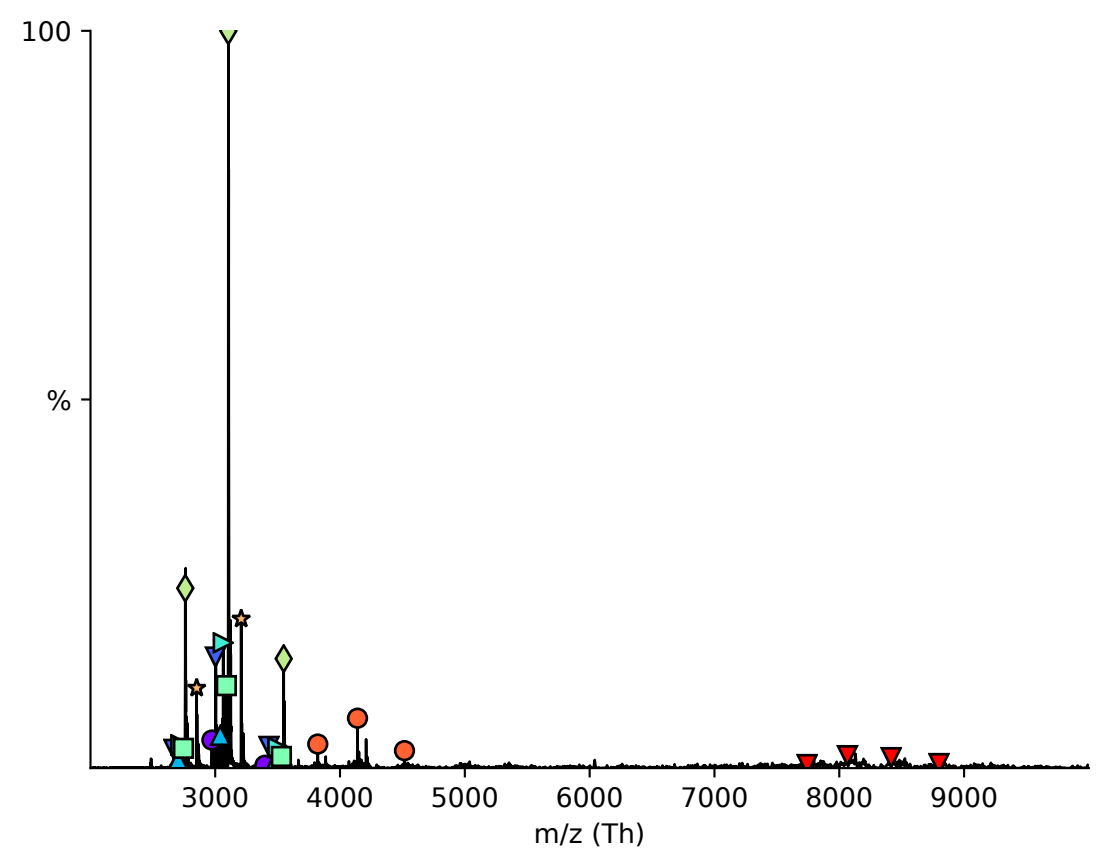

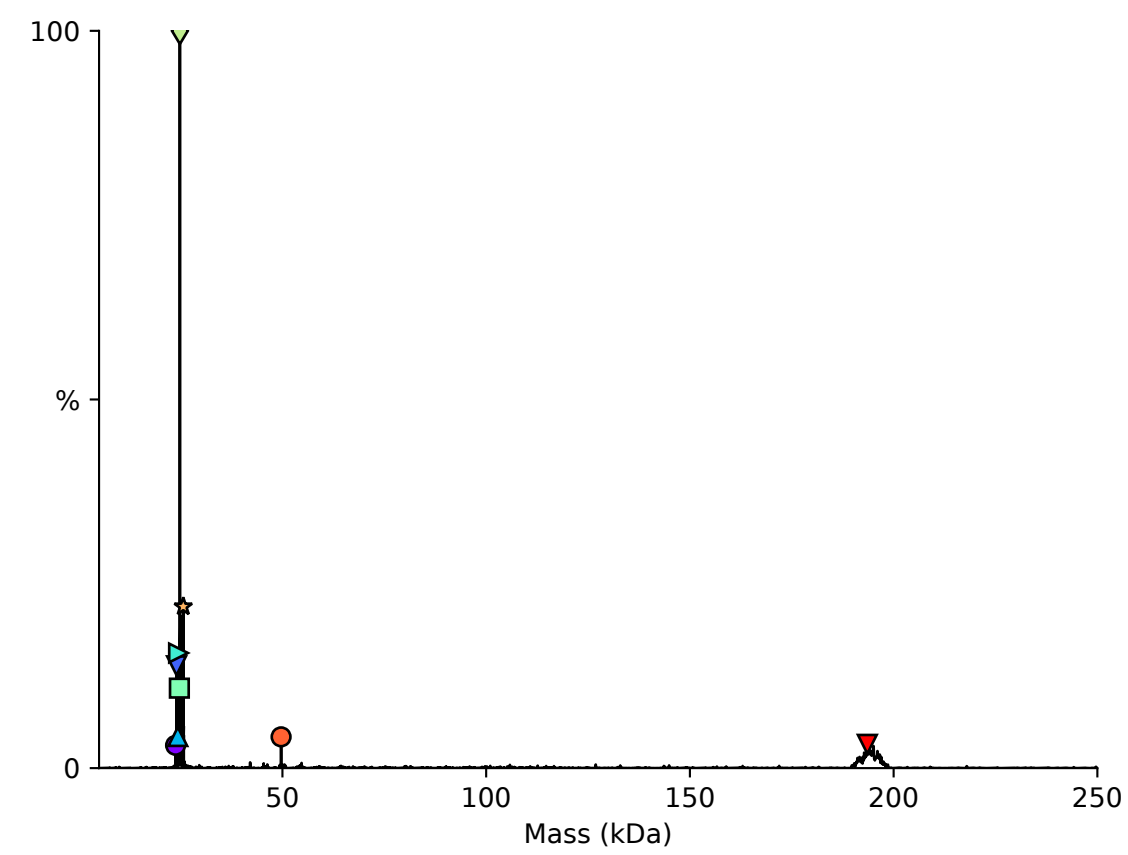

Symbol	Mass (Da)	Intensity[1]	Expected	Difference (Da)	Name/Species/Oligomer
○	23782	3	24845	−1063	
▽	24009	14	24845	−836	
△	24320	4	24845	−525	
▷	24505	16	24845	−340	
□	24705	11	24845	−140	
◊	24834	100	24845	−11	1[RK1002]
☆	25657	22	24845	812	
○	49671	4	49690	−19	2[RK1002]
▽	193545	3	198760	−5215	

[1]Intensity values should not be considered quantitative

RTR11noCys_C8

Laboratory: Wysocki-SC, SML
Client: Ryan K
Shipment Date:
Data Recorded: Mon, 11 Oct 2021, 16:58
Instrument: Q Exactive UHMR Orbitrap
Study: OBE 200 mM AmAc, RO33
Sample Conditions: 25mM Tris pH 8, 300mM NaCl
Concentration (ug/uL): 0.973
Injection (uL): 1.027749229
Comment: Monomer and hexamer are present in this spectrum. No octamer unfortunately.
Date Analyzed: Mon, 18 Oct 2021, 13:22 Eastern Daylight Time
File Name: 20211007_RK1003_6k_IST060_HCD005

Quality Check:
Average Peaks Score: 0.85

Expected Oligomers:
Passed:
Mixed:
Failed:

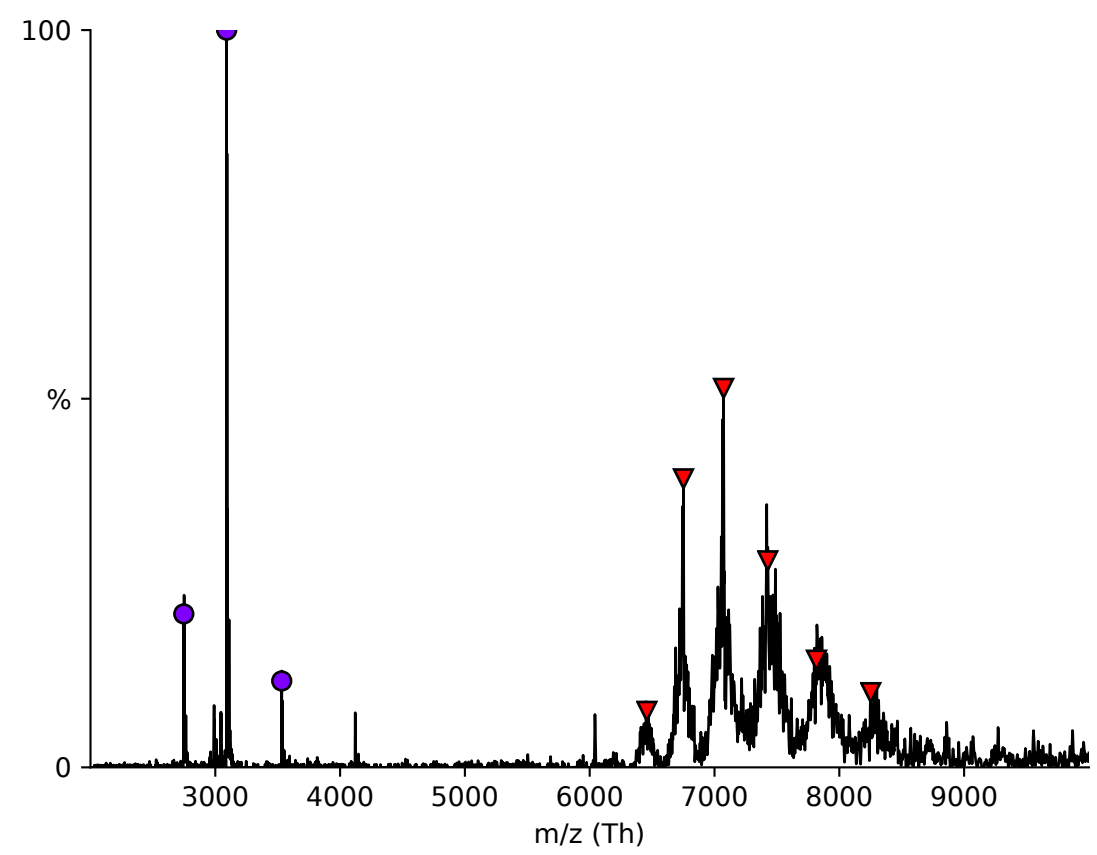

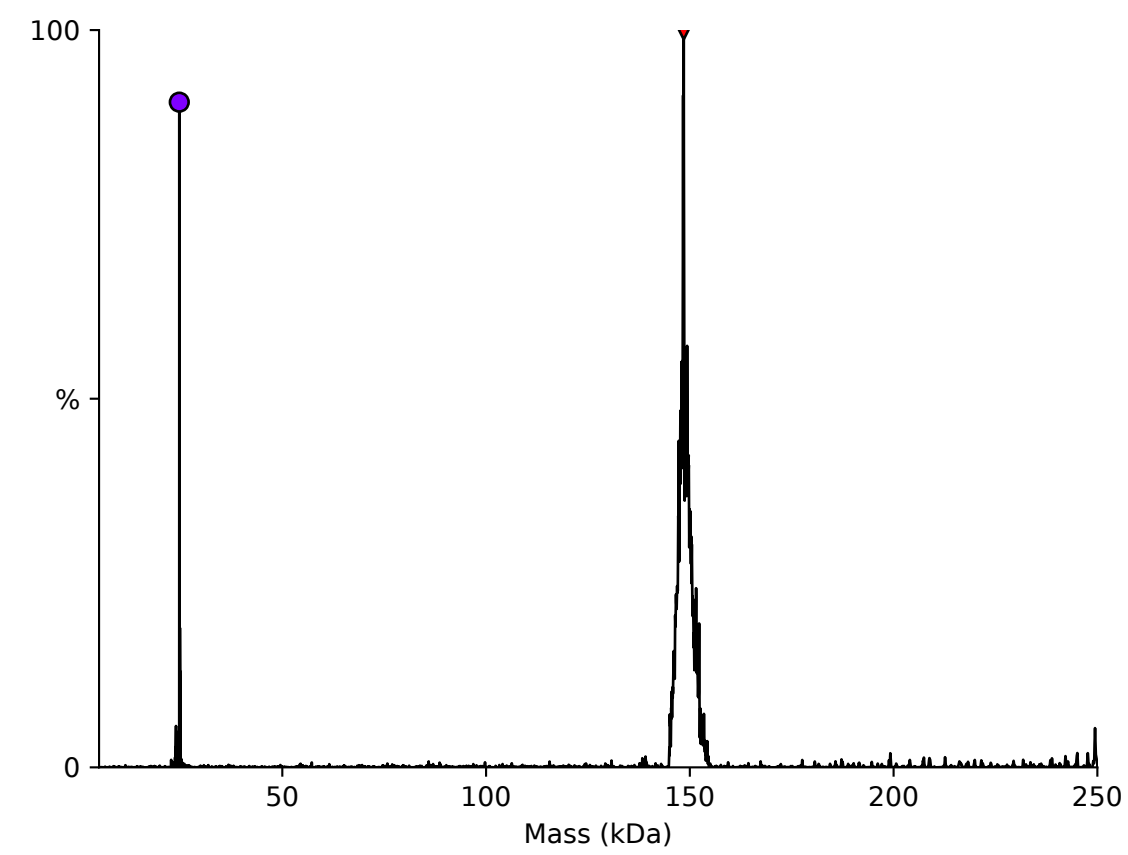

Symbol	Mass (Da)	Intensity[1]	Expected	Difference (Da)	Name/Species/Oligomer
○	24719	90	24730	−11	1[RK1003]
▽	148506	100	148380	126	6[RK1003]

[1]Intensity values should not be considered quantitative

RTR16_C8

Laboratory: Wysocki-SC, SML
Client: Ryan K
Shipment Date:
Data Recorded: Mon, 11 Oct 2021, 16:58
Instrument: Q Exactive UHMR Orbitrap
Study: OBE 200 mM AmAc, RO33
Sample Conditions: 25mM Tris pH 8, 300mM NaCl
Concentration (ug/uL): 1.0
Injection (uL): 1.0
Comment: Lots of proteins in this spectrum. Main protein was found, but no higher order oligomers found unfortunately
Date Analyzed: Mon, 18 Oct 2021, 13:40 Eastern Daylight Time
File Name: 20211007_RK1004_6k_IST060_HCD005

Quality Check:
Average Peaks Score: 0.89

Expected Oligomers:
Passed:
Mixed:
Failed:

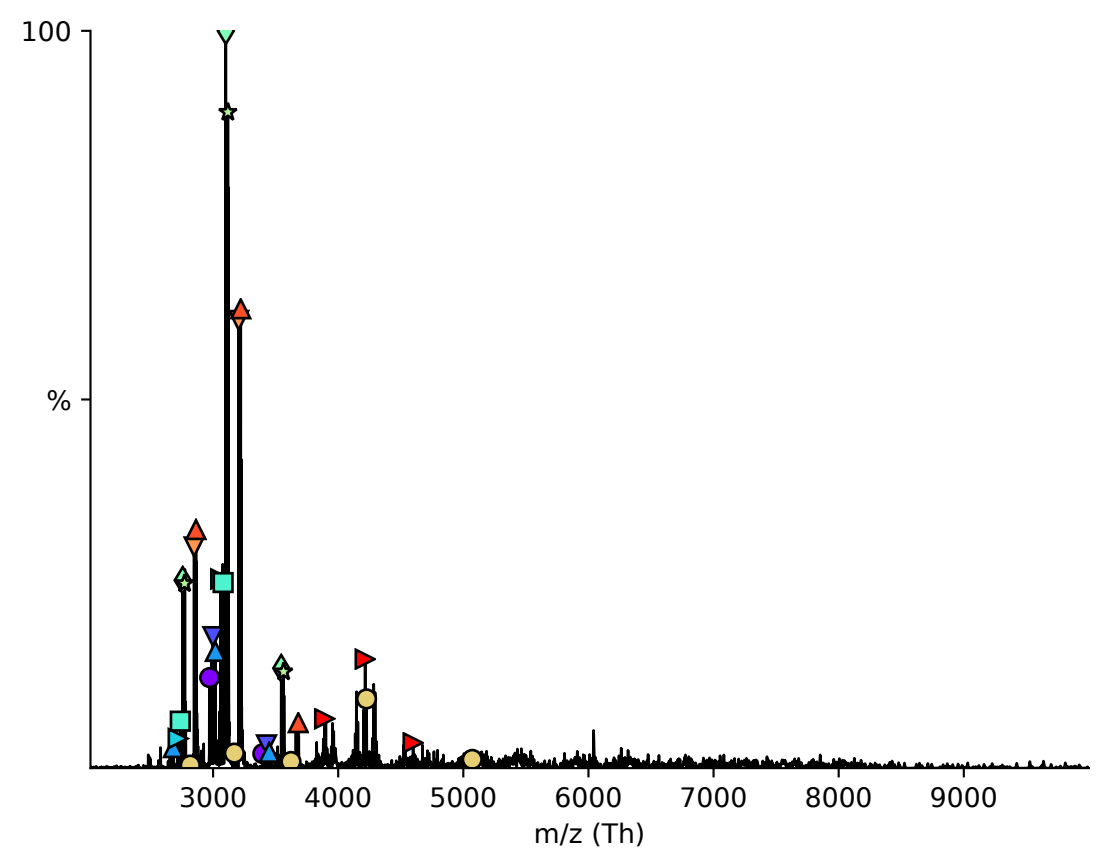

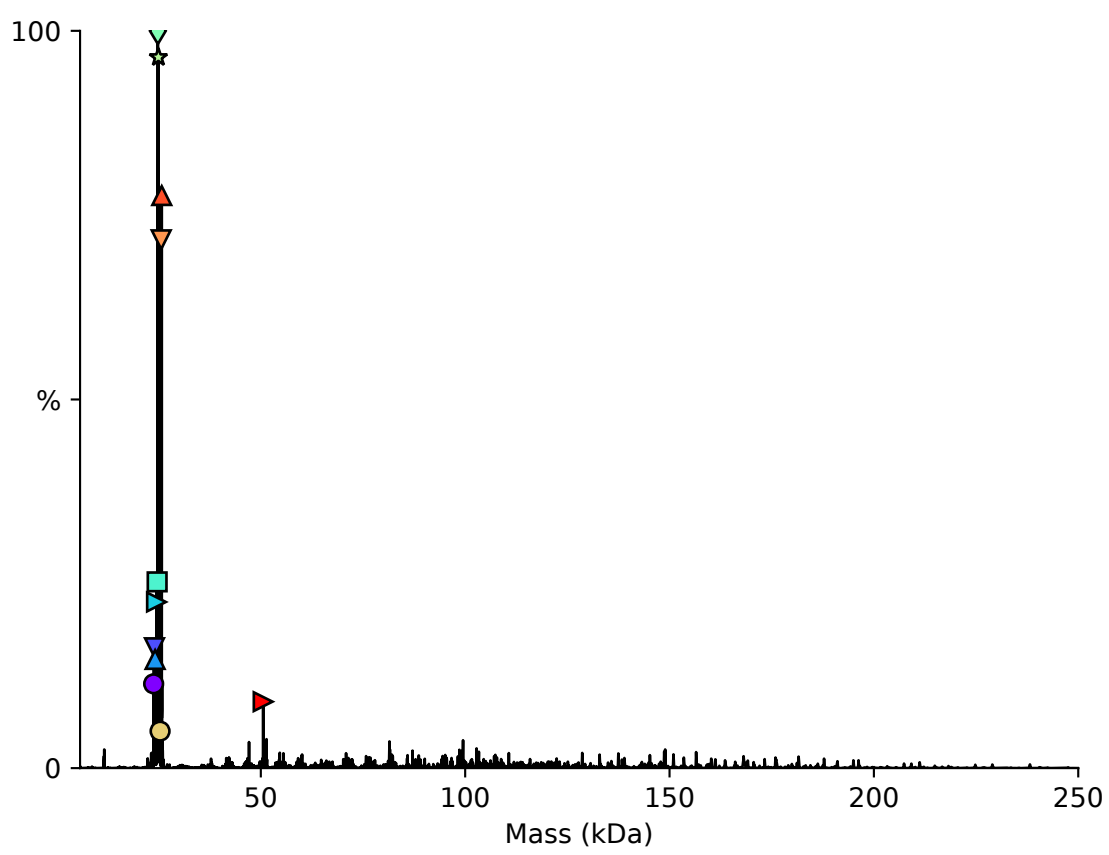

Symbol	Mass (Da)	Intensity[1]	Expected	Difference (Da)	Name/Species/Oligomer
◯	23777	11	25771	−1994	
▽	23982	16	25771	−1789	
△	24137	15	25771	−1634	
▷	24478	23	25771	−1293	
□	24633	25	25771	−1138	
◊	24806	100	25771	−965	
☆	24937	96	25771	−834	
◯	25351	5	25771	−420	
▽	25629	72	25771	−142	
△	25760	78	25771	−11	1[RK1004]
▷	50573	9	51542	−969	

[1] Intensity values should not be considered quantitative

RTR24noCys_C8

Laboratory: Wysocki-SC, SML
Client: Ryan K
Shipment Date:
Data Recorded: Mon, 11 Oct 2021, 16:58
Instrument: Q Exactive UHMR Orbitrap
Study: OBE 200 mM AmAc, RO33
Sample Conditions: 25mM Tris pH 8, 300mM NaCl
Concentration (ug/uL): 1.0
Injection (uL): 1.0
Comment: Pretty messy spectrum, but I was able to pull monomer and dimer out of it.
Date Analyzed: Mon, 18 Oct 2021, 14:24 Eastern Daylight Time
File Name: 20211007_RK1006_6k_IST060_HCD005

Quality Check:
Average Peaks Score: 0.81

Expected Oligomers:
Passed:
Mixed:
Failed:

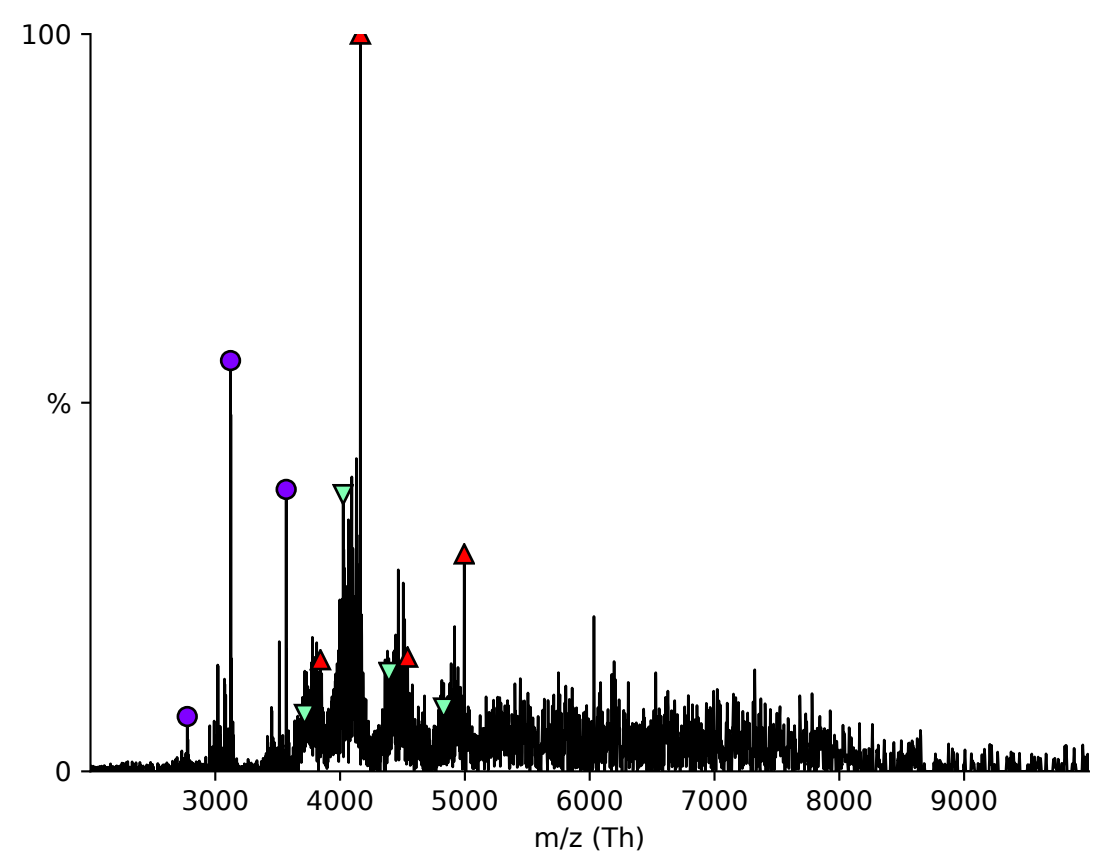

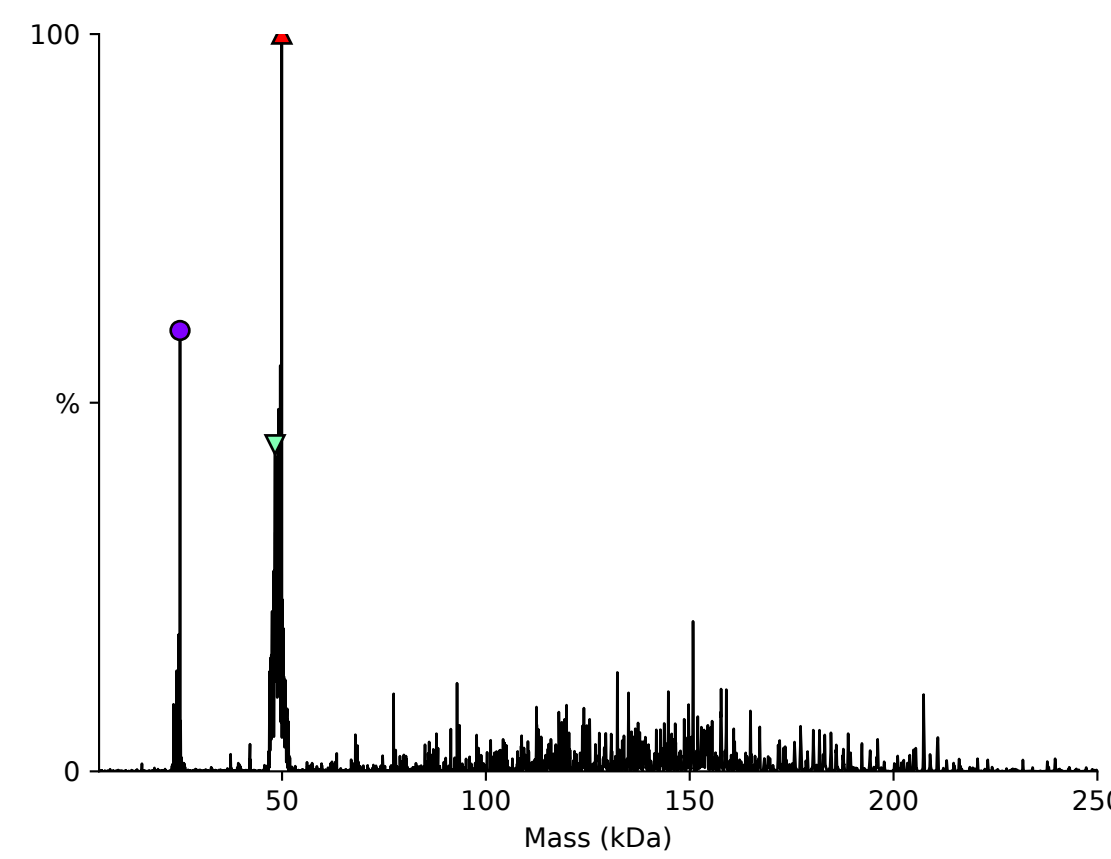

Symbol	Mass (Da)	Intensity[1]	Expected	Difference (Da)	Name/Species/Oligomer
○	24968	60	24978	−10	1[RK1006]
▽	48287	44	49956	−1669	
△	49938	100	49956	−18	2[RK1006]

[1]Intensity values should not be considered quantitative

RTR0_C12

Laboratory: Wysocki-NH
Client: Baker-Ryan
Shipment Date: 2021_01_13
Data Recorded: Fri, 15 Jan 2021, 05:22
Instrument: Q Exactive UHMR Orbitrap
Sample Conditions: 25mM Tris pH8, 300mM NaCl, 10mM EDTA
Study: OBE 200 mM AmAc
Injection (uL): 1.0
Comment: OBE P6
Date Analyzed: Mon, 25 Jan 2021, 08:51 Eastern Standard Time
File Name: 20210114_Ryan_RK656_6k_IST005_HCD060_OBE-P6

Quality Check:
Average Peaks Score: 0.76

Expected Oligomers:
Passed:
Mixed:
Failed:

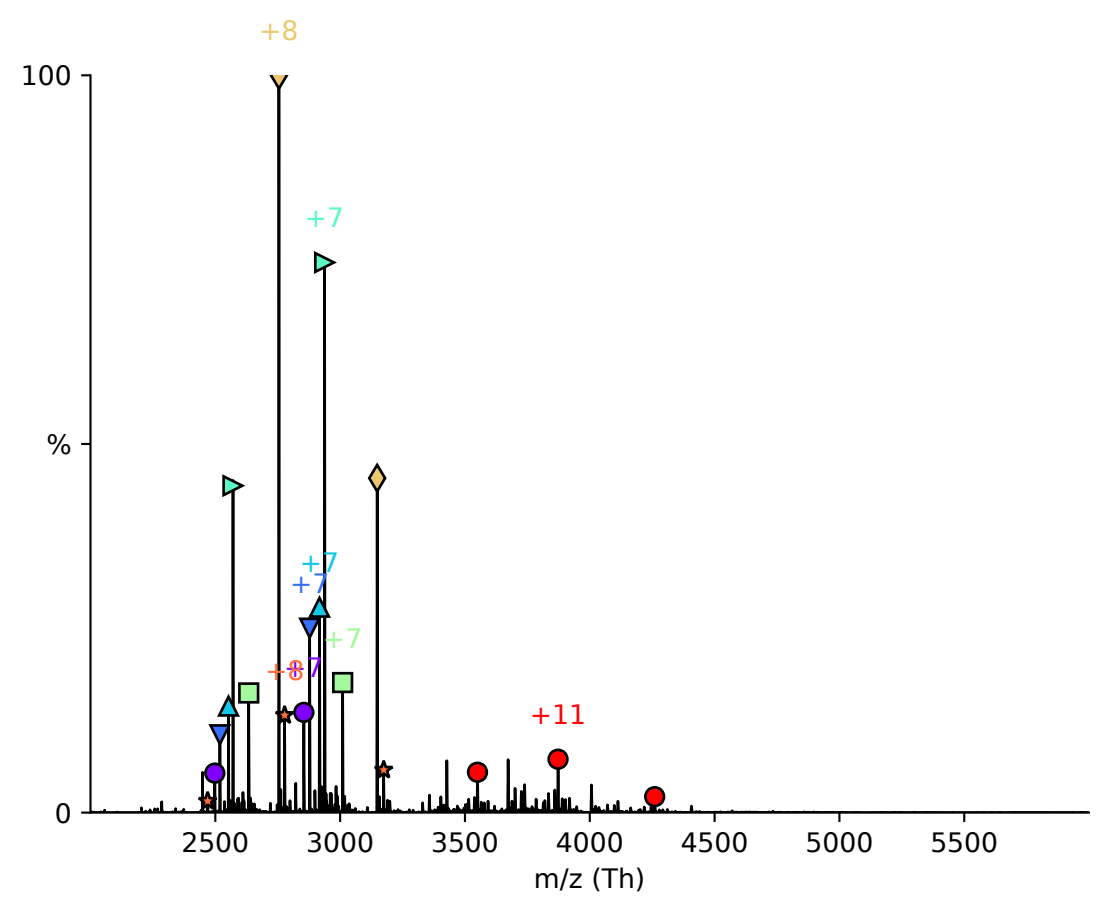

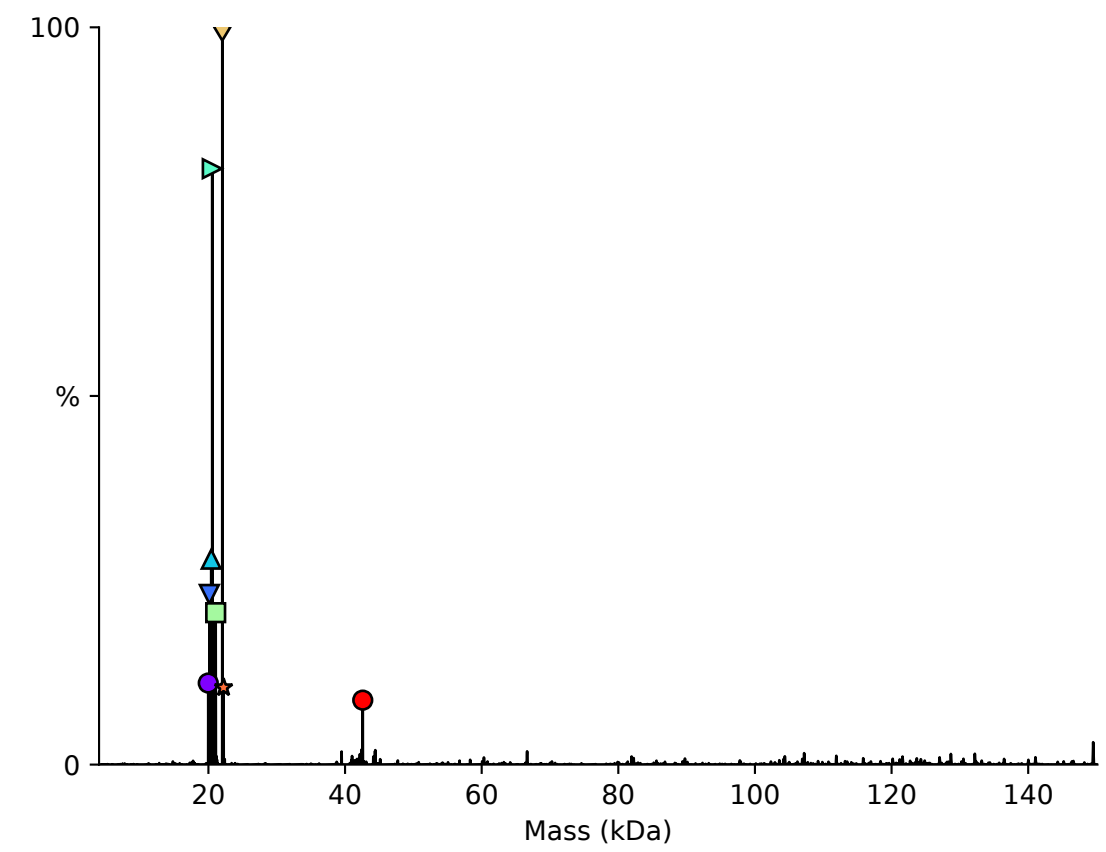

Symbol	Mass (Da)	Intensity[1]	Expected	Difference (Da)	Name/Species/Oligomer
○	19976	11	22032	−2056	
▽	20139	23	22032	−1893	
△	20414	28	22032	−1618	
▷	20558	81	22032	−1474	
□	21063	21	22032	−969	
◊	22031	100	22032	−1	1[RK656]
☆	22214	10	22032	182	
○	42590	9	44064	−1474	

[1] Intensity values should not be considered quantitative

RTR11noCys_C12

Laboratory: Wysocki-SC, SML
Client: Ryan K
Shipment Date:
Data Recorded: Mon, 11 Oct 2021, 16:58
Instrument: Q Exactive UHMR Orbitrap
Study: OBE 200 mM AmAc, RO33
Sample Conditions: 25mM Tris pH 8, 300mM NaCl
Concentration (ug/uL): 0.785868368
Injection (uL): 1.272477734
Comment: Looks closer to decamer instead of dodecamer
Date Analyzed: Mon, 18 Oct 2021, 12:42 Eastern Daylight Time
File Name: 20211007_RK958_6k_IST060_HCD005

Quality Check:
Average Peaks Score: 0.69

Expected Oligomers:
Passed:
Mixed:
Failed:

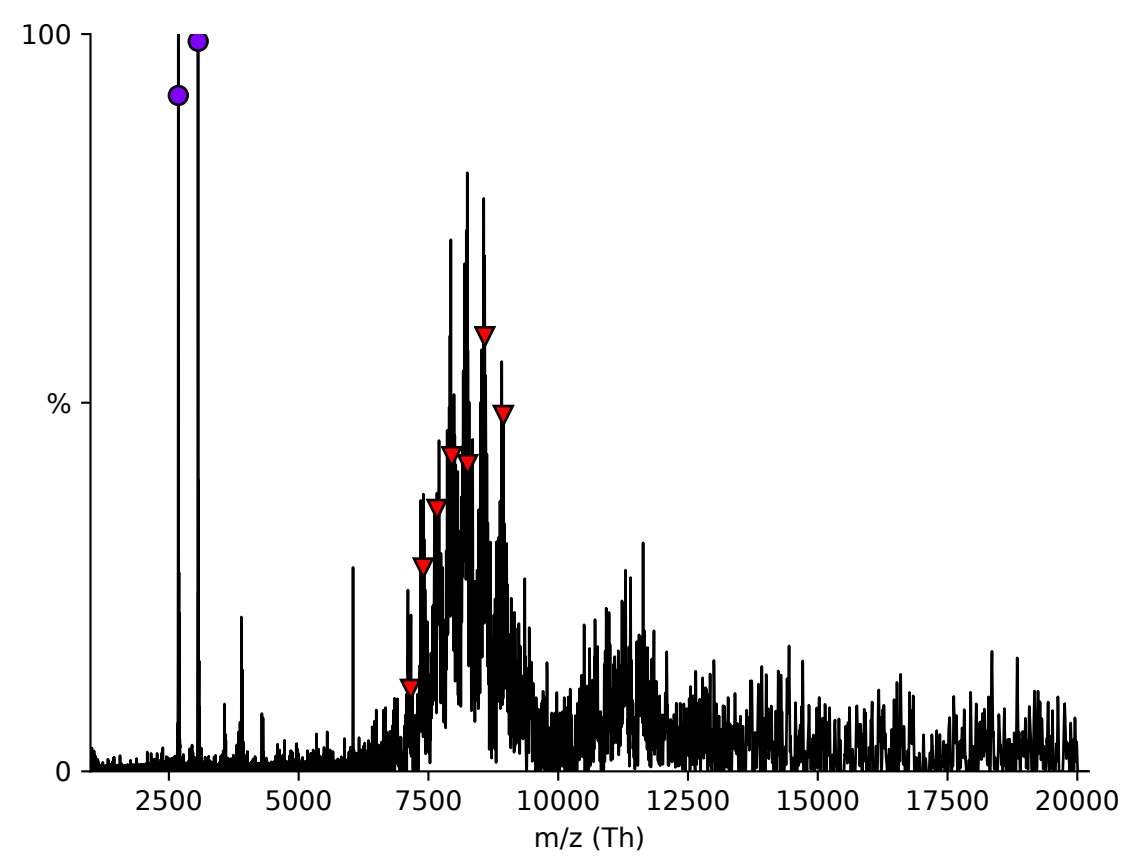

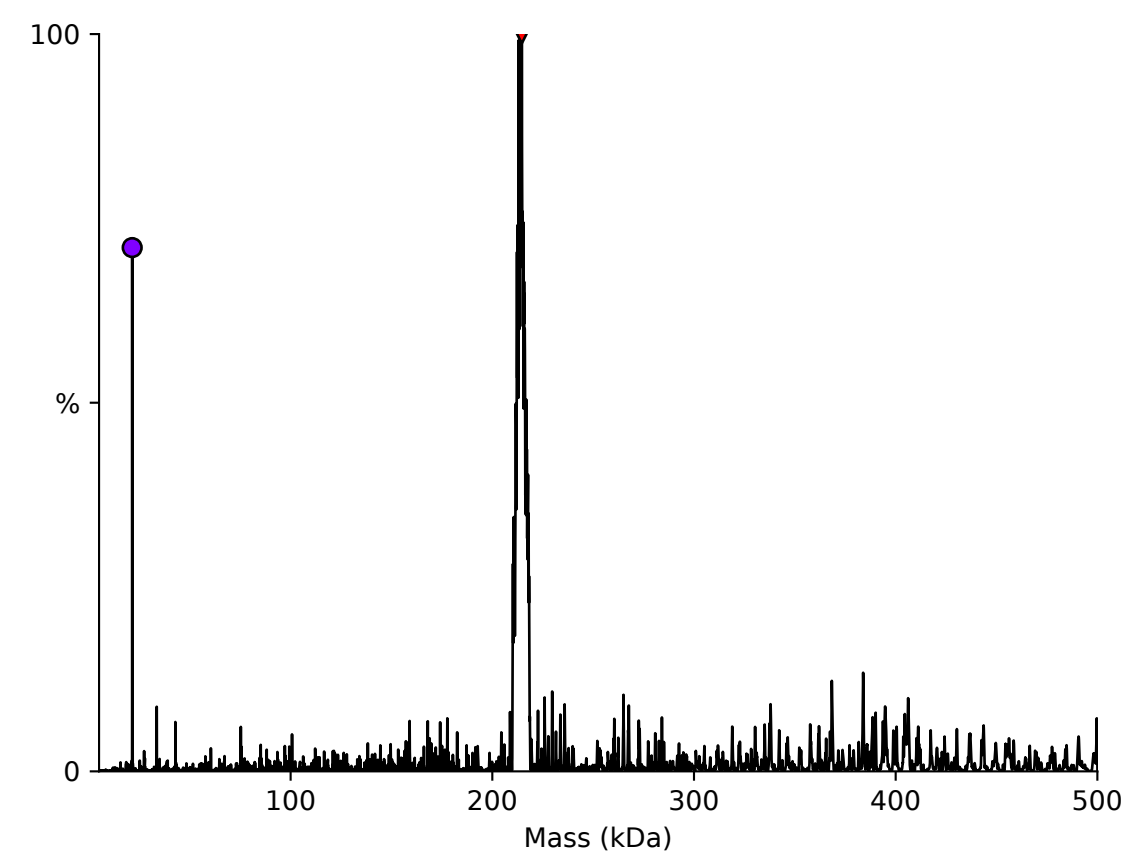

Symbol	Mass (Da)	Intensity[1]	Expected	Difference (Da)	Name/Species/Oligomer
○	21445	71	21432	13	1[RK958]
▽	214624	100	214320	304	10[RK958]

[1]Intensity values should not be considered quantitative

RTR24noCys_C12

Laboratory: Wysocki-NH
Client: Ryan Kibler
Shipment Date:
Data Recorded: Thu, 19 Aug 2021, 22:58
Instrument: Q Exactive UHMR Orbitrap
Study: OBE 200 mM AmAc, RO33
Sample Conditions: 25mM Tris, 300mM NaCl, pH 8
Concentration (ug/uL): 1.0
Injection (uL): 1.0
Comment: Only monomer and dimer found here
Date Analyzed: Tue, 31 Aug 2021, 12:19 Eastern Daylight Time
File Name: 20210819_RK960_6k_ist_005_HCD_005

Quality Check:
Average Peaks Score: 0.55

Expected Oligomers:
Passed:
Mixed:
Failed:

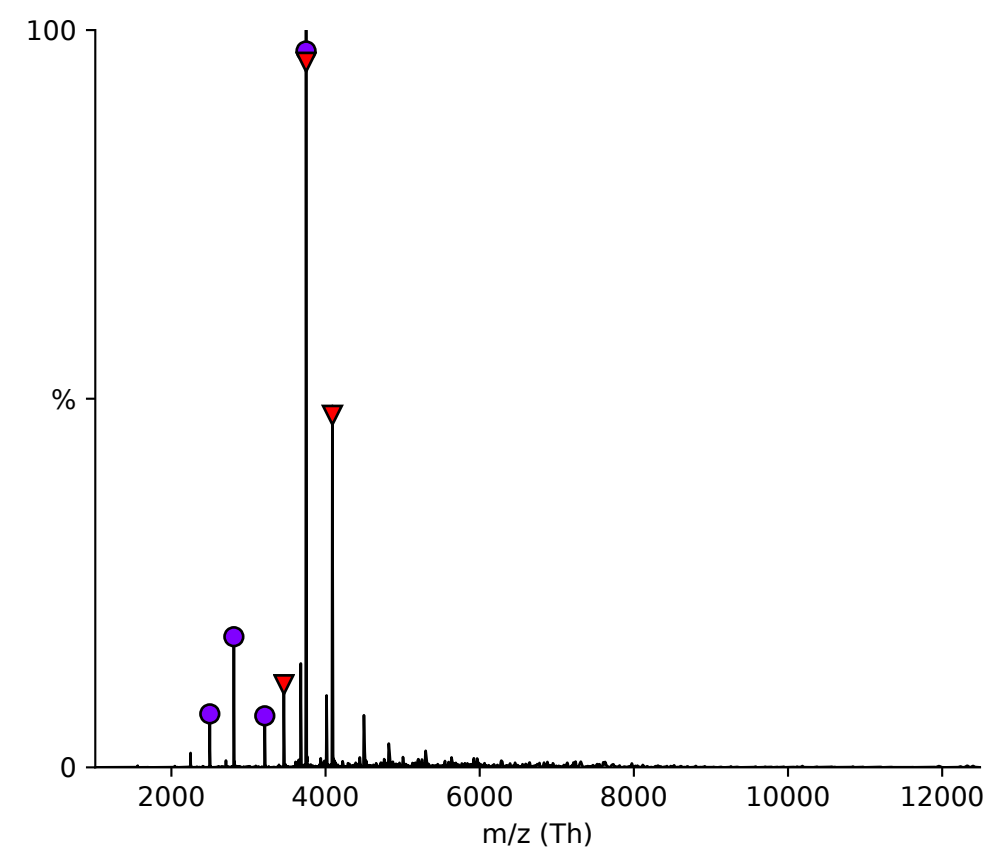

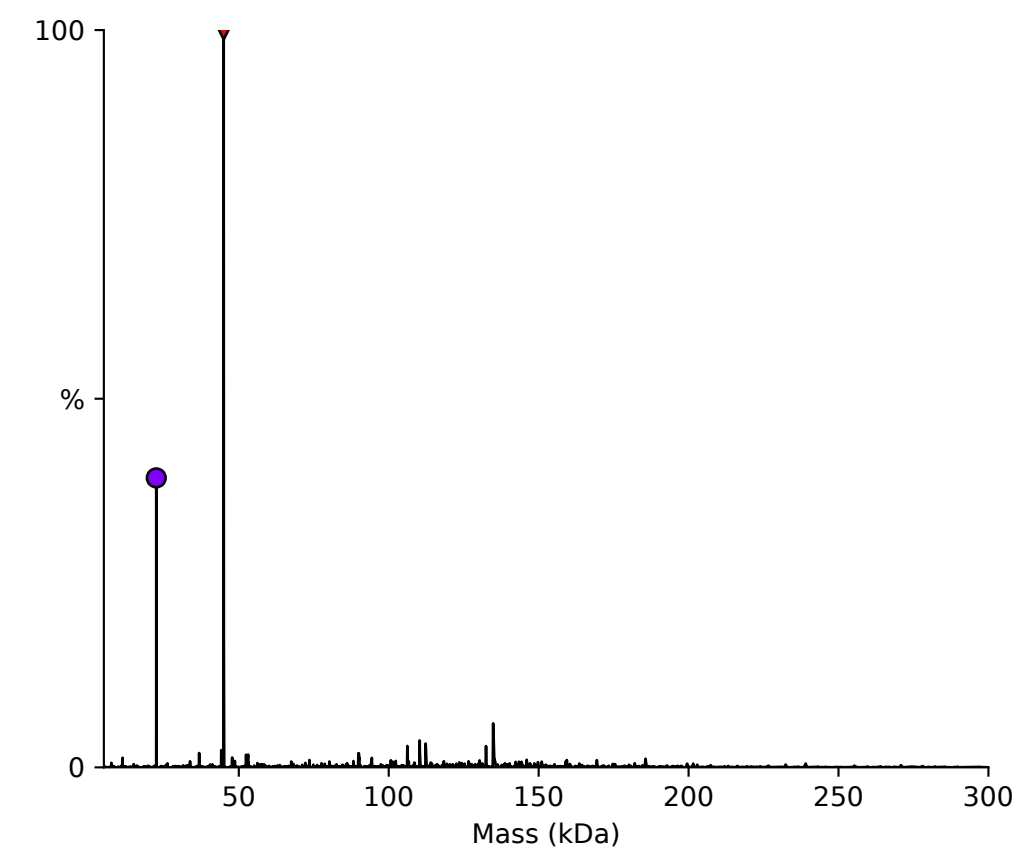

Symbol	Mass (Da)	Intensity[1]	Expected	Difference (Da)	Name/Species/Oligomer
○	22475	39	22610	−135	1[A]
▽	44968	100	45220	−252	2[A]

[1]Intensity values should not be considered quantitative

A2B2-hetRTR05_11

Laboratory: Wysocki-NH
Client: Baker-Ryan
Shipment Date: 2021_06_30
Data Recorded: Fri, 02 Jul 2021, 13:20
Instrument: Q Exactive UHMR Orbitrap
Study: OBE 200 mM AmAc, RO22
Sample Conditions: 25mM Tris pH8, 300mM NaCl
Concentration (ug/uL): 1.0
Injection (uL): 1.0
Comment:
Date Analyzed: Fri, 16 Jul 2021, 11:28 Eastern Daylight Time
File Name: 20210701_Ryan_RK845_IST200_6k

Quality Check:
Average Peaks Score: 0.78

Expected Oligomers:
Passed:
Mixed:
Failed:

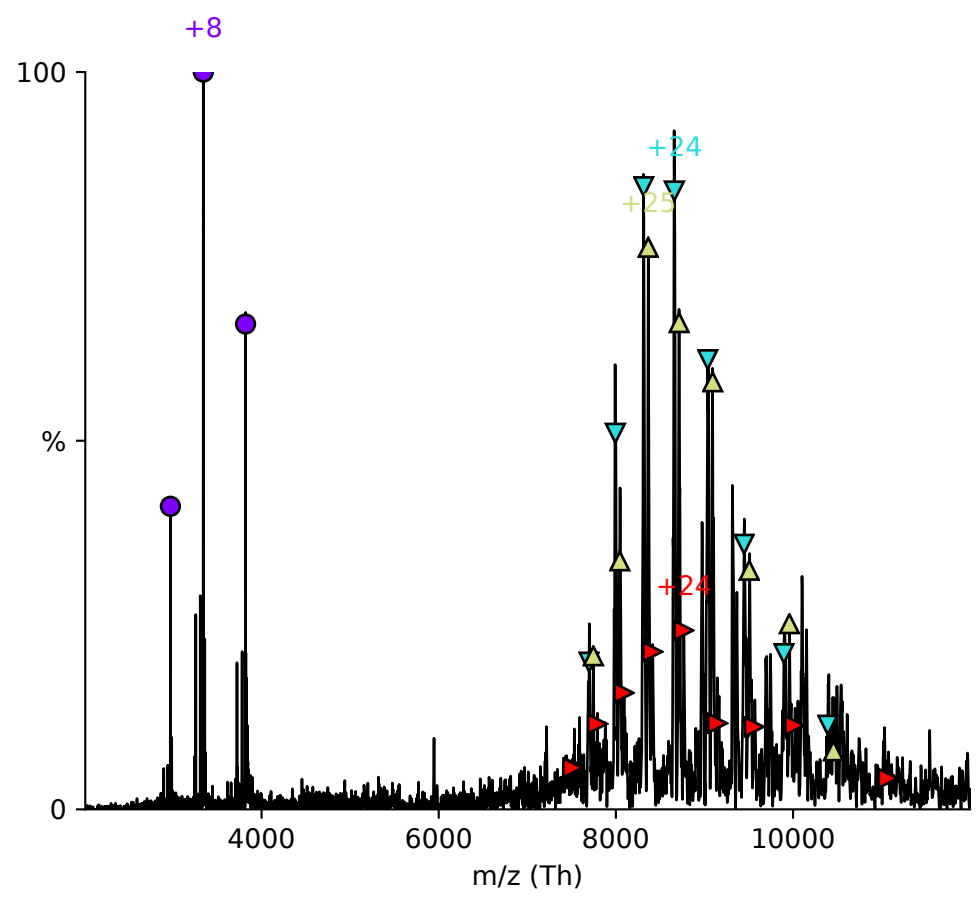

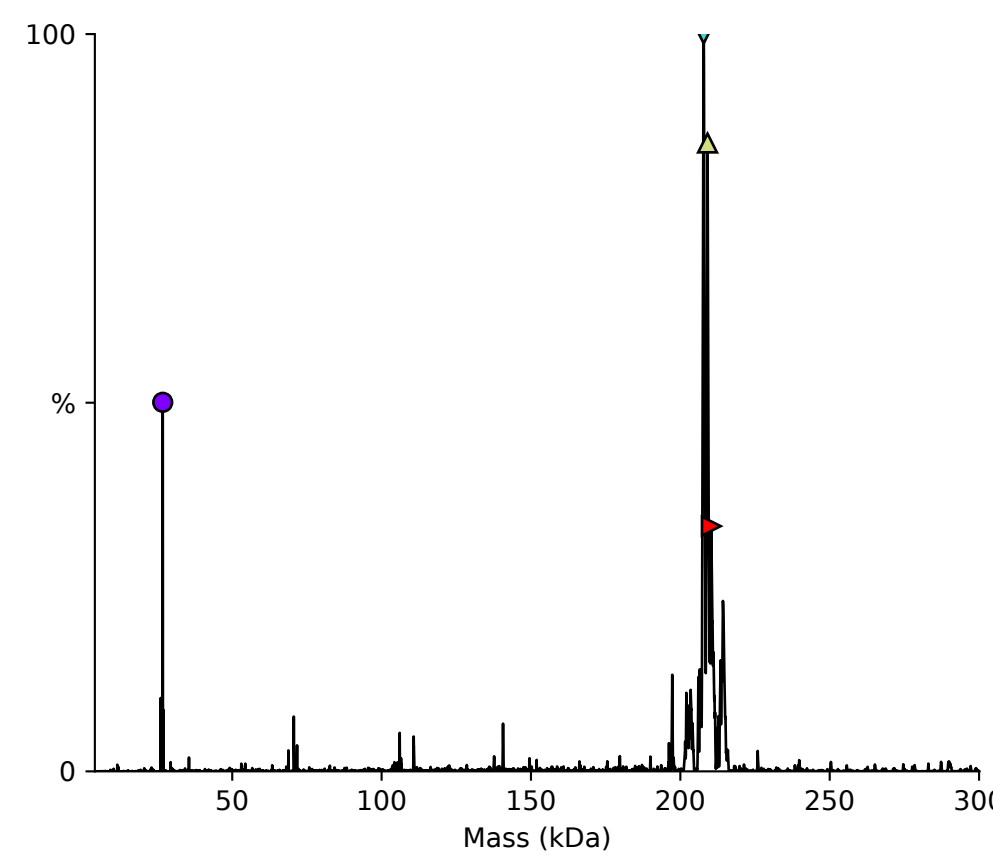

Symbol	Mass (Da)	Intensity[1]	Expected	Difference (Da)	Name/Species/Oligomer
○	26729	50	35927	−9198	
▽	207814	100	207827	−13	3[RK845_A]
△	209106	85	207827	1279	
▷	210375	33	210406	−31	2[RK845_A] 2[RK845_B]

[1]Intensity values should not be considered quantitative

A2B2-hetRTR05_11noCys

Laboratory: Wysocki-NH
Client: Baker-Ryan
Shipment Date: 2021_06_30
Data Recorded: Fri, 02 Jul 2021, 13:31
Instrument: Q Exactive UHMR Orbitrap
Study: OBE 200 mM AmAc, RO22
Sample Conditions: 25mM Tris pH8, 300mM NaCl
Concentration (ug/uL): 1.0
Injection (uL): 1.0
Comment:
Date Analyzed: Fri, 16 Jul 2021, 11:33 Eastern Daylight Time
File Name: 20210701_Ryan_RK846_IST200_6k

Quality Check:
Average Peaks Score: 0.67

Expected Oligomers:
Passed:
Mixed:
Failed:

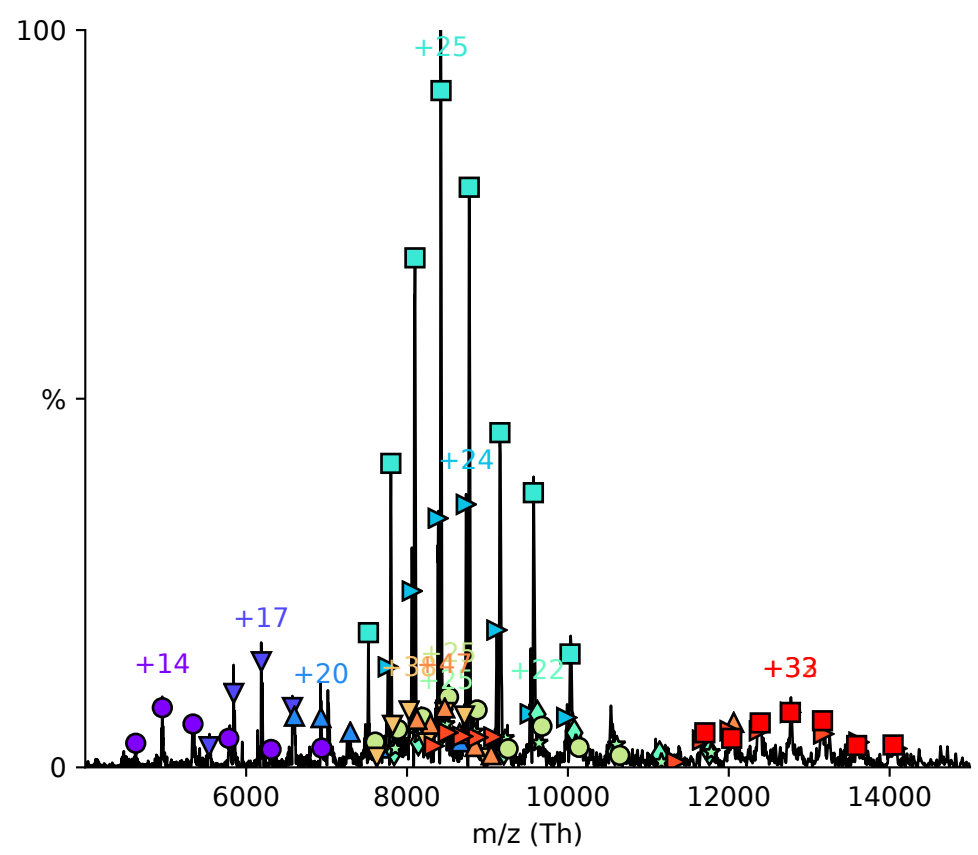

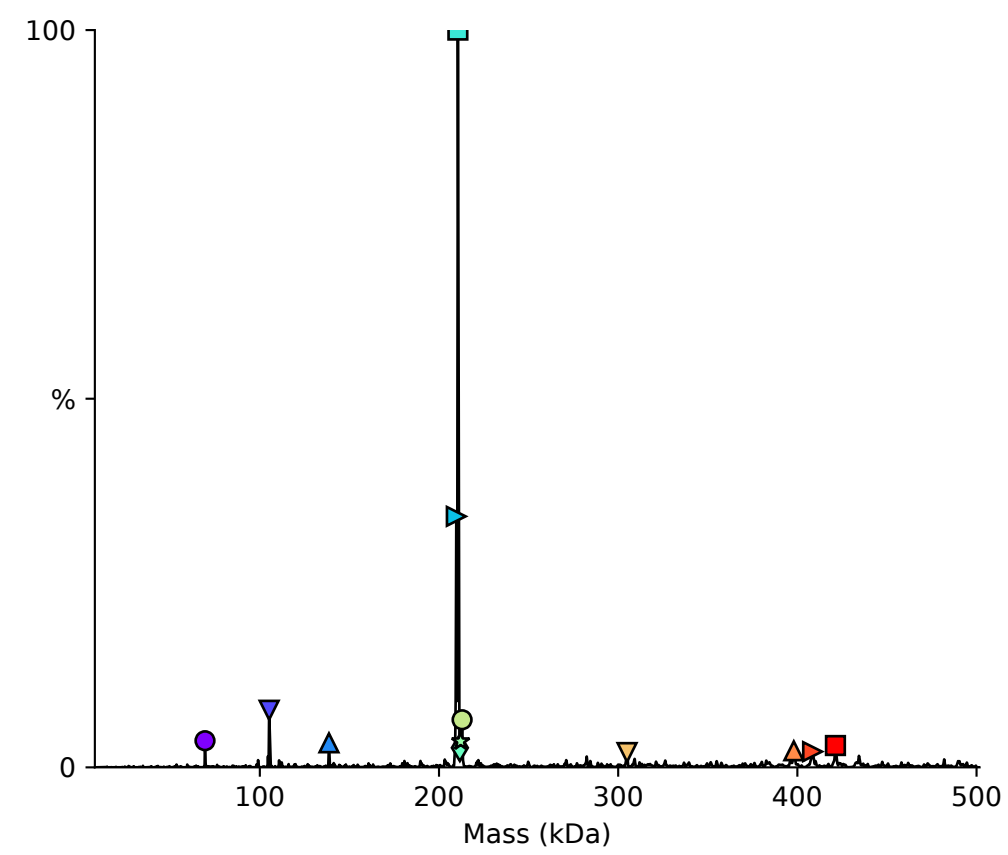

Symbol	Mass (Da)	Intensity[1]	Expected	Difference (Da)	Name/Species/Oligomer
○	69394	4	69377	17	1[RK846_A]
▽	105198	8	105300	−102	1[RK846_B] 1[RK846_A]
△	138579	3	138754	−175	2[RK846_A]
▷	209646	34	210600	−954	
□	210536	100	210600	−64	2[RK846_B] 2[RK846_A]
◊	211638	3	210600	1038	
☆	212074	4	213069	−995	
○	212885	6	213069	−184	4[RK846_B] 1[RK846_A]
▽	304948	2	313431	−8483	
△	398006	2	385277	12729	
▷	408491	2	421200	−12709	
□	421253	3	421200	53	4[RK846_B] 4[RK846_A]

[1] Intensity values should not be considered quantitative

A2B2-hetRTR05_16

Laboratory: Wysocki-NH
Client: Baker-Ryan
Shipment Date: 2021_06_30
Data Recorded: Fri, 02 Jul 2021, 13:42
Instrument: Q Exactive UHMR Orbitrap
Study: OBE 200 mM AmAc, RO22
Sample Conditions: 25mM Tris pH8, 300mM NaCl
Concentration (ug/uL): 1.0
Injection (uL): 1.0
Comment:
Date Analyzed: Fri, 16 Jul 2021, 11:35 Eastern Daylight Time
File Name: 20210701_Ryan_RK847_IST200_6k

Quality Check:
Average Peaks Score: 0.7

Expected Oligomers:
Passed:
Mixed:
Failed:

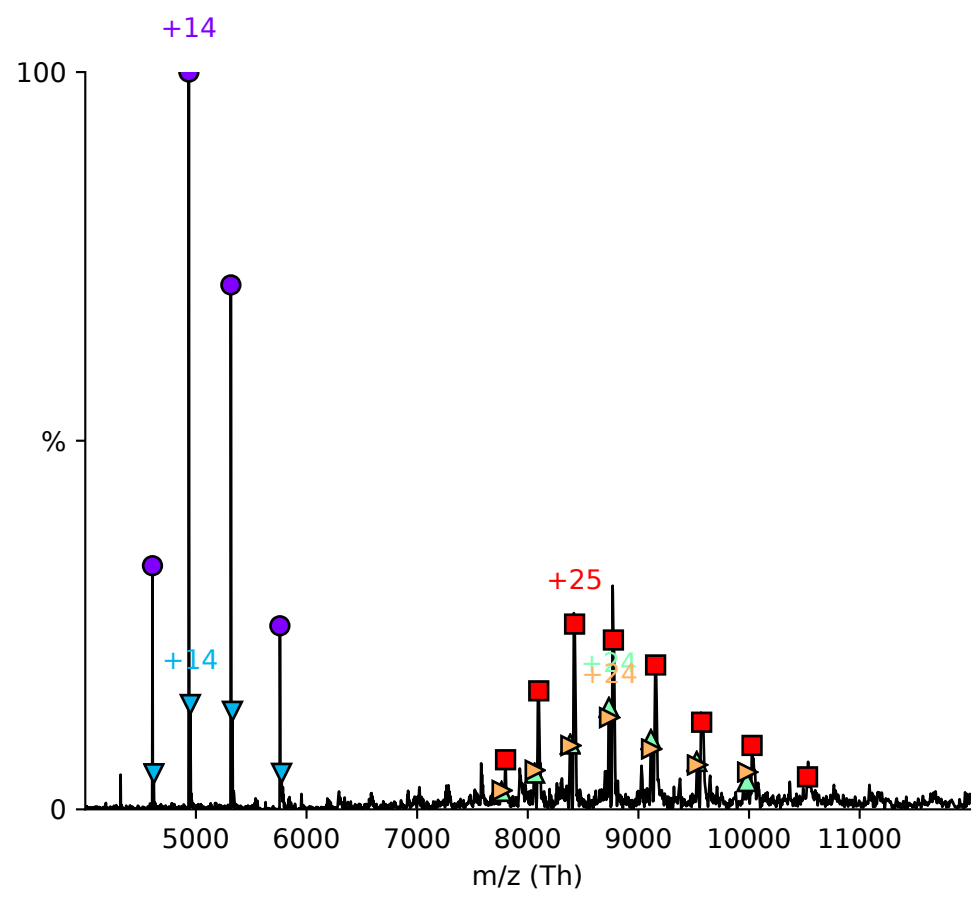

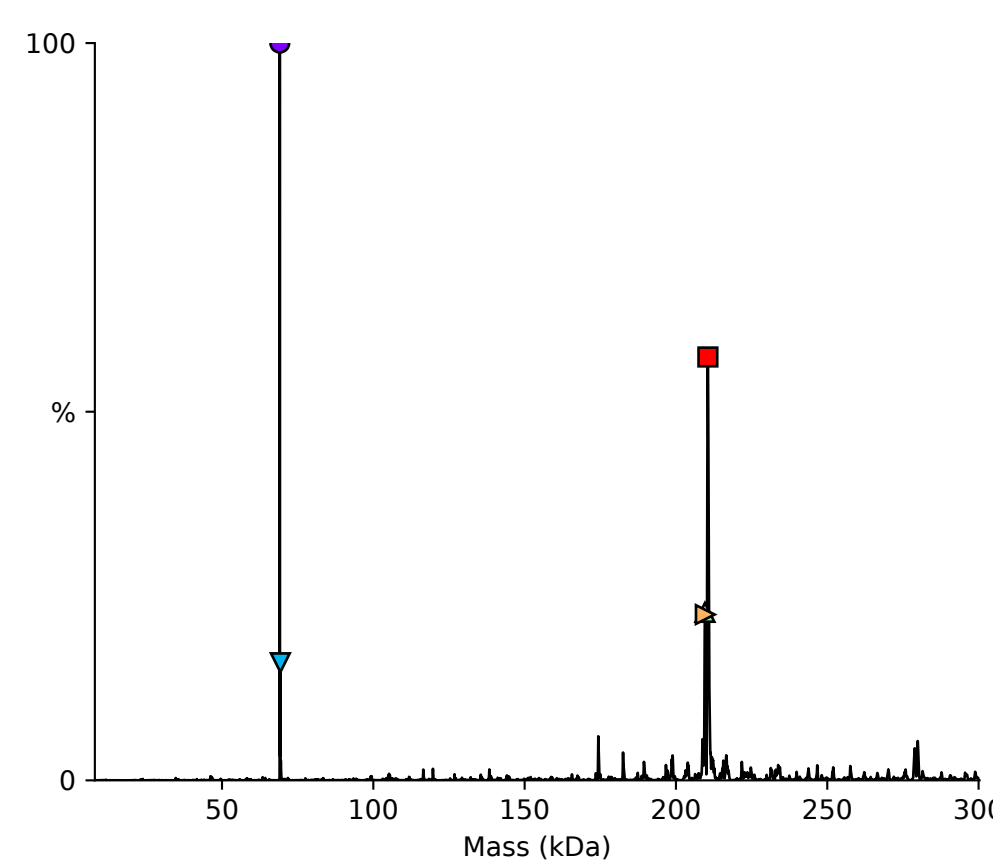

Symbol	Mass (Da)	Intensity[1]	Expected	Difference (Da)	Name/Species/Oligomer
○	69096	100	69249	−153	
▽	69281	16	69249	32	1[RK847_A]
△	209580	23	210630	−1050	
▷	209702	22	210630	−928	
□	210552	57	210630	−78	2[RK847_A] 2[RK847_B]

[1]Intensity values should not be considered quantitative

A2B2-hetRTR05_18noCys

Laboratory: Wysocki-NH
Client: Baker-Ryan
Shipment Date: 2021_06_30
Data Recorded: Fri, 02 Jul 2021, 13:53
Instrument: Q Exactive UHMR Orbitrap
Study: OBE 200 mM AmAc, RO22
Sample Conditions: 25mM Tris pH8, 300mM NaCl
Concentration (ug/uL): 1.0
Injection (uL): 1.0
Comment:
Date Analyzed: Fri, 16 Jul 2021, 11:40 Eastern Daylight Time
File Name: 20210701_Ryan_RK848_IST200_6k

Quality Check:
Average Peaks Score: 0.7

Expected Oligomers:
Passed:
Mixed:
Failed:

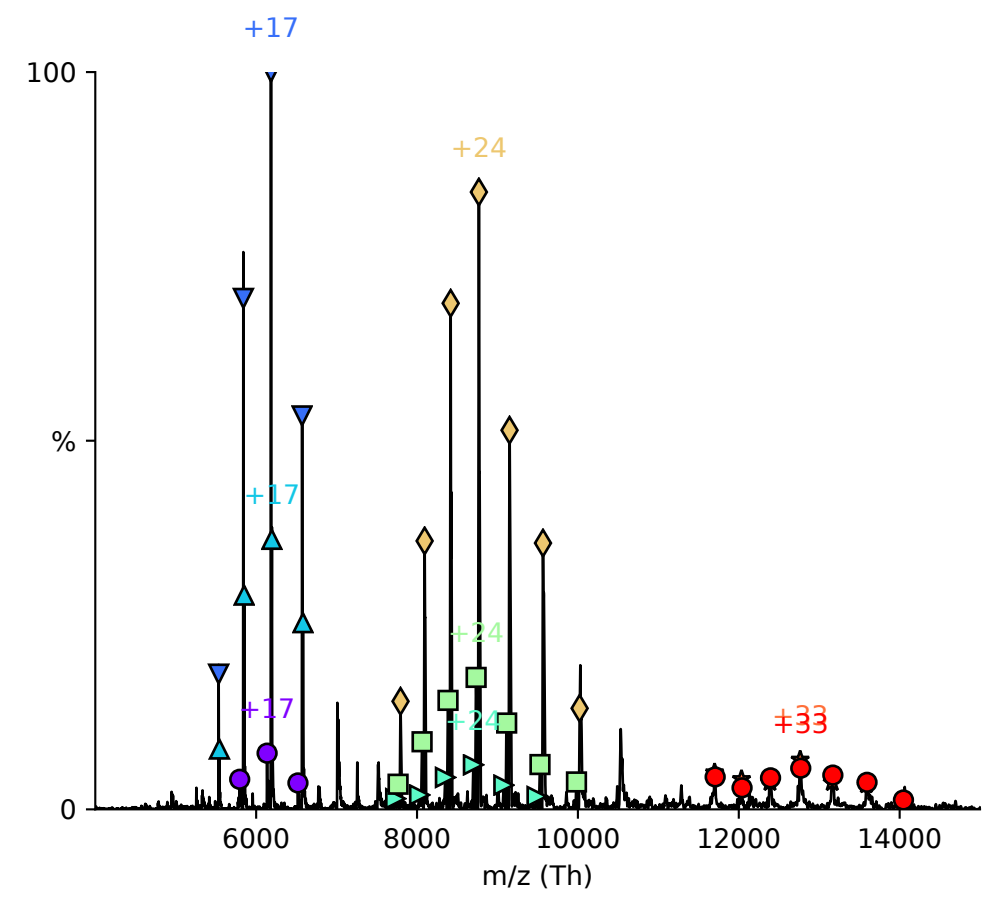

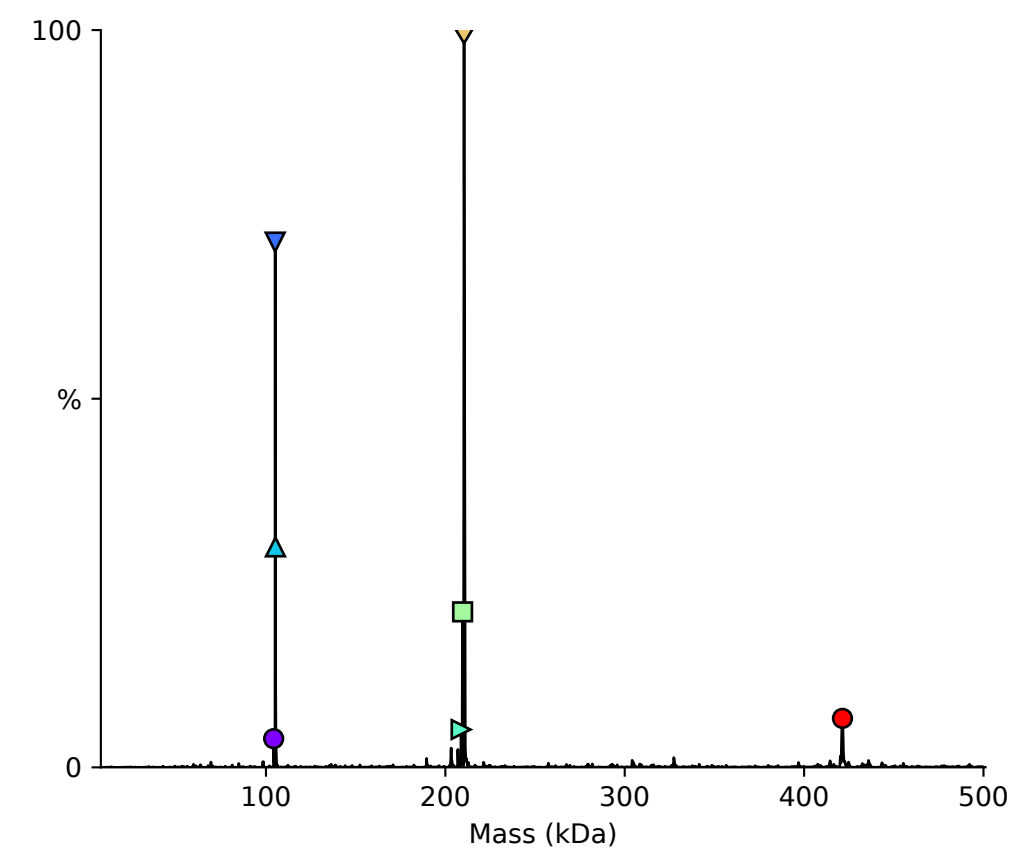

Symbol	Mass (Da)	Intensity[1]	Expected	Difference (Da)	Name/Species/Oligomer
○	104296	4	105398	−1102	
▽	105121	71	105398	−277	1[RK848_B] 1[RK848_A]
△	105297	30	105398	−101	1[RK848_B] 1[RK848_A]
▷	208786	5	208238	548	
□	209606	21	210796	−1190	
◊	210428	100	210796	−368	2[RK848_B] 2[RK848_A]
☆	421150	7	421591	−441	
○	421392	7	421591	−199	4[RK848_B] 4[RK848_A]

[1] Intensity values should not be considered quantitative

A2B2-hetRTR05_24noCys

Laboratory: Wysocki-NH
Client: Baker-Ryan
Shipment Date: 2021_06_30
Data Recorded: Fri, 02 Jul 2021, 14:04
Instrument: Q Exactive UHMR Orbitrap
Study: OBE 200 mM AmAc, RO22
Sample Conditions: 25mM Tris pH8, 300mM NaCl
Concentration (ug/uL): 1.0
Injection (uL): 1.0
Comment:
Date Analyzed: Fri, 16 Jul 2021, 11:45 Eastern Daylight Time
File Name: 20210701_Ryan_RK849_IST200_6k

Quality Check:
Average Peaks Score: 0.2

Expected Oligomers:
Passed:
Mixed:
Failed:

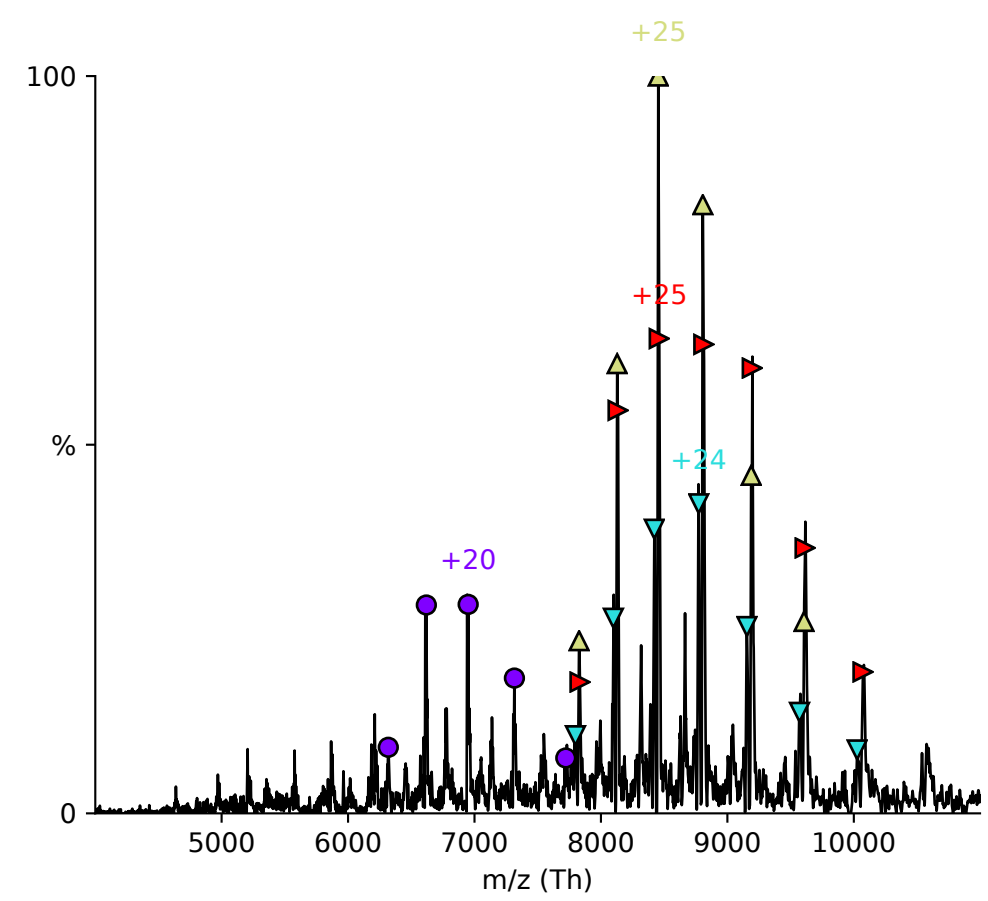

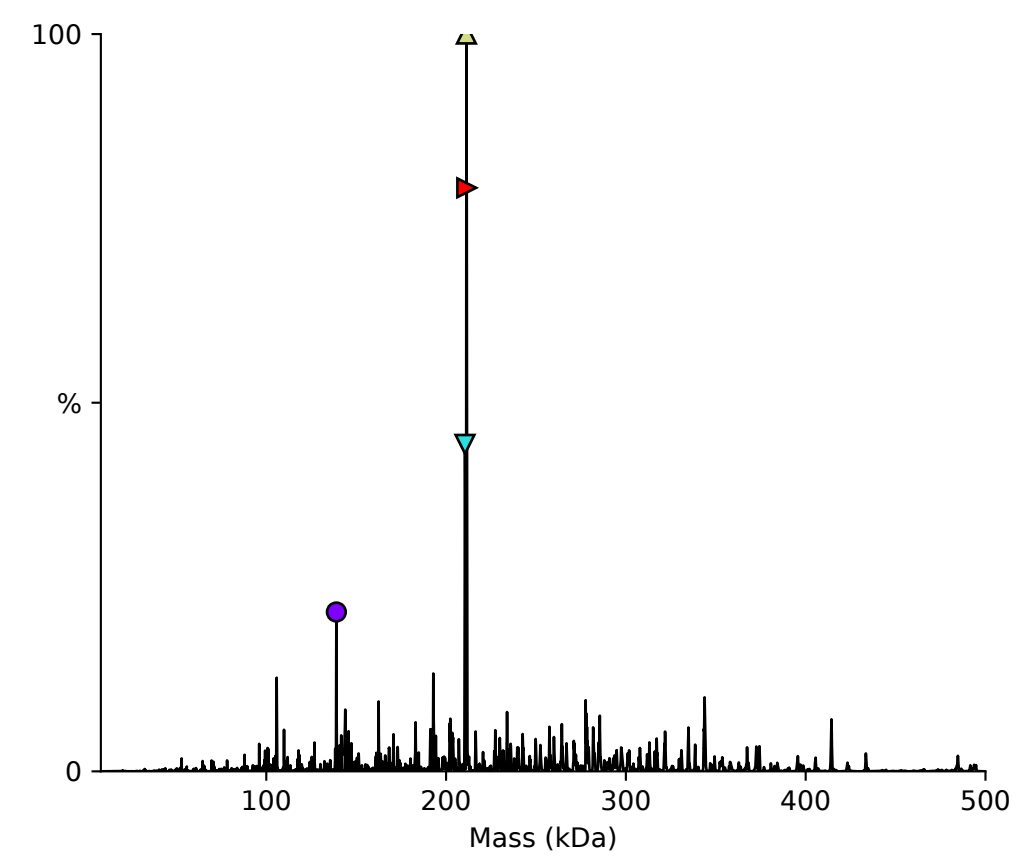

Symbol	Mass (Da)	Intensity[1]	Expected	Difference (Da)	Name/Species/Oligomer
○	138979	22	139104	−125	2[RK849_A]
▽	210530	44	211403	−873	
△	211311	100	211403	−92	2[RK849_B] 2[RK849_A]
▷	211492	79	211403	89	2[RK849_B] 2[RK849_A]

[1] Intensity values should not be considered quantitative

A2B2-hetRTR11_18

Laboratory: Wysocki-NH
Client: Baker-Ryan
Shipment Date: 2021_06_30
Data Recorded: Fri, 02 Jul 2021, 14:15
Instrument: Q Exactive UHMR Orbitrap
Study: OBE 200 mM AmAc, RO22
Sample Conditions: 25mM Tris pH8, 300mM NaCl
Concentration (ug/uL): 1.0
Injection (uL): 1.0
Comment:
Date Analyzed: Fri, 16 Jul 2021, 12:01 Eastern Daylight Time
File Name: 20210701_Ryan_RK850_IST200_6k

Quality Check:
Average Peaks Score: 0.58

Expected Oligomers:
Passed:
Mixed:
Failed:

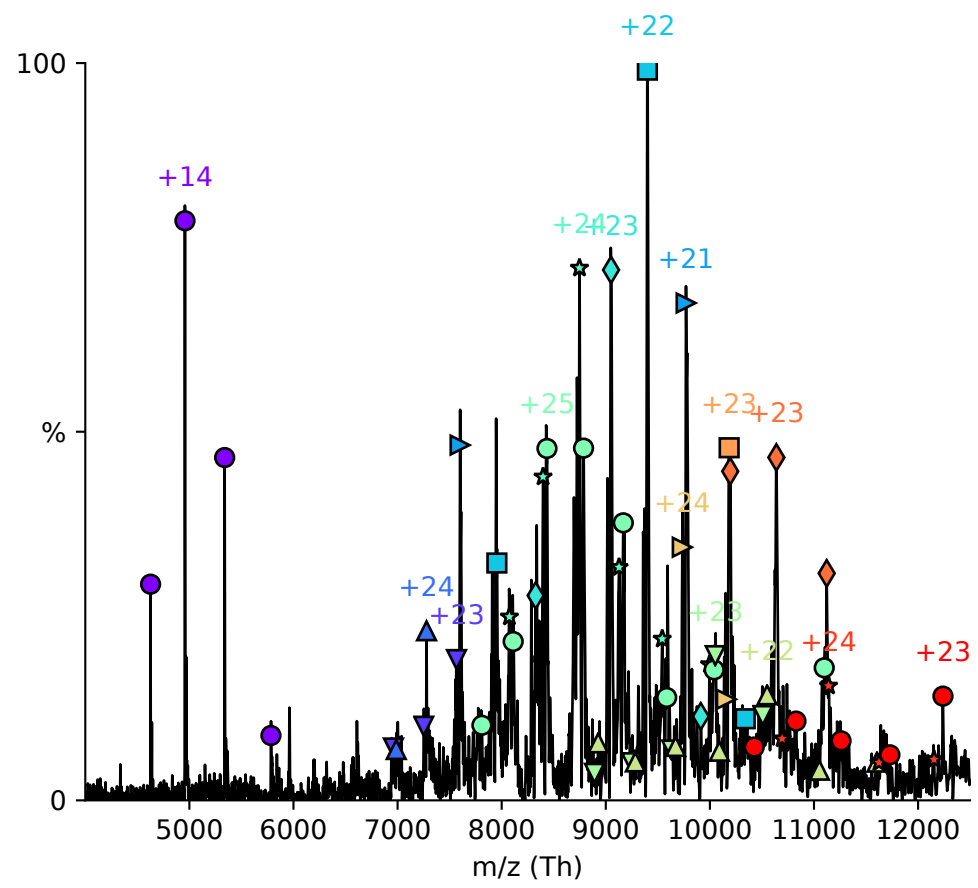

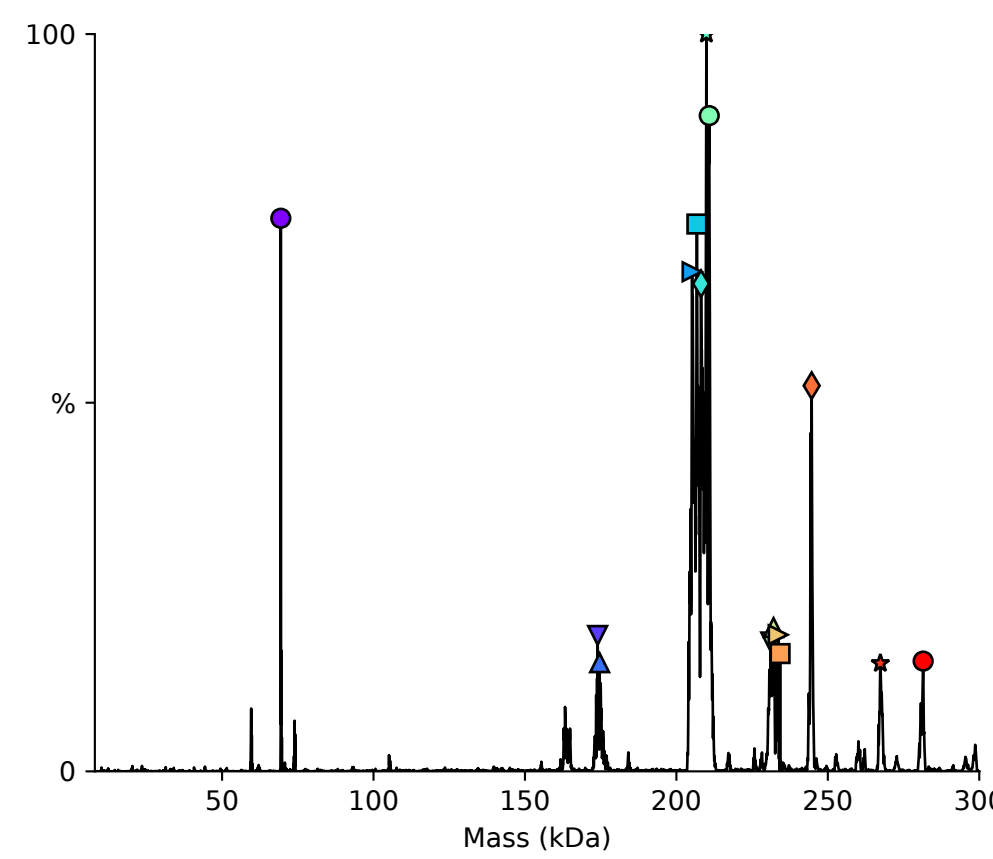

Symbol	Mass (Da)	Intensity[1]	Expected	Difference (Da)	Name/Species/Oligomer
○	69392	75	69548	−156	1[RK850_A]
▽	173993	18	175066	−1073	
△	174648	15	175066	−418	2[RK850_A] 1[RK850_B]
▷	205183	68	208644	−3461	
□	206768	74	208644	−1876	
◊	208137	66	208644	−507	3[RK850_A]
☆	209906	100	211037	−1131	
○	210847	89	211037	−190	2[RK850_A] 2[RK850_B]
▽	231156	18	244614	−13458	
△	232072	20	244614	−12542	
▷	233603	19	244614	−11011	
□	234228	16	244614	−10386	
◊	244664	52	244614	50	3[RK850_A] 1[RK850_B]
☆	267334	15	278192	−10858	
○	281470	15	280585	885	3[RK850_A] 2[RK850_B]

[1] Intensity values should not be considered quantitative

A2B2-hetRTR11noCys_16

Laboratory: Wysocki-AN
Client: Baker-Ryan
Shipment Date: 2021_07_07
Data Recorded: Fri, 09 Jul 2021, 06:43
Instrument: Q Exactive UHMR Orbitrap
Study: OBE 200 mM AmAc, RO22
Sample Conditions: 25mM Tris, 300mM NaCl, pH 8
Concentration (ug/uL): 0.0
Injection (uL): 0.5
Comment: Assignments are bit off
Date Analyzed: Wed, 11 Aug 2021, 12:40 Eastern Daylight Time
File Name: 20210708_Ryan_RK851_6k_IST120_HCD060

Quality Check:
Average Peaks Score: 0.74

Expected Oligomers:
Passed:
Mixed:
Failed:

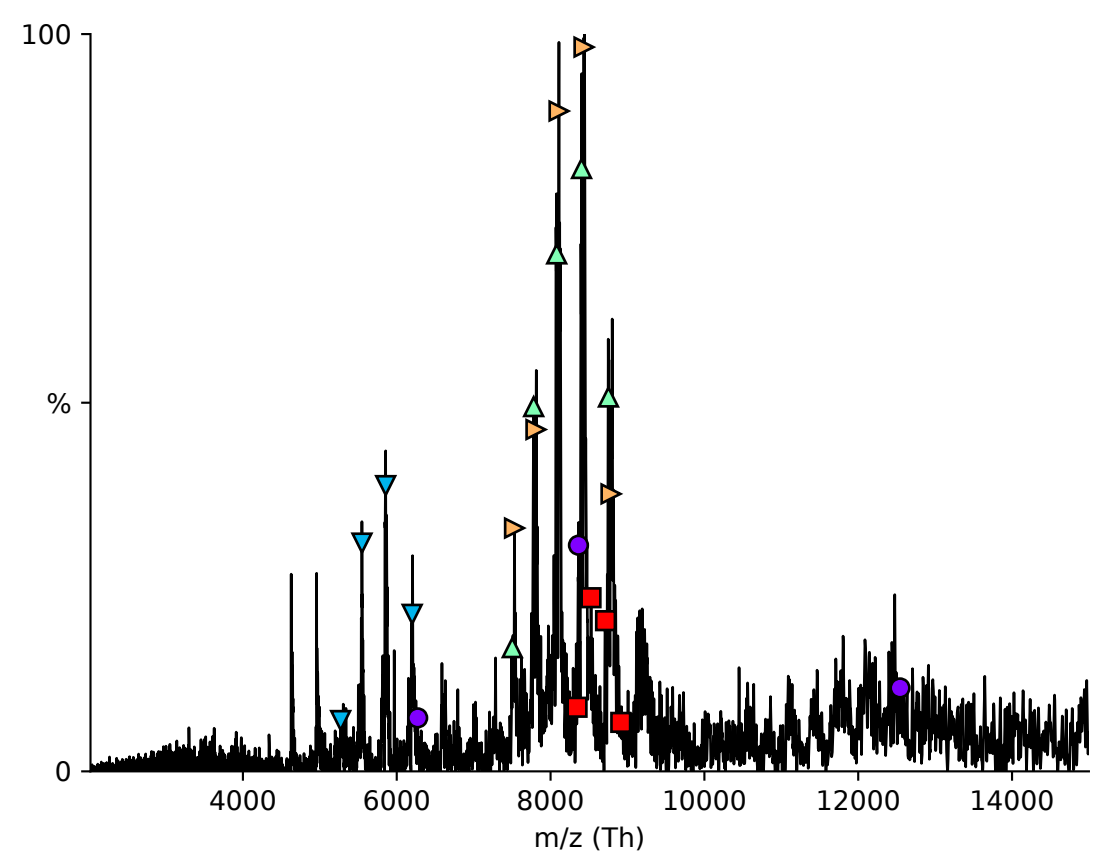

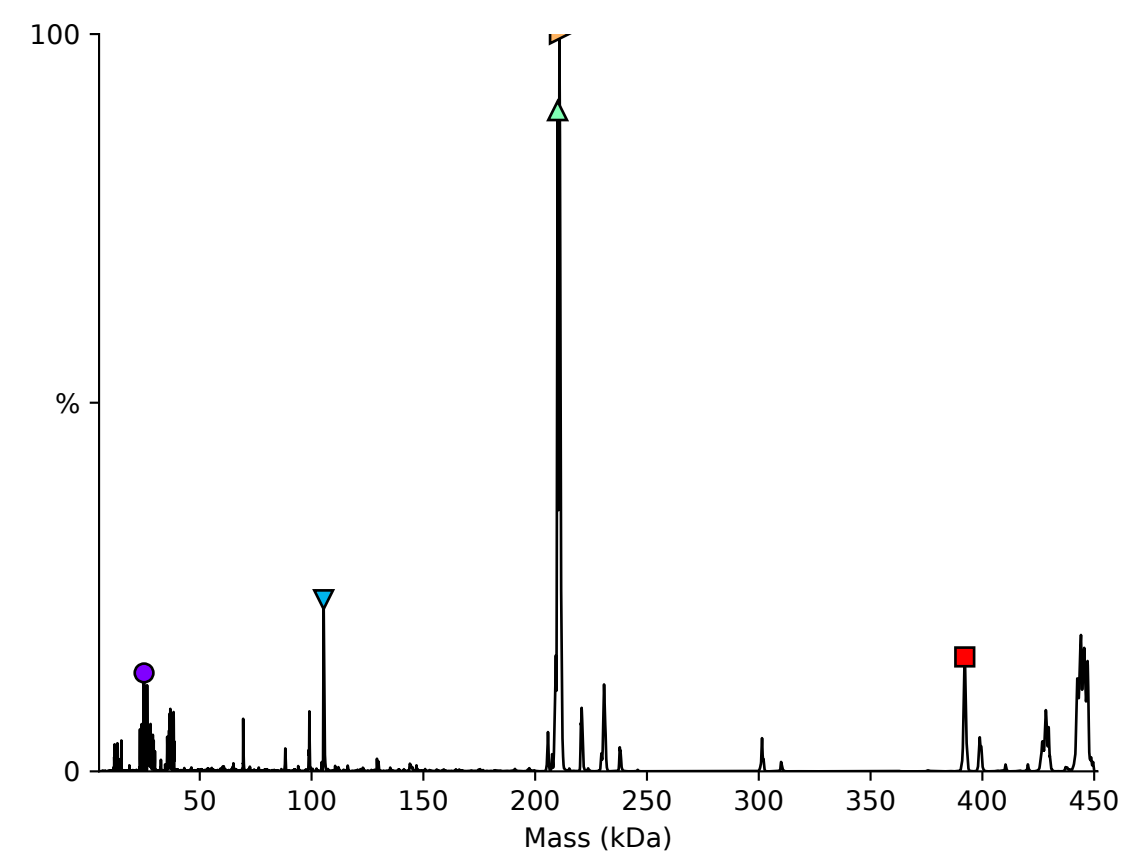

Symbol	Mass (Da)	Intensity[1]	Expected	Difference (Da)	Name/Species/Oligomer
○	25085	13	36043	−10958	
▽	105395	23	105479	−84	1[RK851-B] 1[RK851-A]
△	210040	90	210959	−919	2[RK851-B] 2[RK851-A]
▷	210835	100	210959	−124	2[RK851-B] 2[RK851-A]
□	392130	16	388525	3605	

[1]Intensity values should not be assumed as quantitative

A2B2-hetRTR11noCys_18noCys

Laboratory: Wysocki-AN
Client: Baker-Ryan
Shipment Date: 2021_07_07
Data Recorded: Fri, 09 Jul 2021, 06:54
Instrument: Q Exactive UHMR Orbitrap
Study: OBE 200 mM AmAc, RO22
Sample Conditions: 25mM Tris, 300mM NaCl, pH 8
Concentration (ug/uL): 0.0
Injection (uL): 0.5
Comment:
Date Analyzed: Wed, 11 Aug 2021, 12:49 Eastern Daylight Time
File Name: 20210708_Ryan_RK852_6k_IST120_HCD060

Quality Check:
Average Peaks Score: 0.15

Expected Oligomers:
Passed:
Mixed:
Failed:

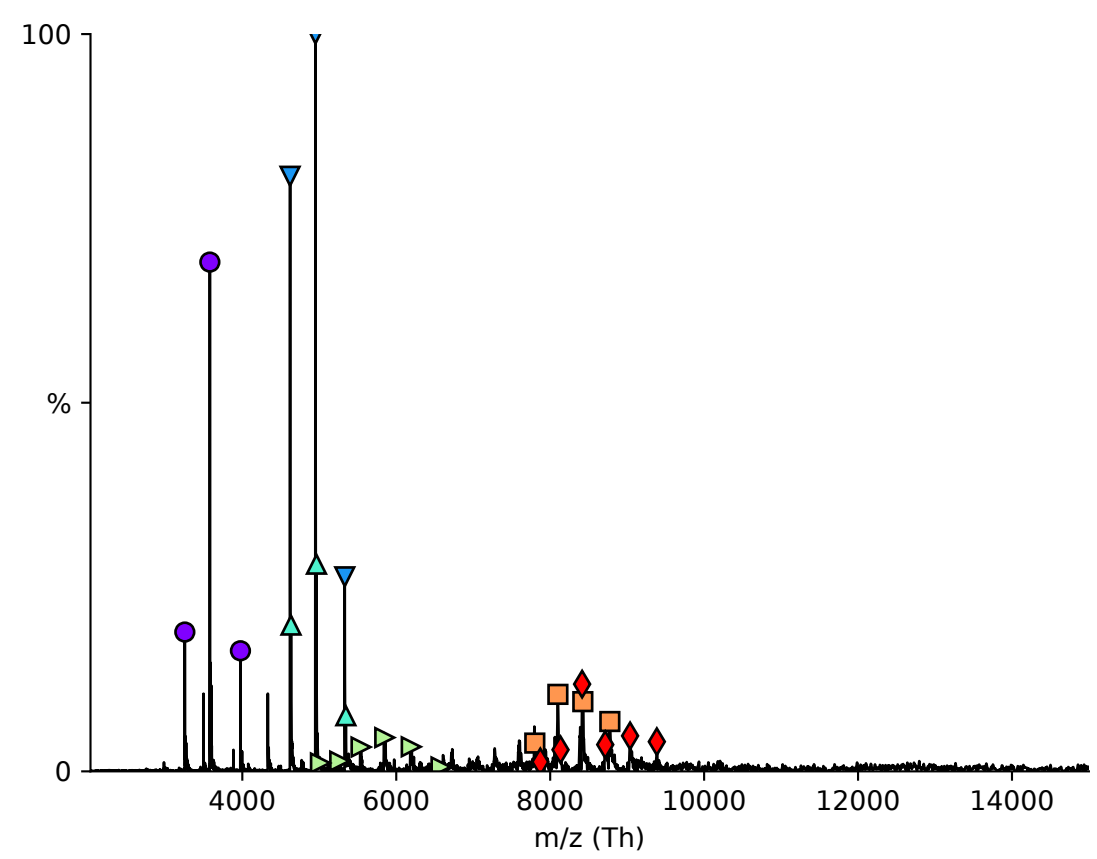

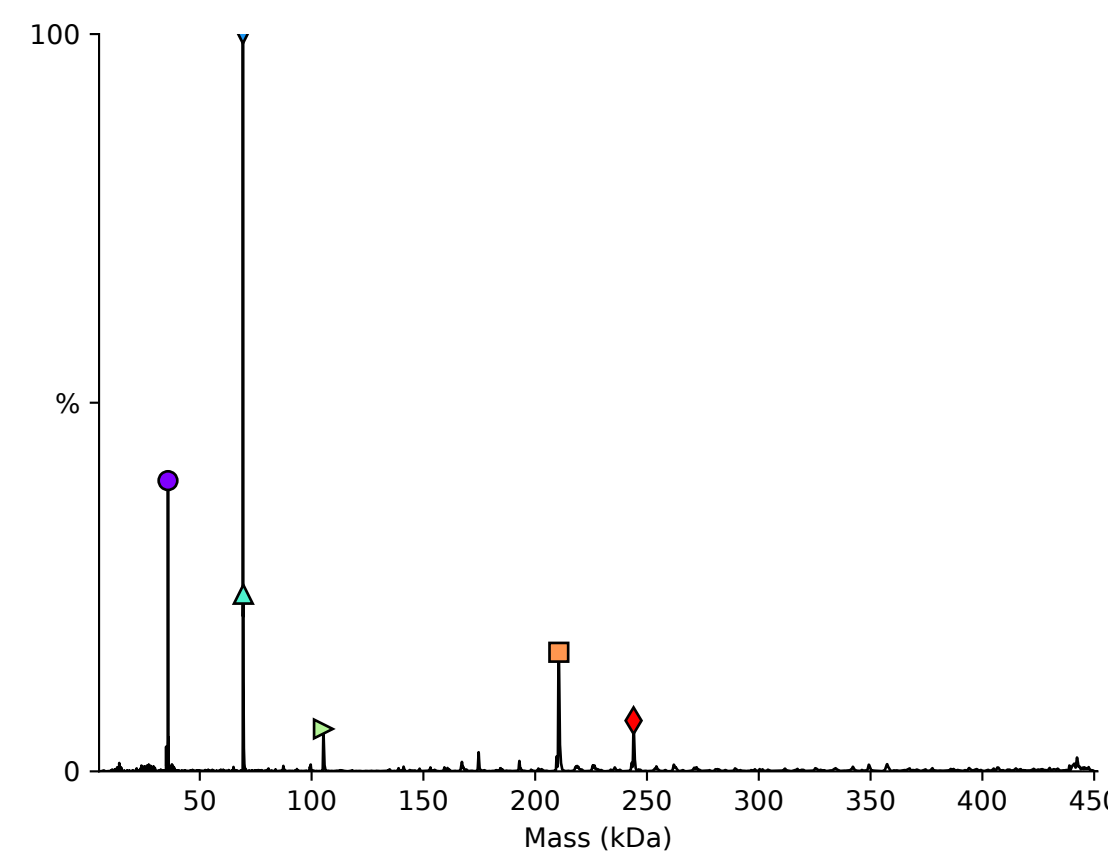

Symbol	Mass (Da)	Intensity[1]	Expected	Difference (Da)	Name/Species/Oligomer
○	35772	39	35904	−132	1[RK852-B]
▽	69291	100	69442	−151	1[RK852-A]
△	69475	24	69442	33	1[RK852-A]
▷	105286	6	105346	−60	1[RK852-A] 1[RK852-B]
□	210576	16	210692	−116	2[RK852-A] 2[RK852-B]
◊	244000	7	244230	−230	3[RK852-A] 1[RK852-B]

[1]Intensity values should not be assumed as quantitative

A2B2-hetRTR11noCys_24noCys

Laboratory: Wysocki-AN
Client: Baker-Ryan
Shipment Date: 2021_07_07
Data Recorded: Fri, 09 Jul 2021, 07:05
Instrument: Q Exactive UHMR Orbitrap
Study: OBE 200 mM AmAc, RO22
Sample Conditions: 25mM Tris, 300mM NaCl, pH 8
Concentration (ug/uL): 0.0
Injection (uL): 0.5
Comment:
Date Analyzed: Wed, 11 Aug 2021, 12:51 Eastern Daylight Time
File Name: 20210708_Ryan_RK853_6k_IST120_HCD060

Quality Check:
Average Peaks Score: 0.16

Expected Oligomers:
Passed:
Mixed:
Failed:

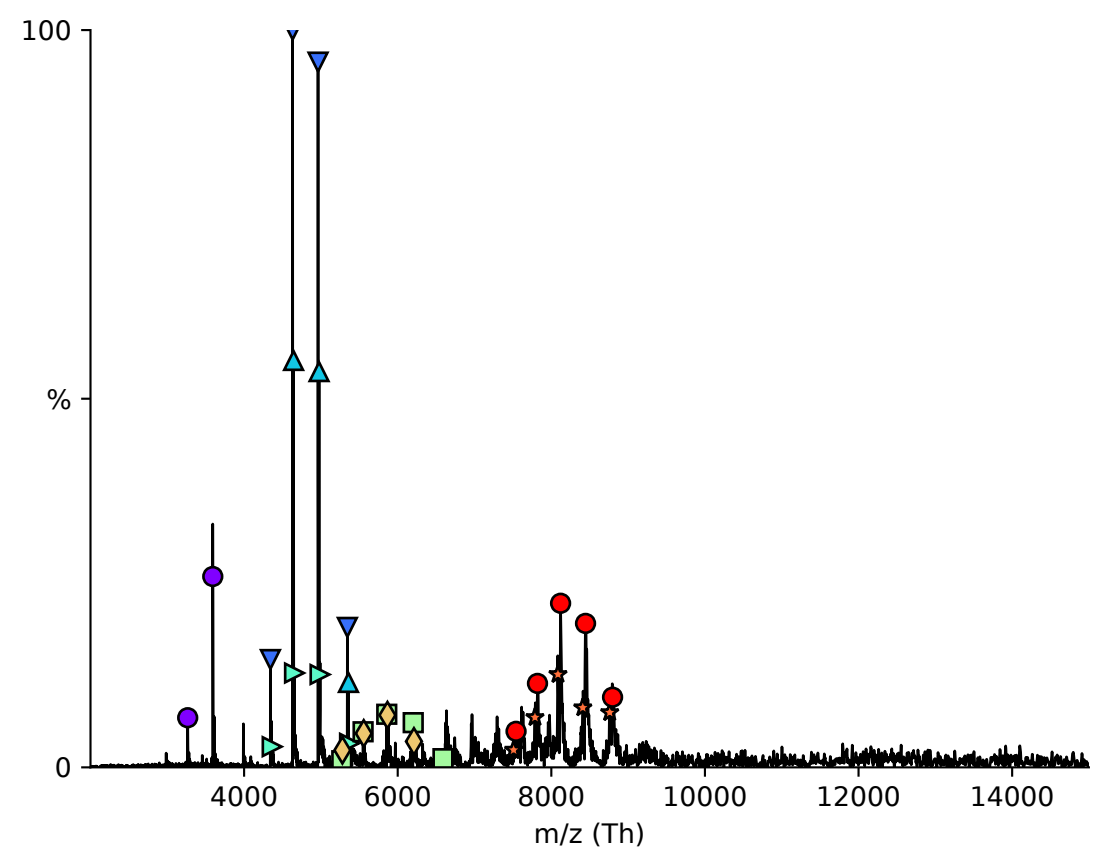

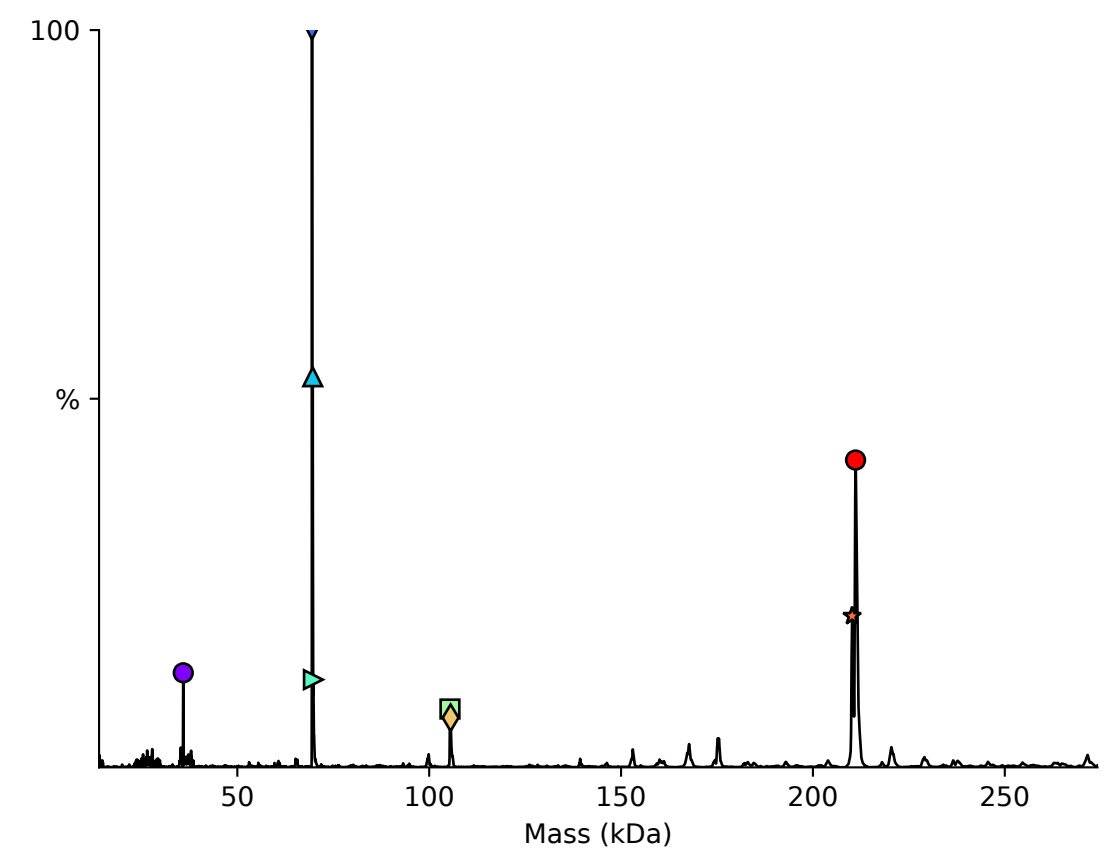

Symbol	Mass (Da)	Intensity[1]	Expected	Difference (Da)	Name/Species/Oligomer
○	35911	13	35908	3	1[RK853-B]
▽	69466	100	69616	−150	1[RK853-A]
△	69649	53	69616	33	1[RK853-A]
▷	69829	12	69616	213	
□	105415	8	105525	−110	1[RK853-B] 1[RK853-A]
◊	105576	7	105525	51	1[RK853-B] 1[RK853-A]
☆	210221	21	211049	−828	
○	211112	42	211049	63	2[RK853-B] 2[RK853-A]

[1]Intensity values should not be assumed as quantitative

A2B2-hetRTR16_18noCys

RK854 Exp: 2A2B A= 6927 B=35925
Date Analyzed: Wed, 03 Aug 2022, 10:58 Eastern Daylight Time
File Name: 20210826_Ryan_RK854_IST120_HCD005_6k_selection

Quality Check:
Average Peaks Score: 0.84

Expected Oligomers:
Passed:
Mixed:
Failed:

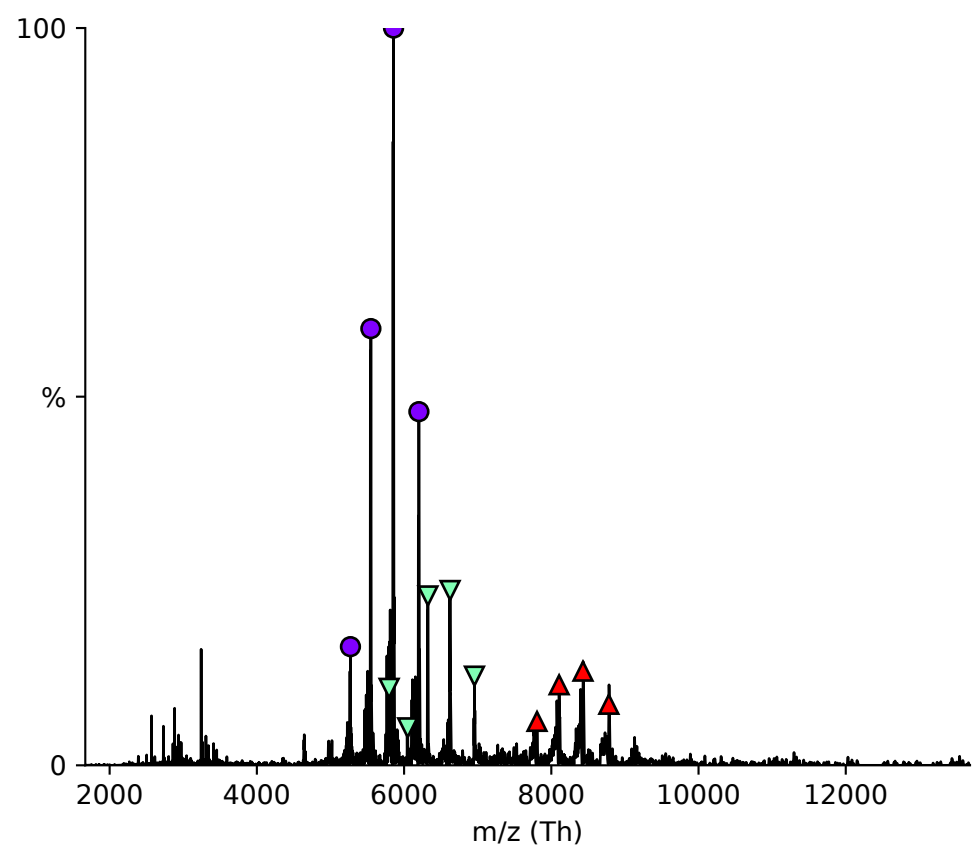

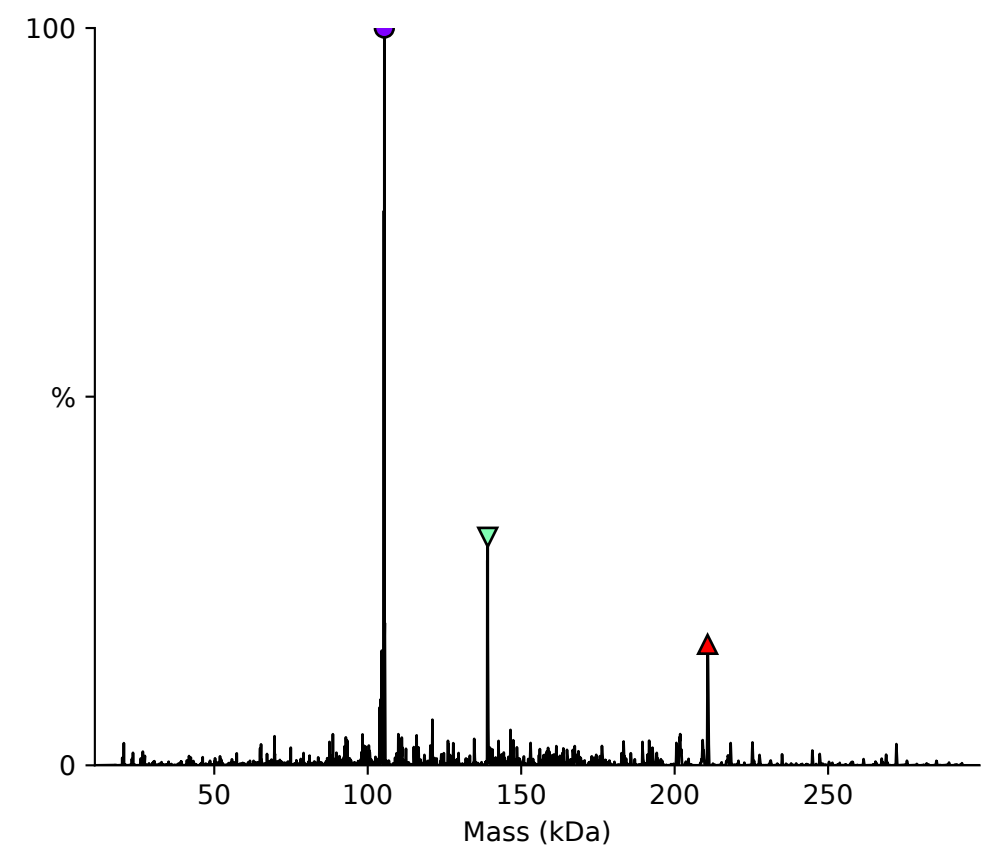

Symbol	Mass (Da)	Intensity[1]	Expected	Difference (Da)	Name/Species/Oligomer
◯	105406	100	105541	−135	1[B] 1[A]
▽	139098	31	139254	−156	2[A]
△	210729	16	211082	−353	2[B] 2[A]

[1]Intensity values should not be considered quantitative

A2B2-hetRTR16_24noCys

RK855 Exp: 2A 2B A= 69724 B=35914
Date Analyzed: Wed, 03 Aug 2022, 11:01 Eastern Daylight Time
File Name: 20210826_Ryan_RK855_IST120_HCD005_6k_selection

Quality Check:
Average Peaks Score: 0.74

Expected Oligomers:
Passed:
Mixed:
Failed:

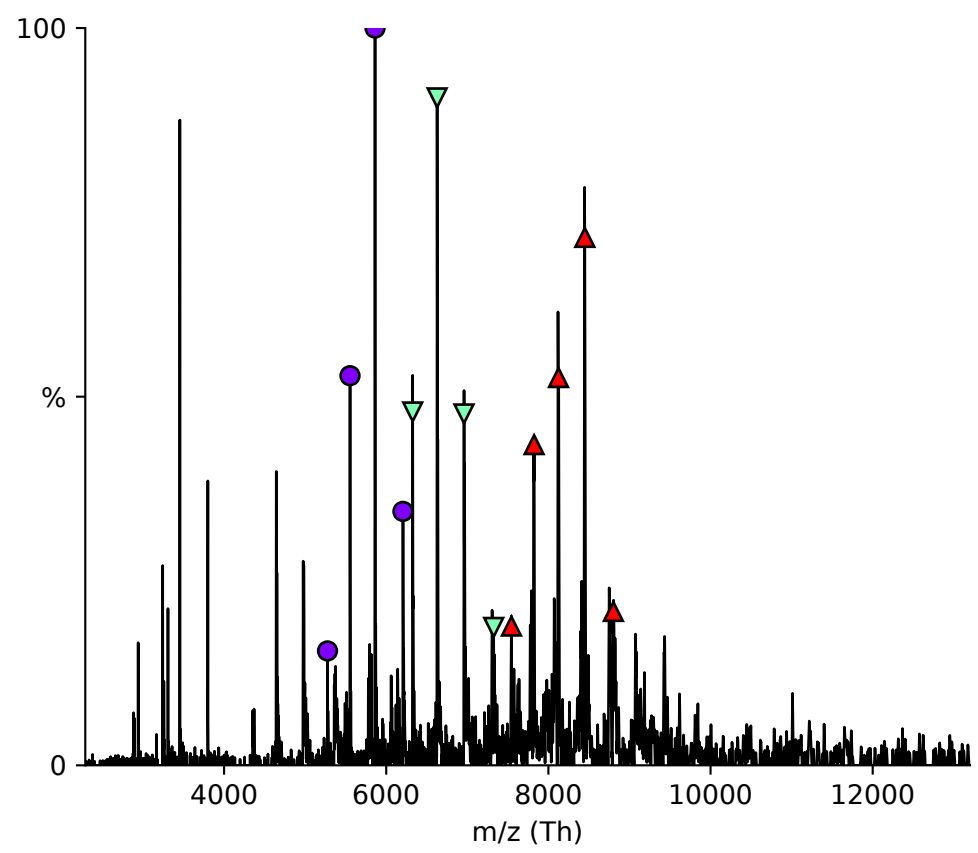

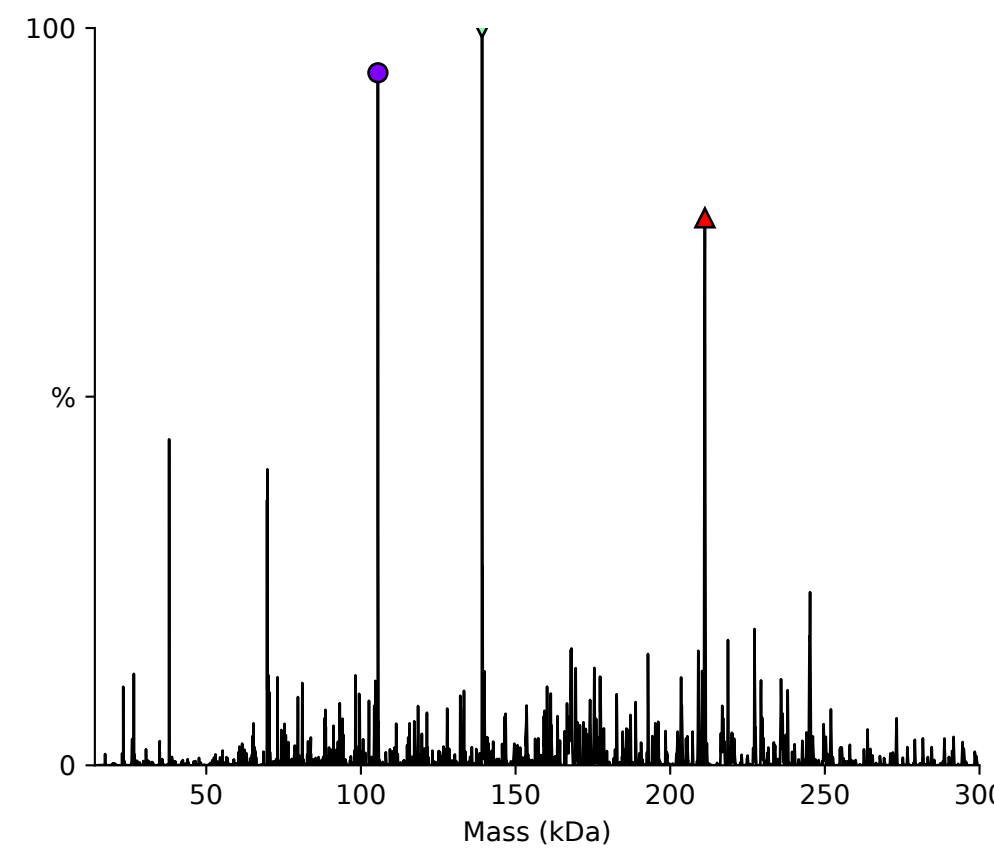

Symbol	Mass (Da)	Intensity[1]	Expected	Difference (Da)	Name/Species/Oligomer
○	105493	94	105638	−145	1[B] 1[A]
▽	139162	100	139449	−287	2[A]
△	211215	74	211277	−62	2[B] 2[A]

[1]Intensity values should not be considered quantitative

A2B2-hetRTR18_24noCys

Laboratory: Wysocki-AN
Client: Baker-Ryan
Shipment Date: 2021_07_07
Data Recorded: Fri, 09 Jul 2021, 07:16
Instrument: Q Exactive UHMR Orbitrap
Study: OBE 200 mM AmAc, RO22
Sample Conditions: 25mM Tris, 300mM NaCl, pH 8
Concentration (ug/uL): 0.0
Injection (uL): 0.5
Comment:
Date Analyzed: Wed, 11 Aug 2021, 12:53 Eastern Daylight Time
File Name: 20210708_Ryan_RK856_6k_IST120_HCD060

Quality Check:
Average Peaks Score: 0.16

Expected Oligomers:
Passed:
Mixed:
Failed:

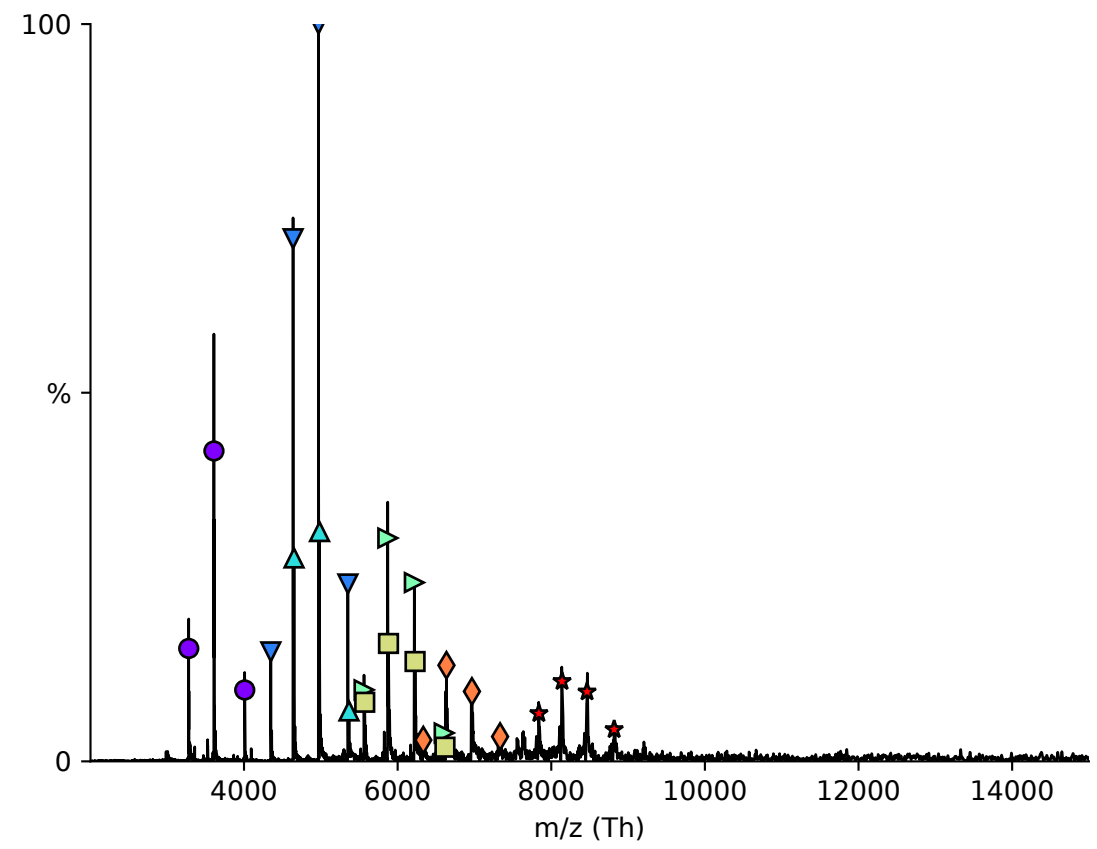

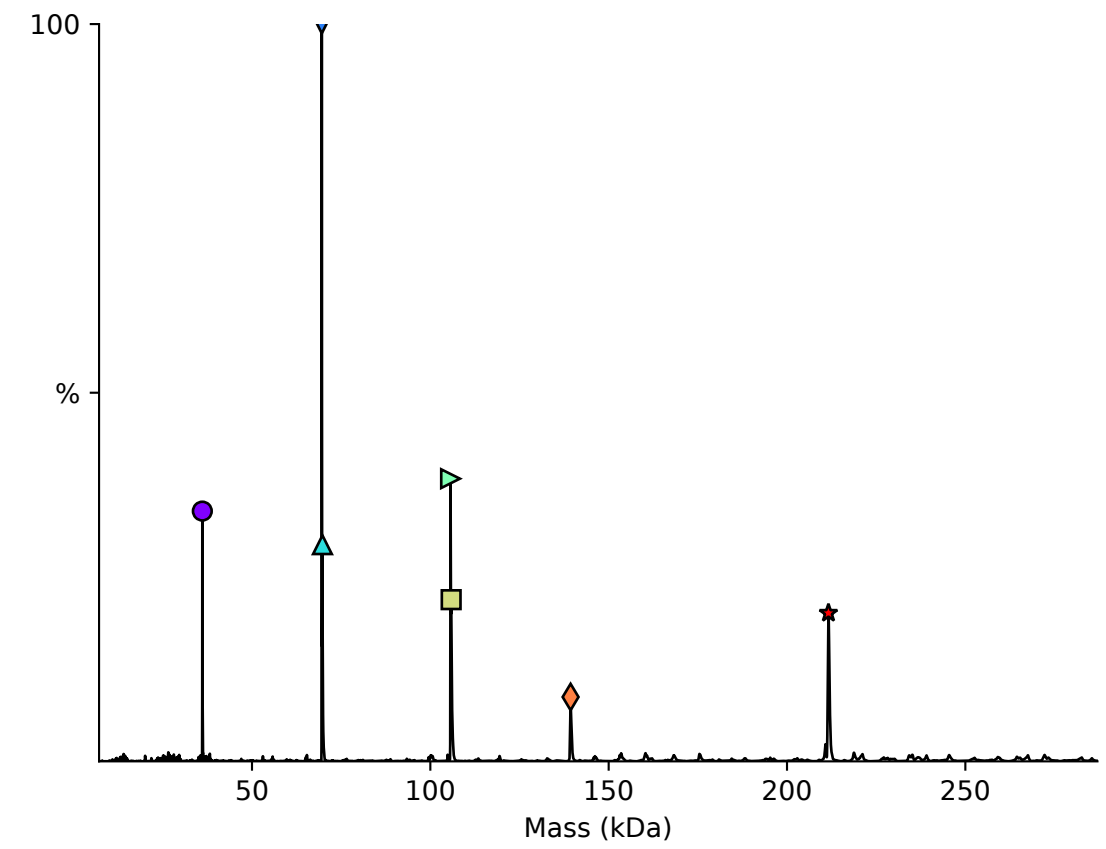

Symbol	Mass (Da)	Intensity[1]	Expected	Difference (Da)	Name/Species/Oligomer
○	36074	34	36070	4	1[RK856-B]
▽	69568	100	69719	−151	1[RK856-A]
△	69753	29	69719	34	1[RK856-A]
▷	105672	38	105790	−118	1[RK856-A] 1[RK856-B]
□	105839	22	105790	49	1[RK856-A] 1[RK856-B]
◊	139321	9	139439	−118	2[RK856-A]
☆	211616	20	211579	37	2[RK856-A] 2[RK856-B]

[1]Intensity values should not be assumed as quantitative

A(5)2B(7)2-hetRTR05_11noCys

Laboratory: Wysocki-SC, SML
Client: Ryan K
Shipment Date:
Data Recorded: Mon, 18 Oct 2021, 14:06
Instrument: Q Exactive UHMR Orbitrap
Study: OBE 200 mM AmAc, RO33
Sample Conditions: 25mM Tris pH 8, 300mM NaCl
Concentration (ug/uL): 1.002145303
Injection (uL): 0.997859289
Comment: Bit of a messy spectrum, but I was able to pull up the disulfide linked dimer, as well as the tetramer mass (assuming 2 di-sulfide linkages). Also found something slightly smaller.
Date Analyzed: Wed, 20 Oct 2021, 12:04 Eastern Daylight Time
File Name: 20211014_RK1007_6k_IST060_HCD005

Quality Check:
Average Peaks Score: 0.82

Expected Oligomers:
Passed:
Mixed:
Failed:

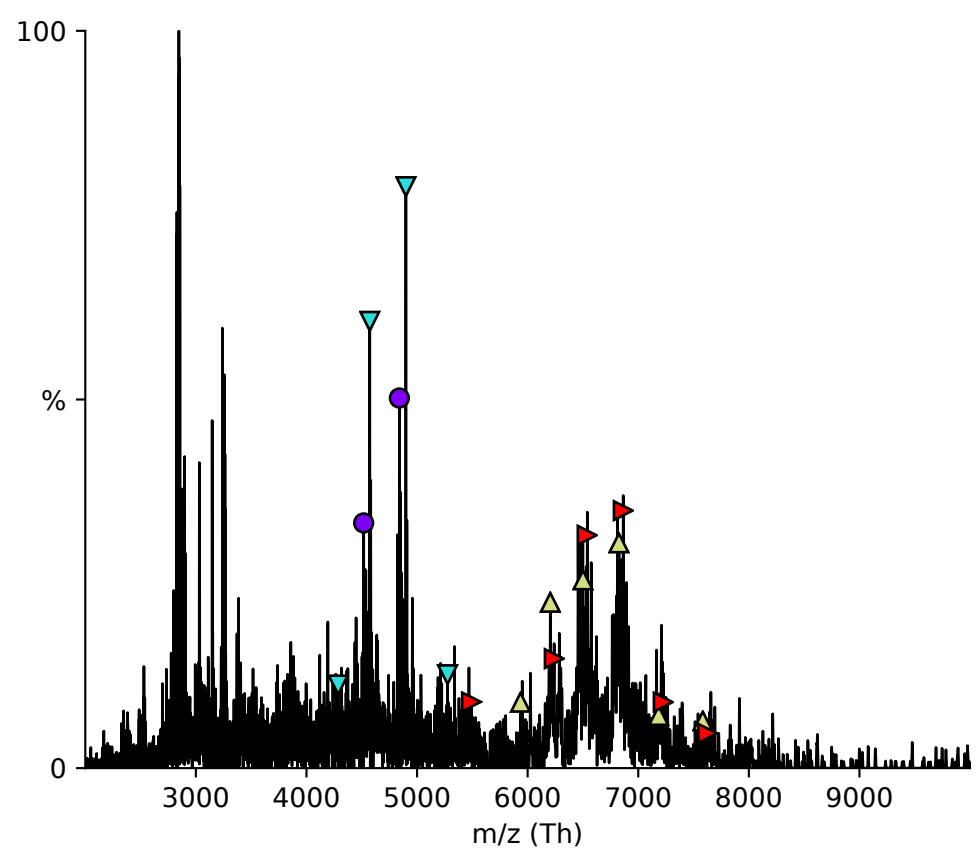

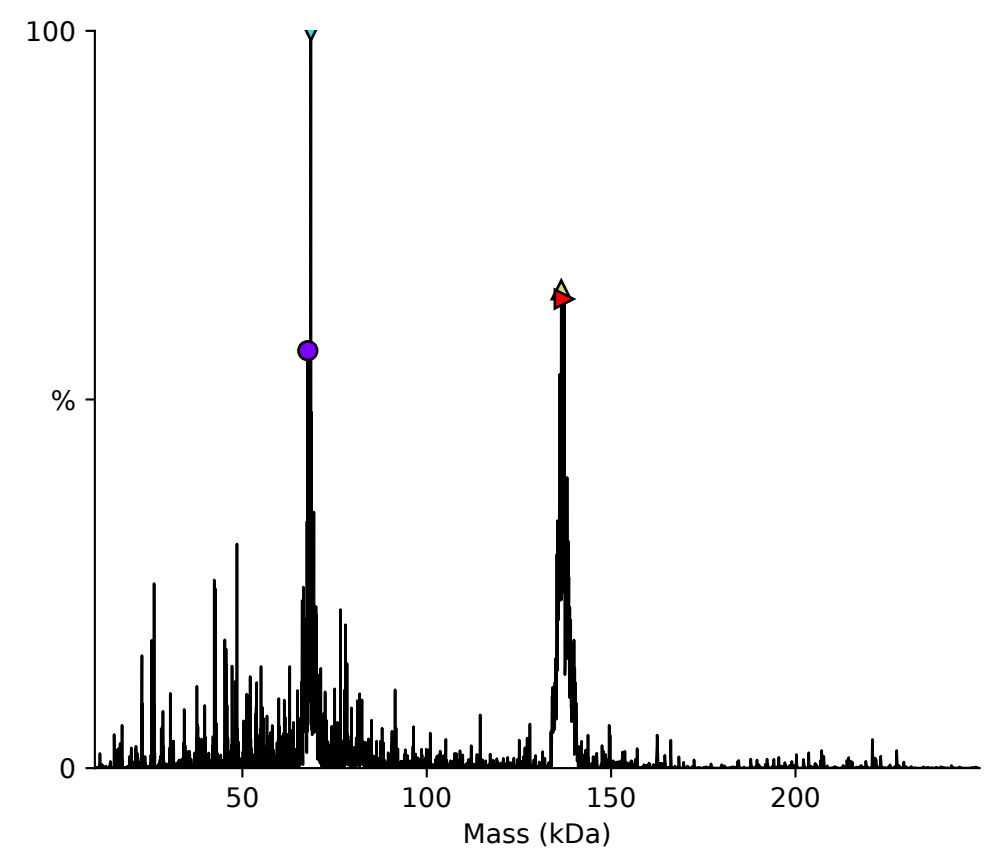

Symbol	Mass (Da)	Intensity[1]	Expected	Difference (Da)	Name/Species/Oligomer
○	67746	57	68040	−294	
▽	68570	100	68586	−16	1[RK1007-disulfide linked]
△	136484	65	136626	−142	
▷	137279	64	137172	107	2[RK1007-disulfide linked]

[1] Intensity values should not be considered quantitative

A(5)2B(7)2-hetRTR11noCys_16

Laboratory: Wysocki-SC, SML
Client: Ryan K
Shipment Date:
Data Recorded: Mon, 18 Oct 2021, 14:06
Instrument: Q Exactive UHMR Orbitrap
Study: OBE 200 mM AmAc, RO33
Sample Conditions: 25mM Tris pH 8, 300mM NaCl
Concentration (ug/uL): 0.770827427
Injection (uL): 1.297307238
Comment: Found the di-sulfide linked dimer. Also found a small amount of tetramer.
Date Analyzed: Tue, 19 Oct 2021, 13:04 Eastern Daylight Time
File Name: 20211014_RK1011_6k_IST060_HCD005

Quality Check:
Average Peaks Score: 0.89

Expected Oligomers:
Passed:
Mixed:
Failed:

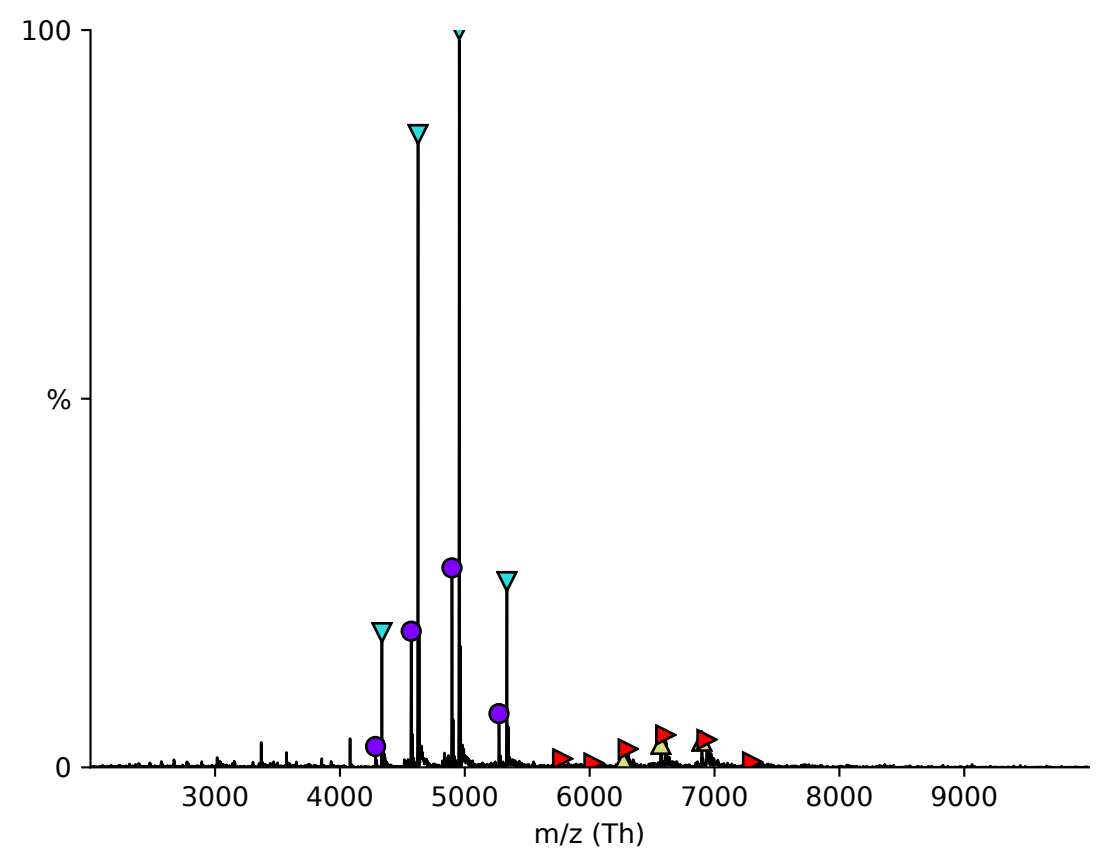

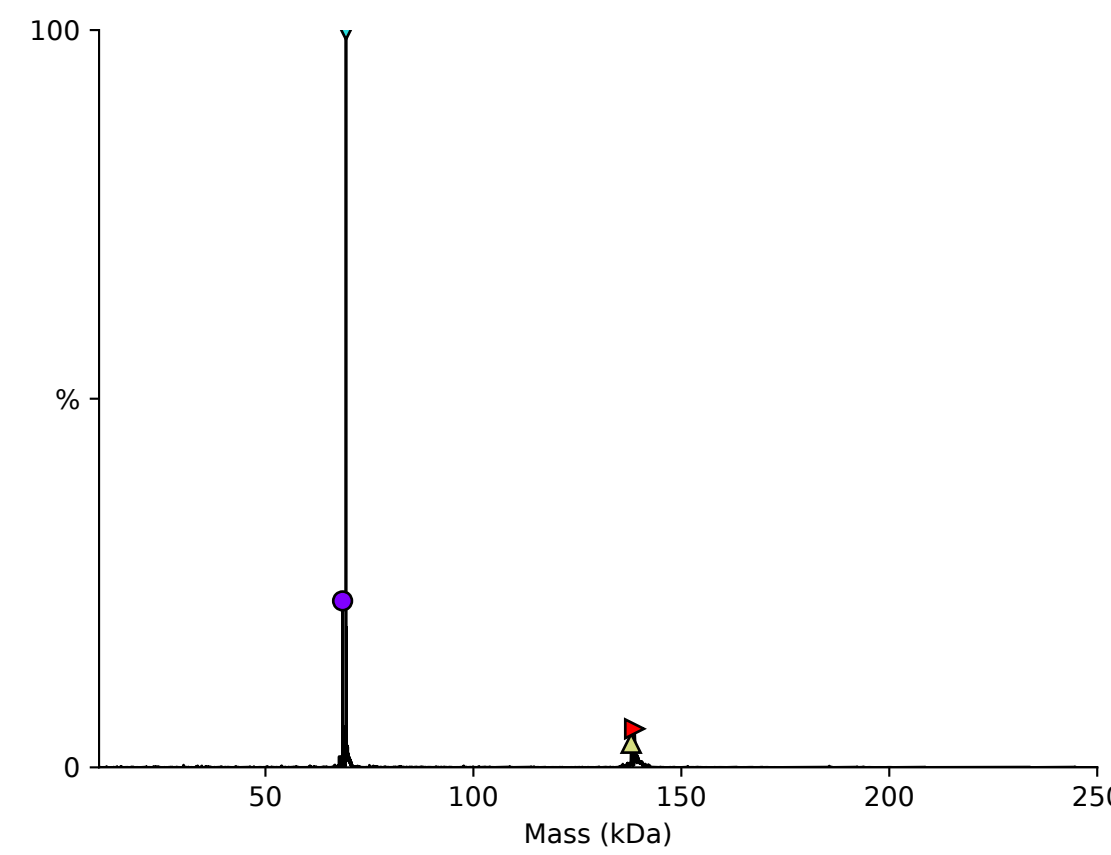

Symbol	Mass (Da)	Intensity[1]	Expected	Difference (Da)	Name/Species/Oligomer
○	68545	23	68076	469	
▽	69365	100	69381	−16	1[A-B-disulfidelinked]
△	137945	3	137457	488	
▷	138764	5	138762	2	2[A-B-disulfidelinked]

[1] Intensity values should not be considered quantitative

A(5)2B(7)2-hetRTR11noCys_24noCys

Laboratory: Wysocki-SC, SML
Client: Ryan K
Shipment Date:
Data Recorded: Mon, 18 Oct 2021, 14:06
Instrument: Q Exactive UHMR Orbitrap
Study: OBE 200 mM AmAc, RO33
Sample Conditions: 25mM Tris pH 8, 300mM NaCl
Concentration (ug/uL): 1.000635108
Injection (uL): 0.999365295
Comment: Found both monomers and the dimer between the two. But no tetramers.
Date Analyzed: Tue, 19 Oct 2021, 13:08 Eastern Daylight Time
File Name: 20211014_RK1012_6k_IST060_HCD005

Quality Check:
Average Peaks Score: 0.89

Expected Oligomers:
Passed:
Mixed:
Failed:

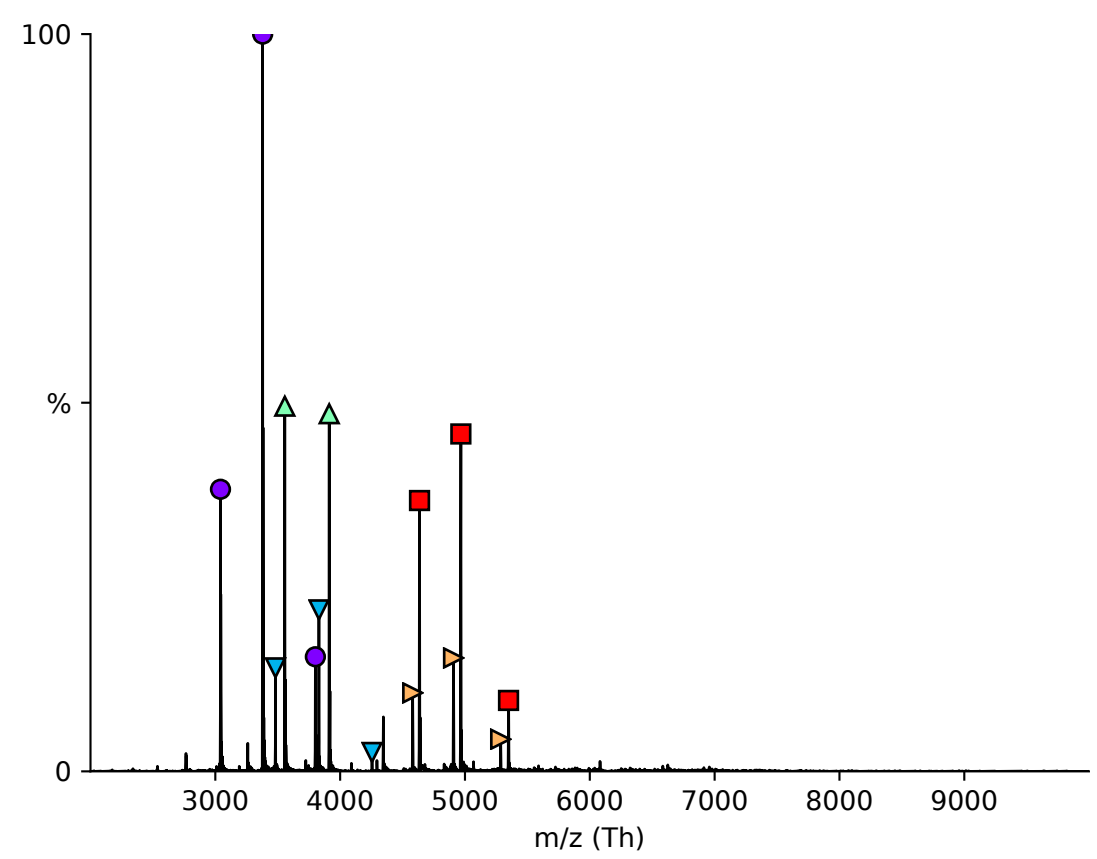

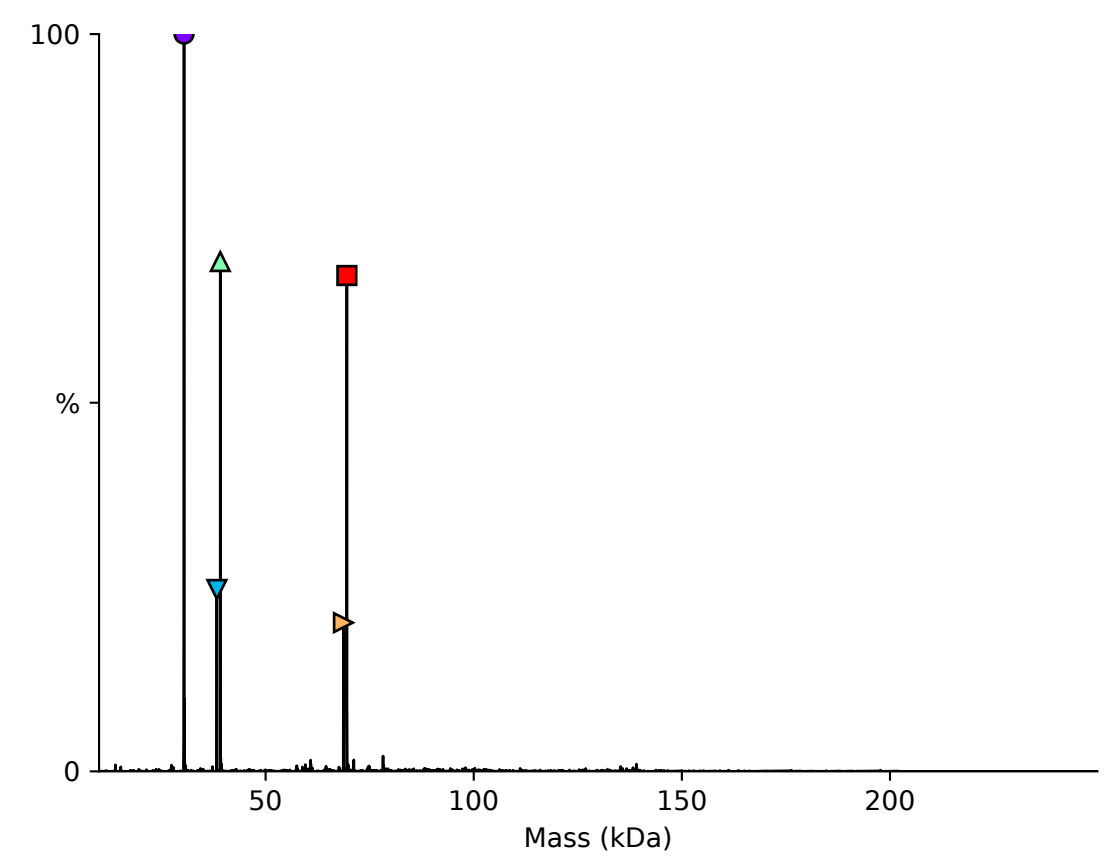

Symbol	Mass (Da)	Intensity[1]	Expected	Difference (Da)	Name/Species/Oligomer
○	30404	100	30416	−12	1[RK1012-A]
▽	38290	25	39127	−837	
△	39113	69	39127	−14	1[RK1012-B]
▷	68702	20	69543	−841	
□	69524	67	69543	−19	1[RK1012-B] 1[RK1012-A]

[1]Intensity values should not be considered quantitative

A3B3-hetRTR05_11noCys

Laboratory: Wysocki-SC, SML
Client: Ryan Kibler
Shipment Date:
Data Recorded: Thu, 18 Nov 2021, 14:04
Instrument: Q Exactive UHMR Orbitrap
Study: OBE 200 mM AmAc, RO33
Sample Conditions: 25 mM Tris, 300 mM NaCl, pH 8
Concentration (ug/uL): 0.8
Injection (uL): 1.25
Comment: Hexamer and dimer present. Hexamer is a little messy, but it appears to be there.
Date Analyzed: Fri, 19 Nov 2021, 14:07 Eastern Standard Time
File Name: session (10)

Quality Check:
Average Peaks Score: 0.86

Expected Oligomers:
Passed:
Mixed:
Failed:

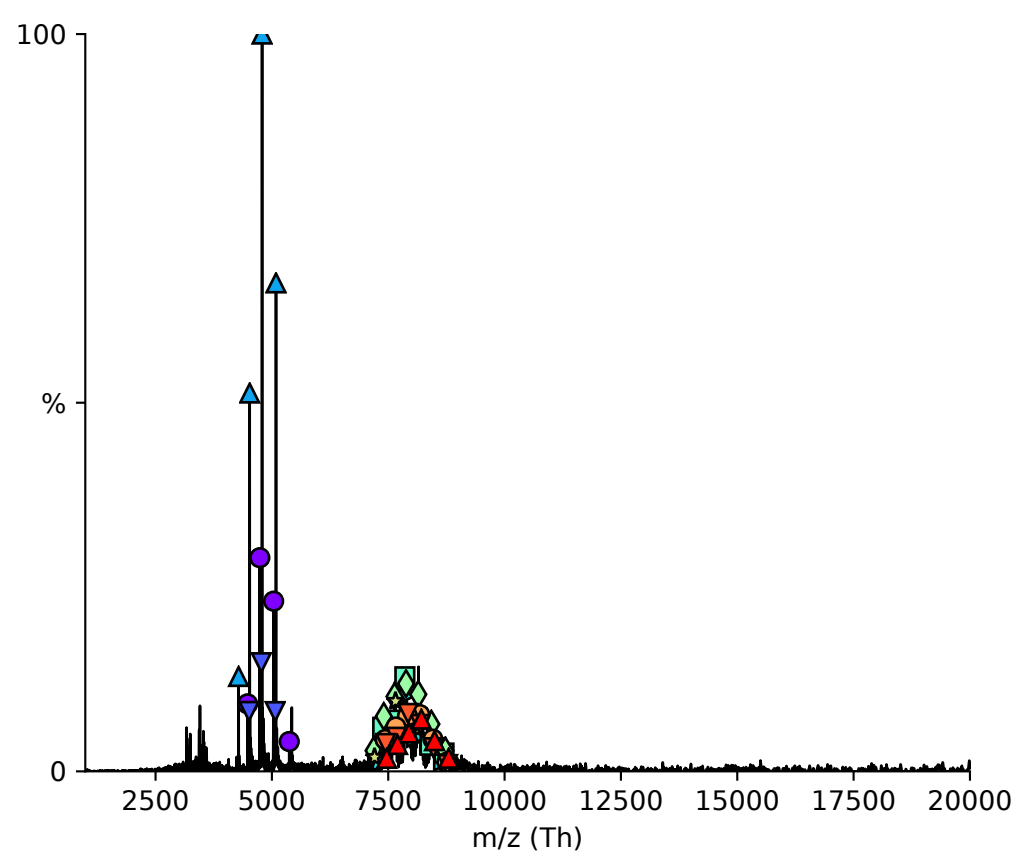

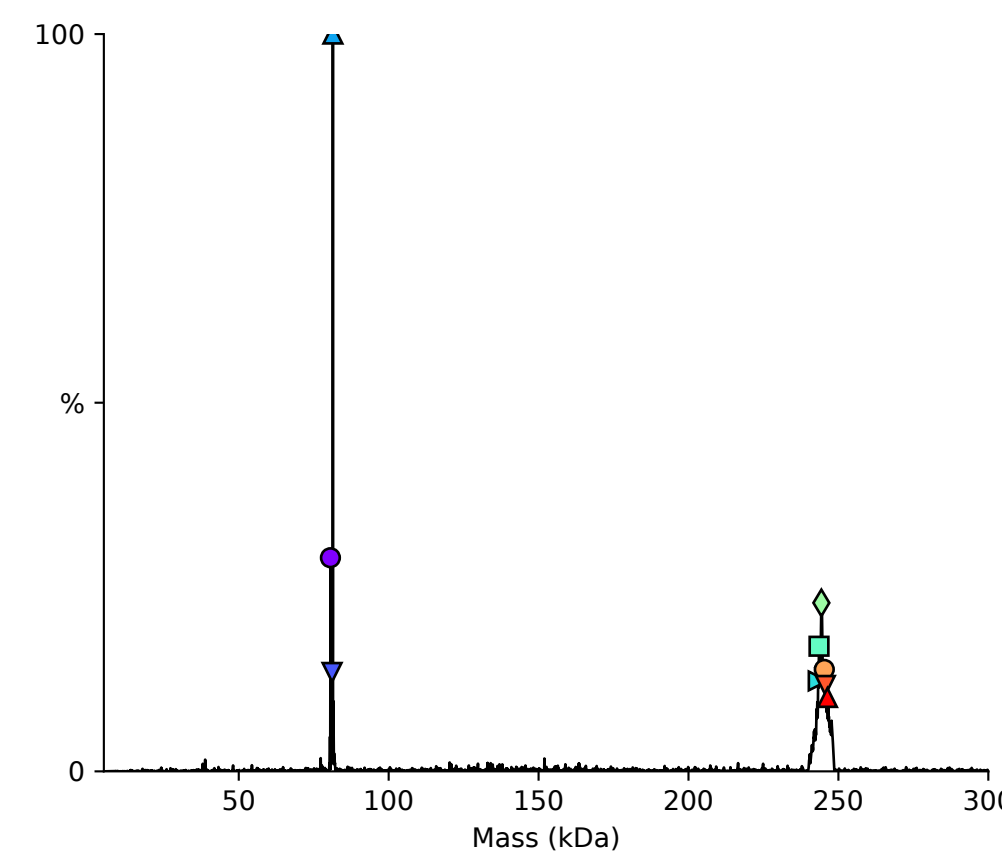

Symbol	Mass (Da)	Intensity[1]	Expected	Difference (Da)	Name/Species/Oligomer
○	80572	29	81417	−845	
▽	81120	13	81417	−297	
△	81395	100	81417	−22	1[RK1043 + RK1044]
▷	243253	12	244251	−998	
□	243533	17	244251	−718	
◊	244319	23	244251	68	3[RK1043 + RK1044]
☆	244966	14	244251	715	
○	245295	14	244251	1044	
▽	245643	12	244251	1392	
△	246337	10	244251	2086	

[1] Intensity values should not be considered quantitative

A3B3-hetRTR11noCys_24noCys

Laboratory: Wysocki-SC, SML
Client: Ryan Kibler
Shipment Date:
Data Recorded: Wed, 24 Nov 2021, 13:16
Instrument: Q Exactive UHMR Orbitrap
Study: OBE 200 mM AmAc, RO33
Sample Conditions: 25 mM Tris, 300 mM NaCl, pH 8
Concentration (ug/uL): 0.4
Injection (uL): 2.5
Comment: Think I found the monomers in here, but the dimer mass is off by about 6 kDa.
Date Analyzed: Wed, 24 Nov 2021, 13:27 Eastern Standard Time
File Name: session (31)

Quality Check:
Average Peaks Score: 0.75

Expected Oligomers:
Passed:
Mixed:
Failed:

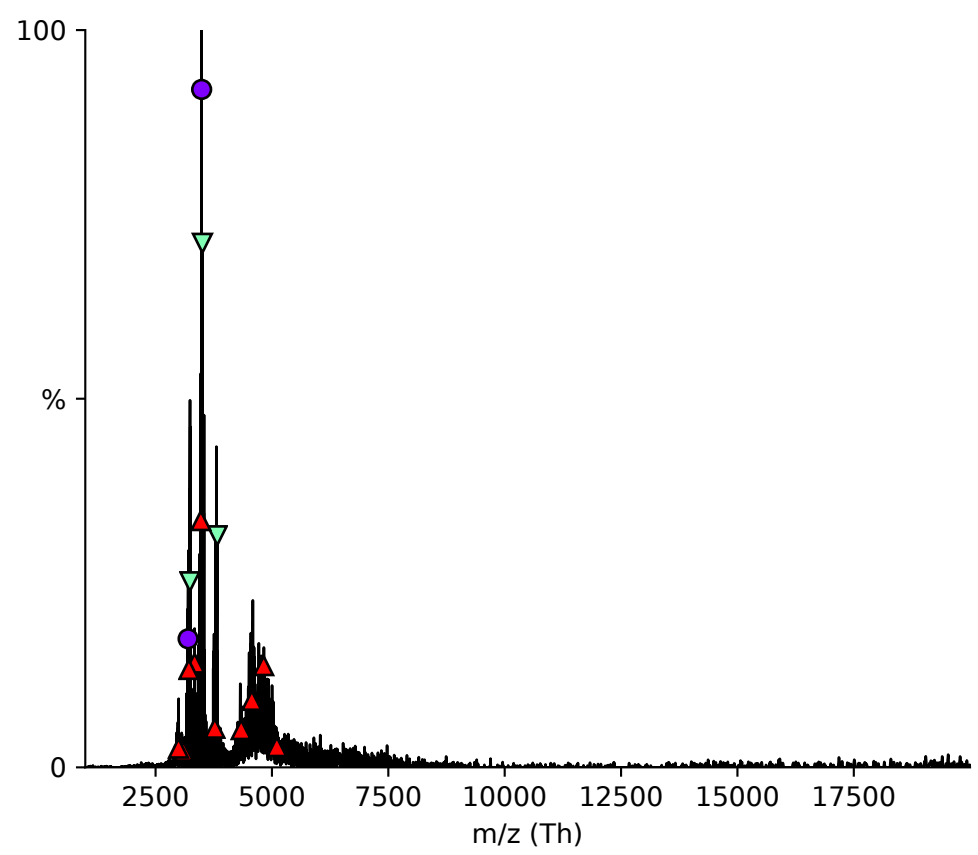

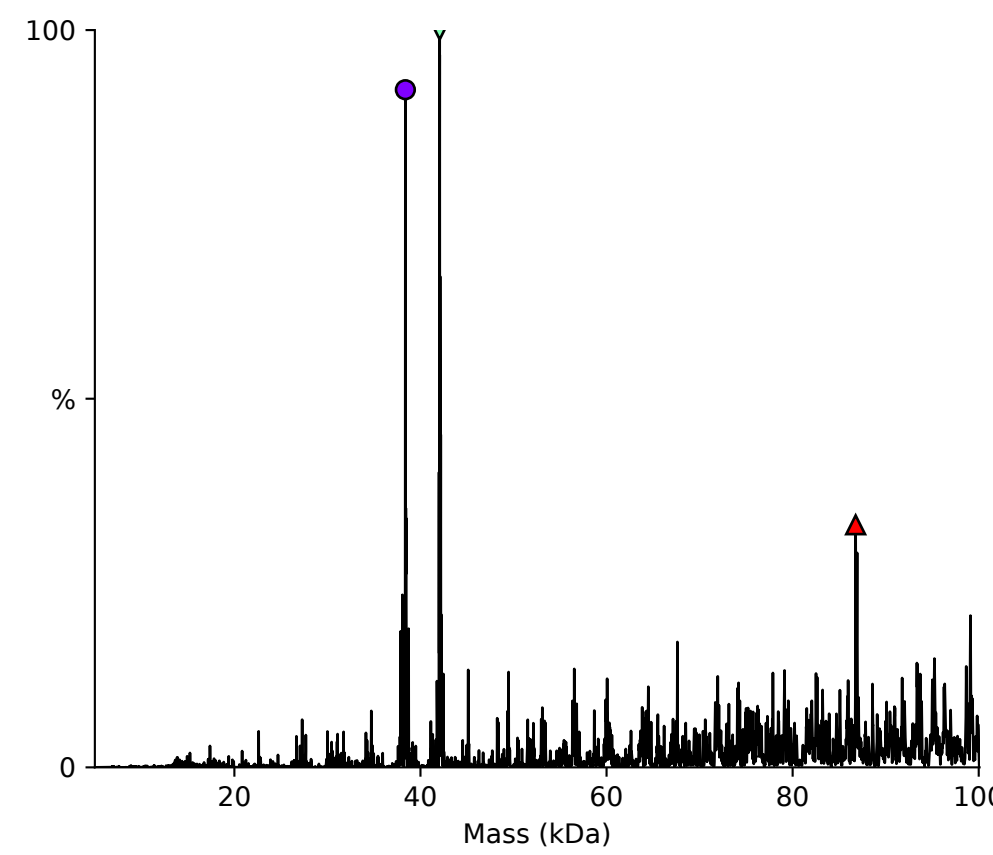

Symbol	Mass (Da)	Intensity[1]	Expected	Difference (Da)	Name/Species/Oligomer
○	38377	92	38532	−155	1[RK1057]
▽	42067	100	42082	−15	1[RK1058]
△	86771	33	80614	6157	

[1]Intensity values should not be considered quantitative
