# Supplementary material for: Design of pseudosymmetric protein hetero-oligomers
Source: Nat Commun. 2024 Dec 18;15:10684. doi: 10.1038/s41467-024-54913-8 (PMC11655659; doi:10.1038/s41467-024-54913-8)
Supplement: Supplementary file 2 [file 41467_2024_54913_MOESM16_ESM.pdf]

Corresponding author(s): David Baker

Last updated by author(s): Nov 4, 2024

Reporting Summary

Nature Portfolio wishes to improve the reproducibility of the work that we publish. This form provides structure for consistency and transparency in reporting. For further information on Nature Portfolio policies, see our Editorial Policies and the Editorial Policy Checklist.

Statistics

For all statistical analyses, confirm that the following items are present in the figure legend, table legend, main text, or Methods section.

n/a	Confirmed	
☐	☒	The exact sample size (*n*) for each experimental group/condition, given as a discrete number and unit of measurement
☒	☐	A statement on whether measurements were taken from distinct samples or whether the same sample was measured repeatedly
☒	☐	The statistical test(s) used AND whether they are one- or two-sided<br>*Only common tests should be described solely by name; describe more complex techniques in the Methods section.*
☒	☐	A description of all covariates tested
☒	☐	A description of any assumptions or corrections, such as tests of normality and adjustment for multiple comparisons
☐	☒	A full description of the statistical parameters including central tendency (e.g. means) or other basic estimates (e.g. regression coefficient) AND variation (e.g. standard deviation) or associated estimates of uncertainty (e.g. confidence intervals)
☒	☐	For null hypothesis testing, the test statistic (e.g. *F*, *t*, *r*) with confidence intervals, effect sizes, degrees of freedom and *P* value noted<br>*Give P values as exact values whenever suitable.*
☒	☐	For Bayesian analysis, information on the choice of priors and Markov chain Monte Carlo settings
☒	☐	For hierarchical and complex designs, identification of the appropriate level for tests and full reporting of outcomes
☒	☐	Estimates of effect sizes (e.g. Cohen's *d*, Pearson's *r*), indicating how they were calculated

Our web collection on statistics for biologists contains articles on many of the points above.

Software and code

Policy information about availability of computer code

Data collection	Rosetta 2018.41.post.dev+171.bcov.dev.507725abe46, DeepAccNet a54150749c80c9a9395c0900d91d5c88b4f54d76,Cytiva Unicorn 7, Thermo Scientific EPU 3.1
Data analysis	Python 3.8.2, PyMol 2.5.0, UniDecs 4.1.2, Bioconfirm, CryoSPARC v3, FoXS from IMP 2.12, HKL2000, Aimless, PHENIX, COOT, Wyatt Technologies ASTRA, TM-align (Version 20220412), ScÅtter IV

For manuscripts utilizing custom algorithms or software that are central to the research but not yet described in published literature, software must be made available to editors and reviewers. We strongly encourage code deposition in a community repository (e.g. GitHub). See the Nature Portfolio guidelines for submitting code & software for further information.

Data

Policy information about availability of data

All manuscripts must include a data availability statement. This statement should provide the following information, where applicable:

- Accession codes, unique identifiers, or web links for publicly available datasets
- A description of any restrictions on data availability
- For clinical datasets or third party data, please ensure that the statement adheres to our policy

The X-ray crystallography coordinates and structure files data generated in this study have been deposited in the Protein Data Bank under the following accession codes: 8E0L [https://doi.org/10.2210/pdb8E0L/pdb] (BGL06), 8E0M [https://doi.org/10.2210/pdb8E0M/pdb] (BGL15), 8E0N [https://doi.org/10.2210/pdb8E0N/

pdb] (BGL18), 8E0O [https://doi.org/10.2210/pdb8E0O/pdb] (hetBGL03-15-18), and 8E12 [https://doi.org/10.2210/pdb8E12/pdb] (BGL14_styr). Coordinates of BGL0 and structure files used in this study are available at the Protein Data Bank with accession code 6XR2 [https://doi.org/10.2210/pdb6XR2/pdb]. The design models tested in this study have been deposited to Zenodo63 with accession code 7851257 [https://doi.org/10.5281/zenodo.7851258]. The SAXS, SEC-MALS, SEC, and nMS data generated in this study have been deposited to Zenodo64 with accession code 7853171 [https://doi.org/10.5281/zenodo.7853172]. The raw OBE MS data generated in this study have been deposited to MassIVE65 under accession code MSV000092722 [https://doi.org/doi:10.25345/C5599ZC1K] (Raw data files correspond to the file names given in the reports in Supplementary Data 13). Source data are provided with this paper.

Research involving human participants, their data, or biological material

Policy information about studies with human participants or human data. See also policy information about sex, gender (identity/presentation), and sexual orientation and race, ethnicity and racism.

Reporting on sex and gender	*Use the terms sex (biological attribute) and gender (shaped by social and cultural circumstances) carefully in order to avoid confusing both terms. Indicate if findings apply to only one sex or gender; describe whether sex and gender were considered in study design; whether sex and/or gender was determined based on self-reporting or assigned and methods used.*<br>*Provide in the source data disaggregated sex and gender data, where this information has been collected, and if consent has been obtained for sharing of individual-level data; provide overall numbers in this Reporting Summary. Please state if this information has not been collected.*<br>*Report sex- and gender-based analyses where performed, justify reasons for lack of sex- and gender-based analysis.*
Reporting on race, ethnicity, or other socially relevant groupings	*Please specify the socially constructed or socially relevant categorization variable(s) used in your manuscript and explain why they were used. Please note that such variables should not be used as proxies for other socially constructed/relevant variables (for example, race or ethnicity should not be used as a proxy for socioeconomic status).*<br>*Provide clear definitions of the relevant terms used, how they were provided (by the participants/respondents, the researchers, or third parties), and the method(s) used to classify people into the different categories (e.g. self-report, census or administrative data, social media data, etc.)*<br>*Please provide details about how you controlled for confounding variables in your analyses.*
Population characteristics	*Describe the covariate-relevant population characteristics of the human research participants (e.g. age, genotypic information, past and current diagnosis and treatment categories). If you filled out the behavioural & social sciences study design questions and have nothing to add here, write "See above."*
Recruitment	*Describe how participants were recruited. Outline any potential self-selection bias or other biases that may be present and how these are likely to impact results.*
Ethics oversight	*Identify the organization(s) that approved the study protocol.*

Note that full information on the approval of the study protocol must also be provided in the manuscript.

Field-specific reporting

Please select the one below that is the best fit for your research. If you are not sure, read the appropriate sections before making your selection.

☒ Life sciences ☐ Behavioural & social sciences ☐ Ecological, evolutionary & environmental sciences

For a reference copy of the document with all sections, see nature.com/documents/nr-reporting-summary-flat.pdf

Life sciences study design

All studies must disclose on these points even when the disclosure is negative.

Sample size	Sample size was determined by the number of protein samples that it was practical to purify at a time (~24) for each of the design groups (BGL, hetBGL, RTR, hetRTR) at the scale of expression and purification employed in this study. Additional samples would have been tested if not enough successful designs were identified during testing.
Data exclusions	No data was excluded from analysis
Replication	All BGL homotrimer constructs, hetBGL constructs, and RTR homo-oligomeric constructs were purified on at least two separate occasions. Those which passed screening were purified at least one additional time. All hetRTR constructs were purified at least once and A2B2-hetRTR05-11, A(5)2B(7)2-hetRTR05-11noCys, A3B3-hetRTR05-11noCys, and A2B2C2-hetRTR11noCys-05-16 were purified twice. All data presented originates from the final purification, though results obtained from earlier purifications were comparable.
Randomization	Randomization was not relevant to this study because we were not studying comparative or conditional effects
Blinding	Blinding was not used in this study because sample triage was needed to focus characterization efforts and thus the sample identities needed to be known.

Reporting for specific materials, systems and methods

We require information from authors about some types of materials, experimental systems and methods used in many studies. Here, indicate whether each material, system or method listed is relevant to your study. If you are not sure if a list item applies to your research, read the appropriate section before selecting a response.

Materials & experimental systems

n/a	Involved in the study
☒	☐ Antibodies
☒	☐ Eukaryotic cell lines
☒	☐ Palaeontology and archaeology
☒	☐ Animals and other organisms
☒	☐ Clinical data
☒	☐ Dual use research of concern
☒	☐ Plants

Methods

n/a	Involved in the study
☒	☐ ChIP-seq
☒	☐ Flow cytometry
☒	☐ MRI-based neuroimaging
