# Supplementary material for: Design of pseudosymmetric protein hetero-oligomers
Source: Nat Commun. 2024 Dec 18;15:10684. doi: 10.1038/s41467-024-54913-8 (PMC11655659; doi:10.1038/s41467-024-54913-8)
Supplement: Supplementary file 3 [file 41467_2024_54913_MOESM17_ESM.pdf]

nature portfolio

Peer Review File

Design of pseudosymmetric protein hetero-oligomers

Corresponding Author: Professor David Baker

This file contains all reviewer reports in order by version, followed by all author rebuttals in order by version.

Version 0:

Reviewer comments:

Reviewer #1

(Remarks to the Author)
The paper by Kibler and colleagues show design and experimental validation of heteromeric proteins. This is not a trivial design task, and even native proteins can adopt unanticipated multimerization – like coiled springs instead of rings. These heteromeric complexes offer an important functionality to bring enzymes together in stoichiometric quantities and positioned optimally to work on a substrate or pathway. This paper is a valuable advance for bioengineering and would be of general interest to Nature Comm readers.

Comments:
1. Were any negative design elements included, to prevent undesired combinations. Or, were only design elements that promote an interface, applied?
2. In figs 2 and 5, the authors show the design juxtaposed to experimental data. For the SAXS, it is difficult to derive any conclusion from the current figs. The data would be much more informative to also look at with real space p(r) curves and for both reciprocal and real space plots, an overlay with the curve predicted from the model. It is also curious that 19 has multiple populations in the main peak, based on looking at the SEC. The authors may also want to document the presence or not of small peaks ahead of the main peak, as it suggests heterogeneous assemblies.
3. The downselection to six models was something of interest, so comparison to the unselected models was desired. However, it was difficult to discern why those 6 models were chosen based on looking at the figures. Notably, supplemental Fig 4 does not include all models, as stated in the figure legend. For future reference, it would be good to have a table with the protein(s) and what analyses were done and a summarized result.
4. For SAXS, the paper is missing Guinier plots and a standard SAXS table, including SAXS-calculated MW and theoretical MW. In case where assembly is changing faster than the SEC run, it would be helpful to compare SAXS curves not just with the model but run mixtures with aggregated or single subunits, to eliminate that contribution to the scattering. Aggregation potential is suggested from the SEC chromatograms. Please include the Porod exponent, as it may be correlated to resolution.
5. For the crystal structures in Fig. 3 and 4, how was the phasing done - were the design models used for the molecular replacement and initial model building. If so, how are the authors sure that there isn't a model bias? Were the higher resolution models used as a restraint – which might improve the electron density for the low resolution???
6. For the heterotrimers, were symmetric multimers observed?
7. Fig. 5 was confusing because it looked like there was discussion of nsEM, but then they say that certain features that they observed were not observed in nsEM. By what were they observable? Clarification would be helpful.
8. Given the complexity of the RTRO profiles, SEC-coupled SAXS is needed. Analysis by programs such as RAW, to separate out components, will improve the confidence in the data, as it would appear that the bulk SAXS measurement will have a mixture of conformers. Again SAXS MW would be helpful to know. It also would be important to know SAXS consistency with the design models, as what happens in crystal or on the surface of a grid may not be occurring in solution.
9. Check for obvious spelling and punctuation errors.
10. As a suggestion as this is outside of the scope of this paper, it would be good to elaborate on the potential applications in case it prompts new applications.

Reviewer #2

(Remarks to the Author)
In this article Kibler et al. computationally design and experimentally validate hetero-oligomeric cyclic proteins. This is an

outstanding challenge for protein design as multiple orthogonal quaternary protein interactions have to developed. Taking inspiration from nature, where genes can duplicate and subsequently diversify, Kibler et al. design two sets of homo-oligomeric proteins based on the structure of two previously designed homomeric protein scaffolds. The cyclic symmetry of the scaffolds mean that each monomer has two unique interfaces that can be altered to change specificity for other monomer units. The homo-oligomers are robustly characterised, and the sequence at the N-termini and C-termini from various successful designs can be combined to form hybrid monomer units containing heteromeric interfaces. These hetero-oligomers are experimentally characterised and successful designs are identified.

This work is original and novel, with the authors making an effort to outline prior art in the field for the design of hetero-oligomer proteins. This is a well-written article, containing only minor typographical errors, that was a joy to read. This work broadly supports all their claims, with good data provided, and reasonable interpretation of the data. Below, I will list recommendations to the authors that, when addressed, should strengthen the article and make it publishable.

Major Recommendations

1. Kibler et al. have made a reassuring and quite impressive effort to use four orthogonal techniques to measure the oligomeric state of the BGL and RTR monomers, namely SEC-MALS, SAXS, nMS and nsEM, and have made equal efforts with the hetBGL proteins. It is, therefore, surprising that the same comprehensive analysis has not been performed on HetRTR constructs. It is entirely possible that I have missed this data, but could the authors explain why SAXS and SEC-MALS data are not provided and discussed for the HetRTR constructs, and why nsEM data has only been presented for A2B2-hetRTR05-11 and A52B72-hetRTR05-11noCys? This is particularly concerning given the heterogeneity of many designs by SEC as pointed out by the authors, i.e. “Many of the designs had multiple peaks on SEC (Fig. S18)” and the presence of some homo-oligomers in some of the nMS. Could the authors either run additional SEC-MALS and/or SAXS on the successful HetRTR designs, or clearly demonstrate why SEC-MALS, SAXS and nsEM were not performed to give the same rigor in analysis as the other successful designs?

Minor Recommendations

1. In the introduction the authors describe prior art as follows “However, while there has been considerable progress with de novo design of protein homo- and hetero-oligomers9–22, with the exception of heterodimers18 and coiled coils 16,20–22…”. I have two comments related to this. Firstly, I agree that their approach is novel and quite different, but this sentence also implies that their designs are not coiled coils, when in fact their designs are based on helical bundles showing coiled-coil character. Perhaps the sentence could be rephrased slightly to address this? Secondly, the authors should also acknowledge that heterotrimeric collagen have been designed too, e.g. Heterotrimers have also been achieved with Collagen - Hydroxyproline-Free Single Composition ABC Collagen Heterotrimer (doi: https://doi.org/10.1021/ja402187t)
2. The rational for choosing designed cyclically symmetric proteins is clear, however no rational is given for specifically choosing tcTRP9_sub3 & C4_1 as starting scaffolds. It is especially surprising given that tcTRP9_sub3 (now BGL0) has poor biophysical behaviour based on the SEC and nMS data presented. Could the authors provide an additional comment on their selection in the manuscript or SI?
3. I appreciate that in order to summarise a large set of designs in a concise fashion, the authors present the results in the form of success rates, and explicitly name and discuss some successful designs. In doing so, however, it can be difficult to identify which designs were considered a success and according to which techniques were some designs deemed a failure. This is especially true as there are multiple figures and supplementary figures to follow for each design. This could be remedied if the authors could provide a summary table of all designs that summarises the results of each technique.
4. HetBGL constructs were co-expressed and copurified, whereas hetRTR monomers were individually expressed, purified and subsequently mixed. Could the authors explain the reason for the difference in protocol? Are the HetBGL monomers stable/soluble in isolation? If unknown, could the authors explain in the manuscript why they don’t think testing this is relevant?
5. In addition to changing the helix composition of A and B in A52B72-hetRTR* complexes, the constructs lack the GFP fusion of the A2B2 designs. If they haven’t already, could the authors state why in the manuscript or SI?
6. One paragraph describes “A2B2 heterotetramers containing the RTR05 or RTR16 interfaces, which as homo-oligomers partially formed pentamers, did not contain any off-target pentameric species, suggesting that the added specificity constraints of heteromeric interactions may prevent off-target assemblies.” However, I couldn’t see a discussion of RTR16 forming successful hetero interfaces earlier in the paragraph.
7. In one section starting “Each subunit of a heterotrimer from Set 1 has a different number of helices…” the authors describe converting HR-N back to HR-C. Could the authors state in the manuscript the rational for doing this, given the effort in designing HR-N constructs in the first place?
8. I think it is reasonable to only continue biophysical characterisation on promising designs. For the authors’ BGL homomers, I assume based on the number of proteins characterised that SEC and SAXS were conduct first, followed by nMS, and lastly SEC-MALS. However, proteins analysed by SEC-MALS are not all represented in the nMS set and vice versa (nMS BGL01, 02, 03, 05, 06, 07, 08, 09, 11, 12, 14, 15, 17; and SEC-MALS – BGL01, 02, 03, 05, 09, 12, 14, 15, 17, 19, 20). Could the authors explain the reason for this?
9. The outer shell high-resolution statistics for the X-ray crystal structures of BGL06 and hetBGL03-15-18 suggest that the crystals diffracted to a higher resolution (outer shell CC1/2 0.998 and 0.969, and I/sigI of 16.2 and 6, respectively). Could the authors explain their reasoning for trimming the data set to a lower resolution?

Reviewer #3

(Remarks to the Author)
Baker and colleagues present a neat paper of generating heteromeric protein assemblies. It is true that general approaches for generating such structures don't really exist beyond coiled coils. The approach the authors take is clever and starts from naturally occurring multimers that are first redesigned to introduce asymmetric interaction followed by slitting and recombination of the asymmetric fragments. I'm not sure how general this approach will be, but it certainly adds to the available tools and, overall, I'm supportive of publishing this work in Nat. Commun. The authors present two examples of this approach, in one case they even succeeded in crystallographic characterization of a heterotrimer, which is a big plus. The rest of the characterization is predominantly mass spectroscopic. Overall, the data supporting the claims are solid, however I should mention here that the data shows fairly large percentage of higher order aggregation species – this isn't a major problem per se, but it raises question about how specific this approach really is. It's quite hard to put a quantitative number as to how big the fraction of non-specific/designed association is, but I'd say it is not insignificant given the presented gel filtration/MS data. I won't request any additional experiments – it's a hard problem to address, but the authors should reflect it in the discussion.

Reviewer #4

(Remarks to the Author)
The manuscript submitted by Kibler and colleagues describes the successful design of pseudosymmetric hetero-oligomers which can be useful for further applications. I consider that the work has potential interesting results to be published in Nature Communications, mainly related to the challenge of accurately designing multiple protein interfaces simultaneously and their extensive experimental characterization. However, multiple experimental aspects and discussions should be addressed before recommending the paper for publication:

• Even though the manuscript shows interesting results, the way how is written and the results shown are confusing for some moments when reading. Since the authors have an extensive amount of constructs and experimental data, I strongly recommend adding a summary table that lists all the proteins discussed in the manuscript (dividing them into the different groups) and then indicates the different techniques i.e. SEC, SEC-MALS, SAXS, nMS, nsETM and what's the output of each of them (homooligomer, trimer, tetramer, multiple species, etc…) in comparison with the intended oligomeric state. I think this summary table will be extremely helpful for the readers to follow all the storylines and what are the best designs of the different groups.
• I find it difficult to follow the overview shown in figure 1, maybe adding point descriptions can help.
• I consider that all the evolutionary hypotheses/phrases related to the ancestry of the hetero-oligomers should be removed since this's not the topic of this work and also I don't see clear support for these discussions.
• SEC-MALS results should be revisited. The way they're presented is not completely adequate and not the expected plotting quality usually reported (especially for the type of conclusions the results have to support). First, plots in Suppl. Figure 4 should be replotted to indicate the molecular weight curve and see its dispersion along the elution peaks. Curves close to 20 min of retention time can be deleted since this information corresponds to the buffer signal. Also, the authors have to discuss the unusually high errors in their measurements (probably due to improper baseline selection or peak broadening/alignment). Authors should report the polydispersity for all the measurements and make clear if they report the mean MW or by peak. In BGL12, MW is indicated as 5.24 kDa, I assume it's a mistake (also, a second peak is observed and the MW of this peak is not reported). Would be better if the authors plot again the results and indicate on a right Y axis the MW for the elution peaks. To better compare with the SEC elution profiles, the X-axis has to be reported in elution volume (mL) and not in time (min).
• I suggest Figure 2b be replaced with the equivalent results from SEC-MALS. That technique is more informative about the oligomerization state and MW in comparison to a normal SEC profile. The same applies to Figure 5b.
• There are multiple SEC-MALS measurements that are missing in the Suppl. Figure 4 (number: 04, 06, 07, 08, 10, 11, 13, 16, 18). Is there any special reason for that? For many of them, it's important to report the data since other measurements are reported (SEC, MS, SAXS…) and would be useful to compare the behavior of the designs with different techniques. For example, it's intriguing why are not showing the results for BGL06 but a three-dimensional structure is reported. Authors have to show all the data for completeness.
• In addition, why SEC-MALS measurements were not collected for hetBGLs? This would be relevant to discuss monodispersity and proper MW of the heterotrimers. Related to that, I don't agree with the authors when they stated “largely monodisperse and had the expected retention volume” since the SEC profile shown in Figure 4 presents multiple peaks (and asymmetry) which is a clear indication of polydispersity. If they want to show monodispersity, then SEC-MALS experiments should be collected.
• I found unclear how was calculated the stoichiometry discussed on Suppl. Figure 6. Especially when extra bands are present like in BGL03-06-05, BGL08-19-09, and others. Authors have to clarify this calculation and their classification between uneven stoichiometries and “approximately” equal stoichiometries. This is especially important because they stated “Protein size affects band staining, so it is difficult to judge stoichiometry from the band darkness/size, but the correct trend in stain density is observed for equal stoichiometry”, then discussing stoichiometries is quite tricky with this approach.
• In general, would be important that the authors discuss their design failures and the discrepancies observed among all the methods. For example, heterotrimers show a clear sign of a mixture of different oligomeric states; for me, it's not convincing that the elution profiles shown in Suppl. Figure 7 are monodisperse as the state in the manuscript, most of them show multiple and asymmetric peaks. Also, the difference in the elution for 03-05-06 and 06-05 is visible, in addition to the extra observed peaks in the elution profiles. SEC-MALS can give further information about their behavior in solution.
• Throughout the manuscript, when authors compare models and structures, they should provide the number of Ca that were compared in the RMSD calculation (text and figure legends).
• I find a bit loose this argument: “the three distinct subunits have an average pairwise sequence identity of 62.3% but high structural similarity (aligned CA-RMSD of 1.5 Å), showing that this method can produce highly (pseudo)symmetric hetero-

oligomers." Could the authors explain what they meant by this?
• SAXS results shown in Figures 2 and 5 should also show the comparison of experimental results with the computed profiles. In addition, many of the SAXS plots show a clear discrepancy between experimental data and model prediction. I think would be informative discussing a bit about these issues since can be helpful to the readers related to data interpretation.
• BGL13 SAXS measurements are missing in the Suppl. Figure 5.
• Regarding the homotetramers, the authors stated: "For 10/24 redesigns, SEC elution profiles suggested a single homogenous species with the target subunit composition (Fig. 5b, Fig. S13)". It's unclear what are those designs and again, to say that they elute like tetramers, SEC-MALS would be helpful in addition to their MS data, which also would help to clarify the degree of polydispersity.

Minor issues:

• Repot cut-off criteria of the crystallographic data. For a couple of structures is not clear why that resolution cut-off was used.
• Why is there a discrepancy between the resolution reported in data collection and refinement for BGL15 structure?
• In addition, Matthews coefficient Vm also should be included in the table; would be important to show the number of molecules in the ASU since this is relevant for heterodimer formation.
• In the methods, the authors didn't say if they pooled all the elution fractions from SEC for further experiments. This might be important if the proteins were showing multiple oligomeric states during the protein preparation/purification.
• With the extensive number of constructs and possible difference of isoelectric points, did the authors consider that some of the failed designs were due to improper pH during purification, i.e. that buffer was not adequate and protein aggregation was due to the pH being too close to the pI?

Reviewer #5

(Remarks to the Author)
The manuscript described a systematic study for designing hetero-protein complexes. This was achieved by first changing subunit interfaces within homocomplexes. Then chimeric subunits were made by fusing halves of two distinct subunit as new subunits, allowing hybrid complexes to assemble in specific ways. The group utilized multiple high throughput screening assays to check the products, selected the successful candidates for the next step in the design. It is a very interesting demonstration of protein design strategy, with lots of data being openly shared for reference by the scientific community. Overall, it is a sound study but require further clarification in writing, particularly related to the interpretation of the experimental data and the integration with the discussion of protein design. I have several major comments:

1. The authors selected different assays at different stages. Higher throughput methods generally had more complete datasets. The best ones were sent to crystallography at the end for detailed interface analysis. The data from different methods did not seem to all overlap perfectly. What is the complementarity of these assays? It is recommended to discuss how the data from different methods were used to drive the decisions (i.e., what is considered good hit or not). Was there any (semi-)quantitative analysis involved? For example, in FigS2 and S3, BGL07 seems to fall apart in native MS but not in SEC.

2. Related to the comment above, nMS appeared to miss data for some samples while SEC data appeared to be more complete (e.g., BGL04, 10, 13,16. BGL20 was only shown in Table S7). Are some of them simply considered bad and discarded? If so, do these "bad" results correlate with other assays and indicate aggregation, etc? Are the seemingly negative data informative in some way for the design?

Other minor comments:

3. The nMS data processing workflow seems to work well for high quality spectra, but not always for noisy spectra. For example, hetBGL0-18-17, hetBGL0-18-19, hetBGL0-19-17, hetBGL0-19-18 seem to be not picking up the peaks around 7000 m/z. BGL0 seems to have extra peaks next to the base peak that may be artifacts. Maybe some spectral smoothing is needed to minimize artifacts in the deconvolution. While these details do not change the main conclusions of the paper, the spectral details may speak to the properties of the proteins.

4. I recommend better clarifying the potential biological applications related to polyhedral assemblies, nanocages for readers to better understand the motivation. This may not be obvious to readers outside the immediate field of expertise. Current design assumes protein core regions are identical and only interfaces were changed. This may not apply to enzymes that have active site in the core and cannot be easily re-engineered. For structural proteins the downstream applications are easier to relate to. Nonetheless, it is better to comment on how this strategy can be broadly applied to design complexes that have sensitive structures in the core of the protein subunits.

5. nMS data for heterocomplex Fig S8 and Fig 4 may be better displayed to highlight the homogeneity of the trimers, i.e., absence of other combinations of trimers. This is a unique contribution for nMS compared to other assays used. It is not obvious what mass differences are expected for ABC vs AAB, etc, and if they are readily resolved and/or quantifiable in nMS spectra with the full range spectra shown in the figures.

6. Some additional peaks in nMS data were labeled with mass shifts not explained (for example hetBGL03-15-18_reloop, hetBGL08-09-19_reloop, RTR04_C4, RTR0_C2, RTR0_C2, etc). Were they from miss-assembled complexes or just from

adducts/truncations/PTMs? Were those extra species being considered as penalties in the design optimization?

Version 1:

Reviewer comments:

Reviewer #1

(Remarks to the Author)
The authors have added in additional data that I have requested. The additional SAXS data in Tables S3, S7, and S10 illustrate the complexity and difficulty of quality assessment of the design molecules. The experimental result summary is meant to address this in an unbiased fashion, but the decision points are based on single values, while the curves that have more information content are not included.

In looking at the SAXS data, paper's threshold of similarity is a concern. If this was a natural protein, then the differences observed in every curve was substantially different than the model, particular when looking at the p(r) where even the peak is shifted suggesting global changes. As this is a de novo designed protein and the crystallography and EM are showing similarity, then the question is whether the same standard be applied? Is it sufficient to be similar, as in an oversimplified analogy, a sphere is to an egg?

Nonetheless, the SAXS data is the ground truth, albeit obscure. Typically, higher resolution analysis is considered decisive in its validation of the model, but can introduce biases from solution/grid conditions and from filtering by crystallization or by single molecule analysis. In contrast, the SAXS solution experiment is a sum of all molecules in solution, and may be an important predictor on what stoichiometric or conformational state for the conditions in which the protein will be used. And here, the differences revealed in the SAXS data is informative and insightful. SAXS data is not simply validating the protein design but providing additional features that is intrinsic to the design.

The authors need to include analysis of the real space SAXS curves when considering whether the SAXS curves truly validate their model.

For example, in the first two SAXS data tables, every protein is larger in solution than the predicted structure or the crystal structure, implying that although the crystallography has indicated that the topology is correct, the protein is expanding in solution and may be more flexible. This flexibility, if true, may be a useful feature. Flexibility can be indicated from the SAXS data using the Porod exponent or the dimensionless Kratky comparison to well-folded proteins. Flexibility may also be critical in permitting non-ideal geometry in the larger order assemblies.

In the third SAXS table, most solution data were not consistent with their respective atomic models. For many, this difference is likely due to problems in stoichiometry at the time that the data was collected. The authors state that they do not want to overinterpret their data using mixed populations, but if that is indeed the case in their actual sample, then it seems that including subunit models is the only way not to overinterpret their data. It is also a way to eliminate subpopulations that do not exist. The SEC reflects the propensity of the design protein to form subpopulations in equilibrium, acting to indirectly support the analysis and reduce the charge of overinterpretation. Interpretation of the SAXS data is needed for every one of these curves. For example something quite strange is happening in RTR24_DHR62.

Additional comments

1. Connecting the molecules being discussed in the main text with what is in the supplemental sections needs to be clarified. It would also be helpful if the authors explicitly call out which molecule that they are referring to, in every place in the main text. For example, the authors state "Our success rate of 24 experimentally-verified hetero-oligomers". It is challenging to trace, even when looking at Tables S2, S6, and S9, which are these 24. The authors tend to count (e.g. three out of 12) than to explicitly list which ones. The supplemental tables summarizing the true/false are critical but even there it is still difficult. Without knowing explicitly which ones that they are referring to, I have guessed from their tables and illustrative examples and it prevents reviewing this work with high confidence.
2. Were the C2 values in the FOXS analysis close to 4, which indicates that the water layer was being stretched erroneously to cover the discrepancy? C2 can lead to lower chi values.
3. The authors use a <30% variability in Rg as a cutoff. This seems overly generous. Typically, sample-to-sample differences are <10%, but 30% allows for different shapes to pass through. Comparison of the p(r) curves show the danger of this. A case in point is BGL14, which is seemingly a good scenario with a change in Rg from 23 to 26. Examination of the p(r) curve indicates that the protein in solution is significantly more elongated than in the prediction.
4. Kratky plot should be shown only to q ~0.3. The reason being is that the higher resolution data is noisier and obscures what happens after the main peak. The curve for a well-folded protein will be more bell-shaped, for a protein with a significant disordered tail, the curve will tail up at higher q; a protein that has global conformational flexibility will go up and decrease at a lower slope than the well-folded protein; a curve for a completely disordered protein will either peak and stay at the peak or even continue to rise. The interpretation of the Kratkys are not given in the table, highlighting the difficulty in extracting this information.
5. The p(r) curves appeared to be normalized based on the maximum value and hightlights changes in the peak position, but

can minimize shape differences. Normalizing based on the area under the curve can bring out features indicative of more elongated structures. The authors should consider both types of curves in their analyses.
6. What were the Porod exponent values?
7. There is no discussion of reduced versus non-reduced in the SAXS data, except for the engineering of the cysteines. It should be included in the main text.

(Remarks on code availability)

Reviewer #2

(Remarks to the Author)
I am satisfied with the changes made to the manuscript and associated documents by the authors. I appreciate the effort made in generating tables to follow design results, and I look forward to seeing future applications of the designs and design protocol.

Best wishes,
Guto Rhys

(Remarks on code availability)

Reviewer #4

(Remarks to the Author)
The authors made a good effort to reply to my and all the other reviewer's comments. I recommend the paper for acceptance in this current version.

(Remarks on code availability)

Reviewer #5

(Remarks to the Author)
The authors have addressed the major questions for reviewer #5.
For reviewer 5 - comment 6: I recommend at least adding some notes in the supporting documents to explain the potential origin of the extra peaks. Chemical modifications, terminal truncations, etc may affect protein function so it is better to inform readers about these possibilities.

(Remarks on code availability)

Open Access This Peer Review File is licensed under a Creative Commons Attribution 4.0 International License, which permits use, sharing, adaptation, distribution and reproduction in any medium or format, as long as you give appropriate credit to the original author(s) and the source, provide a link to the Creative Commons license, and indicate if changes were made.
In cases where reviewers are anonymous, credit should be given to 'Anonymous Referee' and the source.
The images or other third party material in this Peer Review File are included in the article's Creative Commons license, unless indicated otherwise in a credit line to the material. If material is not included in the article's Creative Commons license and your intended use is not permitted by statutory regulation or exceeds the permitted use, you will need to obtain permission directly from the copyright holder.
To view a copy of this license, visit https://creativecommons.org/licenses/by/4.0/

Reviewer #1 (Remarks to the Author):

Comments:
1. Were any negative design elements included, to prevent undesired combinations. Or, were only design elements that promote an interface, applied?

Yes, several negative design elements were incorporated. In the introduction, we mention that hydrogen bond networks (HBNets) are often used as negative design elements to improve interface specificity and talk about their incorporation into the BGL interfaces. We also made larger structural rearrangements to a subset of the BGL designs by permuting the location of the C-terminal helix to induce steric incompatibility between the circularly permuted designs belonging to the HR-N set and the rest of the designs.

We incorporated sequence-level negative design elements into the RTR designs. This consisted of HBNets, as with the BGL designs, and cysteine pair positions, on the hypothesis that the formation of disulfide bonds is geometrically specific enough so as to occur only when the correct interface is formed.

The text in these locations have been updated to more clearly state the reasoning behind the design choices.

2. In figs 2 and 5, the authors show the design juxtaposed to experimental data. For the SAXS, it is difficult to derive any conclusion from the current figs. The data would be much more informative to also look at with real space p(r) curves and for both reciprocal and real space plots, an overlay with the curve predicted from the model.

The SAXS profile overlays presented in figures 2 and 5 only include experimental data and were intended to qualitatively show that the curves of the highlighted redesigns strongly resemble each other. This method was chosen as an unbiased way to present the data, with the only free parameter being a linear scaling factor to account for differences in protein concentration. We recognize that this was not what the text said so we have clarified the text.

We thank the reviewer for the suggestion to investigate the real space curves and to plot a per-design overlay of the predicted curves and have included this analysis in the new supplemental tables containing full SAXS metrics.

It is also curious that 19 has multiple populations in the main peak, based on looking at the SEC. The authors may also want to document the presence or not of small peaks ahead of the main peak, as it suggests heterogeneous assemblies.

The reviewer makes an excellent suggestion. We have expanded paragraph 5 to contain more discussion of alternative species.

3. The downselection to six models was something of interest, so comparison to the unselected models was desired. However, it was difficult to discern why those 6 models were chosen based on looking at the figures. Notably, supplemental Fig 4 does not include all models, as stated in the figure legend. For future reference, it would be good to have a table with the protein(s) and what analyses were done and a summarized result.

The choice to present only six redesigns in Figure 2 was to avoid overwhelming the reader. These six were chosen as representatives for each of the design sets (HR-C, HR-N, and NM). Overall there are 12 we believe have achieved similar levels of validation. We have updated the text to make this more apparent.

We agree that we should have provided a summary of the results for each group of experiments and have added tables for experiments on the homo-oligomeric and hetero-oligomeric BGL and RTR designs (tables S2, S6, S9, and S12). Supplemental Figure 4 (now Figure S7) indeed did contain all the SEC-MALS data we had obtained at the time of initial submission and thus the legend was accurate. Nonetheless, we have collected more data and updated the figure.

4. For SAXS, the paper is missing Guinier plots and a standard SAXS table, including SAXS-calculated MW and theoretical MW. In case where assembly is changing faster than the SEC run, it would be helpful to compare SAXS curves not just with the model but run mixtures with aggregated or single subunits, to eliminate that contribution to the scattering. Aggregation potential is suggested from the SEC chromatograms. Please include the Porod exponent, as it may be correlated to resolution.

We have added new supplemental tables containing the Guinier plots and the requested SAXS metrics (Tables S3, S7, and S10). However, we do not agree that it would be helpful to perform ensemble fits or multistate modeling because it introduces the risk of overfitting to explain poor data; it is better to have a design with experimental data that matches strongly with the theoretical model. Obtaining a fine understanding of how exactly a design failed is outside of the scope of this work. See response to Reviewer 5 comment 2.

5. For the crystal structures in Fig. 3 and 4, how was the phasing done - were the design models used for the molecular replacement and initial model building. If so, how are the authors sure that there isn't a model bias? Were the higher resolution models used as a restraint – which might improve the electron density for the low resolution???

The data was phased via molecular replacement with the original design models, which were modified prior to those computations by removing all newly incorporated side chains in each of the redesigned models. Those side chains were then manually rebuilt into unbiased difference density within each subunit interface. That density, in each case, was unambiguous and allowed for straightforward modeling and subsequent refinement. We have updated the text to include this.

6. For the heterotrimers, were symmetric multimers observed?

Based on our nMS data (Fig S10), we did observe "A2B2C2" species in a subset of samples, which is likely to be a dimer of trimers, but we did not observe any homo-oligomeric complexes (e.g. AAA, BBBB). We cannot confidently say if any of them are symmetric or not without structural characterization.

7. Fig. 5 was confusing because it looked like there was discussion of nsEM, but then they say that certain features that they observed were not observed in nsEM. By what were they observable? Clarification would be helpful.

We believe that the reviewer is referring to the discussion in paragraph 12 about RTR05 and RTR16 containing both pentamers and tetramers according to nsEM (negative stain electron microscopy) but not detecting pentamers by nMS (native mass spectrometry). We believe this misunderstanding stems from a misreading "nMS" for "nsEM". We have clarified the text in this section to avoid confusion. Upon review of the nMS data, we concluded that nMS did detect pentamer in RTR05 (but not RTR16) and have corrected the text.

8. Given the complexity of the RTRO profiles, SEC-coupled SAXS is needed. Analysis by programs such as RAW, to separate out components, will improve the confidence in the data, as it would appear that the bulk SAXS measurement will have a mixture of conformers. Again SAXS MW would be helpful to know. It also would be important to know SAXS consistency with the design models, as what happens in crystal or on the surface of a grid may not be occurring in solution.

The SAXS data we collected on the non-reduced homotetrameric RTR redesigns match well to the theoretical model of the tetramer for many but not all of the designs. We have replaced Fig. S15 with Table S10 which contains full SAXS metrics (including plots) and summary Table S9 to make this comparison easier for the reader.

It is possible for non-tetrameric species or tetramers in alternative conformations to influence the SAXS data. Taking the highlighted redesigns for example, RTR05, RTR11, and RTR16, we observe that RTR05 and RTR16 fit less well to the model of the tetramer (X2=1.14 and 0.78) than RTR11 (X2=0.09), a comparison made under the assumption that the underlying protein structures between designs are the same. This is explained in the negative stain electron microscopy (nsEM) data revealing that RTR11 is purely tetrameric, while RTR05 and RTR16 contain both tetrameric and pentameric species (See Figure S14 for raw micrographs and 2D class averages). Finding this same evidence in the SAXS data would be possible with multistate or ensemble fitting, but this process could be accidentally biased by adding more parameters to fit. While the use of SEC-SAXS may offer a less biased way to analyze this by physically separating species immediately prior to SAXS, this would not be possible with some of the hetRTR designs which were unable to be produced in high enough concentrations for SAXS. Our results indicate that nsEM is the more effective technique for the structural and oligomeric analysis of the whole RTR family (homo-oligomers and hetero-oligomers), so we think it is more

appropriate to focus the discussion on it. To facilitate this, we have removed panel c in figure 5. We have added text to acknowledge the strengths and limitations of nsEM.

9. Check for obvious spelling and punctuation errors.

We thank the reviewer for their suggestion. We will continue to read the text carefully and correct any errors we find.

10. As a suggestion as this is outside of the scope of this paper, it would be good to elaborate on the potential applications in case it prompts new applications.

The reviewer makes an excellent point that discussion of potential applications can inspire new applications. We have expanded the discussion of possible applications in the discussion section.

Reviewer #2 (Remarks to the Author):

Major Recommendations

1. Kibler et al. have made a reassuring and quite impressive effort to use four orthogonal techniques to measure the oligomeric state of the BGL and RTR monomers, namely SEC-MALS, SAXS, nMS and nsEM, and have made equal efforts with the hetBGL proteins. It is, therefore, surprising that the same comprehensive analysis has not been performed on HetRTR constructs. It is entirely possible that I have missed this data, but could the authors explain why SAXS and SEC-MALS data are not provided and discussed for the HetRTR constructs, and why nsEM data has only been presented for A2B2-hetRTR05-11 and A52B72-hetRTR05-11noCys? This is particularly concerning given the heterogeneity of many designs by SEC as pointed out by the authors, i.e. “Many of the designs had multiple peaks on SEC (Fig. S18)” and the presence of some homo-oligomers in some of the nMS. Could the authors either run additional SEC-MALS and/or SAXS on the successful HetRTR designs, or clearly demonstrate why SEC-MALS, SAXS and nsEM were not performed to give the same rigor in analysis as the other successful designs?

We thank the reviewer for their suggestion. As mentioned above in response to Reviewer #1 comment 8, we found nsEM to be considerably more sensitive to oligomeric state and structure than SAXS. Many hetRTRs could also not be produced at sufficient concentration for the other techniques, particularly for the heterohexamers, which effectively limited structural/compositional analysis to only nsEM. We have updated the text to elaborate on our choice to focus primarily on nsEM validation for the hetRTRs, as well as added the SEC traces of the representative hetRTRs to Figure 5 to illustrate the incomplete assembly.

Minor Recommendations

1. In the introduction the authors describe prior art as follows “However, while there has been considerable progress with de novo design of protein homo- and hetero-oligomers9–22, with the exception of heterodimers18 and coiled coils 16,20–22…”. I have two comments related to this. Firstly, I agree that their approach is novel and quite different, but this sentence also implies that their designs are not coiled coils, when in fact their designs are based on helical bundles showing coiled-coil character. Perhaps the sentence could be rephrased slightly to address this? Secondly, the authors should also acknowledge that heterotrimeric collagen have been designed too, e.g. Heterotrimers have also been achieved with Collagen - Hydroxyproline-Free Single Composition ABC Collagen Heterotrimer (doi: https://doi.org/10.1021/ja402187t)

This sentence is referring to design methods relating to the conversion of homo-oligomers into hetero-oligomers and do not relate to the designs themselves discussed later in this work. We will clarify this point.

We thank the reviewer for suggesting the inclusion of heterotrimeric collagen and have corrected this oversight.

2. The rational for choosing designed cyclically symmetric proteins is clear, however no rational is given for specifically choosing tcTRP9_sub3 & C4_1 as starting scaffolds. It is especially surprising given that tcTRP9_sub3 (now BGL0) has poor biophysical behaviour based on the SEC and nMS data presented. Could the authors provide an additional comment on their selection in the manuscript or SI?

We selected tcTRP9 initially because it resembled the interfaces in Chen et al[1] and we had observed good biophysical behavior in our initial work with it[2]. We were surprised to have such difficulty with it in the current work. This difference can possibly be attributed to differences in purification construct design.

>Original (thrombin cleavage)
MASS**HHHHHH**SSG**<u>LVPR/GS</u>**SMG**NLELALKALQILVNAAYVLAEIARDRGNEELLEKAARLAE EAARQAEEIARQARKEGNLELALKALQILVNAAYVLAEIARDRGNEELLEKAARLAEEAARQA EEIARQARKEGNLELALKALQILVNAAYVLAEIARDRGNEELLEKAARLAEEAARQAEEIARQ ARKEG**

>This work (TEV cleavage)
MG**HHHHHH**GS**<u>ENLYFQ/G</u>**WS**LELALKALQILVNAAYVLAEIARDRGNEELLRKAARLAEEAA RQAEEIARQARKEGNLELALKALQILVNAAYVLAEIARDRGNEELLEKAARLAEEAARQAEEI ARQARKEGNLELALKALQILVNAAYVLAEIARDRGNEELLEKAARLAEEAARQAEEIARQAR KEGN**

His6-tag in blue, cleavage site underlined in red (cleavage location indicated with a “/”), and structural sequence in green. There are three differences in the expression construct design.

1. *Cleavage site: The original used a thrombin-cleavable sequence to remove the His6-Tag, but the lab where the protein purification was performed does not use thrombin so the choice was made to switch to a TEVp-cleavable sequence.*
2. *N-terminal tryptophan: To facilitate spectroscopic determination of protein concentration, all BGL constructs include an N-terminal tryptophan in a "GWS" tag right before the structured region.*
3. *Single residue permutation: The N-terminal Asn of the original structural region was inadvertently moved to the C-terminus in the new construct. This residue is located on the loop that was cut to generate the sub3 variant, and the design is cyclic, so this permutation is not expected to impact the behavior of the protein.*

Perhaps some variants of the BGL interface, including BGL0, are sensitive to these differences. We recommend that future work with these constructs explore alternative expression constructs. We have added Supplemental Text 1, a discussion of the scaffold selection process, and Supplemental Text 2, a discussion of this potential limitation of the current BGL expression construct.

3. I appreciate that in order to summarise a large set of designs in a concise fashion, the authors present the results in the form of success rates, and explicitly name and discuss some successful designs. In doing so, however, it can be difficult to identify which designs were considered a success and according to which techniques were some designs deemed a failure. This is especially true as there are multiple figures and supplementary figures to follow for each design. This could be remedied if the authors could provide a summary table of all designs that summarises the results of each technique.

We think this is a great suggestion and have added supplemental tables S2, S6, S9 and S12 which we hope clarifies the criteria we used for success.

4. HetBGL constructs were co-expressed and copurified, whereas hetRTR monomers were individually expressed, purified and subsequently mixed. Could the authors explain the reason for the difference in protocol? Are the HetBGL monomers stable/soluble in isolation? If unknown, could the authors explain in the manuscript why they don't think testing this is relevant?

One goal of this work was to develop pseudosymmetric hetero-oligomers that could be purified as homogenous hetero-oligomers. While interesting and valuable, the requirement for these complexes to be able to be assembled after mixing fully purified subunits was beyond the scope of this work. Thus, we used co-purification in every case and co-expression when possible to make gene ordering and testing more simple. The only exception to this was the construction of the heterohexameric hetTRTs in order to take advantage of the huge combinatorics of reusing the same subunit in different assemblies. However, we did not end up doing as many combinations as initially anticipated in order to focus on the combinations of interfaces that were

found to work together the best in simpler assemblies (RTR05, RTR11, and RTR16). We have clarified the text.

While this work exclusively used co-purification, we have noticed that for a variant of hetBGL0-18-17 modified to form nanocages[3], when expressed separately and mixed prior to lysis (most/all chains having his-tags), it is necessary to wait 24 hours before SEC to allow the final cage to assemble due to, we believe, a slowly exchanging equilibrium between the final more favorable cage and an off-target cage formed out of a homotrimer of one of the hetBGL subunits. It is unclear how much this is due to the hetBGL subunit homotrimer itself or the added cooperativity of the off-target cage formation so it is difficult to draw conclusions.

5. In addition to changing the helix composition of A and B in A52B72-hetRTR* complexes, the constructs lack the GFP fusion of the A2B2 designs. If they haven't already, could the authors state why in the manuscript or SI?

Due to the mass difference between the sub5 and sub7 subunits, the GFP was unnecessary to distinguish between them by SDS-PAGE and mass spectrometry so it was left out. We added a sentence to the text to explain this.

6. One paragraph describes "A2B2 heterotetramers containing the RTR05 or RTR16 interfaces, which as homo-oligomers partially formed pentamers, did not contain any off-target pentameric species, suggesting that the added specificity constraints of heteromeric interactions may prevent off-target assemblies." However, I couldn't see a discussion of RTR16 forming successful hetero interfaces earlier in the paragraph.

We thank the reviewer for noticing this omission. A2B2-hetRTR05_16 indeed did not form any pentameric species according to native mass spec. We have updated the previous paragraph to include this fact, and have also taken this opportunity to clarify the criteria we use to consider a design as being correct, which brings it more in line with what the text claimed originally. This resulted in the count of the number of designs with the intended A2B2 species changing from 4 to 10. The text has been updated and a summary table included.

We do not consider the appearance of apparent partial species to be alarming, as these may be artifacts of nMS or real yet transient states which do not diminish the goal of detecting correctly assembled full complexes. The only truly concerning designs are A2B2-hetRTR11_18 and A2B2-hetRTR11noCys_18noCys (variants of the same interfaces) which both include "A3B1" species, which could correspond with assembled and stable off-target tetramers.

7. In one section starting "Each subunit of a heterotrimer from Set 1 has a different number of helices…" the authors describe converting HR-N back to HR-C. Could the authors state in the manuscript the rational for doing this, given the effort in designing HR-N constructs in the first place?

These designs were made in order to get more designs (in particular a variant of hetBGL03-15-18 which has a crystal structure) that resemble the heterotrimers from Set 2, which "display" the same outer C-terminal helix at each interface. This makes modular structural fusion to each terminus easier which is useful for structural engineering, such as in adding arms which are then used to dock the trimer into nanocages, as done in Lee et al[3]. This has been clarified in text.

8. I think it is reasonable to only continue biophysical characterisation on promising designs. For the authors' BGL homomers, I assume based on the number of proteins characterised that SEC and SAXS were conduct first, followed by nMS, and lastly SEC-MALS. However, proteins analysed by SEC-MALS are not all represented in the nMS set and vice versa (nMS BGL01, 02, 03, 05, 06, 07, 08, 09, 11, 12, 14, 15, 17; and SEC-MALS – BGL01, 02, 03, 05, 09, 12, 14, 15, 17, 19, 20). Could the authors explain the reason for this?

The reviewer assumed correctly that some samples were disqualified from further characterization. We have clarified this in text, and the new summary tables S2 and S6 make this easier to follow.

9. The outer shell high-resolution statistics for the X-ray crystal structures of BGL06 and hetBGL03-15-18 suggest that the crystals diffracted to a higher resolution (outer shell CC1/2 0.998 and 0.969, and I/sigI of 16.2 and 6, respectively). Could the authors explain their reasoning for trimming the data set to a lower resolution?

For two crystallographic structures (BGL06 and hetBGL03-15-18), while the diffraction patterns were quite strong and visible reflections extended well beyond 2.1 Å resolution, the spot profiles were streaky and poorly resolved beyond that point. Attempts to extend resolution beyond 2.1 Å led to poor behavior in refinement (specifically, deterioration of Rwork/Rfree values). Therefore, we conservatively maintained a 2.1 Å resolution cut-off for those refinements. (text updated to include this)

Reviewer #3 (Remarks to the Author):

Overall, the data supporting the claims are solid, however I should mention here that the data shows fairly large percentage of higher order aggregation species – this isn't a major problem per se, but it raises question about how specific this approach really is. It's quite hard to put a quantitative number as to how big the fraction of non-specific/designed association is, but I'd say it is not insignificant given the presented gel filtration/MS data. I won't request any additional experiments – it's a hard problem to address, but the authors should reflect it in the discussion.

We thank the reviewer for their insights. In many cases we were able to successfully purify the intended heteromeric species away from any non-specific assemblies including aggregates, showing that once formed, the designed assemblies are stable. For example, the non-reducing

samples of the RTR homotrimers in Fig S13b are reruns of the shaded fractions in panel a and show much cleaner peaks. In particular RTR14 had no aggregate peak in the rerun despite having a clear aggregate peak in the initial SEC. For the heteromeric designs, it is very difficult to control exact stoichiometric ratios of subunits during co-expression or simple mixture (as was carried out in this work) and mismatch in stoichiometry leading to an excess of one component is likely to lead to unintended behavior such as aggregation, especially since these proteins were not explicitly designed to behave as monomers in isolation. We reasoned that demonstrating stable purification of the intended complex will be sufficient for downstream applications where multiple rounds of purification are carried out anyway (e.g. vaccine epitope display). We have extended the discussion about applications and added caveats about the need to improve the biophysical behavior of these designs.

Reviewer #4 (Remarks to the Author):

1. Even though the manuscript shows interesting results, the way how is written and the results shown are confusing for some moments when reading. Since the authors have an extensive amount of constructs and experimental data, I strongly recommend adding a summary table that lists all the proteins discussed in the manuscript (dividing them into the different groups) and then indicates the different techniques i.e. SEC, SEC-MALS, SAXS, nMS, nsETM and what's the output of each of them (homooligomer, trimer, tetramer, multiple species, etc…) in comparison with the intended oligomeric state. I think this summary table will be extremely helpful for the readers to follow all the storylines and what are the best designs of the different groups.

We agree, and have added tables S2, S6, S9, and S12 to summarize results for BGLs, hetBGLs, RTRs, and hetRTRs, respectively.

2. I find it difficult to follow the overview shown in figure 1, maybe adding point descriptions can help.

We have expanded the overview to include the step of "Interface Diversification" and added guiding words to the figure legend. We hope this clears up any confusion.

3. I consider that all the evolutionary hypotheses/phrases related to the ancestry of the hetero-oligomers should be removed since this's not the topic of this work and also I don't see clear support for these discussions.

We believe that evoking the vocabulary of evolution is useful for conveying the ideas presented here, even if they don't actually relate to specific evolutionary processes involved in the scaffolds themselves. However, there is a clear ancestry that can be described for the final hetero-oligomers, with a lineage that can be traced back to the original design for the individual

interfaces, and the protocol itself was inspired by hypothetical evolutionary pathways for the creation of natural pseudosymmetric hetero-oligomers.

In the light of this reviewer's suggestion, we decided to remove the phrase "in a manner analogous to DNA recombination" and added qualifications to the use of the term "recombination" as it may have been overstepping the analogy, but we believe that the manuscript would be made harder for the broader scientific community to understand if the analogy was removed entirely. We also moved images in Fig 1 of natural proteins which have symmetric and pseudosymmetric homologs to the supplement to limit allusions to evolutionary processes.

4. SEC-MALS results should be revisited. The way they're presented is not completely adequate and not the expected plotting quality usually reported (especially for the type of conclusions the results have to support). First, plots in Suppl. Figure 4 should be replotted to indicate the molecular weight curve and see its dispersion along the elution peaks. Curves close to 20 min of retention time can be deleted since this information corresponds to the buffer signal. Also, the authors have to discuss the unusually high errors in their measurements (probably due to improper baseline selection or peak broadening/alignment). Authors should report the polydispersity for all the measurements and make clear if they report the mean MW or by peak. In BGL12, MW is indicated as 5.24 kDa, I assume it's a mistake (also, a second peak is observed and the MW of this peak is not reported). Would be better if the authors plot again the results and indicate on a right Y axis the MW for the elution peaks. To better compare with the SEC elution profiles, the X-axis has to be reported in elution volume (mL) and not in time (min).

We have updated the way that SEC-MALS results are plotted in the supplemental figure as suggested by the reviewer. Since the SEC-MALS is only supporting evidence and not used to disqualify any designs, we prefer not to belabor its analysis in the main text, but have added appropriate qualifications to the legend of Figure S7 (formerly Figure S4). The BGL12 MW error has been corrected. We have also updated the X-axis to indicate volume rather than time, but it must be noted that the SEC and SEC-MALS were performed on completely different instruments with different flow paths and thus the elution volumes cannot be directly compared. Additionally, some SEC-MALS results were collected at different times, between which there may have been changes to the flow path that prevent comparing volumes/times within the SEC-MALS dataset. We have indicated this in the figure legend.

5. I suggest Figure 2b be replaced with the equivalent results from SEC-MALS. That technique is more informative about the oligomerization state and MW in comparison to a normal SEC profile. The same applies to Figure 5b.

SEC-MALS (both absorbance and light scattering) overlays would be too difficult to read/compare to be useful, and overlaying of just the absorbance data is equivalent to SEC. The SEC-MALS data are better analyzed independently, and are available in Figure S7.

6. There are multiple SEC-MALS measurements that are missing in the Suppl. Figure 4 (number: 04, 06, 07, 08, 10, 11, 13, 16, 18). Is there any special reason for that? For many of them, it's important to report the data since other measurements are reported (SEC, MS, SAXS…) and would be useful to compare the behavior of the designs with different techniques. For example, it's intriguing why are not showing the results for BGL06 but a three-dimensional structure is reported. Authors have to show all the data for completeness.

The SEC-MALS data that was originally collected was on the sample that remained after running other experiments and there was not enough left of those to run SEC-MALS. We agree that it would be important to include SEC-MALS data for BGL06 to match the level of validation for the other designs with crystal structures so we expressed and measured this and the other designs lacking SEC-MALS yet with good SAXS and nMS data, and updated Figure S7.

7. In addition, why SEC-MALS measurements were not collected for hetBGLs? This would be relevant to discuss monodispersity and proper MW of the heterotrimers. Related to that, I don't agree with the authors when they stated "largely monodisperse and had the expected retention volume" since the SEC profile shown in Figure 4 presents multiple peaks (and asymmetry) which is a clear indication of polydispersity. If they want to show monodispersity, then SEC-MALS experiments should be collected.

We did not consider it important to collect SEC-MALS data for the hetBGLs given their potential for off-target oligomers (nearest being within ~10% of the mass of the on-target) to appear like the on-target within the resolution of SEC-MALS (at worst 20% of the on-target). For these, nMS was more useful because it has the resolving power to unambiguously distinguish the alternative species. The reviewer's mention of the potential polydispersity shown in Figure 4b is apt and has been included in the text.

8. I found unclear how was calculated the stoichiometry discussed on Suppl. Figure 6. Especially when extra bands are present like in BGL03-06-05, BGL08-19-09, and others. Authors have to clarify this calculation and their classification between uneven stoichiometries and "approximately" equal stoichiometries. This is especially important because they stated "Protein size affects band staining, so it is difficult to judge stoichiometry from the band darkness/size, but the correct trend in stain density is observed for equal stoichiometry", then discussing stoichiometries is quite tricky with this approach.

This was a qualitative process using no calculations — we have updated the legend of figure 4 to state this more simply. We have also amended the text to make it clear that these observations were not used to filter out any of the designs and SEC and nMS was performed for all. The extra bands are likely to be contaminating endogenous E. coli proteins carried over from IMAC.

9. In general, would be important that the authors discuss their design failures and the discrepancies observed among all the methods. For example, heterotrimers show a clear sign of a mixture of different oligomeric states; for me, it's not convincing that the elution profiles

shown in Suppl. Figure 7 are monodisperse as the state in the manuscript, most of them show multiple and asymmetric peaks. Also, the difference in the elution for 03-05-06 and 06-05 is visible, in addition to the extra observed peaks in the elution profiles. SEC-MALS can give further information about their behavior in solution.

See response to comment 7 from this reviewer and comment 2 from reviewer 5. We have included a note about the interesting differences between heterotrimers sharing the same interfaces but with different behaviors and have added descriptions of experimental discrepancies.

10. Throughout the manuscript, when authors compare models and structures, they should provide the number of Ca that were compared in the RMSD calculation (text and figure legends).

Updated.

11. I find a bit loose this argument: “the three distinct subunits have an average pairwise sequence identity of 62.3% but high structural similarity (aligned CA-RMSD of 1.5 Å), showing that this method can produce highly (pseudo)symmetric hetero-oligomers.” Could the authors explain what they meant by this?

As there is no accepted metric for pseudosymmetry, we chose to highlight the high structural similarity among subunits despite a relatively low sequence identity. Instead, a better metric might be to compare the total aligned sequence identity from a structural alignment by TMalign between the heterotrimer and the parental BGL0, and to report the RMSD and TMscore between aligned residues. TMalign was performed on all chains concatenated into a single chain and with the “circular permutation” flag enabled to account for the circular permutations due to inclusion of an HR-N interface. We found a sequence identity among aligned residues to still be 62.3% with an RMSD of 1.7 (over 501 residues) and a TMscore of 0.94. We believe this whole-complex comparison is more relevant than comparing the individual subunits.

12. SAXS results shown in Figures 2 and 5 should also show the comparison of experimental results with the computed profiles. In addition, many of the SAXS plots show a clear discrepancy between experimental data and model prediction. I think would be informative discussing a bit about these issues since can be helpful to the readers related to data interpretation.

See response to comment 2 from reviewer 1. Overall, many of the SAXS plots for the BGL and RTR designs (especially the ones with good results from other techniques) show an excellent match with model predictions as shown in the new Supplemental SAXS analysis tables and now summarized in Tables S2 and S9. It is typical to plot the raw scattering signal on a logarithmic scale which magnifies differences at smaller values which can be misleading. To help the reader visualize the quality of the comparison, we include residual plots on linear scales under each scattering plot.

13. BGL13 SAXS measurements are missing in the Suppl. Figure 5.

SAXS data was not collected on this design as it was unstable at high concentration. The text has been amended to make this point clear.

14. Regarding the homotetramers, the authors stated: “For 10/24 redesigns, SEC elution profiles suggested a single homogenous species with the target subunit composition (Fig. 5b, Fig. S13)”. It’s unclear what are those designs and again, to say that they elute like tetramers, SEC-MALS would be helpful in addition to their MS data, which also would help to clarify the degree of polydispersity.

We have included summary table S9 which clarifies this. We agree that standard SEC is insufficient for assigning an oligomeric state, hence our use of the term “suggested”. We have made this more clear in the updated text.

Minor issues:

15. Repot cut-off criteria of the crystallographic data. For a couple of structures is not clear why that resolution cut-off was used.

Please see our response to comment #9 of reviewer #2.

16. Why is there a discrepancy between the resolution reported in data collection and refinement for BGL15 structure?

While we agree that the statistics above indicate that the data set may extend to higher resolution than was chosen for a final cut-off, the behavior of the model during refinement (particularly the difference between Rwork and Rfree) indicated that beyond our chosen cut-off, an inappropriate amount of ‘modeling to noise’ was being included. Therefore, we chose a more conservative strategy of lower resolution cut-off for those refinements. We are very confident in the structural details and corresponding interpretations for both of those experimentally determined crystal structures.

17. In addition, Matthews coefficient Vm also should be included in the table; would be important to show the number of molecules in the ASU since this is relevant for heterodimer formation.

This has been included in table S4.

18. In the methods, the authors didn’t say if they pooled all the elution fractions from SEC for further experiments. This might be important if the proteins were showing multiple oligomeric states during the protein preparation/purification.

We have updated the Supplemental Methods and indicated which fractions were taken forward for downstream analysis as shaded regions on the respective supplemental figures showing SEC data and on the individual hetRTR SEC panels in Figure 5. .

19. With the extensive number of constructs and possible difference of isoelectric points, did the authors consider that some of the failed designs were due to improper pH during purification, i.e. that buffer was not adequate and protein aggregation was due to the pH being too close to the pI?

Individual (predicted) isoelectric points of all protein subunits were considered independently and adjusted to be different from the pH of our buffers (pH 8.0) (See Supplemental Information: Computational Methods)

Reviewer #5 (Remarks to the Author):

Overall, it is a sound study but require further clarification in writing, particularly related to the interpretation of the experimental data and the integration with the discussion of protein design.

We thank the reviewer for pointing out the need for clarification surrounding the interpretation of experimental data and the discussion section. We have revisited our explanations of experimental details to the main text and added clarifying details where possible and simplified sentence structure. We have also added details about how this work relates to recent advances in protein design.

Major comments:

1. The authors selected different assays at different stages. Higher throughput methods generally had more complete datasets. The best ones were sent to crystallography at the end for detailed interface analysis. The data from different methods did not seem to all overlap perfectly. What is the complementarity of these assays? It is recommended to discuss how the data from different methods were used to drive the decisions (i.e., what is considered good hit or not). Was there any (semi-)quantitative analysis involved? For example, in FigS2 and S3, BGL07 seems to fall apart in native MS but not in SEC.

See response to reviewer 2 comment 8.

2. Related to the comment above, nMS appeared to miss data for some samples while SEC data appeared to be more complete (e.g., BGL04, 10, 13,16. BGL20 was only shown in Table S7). Are some of them simply considered bad and discarded? If so, do these "bad" results correlate with other assays and indicate aggregation, etc? Are the seemingly negative data informative in some way for the design?

Indeed, data was not collected on all designs usually for reasons of exceptionally poor behavior. Understanding why some designs failed and others succeeded would be interesting and may

lead to new understandings of protein design, but our goal was to demonstrate the creation of pseudosymmetric hetero-oligomers, and we obtained enough successful designs that it was not necessary to fully analyze and rescue the failures. We did learn/reinforce some lessons in the analysis of the designs that worked, like the need to avoid installing HBNets too close to potentially unstructured regions of the protein (See description of the hetBGL03-15-18 crystal structure).

Other minor comments:

3. The nMS data processing workflow seems to work well for high quality spectra, but not always for noisy spectra. For example, hetBGL0-18-17, hetBGL0-18-19, hetBGL0-19-17, hetBGL0-19-18 seem to be not picking up the peaks around 7000 m/z. BGL0 seems to have extra peaks next to the base peak that may be artifacts. Maybe some spectral smoothing is needed to minimize artifacts in the deconvolution. While these details do not change the main conclusions of the paper, the spectral details may speak to the properties of the proteins.

The reviewer thoughtfully suggests removing some of the artifacts in the deconvolved mass spectra using Gaussian smoothing. However, we preferred using the same deconvolution parameters across all mass spectra for consistency, which don't use Gaussian smoothing. As the reviewer points out, it is interesting that some samples desalt better than others; however, we are currently unable to make a definitive conclusion on why this is the case, and it may be something we follow up on in a future manuscript.

4. I recommend better clarifying the potential biological applications related to polyhedral assemblies, nanocages for readers to better understand the motivation. This may not be obvious to readers outside the immediate field of expertise. Current design assumes protein core regions are identical and only interfaces were changed. This may not apply to enzymes that have active site in the core and cannot be easily re-engineered. For structural proteins the downstream applications are easier to relate to. Nonetheless, it is better to comment on how this strategy can be broadly applied to design complexes that have sensitive structures in the core of the protein subunits.

We thank the reviewer for their insight and have added a note to the discussion section pointing out the success of modern diffusion-based backbone generation/diversification methods which are capable of strictly preserving important protein fragments from parental structures. We believe this, along with careful selection of the point/plane used for splicing during structural recombination, would make this technique applicable to many relevant systems.

We have also expanded the discussion surrounding the biological applications of nanocages.

5. nMS data for heterocomplex Fig S8 and Fig 4 may be better displayed to highlight the homogeneity of the trimers, i.e., absence of other combinations of trimers. This is a unique contribution for nMS compared to other assays used. It is not obvious what mass differences

are expected for ABC vs AAB, etc, and if they are readily resolved and/or quantifiable in nMS spectra with the full range spectra shown in the figures.

We thank the reviewer for their suggestion. The alternative trimers closest in mass to the ABC (93.6 kDa) are ABB (83.4 kDa) and ACC (103.8 kDa). We have added an insert to Figure 4d to indicate the expected signal positions for these nearest off-target species.

6. Some additional peaks in nMS data were labeled with mass shifts not explained (for example hetBGL03-15-18_reloop, hetBGL08-09-19_reloop, RTR04_C4, RTR0_C2, RTR0_C2, etc). Were they from miss-assembled complexes or just from adducts/truncations/PTMs? Were those extra species being considered as penalties in the design optimization?

We thank the reviewer for their close inspection of the mass assignments and shifts we observed from the expected mass. These mass shifts are most likely due to additional adducts that remain after the online buffer exchange process. However, they may also be PTMs or truncations. For our typical native MS experiments, we used 6k resolution, which works well for these high-mass complexes but makes it difficult to distinguish between additional adducts or PTMs. A follow-up manuscript we would be interested in is analyzing these samples with different gas-phase dissociation methods, which would eject adducts but retain covalent modifications.

References

1. Chen, Z. *et al.* Programmable design of orthogonal protein heterodimers. *Nature* **565**, 106–111 (2019).
2. Hallinan, J. P. *et al.* Design of functionalised circular tandem repeat proteins with longer repeat topologies and enhanced subunit contact surfaces. *Commun. Biol.* **4**, 1240 (2021).
3. Sangmin Lee *et al.* Design of four component T=4 tetrahedral, octahedral, and icosahedral protein nanocages through programmed symmetry breaking. *bioRxiv* 2023.06.16.545341 (2023) doi:10.1101/2023.06.16.545341.

REVIEWER COMMENTS

Reviewer #1 (Remarks to the Author):

The authors have added in additional data that I have requested. The additional SAXS data in Tables S3, S7, and S10 illustrate the complexity and difficulty of quality assessment of the design molecules. The experimental result summary is meant to address this in an unbiased fashion, but the decision points are based on single values, while the curves that have more information content are not included.

The experimental result summary is meant to provide high-level overview information on each design and show how they were selected for further characterization and/or downstream use. The SAXS profiles and subsequent transformations are available in the SAXS tables with additional analysis, but these analyses were not used to determine the success or failure of a design.

In looking at the SAXS data, paper's threshold of similarity is a concern. If this was a natural protein, then the differences observed in every curve was substantially different than the model, particular when looking at the p(r) where even the peak is shifted suggesting global changes. As this is a de novo designed protein and the crystallography and EM are showing similarity, then the question is whether the same standard be applied? Is it sufficient to be similar, as in an oversimplified analogy, a sphere is to an egg?

In this work, we used this high-throughput SAXS (HT-SAXS) as a fast and low-resolution structural screening tool in the testing pipeline to help identify designs worth spending more time characterizing. Our objective was to only roughly measure the size and shape of the particles, and with this goal in mind we believe that our level of analysis and relatively generous filtering criteria is appropriate. Additionally, while there were some deviations between the theoretical and experimental scattering curves, the experimental scattering curves themselves of different successful designs (e.g. Fig. 2c) overlay remarkably well, which indicates that their shapes in solution are very similar even if they don't exactly match the computational model. This supports the hypothesis that the redesigns are not too different from each other to prevent their structural recombination.

Nonetheless, the SAXS data is the ground truth, albeit obscure. Typically, higher resolution analysis is considered decisive in its validation of the model, but can introduce biases from solution/grid conditions and from filtering by crystallization or by single molecule analysis. In contrast, the SAXS solution experiment is a sum of all molecules in solution, and may be an important predictor on what stoichiometric or conformational state for the conditions in which the protein will be used. And here, the differences revealed in the SAXS data is informative and insightful. SAXS data is not simply validating the protein design but providing additional features that is intrinsic to the design.

The reviewer is correct that an impressive amount of information can be extracted from SAXS data in conjunction with other experimental validation. Our use of HT-SAXS, however, was only as a screening tool. As the reviewer pointed out previously, to get high quality and interpretable SAXS data, SEC-SAXS data would need to be collected and analyzed. Instead we used orthogonal techniques to validate the designs (SEC-MALS, nsEM, nMS, and X-ray crystallography).

The authors need to include analysis of the real space SAXS curves when considering whether the SAXS curves truly validate their model.

For example, in the first two SAXS data tables, every protein is larger in solution than the predicted structure or the crystal structure, implying that although the crystallography has indicated that the topology is correct, the protein is expanding in solution and may be more flexible. This flexibility, if true, may be a useful feature. Flexibility can be indicated from the SAXS data using the Porod exponent or the dimensionless Kratky comparison to well-folded proteins. Flexibility may also be critical in permitting non-ideal geometry in the larger order assemblies.

We thank the reviewer for sharing these intriguing ideas and we agree that the P(r) transformations of the raw data indicate differences from the design model for some samples, particularly of the design BGL series, and that the particles may contain flexible elements. Several contributing factors may lead to the deviations observed between the SAXS data and the design model. These can include experimental effects such as poor buffer subtraction, the presence of buffer components or other conditions that may affect structure (e.g. glycerol, pH), and X-ray radiation damage. Additionally, some design-intrinsic effects such as overpacking leading to swollen particles or fraying of terminal helices (such as in the BGL03 interface as seen in the crystal structure of hetBGL03-15-18) could be behind the right-shifted P(r) plots and flexibility indicated by Kratky plots. As such, we have updated the manuscript to acknowledge this.

In the third SAXS table, most solution data were not consistent with their respective atomic models. For many, this difference is likely due to problems in stoichiometry at the time that the data was collected. The authors state that they do not want to overinterpret their data using mixed populations, but if that is indeed the case in their actual sample, then it seems that including subunit models is the only way not to overinterpret their data. It is also a way to eliminate subpopulations that do not exist. The SEC reflects the propensity of the design protein to form subpopulations in equilibrium, acting to indirectly support the analysis and reduce the charge of overinterpretation. Interpretation of the SAXS data is needed for every one of these curves. For example something quite strange is happening in RTR24_DHR62.

It is true that we may not be using the SAXS data to its fullest potential by limiting ourselves to states we expected to be present a priori. In the case of the third SAXS table, the particles in these samples were hypothesized to be ring-shaped oligomers when the disulfide bonds at the interface are formed and monomers when they are broken in reducing conditions. Therefore, we

fit both the full tetrameric design model, which represents a closed ring of the size we would expect, and an extracted monomer from the design model. By comparing the quality of fit (via X2 values) between these models, we can say which one is better able to explain the data. This lets us tell, under non-reducing conditions, if the particle is likely to be a ring-shaped oligomer or not, which was the primary goal we had of collecting the SAXS data. This level of analysis was sufficient for our purposes, and raw data files from the HT-SAXS experiments and the design models are available on zendono for others to explore in more detail for those seeking more interpretation.

We would like to thank the reviewer for highlighting the strange RTR24_DHR62 SAXS data. This design was not one of the RTR interface redesigns discussed in more detail in the main text. Instead, it was part of the RTR stud extension to diversify RTR shape with results shown in Fig. S16. We regret that the way we included this design and the others from the extension work in the SAXS table S10 may have been confusing. We have edited the RTR SAXS table (Table S10) to better distinguish between these and added Supplemental Text 3 to more thoroughly discuss the results of the Extension campaign, including the SAXS data. Furthermore, we agree that something quite strange did happen with RTR24_DHR62. Based on the large residual between the experimental SAXS data and the fitted profile and the corresponding relatively high X2 value, it seemed that the shape of this particle was significantly different from expected. According to nsEM, a ring with an extension can be observed, but most particles on the grid have a different shape. This should have disqualified it from downstream use. Unfortunately, these fusions were initially tested and chosen for use in hexameric hetRTR designs based only on their SEC traces; the RTR24_DHR62 traces suggested assembly so the DHR62 extension was used in downstream designs. None of the designs using the DHR62 extension assembled correctly, so it is possible that something about this fusion prevented assembly. This explanation is included in Supplemental Text 3, with the recommendation to avoid the DHR62 extension in the future.

Additional comments

1. Connecting the molecules being discussed in the main text with what is in the supplemental sections needs to be clarified. It would also be helpful if the authors explicitly call out which molecule that they are referring to, in every place in the main text. For example, the authors state “Our success rate of 24 experimentally-verified hetero-oligomers”. It is challenging to trace, even when looking at Tables S2, S6, and S9, which are these 24. The authors tend to count (e.g. three out of 12) than to explicitly list which ones. The supplemental tables summarizing the true/false are critical but even there it is still difficult. Without knowing explicitly which ones that they are referring to, I have guessed from their tables and illustrative examples and it prevents reviewing this work with high confidence.

In this example, 24 is the sum of all experimentally verified hetero-oligomers across both the hetBGLs and the hetRTRs. We have updated the text to clarify this point. In all cases, the summary tables do list exactly which ones passed screening in the final “Overall pass” column, as well as per-experiment “pass” columns for the more granular breakdowns. We agree that this

introduces an additional step for the highly invested reader to take to understand exactly which designs are being referred to, but this is a common practice and we believe that listing the name of every design in every instance in the main text would make it unnecessarily crowded and difficult for the typical reader to understand. We have updated the text to list out the names of designs when counting and there are 5 or fewer names. For larger numbers, the reader will have to refer to the summary tables.

2. Were the C2 values in the FOXS analysis close to 4, which indicates that the water layer was being stretched erroneously to cover the discrepancy? C2 can lead to lower chi values.

The c1 and c2 parameters were fixed to 1.05 and 2.0, respectively, for all FoXS fitting to avoid this issue. This detail was included in the initial submission but accidentally removed during the first revision. We apologize for the error and have updated the methods section.

3. The authors use a <30% variability in Rg as a cutoff. This seems overly generous. Typically, sample-to-sample differences are <10%, but 30% allows for different shapes to pass through. Comparison of the p(r) curves show the danger of this. A case in point is BGL14, which is seemingly a good scenario with a change in Rg from 23 to 26. Examination of the p(r) curve indicates that the protein in solution is significantly more elongated than in the prediction.

Given the high relative difference observed for the Rg of the parental model (BGL0: ~36%), we reasoned that Rg may not be the most useful discriminator for predicting design quality, thus we believe the relatively generous cutoff to be appropriate.

4. Kratky plot should be shown only to q ~0.3. The reason being is that the higher resolution data is noisier and obscures what happens after the main peak. The curve for a well-folded protein will be more bell-shaped, for a protein with a significant disordered tail, the curve will tail up at higher q; a protein that has global conformational flexibility will go up and decrease at a lower slope than the well-folded protein; a curve for a completely disordered protein will either peak and stay at the peak or even continue to rise. The interpretation of the Kratkys are not given in the table, highlighting the difficulty in extracting this information.

We thank the reviewer for the explanation. We have truncated to q ~0.3 and added a comment field next to the Kratky plots to supply this interpretation.

5. The p(r) curves appeared to be normalized based on the maximum value and highlights changes in the peak position, but can minimize shape differences. Normalizing based on the area under the curve can bring out features indicative of more elongated structures. The authors should consider both types of curves in their analyses.

We thank the reviewer for this explanation. This level of analysis is outside of the scope of this work and would be better performed on high-quality SAXS data, such as from SEC-SAXS.

6. What were the Porod exponent values?

The Porod exponents were included in the SAXS tables (S3, S7, and S10), as requested.

7. There is no discussion of reduced versus non-reduced in the SAXS data, except for the engineering of the cysteines. It should be included in the main text.

This detail was included in the initial submission but accidentally removed during the first revision. We apologize for the error and have updated the text.

Reviewer #2 (Remarks to the Author):

I am satisfied with the changes made to the manuscript and associated documents by the authors. I appreciate the effort made in generating tables to follow design results, and I look forward to seeing future applications of the designs and design protocol.

Best wishes,
Guto Rhys

We thank the reviewer for their helpful comments in the previous round, and suggest the reviewer keep an eye out for the peer-reviewed publication of the work by Lee et al and posting of work by Rankovic et al to a preprint server.

Reviewer #4 (Remarks to the Author):

The authors made a good effort to reply to my and all the other reviewer's comments. I recommend the paper for acceptance in this current version.

We thank the reviewer again for their helpful comments in the previous round.

Reviewer #5 (Remarks to the Author):

The authors have addressed the major questions for reviewer #5.
For reviewer 5 - comment 6: I recommend at least adding some notes in the supporting documents to explain the potential origin of the extra peaks. Chemical modifications, terminal truncations, etc may affect protein function so it is better to inform readers about these possibilities.

We thank the reviewer for their suggestion and have updated the methods section to include commentary on the identities of the unassigned peaks.
