# Supplementary material for: Design of pseudosymmetric protein hetero-oligomers
Source: Nat Commun. 2024 Dec 18;15:10684. doi: 10.1038/s41467-024-54913-8 (PMC11655659; doi:10.1038/s41467-024-54913-8)
Supplement: Supplementary file 4 [file 41467_2024_54913_MOESM1_ESM.pdf]

Supplementary Figures

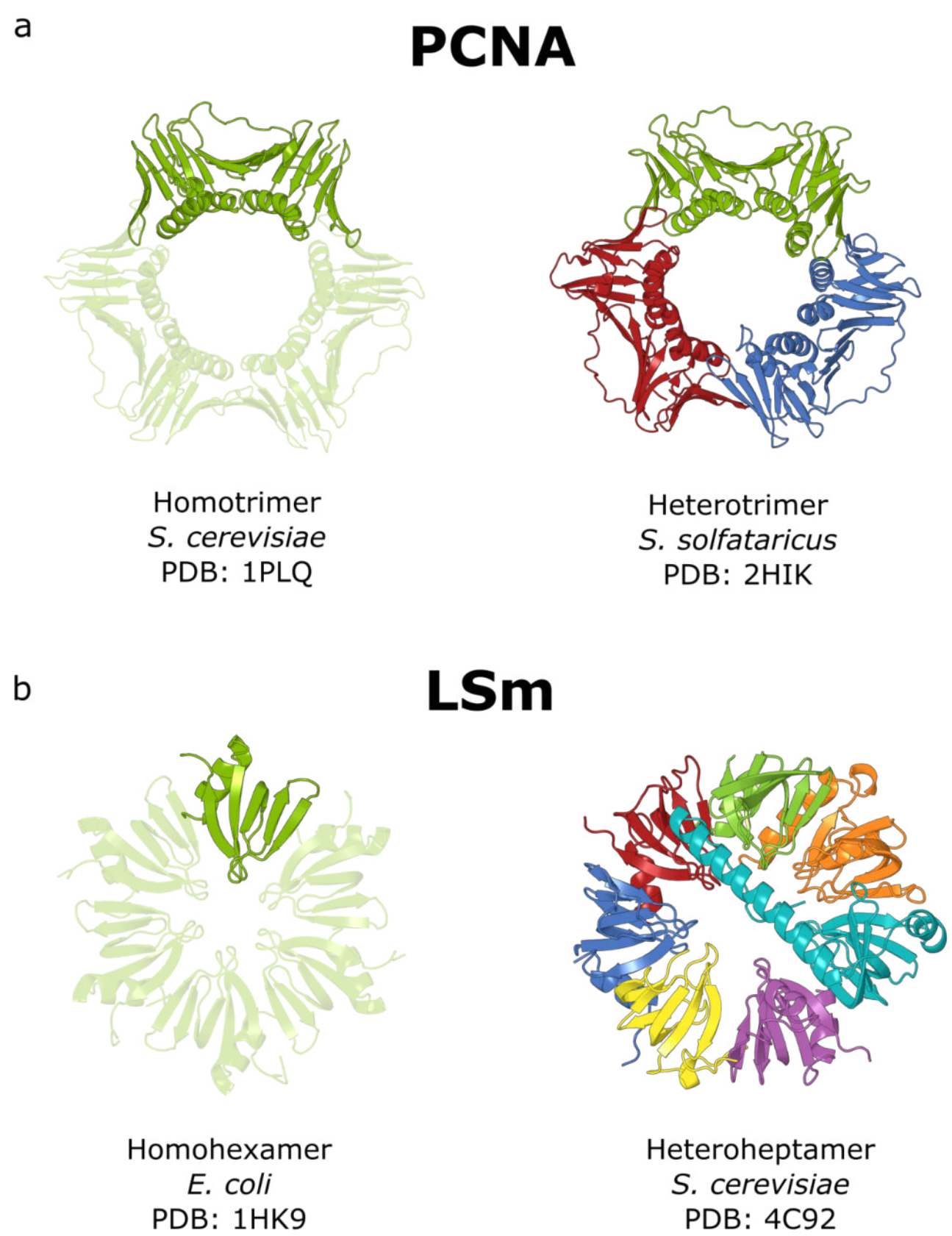

Supplementary Figure 1: Structures of native pseudosymmetric hetero-oligomers
Two examples of natural pseudosymmetric hetero-oligomers (each pair, right) which likely arose from symmetric ancestors, resembling the paired symmetric paralogs (each pair, left), through gene duplication and diversification. (a) LSm complexes from *Escherichia coli* (Hfq) and *Saccharomyces cerevisiae*. (b) PCNA from *S. cerevisiae* and *Sulfolbus solfataricus*.

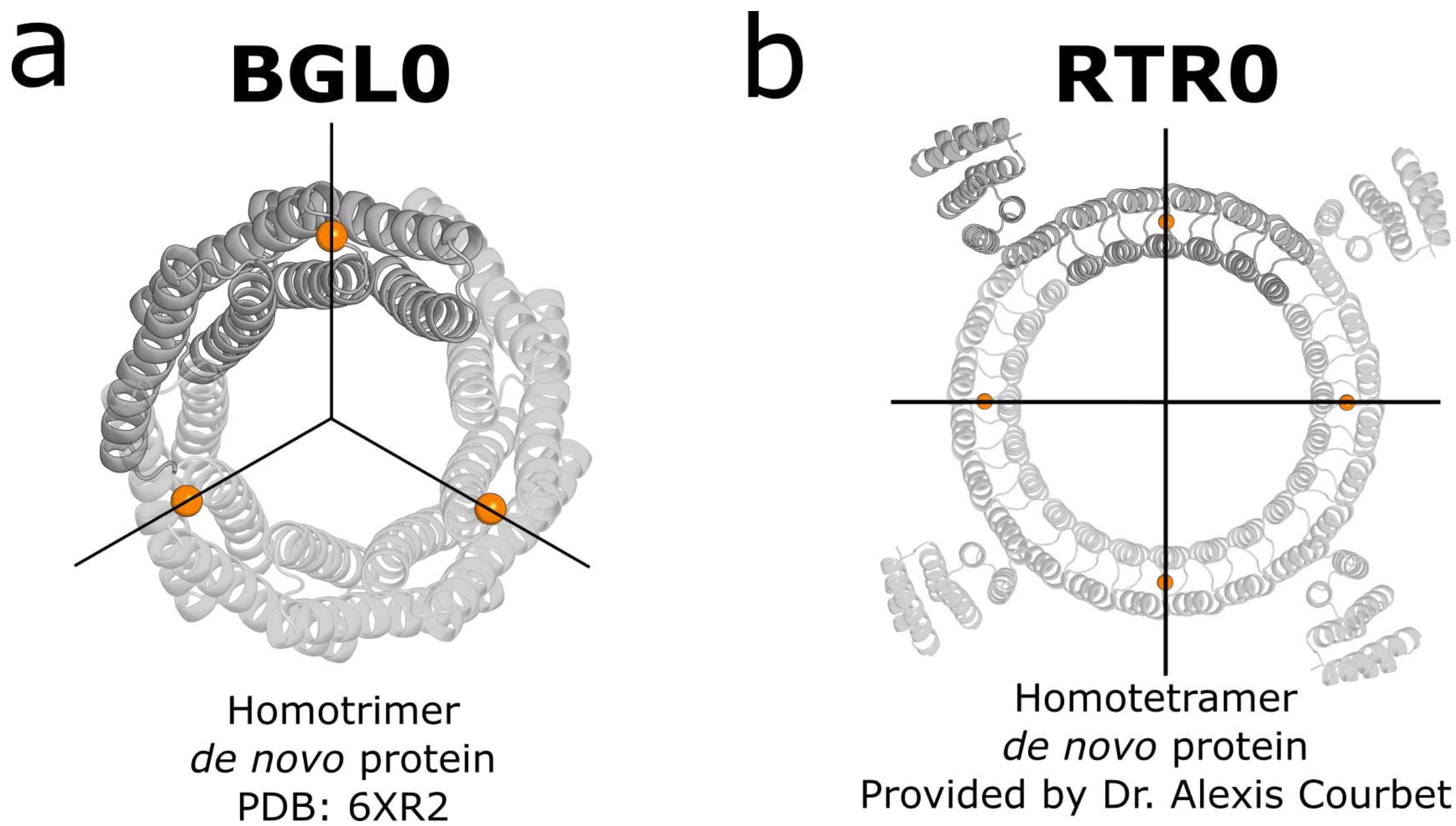

Supplementary Figure 2: Structures of parental homo-oligomers used in this work

The two test scaffolds used in this work, (a) the homotrimeric tcTRP9_sub3 (PDB: 6XR2)[1] from referred to here as BGL0; and (b) the homotetrameric C4_1[2] referred to here as RTR0. Each is intersected by black crosshairs which pass through the orange sphere on each chain, indicating the location of the residue (Arg82 and Leu98 for BGL0 and RTR0, respectively) within the junction region where splicing will occur.

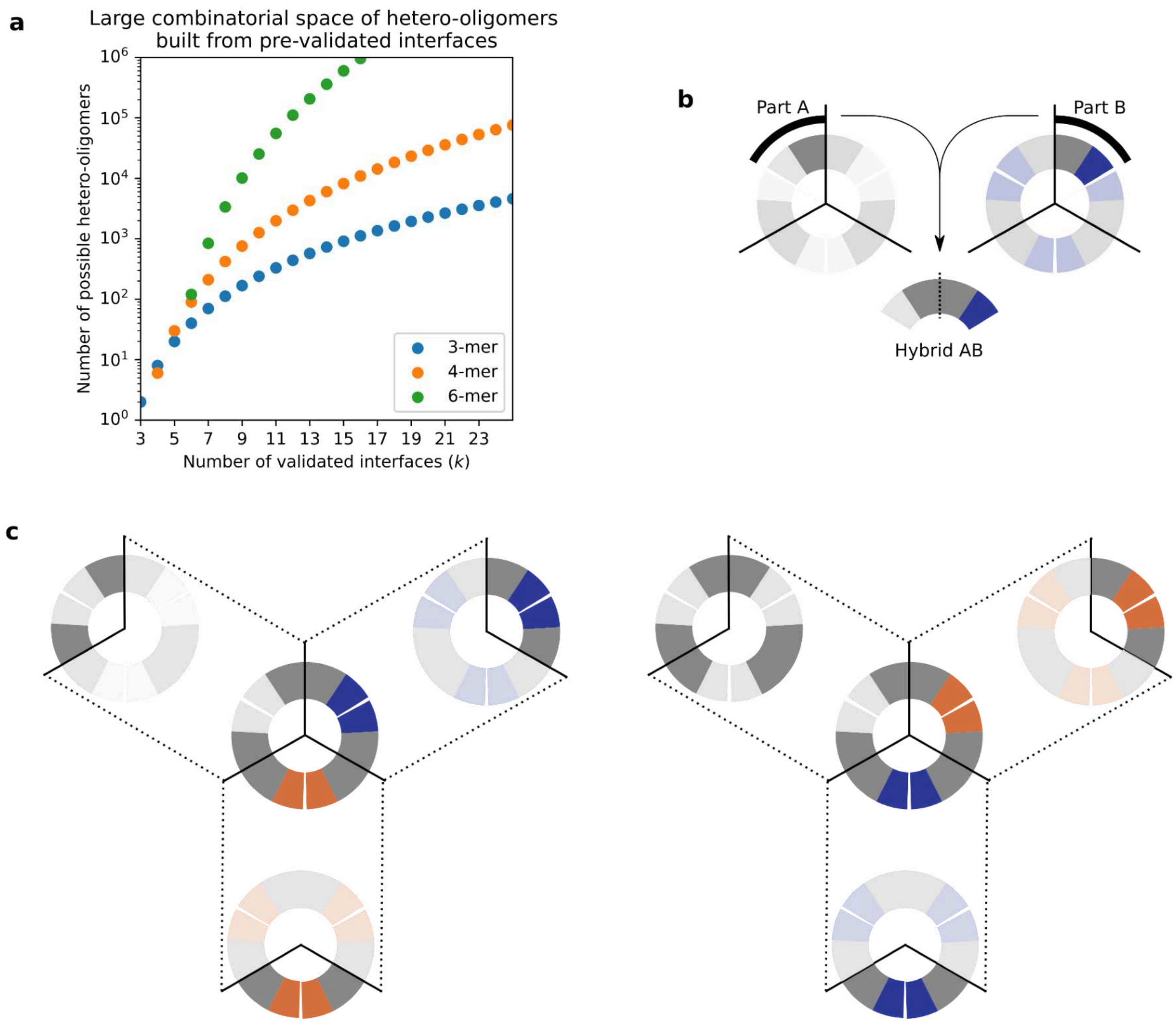

Supplementary Figure 3: Combinatorics of homo-oligomer recombination

(**a**) Plot of the equation describing the number of possible hetero-oligomers that can be made, (k! * (n-1)!) / (n! * (k-n)!), for a few n, where n is the number of different chains in the final heter-n-mer (e.g. heterotrimer = heter-3-mer) and k is the number of interfaces or homo-n-mers which have been validated to assemble similarly. Shown are plots of n=3, 4, or 6, some of the most commonly useful hetero-oligomers, to illustrate the large number of options for combining a few interfaces. (b) The method of combining parts of two homotrimers to form a hybrid protomer of a heterotrimer. The chains of the homotrimers are computationally split into two parts at the same point on each chain as shown by the 3-pointed crosshair overlays, creating part A and part B. Because the molecular context at the cut points are identical, parts A and B can be spliced together at the cut point to form Hybrid AB without altering the structure of the protomer. (c) There are two ways to combine the same three homotrimers to form

pseudosymmetric heterotrimers because order matters for the way the interfaces are arranged around the circle. There are many more ways to combine higher n hetero-n-mers.

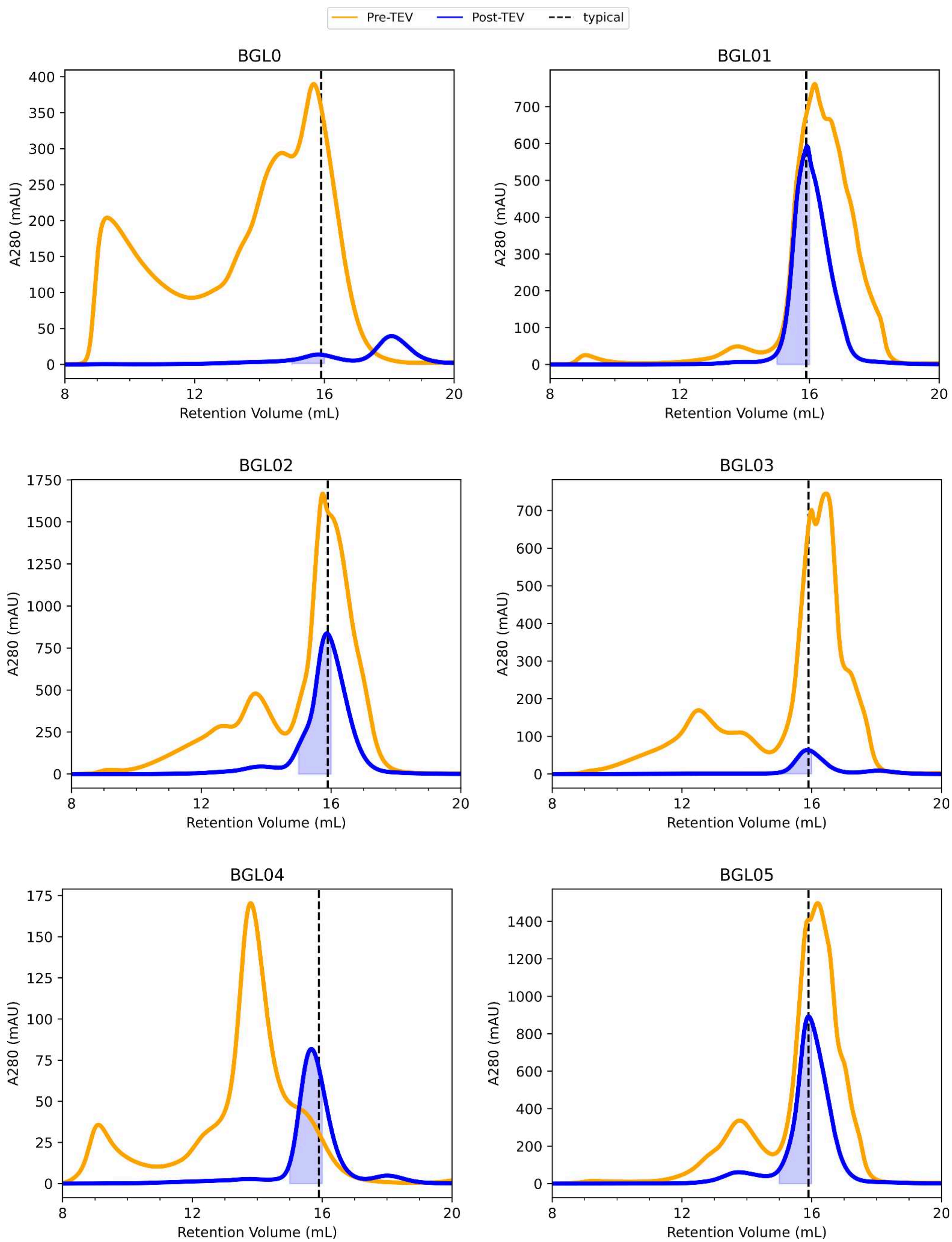

Pre-TEV
Post-TEV
typical
BGL0
BGL01
BGL02
BGL03
BGL04
BGL05
A280 (mAU)
Retention Volume (mL)

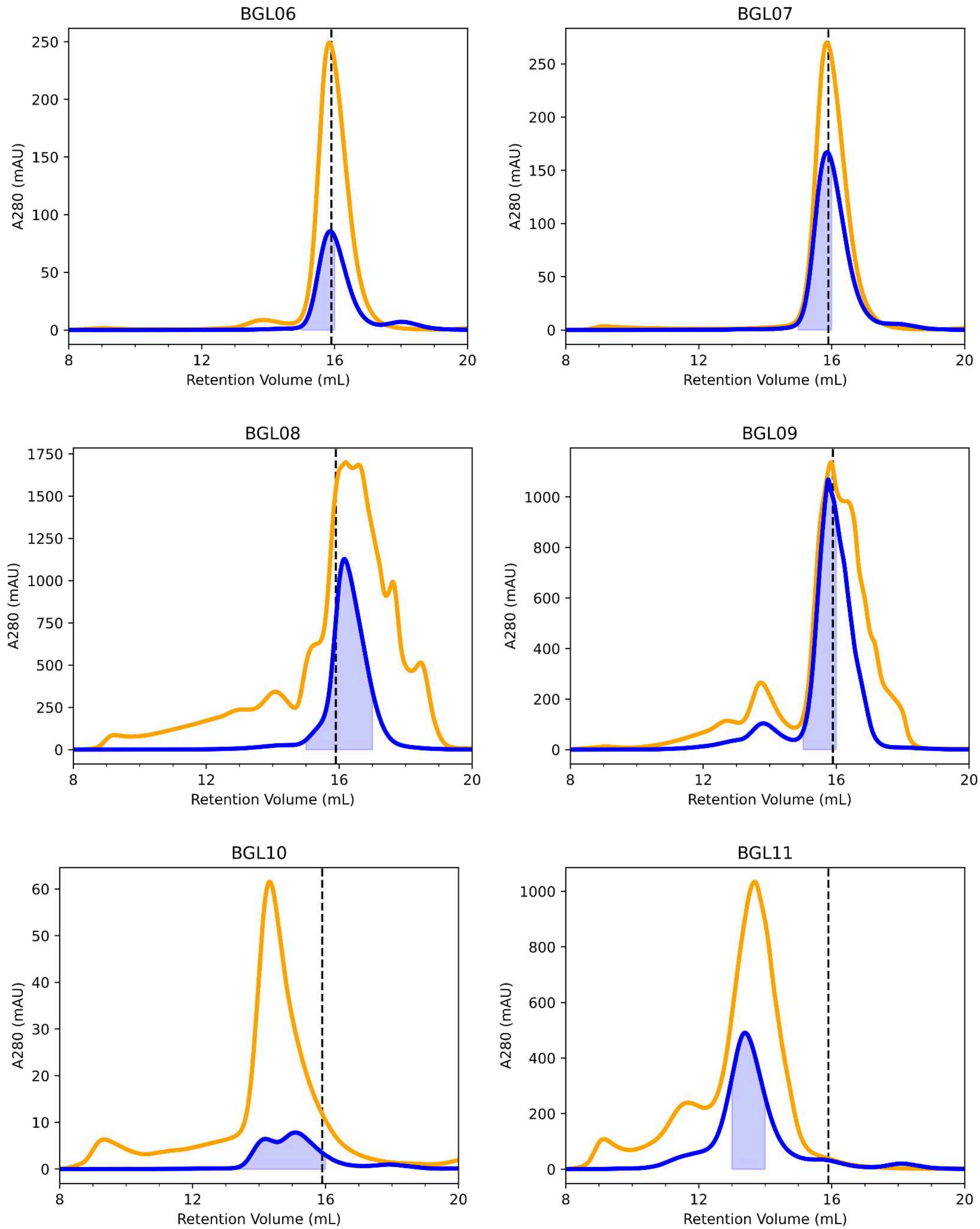
BGL06
A280 (mAU)
Retention Volume (mL)
BGL07
A280 (mAU)
Retention Volume (mL)
BGL08
A280 (mAU)
Retention Volume (mL)
BGL09
A280 (mAU)
Retention Volume (mL)
BGL10
A280 (mAU)
Retention Volume (mL)
BGL11
A280 (mAU)
Retention Volume (mL)

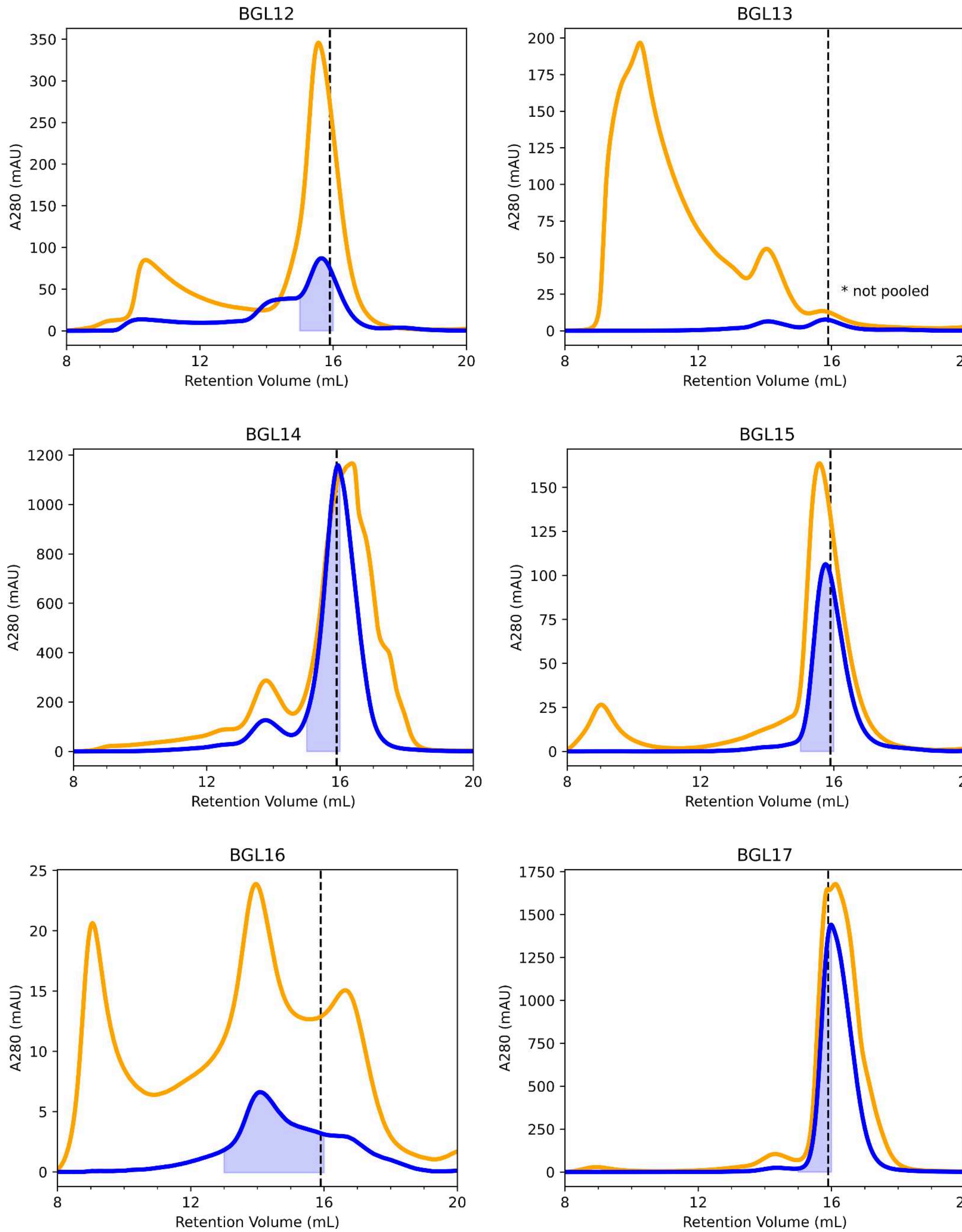

BGL12
BGL13
* not pooled
BGL14
BGL15
BGL16
BGL17
A280 (mAU)
Retention Volume (mL)
8
12
16
20

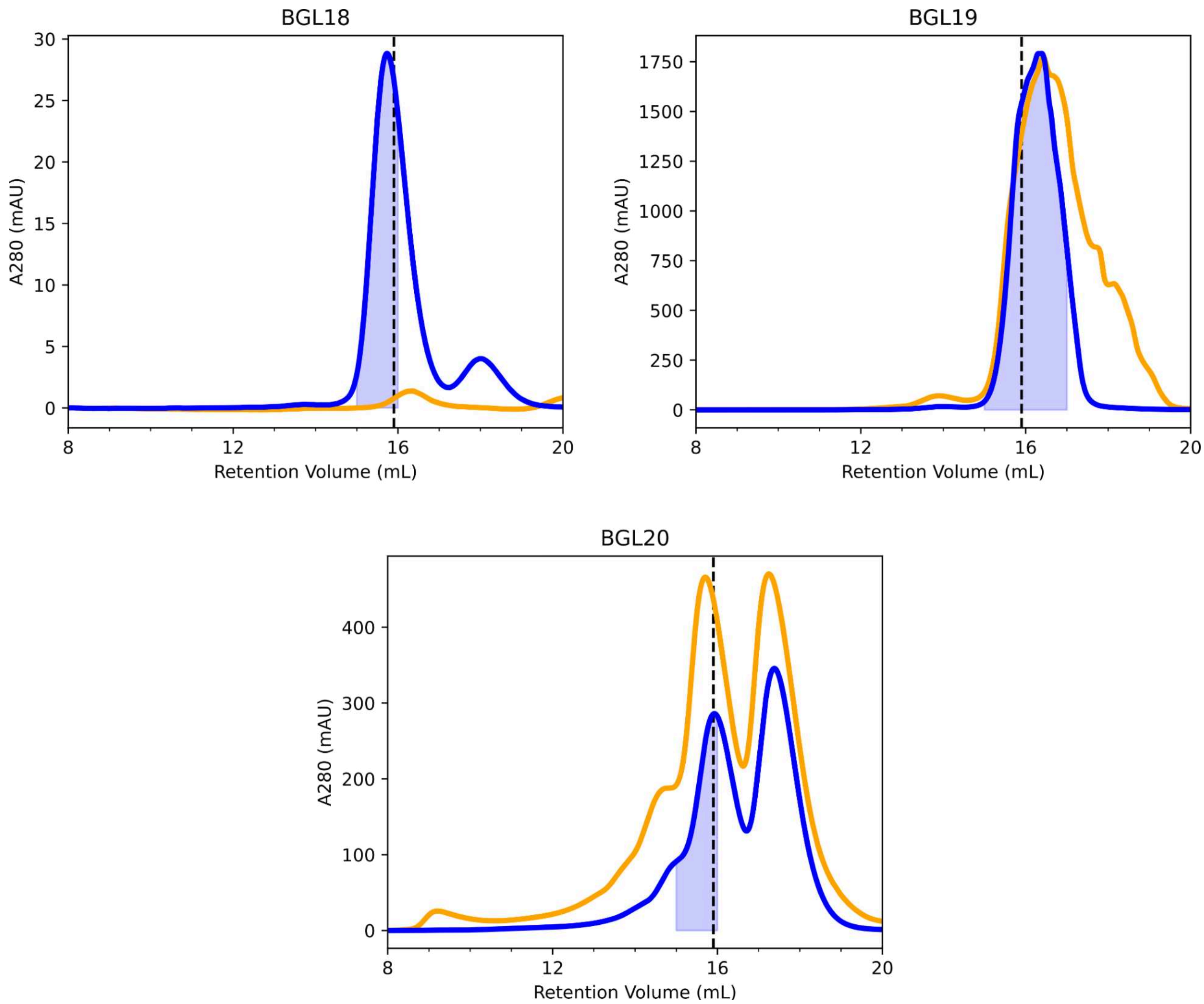

Supplementary Figure 4: SEC of BGL homotrimers

Plots of all designs before (orange) and after (blue) TEVp cleavage, run on S200 Increase 10/300 GL. Blue shading indicates regions used for downstream analysis. Note that some samples were split and run separately so absolute abundance is not comparable in all cases (e.g. BGL18). The right-shift pre- to post-TEV in BGL04 can be explained by removal of interactions via mediated His6-tags, which are a relatively common occurrence in cyclic homo-oligomers. BGL0 was not efficiently cleaved, causing low yield of possibly mis-assembled protein in post-TEV SEC (Supplementary Text 2).

BGL0

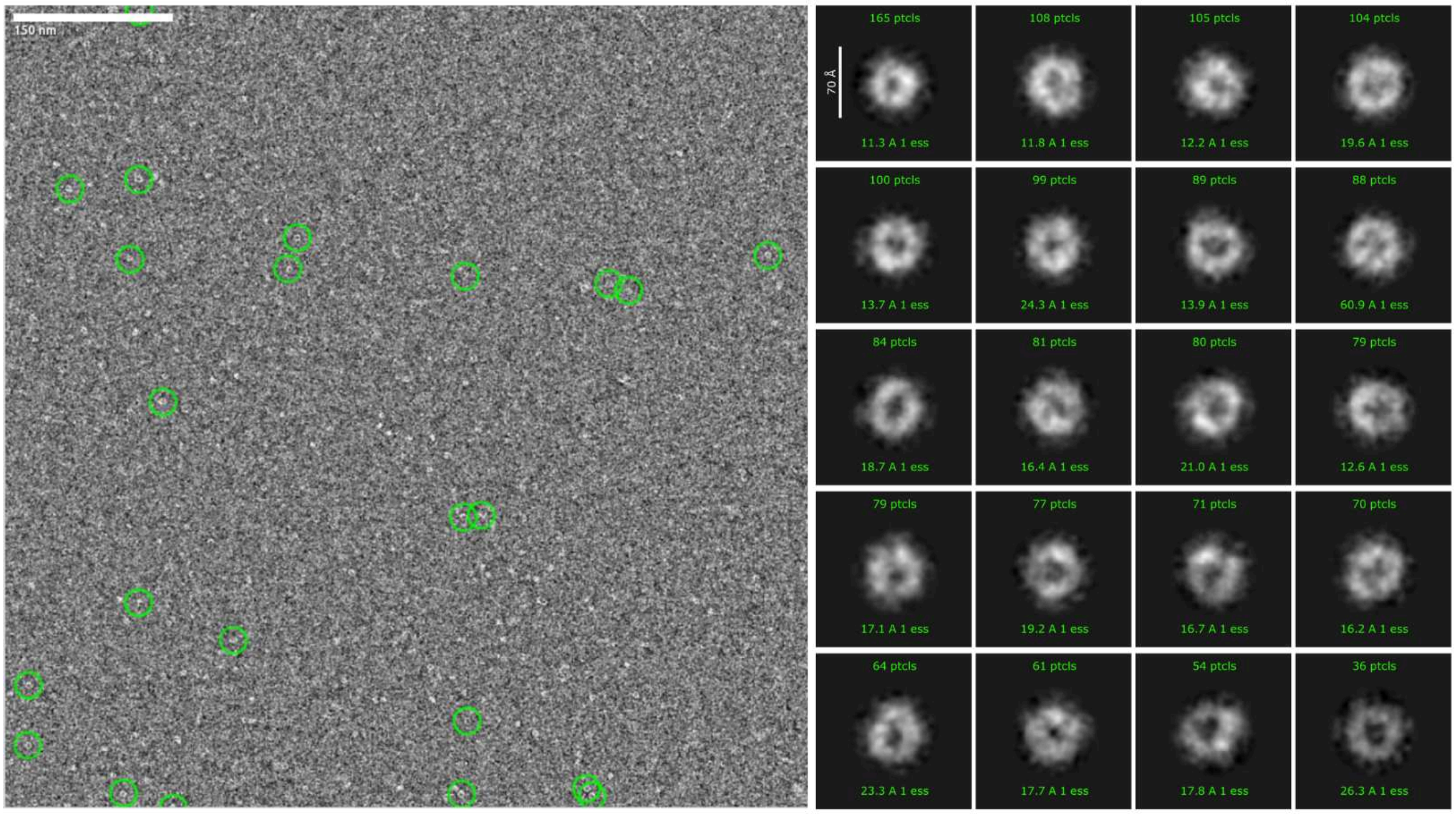

150 nm
70 Å
165 ptcls
11.3 A 1 ess
108 ptcls
11.8 A 1 ess
105 ptcls
12.2 A 1 ess
104 ptcls
19.6 A 1 ess
100 ptcls
13.7 A 1 ess
99 ptcls
24.3 A 1 ess
89 ptcls
13.9 A 1 ess
88 ptcls
60.9 A 1 ess
84 ptcls
18.7 A 1 ess
81 ptcls
16.4 A 1 ess
80 ptcls
21.0 A 1 ess
79 ptcls
12.6 A 1 ess
79 ptcls
17.1 A 1 ess
77 ptcls
19.2 A 1 ess
71 ptcls
16.7 A 1 ess
70 ptcls
16.2 A 1 ess
64 ptcls
23.3 A 1 ess
61 ptcls
17.7 A 1 ess
54 ptcls
17.8 A 1 ess
36 ptcls
26.3 A 1 ess

BGL01

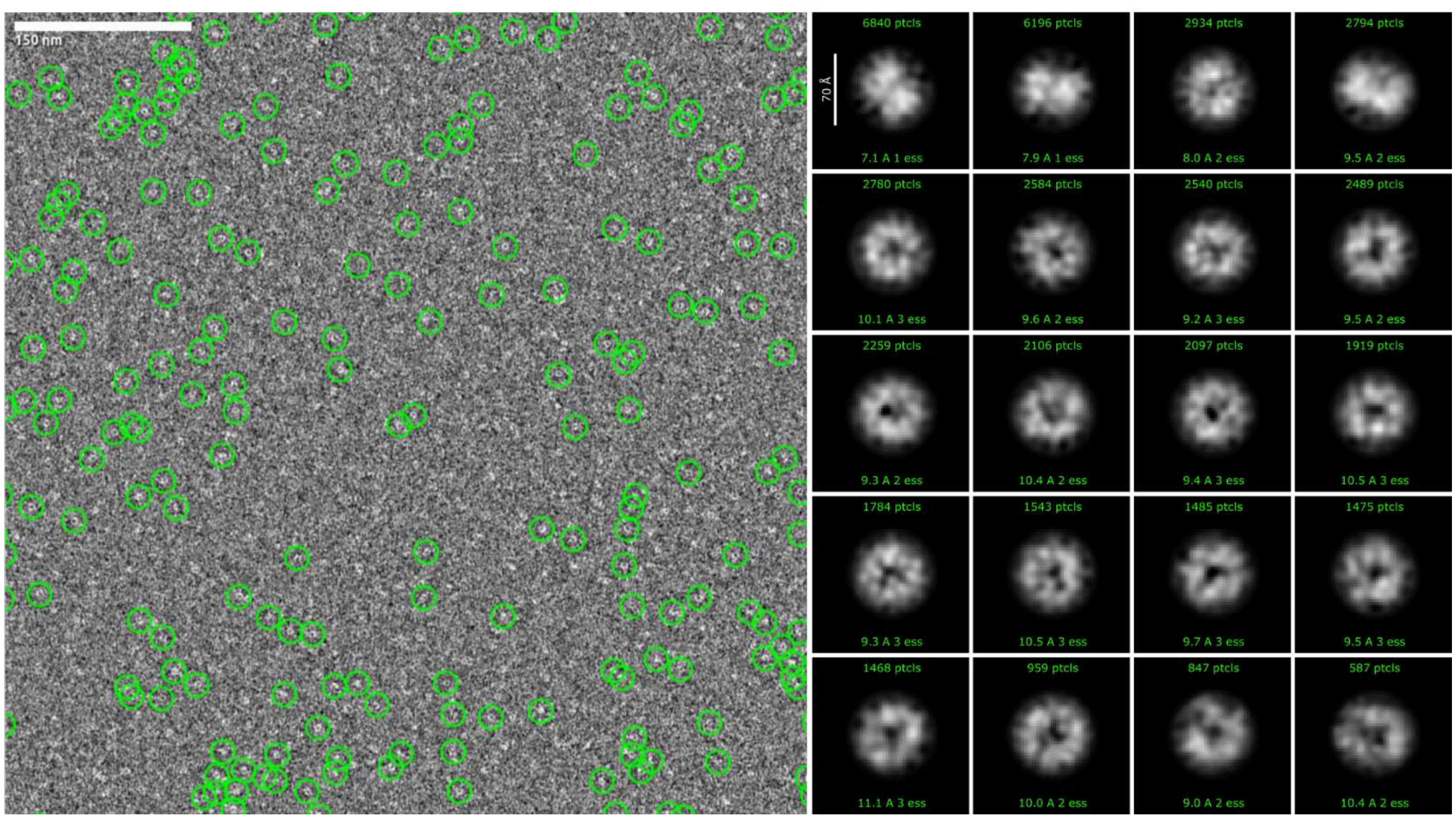

150 nm
70 Å
6840 ptcls
7.1 A 1 ess
6196 ptcls
7.9 A 1 ess
2934 ptcls
8.0 A 2 ess
2794 ptcls
9.5 A 2 ess
2780 ptcls
10.1 A 3 ess
2584 ptcls
9.6 A 2 ess
2540 ptcls
9.2 A 3 ess
2489 ptcls
9.5 A 2 ess
2259 ptcls
9.3 A 2 ess
2106 ptcls
10.4 A 2 ess
2097 ptcls
9.4 A 3 ess
1919 ptcls
10.5 A 3 ess
1784 ptcls
9.3 A 3 ess
1543 ptcls
10.5 A 3 ess
1485 ptcls
9.7 A 3 ess
1475 ptcls
9.5 A 3 ess
1468 ptcls
11.1 A 3 ess
959 ptcls
10.0 A 2 ess
847 ptcls
9.0 A 2 ess
587 ptcls
10.4 A 2 ess

BGL02

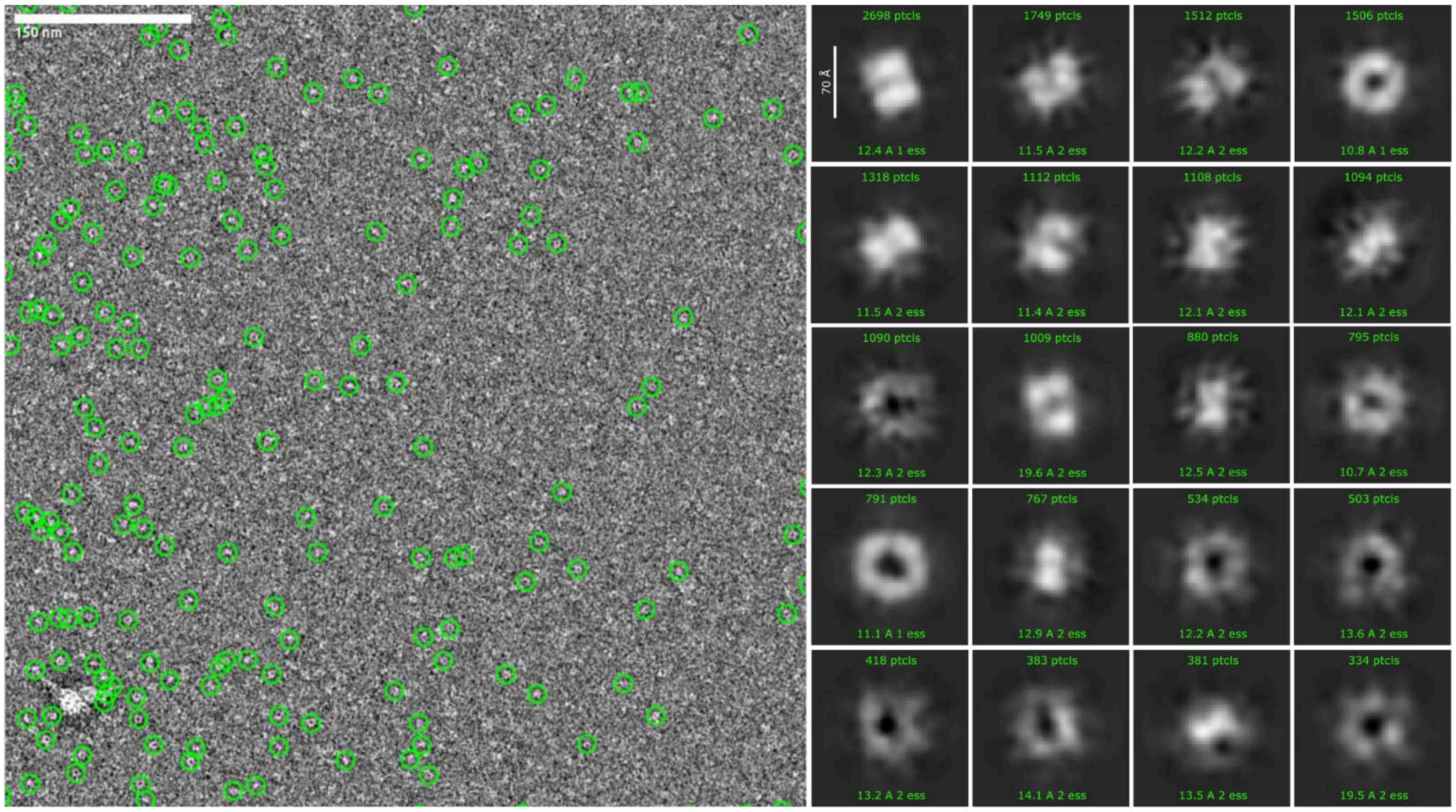
150 nm
70 Å
2698 ptcls
12.4 A 1 ess
1749 ptcls
11.5 A 2 ess
1512 ptcls
12.2 A 2 ess
1506 ptcls
10.8 A 1 ess
1318 ptcls
11.5 A 2 ess
1112 ptcls
11.4 A 2 ess
1108 ptcls
12.1 A 2 ess
1094 ptcls
12.1 A 2 ess
1090 ptcls
12.3 A 2 ess
1009 ptcls
19.6 A 2 ess
880 ptcls
12.5 A 2 ess
795 ptcls
10.7 A 2 ess
791 ptcls
11.1 A 1 ess
767 ptcls
12.9 A 2 ess
534 ptcls
12.2 A 2 ess
503 ptcls
13.6 A 2 ess
418 ptcls
13.2 A 2 ess
383 ptcls
14.1 A 2 ess
381 ptcls
13.5 A 2 ess
334 ptcls
19.5 A 2 ess

BGL03

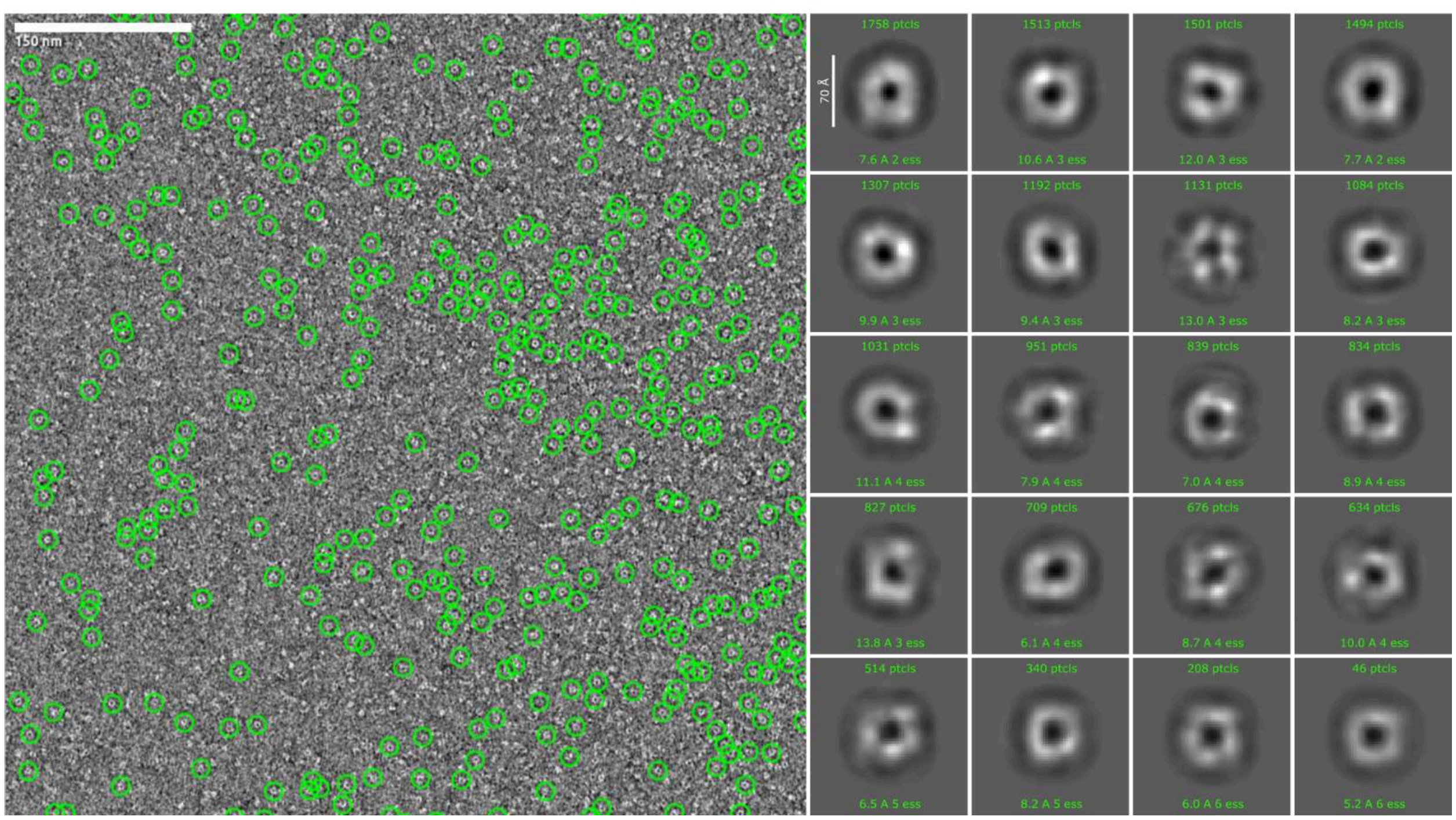
150 nm
70 Å
1758 ptcls
7.6 A 2 ess
1513 ptcls
10.6 A 3 ess
1501 ptcls
12.0 A 3 ess
1494 ptcls
7.7 A 2 ess
1307 ptcls
9.9 A 3 ess
1192 ptcls
9.4 A 3 ess
1131 ptcls
13.0 A 3 ess
1084 ptcls
8.2 A 3 ess
1031 ptcls
11.1 A 4 ess
951 ptcls
7.9 A 4 ess
839 ptcls
7.0 A 4 ess
834 ptcls
8.9 A 4 ess
827 ptcls
13.8 A 3 ess
709 ptcls
6.1 A 4 ess
676 ptcls
8.7 A 4 ess
634 ptcls
10.0 A 4 ess
514 ptcls
6.5 A 5 ess
340 ptcls
8.2 A 5 ess
208 ptcls
6.0 A 6 ess
46 ptcls
5.2 A 6 ess

BGL04 -- no data

BGL05

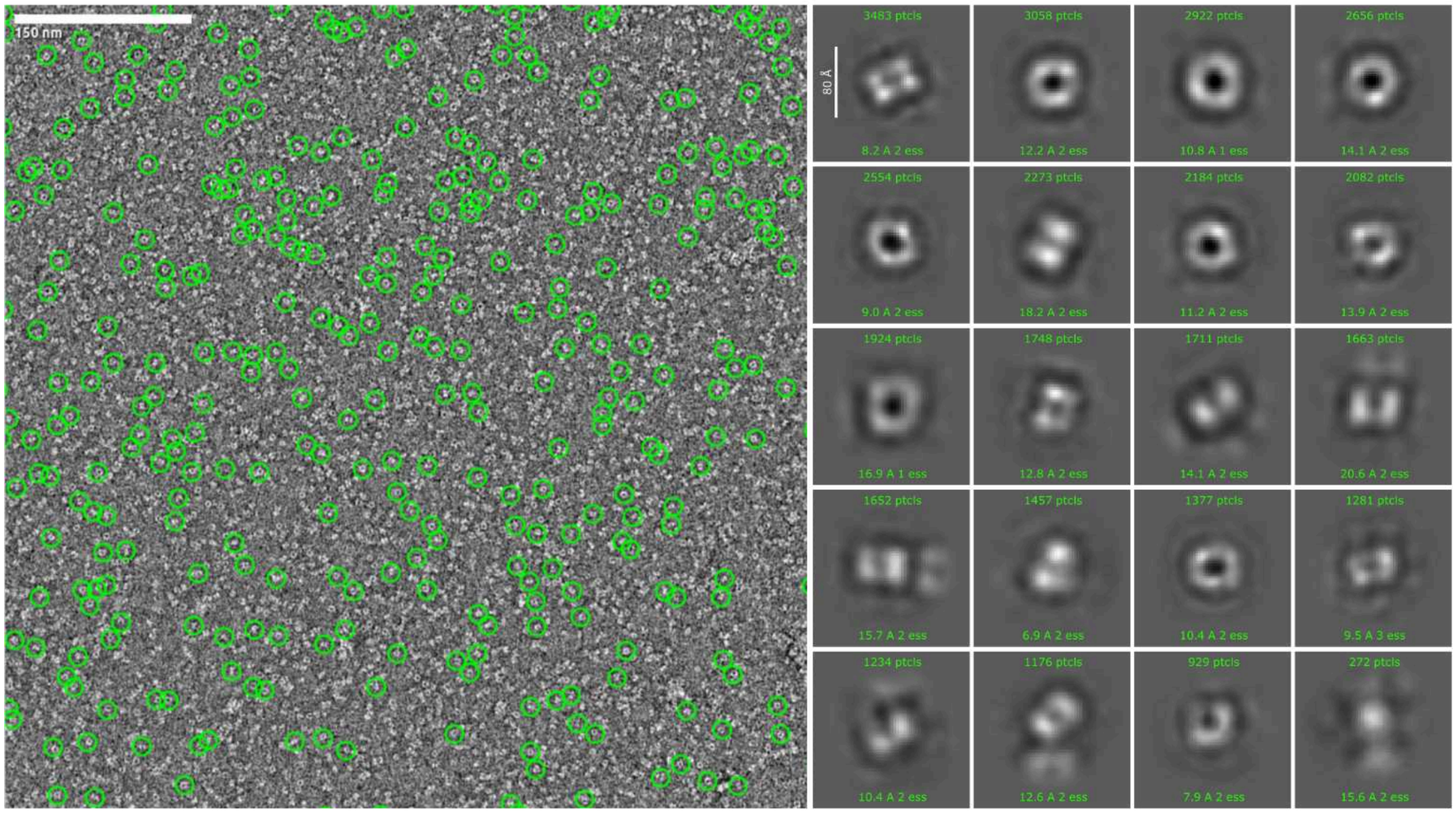

BGL06

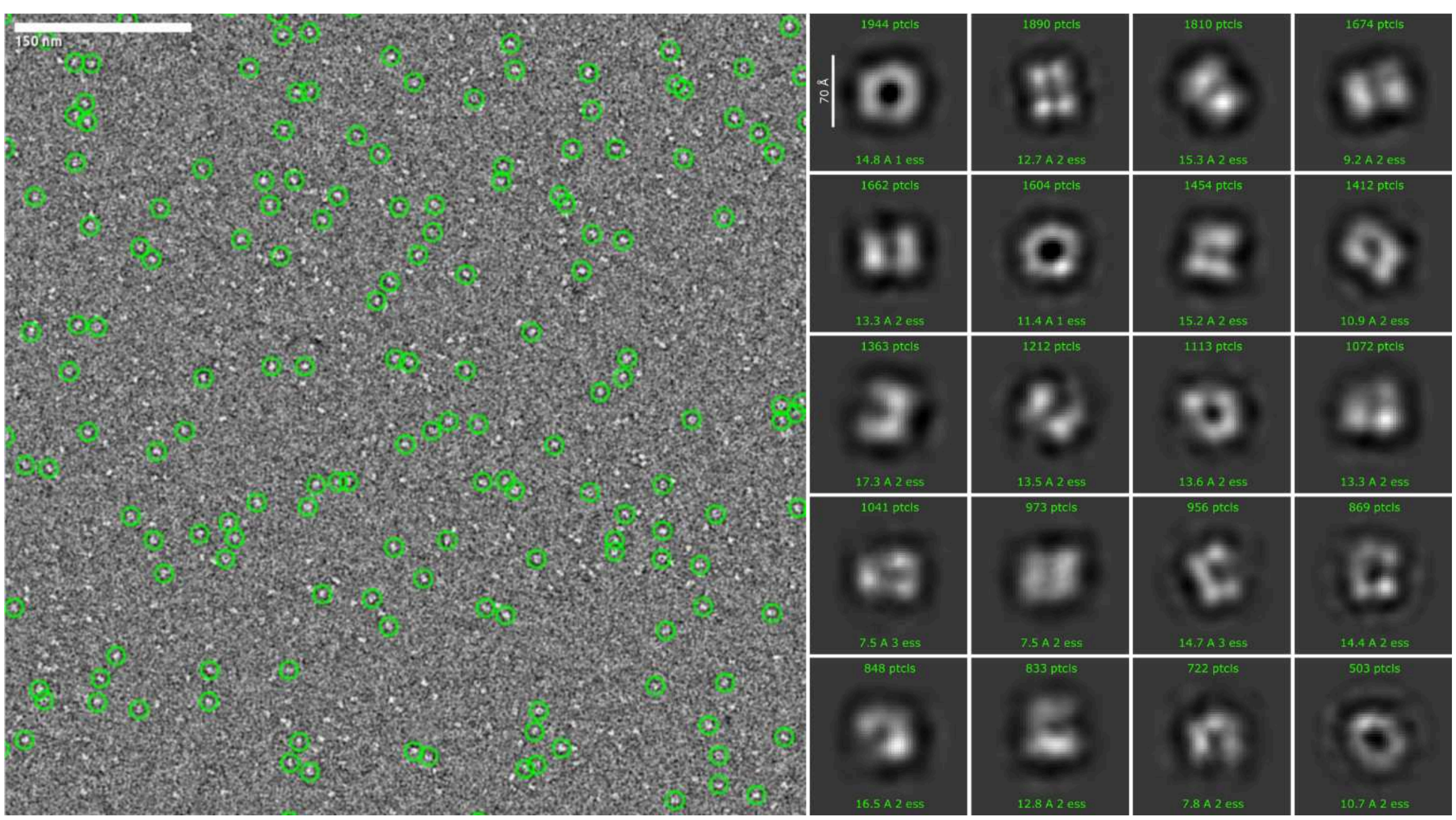

BGL07 -- no data

BGL08

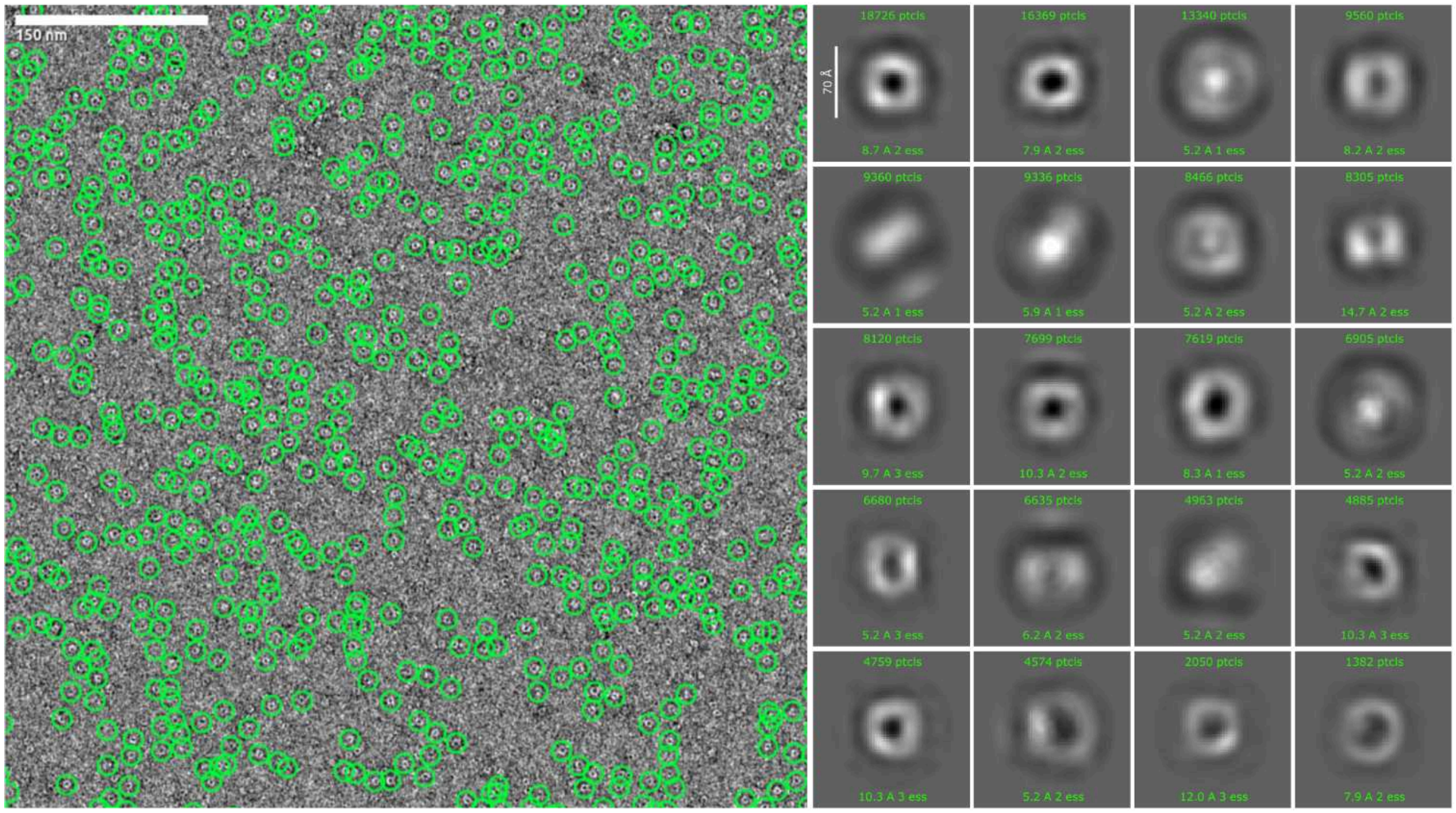

BGL09

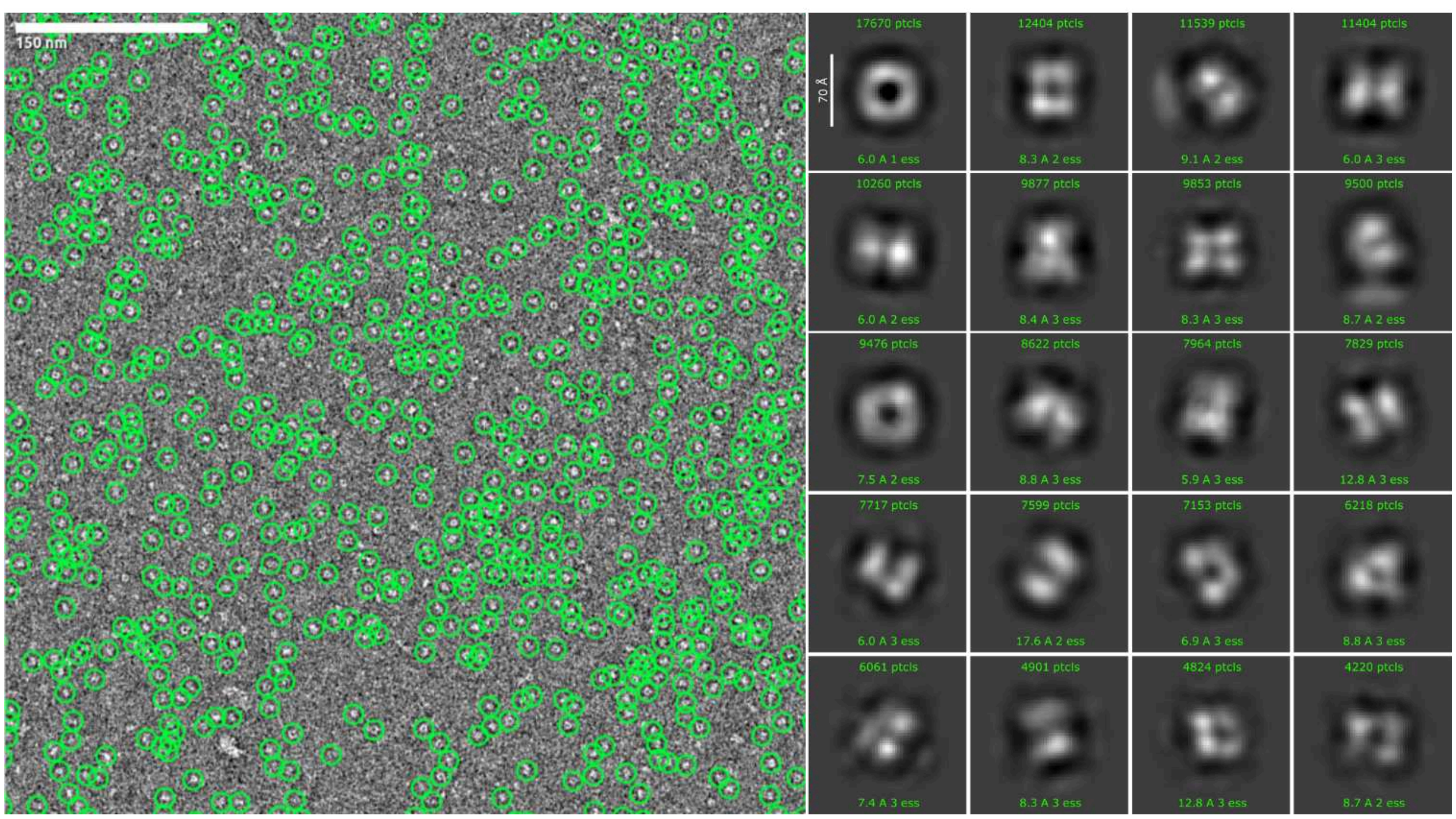

BGL10-13 -- no data

BGL14

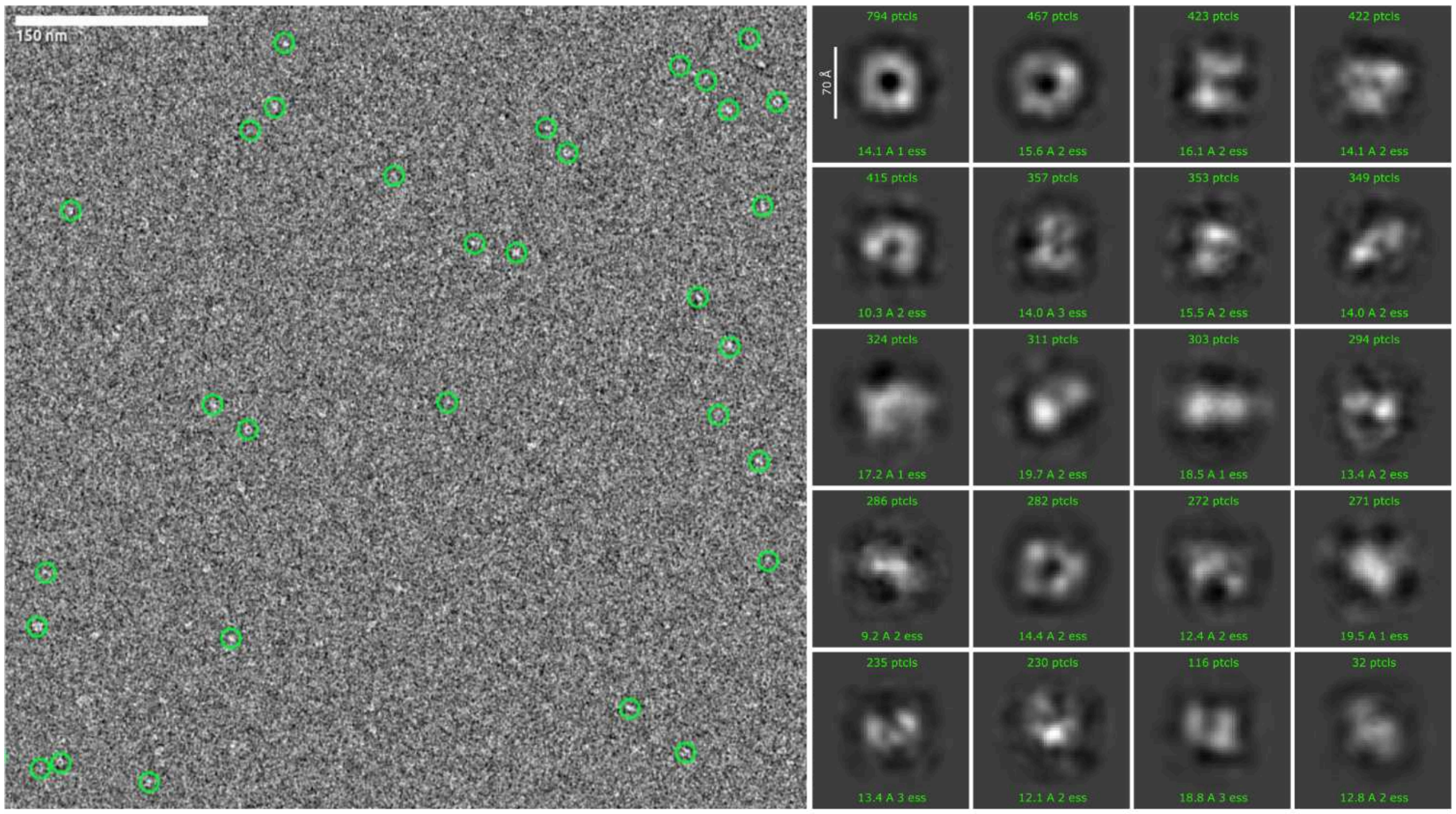

BGL15

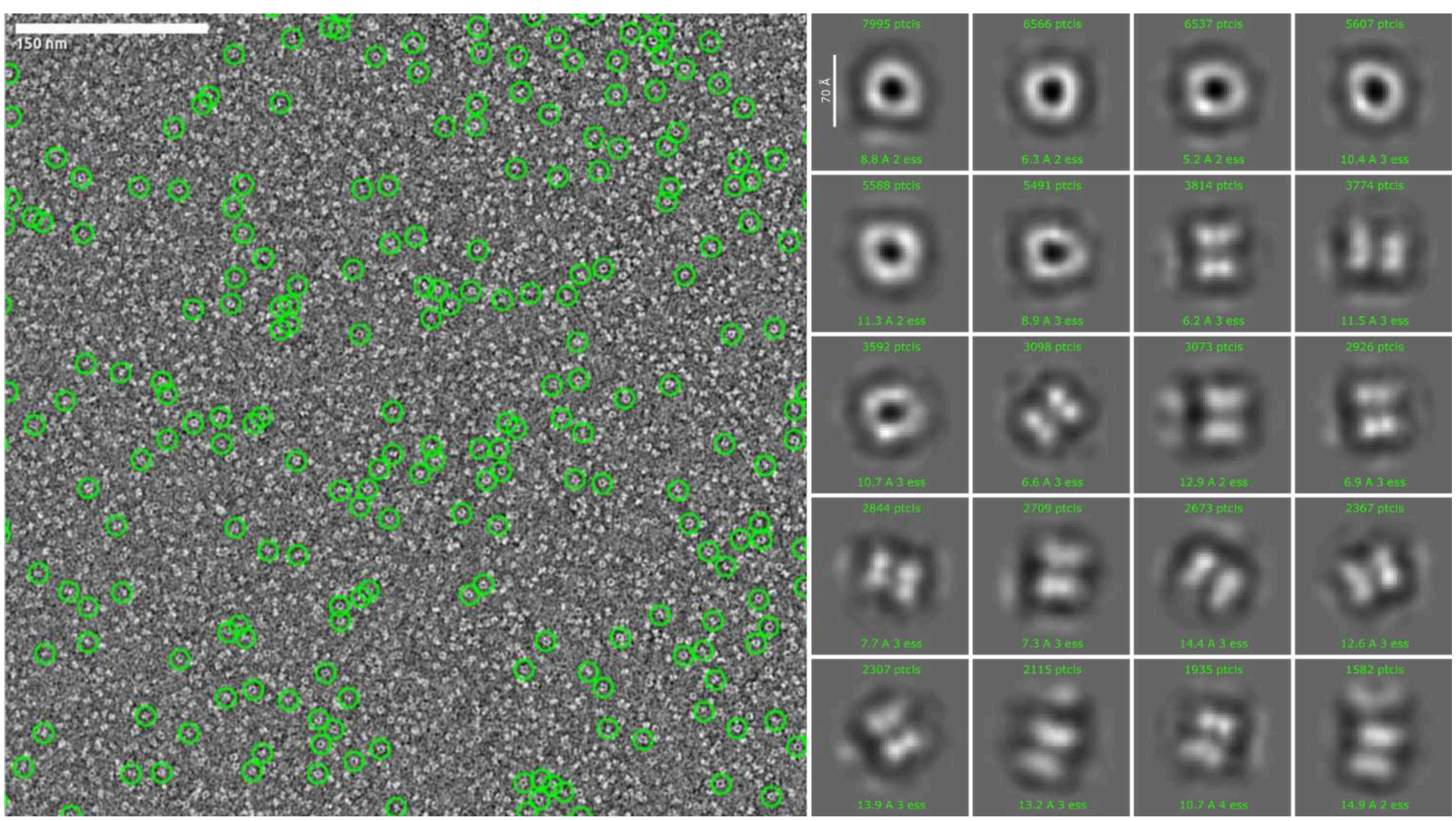

BGL16 -- no data

BGL17

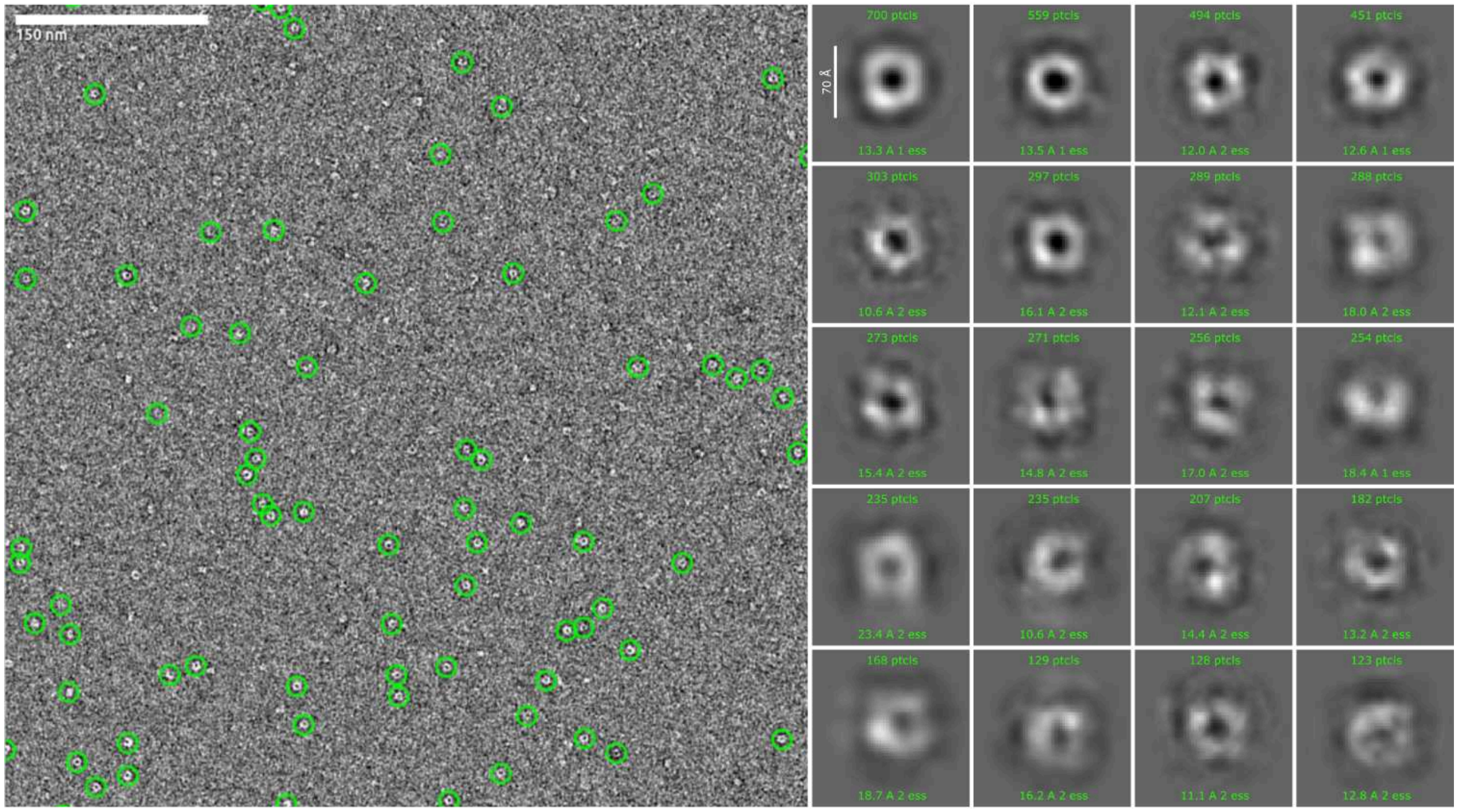

BGL18

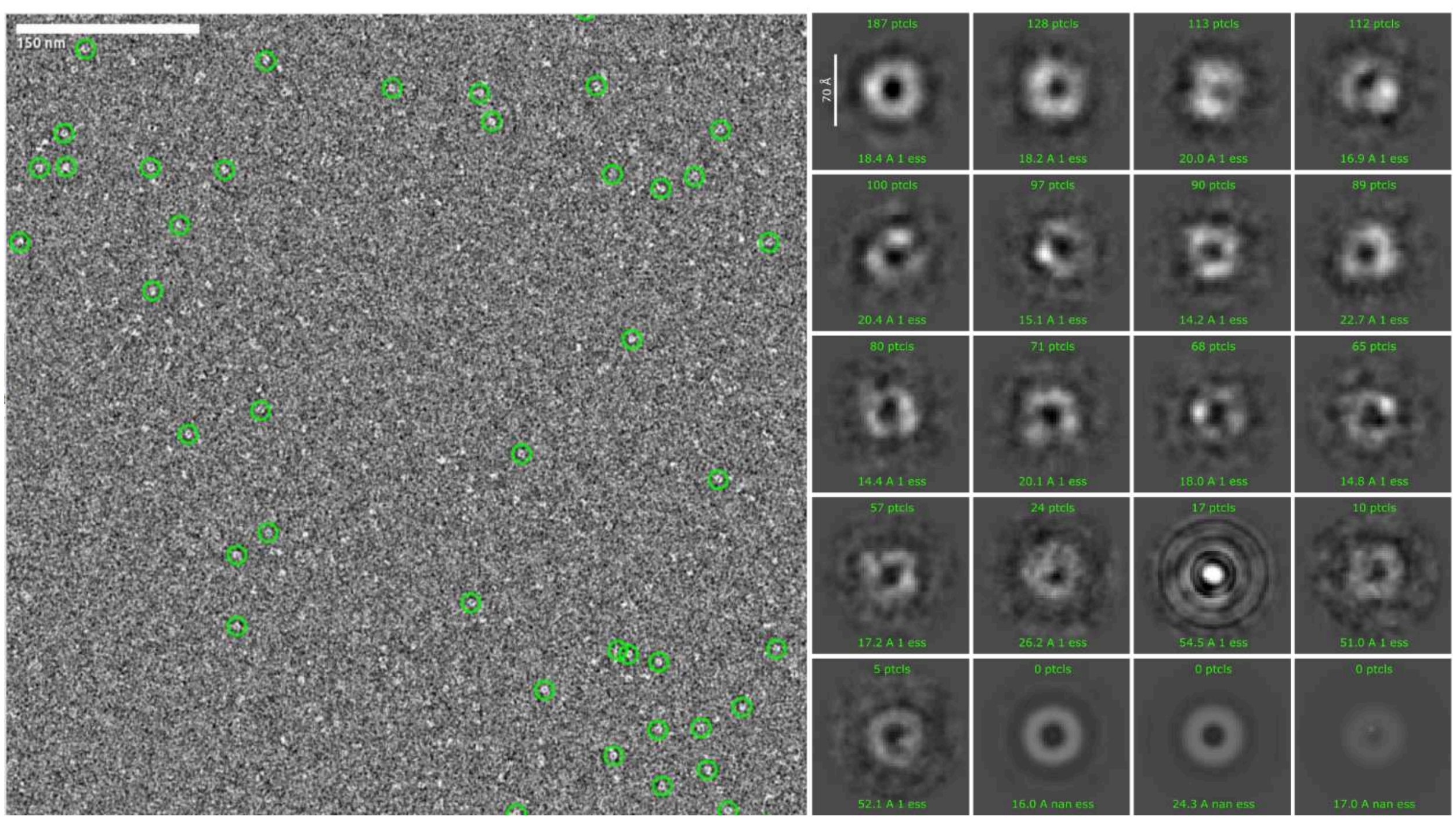

BGL19

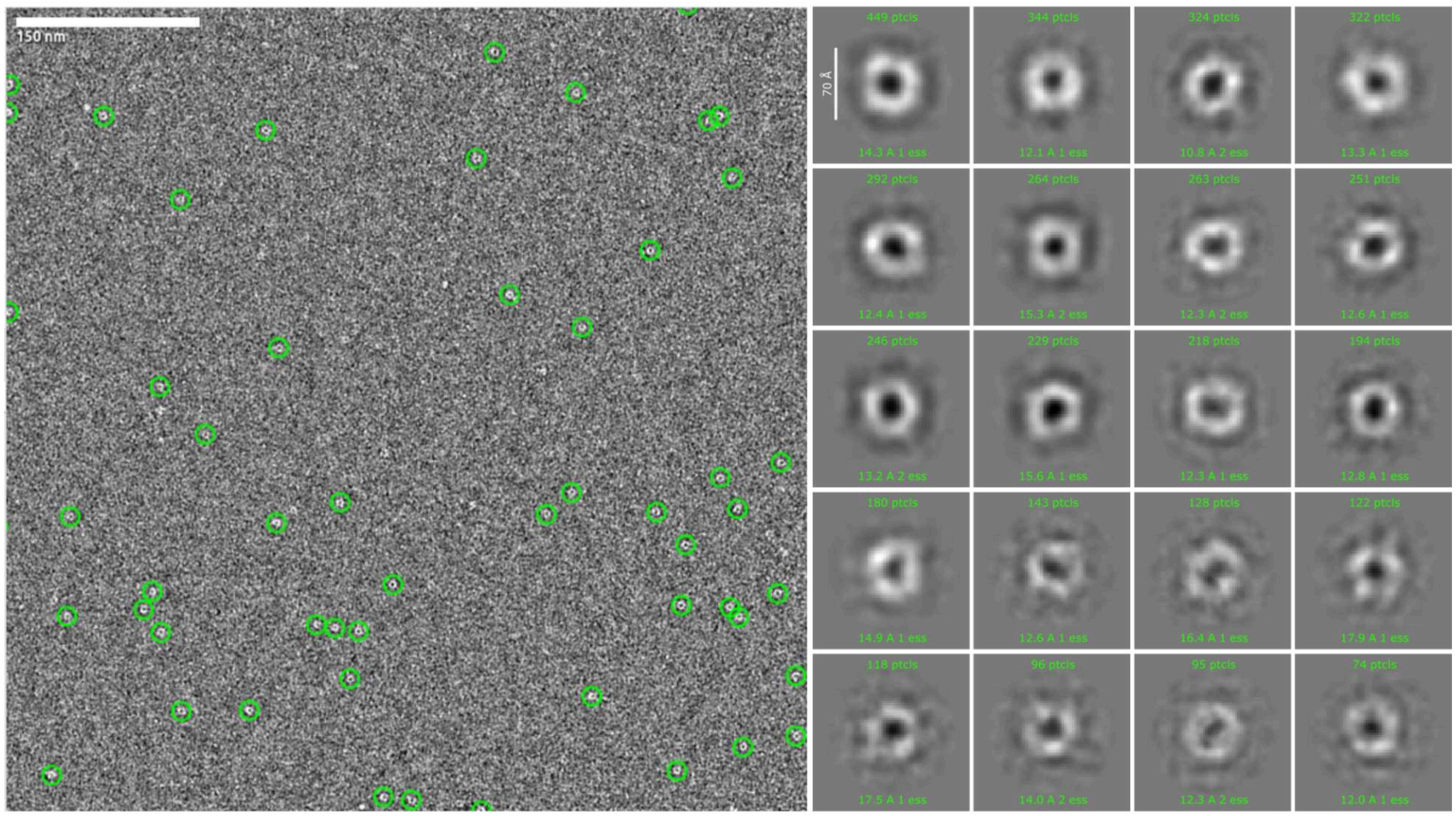

BGL20 -- no data

Supplementary Figure 5: nsEM micrographs and 2D class averages of BGL homotrimers
For each design, a representative micrograph (left) with circled particles included in the dataset used to construct 2D class averages (right). Scale is indicated by labeled scale bars on the micrograph and the first class. Most datasets were collected a single time, with between 50 and 200 micrographs.

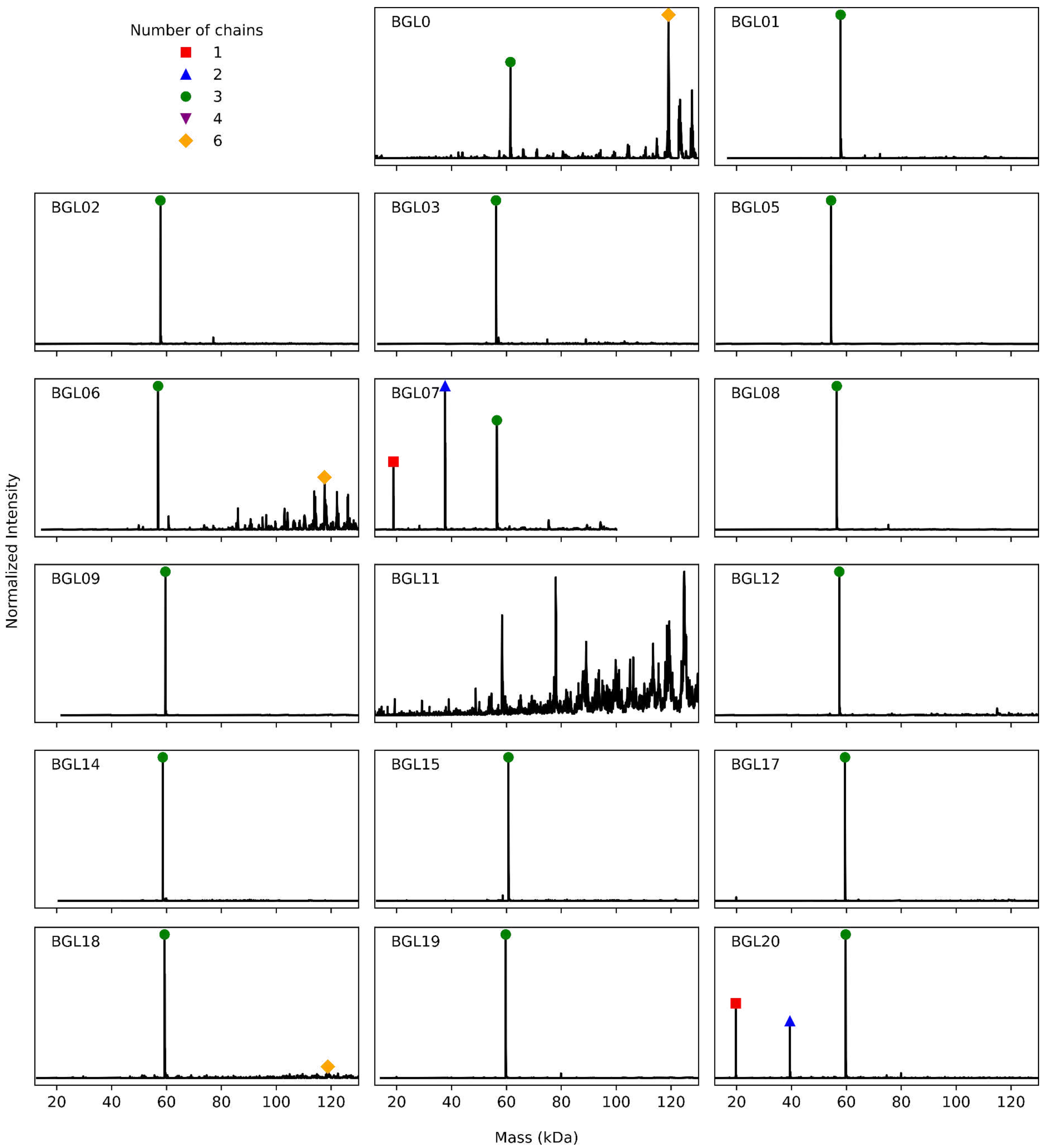

Supplementary Figure 6: nMS of BGL homotrimers

Plots of all designs with nMS data. Black, deconvoluted masses obtained from m/z spectra (Supplementary Data 13). Identified species corresponding to monomer (red square), dimer (blue triangle), trimer (green circle), tetramer (purple upside-down triangle), and hexamer (yellow diamond) are annotated; other un-annotated peaks are unidentified species with masses that do not match expected oligomeric states.

BGL0

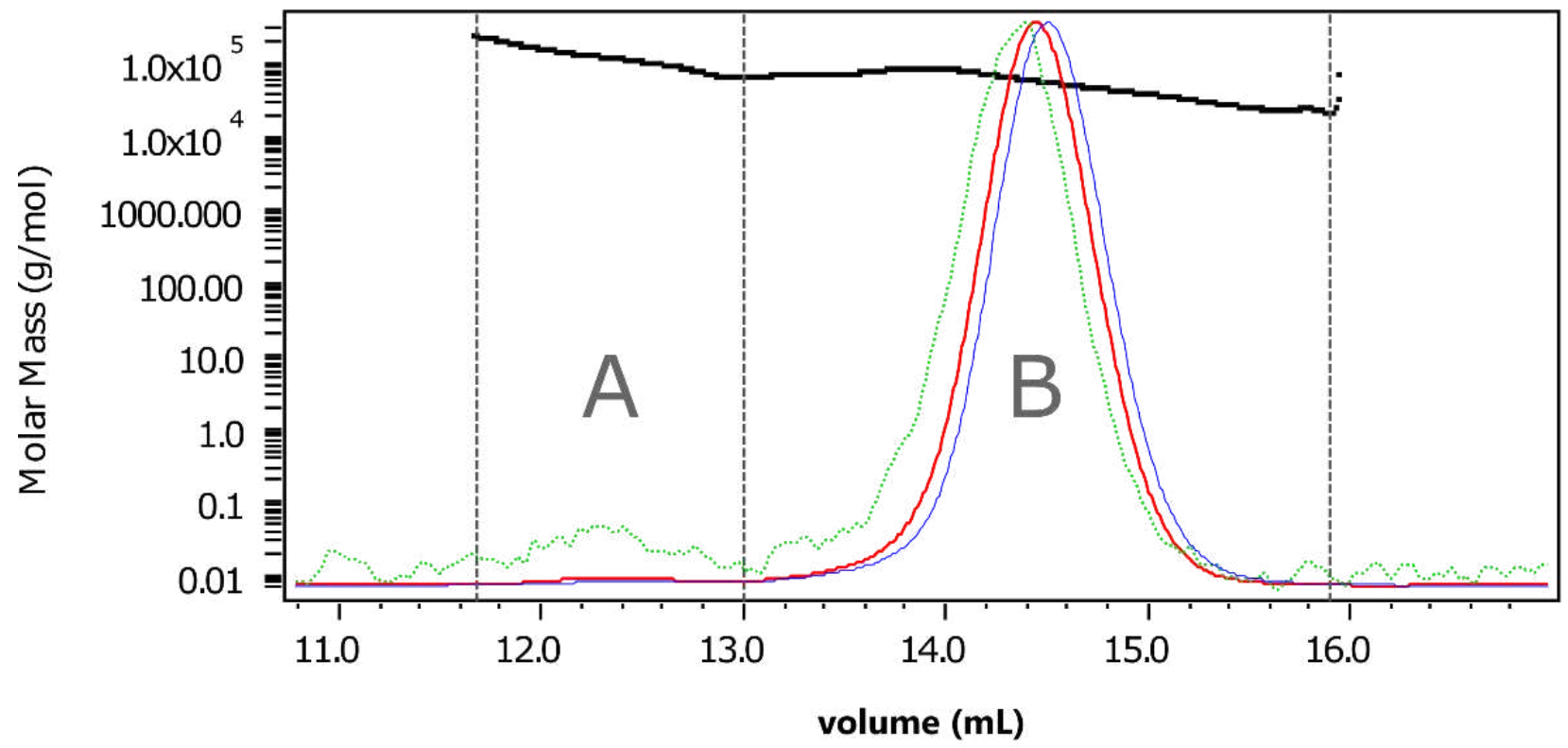

Molar Mass LS UV RI

A: 113.8 kDa(±5.061%)
B: 56.62 kDa(±1.185%)
Batch 2

BGL01

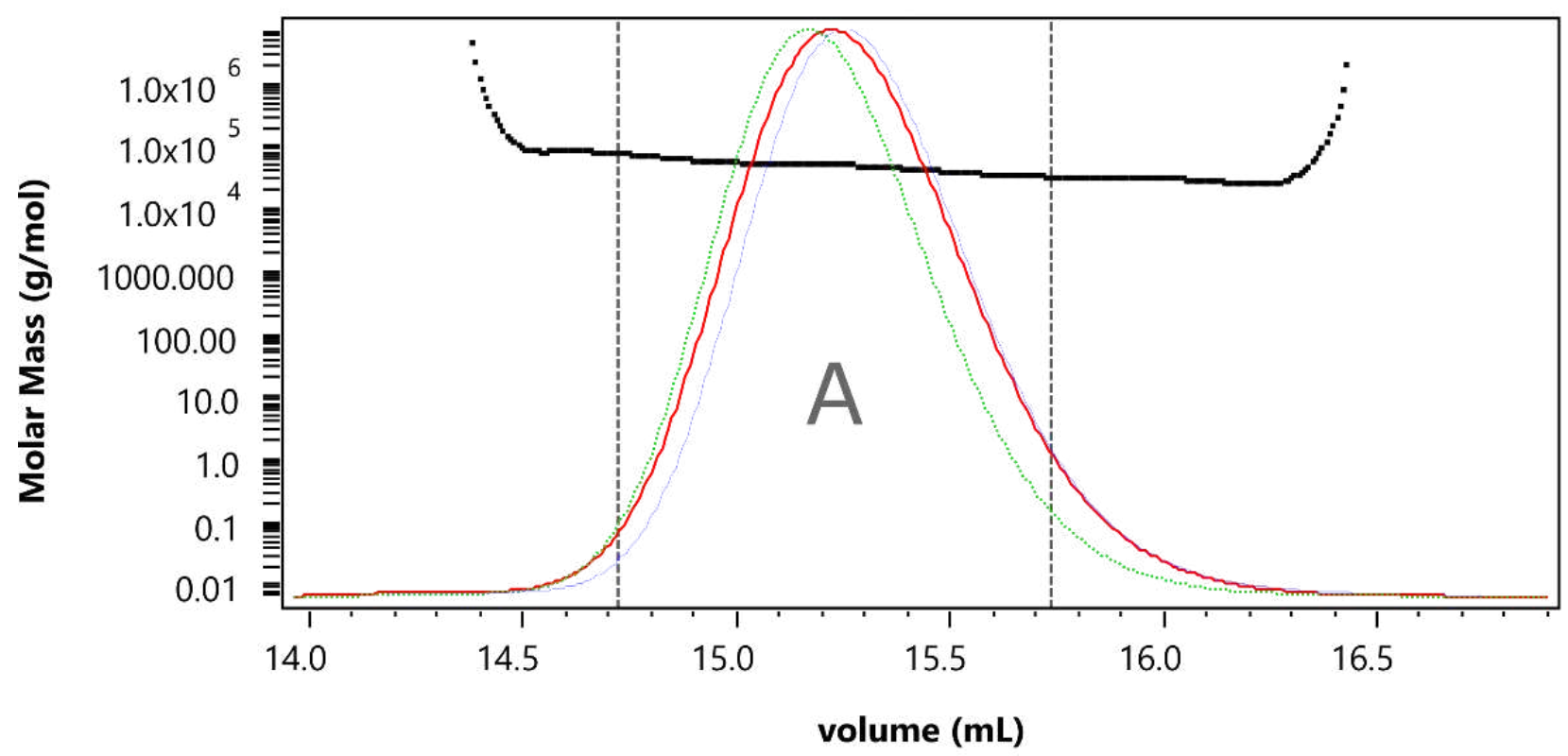

Molar Mass LS UV RI

A: 48.29 kDa(±17.793%)
Batch 1

BGL02

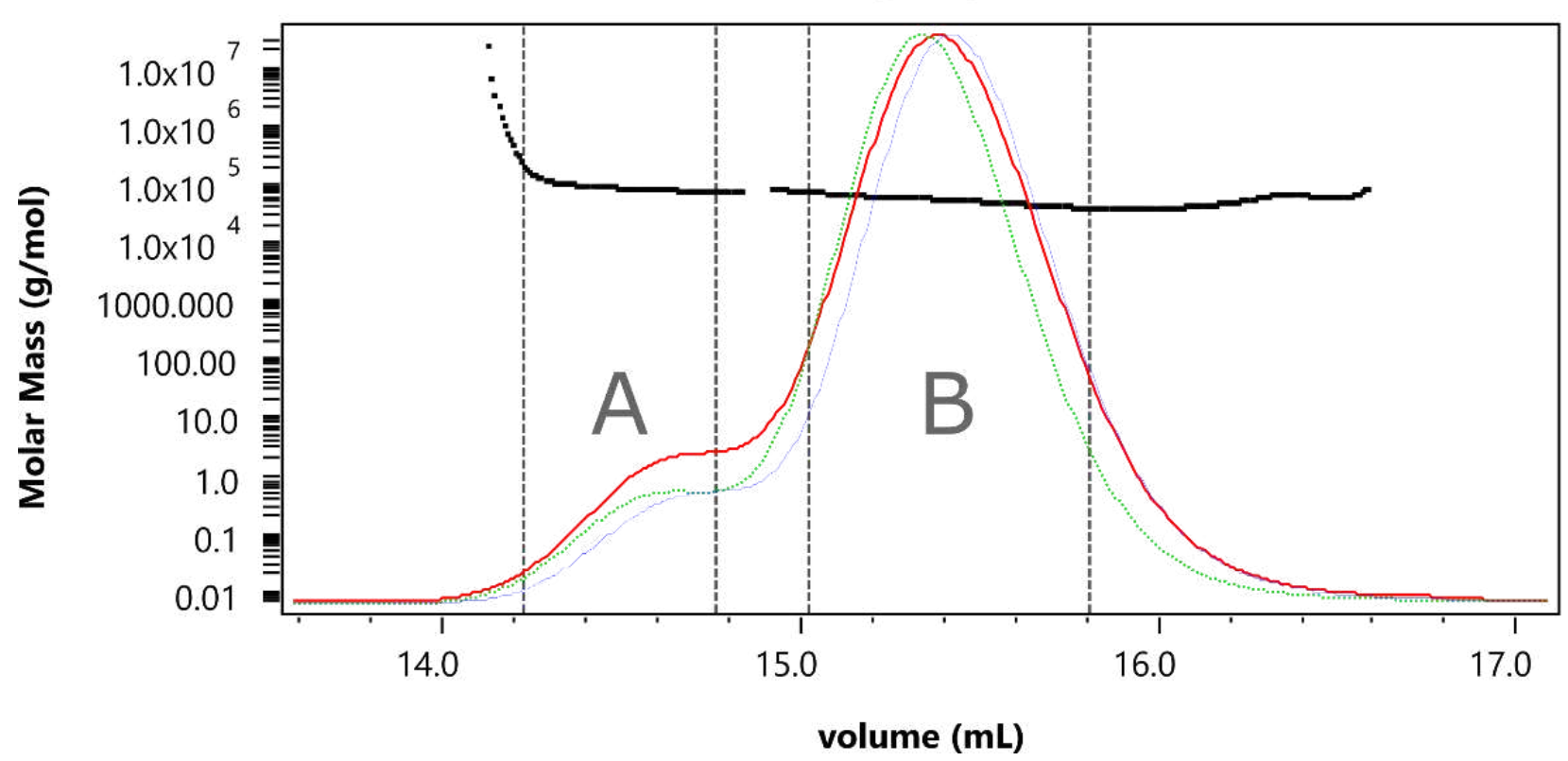

Molar Mass LS UV RI

A: 79.38 kDa(±19.56%)
B: 52.07 kDa(±16.28%)
Batch 1

BGL03

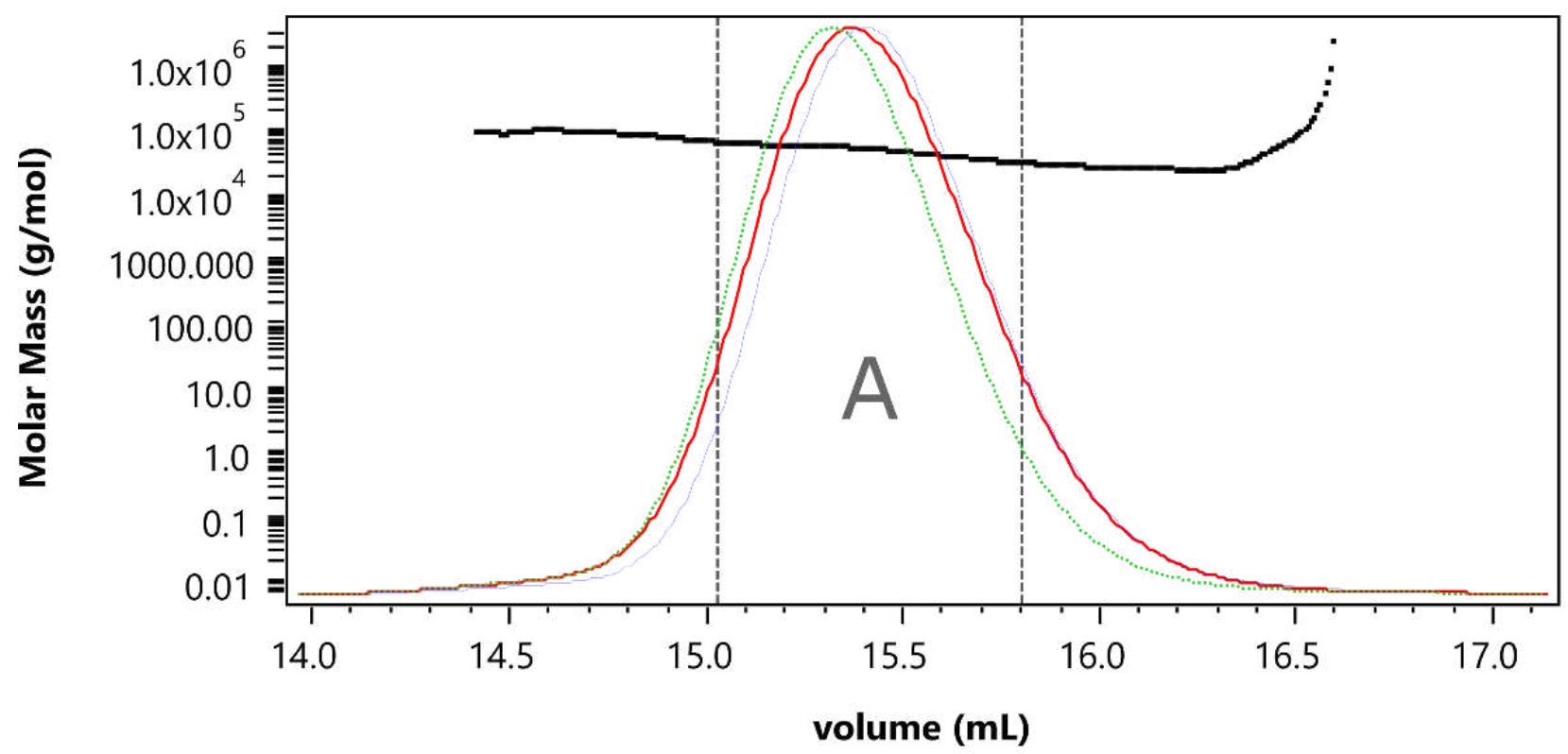

· Molar Mass — LS --- UV — RI

A: 49.10 kDa(±20.00%)
Batch 1

BGL05

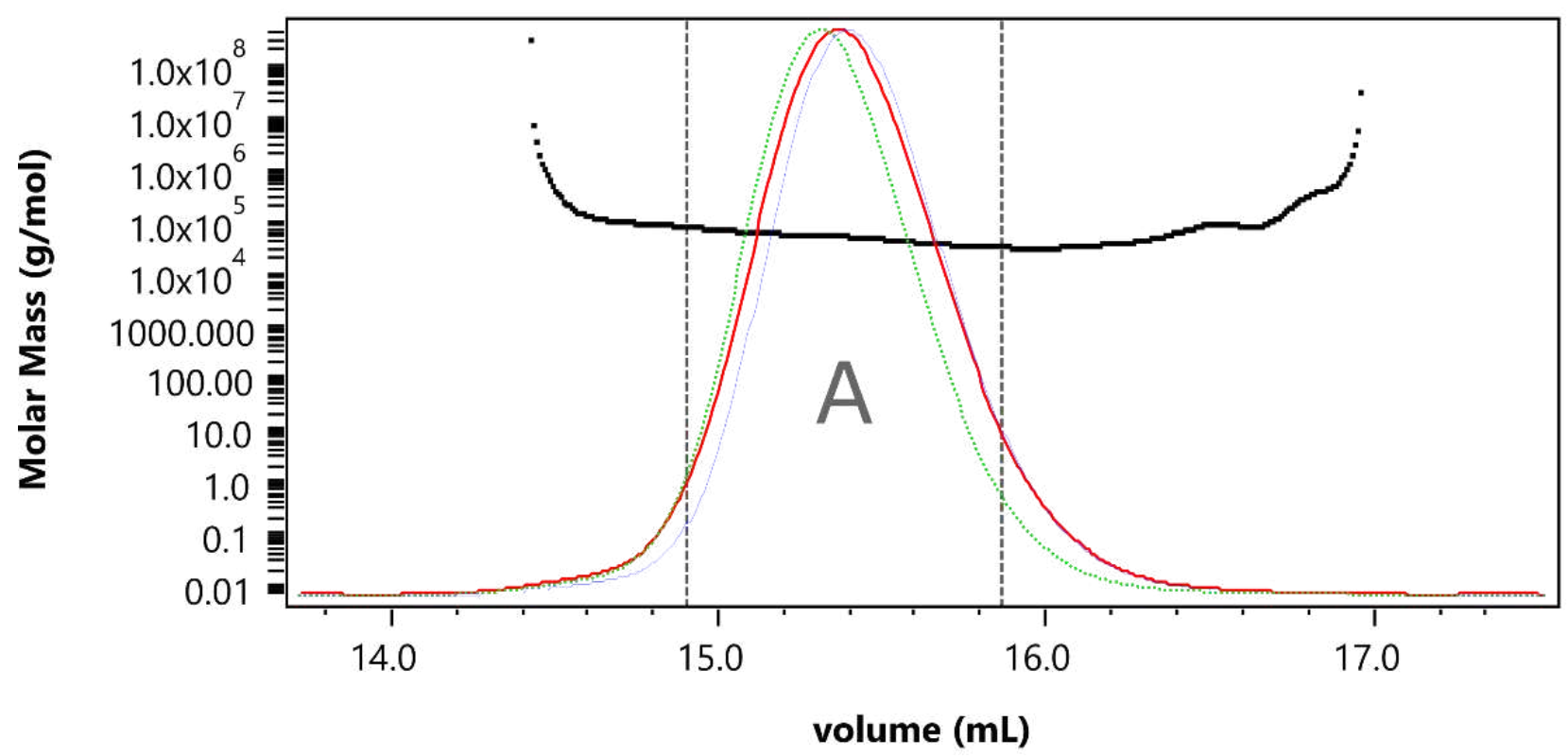

· Molar Mass — LS --- UV — RI

A: 47.63 kDa(±19.33%)
Batch 1

BGL06

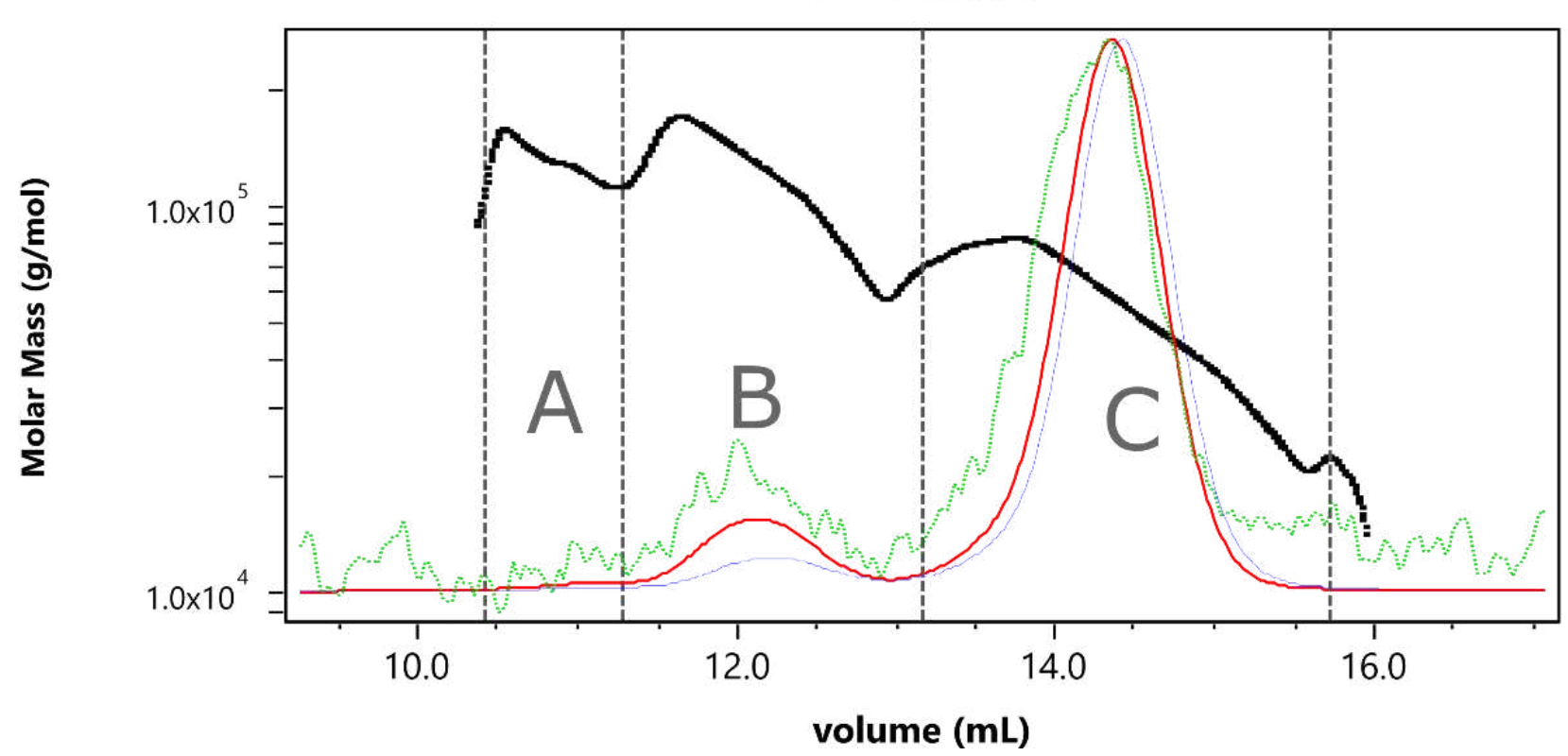

· Molar Mass — LS --- UV — RI

A: 123.6 kDa(±6.094%)
B: 115.7 kDa(±1.158%)
C: 57.83 kDa(±1.120%)
Batch 2

BGL08

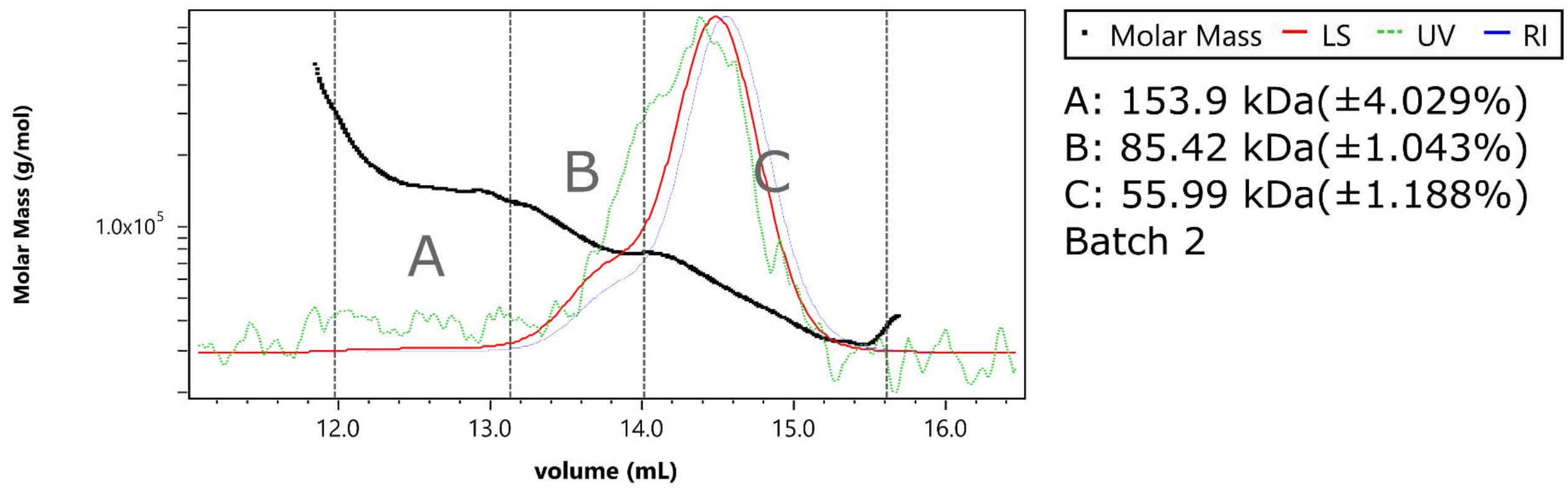

A: 153.9 kDa(±4.029%)
B: 85.42 kDa(±1.043%)
C: 55.99 kDa(±1.188%)
Batch 2

BGL09

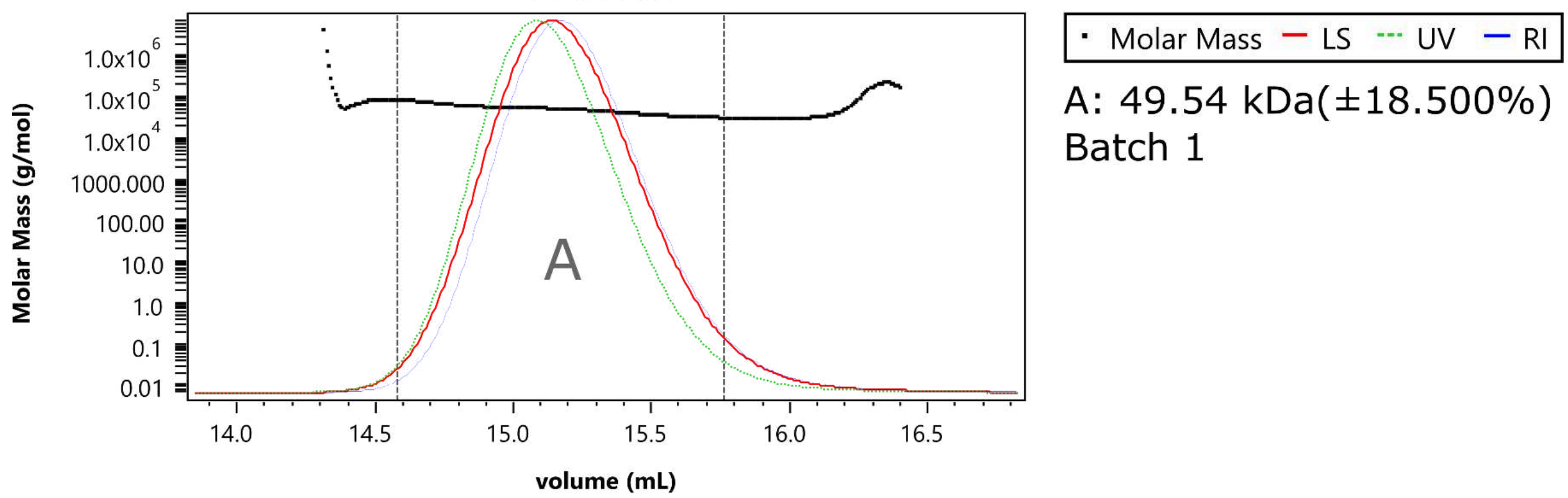

A: 49.54 kDa(±18.500%)
Batch 1

BGL14

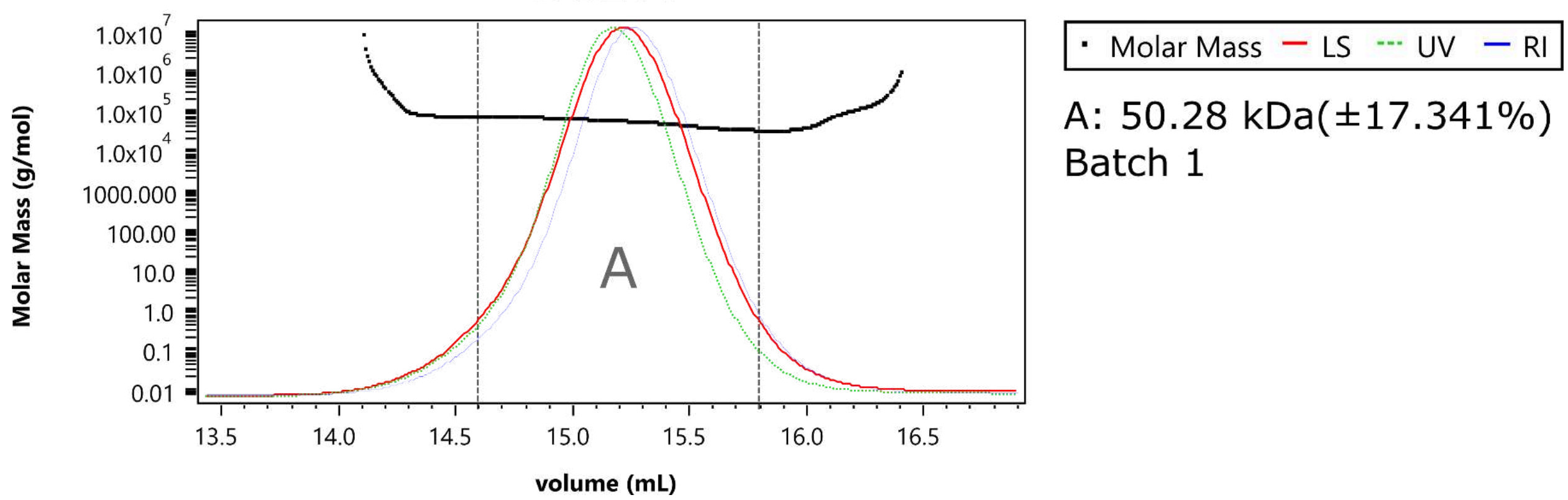

A: 50.28 kDa(±17.341%)
Batch 1

BGL15

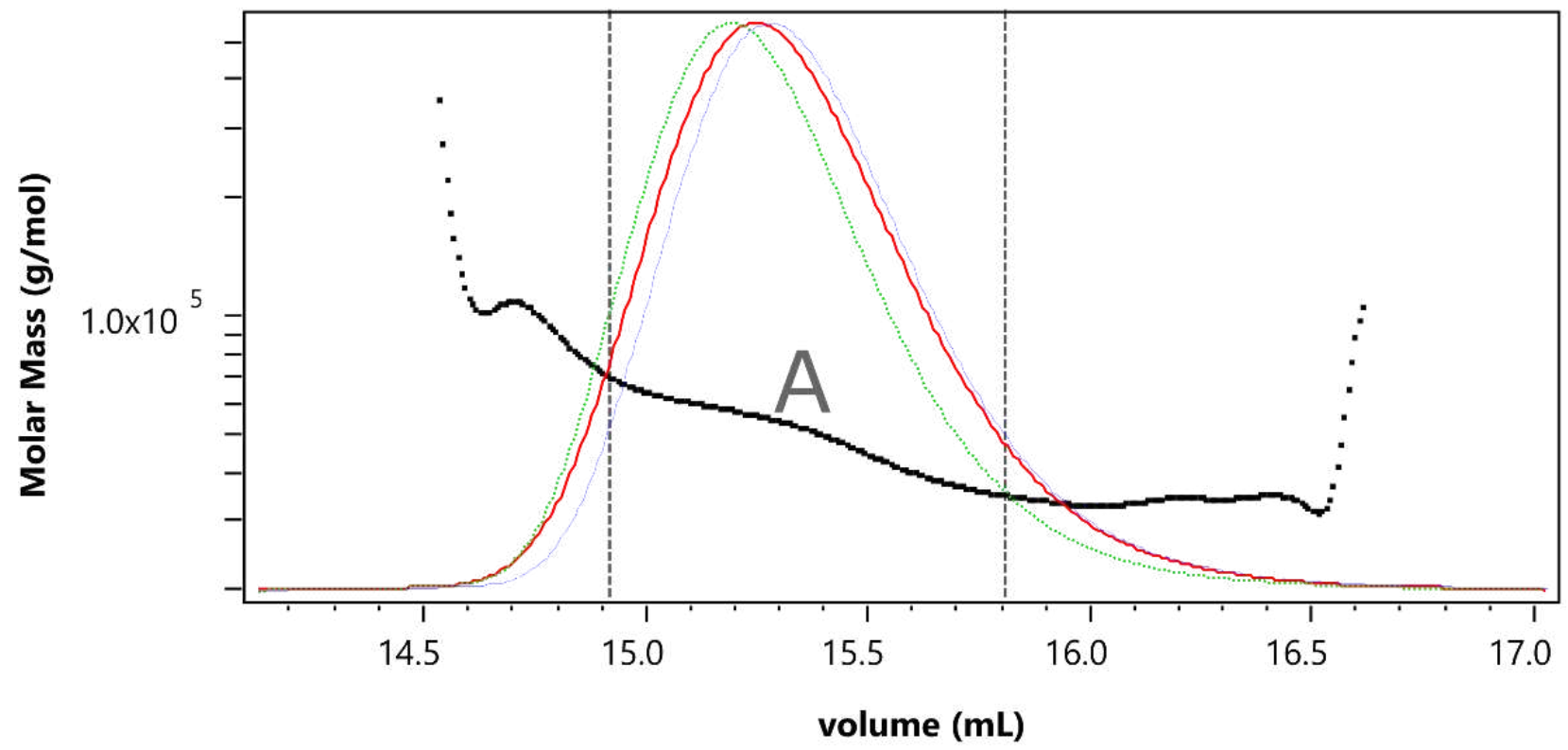

A: 52.69 kDa(±18.100%)
Batch 1

BGL17

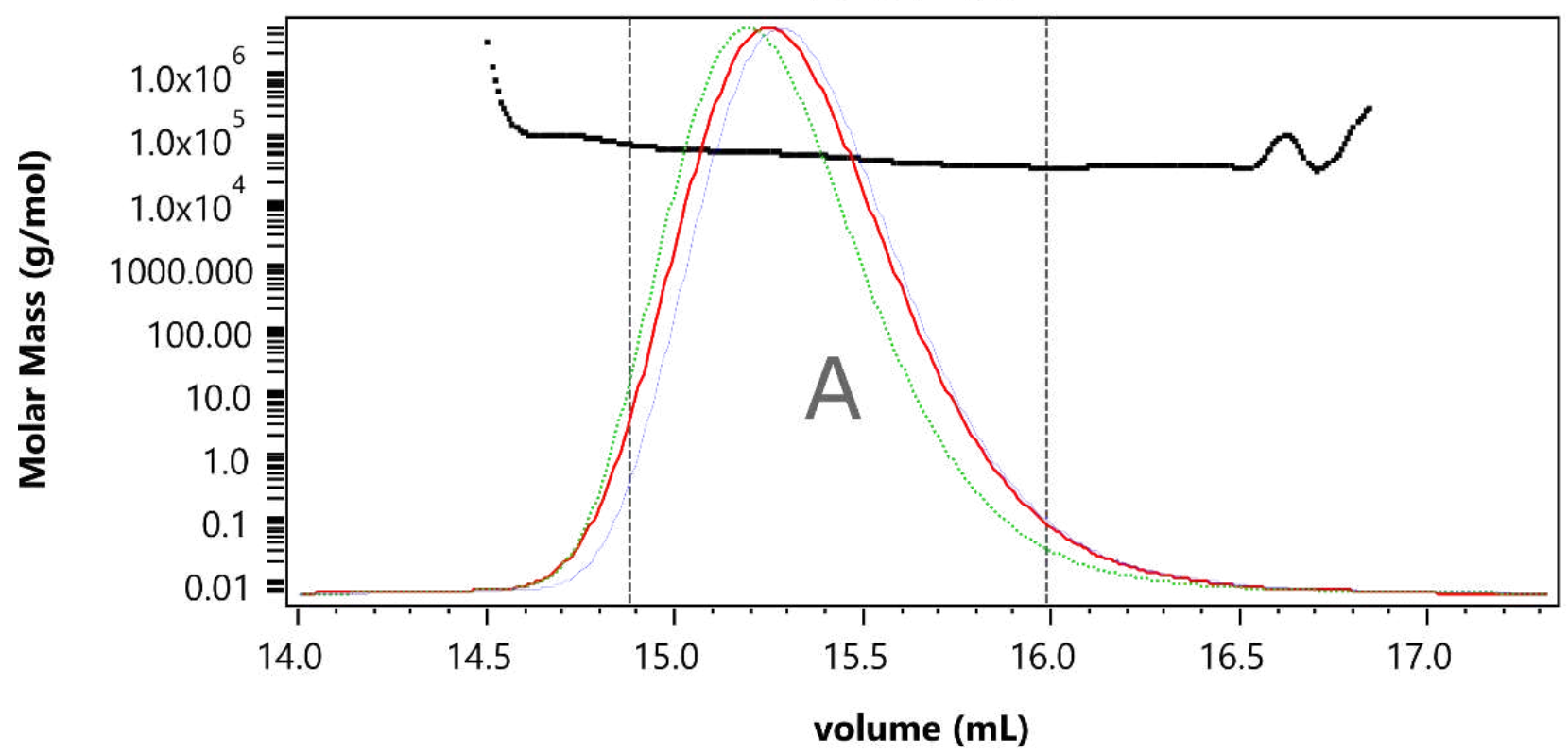

A: 51.94 kDa(±17.252%)
Batch 1

BGL18

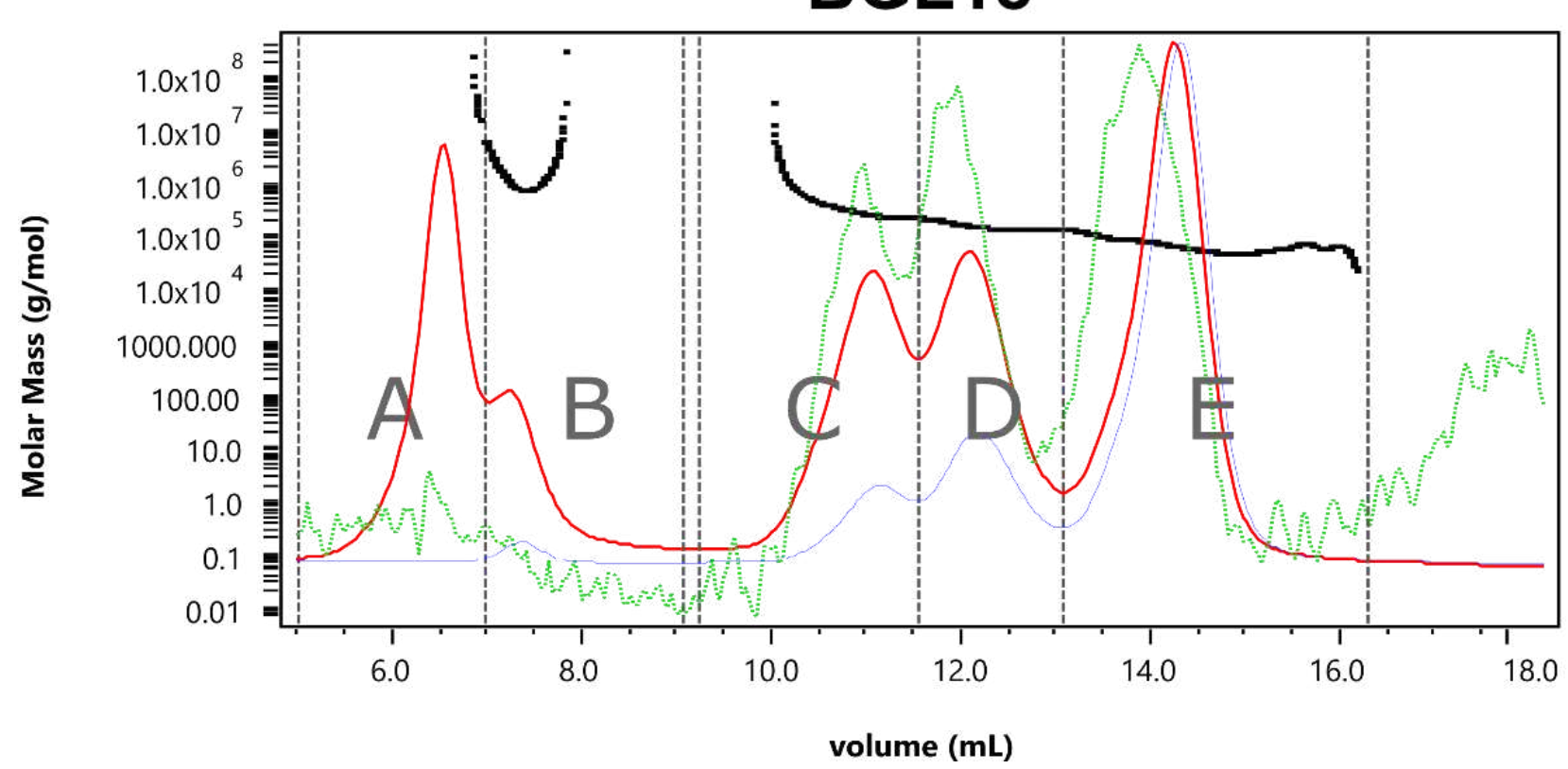

A: 32.83 MDa(±1.756%)
B: 1.196 MDa(±1.452%)
C: 279.9 kDa(±1.081%)
D: 152.0 kDa(±1.014%)
E: 65.82 kDa(±1.148%)
Batch 2

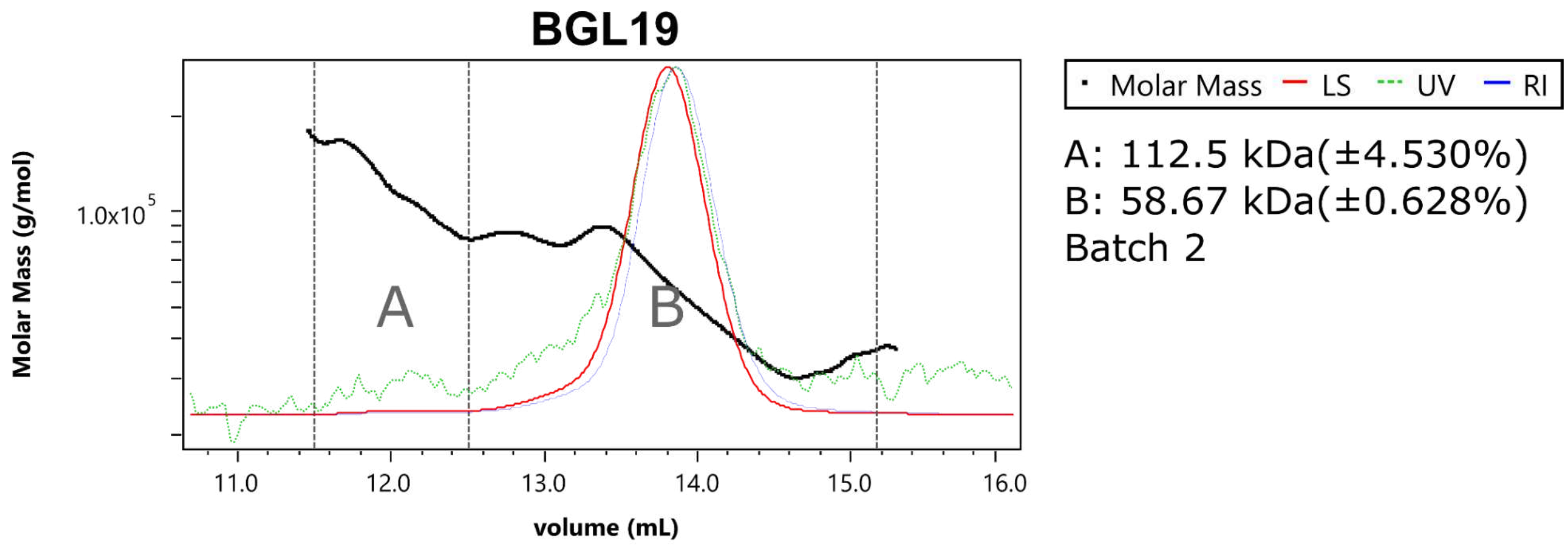

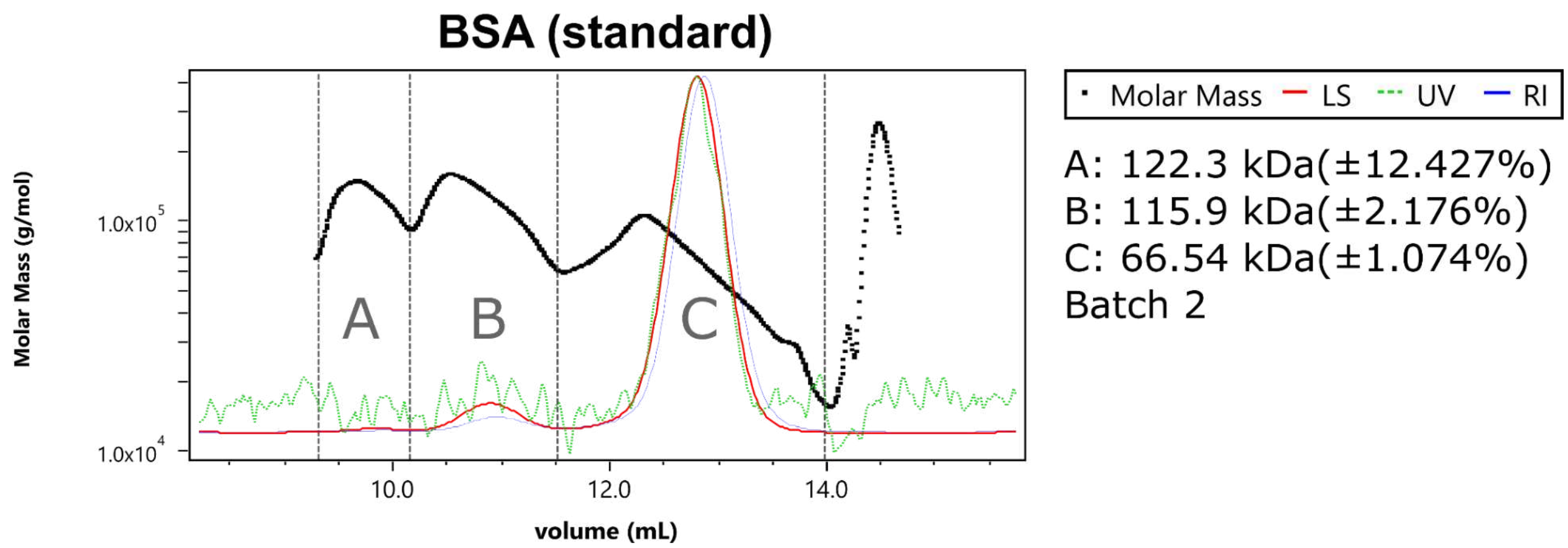

Supplementary Figure 7: SEC-MALS of BGL homotrimers

Plots of all designs with SEC-MALS data. Red, light scattering signal. Green, UV absorbance. Blue, differential refractive index (dRI). Weight average molecular weights (Mw) expressed with percent uncertainties for each labeled region between dashed vertical lines are listed next to each plot. Large uncertainties may be due to poor baseline selection, peak broadening, or inadequate separation medium. Results were collected in two separate batches (as indicated next to each plot) which differed in instrument setup, thus retention volumes between batches are not comparable.

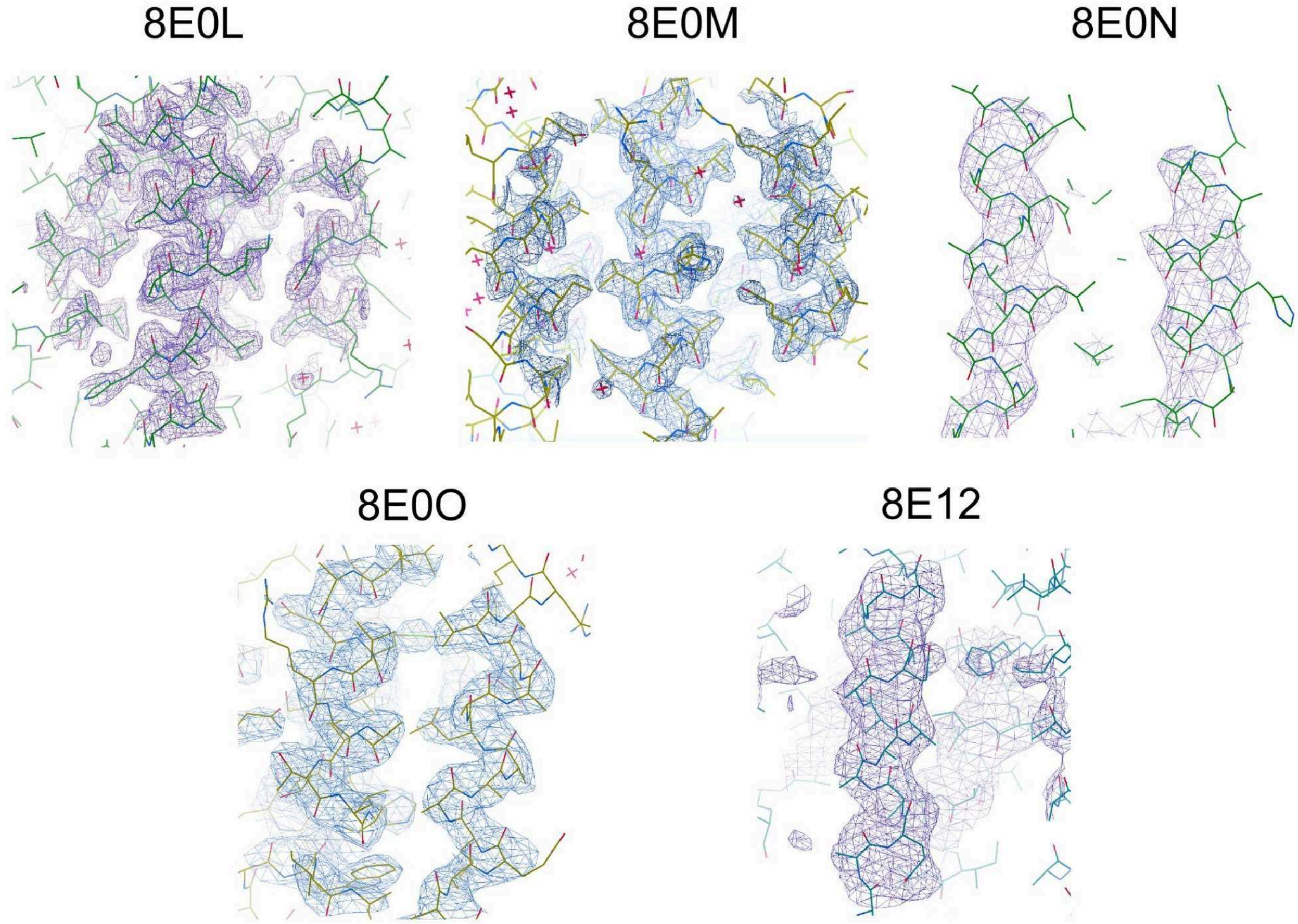

Supplementary Figure 8: Electron density maps

Images of a portion of the 2Fo-Fc map for each crystal structure contoured at 2.5 sigma.

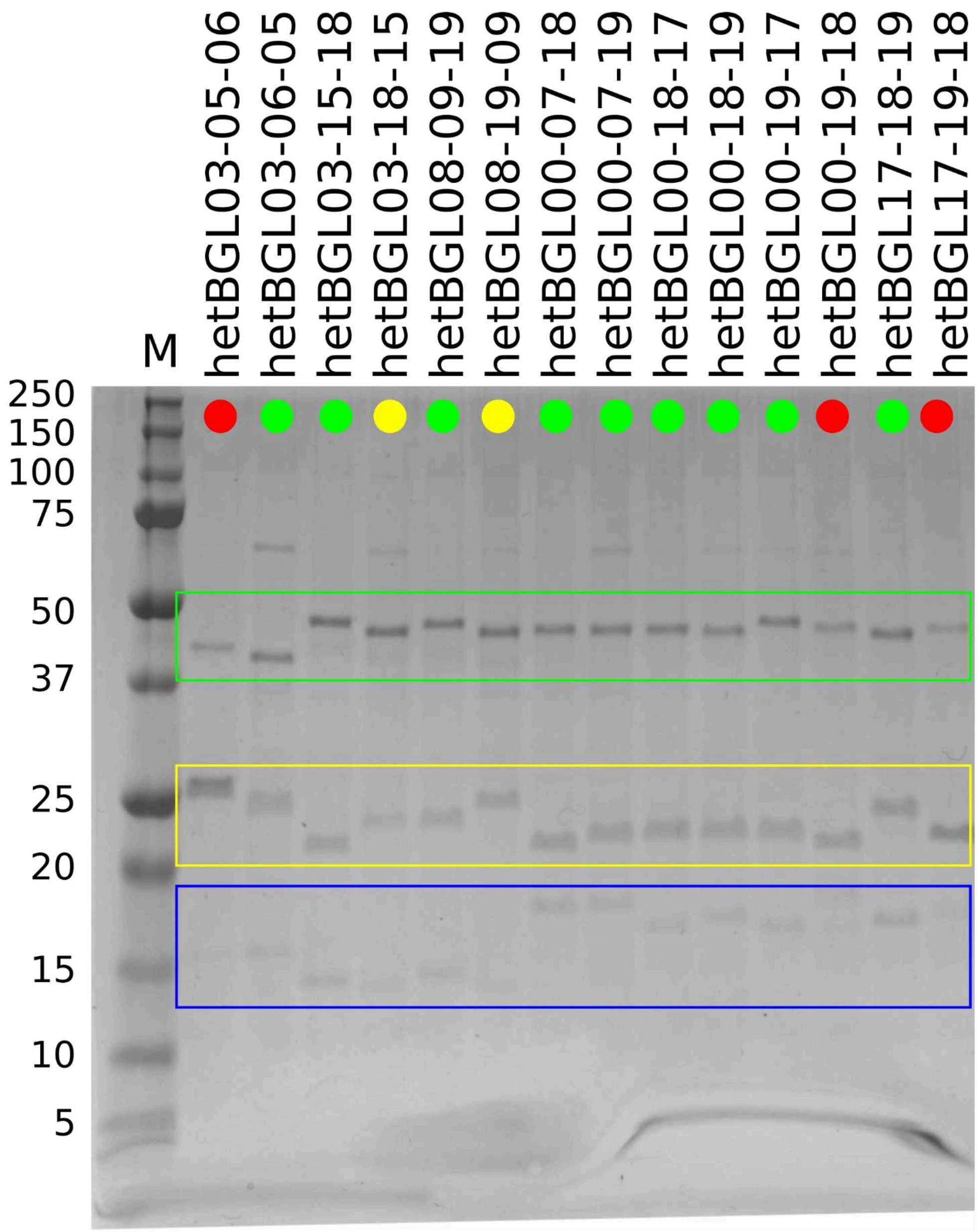

Supplementary Figure 9: SDS-PAGE of hetBGL co-purifications

IMAC co-purified co-expressions, where each construct only contains a single His6-tag. Refer to Supplementary Data 4 for His6-tag placement. The three chains are fused to different mass-adding tags to differentiate them. Green box, GFP-tagged. Yellow box, EHEE-tagged. Blue box, no mass tag. M lane is Precision Plus Protein Dual Xtra (Bio-Rad) and listed masses are in kDa. Sample lanes are annotated with dots to indicate qualitative success. Red dot, missing

chain. Yellow dot, all 3 chains present but clearly uneven stoichiometries. Green dot, all 3 chains present in approximately equal stoichiometries. Source data are provided as a Source Data file.

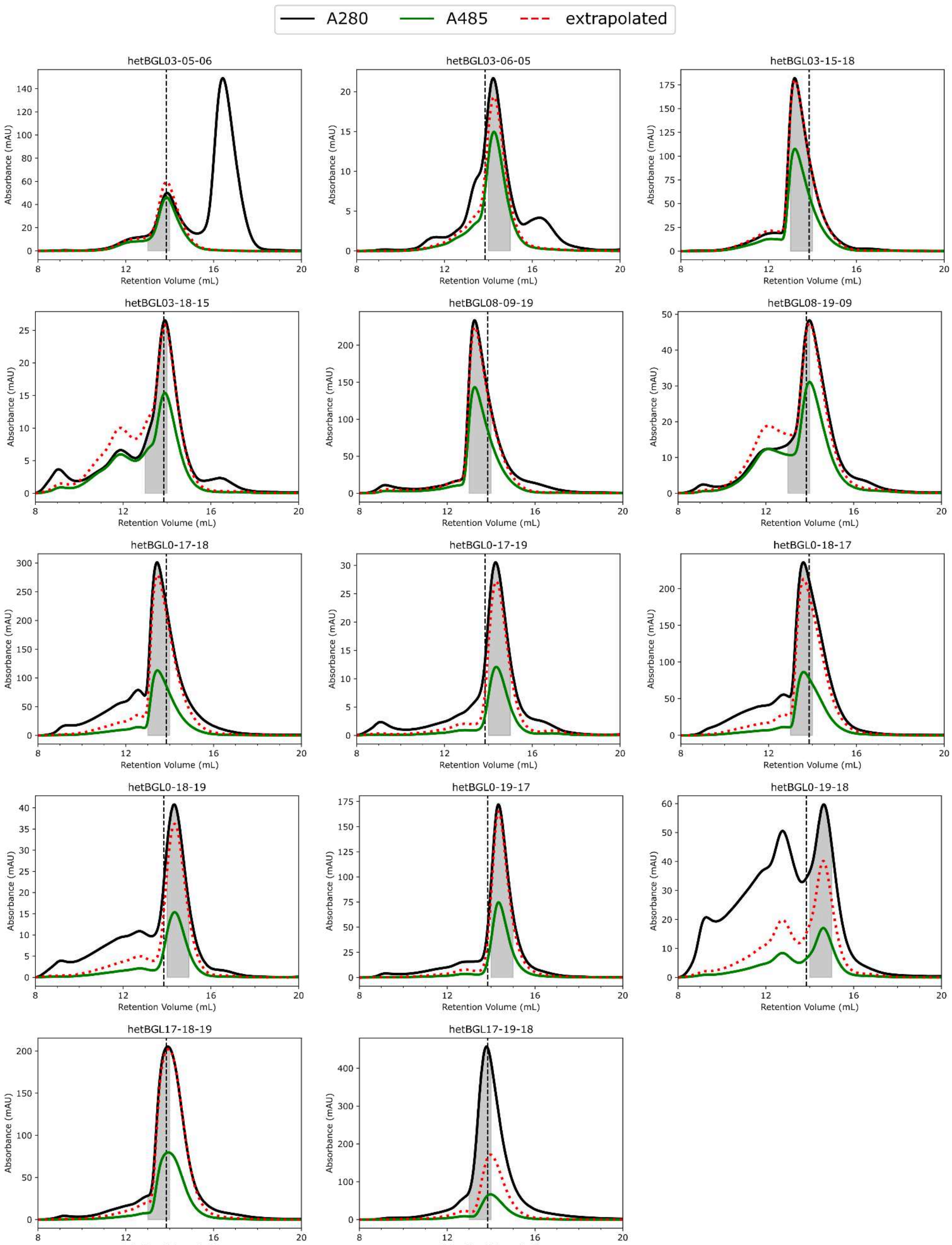
A280
A485
extrapolated
hetBGL03-05-06
hetBGL03-06-05
hetBGL03-15-18
hetBGL03-18-15
hetBGL08-09-19
hetBGL08-19-09
hetBGL0-17-18
hetBGL0-17-19
hetBGL0-18-17
hetBGL0-18-19
hetBGL0-19-17
hetBGL0-19-18
hetBGL17-18-19
hetBGL17-19-18
Absorbance (mAU)
Retention Volume (mL)

Supplementary Figure 10: SEC of Set 1 and Set 2 hetBGLs

Each successfully assembling construct will have a single GFP tag associated with it, so tracking A280 (absorbance of aromatics in every chain) and A485 (absorbance specific to the GFP chromophore) separately allows for estimation of expected signal given another signal which implies GFP:complex stoichiometry. Black, measured A280. Green, measured A485. Red dashed, expected A280 signal given the A485 signal using an estimated EC485 of 36650 $M^{-1}cm^{-1}$ and estimated A280 specific to each construct. Shaded region, fraction used in downstream analysis.

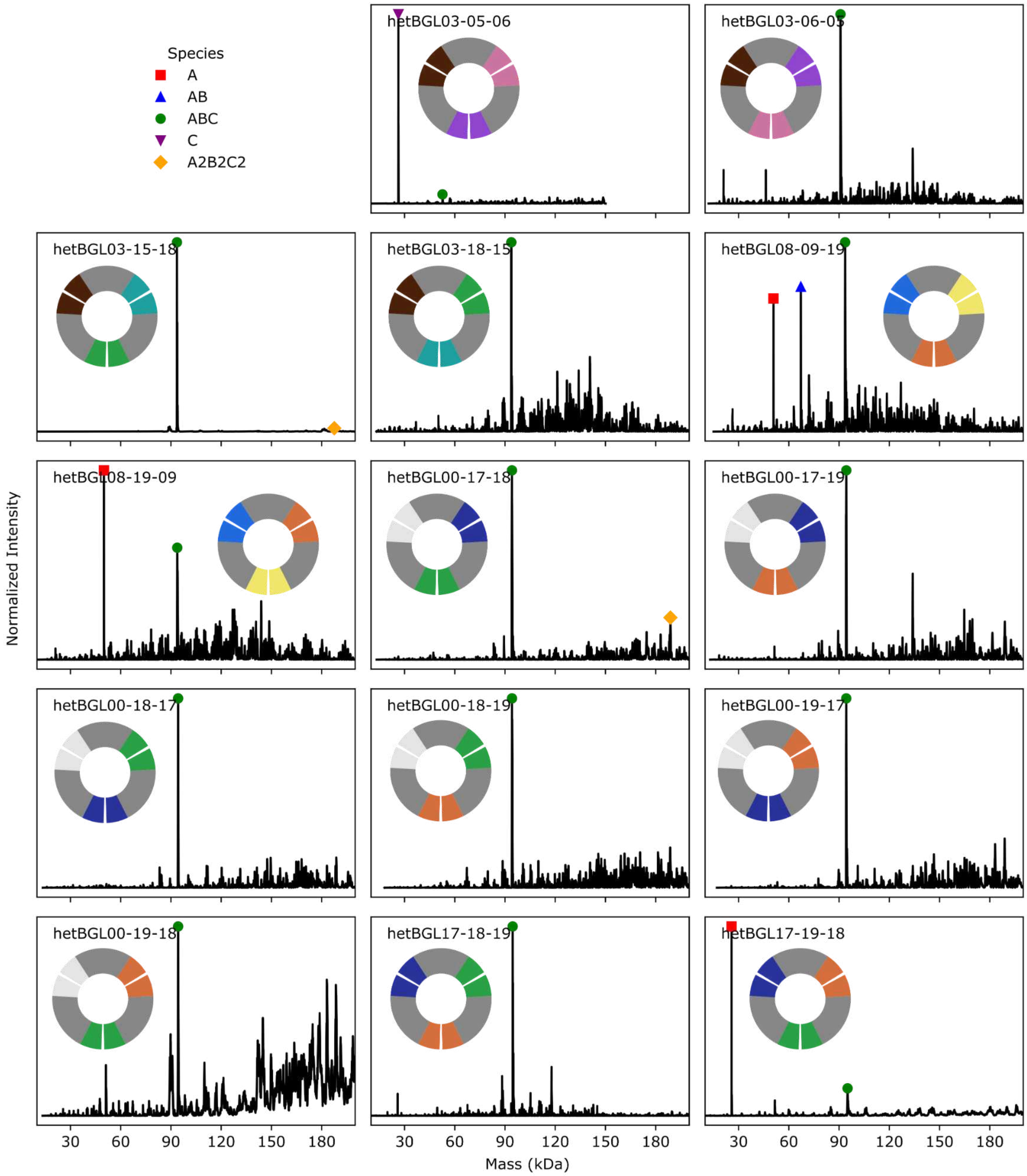

Supplementary Figure 11: nMS of hetBGLs

Black, deconvoluted masses obtained from m/z spectra (Supplementary Data 13). Identified species corresponding to chain A (red square), A+B (blue triangle), A+B+C (green circle), C (purple upside-down triangle), and (A+B+C)x2 (yellow diamond) are annotated; other un-annotated peaks are unidentified species.

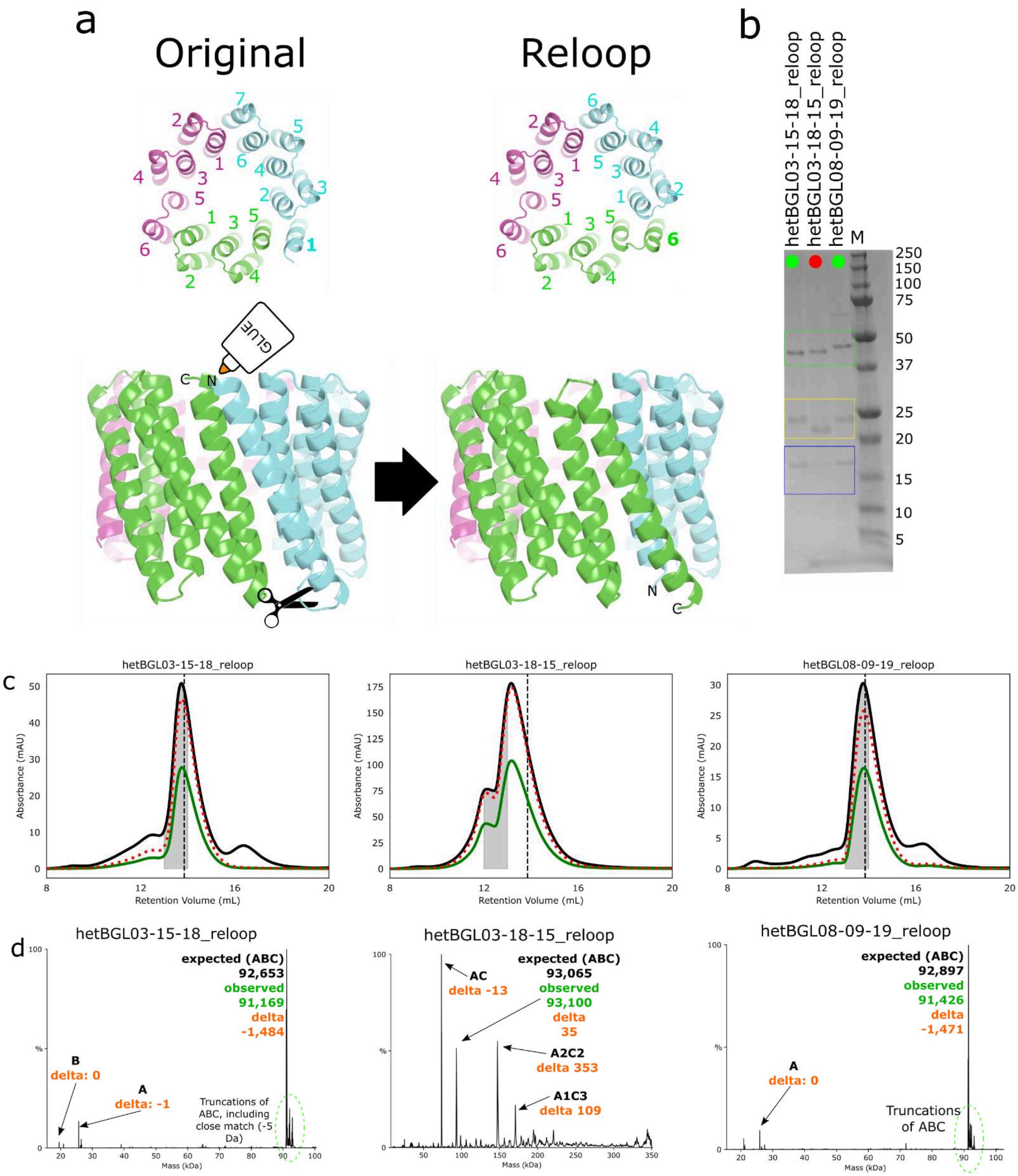

Supplementary Figure 12: Circular permutation of Set 1 hetBGLs

(a) Set 1 hetBGLs ("Original") have different numbers of helices in each chain. To convert them into structures more resembling those of Set 2 which have six helices per chain, the loop between the first and second helix of the longest chain (blue, left) was cut and attached to the

C-terminus of the shortest chain (green) using the same loop design procedure used to make the HR-C homotrimers. The result is the heterotrimer on the right with six helices per chain and where all chains have the same topology. (b) SDS-PAGE of IMAC co-purified co-expressions of relooped hetBGLs, where each construct only contains a single His6-tag. Refer to Supplementary Data 4 for His6-tag placement. The three chains are fused to different mass-adding tags to differentiate them. Green box, GFP-tagged. Yellow box, EHEE-tagged. Blue box, no mass tag. M lanes are Precision Plus Protein Dual Xtra (Bio-Rad) and listed masses are in kDa. Sample lanes are annotated with dots to indicate qualitative success. Red dot, missing chain. Yellow dot, all 3 chains present but uneven stoichiometries. Green dot, all 3 chains present in approximately equal stoichiometries. Source data are provided as a Source Data file. (c) SEC traces. Black, measured A280. Green, measured A485. Red dashed, expected A280 signal given the A485 signal using an estimated EC485 of 36650 $M^{-1}cm^{-1}$ and estimated A280 specific to each construct. Shaded region indicates the fraction used for downstream analysis. (d) Black, deconvoluted masses obtained from m/z spectra (Supplementary Data 13). Annotated peaks are indicated with arrows with masses listed in the top left corner of each plot.

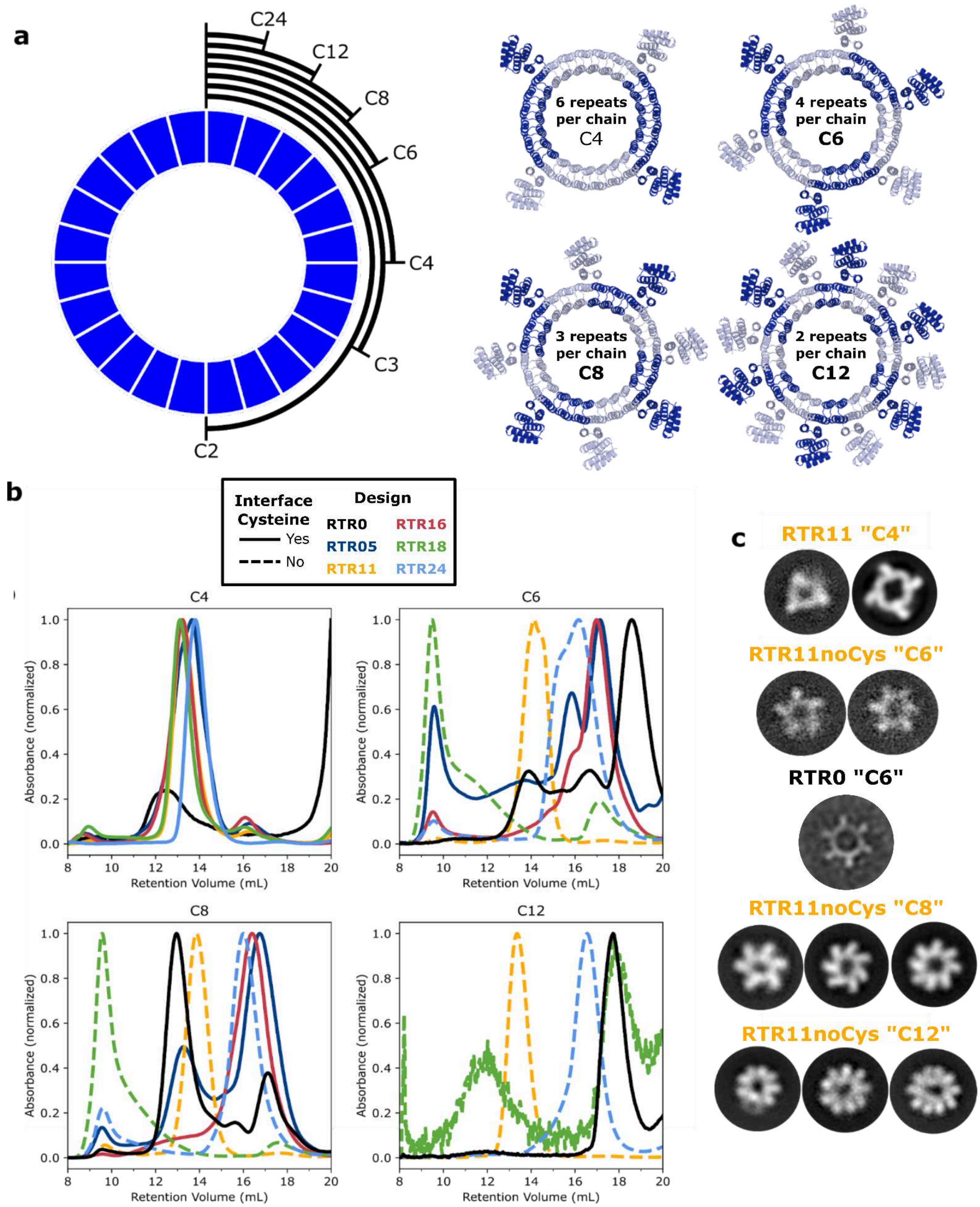

a
C24
C12
C8
C6
C4
C3
C2
6 repeats per chain C4
4 repeats per chain C6
3 repeats per chain C8
2 repeats per chain C12
b
Interface Cysteine
Yes
No
Design
RTR0
RTR16
RTR05
RTR18
RTR11
RTR24
C4
C6
C8
C12
Absorbance (normalized)
Retention Volume (mL)
c
RTR11 "C4"
RTR11noCys "C6"
RTR0 "C6"
RTR11noCys "C8"
RTR11noCys "C12"

Supplementary Figure 13: Variable oligomeric state of RTR homo-oligomers

(a, left) Schematic representation of how a cyclic (24x) repeat protein (toroid) can be divided into different homo-oligomers which assemble into symmetries depending on the number of repeats combined into a single chain. The final assemblies all have the same number of total repeats. For example, a C4 assembly is made from a single chain containing 6 repeats. (right) Cartoon models of C4, C6, C8, and C12 versions of the RTR backbone, constructed by removing repeats distant from the interface repeats. (b) SEC traces of RTR variants with different numbers of repeats per chain. Solid traces, interfaces with cysteines. Dashed traces, interfaces without cysteines. Black, RTR0. Dark blue, RTR05. Yellow, RTR11. Red, RTR16. Green, RTR18. Light blue, RTR24. (c) Negative stain EM 2D class averages of RTR0 or RTR11 with different numbers of repeats per chain. Each class represents a different observed oligomeric state.

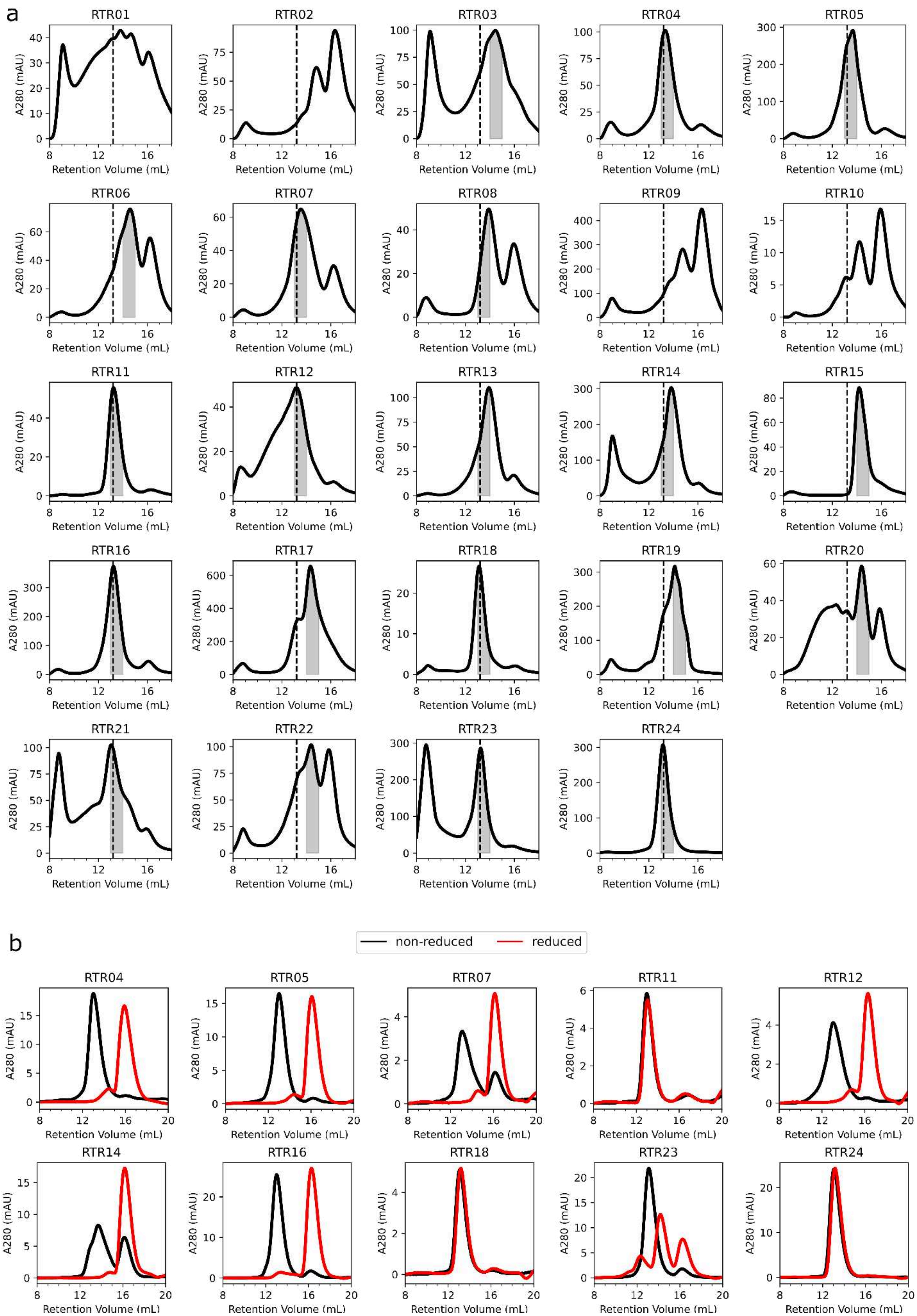
a
RTR01
RTR02
RTR03
RTR04
RTR05
RTR06
RTR07
RTR08
RTR09
RTR10
RTR11
RTR12
RTR13
RTR14
RTR15
RTR16
RTR17
RTR18
RTR19
RTR20
RTR21
RTR22
RTR23
RTR24
A280 (mAU)
Retention Volume (mL)
b
non-reduced
reduced
RTR04
RTR05
RTR07
RTR11
RTR12
RTR14
RTR16
RTR18
RTR23
RTR24
A280 (mAU)
Retention Volume (mL)

Supplementary Figure 14: SEC of RTR homo-tetramers

SEC traces of (a) initial RTR samples on S200 column and (b) reran isolated fractions after being reduced with 10mM TCEP or not. Shaded regions, fractions used in downstream analysis. Dashed line, expected retention volume.

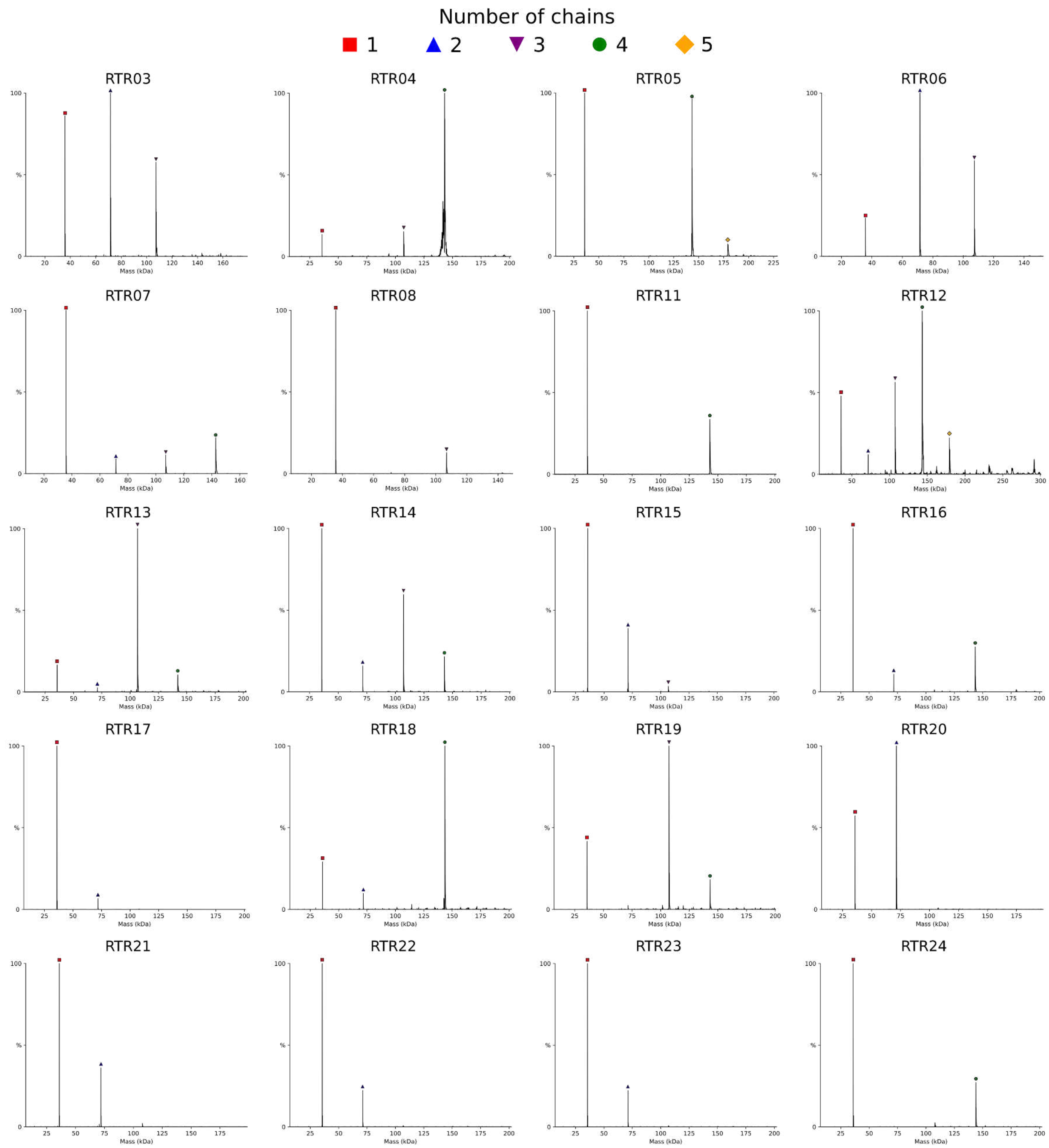

Supplementary Figure 15: nMS of RTR homo-tetramers

Plots of all designs with nMS data. Black, deconvoluted masses obtained from m/z spectra (Supplementary Data 13). Identified species corresponding to monomer (red square), dimer

(blue triangle), trimer (purple upside-down triangle), tetramer (green circle), and hexamer (yellow diamond) are annotated; other un-annotated peaks are unidentified species.

RTR0 (C4)

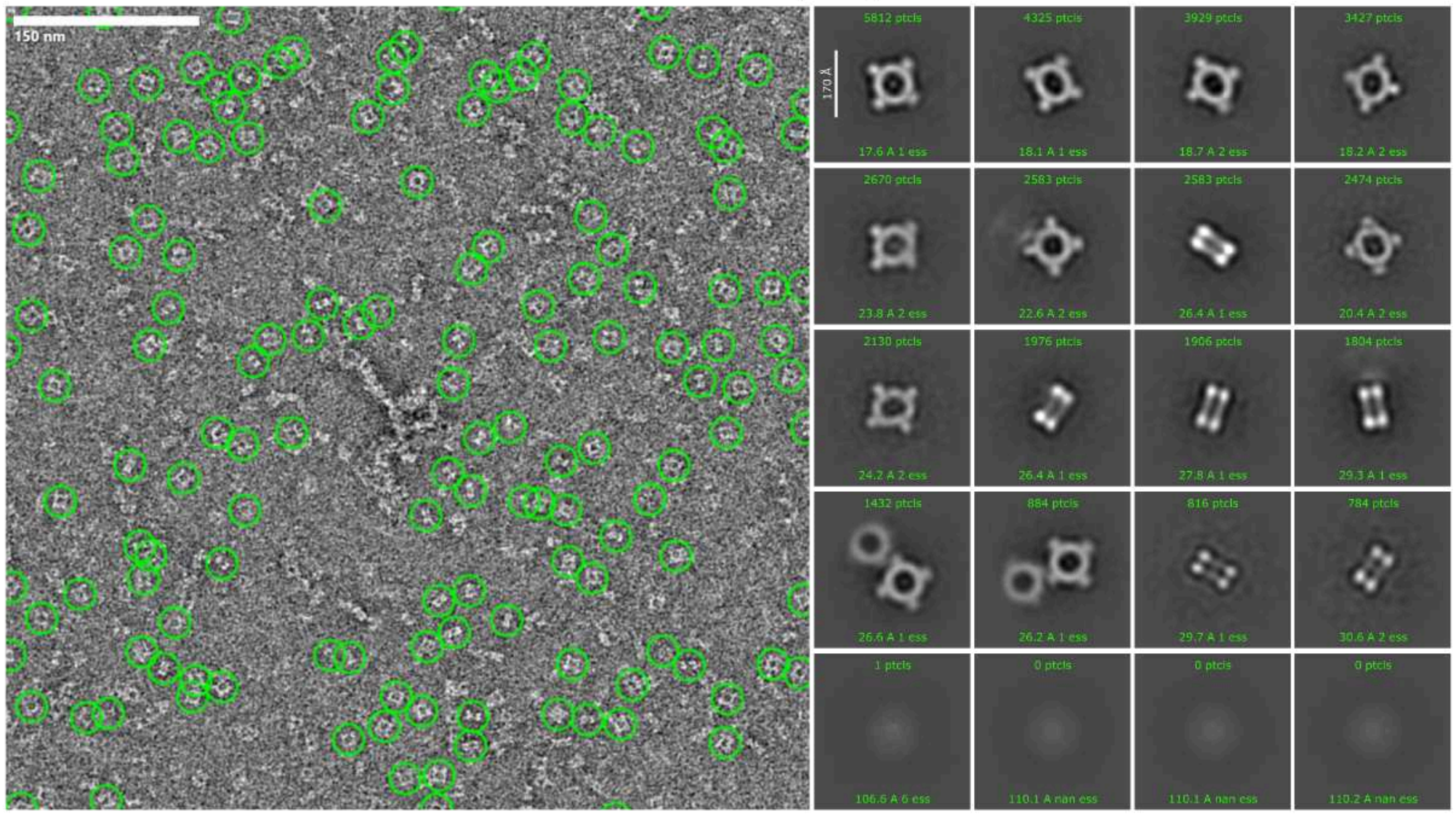

Rigid body fit into map from
non-uniform refinement

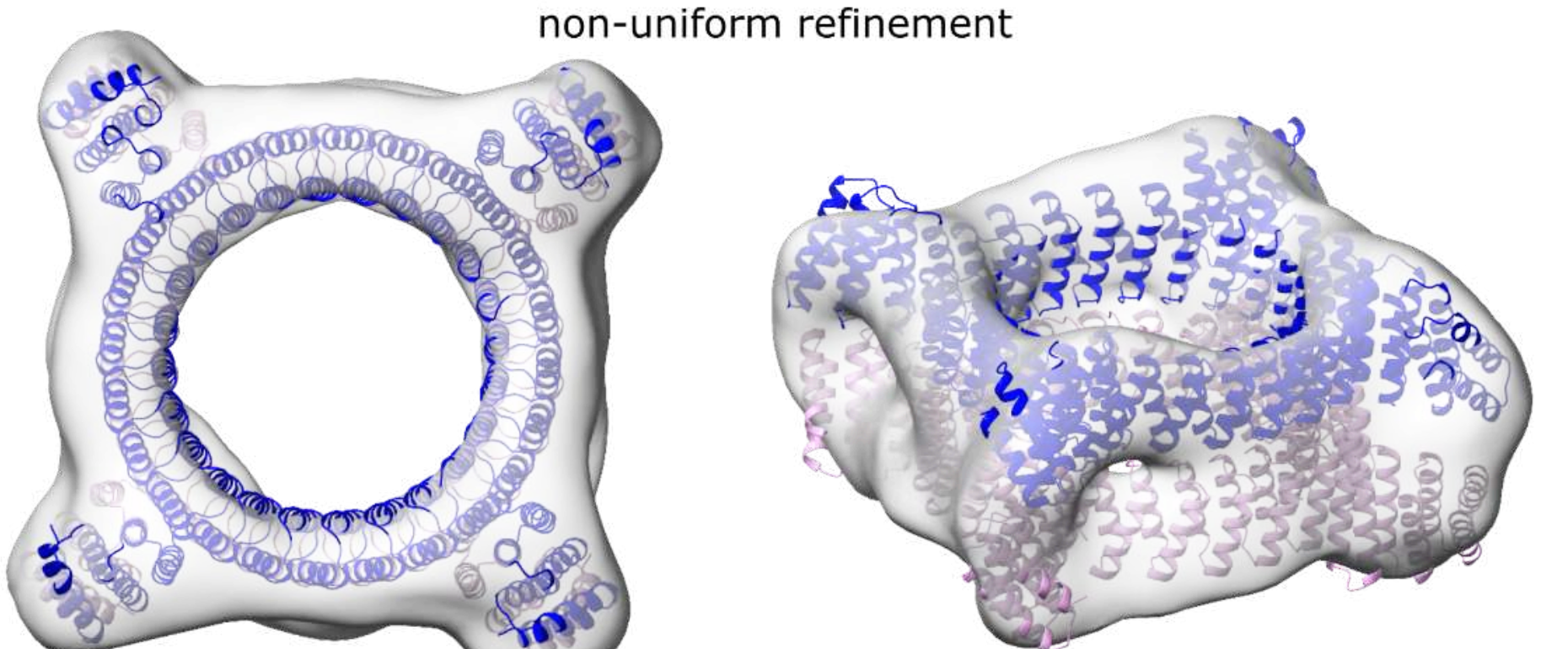

RTR0 (C6)

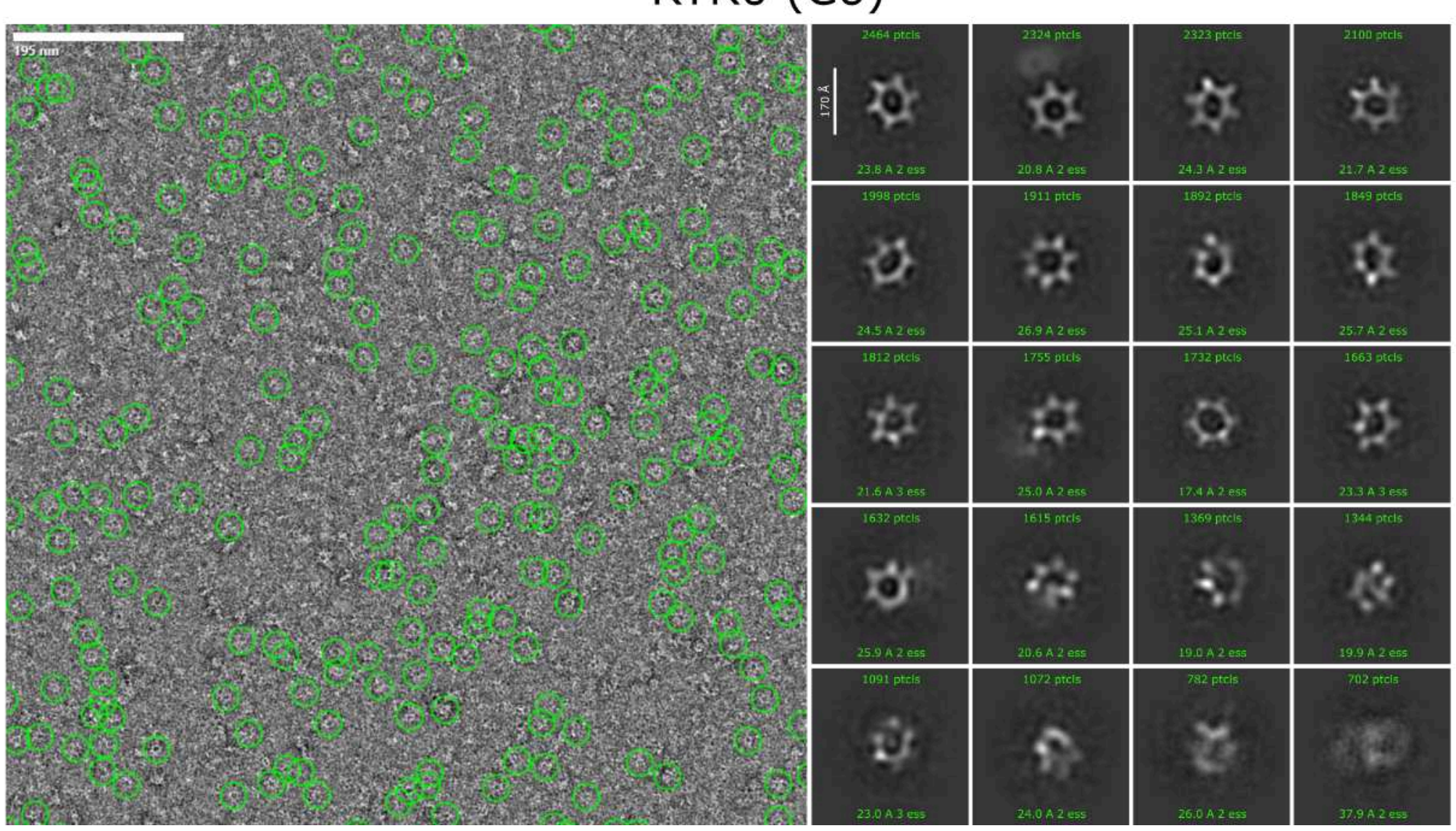

RTR05 (C4)

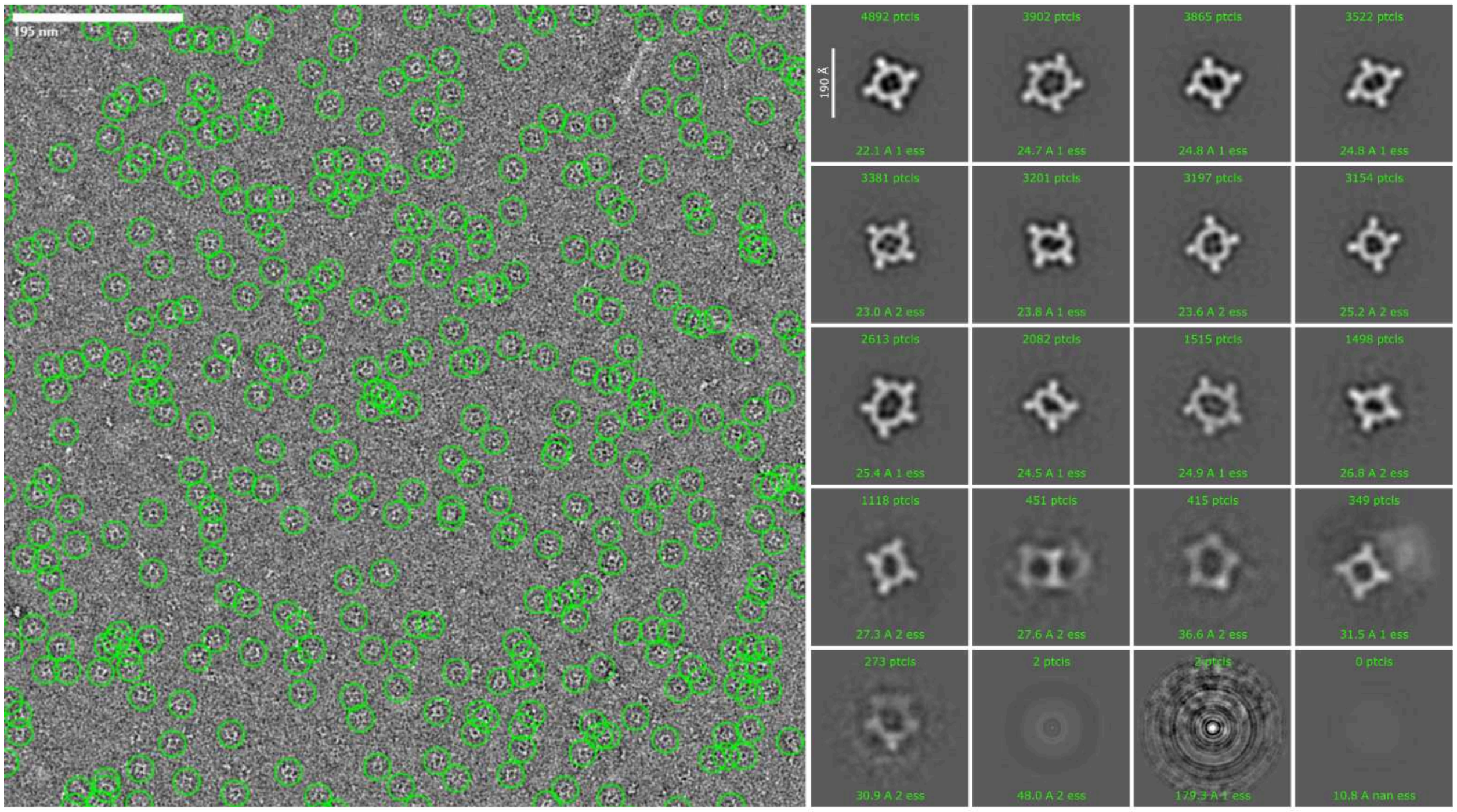

RTR11 (C4)

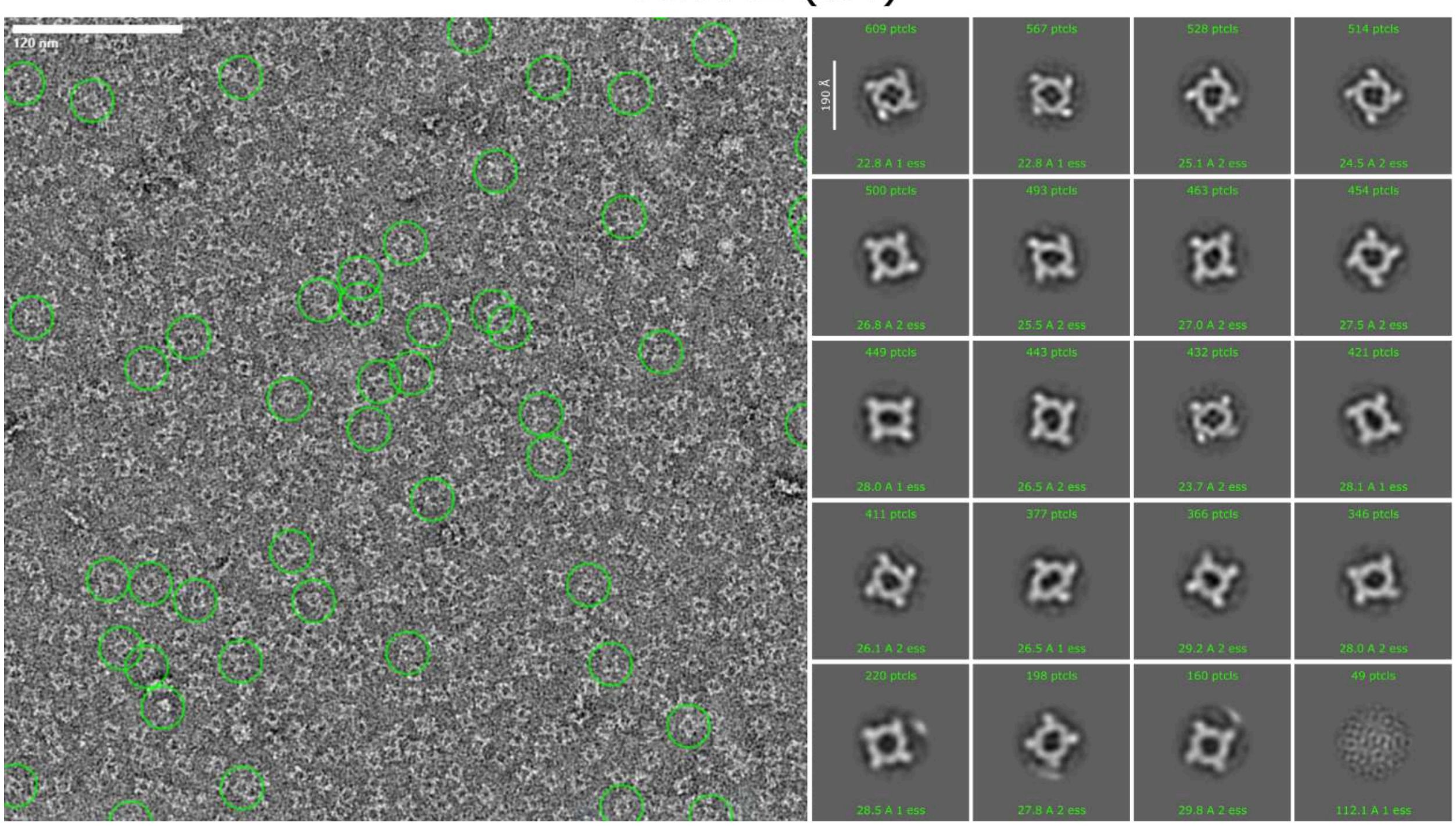

RTR11_noCys (C6)

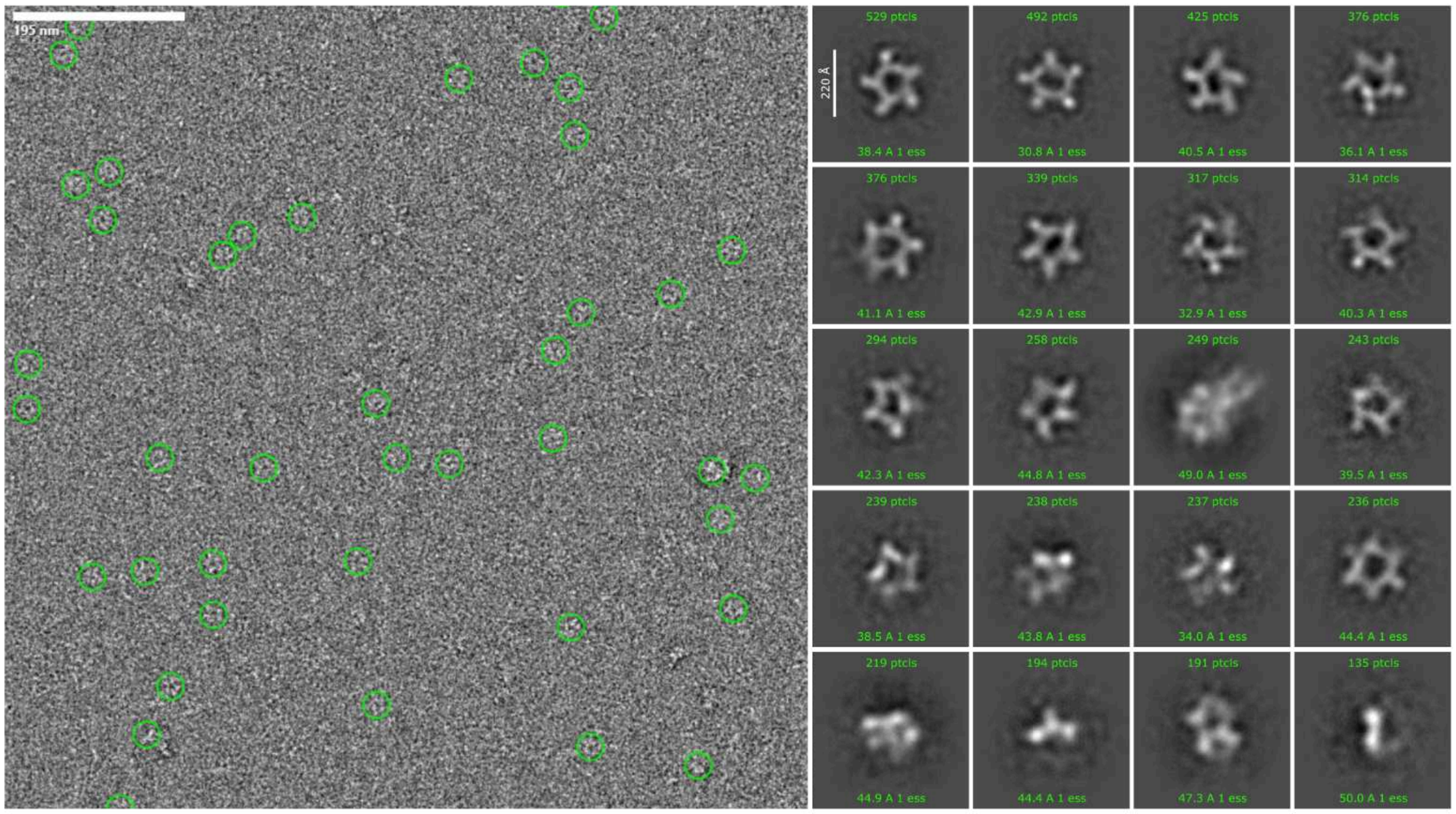

RTR11_noCys_1d6b5438aa0d917bea2962dcb63062e6_0001_1 (C6)

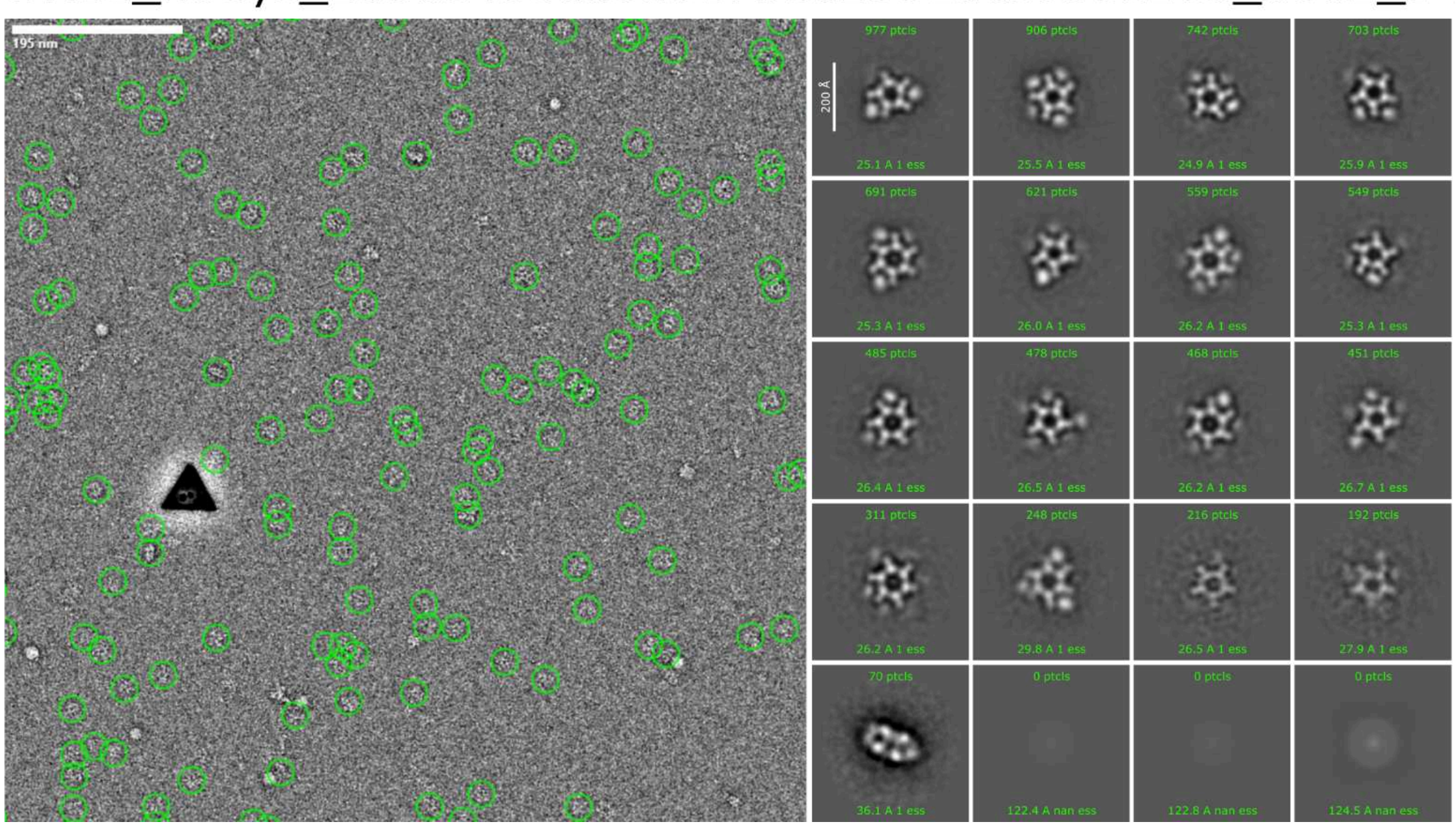

RTR11_noCys (C8)

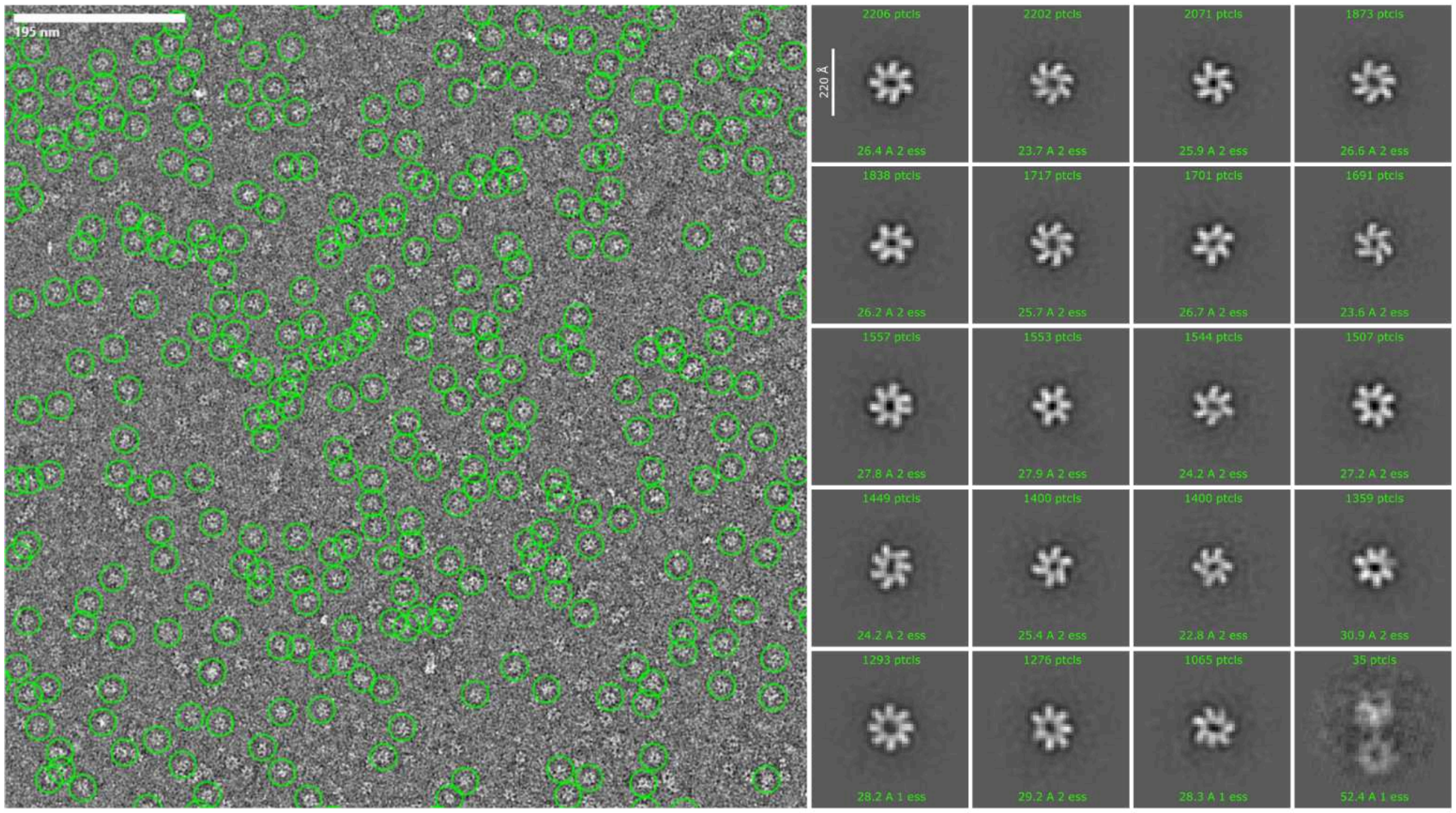

RTR11_noCys (C12)

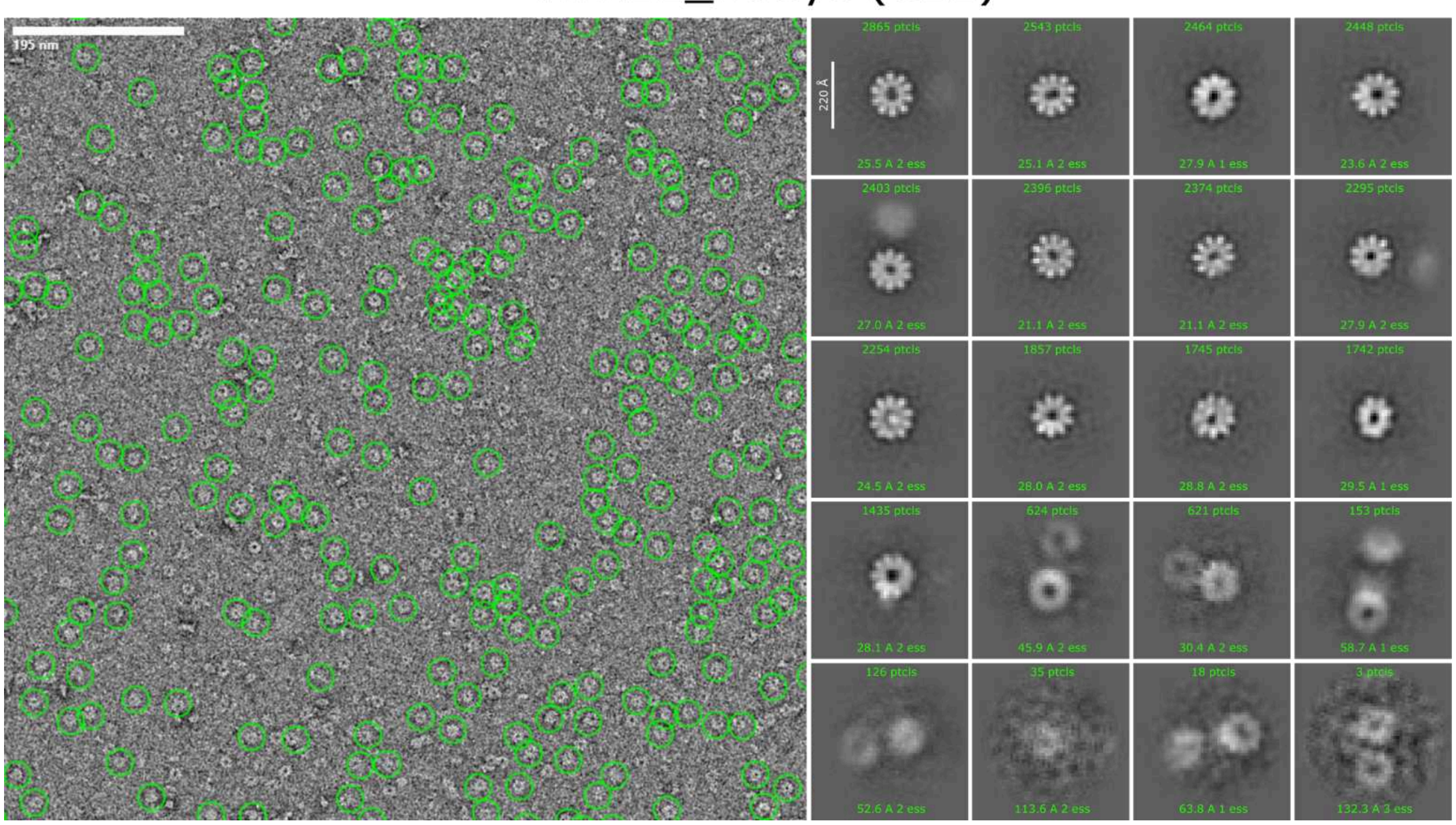

RTR16 (C4)

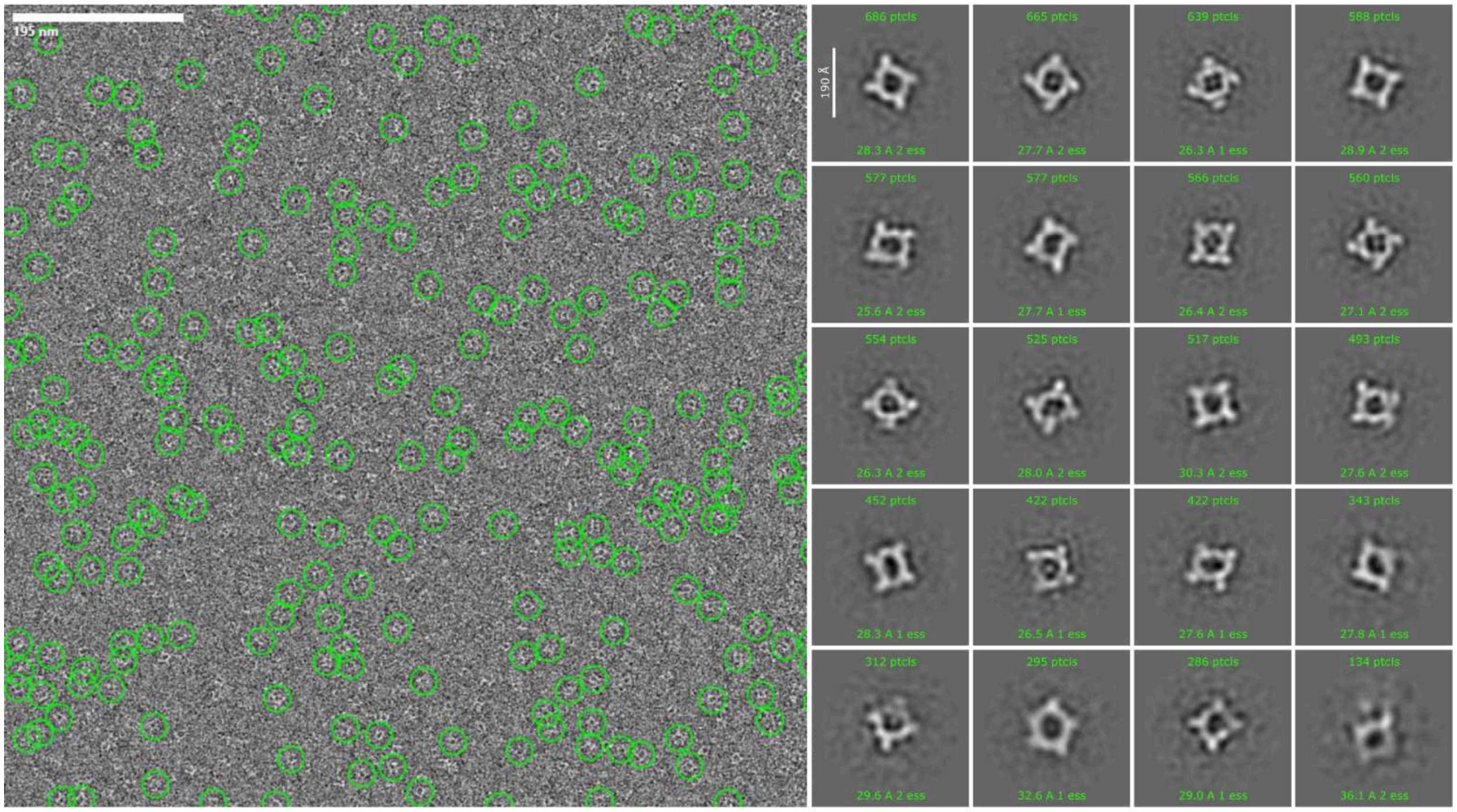

RTR24 (C4)

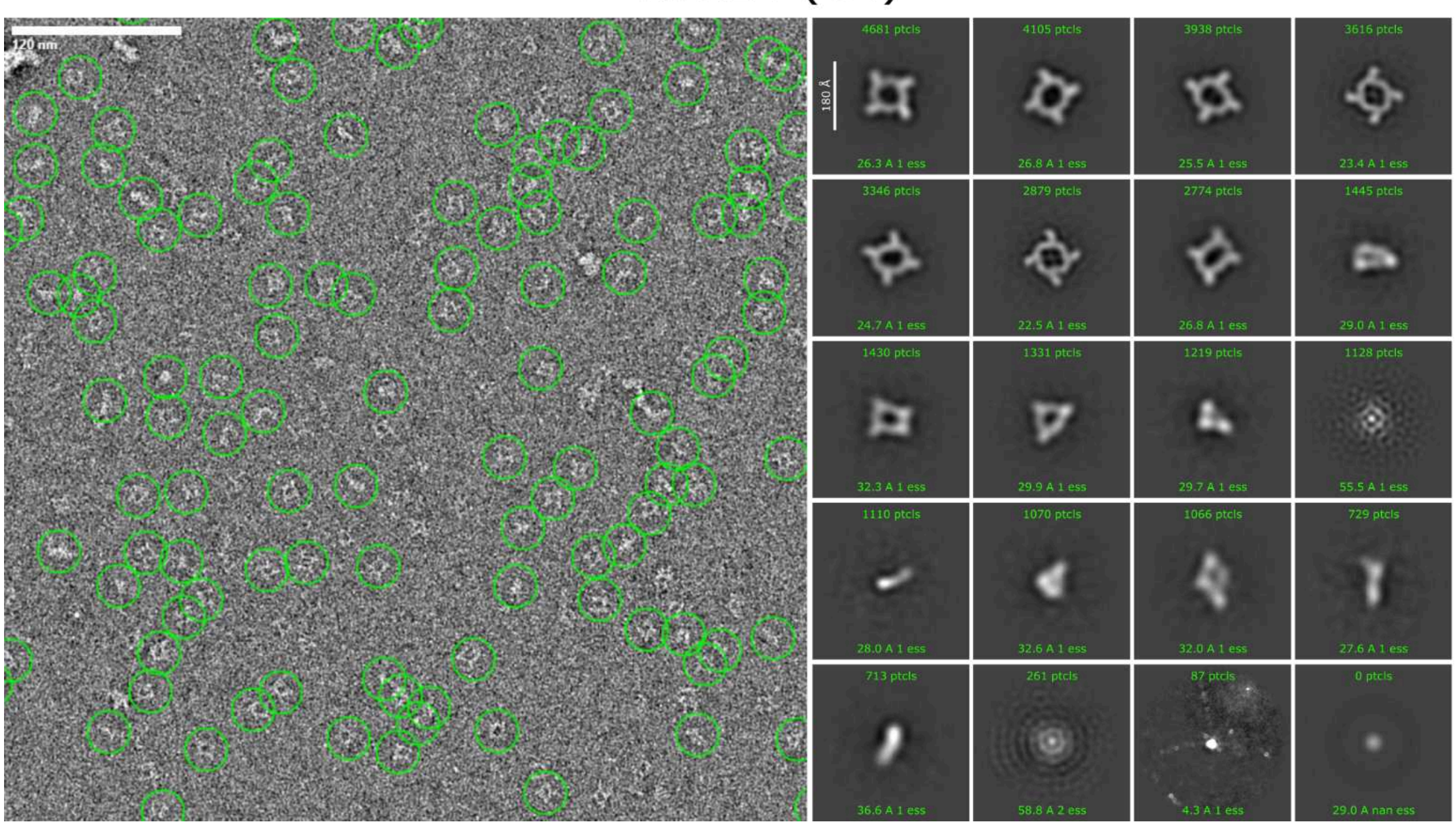

RTR24_DHR52 (C4)

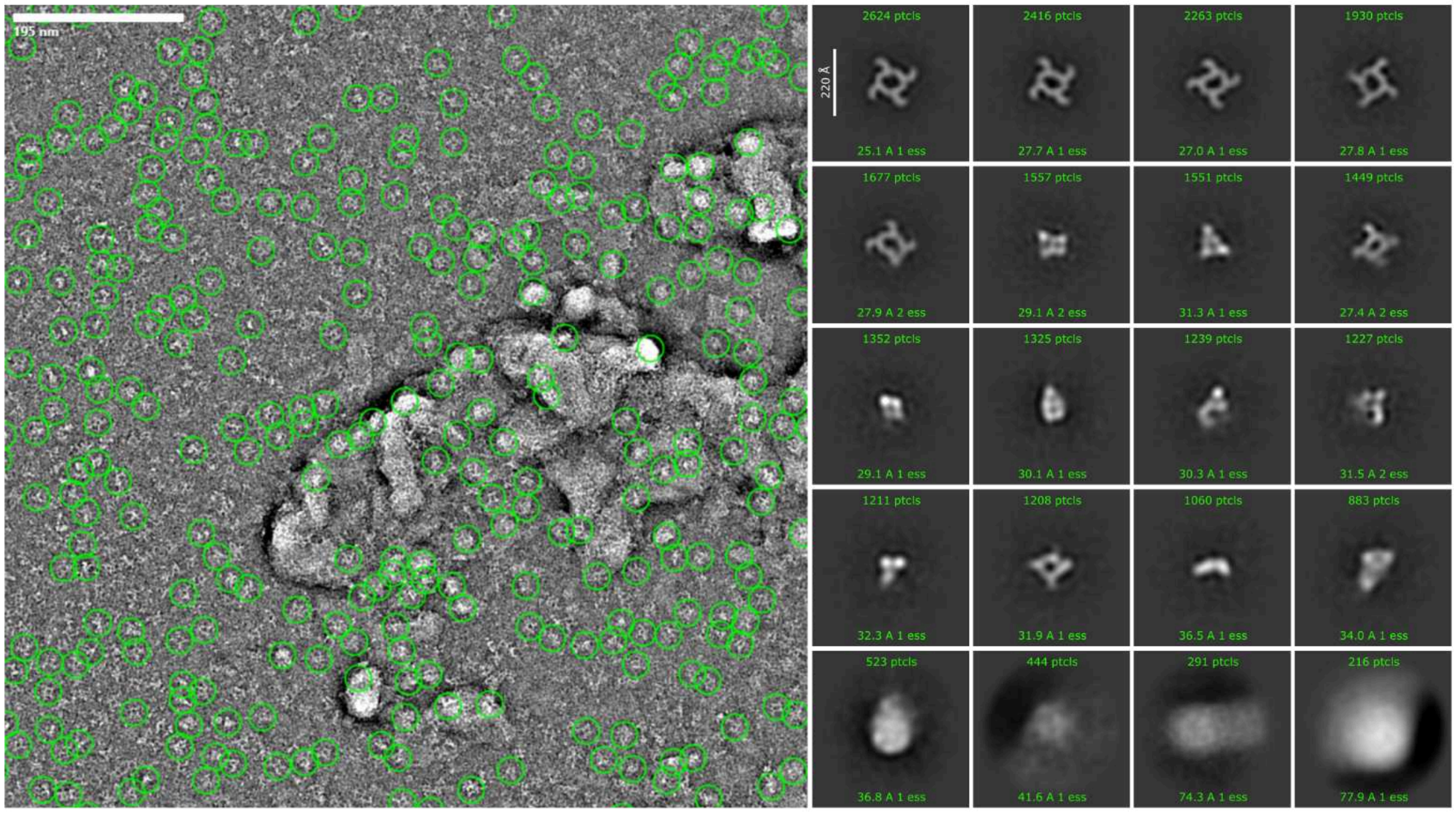

RTR24_DHR62 (C4)

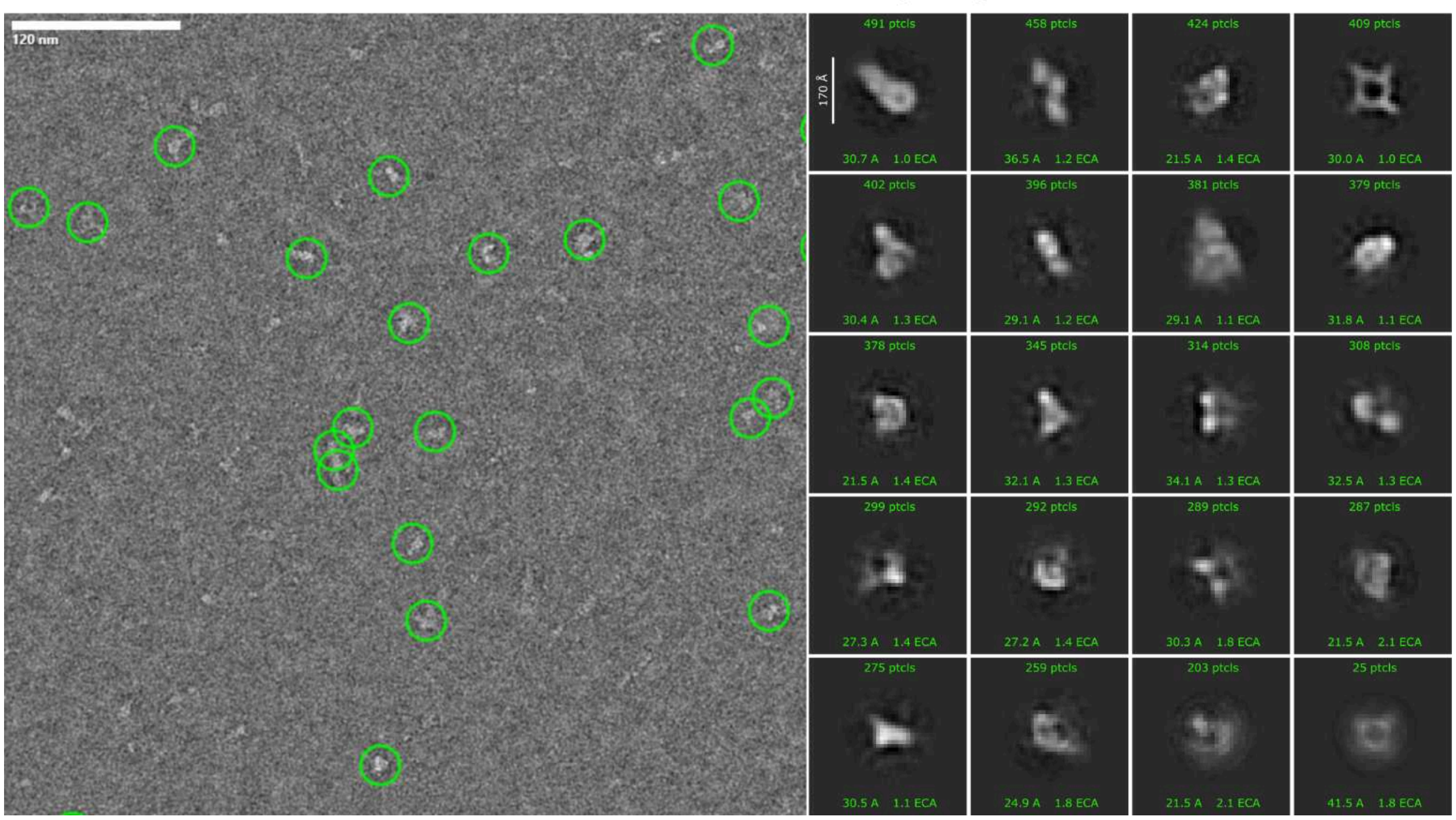

RTR24_DHR70_1 (C4)

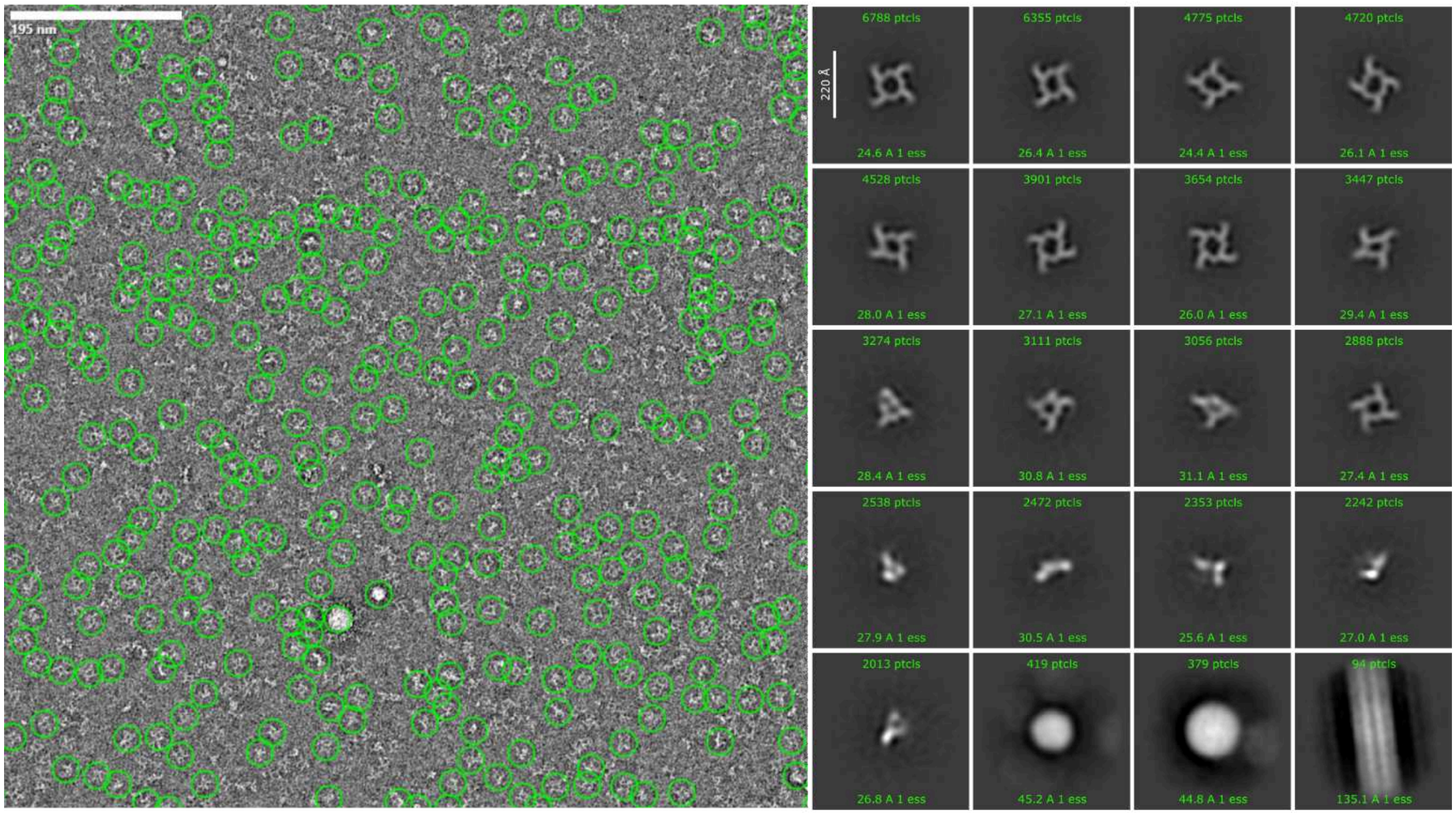

RTR24_DHR70_2 (C4)

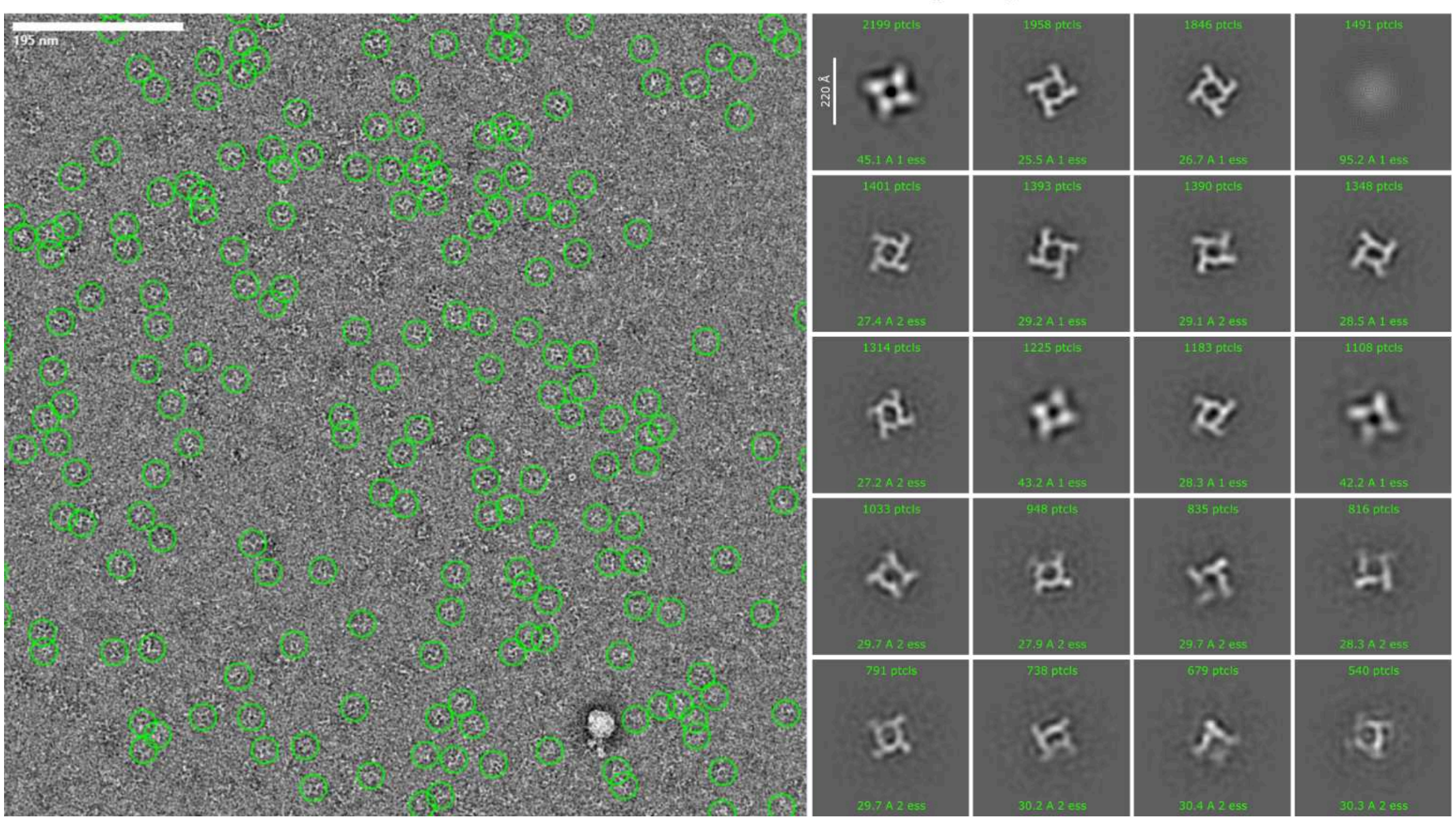

RTR24_DHR80_1 (C4)

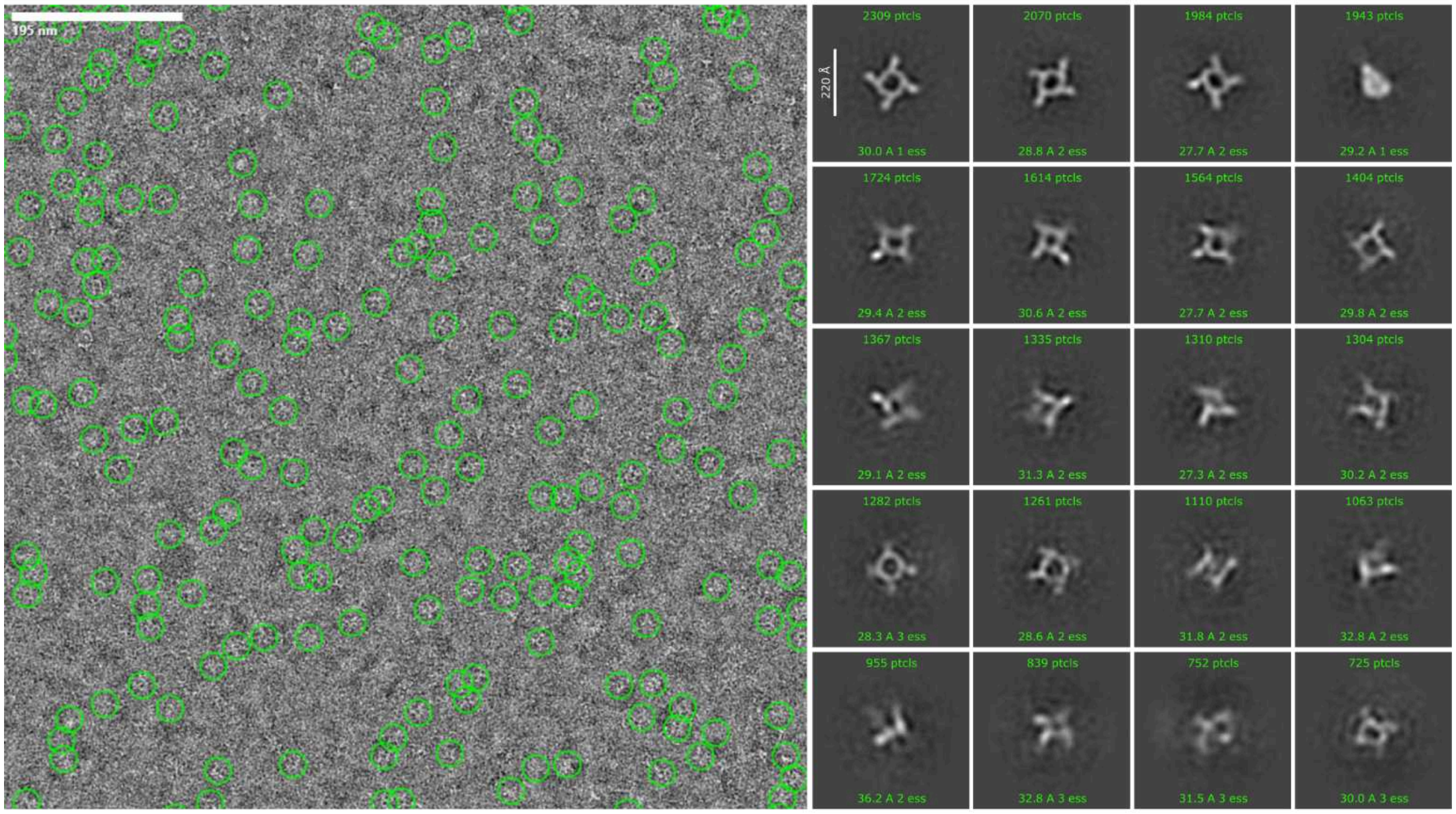

RTR24_DHR81 (C4)

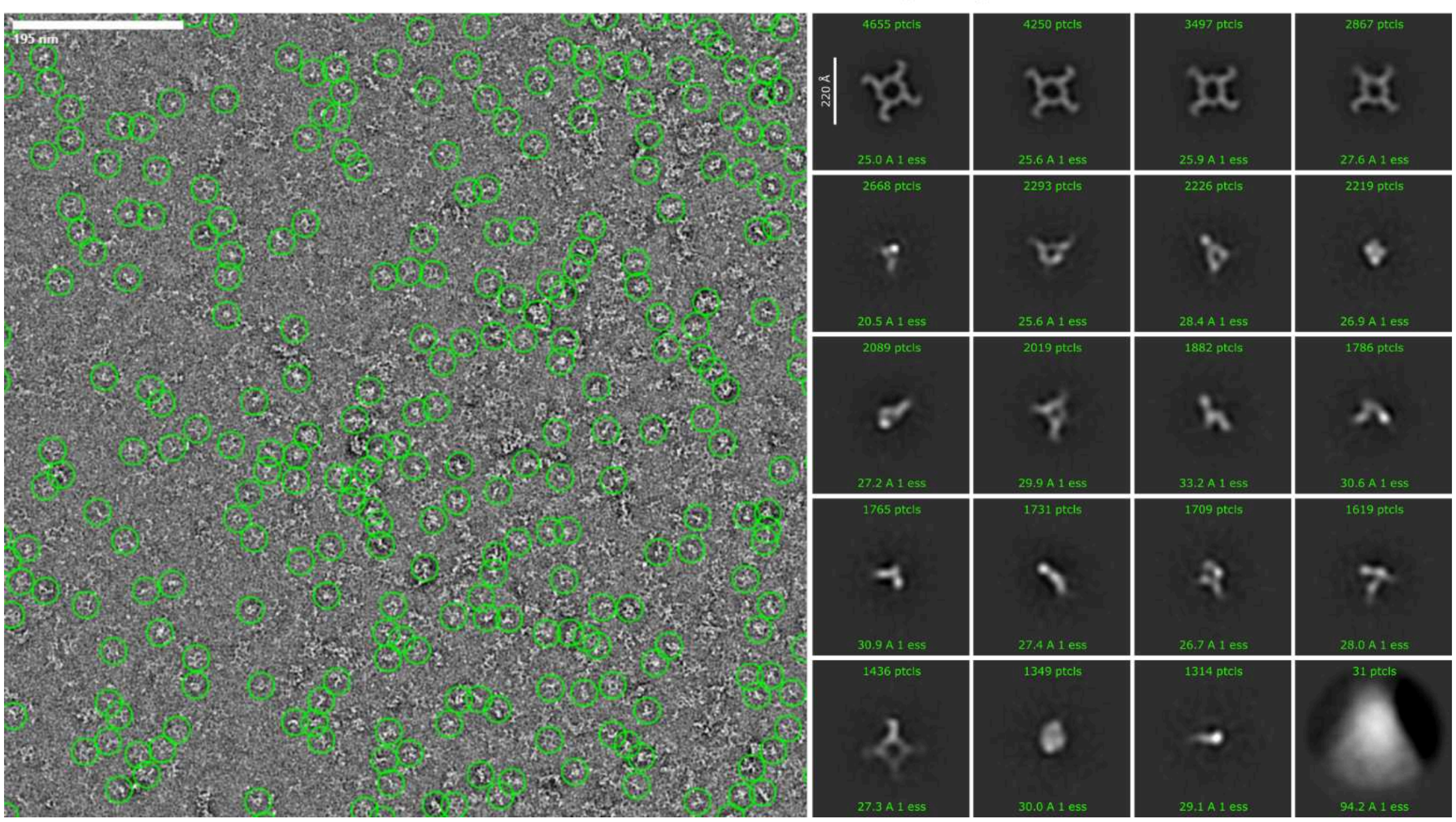

Supplementary Figure 16: nsEM micrographs and 2D class averages of RTR homo-oligomers
For each design, a representative micrograph (left) with circled particles included in the dataset used to construct 2D class averages (right). Scale is indicated by labeled scale bars on the micrograph and the first class. RTR0 (C4) shows a map from a homogeneous refinement with C4 symmetry with a rigidbody fit of two copies of the design model, implying dimerization.

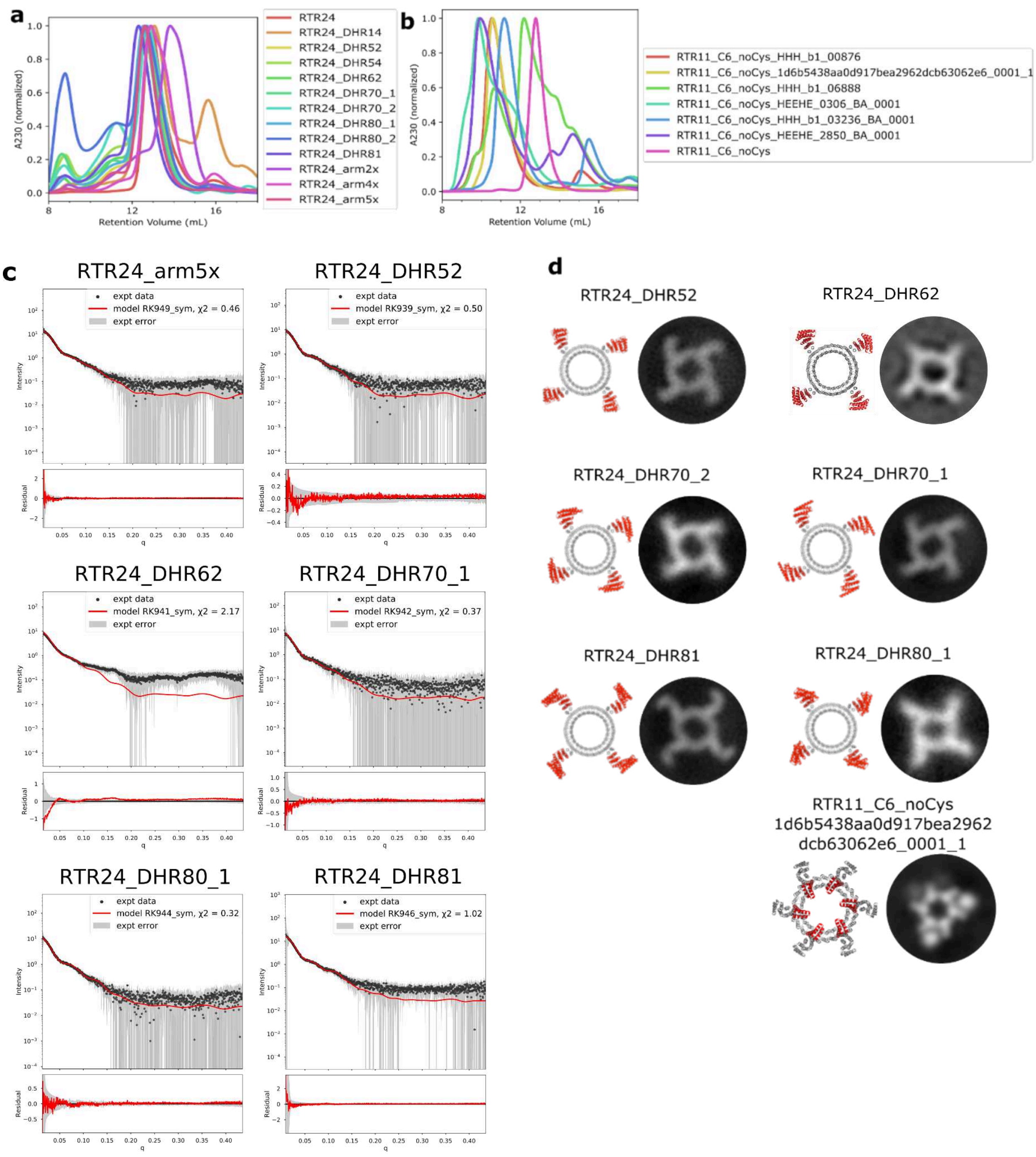

Supplementary Figure 17: Characterization of RTR extensions

(a) SEC traces of RTR24 with c-terminal extensions. RTR24 included as a size reference. (b) SEC traces of RTR11_C6 with n-terminal extensions and flexible GFP fusions. RTR11_C6 without the GFP fusion included as a rough size reference. (c) SAXS profiles of six c-terminally extended designs. Black, experimental data. Red, theoretical profile based on design model fit to experimental data. Gray shading, standard deviation of averaged micrographs. (d) Negative stain

EM 2D class averages of six c-terminally and one n-terminally extended RTR variants paired with cartoon models with the extended region shown in red. RTR11_C6_noCys_1d6b5438aa0d917bea2962dcb63062e6_0001_1 was tagged with GFP, which appears as ordered density between the outer arms.

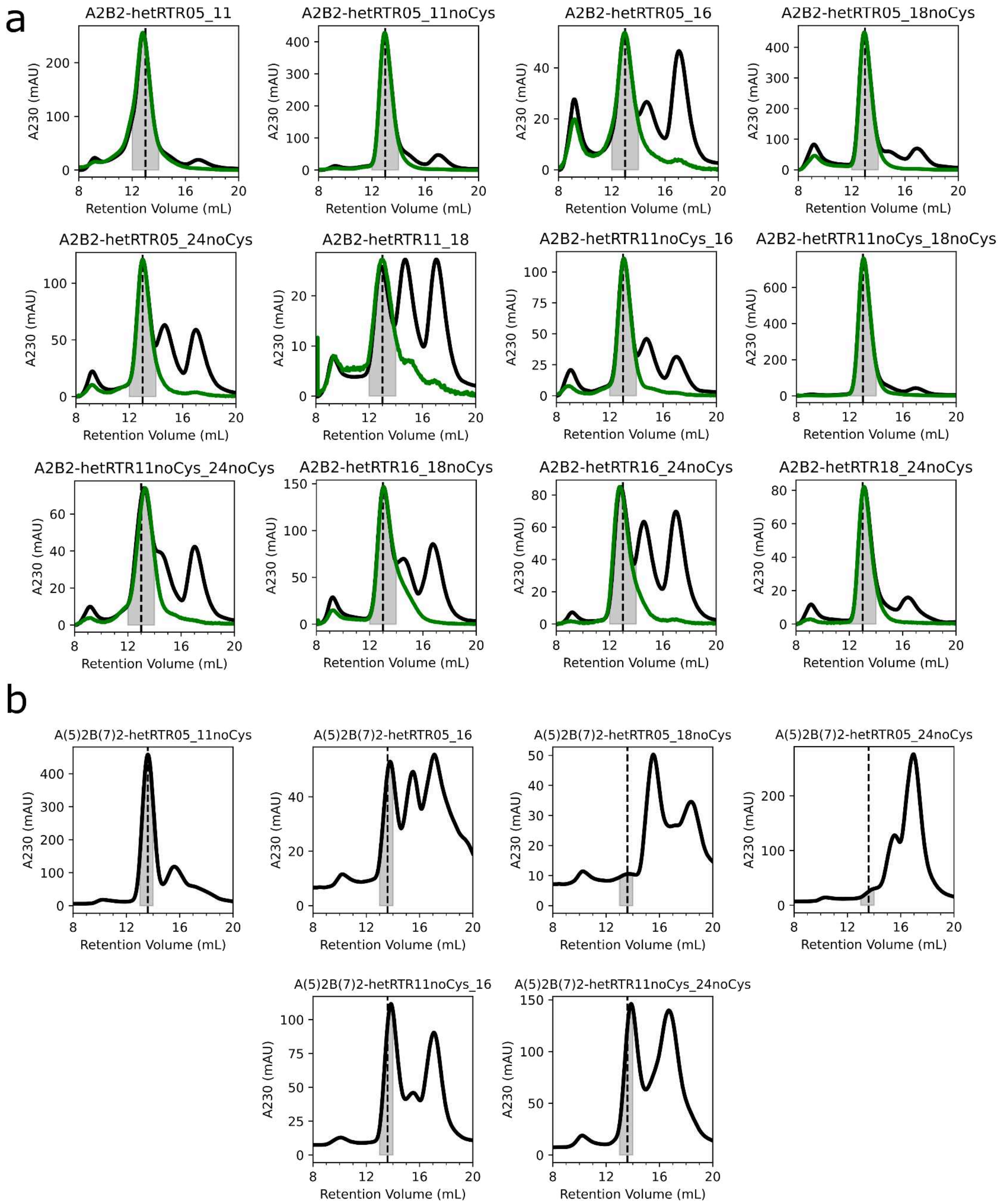

Supplementary Figure 18: SEC of A2B2-hetRTRs

(a) Black, normalized absorbance at 230nm. Green, normalized absorbance at 480nm, corresponding to the GFP-tagged chain. Shaded area, fraction used for downstream analysis. Dashed line, expected retention volume. (b) Normalized absorbance at 230nm.

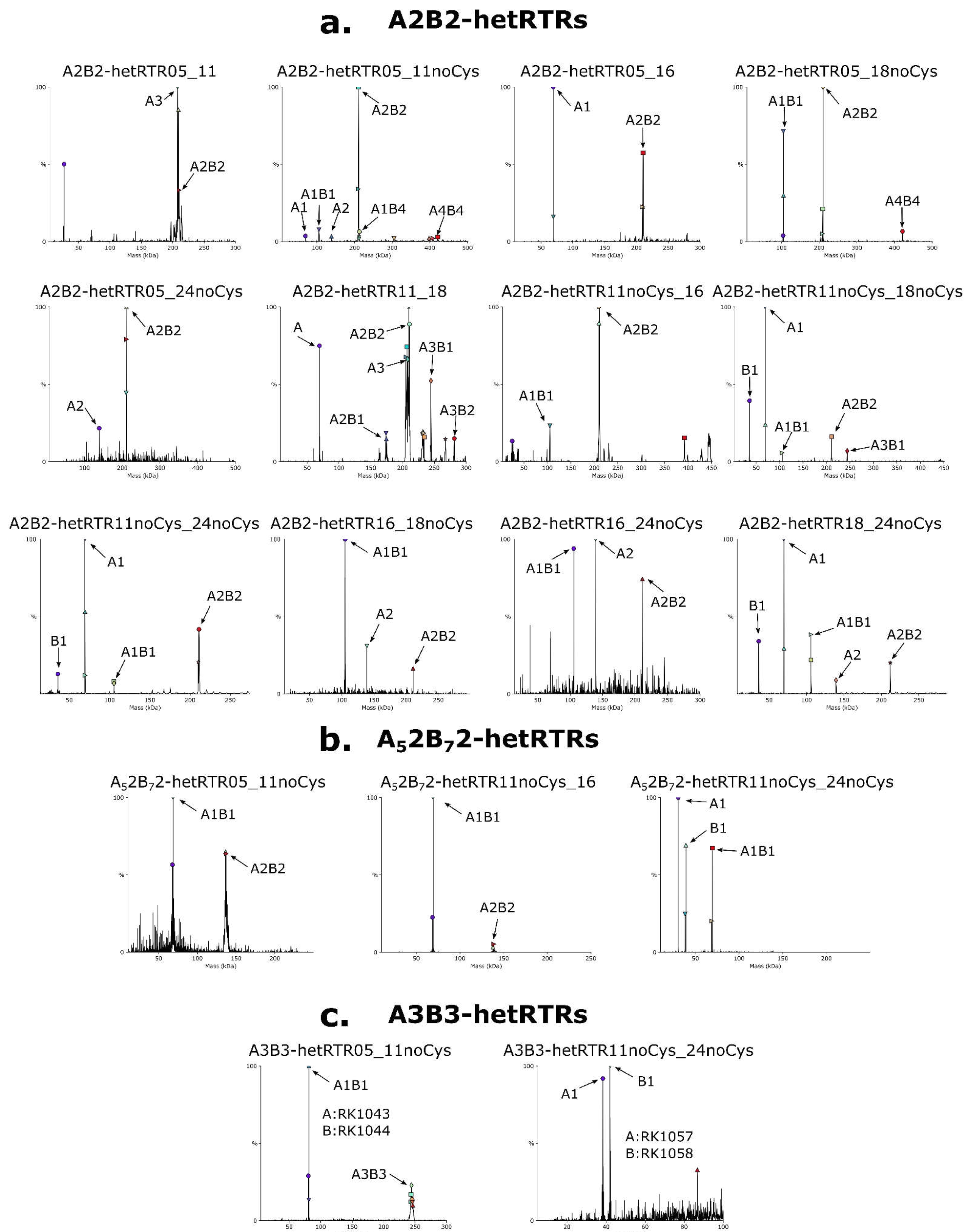

Supplementary Figure 19: nMS of hetRTRs

Plots of all designs of (a) A2B2-hetRTRs, (b) $A_5$2$B_7$2-hetRTRs, and (c) A3B3-hetRTRs with nMS data showing deconvoluted masses obtained from m/z spectra (Supplementary Data 13). Identified species corresponding are annotated with arrows and labels. Unannotated peaks are unknown species.

A2B2-hetRTR05_11$_{noCys}$

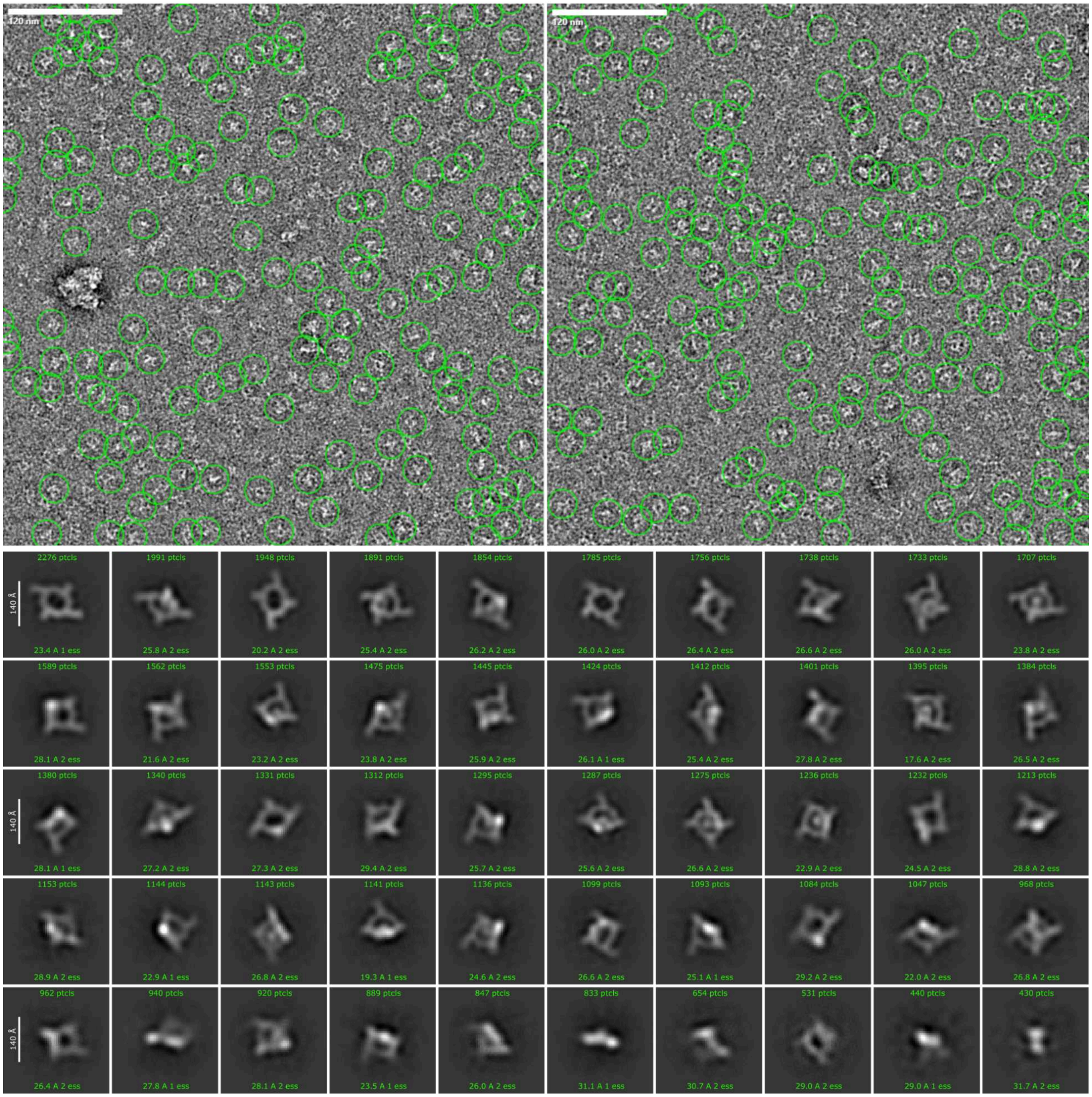

A_52B_72-hetRTR05-11$_{noCys}$

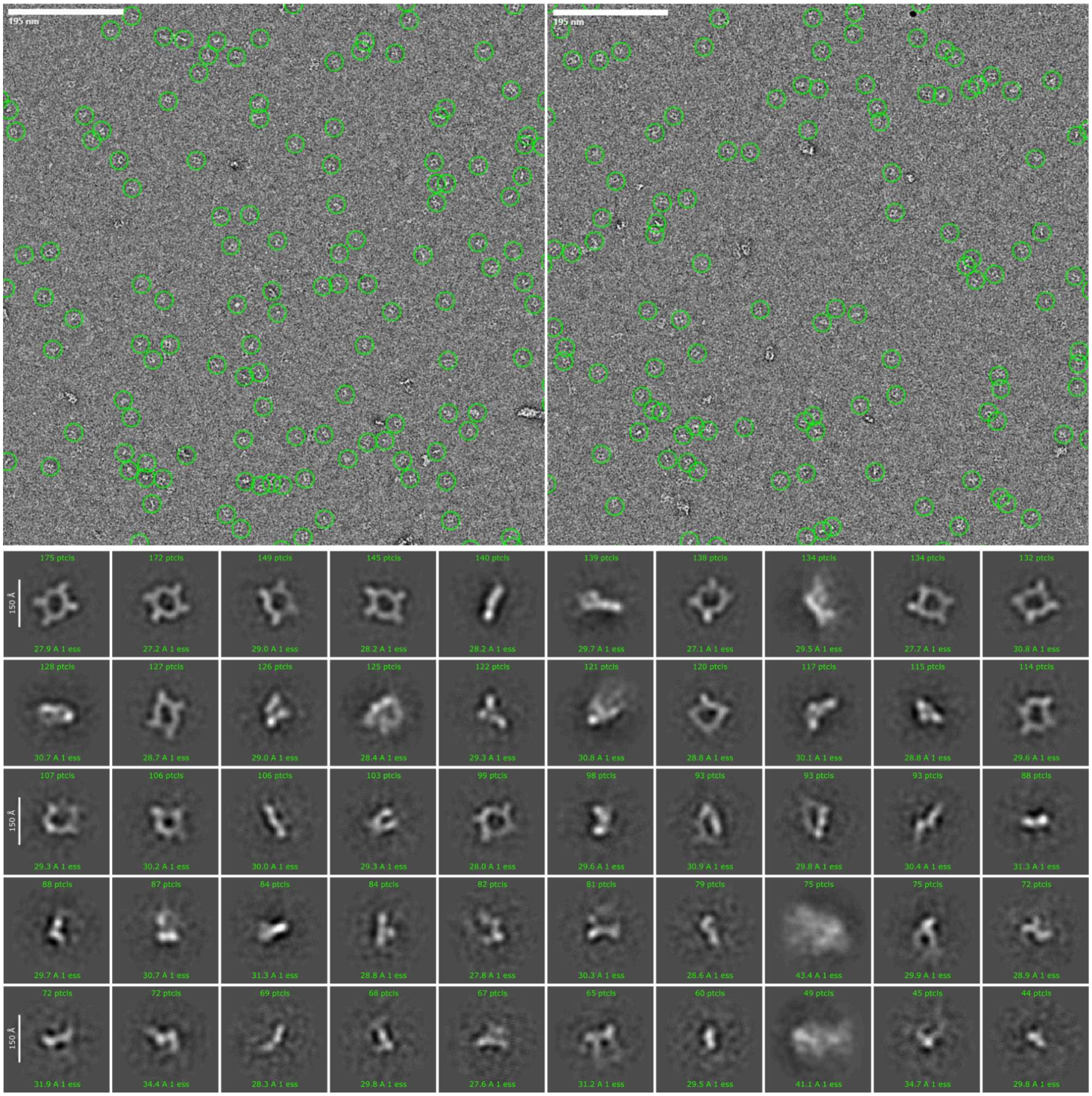

A3B3-hetRTR05-11$_{noCys}$

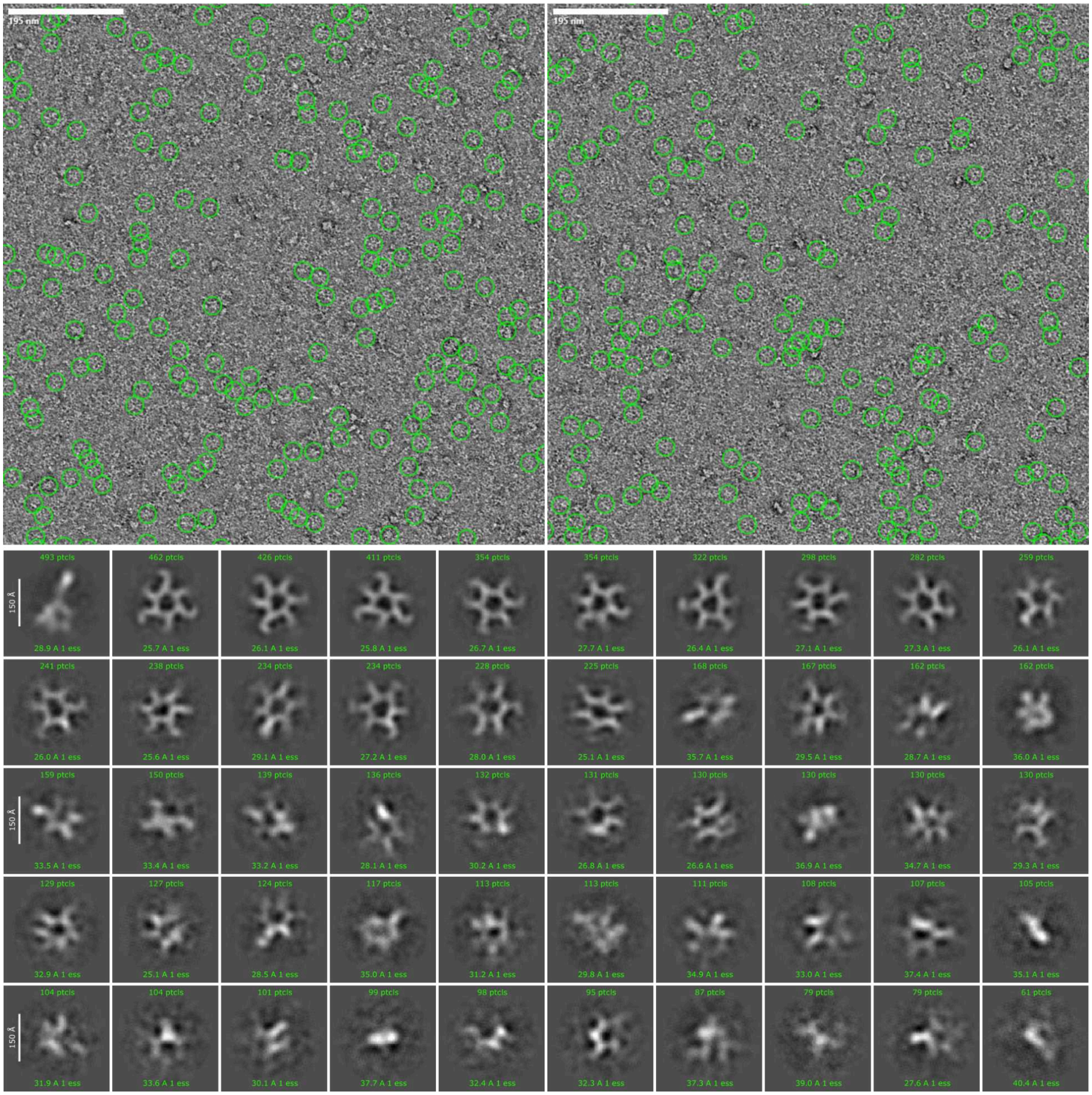

A2B2C2-hetRTR11$_{noCys}$-05-16

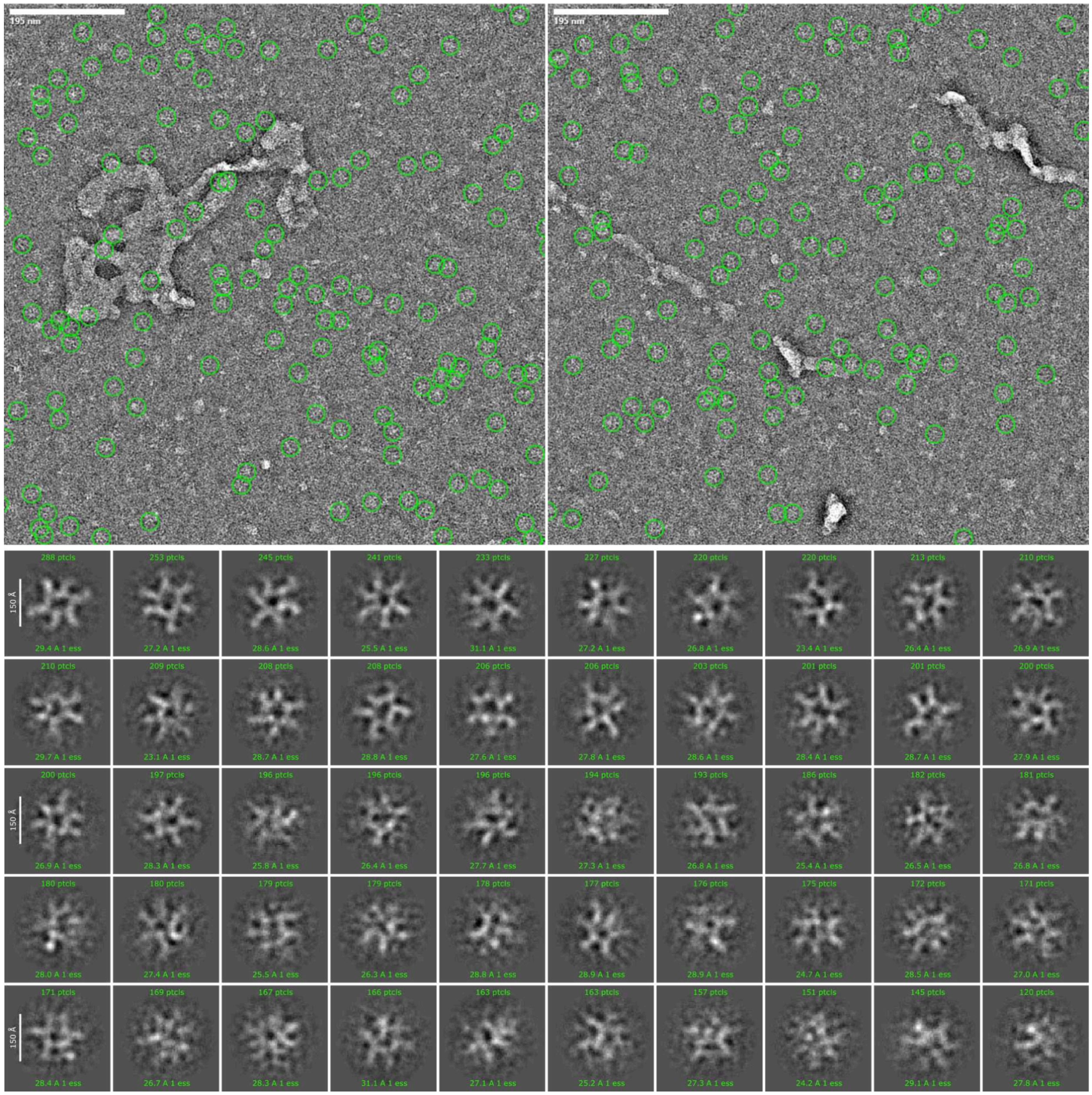

Supplementary Figure 20: nsEM micrographs and 2D class averages of RTR homo-oligomers
For each design, a pair of representative micrograph (top) with circled particles included in the dataset used to construct 2D class averages (bottom). Scale is indicated by labeled scale bars on the micrograph and the first class. Most datasets were collected a single time, with between 50 and 200 micrographs.

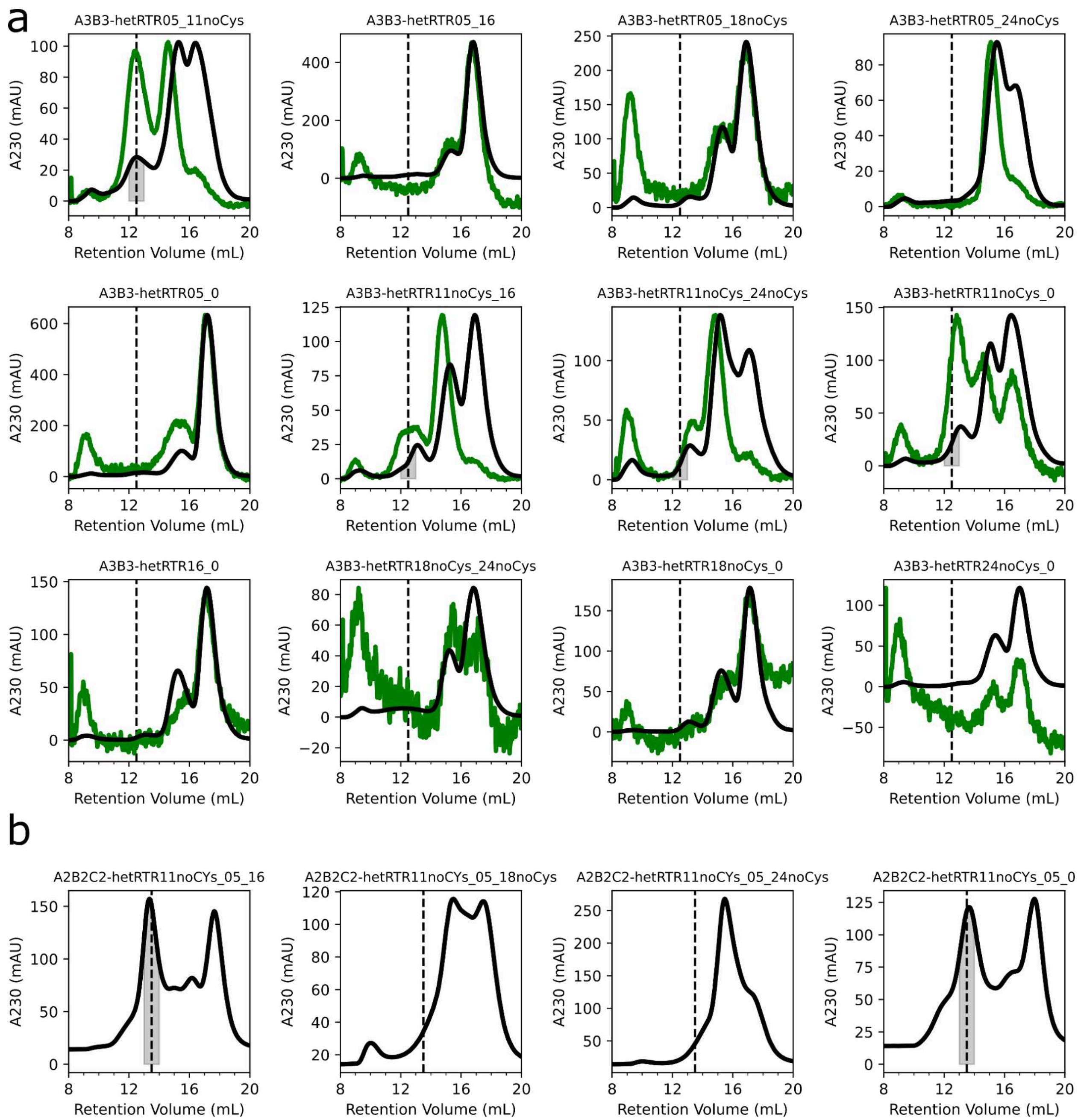

Supplementary Figure 21: SEC of A3B3/A2B2C2-hetRTRs

(a) A3B3 samples. Black, absorbance at 230nm. Green, absorbance at 480nm, corresponding to the GFP-tagged chain, normalized to max absorbance at 230nm. (b) A2B2C2 samples. Black, absorbance at 230nm. A2B2C2 genes were expressed with His6-Tags only.

Supplementary Table 1: BGL crystallographic information

Name	BGL06	BGL15	BGL18	hetBGL03-15-18	BGL14_styr
PDB accession code	8E0L	8E0M	8E0N	8E0O	8E12
**Crystallography**					
Well solution	20% (w/v) PEG-1000 phosphate-citrate pH 4.2, 200 mM Li2SO4	30% (v/v) PEG-400, Tris pH 8.5, 200 mM MgCl2	30% (v/v) PEG-400, CAPS pH 10.5	20% (w/v) PEG-3350, 0.1 M Bis-Tris pH 6.5, 0.2 M MgCl2	15% (w/v) PEG-3350, 0.1 M Magnesium formate dihydrate
Cryoprotectant	All flash-frozen in well solution plus 20% ethylene glycol				
**Data Collection**					
Space Group	$P2_1$	$P2_1$	$P3_2$	$P2_1$	$P3_221$
Unit Cell Dimensions	a=58.2 Å, b=78.9 Å c=58.7 Å, b=118.3°	a=105.8 Å, b=83.2 Å c=119.8 Å, b=93.0°	a=b=97.8 Å c=133.9 Å	a=58.9 Å, b=61.7 Å c=73.7 Å , [illegible]=100.2°	a=b=59.3 Å c=260.3 Å
Resolution	2.1 Å (2.14-2.1)	3.3 Å (3.39-3.33)	3.0 Å (3.05-3.0)	2.1 Å (2.18-2.1)	3.0 Å (3.05-3.0)
Reflections	27320	30779	28352	30567	10082
Completeness	99.8 % (99.7 %)	99.8 % (100%)	99.1 % (96.8 %)	98.7 % (99.6 %)	88.4 % (53.6 %)
Redundancy	6.4 (6.7)	6.4 (6.4)	10.3 (9.8)	9.9 (9.9)	8.4 (3.1)
$I/\sigma(I)$	56.2 (16.2)	11.0 (1.3)	10.9 (1.3)	27.1 (6.0)	28.5 (1.05)
R_{merge}	0.061 (0.132)	0.084 (1.682)	0.192 (1.224)	0.057 (0.354)	0.110 (0.660)
R_{pim}	0.026 (0.054)	0.033 (0.659)	0.063 (0.412)	0.019 (0.118)	0.038 (0.397)
CC1/2	1.002 (0.998)	0.999 (0.524)	0.999 (0.742)	1.000 (0.969)	1.003 (0.664)
Number of protein atoms	3571	10690	7569	3689	3074
Number of water atoms	110	0	13	55	1
**Refinement**					
Resolution	2.1 Å	4.0 Å	3.0 Å	2.1 Å	3.0 Å
R_{work} / R_{free}	0.210 / 0.255	0.280 / 0.328	0.207 / 0.259	0.238 /0.262	0.282 / 0.321
Rmsd bonds	0.002 Å	0.002 Å	0.002 Å	0.001 Å	0.002 Å
Rmsd angles	0.38°	0.52°	0.52°	0.35°	0.656°
Wilson B-factor	35.65	141.65	63.77	26.36	100.57
Ramachandran Distribution (Favored%/Allowed%/Outlier%)	99.38 / 0.62 / 0.0	97.69 / 2.05 / 0.26	99.31 / 0.69 / 0.0	98.99 / 1.01 / 0.0	98.32 / 1.26 / 0.42

Supplementary Methods

Computational Methods

All code is available at https://github.com/rdkibler/pseudosymmetric_hetero-oligomers

Protein design was performed using Rosetta (version 2018.41.post.dev+171.bcov.dev.507725abe46). While Rosetta is capable of performing every step of the interface redesign process using its symmetry machinery[3] and thus using a symmetric pose at every step, because we require the homology region to have identical sequence and coordinates between every design, we cannot make use of the concerted subunit rigid body transformation degrees of freedom that the symmetry machinery is, in part, intended to provide. Any full-chain movements away from the input structure in the homology region are not allowed. Additionally, we would not use the symmetry machinery's functions which make the packer and minimizer aware of the other chains in the structure due to the physical separation between the interfaces. Therefore, all steps until the final structure checks are performed on the structure asymmetrically using coordinate constraints to hold the homology regions in place. In the case of RTR0, we also got a large speed up by using a significantly truncated version of the interface region which included just the edge of the homology region.

1. BGL redesign

All BGL design was carried out on the full 18 helix structure of BGL0 (PDB 6XR1), since this is semantically equivalent to working on the fixed-size homotrimer using symmetry operations, and avoids the need for symmetry which increases the computational overhead.

1.1 Backbone perturbation

1.1.1 Helix Rebuilding (HR) set

Canonically coiled alpha-helical backbones can be described by their Crick helical parameters[4,5], that is, for a given helix coiling around the Z-axis, the values of r0, omega0, omega1, phi0, phi1, detla_z, and whether the helix runs toward negative Z (inverted) or toward positive Z (not inverted). With the axis of structural symmetry lying on the Z-axis, the outer helices of BGL0 resemble the helix of a coiled coil with very large r0 (distance from the Z-axis). We sought to resample the backbone positions of the outer helix at the interface, and reasoned that replacement helices closeby in the parameter space of the original helix would also fit well physically while giving a large increase in backbone position (especially via sampling uniformly over phi1). To accomplish this, we wrote an algorithm which discovered the 7 helical parameters

via least squares minimization. Most parameters were largely unconstrained save for r0 bounded between 1e-6 and 40 and phi0 bounded to >= 0. The parameters r0, omega0, omega1, phi0, phi1, and delta_z, were initialized to a generic left-handed supercoiled helix, with phi0 and r0 estimated from the position of the target helix's center of mass, and at every step the per-backbone-atom RMSD was computed between the target helix and a helix with the current parameters and a least squares algorithm was used to find the set of parameters which minimizes RMSD. The target in this campaign for BGL0 was the C-terminal helix, one of the outer ring of helices and the closest fit we achieved was 0.8A. Given this best description of the target helix as an ideal coiled coil helix, we deleted the target helix from the BGL0 structure and sampled 124,852 new helices with parameters r0 between 20 and 25, delta_z +/- 6 from the fitted value, omega0 +/- 1 degree from the fitted value, phi0 +/- 20 degrees from the fitted value, and phi1 +/- 154.3 degrees from the fitted value. Some solutions clashed with the rest of the BGL0 structure, so rather than fine-tuning parameter ranges which might have a complex relationship, we roughly evaluated clashing with a Rosetta scorefunction scoring only fa_rep, fa_atr, and fa_sol (delta REU from original structure < 9000) and buried surface area (at least 350 with either side of the interface).

The output of the helix rebuilding procedure is a disconnected helix roughly in the same position as the target helix and it needs to be re-connected to the rest of the structure via a loop. Because BGL0 is a toroid, the N- and C-termini are physically adjacent to each other and could be re-connected to either side. Therefore, for each output of helix rebuilding, we attempted to re-connect the helix as either the N-terminus (building a loop between the current N-terminus of the full structure and the new helix) or the C-terminus (building a loop between the current C-terminus of the full structure and the new helix) using ConnectChainsMover[6] with loop lengths between 2 and 5 residues and the option to add or remove five residues from either side of the loop. This resulted in the creation of 3524 outputs each for N-term closure and C-term closure.

Extra functionality

While not necessary for this work, we found that Crick helical parameters do not always generalize to helical segments which appear curved like coiled coils but, for example, whose superhelical curvature is on an axis which does not pass through the center of mass of the protein. To account for this, we have also developed a method for fitting a reduced set of Crick helical parameters and 6 additional parameters corresponding to a homogeneous transform (3 rotation and 3 translation) of the helix using least squares minimization. The parameters made redundant by this approach and which are thus never sampled are delta_z and invert because they can be easily described by the transform parameters. The algorithm proceeds in three phases.

In the first phase, the goal is to roughly locate the superhelical axis. The helix parameters are initialized to those of a generic left-handed superhelical helix (r0 = 5, phi1=0 degrees, phi0=100 degrees, omega0=-2.85 degrees, omega1=102.85 degrees) and the transform is initialized to a rough initial guess based on the target helix's center of mass and orientation of the termini relative to the Z-axis. Phi1 and transform are fitted to the target helix to refine the initial guess of the transform of the superhelical axis. The signal from the phase of the minor helix (the atomic positions) is much stronger than the major helix (superhelical axis), so phi1 is allowed to vary to avoid biasing the transform by a bad guess of phi1.

In the second phase, which may not be necessary, the transform is fixed and omega1, omega0 (coupled to omega1 via the relationship abs(abs(omega0+omega1)-radians(100)) < 10), phi0, and phi1 are allowed to minimize in order to roughly shape the helix and get it close to its final form in the context of a new superhelical axis.

In the third phase, r0, omega0, omega1, phi1 (not phi0), and the transform are allowed to vary to simultaneously fine-tune the helical parameters and the effective location of the superhelical axis. The tolerance of the fit is set much tighter than for phases 1 or 2. Given a set of parameters that describe the new helix, a similar closeby parameter search and hash table clash check could be performed.

1.1.2 Normal Modes (NM) Relaxation

Separately from HR, we generated conformations very similar to the native structure but which may differ only via molecular breathing type motions by applying the NormalModeRelaxMover (NMRM)[7] to generate another set of designs (NM). We restricted backbone perturbation to the two and a half helices on either side of the interface (N-term Interface: n-term through the middle of the 3rd helix; C-term Interface: the middle of the 3rd-to-last helix through the c-term), and additionally used coordinate constraints to hold everything else in place. NMRM was run successively and separately on the C-term Interface then the N-term interface, with the rationale that allowing the movement of the C-term Interface and its outer C-terminal helix first would create room for a greater range of movement by the N-term Interface. 500 backbones were generated with pertscale=2.0, nmodes=60, and mix_modes=true for both NMRM runs.

1.2 HBNet design

HBNet design was carried out slightly differently depending on the design set. Common to both were the region within which HBNets could be designed (the union of the N-term Interface and C-term interface), as well as a requirement to have at least 4 network residues in the core, have no unsatisfied heavy atoms or polar hydrogens, make at least 2 intermolecular hbonds, and have between 1 and 3 networks in the output structure. Hb_threshold was set to -0.6 and the MC sampling was turned on with seed_hbond_threshold -0.4 and 10,000 MC steps

for HR and 100,000 for NM for deeper sampling. Core cutoff for HR was set to SASA < 20 (ball radius 2.5), but for HR was SASA < 6 (more stringent) to encourage deeper HBNets at a cost to sampling.

The HBNets created for the NM set were highly redundant due to the similarity of the backbones inputs and depth of sampling, so the 12954 outputs were grouped by the sequence of their buried HBNet residues (SASA < 20 with ball radius 2.5) and the best scoring output was kept for each group (3332 total).

The HBNets for HR could not be grouped as easily but were qualitatively more diverse, so the 112039 outputs were instead briefly fixed-backbone designed with HBNet's AtomPair distance constraints on and sidechain relaxed with constraints off to allow poorly positioned and/or poorly packed HBNets to move out of position. Then, designs were filtered on vbuns <= 2, # alanines <= 126, and total HBNet constraint score (indicating movement of HBNet residues) < 1.0, yielding 2538 outputs. The new helix and new loops were cartesian minimized in preparation for interface design.

1.3 Interface design

In both cases, flexible backbone FastDesign was performed with the standard suite of TaskOperations (InitializeFromCommandline, DesignRestrictions, IncludeCurrent, LimitAromaChi2, and ExtraRotamersGeneric [ex1 & ex2]). Design was carried out with variable bondangle and bondlengths, and the approximate_buried_unsat_pentality and PruneBuriedUnsats[8] were used to aid in retaining or extending the HBNets. Flexible backbone movement was only allowed on the N-term Interface and C-term Interface regions, but sequence design was allowed at the interface between the Interface regions and in a 7A neighborhood around each HBNet residue (but not including the HBNet residues or proline or glycine). The residues within 6A of the designable region were allowed to repack and sidechain minimize and the HBNet residues had their AtomPair constraints enabled. FastDesign was carried out with a modified version of the MonomerDesign2019 relax script which does not ramp coordinate constraints.

1.3.1 HR specific

Calpha atoms were loosely coordinate constrained over the entire pose. The pose has a reasonable sequence already from the HBNet filtering step, so only minor changes to core packing were allowed for L, I, and V residues which were only allowed to change to L, I, or V. PruneBadRotamers and ConsensusLoopDesign TaskOperations were also employed. The surface was not designed in order to speed up design around the HBNets which were buried. Finally, because the new helix created by HR was given a "rosetta default" sequence, additional amino acid composition constraints were used to constrain the structure to have an amino acid

sequence profile roughly similar to the original design, namely requiring at most 1 methionine, 127 alanines, 2 phenylalanines, and 5 threonines, with some combination of linearly increasing penalties within a small range then increasing quadratically at higher counts. 7 separate design trajectories were run on each input, resulting in creation of 12256 outputs.

1.3.2 NM specific

Calpha atoms were tightly coordinate constrained only over the non-Interface region to allow more movement at the Interface. 7 separate design trajectories were run on each input, resulting in creation of 12879 outputs.

1.4 Filtering

Designs were filtered on a variety of criteria to reduce each design set to 100 for manual inspection. The criteria are the following:

HR:

- Cst_after < 1.0
- Mismatch_probability < 0.06
- Network_holes < -1.4
- Interface_holes < 0
- dSASA_polar > 1500
- vbuns5.5_heavy_ball_1.1D = 0
- RotamerBoltzmannWeight (HBNet resis) < -0.51
- lowest 100 by worst9mer

NM:

- Cst_after <1.0
- Network_holes < -1.4
- Interface_holes < 0
- P_aa_pp < -130
- Mismatch_probability < 0.07
- vbuns5.5_heavy_ball_1.1D = 0
- RotamerBoltzmannWeight (HBNet resis) < -0.52
- Highest 100 by dSASA_polar

The sets were then manually inspected, visually looking for hydrophobic interdigitation in the core, interesting and practical HBNets, and reasonable burial of HBNets. Finally, 16 HR designs and 4 NM designs from each set of 100 were rearranged to be (6 helix) homotrimers, ordered, and characterized.

1.2 RTR redesign

Design was carried out on a truncated sub-structure of RTR0 focusing on the interface region, comprising just 210 residues which represent four helices from the N-terminal side of the interface (two participating in the interface and two representing the fixed homology region) and 8 helices from the C-terminal side of the interface (4 which participate in the interface, two which reprepresent the fixed homology region of the ring, and two which represent the fixed part of the C-terminal extension).

1.1 Backbone perturbation

NMRM parameters were optimized to create a variety of pocket sizes and shapes in the interface (pertscale = 1.6, nmodes = 31, mix_modes=true). The regions allowed to move, Interface A and Interface B, were defined as regions 1-33 and 100-174 on the truncated "mini"-pose RC4_20_mini.pdb. Before running NMRM, the sequence of the pose was changed to polyvaline and the beta_nov16_soft weights were used in order to allow the structures to slide past each other more easily. NMRM was also run in a single step, letting both interfaces move at once. After perturbation, the original sequence was restored. A total of 15500 outputs were produced.

To reduce the runtime of the protocol, the NMRM source code (source/src/protocols/normalmode/NormalModeRelaxMover.cc) was modified. NMRM stores all computed conformations it creates during the course of a given trajectory but only returns the best scoring one (throwing away the rest). We updated the mover to allow retrieval of all computed conformations via a MultiplePoseMover in either a random or best-score-sorted order.

1.2 HBNet design

The region where HBNets can be designed into the RTR interface is smaller and empirically more difficult to find extensive HBNets at diverse positions. Therefore, HBNet design in the case of RTR was modified to require fewer HBNet residues to be in the core and filtering was set up to require that at least one buried HBNet residue be on either side of the interface. max_unsat_Hpol was also increased to two. See production_varset.json for a complete list of parameters used.

HBNet produced 15692 outputs and we found 3815 unique networks. We kept either the best 3 or the best 10% of structures with each network, whichever was higher, for a total of 12649.

1.3 Interface design

The interface between Chain A and Chain B and the region 7.0 A around the HBNet residues, not including the HBNet residues themselves, were allowed to redesign, excluding serine 154 which seemed to be important. Flexible backbone movement was allowed for the

n-terminal two helices and loops on chain A and the four helices which are closest to chain A on chain B with all other residues (essentially the two helices on chain A and chain B which connect the interface to the rest of the ring and the two helices which connect the interface to the rest of the extension on chain B) were fixed in place with coordinate constraints and prevented from backbone minimization. Then flexible backbone FastDesign was carried out in cartesian space with HBNet residue AtomPair distance constraints, allowing at most 2 methionines, allowing at most 2 boundary tryptophans, and requiring between 1 and 3 boundary tyrosines. The same task operations were used as 1.3 for NM designs.

Next, the surface was separately redesigned with sequence constraints which require a very negative net charge to ensure any combination of interfaces downstream has a very low isoelectric point (pI). Designs were filtered to have mismatch_probability, core_holes, interface_sc, and ss_sc not worse than the parent structure, resulting in 880 unique outputs.

The minimal structures were then converted back into the full (tetrameric) ring through superimposition of the homology region helices with the corresponding regions of the full structure and splicing of the redesigned interface into the full structure using PyMol (Schrödinger). The symmetric structures were then cartesian minimized with HBNet constraints on with Rosetta to fix any bad backbone geometry from the splicing procedure, and finally they were flexible backbone relaxed without HBNet constraints.

1.4 Filtering

DeepAccNet[9] (version a54150749c80c9a9395c0900d91d5c88b4f54d76) was used to predict the lddt of the designs post-relaxation, and any designs with residues with lddt < 90 were discarded. Designs which did not increase much in energy after flexible backbone relaxation without HBNet constraints (REU after - REU before < 0.5) were also discarded, resulting in 79 designs which were subsequently visually inspected and narrowed down to 24 designs.

All interface design was previously carried out while disallowing cystine, but an interface disulfide seemed to have been important for high order structure assembly[10], so we used stapler (https://github.com/atom-moyer/stapler) to consider potential positions for disulfide bonds to exist on the selected designs, then chose positions which were as different as possible from the others in the chosen set. These were then ordered and characterized.

2. Computational recombination

2.1 hetBGLs

The process of combining multiple homo-oligomers into one hetero-oligomeric structure was carried out using a python script, recombine_2.1.py. It uses PyMol to superimpose and splice together homo-oligomer structures at the specified recombination point in the desired order, controlled through commandline arguments. The recombined structure could then be

directly outputted, or optimized using Rosetta cartesian backbone minimization to fix any bad backbone geometry. Additionally, because local interface pI was not considered during the initial BGL design, some naive combinations could have individual chains with pI >= 8, which would result in neutrally or positively charged chains which are more likely to have expression and stability issues. Therefore, the surfaces of hybrid chains could also be redesigned to enforce a small pI for each chain individually in the final hetero-oligomers. Finally, if not present before, tyrosines were added to the surfaces to act as crystal contacts to aid crystallography.

2.2 hetRTRs

Recombination of RTRs into hetRTRs was done either using a version of the recombinase script which does not perform surface design, or with a text editor to simply combine the amino acid sequences to form the appropriate hybrid chains. In the latter case, design models were constructed manually using PyMol.

Experimental Methods

Buffer and media recipes

All buffers and media were made using Milli-Q filtered water.

Autoinduction media (TBM-5052)

1.2% [wt/vol] tryptone, 2.4% [wt/vol] yeast extract, 0.5% [wt/vol] glycerol, 0.05% [wt/vol] D-glucose, 0.2% [wt/vol] D-lactose, 25 mM Na_2HPO_4, 25 mM KH_2PO_4, 50 mM NH_4Cl, 5 mM Na_2SO_4, 2 mM $MgSO_4$, 10 µM $FeCl_3$, 4 µM $CaCl_2$, 2 µM $MnCl_2$, 2 µM $ZnSO_4$, 400 nM $CoCl_2$, 400 nM $NiCl_2$, 400 nM $CuCl_2$, 400 nM Na_2MoO_4, 400 nM Na_2SeO_3, 400 nM H_3BO_3

Lysis buffer

25 mM Tris, 300 mM NaCl, 20 mM imidazole, 10% glycerol, pH 8.0 at room temperature

Wash buffer

25 mM Tris, 300 mM NaCl, 40 mM imidazole, 10% glycerol, pH 8.0 at room temperature

Elution buffer

25 mM Tris, 300 mM NaCl, 300 mM imidazole, 100 mM EDTA, 10% glycerol, pH 8.0 at room temperature

Het Lysis buffer

25 mM Tris, 300 mM NaCl, 10 mM imidazole, 10% glycerol, pH 8.0 at room temperature

Het Wash buffer
25 mM Tris, 300 mM NaCl, 20 mM imidazole, 10% glycerol, pH 8.0 at room temperature

Het Elution buffer
25 mM Tris, 300 mM NaCl, 50 mM imidazole, 10% glycerol, pH 8.0 at room temperature

SEC buffer
25 mM Tris, 300 mM NaCl, pH 8.0 at room temperature

SAXS buffer
25 mM Tris, 300 mM NaCl, 2% glycerol, pH 8.0 at room temperature

TEV buffer
25 mM Tris, 100 mM NaCl, 0.5 mM EDTA, 1mM DTT, pH 8.0 at room temperature

AEC buffer A
25 mM Tris, pH 8 at room temperature

AEC buffer B
25 mM Tris, 1M NaCl, pH 8 at room temperature

Construction of synthetic genes

All synthetic genes were ordered from either Integrated DNA Technologies Inc. (Coralville, IA, USA) (IDT) or Genscript Inc. (Piscataway, NJ, USA). Genes ordered from IDT were reverse translated and codon optimized using Domesticator (https://github.com/rdkibler/domesticator_3). Protein tags were added to aid in purification (His6-tag) or identification (EHEE_rd2_0005 (aka EHEE)[11], superfolderGFP, or mScarlet-I) were separated from the designed protein with a tobacco etch virus protease (TEVp) cleavage site (ENLYFQG). In many cases, stop codons were introduced at the end of the inserted gene to prevent incorporation of the vector's built-in tags. Cloning into pET29b+ or its derivatives is always done at the NdeI/NcoI site.

For expression of BGLs, protomer sequences were extracted and the n-terminal tag "MGHHHHHHGSENLYFQGWS" was added. Codon-optimized genes with a 3' stop codon were cloned into pET29b+ by IDT (Supplementary Data 1).

For co-expression of hetBGLs, all three proteins were arranged sequentially off the same transcript, separated by ribosome binding sequences (RBSs) (first: TAAGAAGGAGATATCATCATG; second: TAAAGAAGGAGATATCATATG). One protomer received superfolderGFP, another received EHEE, and the third was untagged. One protomer (with either superfolderGFP for the EHEE) received a His6-tag. These were ordered from IDT (Supplementary Data 4).

For expression of RTRs, amino acid sequences (Supplementary Data 7) were reverse translated and inserted into pET29b+ with a stop codon added before the end of the insert. These were ordered from IDT.

For co-expression of tetrameric hetRTRs, amino acid sequences from Supplementary Data 10 corresponding with the A2B2-hetRTRs were reverse translated and inserted into a pET29b+ vector variant which expresses the N-terminal superfolderGFP tag such that chain A was tagged with TEV-cleavable sfGFP. Chain A and B were separated by an RBS (TAAGAAGGAGATATCATCATG), and chain B received the vector's C-terminal His6-tag. The sequences corresponding to A(5)2B(7)2 were prepared in the same way but inserted into the standard pET29b+ vector. All C4 sequences were ordered from IDT and everything else was ordered from Genscript.

For separate expression of hexameric hetRTRs, the amino acid sequences from Supplementary Data 12 were reverse translated and inserted into pET29b+ using the vector's C-terminal His6-tag. These were ordered from Genscript.

Transformation and expression

Plasmids (100ng) were transformed into chemically competent E. coli expression strain BL21(DE3)Star (Invitrogen) for protein expression following manufacturer's protocol, with the exception of using 10ul competent cells per reaction. Following transformation and recovery, the entire transformation products were used to inoculate 1 mL Luria-Bertani (LB) medium containing 100 ug/mL kanamycin and grown at 37°C with shaking at 225 rpm overnight. 500ul of overnight cultures were diluted into 50 mL TBM-5052 supplemented with 100 ug/mL kanamycin in 250 mL baffled flasks, and incubated at 37°C with shaking at 225 rpm for 18-24 hours.

Immobilized metal affinity chromatography (IMAC)

Cultures were harvested by centrifugation at 4000 rcf for 10 minutes, culture supernatant decanted, and pellets resuspended to 30 mL in Lysis buffer. 300ul PMSF (100mM in

100% EtOH) is added immediately prior to sonication at 70% power for 5 minutes. “Lysate” fractions are saved, and then lysates were clarified by ultracentrifugation at 14,000 rcf at 12°C for at least 30 minutes and applied to 1.5 mL Ni-NTA resin (Qiagen) pre-equilibrated with Lysis buffer and packed into Econo-Pac columns (Bio-Rad) for gravity chromatography. The columns were washed twice with 15 mL Wash buffer and eluted with 10 mL Elution buffer.

Co-expressed hetero-oligomers were purified according to a similar procedure, except they used the “Het” variants of the the Lysis, Wash, and Elution buffers, followed by a final elution with the standard Elution buffer, as this was found to improve the yield of single-His6-tagged complexes over non-specific multiple-His6-tagged complexes which can arise at high concentrations and likely dominate binding to the Ni-NTA resin. Samples prepared for crystallization were treated similarly, except 500 mL cultures were used and lysate was divided among six gravity columns.

Size-exclusion chromatography (SEC)

Samples were concentrated using 10k MWCO spin concentrators and were purified using a Superdex 200 10/300 increase column (Cytiva) in SEC buffer using an ÄKTA Pure system (Cytiva). SEC traces were also used to qualitatively determine homogeneity and quantitatively measure total yield by A280 absorbance integrated over the collected fractions using Unicorn 7 (Cytiva) for samples where the detector was not saturated, otherwise a NanoDrop 8000 (ThermoFisher) was used.

TEVp cleavage

All fractions excluding the void peak (fractions 4-12 with retention volumes 11-20ml) from SEC following IMAC were pooled and buffer exchanged into TEV buffer by dilution and cleaved with TEVp at a ratio of 1 mg TEVp per 100 mg substrate for 24-72h at room temperature. After TEVp cleavage, samples were exchanged into Lysis buffer by 10k MWCO spin concentrators, passed over a Ni-NTA gravity column, and washed with 10ml lysis buffer. Flowthrough was collected, concentrated using 10k MWCO spin concentrators, and purified once again by SEC using the same parameters as the previous SEC. Fractions indicated on Figures S4, S9, and S13 were pooled for use in downstream analysis.

Anion exchange chromatography (AEC)

TEVp-cleaved samples of hetBGL03-15-18 intended for crystallization remained contaminated with GFP due to an oversight that meant GFP did not have a His6-tag. To remove the GFP, samples were exchanged into AEC buffer A and loaded onto a HiScreen Q FF (Cytiva) column pre-equilibrated in AEC buffer A using an ÄKTA Pure system. A gradient of AEC buffer B was applied with pauses at 15% (150 mM NaCl) and 25% (250mM NaCl) to elute the GFP and

hetBGL03-15-18, respectively. Separation was measured by differential absorbance at 480 nm (GFP absorbance) and 280 nm, and SDS-PAGE.

SDS-Polyacrylamide Gel Electrophoresis (SDS-PAGE)

Samples were diluted 1:1 with 2x Laemmli Sample Buffer (Bio-Rad) without Beta-mercaptoethanol and 15ul were loaded onto AnykD™ Criterion™ TGX™ Precast Midi Protein Gels (Bio-Rad). Ladder was 10ul of Precision Plus Protein™ Kaleidoscope™ Prestained Protein Standards (Bio-Rad). Gels were run at 300V for 18 minutes, then stained using an eStain™ L1 Protein Staining System (Genscript). Stained gels were imaged using a Chemidoc XRS+ (Bio-Rad).

Liquid chromatography mass spectrometry (LC-MS)

To identify the molecular mass of each protein and thus verify sample identity and integrity, intact mass spectra was obtained via reverse-phase LC/MS on an Agilent G6230B TOF on an AdvanceBio RP-Desalting column, and subsequently deconvoluted by way of Bioconfirm using a total entropy algorithm.

Size Exclusion Chromatography - Multi Angle Light Scattering (SEC-MALS)

Samples were purified by IMAC and SEC (excluding only the void fractions 1-3 for buffer exchange and removing soluble aggregates) then concentrated to ~5mg/ml by 10k MWCO spin concentrators. 100uL of each sample was injected on a Superdex 200 10/300 in 20 mM Tris, 150 mM NaCl, pH 8 and run at 1 mL/min on an Agilent 1200 series HPLC in line with a Heleos multi-angle 9 static light scattering and an Optilab T-rEX detector (Wyatt Technology Corporation). Data was analyzed using ASTRA (Wyatt Technologies) to calculate the weighted average molar mass (Mw) of the selected species.

Supplementary Notes

Supplementary Note 1: Scaffold selection

BGL family

We chose tcTRP9_sub3 as the starting scaffold. It was described in its original publication[1] to have good expression and to purify a homogenous trimer. The structure of the interface strongly resembled that of the helical bundles used in Chen et al[12], which both gave us confidence that a diverse set of HBNets could be found to achieve a similar level of orthogonality. The use of similar backbones also allowed us to adapt the sequence design pipeline developed for that work for use here.

RTR family

For the second example scaffold, we selected C4_1 from Courbet et al[2], which is a fusion between cTRP24[10] and DHR20[13]. We were inspired to use C4_1 due to its high internal symmetry with 24 repeats. Because 24 can be divided into many factors (1, 2, 3, 4, 6, 8, 12, 24), it could in theory be separated into many different oligomers. The ability for cTRP24 to be split was initially found to be limited to just dimer (12 repeats per chain)[10], but after fusion to DHR20 and some interior surface redesign, C4_1 was able to exist as a tetramer. We suspect that the added interface surface area caused by the DHR fusion enables C4_1 to exist in higher oligomeric states. Another important advantage of C4_1 over the parental cTRP24 is the DHR arm is visible under nsEM and allows unambiguous determination of oligomeric state by counting.

Supplementary Note 2: BGL0 behavior

For side-by-side comparisons, we synthesized the parental design with the same purification tags used for the BGL redesigns and observed markedly different biophysical behavior from what was initially reported[1], including aggregation and the appearance of an apparent monomeric species by SEC after TEVp cleavage. We speculate that this was caused by differences in the expression construct. The constructs differed in the following ways:

1. Cleavage site. The original used a thrombin-cleavable sequence to remove the His6-tag, but the lab where the protein purification was performed does not use thrombin so the choice was made to switch to a TEVp-cleavable sequence. This cleavage site may not be very accessible, leading to inefficient TEVp cleavage.
2. N-terminal tryptophan: To facilitate spectroscopic determination of protein concentration, all BGL constructs include an N-terminal tryptophan in a “GWS” tag right before the structured region.

3. Single residue permutation: The N-terminal Asn of the original structural region was inadvertently moved to the C-terminus in the new construct. This residue is located on the loop that was cut to generate the sub3 variant, and the design is cyclic, so this permutation should not be a major concern.

Future purification campaigns should consider using an alternative tag construct, such as using the thrombin cleavage sequence and/or removing the N-terminal tryptophan. Perhaps other BGL redesigns would also benefit from a better expression construct. See below for the expression constructs with His6-tag in blue, cleavage site underlined in red with cleavage tag in red (cleavage location indicated with a "/"), spectroscopic tryptophan in purple, and structural sequence in green:

>Original (thrombin cleavage)
MASS**HHHHHH**SSG**LVPR/GS**SMG**NLELALKALQILVNAAYVLAEIARDRGNEELLEKAARLAEEAARQAEEIARQARKEGNLELALKALQILVNAAYVLAEIARDRGNEELLEKAARLAEEAARQAEEIARQARKEGNLELALKALQILVNAAYVLAEIARDRGNEELLEKAARLAEEAARQAEEIARQARKEG**

>This work (TEVp cleavage)
MG**HHHHHH**GS**ENLYFQ/GW**S**LELALKALQILVNAAYVLAEIARDRGNEELLRKAARLAEEAARQAEEIARQARKEGNLELALKALQILVNAAYVLAEIARDRGNEELLEKAARLAEEAARQAEEIARQARKEGNLELALKALQILVNAAYVLAEIARDRGNEELLEKAARLAEEAARQAEEIARQARKEGN**

Supplementary Note 3: RTR Extensions

Negative stain electron microscopy (nsEM) was the most useful technique for qualitatively determining RTR oligomeric state and general shape due to the easily recognizable studded ring structure of the protein assembly. To use nsEM to distinguish between chains for determining chain identity and stoichiometry of the pseudosymmetric hetRTRs we needed to significantly alter their shapes without affecting assembly characteristics. Altering the C-terminal studs on the ring is a good option for this due to the physical separation between the C-terminus and the ring interface. The 'stud' is DHR20, a designed helical repeat protein[13], and there are a number of pre-characterized fusion junctions between the C-terminus of DHR20 and other DHR proteins that we could use[6]. By trimming off the C-terminal soluble "cap" repeat of DHR20 on the RTR scaffold and the N-terminal soluble cap repeat of a DHR20 junction, we can make simple structural fusions by concatenating the trimmed RTR and junction sequences.

We chose 9 of these simple fusions to characterize based on maximizing the visual differences between designs. Additionally, DHRs can be trivially extended or contracted by changing the number of internal (non terminal 'cap') repeats, so we also tested one one shortened ('arm2') and two lengthened ('arm4' and 'arm5') versions of the DHR20 stud.

Because the studs are supposed to be identical between all designs, we arbitrarily chose the tetrameric version of RTR24 as a nicely expressed test case. These were characterized by SEC (Supplementary Fig. 17a) and found to elute generally around the same volume, except for the arm2 fusion which may have destabilized the interface. These samples had varying degrees of soluble aggregation, with the worst being for the fusion to DHR80_2. Many samples also had another smaller peak that eluted earlier than the main peak which we hypothesized is a dimer of the tetramer but never confirmed.

We ordered constructs for testing the hexameric hetRTRs assemblies before completing the structural characterization of the RTR24 fusions, chosen based only on SEC results which indicated assembly and structural diversity under the assumption that the fusion was correct. This assumption was made due to the pre-validated nature of all the parts involved. We had chosen the arm5, DHR62, DHR70_1, DHR80_1, and DHR81 Extensions to be paired with the interfaces from RTR18noCys, RTR24noCys, RTR05, RTR16, and RTR11noCys, respectively, in the hybrid subunits making the hetRTR assemblies. Afterward, we collected SAXS and nsEM data and found most chosen Extensions to indeed have excellent agreement with their design models (Supplementary Fig. 17c,d; Supplementary Data 9, lower part), with the exception of the DHR62 Extension (in RTR24_DHR62) which had a deviated SAXS profile and P(r) plot lacking the left density. While studded ring-shaped particles can be found on nsEM, the majority of particles are misshapen and do not resemble the ring (Supplementary Fig. 16). It is likely no coincidence, therefore, that all hetRTRs using the DHR62 extension failed to assemble. We recommend that future efforts with this scaffold use the similarly-shaped but more performant DHR52 extension instead – thus the number of structurally validated extensions remains 5 which, in combination with with the original non-extended stub, gives 6 unique arms that can be used to test a fully asymmetric ABCDEF-hetRTR.

We also experimented with making alterations to the N-terminus using HFuse[14] to attach miniproteins[15] with C-terminal helices that superimpose well to the N-terminal RTR helix without clashing with the rest of the structure. This fusion was more risky due to the proximity to the ring interface at the first two helices of the RTR ring, which did result in a greater variation in SEC profile shapes (Supplementary Fig. 17b). While we did structurally validate one fusion, RTR11_C6_noCys_1d6b5438aa0d917bea2962dcb63062e6_0001_1, we did not use it with any hetRTRs.

Supplementary References

1. Hallinan, J. P. *et al.* Design of functionalised circular tandem repeat proteins with longer repeat topologies and enhanced subunit contact surfaces. *Commun. Biol.* **4**, 1240 (2021).
2. Courbet, A. *et al.* Computational design of mechanically coupled axle-rotor protein assemblies. *Science* **376**, 383–390 (2022).
3. DiMaio, F., Leaver-Fay, A., Bradley, P., Baker, D. & André, I. Modeling Symmetric Macromolecular Structures in Rosetta3. *PLOS ONE* **6**, e20450 (2011).
4. Crick, F. H. C. The Fourier transform of a coiled-coil. *Acta Crystallogr.* **6**, 685–689 (1953).
5. Grigoryan, G. & DeGrado, W. F. Probing Designability via a Generalized Model of Helical Bundle Geometry. *J. Mol. Biol.* **405**, 1079–1100 (2011).
6. Brunette, T. *et al.* Modular repeat protein sculpting using rigid helical junctions. *Proc. Natl. Acad. Sci.* **117**, 8870–8875 (2020).
7. Park, H., DiMaio, F. & Baker, D. CASP11 refinement experiments with ROSETTA. *Proteins Struct. Funct. Bioinforma.* **84**, 314–322 (2016).
8. Coventry, B. & Baker, D. Protein sequence optimization with a pairwise decomposable penalty for buried unsatisfied hydrogen bonds. *PLOS Comput. Biol.* **17**, e1008061 (2021).
9. Hiranuma, N. *et al.* Improved protein structure refinement guided by deep learning based accuracy estimation. *Nat. Commun.* **12**, 1340 (2021).
10. Correnti, C. E. *et al.* Engineering and functionalization of large circular tandem repeat protein nanoparticles. *Nat. Struct. Mol. Biol.* **27**, 342–350 (2020).
11. Rocklin, G. J. *et al.* Global analysis of protein folding using massively parallel design, synthesis and testing. *Science* **357**, 168–175 (2017).
12. Chen, Z. *et al.* Programmable design of orthogonal protein heterodimers. *Nature* **565**, 106–111 (2019).

13. Brunette, T. J. *et al.* Exploring the repeat protein universe through computational protein design. *Nature* **528**, 580–584 (2015).

14. Hsia, Y. *et al.* Design of multi-scale protein complexes by hierarchical building block fusion. *Nat. Commun.* **12**, 2294 (2021).

15. Cao, L. *et al.* Design of protein-binding proteins from the target structure alone. *Nature* **605**, 551–560 (2022).
