# Supplementary material for: Design of pseudosymmetric protein hetero-oligomers
Source: Nat Commun. 2024 Dec 18;15:10684. doi: 10.1038/s41467-024-54913-8 (PMC11655659; doi:10.1038/s41467-024-54913-8)
Supplement: Supplementary file 5 [file 41467_2024_54913_MOESM2_ESM.pdf]

Description of Additional Supplementary Files

File name: Supplementary Data 1
Description: BGL homotrimer sequences.

File name: Supplementary Data 2
Description: Experimental result summary for BGL homotrimers.

File name: Supplementary Data 3
Description: SAXS analyses for BGL homotrimers.

File name: Supplementary Data 4
Description: BGL heterotrimer sequences.

File name: Supplementary Data 5
Description: Experimental result summary for BGL heterotrimers.

File name: Supplementary Data 6
Description: SAXS analyses for BGL heterotrimers.

File name: Supplementary Data 7
Description: RTR homo-oligomer sequences.

File name: Supplementary Data 8
Description: Experimental result summary for RTR homo-oligomers.

File name: Supplementary Data 9
Description: SAXS analyses for RTR homo-oligomers.

File name: Supplementary Data 10
Description: A2B2-hetRTR sequences.

File name: Supplementary Data 11
Description: Experimental result summary for RTR hetero-oligomers.

File name: Supplementary Data 12
Description: A3B3/A2B2C2-hetRTR sequences.

File name: Supplementary Data 13
Description: Native MS spectra of all designs.
